# Transcriptomic and metabolomic analyses identify a role for chlorophyll catabolism and phytoalexin during *Medicago* nonhost resistance against Asian soybean rust

**DOI:** 10.1038/srep13061

**Published:** 2015-08-12

**Authors:** Yasuhiro Ishiga, Srinivasa Rao Uppalapati, Upinder S. Gill, David Huhman, Yuhong Tang, Kirankumar S. Mysore

**Affiliations:** 1 Plant Biology Division, The Samuel Roberts Noble Foundation, Ardmore, OK 73401, USA; 2 Faculty of Life and Environmental Sciences, University of Tsukuba, 1-1-1 Tennodai, Tsukuba, Ibaraki 305-8572, Japan

## Abstract

Asian soybean rust (ASR) caused by *Phakopsora pachyrhizi* is a devastating foliar disease affecting soybean production worldwide. Understanding nonhost resistance against ASR may provide an avenue to engineer soybean to confer durable resistance against ASR. We characterized a *Medicago truncatula-*ASR pathosystem to study molecular mechanisms of nonhost resistance. Although urediniospores formed appressoria and penetrated into epidermal cells of *M. truncatula*, *P. pachyrhizi* failed to sporulate. Transcriptomic analysis revealed the induction of phenylpropanoid, flavonoid and isoflavonoid metabolic pathway genes involved in the production of phytoalexin medicarpin in *M. truncatula* upon infection with *P. pachyrhizi*. Furthermore, genes involved in chlorophyll catabolism were induced during nonhost resistance. We further characterized one of the chlorophyll catabolism genes, *Stay-green* (*SGR*), and demonstrated that the *M. truncatula sgr* mutant and alfalfa *SGR*-RNAi lines showed hypersensitive-response-like enhanced cell death upon inoculation with *P. pachyrhizi*. Consistent with transcriptomic analysis, metabolomic analysis also revealed the accumulation of medicarpin and its intermediate metabolites. *In vitro* assay showed that medicarpin inhibited urediniospore germination and differentiation. In addition, several triterpenoid saponin glycosides accumulated in *M. truncatula* upon inoculation with *P. pachyrhizi*. In summary, using multi-omic approaches, we identified a correlation between phytoalexin production and *M. truncatula* defense responses against ASR.

Even though plants are immune to almost all pathogens in nature, enormous losses in crop production occur due to specific pathogen infestation in agricultural fields^1^. Plants have developed many strategies to defend themselves against pathogens. The first line of defense is the recognition of conserved molecules from pathogens, called pathogen-associated molecular patterns (PAMPs), by plant pattern-recognition receptors, resulting in PAMP-triggered immunity (PTI)^2^. The second line of defense that exists to defend plants from pathogens is effector-triggered immunity (ETI) or host-specific resistance based on the gene-for-gene interaction which is governed by individual plant resistance (*R*) genes and their corresponding avirulence (*Avr*) genes^3^. Breeding for disease resistance in crop plants has mainly relied on introducing *R* genes by crossing cultivated species with closely related wild species that are resistant to certain diseases. However, the resistant varieties produced by such an approach are not always durable. The pathogen can evolve to overcome the resistance by mutations in the Avr protein^4^. For example, wheat varieties that were resistant for over 50 years to wheat stem rust caused by *Puccinia graminis f. sp. tritici* now have compromised resistance against a new race of stem rust, Ug99, after it was first reported in Uganda in 1999^5^. The Ug99 strain of *P. graminis* has become a potential threat to worldwide wheat cultivation^6^. In addition to wheat stem rust, other important diseases such as late blight of potato, banana black sigatoka disease, Asian soybean rust (ASR), rice blast and cassava streak virus are a threat to global food security^1^. Therefore, for the stability of worldwide food supply, there is a need for durable and sustainable resistance against important diseases^7^,^8^.

Pathogen outbreaks in important crop plants could have a significant negative impact on human life^9^. For example, the outbreak of ASR, caused by *Phakopsora pachyrhizi*, impacted the economy of South America in recent years^10^. It has been reported that the losses caused by ASR were ~2 billion US dollars in Brazil alone in 2003^10^. Unlike wheat rust fungus that has high host specificity, the host range of ASR is broad^11^. The *P. pachyrhizi* fungus, belonging to Basidiomycetes, forms uredinia five to eight days after infection and then produces urediniospores by asexual reproduction^12^. Urediniospores can be dispersed by wind and germinate on the host plant to form appressoria. Unlike the cereal rust fungus that penetrates through stomata^13^, *P. pachyrhizi* directly penetrates into the epidermal cells of host plants. After penetration, *P. pachyrhizi* extends the infection hyphae and forms haustoria (feeding structures) in the mesophyll cells. Generally, haustoria formation is observed 24–48 hours after infection^14^. Six soybean accessions that are resistant to a particular *P. pachyrhizi* race have been identified, and the *R* genes (*Rpp1–6*) that confer resistance have also been identified^15^,^16^,^17^. However, none of the soybean accessions in the world show resistance to all races of *P. pachyrhizi*^18^. Therefore, timely application of fungicides is the only means to control ASR^19^.

Nonhost resistance, contrary to *R*-gene-mediated, host-specific resistance, is believed to be more robust and universal in plant defense systems^20^,^21^,^22^. In studies based on interactions between the barley powdery mildew pathogen and a nonhost plant, Arabidopsis, *PEN* (*PENETRATION*) genes of Arabidopsis involved in nonhost resistance have been identified^23^,^24^,^25^. *PEN* genes are essential for nonhost resistance, which prevents penetration by certain potentially pathogenic filamentous fungi^26^,^27^,^28^. These Arabidopsis *PEN* genes are reported to also be essential for nonhost resistance against ASR^29^,^30^. *PEN*-mediated resistance is broadly involved in nonhost pre-invasive defense against fungal pathogens^24^. Enhanced penetration was observed in the Arabidopsis *pen3* mutant, suggesting pre-invasive defense in Arabidopsis as a component of nonhost resistance against ASR^30^. The Arabidopsis *pen3* mutant did not completely compromise nonhost resistance against ASR, due to active apoplastic defense. Genetic studies using Arabidopsis to identify components of apoplastic defense against ASR will be challenging since the studies have to be done in the *pen3* mutant background. Furthermore, based on analysis using the double mutants of salicylic acid (SA) or jasmonic acid (JA) signaling pathways in the *pen3* mutant background, it has been suggested that both SA and JA signaling pathways are involved in nonhost resistance of Arabidopsis against ASR^30^. However, molecular mechanisms of nonhost post-invasive defense against fungal pathogens are still unclear.

In this study, we have characterized *Medicago truncatula* as a model nonhost pathosystem for ASR to investigate the molecular mechanisms of nonhost resistance. Based on transcriptome and metabolome analyses, we show the importance of phytoalexins such as isoflavonoids in *M. truncatula* to confer nonhost resistance against ASR. In addition, we demonstrate that the *M. truncatula* chlorophyll catabolism pathway genes were differently regulated during nonhost resistance to ASR and one of chlorophyll catabolism pathway mutant *stay-green* (*sgr*) has enhanced defense response to ASR.

## Results

### *Medicago truncatula* exhibits nonhost resistance to ASR

**Figure 1:**
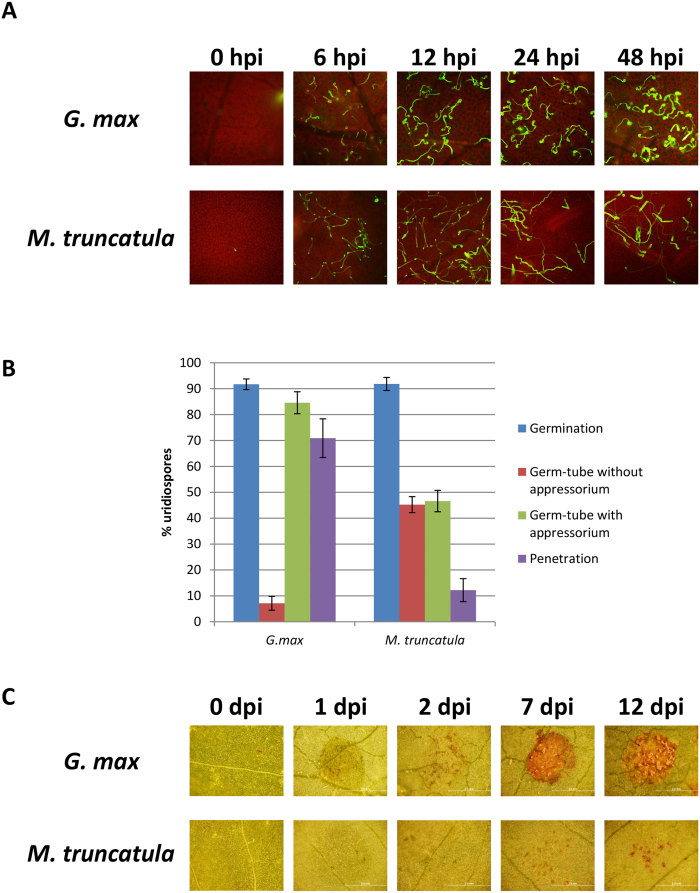
Disease and resistance phenotypes of soybean (*Glycine max*) and *M. truncatula* leaves inoculated with *P. pachyrhizi*. (**A**) Epifluorescence micrographs of *P. pachyrhizi* on abaxial leaf surfaces of soybean (*G. max* cv. Williams) and *M. truncatula* wild-type R108 at 6, 12, 24 and 48 hours post-inoculation (hpi). The 4-week-old soybean and *M. truncatula* plants were spray-inoculated with 1 × 10^5^ spores/ml (0.001% Tween 20) of *P. pachyrhizi* using an artist air-brush. For fluorescence microscopy, fungal mycelia in the inoculated leaves were stained with 10 μg/mL WGA-Alexa Fluor 488 for 20 min at room temperature. After washing with PBS, the leaves were observed using an epifluorescence microscope. (**B**) Quantification of germination and pre-infection structures of *P. pachyrhizi* on abaxial leaf surfaces of soybean (*G. max* cv. Williams) and *M. truncatula* wild-type R108 at 72 hpi. Four-week-old soybean and *M. truncatula* plants were spot-inoculated with 10 μl of 1 × 10^4^ spores/ml (0.001% Tween 20) of *P. pachyrhizi*. The formation of pre-infection structures was counted from 20 random fields on three independent leaves. The number of dead autofluorescing epidermal cells resulting from direct penetration of *P. pachyrhizi* were counted from 20 random fields per each inoculated site and are used to calculate the percentage of penetration. The experiments were repeated at least 3 times. (**C**) Disease symptom development of soybean (*G. max* cv. Williams) and *M. truncatula* abaxial leaf surfaces inoculated with *P. pachyrhizi* at 1, 2, 7 and 12 days post-inoculation (dpi). Four-week old soybean and *M. truncatula* plants were spot-inoculated on the adaxial side of the leaf with 10 μl of 1 × 10^4^ spores/ml (0.001% Tween 20) of *P. pachyrhizi*. The inoculated leaves were maintained in a dew chamber for 24 hrs with 100% humidity maintained at 19 °C; 0-hrs-light/24-hrs-dark cycle. The leaves were then transferred to a growth chamber (22 °C/19 °C with 12 hrs-light/12 hrs-dark cycle) and incubated further to allow symptom development. Photographs were taken at time points indicated.

To investigate the molecular mechanisms of nonhost resistance, especially apoplastic defense, against ASR, we decided to use the model legume *M. truncatula* since *P. pachyrhizi* can penetrate *M. truncatula*. Furthermore, identification of genes in *M. truncatula* that confer nonhost resistance against ASR will provide an avenue to engineer soybean for durable resistance against ASR. To assess the feasibility of using *M. truncatula* as a model pathosystem to study nonhost resistance, leaves of both soybean (host) and *M. truncatula* (nonhost) were inoculated with urediniospores of ASR. In soybean, around 90% of urediniospores germinated, and 70% of them penetrated the epidermal tissue within 72 hours post inoculation (hpi). Meanwhile, although around 90% of urediniospores also germinated in *M. truncatula*, ~15% of them penetrated the epidermis (Fig. 1A,B), suggesting a weaker pre-invasive nonhost defense in *M. truncatula* against ASR when compared to Arabidopsis. In soybean, uredinia formation was observed within seven days post inoculation (dpi), and they produced urediniospores (Fig. 1C). However, *M. truncatula* did not produce urediniospores but produced localized cell death at the penetration site, suggesting an active apoplastic nonhost defense against ASR (Fig. 1C). Together, these results suggest that *M. truncatula* is a suitable model to study nonhost resistance against ASR.

### Transcriptome analysis identifies a correlation between phytoalexin production and *M. truncatula* defense responses against ASR

To further investigate the molecular mechanism of nonhost resistance of *M. truncatula* against ASR, we performed integrated transcriptome and metabolome analyses using samples derived from *M. truncatula* leaves inoculated with *P. pachyrhizi*. To identify host signaling pathways triggered by *P. pachyrhizi* infection, we carried out microarray analysis to monitor the expression profiles associated with nonhost resistance, including pre-invasive (12 hpi) and apoplastic defense (24 hpi), using Affymetrix GeneChip® Medicago Genome Array (Affymetrix). Using a Bonferroni corrected *P* value threshold of 1.1406e-006 and a threefold ratio cutoff, 2,704 and 3,386 genes were identified as induced or repressed, respectively, at 12 hpi when compared with the mock controls (Supplementary Figs S1 and S2; Supplementary Tables S1 and S2). Furthermore, at 24 hpi, 2,809 and 3,504 genes were identified as induced or repressed, respectively (Supplementary Figs S1 and S2; Supplementary Tables S3 and S4). ASR-regulated *M. truncatula* genes were classified into different functional groups using the new BIN structure developed by Goffard and Weiller^31^.

**Figure 2:**
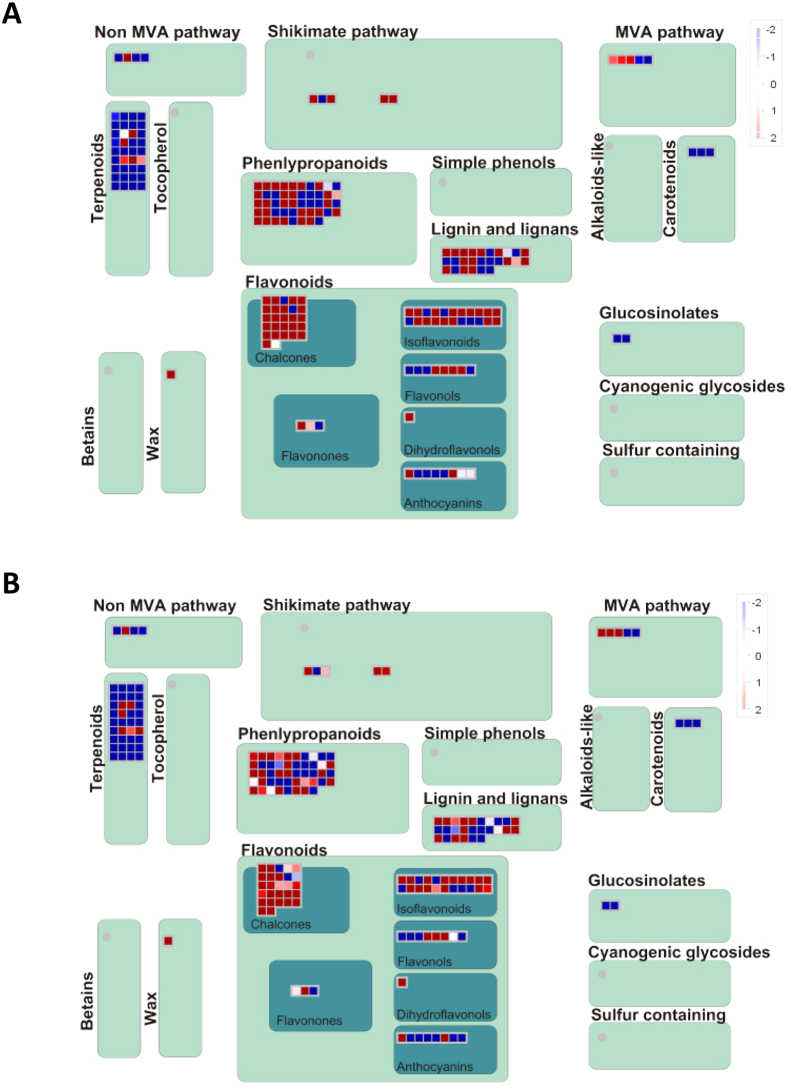
MAPMAN illustration of *M. truncatula* Affymetrix data showing the genes involved in secondary metabolism during *M. truncatula*-*P. pachyrhizi* interactions at 12 **(A) and 24 (B) hours post-inoculation (hpi).** Four-week old *M. truncatula* plants were spray-inoculated with 1 × 10^5^ spores/ml (0.001% Tween 20) of *P. pachyrhizi*; total RNA was isolated from leaves inoculated with *P. pachyrhizi* at 12 and 24 hpi, or mock treatment. Three biological replicates were used for each inoculation. Each biological replicate consisted of a pool of eight plants. Red indicates upregulation, and blue indicates downregulation.

MAPMAN software was used to obtain an overview of genes belonging to various metabolic pathways in the nonhost resistance of *M. truncatula* to ASR (Fig. 2; Supplementary Figs S3 and S4). Using automated and manual annotation, genes represented in the *M. truncatula* microarray were classified into different functional groups and assigned BIN numbers. MAPMAN analysis revealed that there are no significant differences in primary metabolism pathways among the genes differentially regulated between 12 h and 24 h after inoculation with ASR (Fig. 2; Supplementary Figs S1, S2, S3 and S4). Analysis of gene expression profiles between *P. pachyrhizi*-infected and mock-inoculated *M. truncatula* leaves revealed that 149 and 160 genes (around 5%) belonging to secondary metabolism were induced at 12 or 24 hpi, respectively. (Supplementary Figs S1, S2 and S3). In addition, the genes involved in photosynthesis, including light reactions and photorespiration, were significantly suppressed during nonhost interactions (Supplementary Figs S1, S2 and S3).

Several genes related to phytohormones including salicylic acid (SA), jasmonic acid (JA), ethylene (ET), auxin, cytokinin and gibberellin were differently regulated during nonhost interactions (Supplementary Fig. S4; Supplementary Tables S5, S6, S7 and S8). Several SA-regulated *Pathogenesis-related* (*PR*) genes were also induced during nonhost resistance (Supplementary Tables S5 and S7). Further, several genes involved in ethylene biosynthesis were induced during nonhost interactions (Supplementary Tables S5 and S7). These genes included *1-Aminocyclopropane-1-carboxylate synthase* (*ACC synthase*) and *1-Aminocyclopropane-1-carboxylate oxidase* (*ACC oxidase*). *P. pachyrhizi* also induced the expression of genes encoding ET-responsive transcription factors (AP2/ERF family members) (Supplementary Fig. S4; Supplementary Tables S6 and S7). In addition, several genes involved in JA biosynthesis were induced during nonhost interactions. These genes included *Lipoxygenases* (*LOX*), *Allene oxide synthase* (*AOS*) and *12-Oxophytodienoic acid 10,11-reductase* (*OPR*) (Supplementary Fig. S1). However, the expression of several other members of JA-related genes including *LOX*, *Allene oxide cyclase* (*AOC*) and *OPR* were downregulated during nonhost resistance (Supplementary Fig. S2; Supplementary Tables S6 and S8). These results indicate a correlation between up-regulation of SA-, ET- and JA-mediated signaling pathways and nonhost resistance of *M. truncatula* against ASR.

**Figure 3:**
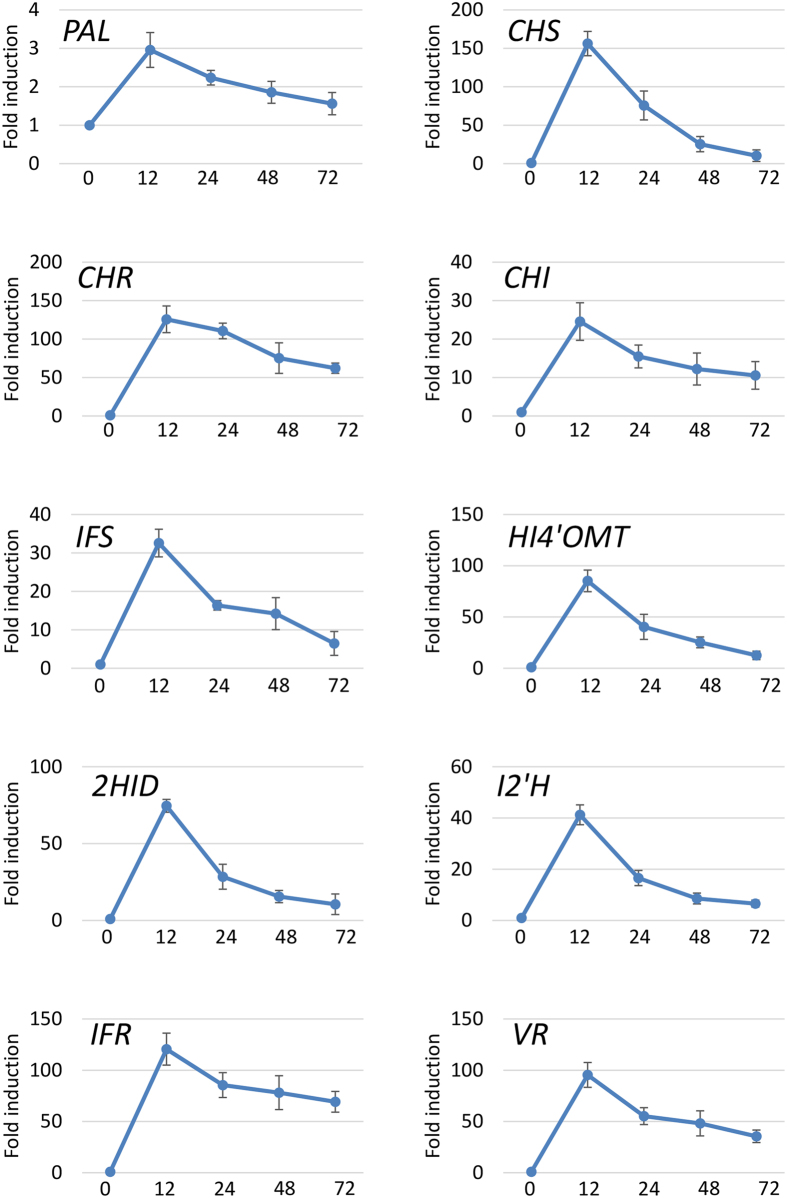
Expression profiles of genes involved in phenylpropanoid and flavonoid pathways in *M. truncatula* inoculated with *P. pachyrhizi*. *M. truncatula* leaves were inoculated with *P. pachyrhizi* at 12, 24, 48 and 72 hours post-inoculation (hpi) or mock control (0 time). Expression profiles are presented for genes encoding Phenylalanine ammonia-lyase (*PAL*), Chalcone synthase (*CHS*), Chalcone reductase (*CHR*), Chalcone isomerase (*CHI*), Isoflavone synthase (*IFS*), Isoflavone 4′-O-methyltransferase (*HI4′OMT*), 2-Hydroxyisoflavanone dehydratase (*2HID*), Isoflavone 2′- hydroxylase (*I2′H*), Isoflavone reductase (*IFR*) and Vestitone reductase (*VR*). The expression of genes was evaluated by RT-qPCR with gene-specific primer sets (Supplementary Table S10). The values represent the fold induction compared to mock control. Three biological replicates were used for each inoculation. The *Ubiquitin* gene was used as an internal control. Bars represent the means ± standard deviation (SD).

As shown in Fig. 2 and Supplementary Figs S1 and S2, microarray analysis revealed a significant induction of transcripts derived from genes encoding proteins associated with phenylpropanoid, flavonoid and isoflavonoid metabolic pathways in *M. truncatula* leaves at 12 and 24 hpi with *P. pachyrhizi* (Fig. 2). Interestingly, the genes involved in the phytoalexin production pathway were induced and the genes involved in the terpenoid production pathway were suppressed in response to *P. pachyrhizi* inoculation (Fig. 2). Furthermore, real-time quantitative RT-PCR (RT-qPCR) analysis confirmed the induction of genes involved in the phytoalexin medicarpin metabolic pathway, including *Phenylalanine ammonia-lyase* (*PAL*), *Chalcone synthase* (*CHS*), *Chalcone reductase* (*CHR*), *Chalcone isomerase* (*CHI*), *Isoflavone synthase* (*IFS*), *Isoflavone 4′-O-methyltransferase* (*HI4′OMT*), *2-Hydroxyisoflavanone dehydratase* (*2HID*), *Isoflavone 2′-hydroxylase* (*I2′H*), *Isoflavone reductase* (*IFR*) and *Vestitone reductase* (*VR*) (Fig. 3). The expression profiles of these genes in response to *P. pachyrhizi* inoculation were almost identical. The transcripts of these genes were significantly induced within 12 hpi and slightly decreased until 72 hpi with *P. pachyrhizi*. These results indicate a correlation between phytoalexin medicarpin pathway and nonhost resistance against ASR.

### The chlorophyll catabolism pathway is upregulated by *P. pachyrhizi* infection

**Figure 4:**
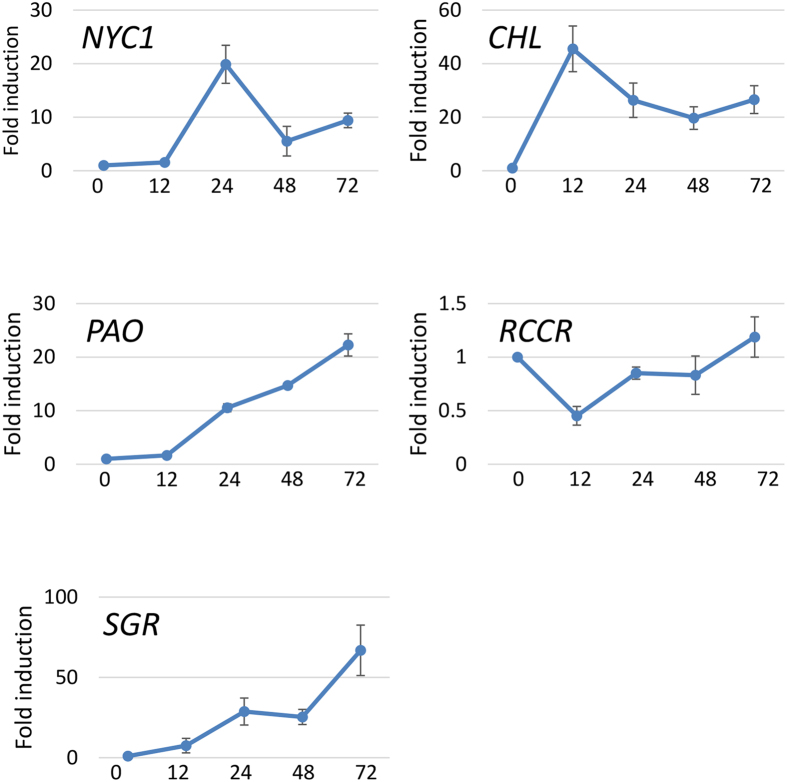
Expression profiles of genes involved in the chlorophyll catabolism pathway in *M. truncatula* inoculated with *P. pachyrhizi*. *M. truncatula* leaves were inoculated with *P. pachyrhizi* at 12, 24, 48 and 72 hours post-inoculation (hpi) or mock control (0 time). Expression profiles are presented for genes encoding Chlorophyll b reductase (*NYC1*), Chlorophyllase (*CHL*), Pheophorbide A oxygenase (*PAO*), Red chlorophyll catabolite reductase (*RCCR*) and Stay-green (*SGR*). The expression of genes was evaluated by RT-qPCR with gene-specific primer sets (Supplementary Table S10). The values represent the fold induction compared to mock control. Three biological replicates were used for each inoculation. The *Ubiquitin* gene was used as an internal control. Bars represent the means ± standard deviation (SD).

Transcriptome analysis revealed downregulation of a large number of genes involved in photosynthesis in response to *P. pachyrhizi* inoculation (Supplementary Figs S2 and S3). At the same time, many genes with exception of *Red chlorophyll catabolite reductase* (*RCCR*) that encode enzymes involved in the chlorophyll catabolism pathway, including *Chlorophyll b reductase* (*NYC1*), *Chlorophyllase* (*CHL*), *Pheophorbide a oxygenase* (*PAO*) and *Stay-green* (*SGR*), were upregulated in response to *P. pachyrhizi* inoculation (Supplementary Table. S9). Previous studies have demonstrated an important link between light and disease resistance, based on the mutants involved in the chlorophyll degradation pathway in Arabidopsis^32^,^33^. To further investigate the molecular mechanism of the chlorophyll catabolism pathway in nonhost resistance of *M. truncatula* against ASR, expression analysis of *NYC1*, *CHL*, *PAO*, *RCCR* and *SGR* was performed by RT-qPCR. Consistent with the microarray results (Supplementary Table S9), the expression of *NYC1*, *CHL*, *PAO* and *SGR* was significantly induced by ASR (Fig. 4). Interestingly, the expression of *RCCR* was suppressed 12 hours after inoculation with ASR (Fig. 4; Supplementary Table S9). Among these genes, *NYC1*, *CHL*, *PAO* and *RCCR* are known to encode enzymes involved in chlorophyll degradation^34^. Although *SGR* is believed to regulate the enzymes involved in chlorophyll degradation and involved in disease symptom development^35^,^36^,^37^, the precise function of *SGR* in plant defense is still unknown. Therefore, we decided to further characterize the role of *SGR* in nonhost resistance against ASR.

### *M. truncatula sgr* mutant shows enhanced defense responses to ASR

**Figure 5:**
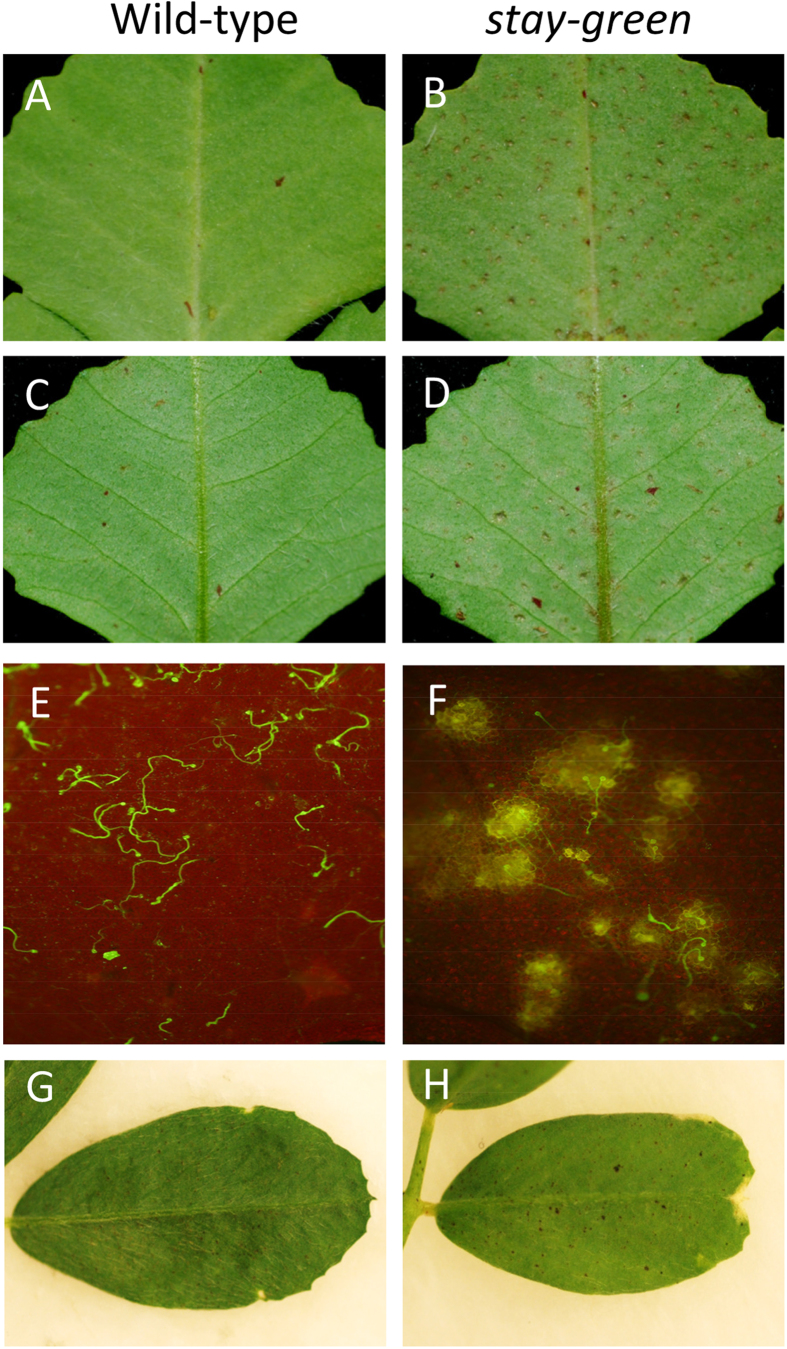
Response of *M. truncatula* wild-type, *stay-green* (*sgr*) mutant, alfalfa wild-type and alfalfa *SGR*-RNAi line to *P. pachyrhizi*. (**A**–**D**) Response of *M. truncatula* wild-type R108 (**A**,**C**) and *sgr* mutant (**B**,**D**) to *P. pachyrhizi* at 48 hours post-inoculation (hpi) on adaxial (**A**,**B**) or abaxial (**C**,**D**) leaf surfaces. Four-week old *M. truncatula* wild-type R108 and *sgr* mutant were spray-inoculated with 1 × 10^5^ spores/ml (0.001% Tween 20) of *P. pachyrhizi* using an artist air-brush. (**E**,**F**) Epifluorescence micrographs of WGA-Alexa Fluor 488-stained pre-infection structures of *P. pachyrhizi* on abaxial leaf surfaces of *M. truncatula* wild-type R108 (**E**) or *sgr* mutant (**F**) at 48 hpi. Fungal mycelia in the inoculated leaves were stained with 10 μg/mL WGA-Alexa Fluor 488 for 20 min at room temperature. After washing with PBS, the leaves were observed using an epifluorescence microscope. (**G**,**H**) Response of alfalfa wild-type Regan SY4D (**G**) or alfalfa *SGR*-RNAi line (**H**) to *P. pachyrhizi* at 48 hours post-inoculation (hpi) on abaxial leaf surfaces.

To investigate the functional role of *SGR* in nonhost resistance of *M. truncatula* against ASR, we inoculated the previously characterized *sgr* mutant^38^ and its corresponding wild type (R108) with *P. pachyrhizi* and observed for symptom development. Previous study has demonstrated that dark-induced senescence was delayed in the *M. truncatula sgr* mutant^38^. As shown in Fig. 5 and as expected, no visible symptoms were observed on both adaxial (Fig. 5A) and abaxial (Fig. 5C) leaf surfaces of wild-type R108 at 48 hpi with *P. pachyrhizi* (Fig. 5E). Wild-type *M. truncatula* produces HR-like cell death at the penetration site only seven days after inoculation (Fig. 1C). Interestingly, necrotic symptoms that look like hypersensitive response (HR) cell death were observed in the *sgr* mutant 48 hpi with *P. pachyrhizi* (Fig. 5B,D). Microscopic observation also showed autofluorescence around the *P. pachyrhizi* penetration site in the *sgr* mutant (Fig. 5F).

To further confirm the HR cell death phenotype of *sgr*, we utilized another nonhost plant, alfalfa (*Medicago sativa*) which is a close relative of *M. tuncatula*. RNAi lines of alfalfa that downregulates *SGR* has been previously developed and characterized for the *sgr* phenotype^38^. Wild-type alfalfa (Regan SY4D) and *SGR*-RNAi lines were inoculated with *P. pachyrhizi*. As expected, no visible symptoms were observed on abaxial leaf surfaces of wild-type alfalfa at 48 hpi with *P. pachyrhizi* (Fig. 5G). Consistent with the phenotype of *M. truncatula sgr* mutant, necrotic symptoms were observed in the *SGR*-RNAi lines 48 hpi with *P. pachyrhizi* (Fig. 5H). Together, these results indicate that *sgr* mutant and *SGR*-RNAi lines have enhanced defense responses against ASR infection.

**Figure 6:**
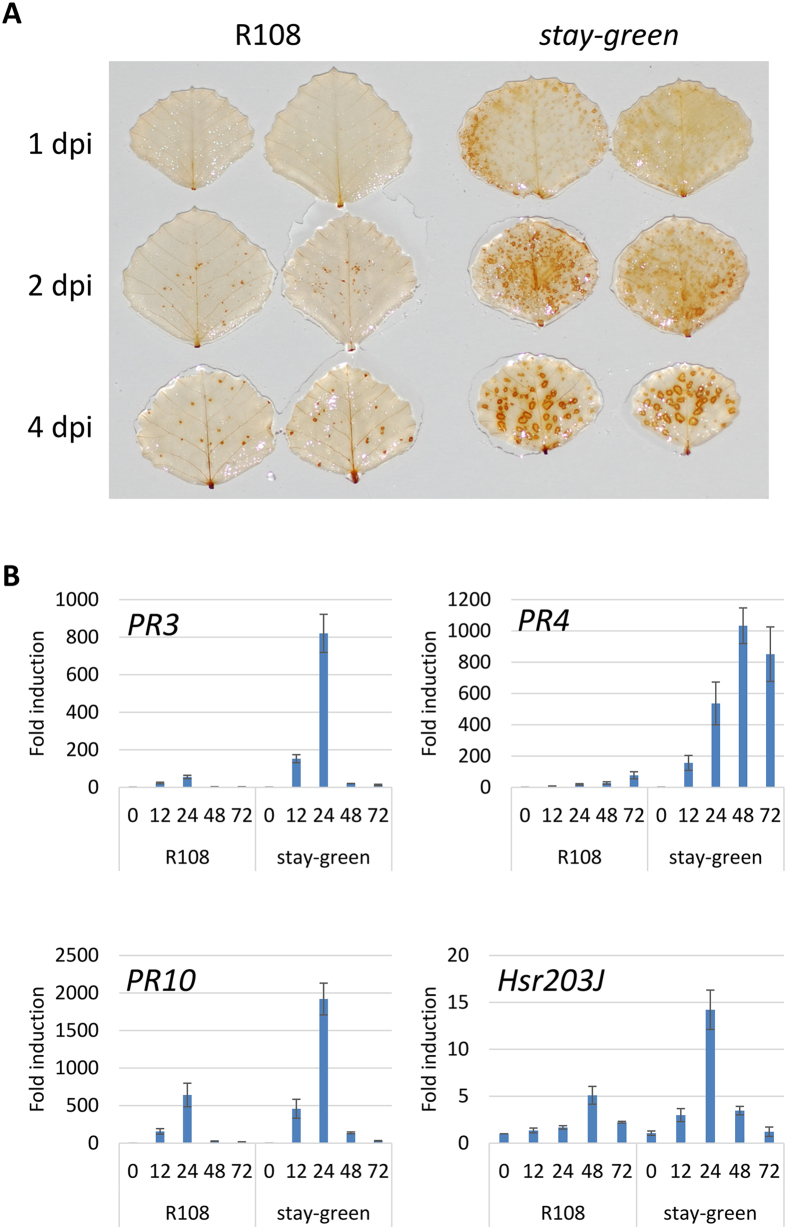
The production of reactive oxygen species and gene expression profiles of *M. truncatula* wild-type R108 and the *stay-green* (*sgr*) mutant to *P. pachyrhizi*. (**A**) 3,3-diaminobenzidine (DAB) staining for detection of hydrogen peroxide. Four-week-old *M. truncatula* wild-type R108 and *sgr* mutant were spray-inoculated with 1 × 10^5^ spores/ml (0.001% Tween 20) of *P. pachyrhizi*, then incubated with DAB for 1, 2 and 4 days post-inoculation (dpi). (**B**) Expression profiles of genes involved in defense responses in *M. truncatula* inoculated with *P. pachyrhizi*. *M. truncatula* leaves spray-inoculated with *P. pachyrhizi* were sampled at 12, 24, 48 and 72 hours post-inoculation (hpi) or mock control (0 time). Expression profiles are presented for genes encoding Pathogenesis-related protein 3 (*PR3*), Pathogenesis-related protein 4 (*PR4*), pathogenesis-related protein 10 (*PR10*) and *Hsr203J*. The expression of genes was evaluated by RT-qPCR with gene-specific primer sets (Supplementary Table S10). The values represent the fold induction compared to mock control. Three biological replicates were used for each inoculation. The *Ubiquitin* gene was used as an internal control. Bars represent the means ± standard deviation (SD).

To further study the defense responses of the *sgr* mutant against *P. pachyrhizi*, we checked the accumulation of reactive oxygen species (ROS) and the expression of defense-related genes in the *sgr* mutant and its wild-type R108. To visualize the accumulation of ROS, especially hydrogen peroxide, we stained the inoculated leaves with DAB (3,3′-Diaminobenzidine). As shown in Fig. 6A, a slight accumulation of hydrogen peroxide was observed as brown-colored spots around the penetration sites at 2 dpi in wild type (Fig. 6A). Strikingly, a significantly greater accumulation of hydrogen peroxide was observed within 1 dpi in the *sgr* mutant when compared to wild type, and it gradually increased up to 4 dpi suggesting that the *sgr* mutant accumulates more ROS compared to wild type in response to *P. pachyrhizi* inoculation (Fig. 6A). Although the expression of *PR* genes, including *PR3*, *PR4* and *PR10*, was induced in response to *P. pachyrhizi* in both wild type and the *sgr* mutant, the induction levels of these genes were significantly higher in the *sgr* mutant when compared to the wild type (Fig. 6B). The expression of *M. truncatula Hsr203J*, an orthologous gene for tobacco *Hsr203J* whose expression is rapidly induced in HR cell death, was highly (~14-fold) induced 24 hrs after inoculation with *P. pachyrhizi* in the *sgr* mutant (Fig. 6B). In contrast, the *Hsr203J* induction was delayed in the wild type by 24 hrs when compared to the *sgr* mutant and was induced only ~five-fold after inoculation with *P. pachyrhizi* (Fig. 6B). Together, these results suggest that the *sgr* mutant has enhanced defense responses against ASR.

### SGR plays an important role for chlorosis symptom development

Previous studies have shown that *Arabidopsis* STAYGREEN has an important role for disease symptom development, especially chlorosis, for both bacterial and fungal pathogens including *Pseudomonas syringae* pv. *tomato* DC3000 and *Alternaria brassicicola*^34^. To investigate the molecular basis of STAYGREEN in disease development in legumes, we inoculated *M. truncatula sgr* mutant and alfalfa *SGR*-RNAi lines^38^ with an anthracnose pathogen, *Colletotrichum trifolii*. Consistent with the previous report in *Arabidopsis*^34^, chlorosis symptom development in *M. truncatula sgr* mutant and alfalfa *SGR*-RNAi lines after inoculation with *C. trifolii* was delayed compared with wild type (Supplementary Fig. S5). These results indicate that *M. truncatula* SGR also has a role for disease development, especially chlorosis symptoms.

### Metabolome analysis identifies a correlation between phytoalexins and *M. truncatula* defense responses against ASR

**Figure 7:**
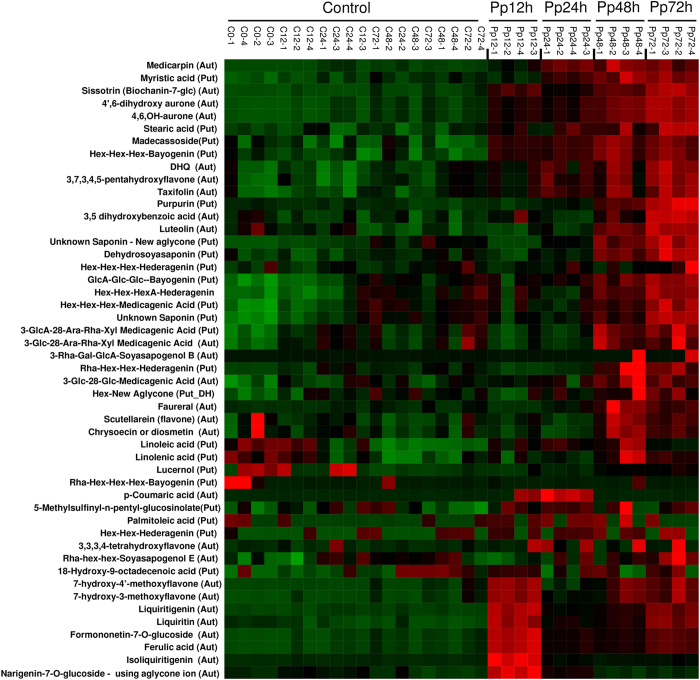
Heat map illustration of metabolites during *M. truncatula*-*P. pachyrhizi* interactions at 12, 24, 48 and 72 hours post-inoculation (hpi). Four-week-old soybean and *M. truncatula* plants were spray-inoculated with 1 × 10^5^ spores/ml (0.001% Tween 20) of *P. pachyrhizi*. Total metabolites derived from *P. pachyrhizi*-inoculated leaves at 12, 24, 48 and 72 hpi, and water-treated controls were isolated in 80% methanol. The metabolites were quantified by UPLC system fitted with a hybrid quadrupole time-of-flight (QTOF) Premier mass spectrometer. Four biological replicates were used for each inoculation. Red indicates upregulation, and green indicates downregulation. Put and Aut mean compound confirmation as putative and authentic, respectively.

Transcriptome analysis revealed a significant induction of genes involved in phytoalexin medicarpin pathways in *M. truncatula* at 12 and 24 hpi with *P. pachyrhizi* (Figs 2 and 3; Supplementary Fig. S3). To investigate whether *M. truncatula* accumulated the phytoalexin medicarpin and intermediate metabolites such as isoflavones and flavones during nonhost resistance response against ASR, metabolome analysis was carried out using total metabolites derived from *P. pachyrhizi*-inoculated *M. truncatula* leaves at 12, 24, 48 and 72 hpi, and mock-inoculated controls. Consistent with the transcriptome analysis, a significant accumulation of intermediate metabolites such as isoflavones and flavones, including narigenin-7-O-glucoside, formononetin-7-O-glucoside, liquiritin, liquiritigenin, 7-hydroxy-3-methoxyflavone and 7-hydroxy-4′-methoxyflavone was observed 12 hpi with *P. pachyrhizi* when compared to the control (Fig. 7). Interestingly, their induction levels decreased 12 hpi, whereas the accumulation of medicarpin was observed at 24, 48 and 72 hpi (Fig. 7). Taken together with the transcriptome data, these results suggest a correlation between isoflavonoid metabolic pathways including the medicarpin pathway and nonhost resistance against ASR.

In addition to phytoalexins, metabolome analysis identified a significant accumulation of triterpenoid saponins, such as madecassoside, Hex-Hex-Hex-bayogenin, dehydrosoyasaponin, Hex-Hex-Hex-hederagenin, GlcA-Glc-Glc-bayogenin, Hex-Hex-HexA-hederagenin, Hex-Hex-Hex-medicagenic acid, 3-GlcA-28-Ara-Rha-Xyl medicagenic acid, 3-Glc-28-Ara-Rha-Xyl medicagenic acid, 3-Rha-Gal-GlcA-soyasapogenol B, Rha-Hex-Hex-hederagenin and 3-Glc-28-Glc-medicagenic acid, mainly at 72 hpi with *P. pachyrhizi* (Fig. 7). We understand very little about the role of saponins in nonhost resistance.

### *M. truncatula* phytoalexin medicarpin and its intermediate formononetin-7-O-glucoside suppress the pre-infection structure formation of *P. pachyrhizi*

**Figure 8:**
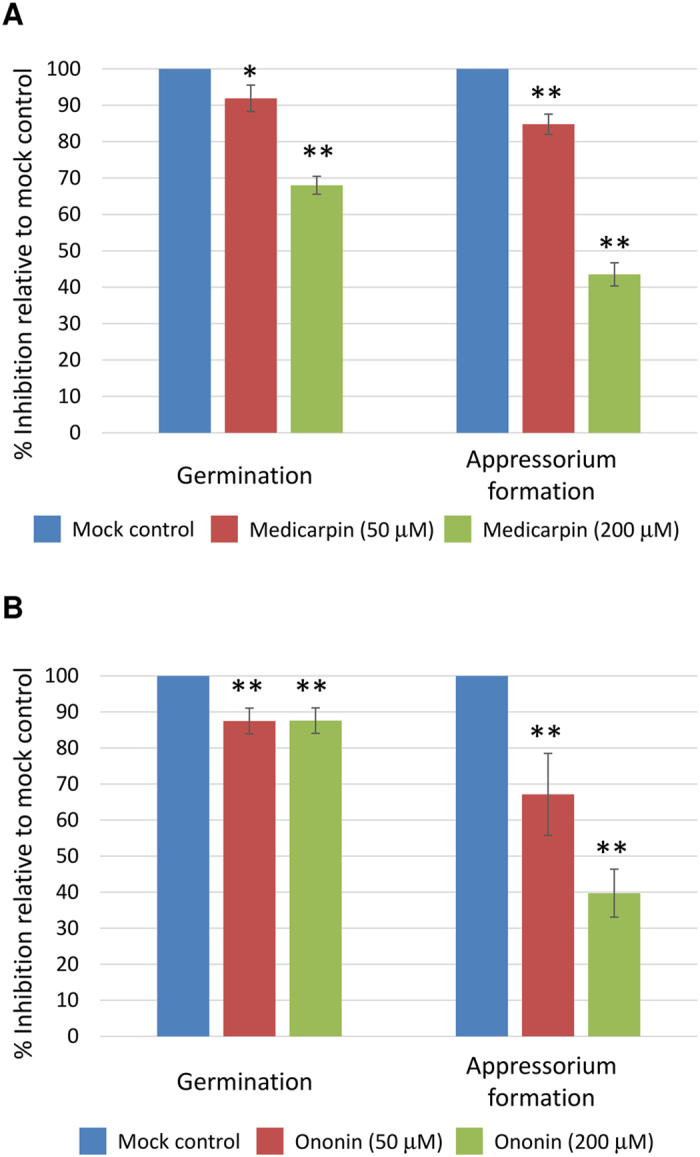
Effect of phytoalexin medicarpin and its intermediate formononetin-7-O-glucoside (ononin) on differentiation of urediniospores of *P. pachyrhizi*. *P. pachyrhizi* urediniospores were incubated with DMSO (mock control) or medicarpin or ononin to observe pre-infection structure formation. The percentage of germination and appressorium formation were counted 18 hours after treatment. Bars represent the means ± standard deviation (SD) of eight replications from two independent experiments for each data point. Asterisks indicate significant difference evaluated using paired Student’s *t* test (*P < 0.05, **P < 0.01).

Transcriptome and metabolome analyses revealed a significant induction of phytoalexin medicarpin pathway during nonhost resistance of *M. truncatula* against *P. pachyrhizi* (Figs 2,3 and 7; Supplementary Fig. S3). To further confirm the importance for phytoalexin during nonhost resistance, we studied germination and differentiation of *P. pachyrhizi* urediniospores on a hydrophobic surface in the presence of phytoalexin medicarpin or its intermediate compound formononetin-7-O-glucoside (ononin). Interestingly, the germination of urediniospores was slightly inhibited in the presence of either medicarpin or ononin when compared to mock control (Fig. 8). In addition, the formation of pre-penetration structures including germ-tubes and appressoria were significantly suppressed by treatment with either medicarpin or ononin (Fig. 8). Inhibition was more prominent at higher concentration. These results clearly indicate that phytoalexin medicarpin plays an important role in nonhost resistance of *M. truncatula* against *P. pachyrhizi.*

## Discussion

In this study, we characterized a model pathosystem between *M. truncatula* and ASR to investigate the molecular mechanism of nonhost resistance (Fig. 1). We identified the key genes or pathways for nonhost disease resistance of *M. truncatula* against ASR and demonstrated that a large number of genes involved in phenylpropanoid and (iso) flavonoid biosynthetic pathways that produce phytoalexin medicarpin were upregulated after inoculation with *P. pachyrhizi* (Figs 2 and 3; Supplementary Fig. S3). Furthermore, a significant accumulation of the metabolite medicarpin was detected 72 hours after inoculation with *P. pachyrhizi* (Fig. 7). Consistent with the results from transcriptome and metabolome analyses during nonhost resistance, phytoalexin medicarpin significantly inhibited the pre-infection structure formation of *P. pachyrhizi* (Fig. 8). These results clearly suggest a correlation between phenylpropanoid and (iso) flavonoid pathways leading to medicarpin production and nonhost resistance in *M. truncatula* against fungal pathogens such as *P. pachyrhizi*. Interestingly, similar results were observed in soybean transcriptome analysis for *Rpp2*-mediated disease resistance against ASR wherein the expression of phenylpropanoid pathway genes, such as *PAL*, was induced during infection^39^. It was reported that *PAL1*-silenced soybean plants compromised *Rpp2*-mediated disease resistance against ASR^40^. In addition, the expression of genes involved in the phenylpropanoid pathway was also reported to be induced in *Rpp3*-mediated disease resistance in soybean^41^. It is known that the phenylpropanoid pathway has an important role in providing precursors not only for the production of antimicrobial metabolites, such as phytoalexins, but also for lignin biosynthesis^42^. Together with our present study, these results suggest that both *R*-gene-mediated resistance and nonhost resistance utilize the phenylpropanoid pathway to produce antimicrobial metabolites.

Metabolome analysis identified a significant accumulation of flavones and isoflavones, precursors for the production of antimicrobial metabolites, after inoculation with *P. pachyrhizi* (Fig. 7). The accumulation of these metabolites correlated with the expression profiles of genes involved in phenylpropanoid and flavonoid pathways (Figs 2 and 3). We also detected a significant accumulation of triterpenoid saponins 72 hpi with *P. pachyrhizi* (Fig. 7). However, unlike phenylpropanoid and flavonoid pathways, no significant induction of genes involved in triterpenoid saponins biosynthesis pathways was observed in transcriptome analysis (Fig. 2). This is probably due to the early time points (12 and 24 hpi) used for gene expression analyses. It is known that triterpenoid saponins function as important antimicrobial compounds and have beneficial effects for human health^43^. However, the biosynthetic pathway of saponins is still poorly understood^44^. Recent studies have shown that despite the accumulation of medicagenic acid, a major component in *M. truncatula* saponins in leaves, the transcripts of *CYP716A12* that encode the key enzyme for saponin biosynthesis were highly expressed in roots^45^. Furthermore, *M. truncatula UGT73F3*, which encodes glycosyltransferases in triterpene saponin biosynthesis, was also mainly expressed in roots^44^. It has been demonstrated that mutants corresponding to these genes showed severe growth inhibition indicating that triterpene saponins may have an important role in plant developmental processes. In addition, Papadopoulou *et al.*^46^ have shown that saponin-deficient (*sad*) mutants of oat species *Avena strigose* were compromised in their resistance to a variety of fungal pathogens. Triterpene 12,13β-epoxy-16β-hydroxy-β-amyrin is also shown to be an important antifungal compound^47^ (Geisler *et al.*, 2013). Medicagenic acid saponins are shown to have antifungal activity to *Sclerotium rolfsii*, *Rhizoctonia solani*, *Trichoderma viride*, *Aspergillus niger* and *Fusarium oxysporum*^48^. Antifungal activity of saponin glycosides, including hederagenin 3-O-β-D-glucopyranoside and medicagenic acid 3-O-β-D-glucopyranoside, is demonstrated^49^. Thus, it is likely that triterpene saponins have dual functions involved in defense responses and in plant developmental processes. Further precise investigation of the expression profiles is needed to understand the function of triterpene saponins in nonhost resistance of *M. truncatula*.

In recent years, the importance of chloroplasts in plant immunity has been reported^50^. There are numerous reports showing that pathogens target host chloroplast to cause disease^51^,^52^,^53^. Furthermore, there is an important link between light and disease resistance. The intermediate metabolites derived from chlorophyll degradation are known to generate ROS which function as signaling molecules to cause cell death, that play a role in disease resistance^32^,^54^,^55^. Consistent with these reports, the present study demonstrated that the expression of genes involved in chlorophyll catabolism, including *NYC1*, *CHL*, *PAO* and *SGR*, was significantly induced by inoculation with *P. pachyrhizi* (Fig. 4; Supplementary Table S9). On the other hand, the expression of genes related to photosynthetic pathways was suppressed during *P. pachyrhizi* infection (Supplementary Figs S2 and S3). These results suggest that optimization of chlorophyll catabolism could be essential for protecting plants against the toxicity of ROS, especially when the photosynthetic activity is suppressed by stresses such as pathogen infection. Interestingly, similar results were observed in a previous study of Arabidopsis *SGR*. It was reported that the expression of *AtSGR* was induced after inoculation with an avirulent bacterial pathogen, *Pseudomonas syringae* pv. *tomato* DC3000 (*Pst* DC3000) carrying *AvrRpm1*^55^, suggesting that *SGR* may play a role in plant defense. Furthermore, transcriptome analysis for soybean demonstrated that the expression of genes involved in chlorophyll catabolism including *SGR* and *CHL* was induced in the compatible interactions with *P*. *pachyrhizi*^41^. However, the induction of *SGR* and *CHL* was ~2.5-fold in contrary to ~40-fold induction of *SGR* and *CHL* during nonhost resistance in *M*. *truncatula* (Fig. 4). Consistent with the fact that pathogen effectors modulate host defense machinery, we speculate that *P. pachyrhizi* utilizes effectors to interfere with the expression of chlorophyll catabolism pathway genes, especially *SGR*, to suppress plant defense.

A *M. truncatula Tnt1* insertion collection^56^,^57^ was previously utilized to identify a *sgr* mutant line^38^. We used the *M. truncatula sgr* mutant to characterize the function of SGR in plant defense response. A previous study of the *Arabidopsis no chlorosis1* (*noc1*/*sgr1*) mutant with hemibiotrophic bacterial pathogen *Pst* DC3000 and necrotrophic fungal pathogen *Alternaria brassicicola* has shown that SGR plays a role in chlorosis symptom development^34^. It was also reported that the expression of *AtSGR1* was induced during *Pst* DC3000 or *A. brassicicola* infection^34^. Interestingly, our study demonstrated that the necrotic cell death associated with high levels of ROS accumulation was observed in the *M. truncatula sgr* mutant inoculated with *P. pachyrhizi* suggesting a negative role of SGR in HR-like cell death (Fig. 6). In contrast, earlier studies reported a positive role for SGR in cell death phenomenon. Overexpression of *AtSGR* in Arabidopsis caused spontaneous, HR-like necrotic cell death, and *AtSGR* RNAi lines showed a delay in HR upon inoculation with an avirulent pathogen^55^. A negative role of *MtSGR* in HR-like cell death is intriguing. In addition, it has been reported that SGR is essential for recruiting the enzymes involved in chlorophyll catabolism during senescence by binding to light-harvesting complex II^37^. Therefore, it is likely that SGR may function as a key regulator, not only for leaf senescence and disease symptom development, but also for plant immunity through feedback regulation in certain plant species. *P. pachyrhizi* could utilize its effectors to interfere with a plant’s chlorophyll catabolism pathway for pathogenicity by inducing *SGR* to increase chlorosis in infected tissue. In addition, the induction of *SGR* in *M. truncatula* inhibits cell death related to HR, and this helps with the virulence of the pathogen. However, the suppression of HR alone by *SGR* is perhaps not sufficient for *P. pachyrhizi* to cause disease in *M. truncatula* (Fig. 1). The exact role of *SGR* in HR-like cell death needs further investigation using overexpression lines for *SGR*.

## Methods

### Plant growth conditions and pathogen inoculation assay

Seeds of *M. truncatula* ecotype R108 and *sgr* mutant (NF2089) were scarified for 8 min using concentrated sulfuric acid, washed thrice with distilled water and germinated on moist filter papers. Two days after germination in darkness at 24 °C, 12 seedlings were transferred to soil (one seedling per cell in 6 × 12 celled trays). Following three weeks’ incubation in the greenhouse, the plants were transferred to growth chambers located in a USDA-APHIS approved BSL2 + facility to conduct ASR inoculation assays.

Pathogen inoculation and cytological studies were conducted as described previously^58^. An isolate of the ASR pathogen *P. pachyrhizi*, from Illinois, was maintained on the susceptible soybean cultivar (*Glycine max* cv. Williams) in a growth chamber at 22 °C/19 °C with 12-hrs-light/12-hrs-dark cycle (100–150 μmol m^–2^ s^–1^). Fresh urediniospores were collected using a gelatin capsule spore collector designed by the CDL, St. Paul, MN, and suspended in distilled water with 0.001% Tween 20. The 4-week-old *M. truncatula* plants were spray-inoculated with 1 × 10^5^ spores/ml (0.001% Tween 20) using an artist air-brush (Paasche Airbrush Co. Chicago, IL, USA) set at 2 PSI with a portable air-pump (Gast Mfd Co. Benton Harbor, MI, USA) for uniform spore deposition. The inoculated plants were maintained in a dew chamber for 24 hrs with 100% humidity maintained at 19 °C; 0-hrs-light/24-hrs-dark cycle. The plants were then transferred to a growth chamber (22 °C/19 °C with 12 hrs-light/12 hrs-dark cycle) and incubated further to allow symptom development.

To quantify the formation of pre-penetration structures, approximately 100 spores of *P. pachyrhizi* in 10-μL aliquots were placed on the abaxial surface of the detached leaves of 4-week-old soybean (*Glycine max* cv. Williams) or *M. truncatula* wild-type R108 and incubated in dark overnight and then transferred to a growth chamber (22 °C/19 °C with 12-h-light/12-h-dark cycle). At 24 hours after inoculation, the leaves were stained with 10 μg/mL WGA-Alexa Fluor 488 (WGA-Alexa Fluor® 488; Invitrogen, Corp., Carlsbad, CA, USA) supplemented with 0.05% Tween 20 to visualize the fungal germ-tubes and appressoria. The subsequent developments were followed 72 hours after inoculation, and the germ-tubes forming differentiated appressoria were counted as appressoria. The differentiated germ-tubes without appressoria that grew on the surface were also counted from 20 random fields on three independent leaves. The number of dead autofluorescing epidermal cells resulting from direct penetration of *P. pachyrhizi* were counted 72 hours after inoculation from 20 random fields per each inoculated site and are used to calculate the percentage of penetration.

Alfalfa anthracnose pathogen, *C. trifolii* race 1 was maintained on potato dextrose agar media. Two-weeks-old cultures of conidia were harvested, washed in water, and re-suspended in sterile distilled water. Leaves from 4-week-old wild-type R108 and *sgr* mutant were harvested and spot- inoculated with 10 μL of suspension 1 × 10^6^ spores/mL in 0.005% Tween 20. The disease symptom development was recorded 4 days after inoculation.

### Light microscopy

Initial interactions of *P. pachyrhizi* with *M. truncatula* were recorded by direct observation of inoculated leaves using an Olympus stereo (SZX19, Olympus Corporation Co. Ltd., Tokyo, Japan) or compound microscopes (BX 41, Olympus Corporation Co. Ltd., Tokyo, Japan) equipped with fluorescence attachment. For fluorescence microscopy, fungal mycelia were stained with wheat germ agglutinin (WGA) coupled to green fluorescent dye Alexa Fluor 488 as described previously^59^. Inoculated leaves were stained with 10 μg/mL WGA-Alexa Fluor 488 by a brief vacuum infiltration in phosphate-buffered saline (PBS) followed by a 20 min incubation at room temperature. For microscopic observations, after washing with PBS, whole or sections of the leaf were placed on a glass slide and mounted using a cover glass with Dow Corning® (Midland, MI, USA) high vacuum grease for microscopy. Fluorescence microscopy to document the infection process was done using Olympus epifluorescence microscope.

### Detection of hydrogen peroxide

The generation of hydrogen peroxide was detected using 3,3′-diaminobenzidine (DAB) staining as described previously^53^. Inoculated leaves were placed in 1 mg/ml DAB-HCl (pH 3.8). After incubation for 6 hours at room temperature, chlorophyll was removed with 95% ethanol.

### Transcriptome analysis

The inoculated leaves were gently ground by mortar and pestle with liquid nitrogen. The powdered samples were split into two aliquots for transcriptome and metabolome analyses. Total RNA was purified from inoculated leaves using TRIzol reagent (Invitrogen). For microarray analysis, the total RNA was purified using the RNAeasy MinElute Cleanup Kit (Qiagen, Valencia, CA, USA), and 500 ng of total RNA was used for hybridization with the Affymetrix *Medicago* GeneChip. Three biological replicates were used for each inoculation. Each biological replicate consisted of a pool of eight plants. Experimental and statistical procedures were as described previously^59^. Briefly, data normalization between chips was conducted using RMA (Robust Multi-chip Average^60^). Presence/absence call for each probe set was obtained using dCHIP^61^. Gene selections based on associative t-test^62^ (Dozmorv and Centola, 2003) were made using Matlab (MathWorks, Natick, MA, USA). A selection threshold of 3 for transcript ratios (where applicable) and a Bonferroni correction *P* value threshold of 1.1406e-006 were used. Differentially expressed genes between mock and ASR inoculation were selected, and functional annotation of genes was performed as described previously^58^. Microarray data are available in the ArrayExpress database ( www.ebi.ac.uk/arrayexpress) under accession number E-MTAB-2911.

### Real-time quantitative RT-PCR analyses

Real-time quantitative RT-PCR (RT-qPCR) was performed as described^59^ using the gene-specific primers designed based on the target sequence (Supplemental Table S10). Total RNA was treated with Turbo DNase (Ambion) to eliminate genomic DNA, and 5 μg of DNase-treated RNA was reverse-transcribed using Superscript III reverse transcriptase (Invitrogen) with oligo d(T)20 primers. The cDNA (1:10) was then used for RT-qPCR. The RT-qPCR was performed using gene-specific primer sets (Supplementary Table S10) and Power SYBR Green PCR master mix (Applied Biosystems, Foster City, CA, USA) in an optical 384-well plate with an ABI Prism 7900 HT sequence detection system (Applied Biosystems). Melt-curve analysis was performed to monitor primer-dimer formation and to check amplification of gene-specific products. The average threshold cycle (CT) values calculated from triplicate biological samples were used to determine the fold expression relative to the controls. Primers specific for *Ubiquitin* were used to normalize small differences in template amounts.

### Metabolome analysis

The total metabolites were extracted from 10 mg of dried samples in 80% methanol containing 18 ng/μl of umbelliferone (Sigma-Aldrich, St. Louis, MO, USA) as an internal standard. Samples were agitated for 2 hours at room temperature followed by centrifugation at 2900 × g for 30 minutes. The supernatant was transferred to a 2 mL autosampler vial. Five microliters of the solution were injected on to a Waters Acquity UPLC system fitted with a hybrid quadrupole time-of-flight (QTOF) Premier mass spectrometer (MS; Waters). A reverse-phase, 1.7-μm UPLC BEH C18, 2.1 × 150 mm column (Waters) was used for separations. The mobile phase consisted of eluent A (0.05% [v/v] formic acid/water) and eluent B (acetonitrile), and separations were achieved using a linear gradient of 95 to 30% A over 30 min, 30 to 5% A over 3 min, and 5 to 95% A over 3 min. The flow rate was 0.56 mL/min, and the column temperature was maintained at 60 °C. Masses of the eluted compounds were detected in the negative ESI mode from 50 to 2,000 mass-to-charge ratio. The QTOF Premier mass spectrometer was operated under the following instrument parameters: desolvation temperature of 375 °C; desolvation nitrogen gas flow of 850 liters/h; capillary voltage of 2.9 kV; cone voltage of 48 eV; and collision energy of 10 eV. The MS system was calibrated using sodium formate, and raffinose was used as the lockmass. Metabolites were identified based on accurate masses and retention times relative to authentic standards. Data Bridge (Mass Lynx version 4.1) was used to convert the raw data files to NetCDF. Relative abundances were calculated using MET-IDEA, and the peak areas were normalized by dividing each peak area by the value of the internal standard peak area.

### *In vitro* urediniospore germination and differentiation assay

Two hundred microliters of *P. pachyrhizi* urediniospores (1 × 10^6^ spores/mL) were mixed with DMSO (mock control) or 5 mM medicarpin or 5 mM ononin at a final concentration of 50 μM or 200 μM of medicarpin or ononin. These urediniospores were incubated in 6-well cell plastic culture plates (BD Falcon) for 18 h in dark. After incubation, the number of *P. pachyrhizi* spores that germinated and germ tubes with and without differentiated appressoria were counted from eight random fields. Average of eight observations was used to calculate the percentage of spore germination and germ tubes with and without an appressorium. These experiments were repeated more than two times.

## Additional Information

**How to cite this article**: Ishiga, Y. *et al.* Transcriptomic and metabolomic analyses identify a role for chlorophyll catabolism and phytoalexin during *Medicago* nonhost resistance against Asian soybean rust. *Sci. Rep.* **5**, 13061; doi: 10.1038/srep13061 (2015).

## Supplementary Material

Supplementary Information
