# Supplementary material for: Transcriptomic and metabolomic analyses identify a role for chlorophyll catabolism and phytoalexin during Medicago nonhost resistance against Asian soybean rust
Source: Sci Rep. 2015 Aug 12;5:13061. doi: 10.1038/srep13061 (PMC4533520; doi:10.1038/srep13061)
Supplement: Supplementary Information [file srep13061-s1.pdf]

Manuscript title:

Transcriptomic and metabolomic analyses identify a role for chlorophyll catabolism and phytoalexin during *Medicago* nonhost resistance against Asian soybean rust

Authors' full names:

Yasuhiro Ishiga[1], Srinivasa Rao Uppalapati[1,2], Upinder S. Gill, David Huhman, Yuhong Tang and Kirankumar S. Mysore[*]

Institution addresses:

Plant Biology Division, The Samuel Roberts Noble Foundation, Ardmore, OK 73401, USA (Y.I., S.U., U.G., D.H., Y.T., K.M.)

Faculty of Life and Environmental Sciences, University of Tsukuba, 1-1-1 Tennodai, Tsukuba, Ibaraki 305-8572, Japan (Y. I.)

[1]These authors contributed equally.

Running title:

Omics of *M. truncatula*-soybean rust interaction

For correspondence:
Kirankumar S. Mysore
Address:
Plant Biology Division, The Samuel Roberts Noble Foundation, Ardmore, OK 73401, USA
Email: ksmysore@noble.org
Tel. (+1) 580-224-6740; Fax (+1) 580-224-6692

Fig. S1

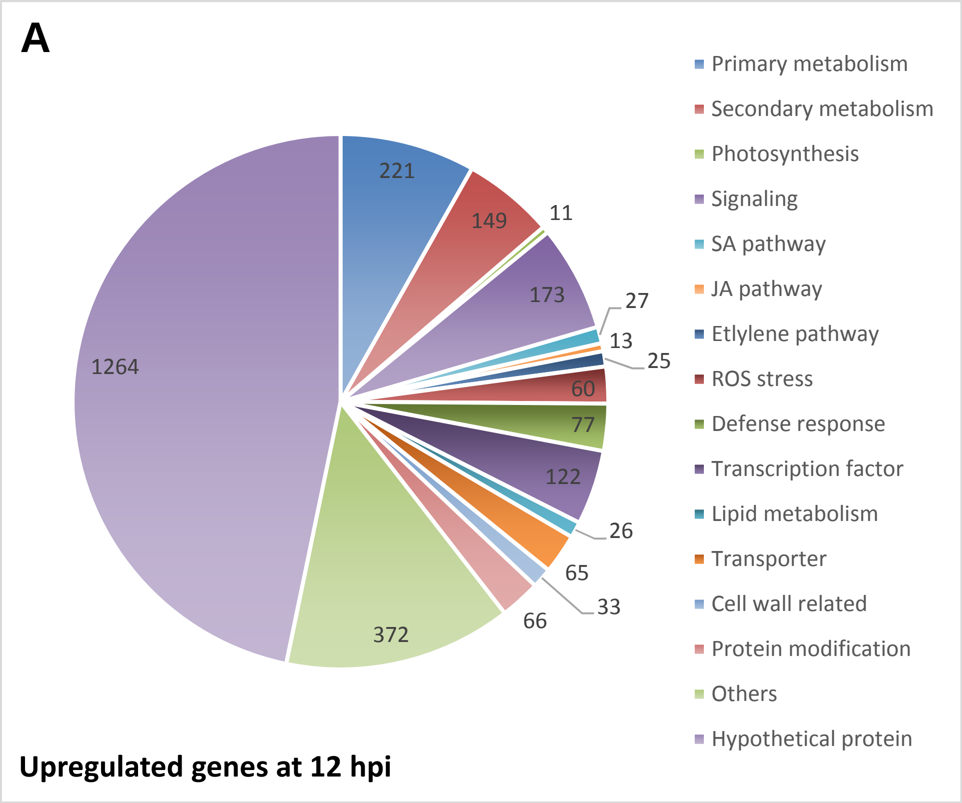

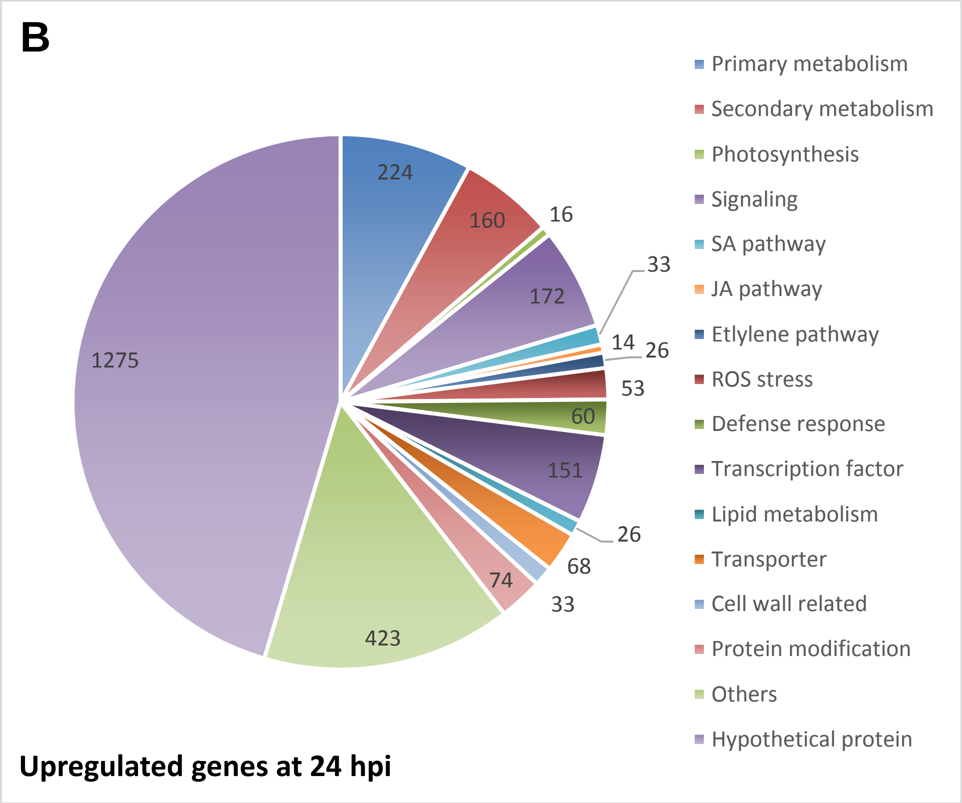

Supplementary Figure S1. Piecharts showing functional classification of upregulated genes during *M. truncatula-P. pachyrhizi* interactions at 12 (A) and 24 (B) hours postinoculation (hpi).

Fig. S2

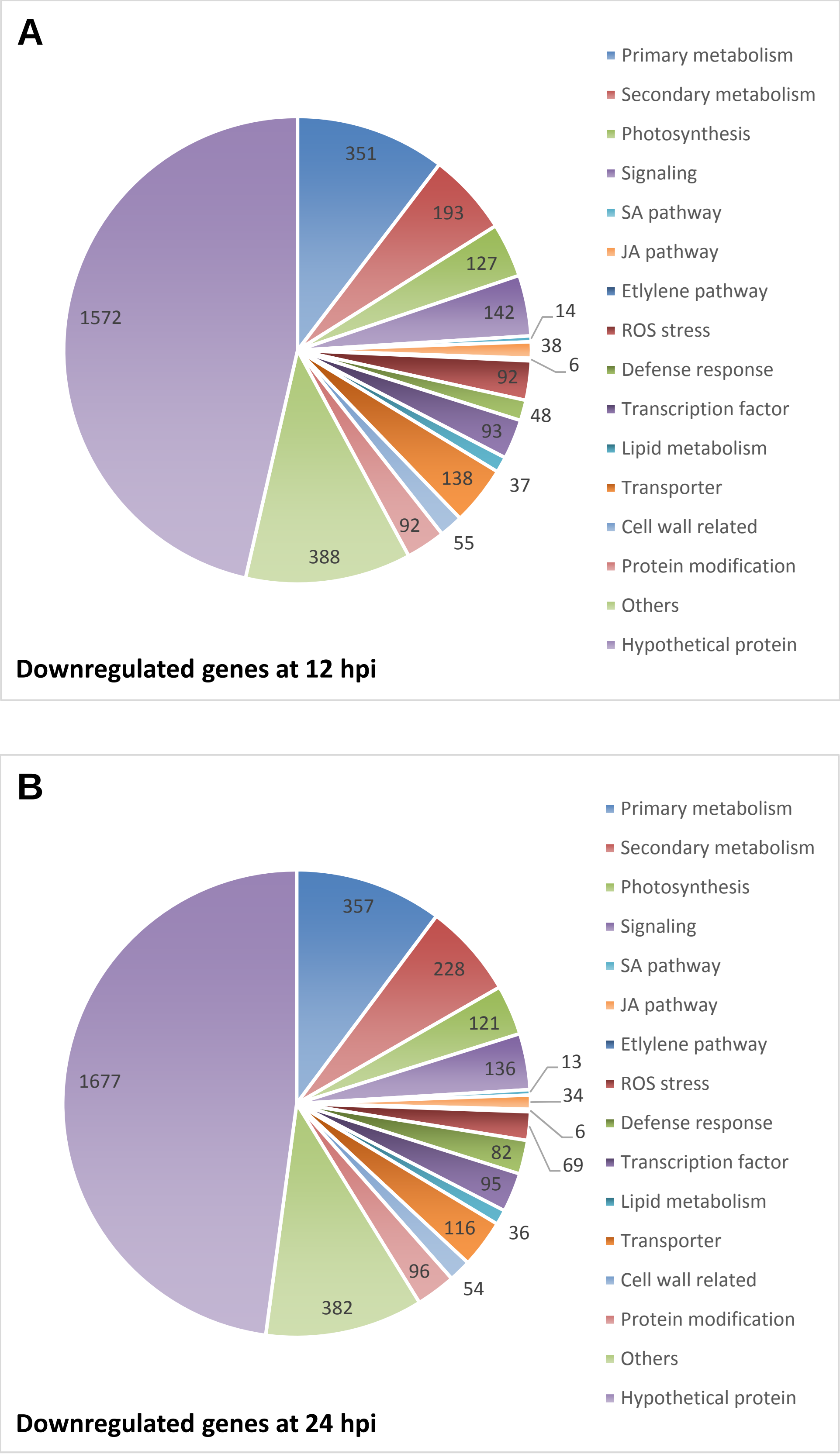

Supplementary Figure S2. Piecharts showing functional classification of downregulated genes during *M. truncatula-P. pachyrhizi* interactions at 12 (A) and 24 (B) hours postinoculation (hpi).

Fig. S3

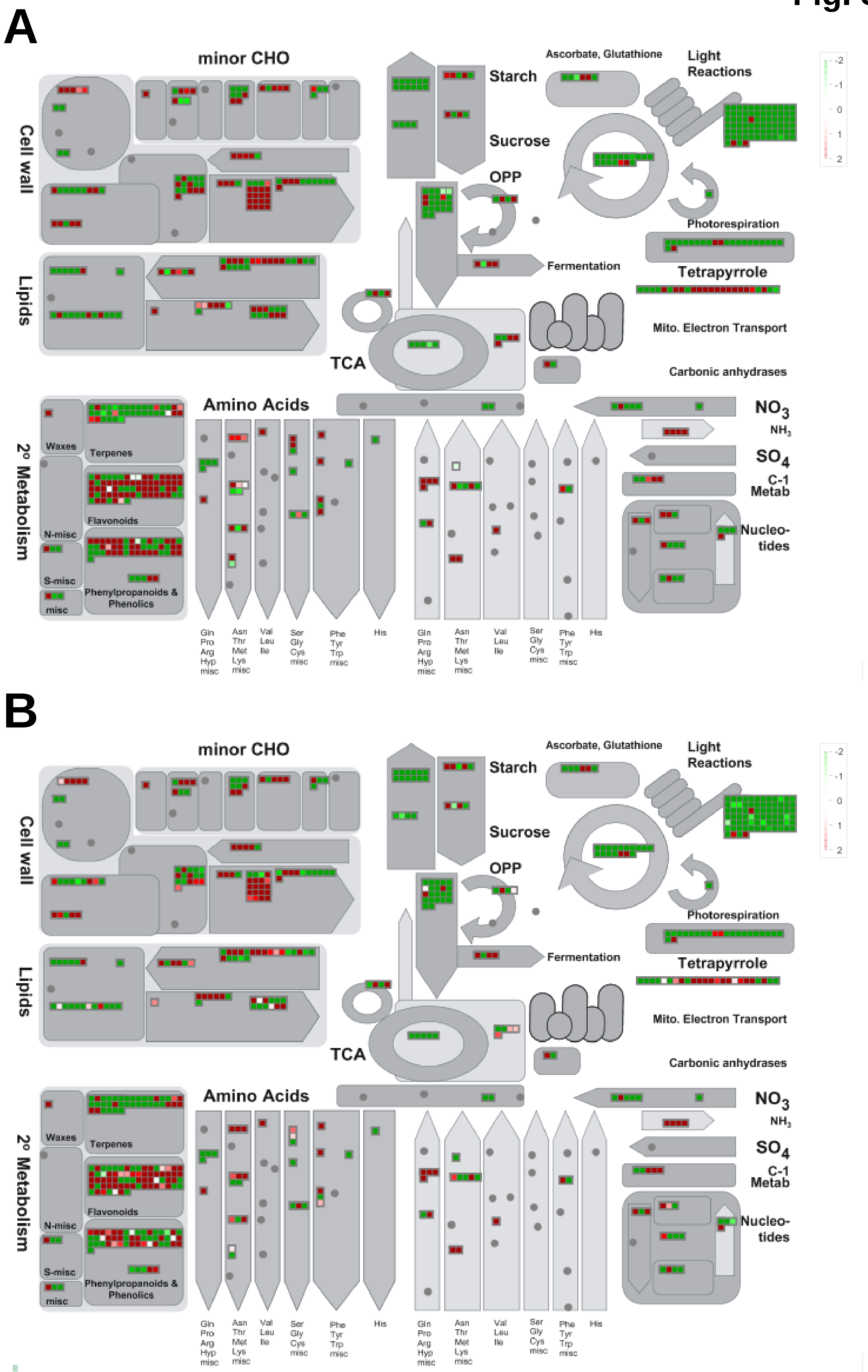

Supplementary Figure S3. MAPMAN illustration of *Medicago truncatula* Affymetrix data showing the genes during *M. truncatula-P. pachyrhizi* interactions at 12 (A) and 24 (B) hours postinoculation (hpi). Red indicates up-regulation and green indicates down-regulation.

Fig. S4

A

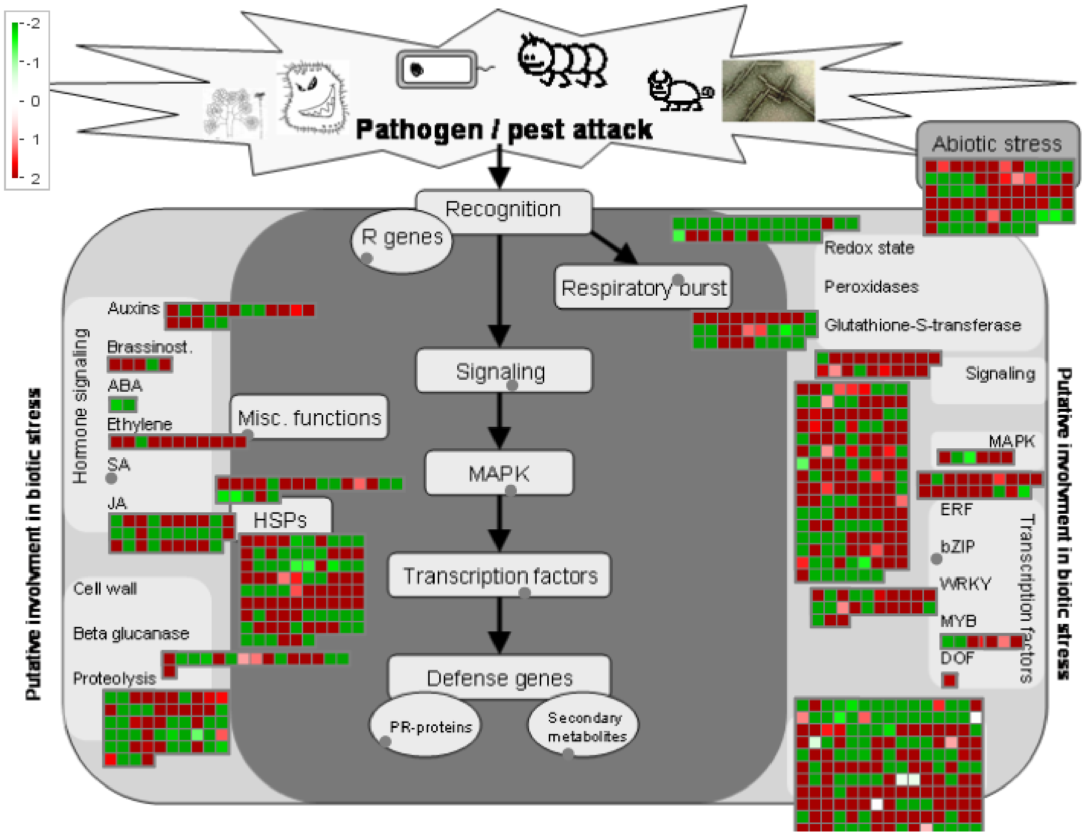

B

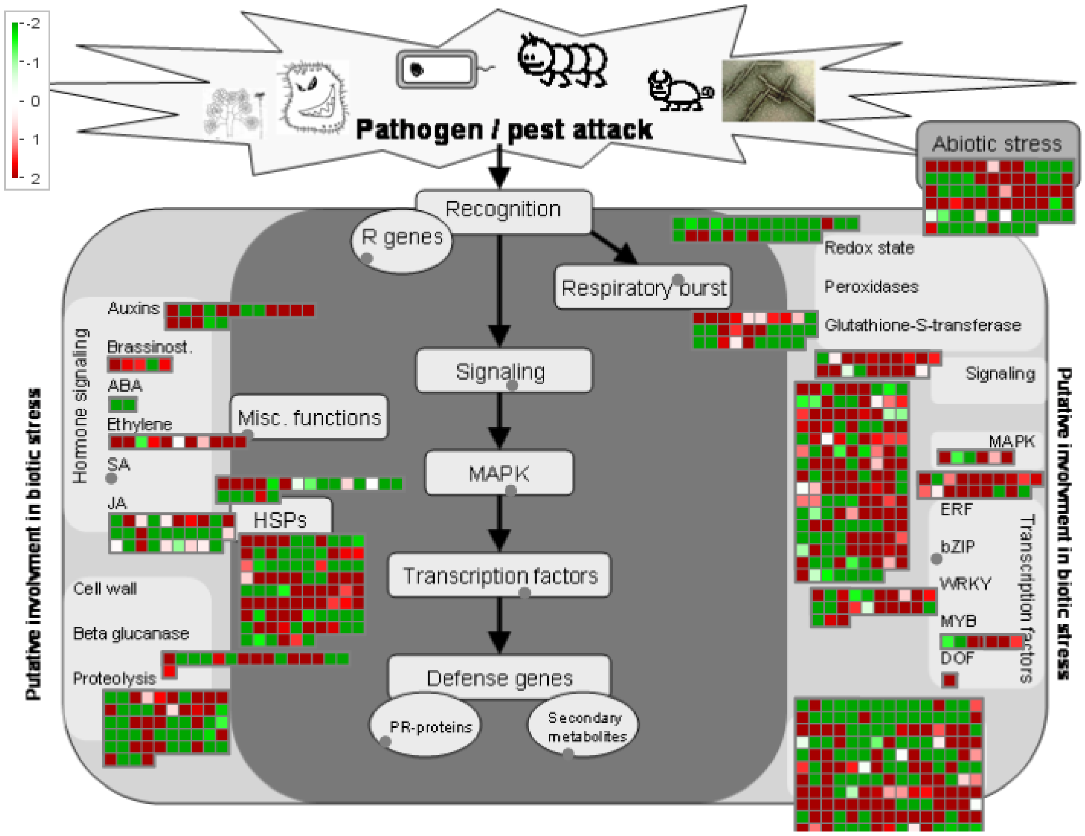

Supplementary Figure S4. MAPMAN illustration of *Medicago truncatula* Affymetrix data showing the genes involved in stress responses during *M. truncatula-P. pachyrhizi* interactions at 12 (A) and 24 (B) hours post inoculation (hpi). Red indicates up-regulation and green indicates down-regulation.

Fig. S5

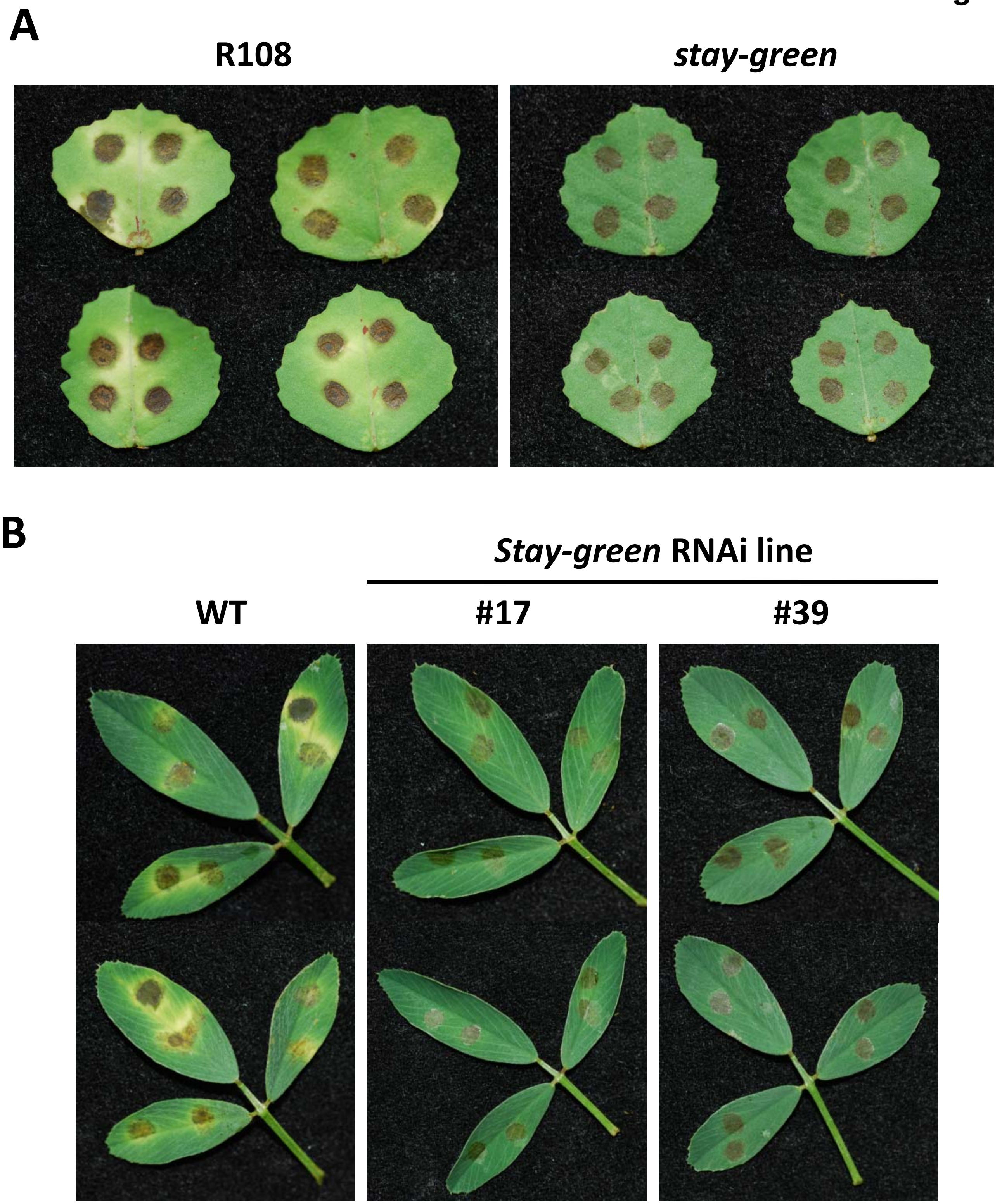

Supplementary Figure S5. Disease symptom development of *M. truncatula* and alfalfa leaves inoculated with *Colletotrichum trifolii*. (A) Disease symptom development of *M. truncatula* wild-type R108 and *sgr* mutant at 4 days post-inoculation (dpi). Four-week-old *M. truncatula* wild-type R108 and *sgr* mutant were spot-inoculated with 10 µl of 1×10^6 spores/mL in 0.005% Tween 20. Photos were taken 5 days after inoculation. (B) Disease symptom development of alfalfa wild-type and *SGR*-RNAi lines at 4 dpi. Alfalfa leaves were inoculated in the same fashion as *M. truncatula* lines described above. All the experiments were repeated at least thrice with similar results.

Supplementary Table S1. Upregulated genes at 12 hours after inoculation with soybean rust

Probesets	Target Description	Fold induction
Mtr.37395.1.S1_at	Deoxycytidine deaminase	869.00
Mtr.43146.1.S1_at	Branched-chain-amino-acid aminotransferase 2	684.50
Mtr.15537.1.S1_at	Hypothetical protein	498.39
Mtr.18650.1.S1_at	Glycoside hydrolase, family 17	483.72
Mtr.20464.1.S1_x_at	Naringenin-chalcone synthase	453.88
Mtr.19796.1.S1_at	Hypothetical protein	448.29
Mtr.43467.1.S1_at	Hypothetical protein	425.57
Mtr.37375.1.S1_at	Hypothetical protein	417.63
Mtr.17526.1.S1_at	Hypothetical protein	417.11
Mtr.8498.1.S1_s_at	Asparagine synthetase	388.70
Mtr.22656.1.S1_s_at	Deoxycytidylate deaminase	385.47
Mtr.1538.1.S1_at	Hypothetical protein	335.94
Mtr.11725.1.S1_at	Isoflavone synthase	332.58
Mtr.45667.1.S1_x_at	Naringenin-chalcone synthase	328.39
Msa.1736.1.S1_at	Hypothetical protein	304.80
Mtr.5486.1.S1_s_at	Hypothetical protein	301.78
Mtr.11267.1.S1_at	Protein kinase-like protein	273.51
Mtr.35334.1.S1_s_at	Hypothetical protein	262.59
Mtr.18127.1.S1_at	Hypothetical protein	253.13
Mtr.33212.1.S1_s_at	Beta-glucosidase	228.45
Mtr.8498.1.S1_at	Asparagine synthetase	222.37
Mtr.44900.1.S1_at	Hypothetical protein	222.02
Mtr.8417.1.S1_at	Mitochondrial uncoupling protein (UCP protein)	215.40
Msa.2530.1.S1_s_at	Hypothetical protein	214.94
Msa.2901.1.S1_s_at	Hypothetical protein	211.08
Mtr.44354.1.S1_at	Cytochrome P450 monooxygenase	210.43
Msa.1879.1.S1_s_at	Hypothetical protein	199.51
Mtr.40147.1.S1_s_at	Class 10 PR protein	199.02
Mtr.44559.1.S1_at	Hypothetical protein	198.53
Mtr.37984.1.S1_at	Adenosine nucleotide translocator	194.52
Mtr.43830.1.S1_at	Hypothetical protein	193.67
Mtr.9372.1.S1_at	DnaJ protein	190.99
Mtr.12193.1.S1_at	RCa12	189.00
Mtr.40564.1.S1_at	Beta-glucosidase	188.98
Mtr.45497.1.S1_s_at	Geraniol synthase	187.17
Mtr.40120.1.S1_at	Cationic peroxidase 1 precursor (PNPC1)	181.77
Mtr.15678.1.S1_s_at	Aldo/keto reductase	180.19
Mtr.17404.1.S1_s_at	Aldo/keto reductase	179.94
Msa.1060.1.S1_at	Hypothetical protein	175.79
Mtr.23578.1.S1_s_at	Polygalacturonase-inhibiting protein precursor	175.72
Mtr.44496.1.S1_at	AP2 domain-containing protein AP29-like	168.51
Mtr.11851.1.S1_at	Hypothetical protein	165.21
Mtr.51826.1.S1_at	Hypothetical protein	164.22
Mtr.35755.1.S1_at	Hypothetical protein	161.83
Mtr.43284.1.S1_at	Alcohol dehydroge	145.45
Msa.2530.1.S1_at	Hypothetical protein	143.16
Mtr.29026.1.S1_at	Hypothetical protein	142.69
Mtr.10502.1.S1_s_at	Glutathione S-transferase	142.41
Mtr.43363.1.S1_at	Syringolide-induced protein 13-1-1	142.17
Mtr.34114.1.S1_s_at	Pathogenesis related protein	139.27
Mtr.410.1.S1_s_at	Isoflavone reductase	134.94
Mtr.16278.1.S1_at	Proteinase inhibitor I12	133.70

Mtr.43189.1.S1_at	Disease resistance response protein	131.36
Mtr.17621.1.S1_x_at	Naringenin-chalcone synthase	128.32
Mtr.17612.1.S1_x_at	Naringenin-chalcone synthase	127.58
Mtr.42416.1.S1_at	Hypothetical protein	127.45
Mtr.21662.1.S1_at	Plant integral membrane prote	126.40
Mtr.37358.1.S1_s_at	Cytochrome P450 71B13	126.22
Mtr.42989.1.S1_at	Thaumatin-like protein PR-5b	126.14
Mtr.42268.1.S1_at	Inositol oxygenase (Myo-inositol oxygenase)	125.59
Mtr.43008.1.S1_at	Hypothetical protein	124.73
Mtr.48534.1.S1_at	Cytochrome b561 / ferric reductase	122.44
Mtr.34158.1.S1_s_at	Hypothetical protein	120.76
Mtr.15831.1.S1_s_at	Hypothetical protein	120.18
Mtr.11055.1.S1_at	SRG1 protein	119.87
Mtr.10434.1.S1_at	SRG1 protein	117.95
Mtr.22592.1.S1_at	Cytosolic fructose-1,6-bisphosphatase	115.88
Mtr.6667.1.S1_at	Cytochrome P450 monooxygenase	115.48
Mtr.46391.1.S1_at	Hypothetical protein	111.35
Mtr.49411.1.S1_at	Hypothetical protein	111.22
Mtr.16615.1.S1_at	Prephenate dehydratase	108.21
Mtr.37356.1.S1_at	Cytochrome P450 71B25	107.68
Mtr.43254.1.S1_at	Hypothetical protein	105.29
Mtr.37033.1.S1_s_at	Metalloproteinase	103.37
Mtr.79.1.S1_at	Hypothetical protein	103.23
Mtr.43279.1.S1_at	Proline dehydrogenase	102.24
Mtr.11773.1.S1_s_at	Hypothetical protein	98.32
Mtr.38764.1.S1_at	Cytochrome P450	96.76
Mtr.40122.1.S1_s_at	Chalcone synthase 4	95.35
Mtr.43203.1.S1_at	Hypothetical protein	94.75
Mtr.35677.1.S1_at	Zinc finger protein family-like	94.56
Mtr.42129.1.S1_at	AP2 domain transcription factor	94.43
Mtr.49341.1.S1_at	Peptidase	93.09
Mtr.17616.1.S1_x_at	Naringenin-chalcone synthase	92.61
Mtr.43594.1.S1_at	Hypothetical protein	92.49
Mtr.45394.1.S1_at	Synaptobrevin-like protein	91.96
Mtr.10368.1.S1_at	Chalcone synthase 8	91.23
Mtr.33545.1.S1_s_at	Non-cyanogenic beta-glucosidase	91.01
Mtr.42255.1.S1_at	Hypothetical protein	90.54
Mtr.43259.1.S1_at	Hypothetical protein	88.23
Mtr.43942.1.S1_s_at	Hypothetical protein	87.16
Mtr.10760.1.S1_at	N-hydroxycinnamoyl/benzoyltransferase-like protein	87.12
Mtr.35802.1.S1_at	Hypothetical protein	85.55
Mtr.41059.1.S1_at	Hypothetical protein	84.57
Mtr.43159.1.S1_at	Bacterial-induced peroxidase	82.48
Mtr.12748.1.S1_at	Hypothetical protein	82.45
Mtr.28242.1.S1_at	Beta-glucan-elicitor receptor	81.70
Mtr.331.1.S1_at	Chitinase	79.77
Mtr.5385.1.S1_at	Ethylene reponse factor-like AP2 domain transcription factor	79.56
Mtr.10391.1.S1_at	Class 10 PR protein	78.99
Mtr.47292.1.S1_at	Hypothetical protein	78.70
Mtr.13321.1.S1_at	Pyruvate, phosphate dikinase	78.51
Mtr.27937.1.S1_at	Nodulin-like protein	78.41
Mtr.23418.1.S1_s_at	AP2 domain transcription factor	78.36
Mtr.34416.1.S1_at	Pyruvate,orthophosphate dikinase	77.60
Mtr.4663.1.S1_at	Light-inducible protein ATLS1	77.52

Mtr.13532.1.S1_at	Cytochrome P450	77.16
Mtr.23863.1.S1_at	Glucan 1,3-beta-glucosidase	76.96
Mtr.8652.1.S1_at	6a-hydroxymaackiain methyltransferase	76.69
Mtr.38035.1.S1_at	S-locus-like receptor protein kinase	76.06
Mtr.12424.1.S1_at	Disease resistance response protein	75.35
Mtr.34968.1.S1_at	Nucellin-like protein	75.13
Mtr.43196.1.S1_at	Hypothetical protein	74.50
Mtr.30508.1.S1_at	Isoflavone reductase	74.11
Mtr.12487.1.S1_at	Cyanogenic Beta-Glucosidase	73.71
Mtr.43852.1.S1_at	Acidic glucanase	73.13
Mtr.23580.1.S1_at	Polygalacturonase inhibitor protein	72.34
Mtr.21098.1.S1_at	Oxysterol-binding protein	71.25
Mtr.30776.1.S1_at	Patatin-like protein 1	71.22
Mtr.2105.1.S1_at	Hypothetical protein	70.71
Mtr.41238.1.S1_at	Hypothetical protein	70.05
Mtr.45666.1.S1_at	Glycoside hydrolase, family 32	69.96
Mtr.22854.1.S1_at	bHLH protein family	69.69
Mtr.10435.1.S1_s_at	SRG1 protein	69.13
Mtr.15261.1.S1_s_at	Curculin-like (mannose-binding) lectin	68.67
Mtr.25008.1.S1_x_at	Vestitone reductase	67.12
Mtr.6976.1.S1_at	Hypothetical protein	67.03
Mtr.10272.1.S1_at	Calmodulin	67.02
Mtr.10466.1.S1_at	Hypothetical protein	67.00
Mtr.37376.1.S1_s_at	Hypothetical protein	65.60
Mtr.38079.1.S1_at	Calcium/calmodulin protein kinase 1	65.36
Mtr.42451.1.S1_at	Bimodular protein	64.68
Mtr.10122.1.S1_at	Nine-cis-epoxycarotenoid dioxygenase1	62.89
Mtr.10573.1.S1_at	Hypothetical protein	62.88
Msa.1231.1.S1_at	Hypothetical protein	62.47
Mtr.25021.1.S1_at	Vestitone reductase	62.45
Mtr.45350.1.S1_at	Hypothetical protein	61.93
Mtr.41478.1.S1_at	Class 10 PR protein	60.99
Mtr.8507.1.S1_at	Pathogenesis related protein	60.07
Mtr.34634.1.S1_at	Epoxide hydrolase-like protein	59.63
Mtr.51252.1.S1_s_at	Hypothetical protein	59.56
Mtr.6639.1.S1_s_at	Hypothetical protein	58.91
Mtr.8558.1.S1_at	EDGP precursor	58.57
Mtr.13782.1.S1_s_at	Hypothetical protein	57.88
Mtr.17910.1.S1_at	Protein kinase	57.86
Mtr.37378.1.S1_at	Hypothetical protein	57.30
Mtr.48829.1.S1_at	Peptidase aspartic	56.68
Mtr.43772.1.S1_at	Hypothetical protein	56.35
Mtr.40890.1.S1_at	WRKY transcription factor 40	55.92
Mtr.38234.1.S1_at	Epoxide hydrolase-like protein	55.74
Mtr.40553.1.S1_at	Hypothetical protein	54.83
Mtr.35994.1.S1_at	Hypothetical protein	54.74
Mtr.40563.1.S1_at	Beta-Glucosidase	54.71
Mtr.45003.1.S1_at	Non-cyanogenic beta-glucosidase	54.68
Mtr.38513.1.S1_at	Syringolide-induced protein 14-1-1	54.53
Mtr.6645.1.S1_at	Lysine and histidine specific transporter	54.39
Mtr.41053.1.S1_at	AT-hook DNA-binding protein	54.03
Mtr.5060.1.S1_at	Cyanogenic Beta-Glucosidase	54.00
Mtr.4441.1.S1_at	Hypothetical protein	52.91
Mtr.12502.1.S1_at	Laccase-like protein	52.51

Mtr.40293.1.S1_at	Glutathione S-transferase	52.51
Mtr.16207.1.S1_at	Hypothetical protein	52.42
Mtr.44487.1.S1_at	Hypothetical protein	52.41
Mtr.42966.1.S1_at	Disease resistance response protein Pi49 (PR10)	52.33
Mtr.40504.1.S1_at	O-methyltransferase	52.21
Mtr.34185.1.S1_at	Hypothetical protein	50.05
Mtr.11104.1.S1_s_at	Hypothetical protein	48.95
Mtr.8442.1.S1_at	Hydroxyproline-rich glycoprotein (HRGP)	47.72
Mtr.5750.1.S1_at	Nicotiana EREBP-3-like protein	47.69
Mtr.35325.1.S1_at	Hypothetical protein	47.63
Mtr.45373.1.S1_at	Hypothetical protein	47.56
Mtr.34199.1.S1_at	2-oxoglutarate-dependent oxygenase	47.46
Mtr.10692.1.S1_at	Cytochrome P450 76C4	47.27
Mtr.32248.1.S1_at	3-hydroxyisobutyrate dehydrogenase-like protein	47.13
Mtr.10898.1.S1_at	Hypothetical protein	46.10
Mtr.47295.1.S1_x_at	Polygalacturonase inhibitor protein	46.05
Mtr.37316.1.S1_at	NAD(P)H dependent 6'-deoxychalcone synthase	46.02
Mtr.18422.1.S1_at	Hypothetical protein	45.91
Mtr.47992.1.S1_at	Hypothetical protein	45.69
Mtr.33217.1.S1_at	Syringolide-induced protein	45.64
Mtr.14428.1.S1_at	Naringenin-chalcone synthase	45.42
Mtr.37275.1.S1_at	Pathogen-inducible alpha-dioxygenase	45.41
Mtr.14224.1.S1_at	Proteinase inhibitor I13	45.25
Mtr.10896.1.S1_s_at	WIZZ	45.14
Mtr.27695.1.S1_at	Auxin response factor 30	44.90
Mtr.1402.1.S1_at	Hypothetical protein	44.76
Mtr.2340.1.S1_s_at	Hypothetical protein	44.16
Mtr.40601.1.S1_s_at	Collagen-like protein	44.02
Mtr.40598.1.S1_at	Hypothetical protein	43.84
Mtr.39844.1.S1_at	Hypothetical protein	43.76
Mtr.24901.1.S1_at	Phospholipase	43.73
Mtr.42078.1.S1_at	Anthocyanin 5-aromatic acyltransferase-like protein	42.73
Mtr.10396.1.S1_at	Syringolide-induced protein	42.60
Mtr.12616.1.S1_at	Cytochrome P450-like protein	42.41
Mtr.9091.1.S1_at	Hypothetical protein	42.39
Mtr.8601.1.S1_at	Hypothetical protein	42.32
Mtr.13317.1.S1_at	Hypothetical protein	42.09
Mtr.49090.1.S1_at	GCN5-related N-acetyltransferase	42.00
Mtr.7260.1.S1_at	Ethylene-induced esterase	41.86
Msa.2552.1.S1_at	Hypothetical protein	41.68
Msa.2942.1.S1_s_at	Hypothetical protein	41.24
Mtr.39762.1.S1_at	Chlorophyllase 1	41.00
Mtr.9846.1.S1_at	Hypothetical protein	40.92
Mtr.10427.1.S1_at	Hypothetical protein	40.55
Mtr.11565.1.S1_at	Patatin-like protein 1	40.32
Mtr.11047.1.S1_at	Mitogen-activated protein kinase 4	40.13
Mtr.38043.1.S1_at	Glucosyltransferase-10	39.78
Mtr.20344.1.S1_at	Zn-finger, RING protein	39.49
Mtr.28790.1.S1_at	Hypothetical protein	39.47
Mtr.25362.1.S1_s_at	LEDI-5c protein	39.38
Mtr.39322.1.S1_at	UDP-glucuronosyltransferase	38.82
Mtr.10319.1.S1_at	Pprg2 protein	38.74
Mtr.18521.1.S1_at	Thioredoxin-related protein	38.71
Mtr.33275.1.S1_s_at	Cell wall integrity and stress response component 4	38.70

Mtr.51082.1.S1_at	Trypsin-alpha amylase inhibitor	38.63
Mtr.14428.1.S1_x_at	Naringenin-chalcone synthase	38.63
Mtr.48768.1.S1_at	Gibberellin regulated protein	38.44
Mtr.6666.1.S1_at	Hypothetical protein	38.41
Mtr.42263.1.S1_s_at	Hypothetical protein	38.29
Mtr.51557.1.S1_at	Glycoside hydrolase, family 19	38.28
Mtr.13239.1.S1_at	Hypothetical protein	38.24
Mtr.5695.1.S1_at	Hypothetical protein	38.00
Mtr.10317.1.S1_at	Pprg2 protein	38.00
Mtr.5785.1.S1_s_at	PDR7 ABC transporter	37.94
Mtr.1203.1.S1_at	CCR4-associated factor-related protein	37.90
Mtr.9569.1.S1_at	Thaumatin-like protein	37.49
Mtr.44326.1.S1_at	Ephrin type-A receptor 5	37.22
Mtr.41268.1.S1_at	Serine/threonine-specific protein kinase APK2a	37.03
Mtr.5791.1.S1_at	Malonyl CoA:anthocyanin 3-O-glucoside-6"-O-malonyltransferase	37.02
Mtr.28311.1.S1_s_at	Hypothetical protein	36.99
Mtr.12662.1.S1_at	Patatin-like protein 1	36.92
Msa.886.1.S1_s_at	Hypothetical protein	36.59
Mtr.4593.1.S1_at	Histidine amino acid transporter	36.43
Mtr.18602.1.S1_at	Pathogenesis-related transcriptional factor	36.40
Mtr.48557.1.S1_at	Hypothetical protein	36.36
Mtr.40588.1.S1_at	Glutathione S-transferase GST 13	35.98
Mtr.12675.1.S1_at	Hypothetical protein	35.94
Mtr.43009.1.S1_s_at	Isoflavone 2'-hydroxylase, complete	35.63
Mtr.39214.1.S1_at	Hypothetical protein	35.43
Mtr.11007.1.S1_at	Avr9/Cf-9 rapidly elicited protein 132	34.71
Mtr.43199.1.S1_at	Hypothetical protein	34.46
Mtr.43280.1.S1_x_at	Cyanogenic Beta-Glucosidase	34.43
Mtr.42540.1.S1_at	Elicitor-inducible cytochrome P450	34.14
Mtr.34175.1.S1_at	Peroxidase 5	34.04
Mtr.20486.1.S1_at	Trp repressor/replication initiator	33.74
Mtr.9107.1.S1_at	Hypothetical protein	33.66
Mtr.15250.1.S1_at	ATPase	33.11
Mtr.10311.1.S1_at	Pprg2 protein	33.00
Mtr.12470.1.S1_at	Syringolide-induced protein 13-1-1	32.73
Mtr.10592.1.S1_at	Isoflavone reductase	32.49
Mtr.38241.1.S1_at	Hypothetical protein	32.49
Mtr.47795.1.S1_at	Hypothetical protein	32.46
Mtr.20618.1.S1_s_at	Hypothetical protein	32.37
Mtr.8666.1.S1_s_at	Hypothetical protein	32.31
Mtr.34121.1.S1_at	Hypothetical protein	32.31
Mtr.39679.1.S1_at	Hypothetical protein	32.27
Mtr.40605.1.S1_at	Xyloglucan-specific fungal endoglucanase inhibitor protein	32.17
Mtr.40412.1.S1_at	Hypothetical protein	31.96
Mtr.43569.1.S1_at	Medicago nodulin N21-like protein	31.87
Mtr.29306.1.S1_at	Hypothetical protein	31.57
Mtr.34720.1.S1_at	Hypothetical protein	31.52
Mtr.37253.1.S1_at	Amino acid transporter-like protein 1	31.51
Mtr.45572.1.S1_at	Hypothetical protein	31.21
Mtr.48647.1.S1_s_at	Cellulose synthase	31.20
Mtr.39165.1.S1_at	Cobalamin synthase	30.87
Mtr.41495.1.S1_at	Hypothetical protein	30.75
Mtr.46788.1.S1_at	L-allo-threonine aldolase	30.65
Mtr.34867.1.S1_s_at	Hypothetical protein	30.53

Mtr.40377.1.S1_at	Cysteine proteinase inhibitor	30.23
Mtr.12615.1.S1_at	Pathogenesis-related protein PR10A	29.95
Mtr.12723.1.S1_at	Hypothetical protein	29.90
Mtr.38072.1.S1_at	Hypothetical protein	29.63
Mtr.5486.1.S1_at	Hypothetical protein	29.25
Mtr.49999.1.S1_at	Hypothetical protein	29.13
Mtr.22459.1.S1_at	Hypothetical protein	28.90
Mtr.23575.1.S1_x_at	Polygalacturonase inhibitor protein	28.70
Mtr.12466.1.S1_at	Chitinase	28.59
Mtr.9819.1.S1_at	Hypothetical protein	28.50
Mtr.8559.1.S1_at	Hypothetical protein	28.49
Mtr.41091.1.S1_at	Mitogen-activated protein kinase MMK2	28.43
Mtr.41563.1.S1_s_at	Ring finger E3 ligase	28.37
Mtr.43193.1.S1_at	Glutamate dehydrogenase 3	28.36
Mtr.12485.1.S1_at	Hypothetical protein	28.29
Mtr.38067.1.S1_at	Hypothetical protein	28.24
Mtr.26304.1.S1_at	Hypothetical protein	28.14
Mtr.37973.1.S1_at	Hypothetical protein	27.98
Mtr.6154.1.S1_s_at	Wall-associated kinase	27.87
Mtr.38997.1.S1_at	Serine/threonine protein kinase	27.79
Mtr.4996.1.S1_at	Hypothetical protein	27.62
Mtr.44504.1.S1_at	Beta-glucan-elicitor receptor	27.62
Mtr.31521.1.S1_at	Lysine and histidine specific transporter	27.34
Mtr.40339.1.S1_s_at	Hypothetical protein	27.16
Mtr.22423.1.S1_at	Hin1 like protein	27.01
Mtr.35430.1.S1_s_at	Hypothetical protein	26.84
Mtr.27130.1.S1_s_at	Hypothetical protein	26.76
Mtr.17570.1.S1_at	Zn-finger, RING protein	26.73
Mtr.12653.1.S1_at	Hypothetical protein	26.51
Mtr.39424.1.S1_at	Serine/threonine protein kinase	26.35
Mtr.3130.1.S1_at	Hypothetical protein	26.24
Mtr.39384.1.S1_at	Hypothetical protein	26.24
Mtr.1951.1.S1_s_at	Zinc finger protein	26.17
Mtr.8645.1.S1_at	Riboflavin biosynthesis protein ribA	26.16
Mtr.15278.1.S1_s_at	Zn-finger, C2H2 type	26.08
Mtr.41237.1.S1_at	Nt-gh3 deduced protein	25.61
Mtr.32914.1.S1_at	Ethylene-induced esterase	25.61
Mtr.12906.1.S1_at	Glutamate decarboxylase 4a	25.30
Mtr.10689.1.S1_at	Translation factor EF-1 alpha	25.29
Mtr.15525.1.S1_at	Dynein light chain	25.17
Mtr.11959.1.S1_at	Serine/threonine protein phosphatase 2A	25.15
Mtr.8725.1.S1_at	Hypothetical protein	25.05
Mtr.33508.1.S1_s_at	Medicago nodulin N21-like protein	25.00
Mtr.43769.1.S1_at	Seed imbibition protein	24.83
Mtr.281.1.S1_at	Trehalose-6-phosphate phosphatase	24.82
Mtr.11550.1.S1_at	Diacylglycerol kinase variant B	24.82
Mtr.10079.1.S1_at	Hypothetical protein	24.80
Mtr.1299.1.S1_s_at	Cytochrome P450 93B1	24.79
Mtr.6834.1.S1_at	Zeatin O-glucosyltransferase	24.75
Mtr.7570.1.S1_at	Hypothetical protein	24.70
Mtr.27711.1.S1_at	Hypothetical protein	24.69
Mtr.40763.1.S1_at	Hypothetical protein	24.66
Mtr.15339.1.S1_at	Homeodomain-like protein	24.46
Mtr.40340.1.S1_at	Alkaline alpha galactosidase I	24.38

Mtr.29300.1.S1_s_at	Hypothetical protein	24.35
Mtr.1493.1.S1_at	Hypothetical protein	24.08
Mtr.6654.1.S1_at	Hypothetical protein	24.03
Mtr.11947.1.S1_at	Early growth response protein	23.95
Mtr.12710.1.S1_at	Syringolide-induced protein B13-1-1	23.95
Msa.1042.1.S1_at	Hypothetical protein	23.89
Mtr.14283.1.S1_at	Aminotransferase class-III	23.83
Mtr.17788.1.S1_at	Hypothetical protein	23.76
Mtr.43513.1.S1_at	Syringolide-induced protein 19-1-5	23.72
Msa.1604.1.S1_at	Hypothetical protein	23.64
Mtr.9223.1.S1_at	Ethylene reponse factor-like AP2 domain transcription factor	23.58
Mtr.38844.1.S1_at	Hypothetical protein	23.46
Msa.3005.1.S1_at	Hypothetical protein	23.43
Mtr.49305.1.S1_at	Fatty acid elongase	23.24
Mtr.38654.1.S1_at	Hypothetical protein	23.02
Mtr.40195.1.S1_at	Pectin methylesterase 5	22.79
Mtr.31314.1.S1_at	NAC-type DNA binding protein	22.65
Mtr.44416.1.S1_at	WRKY-type DNA binding protein	22.64
Mtr.33206.1.S1_s_at	Lustrin A-like protein	22.43
Mtr.11520.1.S1_at	Protein phosphatase 2C	22.37
Mtr.39059.1.S1_at	Hypothetical protein	22.28
Mtr.9661.1.S1_at	Scarecrow-like 14	22.24
Mtr.18629.1.S1_at	Hypothetical protein	22.21
Mtr.50682.1.S1_at	14-3-3 protein	22.07
Mtr.26629.1.S1_at	CCR4-associated factor 1-related protein	21.97
Mtr.49572.1.S1_x_at	Naringenin-chalcone synthase	21.91
Mtr.25007.1.S1_at	Vestitone reductase	21.89
Mtr.43646.1.S1_at	Hypothetical protein	21.86
Mtr.41694.1.S1_s_at	AP2 domain transcription factor	21.86
Mtr.27129.1.S1_at	Zinc finger protein	21.78
Mtr.37579.1.S1_at	Branched chain alpha-keto acid dehydrogenase E2 subunit	21.74
Mtr.11219.1.S1_at	Hypothetical protein	21.66
Mtr.9254.1.S1_at	Phe ammonia lyase	21.57
Mtr.38121.1.S1_at	Avr9/Cf-9 rapidly elicited protein	21.52
Mtr.48287.1.S1_s_at	Hypothetical protein	21.51
Mtr.39677.1.S1_x_at	Immunoglobulin heavy chain variable region	21.51
Mtr.39487.1.S1_s_at	Lipoxygenase	21.45
Mtr.43916.1.S1_at	Isoflavone reductase	21.39
Mtr.37455.1.S1_at	Glucosyltransferase-13	21.35
Mtr.43921.1.S1_at	Hypothetical protein	21.14
Mtr.38270.1.S1_at	ABA-responsive protein	21.11
Mtr.20803.1.S1_at	Hypothetical protein	21.09
Mtr.10438.1.S1_at	Kunitz proteinase inhibitor-1	20.98
Mtr.18040.1.S1_at	Hypothetical protein	20.98
Mtr.22903.1.S1_at	Chitinase (Class II)	20.89
Mtr.43514.1.S1_s_at	Syringolide-induced protein 19-1-5	20.88
Msa.2919.1.S1_at	Hypothetical protein	20.83
Mtr.8646.1.S1_at	Nine-cis-epoxycarotenoid dioxygenase1	20.79
Mtr.25575.1.S1_x_at	Hypothetical protein	20.78
Mtr.42819.1.S1_at	Hypothetical protein	20.77
Mtr.6769.1.S1_at	Hypothetical protein	20.61
Mtr.15010.1.S1_s_at	Homeodomain-like	20.59
Mtr.11354.1.S1_at	Bacterial-induced peroxidase	20.55
Mtr.45768.1.S1_at	Serine/threonine protein kinase	20.49

Mtr.15332.1.S1_at	Homeodomain-like	20.48
Mtr.18.1.S1_at	Hypothetical protein	20.43
Mtr.33892.1.S1_at	Zinc finger protein constans-like 6	20.42
Mtr.40177.1.S1_at	Hypothetical protein	20.37
Mtr.9258.1.S1_at	Receptor-like protein kinase	20.37
Mtr.28194.1.S1_at	Flavonol 3-O-glucosyltransferase 5	20.34
Mtr.31288.1.S1_at	Isoflavonoid glucosyltransferase	20.33
Mtr.14737.1.S1_at	Protein kinase	20.28
Mtr.37377.1.S1_at	Hypothetical protein	20.10
Msa.3122.1.S1_at	Hypothetical protein	19.96
Mtr.12749.1.S1_at	Hypothetical protein	19.95
Mtr.42084.1.S1_at	Hypothetical protein	19.95
Mtr.40882.1.S1_at	Aldehyde dehydrogenase	19.92
Mtr.43278.1.S1_at	Cellulose synthase	19.92
Mtr.50276.1.S1_at	Phosphoinositide-binding clathrin adaptor	19.91
Mtr.51996.1.S1_at	Hypothetical protein	19.85
Mtr.19594.1.S1_at	Cyclin-like F-box	19.82
Mtr.32898.1.S1_at	Protein phosphatase 2C	19.79
Mtr.46200.1.S1_at	CCCH-type zinc finger protein	19.78
Mtr.39160.1.S1_at	Hypothetical protein	19.58
Mtr.41299.1.S1_at	Hypothetical protein	19.51
Mtr.35324.1.S1_at	Peroxidase 2	19.48
Mtr.10127.1.S1_s_at	Phospholipase	19.48
Mtr.10895.1.S1_at	Hypothetical protein	19.38
Mtr.5797.1.S1_at	Hypothetical protein	19.36
Mtr.38547.1.S1_at	Tuber-specific and sucrose-responsive element binding factor	19.34
Mtr.44391.1.S1_at	Cytochrome P450 82A2	19.31
Mtr.43585.1.S1_at	1-deoxy-D-xylulose 5-phosphate synthase 2	19.31
Mtr.43144.1.S1_at	Hypothetical protein	19.27
Mtr.41670.1.S1_at	F-box protein	19.23
Mtr.2844.1.S1_at	Hypothetical protein	19.20
Mtr.43280.1.S1_at	Cyanogenic Beta-Glucosidase	19.18
Mtr.50317.1.S1_at	Zn-finger, RING protein	19.11
Mtr.41653.1.S1_at	Mitochondrial phosphate translocator	19.09
Mtr.41841.1.S1_at	Hypothetical protein	19.05
Mtr.38200.1.S1_at	Hypothetical protein	19.00
Mtr.28801.1.S1_at	Caffeoyl-CoA O-methyltransferase	18.98
Mtr.12141.1.S1_at	Hypothetical protein	18.94
Mtr.7503.1.S1_at	Hypothetical protein	18.87
Mtr.16167.1.S1_at	Hypothetical protein	18.75
Mtr.43252.1.S1_at	Avr9/Cf-9 rapidly elicited protein	18.71
Mtr.26218.1.S1_s_at	Isovaleryl-CoA Dehydrogenase	18.66
Mtr.45405.1.S1_at	Hypothetical protein	18.60
Mtr.11656.1.S1_at	Hypothetical protein	18.59
Mtr.40481.1.S1_at	Isovaleryl-CoA Dehydrogenase	18.57
Mtr.6489.1.S1_at	Glutathione S-transferase GST 15	18.55
Mtr.10805.1.S1_at	Avr9/Cf-9 rapidly elicited protein 31	18.45
Mtr.34728.1.S1_at	Hypothetical protein	18.37
Mtr.8851.1.S1_at	Clt1	18.32
Mtr.38612.1.S1_at	Phe ammonia lyase	18.30
Mtr.41944.1.S1_at	Hypothetical protein	18.28
Mtr.1343.1.S1_at	Hypothetical protein	18.22
Mtr.12866.1.S1_at	Hypothetical protein	18.22
Mtr.43644.1.S1_at	Homeodomain transcription factor	18.22

Mtr.39677.1.S1_at	Immunoglobulin heavy chain variable region	18.17
Mtr.33150.1.S1_s_at	Syringolide-induced protein 19-1-5	18.12
Mtr.38181.1.S1_at	Expansin-like protein B	18.09
Mtr.12764.1.S1_at	AMP-binding protein	18.02
Mtr.38957.1.S1_at	Hypothetical protein	18.02
Mtr.16848.1.S1_at	Harpin-induced 1	17.95
Mtr.19367.1.S1_at	Zn-finger, C2H2 type	17.94
Mtr.12980.1.S1_at	Hypothetical protein	17.89
Mtr.41031.1.S1_at	4-coumarate-CoA ligase	17.81
Mtr.48791.1.S1_at	Cys-rich domain protein	17.77
Msa.3124.1.S1_at	Hypothetical protein	17.73
Mtr.37204.1.S1_at	Glutathione S-transferase GST 18	17.66
Mtr.38233.1.S1_at	DAZ-associated protein 1	17.66
Mtr.35763.1.S1_at	Hypothetical protein	17.60
Mtr.15180.1.S1_at	Glucose-6-phosphate dehydrogenase	17.58
Mtr.38564.1.S1_at	Nodulin-like protein	17.56
Mtr.10971.1.S1_s_at	Protein serine/threonine kinase	17.53
Mtr.38765.1.S1_at	LOB domain protein 38	17.52
Mtr.6352.1.S1_s_at	Alternative oxidase 3	17.51
Mtr.1156.1.S1_at	Rhicadhesin receptor precursor (Germin-like protein)	17.49
Mtr.33373.1.S1_at	Hypothetical protein	17.47
Mtr.24422.1.S1_at	Hypothetical protein	17.42
Mtr.41772.1.S1_at	AMP-binding protein	17.42
Mtr.8531.1.S1_at	Chalcone isomerase 1	17.40
Mtr.52169.1.S1_at	Hypothetical protein	17.31
Mtr.3060.1.S1_s_at	Hypothetical protein	17.26
Mtr.42703.1.S1_at	Hypothetical protein	17.25
Mtr.10696.1.S1_at	Syntaxin-related protein	17.21
Mtr.37736.1.S1_at	Hypothetical protein	17.21
Mtr.24896.1.S1_at	Hypothetical protein	17.20
Msa.1404.1.S1_at	Hypothetical protein	17.19
Mtr.40613.1.S1_at	Hypothetical protein	17.15
Mtr.39195.1.S1_at	Nitrate transporter NTL1	17.07
Mtr.8667.1.S1_at	Hypothetical protein	17.06
Mtr.51997.1.S1_at	Hypothetical protein	17.00
Mtr.49418.1.S1_s_at	Hypothetical protein	17.00
Mtr.11285.1.S1_at	WAK-like kinase	16.96
Mtr.13171.1.S1_at	Hypothetical protein	16.92
Mtr.45010.1.S1_at	Serine/threonine kinase	16.89
Mtr.43252.1.S1_s_at	Avr9/Cf-9 rapidly elicited protein 74	16.87
Mtr.37708.1.S1_at	Sulfate transporter-like protein	16.84
Mtr.6769.1.S1_s_at	Hypothetical protein	16.82
Msa.1879.1.S1_at	Hypothetical protein	16.82
Mtr.43115.1.S1_at	Pheromone receptor-like protein	16.81
Mtr.6373.1.S1_at	Acetyl-CoA carboxylase	16.80
Mtr.32279.1.S1_at	SPF1 protein	16.79
Mtr.28696.1.S1_at	Hypothetical protein	16.72
Msa.2935.1.S1_at	Hypothetical protein	16.72
Mtr.49862.1.S1_at	Isoflavone-7-o-methytransferase 9	16.71
Mtr.29368.1.S1_at	Receptor-like protein kinase	16.67
Mtr.3602.1.S1_at	Hypothetical protein	16.65
Mtr.43048.1.S1_at	Early nodulin 12A	16.63
Mtr.46130.1.S1_at	Hypothetical protein	16.63
Mtr.20185.1.S1_at	Naringenin-chalcone synthase	16.60

Mtr.5817.1.S1_at	Phospholipid-transporting ATPase 1	16.49
Mtr.39569.1.S1_at	Hypothetical protein	16.48
Mtr.40125.1.S1_at	Peroxidase1B	16.44
Msa.2506.1.S1_at	Hypothetical protein	16.43
Mtr.8538.1.S1_at	Plant regulatory factor 7	16.42
Mtr.8599.1.S1_s_at	Caffeic acid 3-O-methyltransferase 1	16.38
Mtr.45356.1.S1_at	Glutamate dehydrogenase	16.30
Msa.2701.1.S1_at	Hypothetical protein	16.26
Mtr.47086.1.S1_s_at	Glucan 1,3-beta-glucosidase	16.13
Mtr.28714.1.S1_at	Chalcone synthase 3	16.11
Mtr.44713.1.S1_at	Amino acid transporter	16.11
Mtr.43217.1.S1_at	Hypothetical protein	16.03
Mtr.19471.1.S1_at	Hypothetical protein	16.01
Mtr.24851.1.S1_at	Hypothetical protein	15.98
Mtr.33191.1.S1_at	Hypothetical protein	15.98
Mtr.5781.1.S1_at	Hypothetical protein	15.87
Mtr.6543.1.S1_at	Hypothetical protein	15.70
Mtr.26898.1.S1_at	Hypothetical protein	15.66
Mtr.12569.1.S1_at	Hypothetical protein	15.63
Mtr.38907.1.S1_at	Alcohol dehydrogenase	15.61
Mtr.38443.1.S1_at	Hypothetical protein	15.59
Mtr.32982.1.S1_at	MutT-like protein	15.51
Mtr.42620.1.S1_at	Organic anion transporter	15.51
Mtr.1886.1.S1_at	Hypothetical protein	15.51
Mtr.8696.1.S1_at	Zinc finger DNA-binding protein	15.46
Mtr.20185.1.S1_x_at	Naringenin-chalcone synthase	15.41
Mtr.32486.1.S1_at	Strubbelig receptor family 1	15.41
Mtr.12950.1.S1_at	Hypothetical protein	15.39
Mtr.34762.1.S1_s_at	Hypothetical protein	15.35
Mtr.40333.1.S1_x_at	Uclacyanin II precursor (Blue copper-binding protein II)	15.34
Mtr.6916.1.S1_s_at	Hypothetical protein	15.32
Mtr.11066.1.S1_at	Hypothetical protein	15.32
Mtr.9788.1.S1_at	ACC synthase	15.31
Mtr.12006.1.S1_at	Bacterial-induced peroxidase	15.28
Mtr.38485.1.S1_at	Hypothetical protein	15.26
Mtr.25967.1.S1_at	Hypothetical protein	15.25
Mtr.52180.1.S1_at	Zn-finger, C2H2 type	15.21
Msa.2722.1.S1_at	Hypothetical protein	15.18
Mtr.11156.1.S1_at	Leucine-rich repeat receptor-like protein kinase 1	15.15
Mtr.17940.1.S1_at	U box protein	15.07
Mtr.49085.1.S1_at	GCN5-related N-acetyltransferase	15.07
Mtr.45331.1.S1_at	Hypothetical protein	15.02
Msa.2755.1.S1_at	Hypothetical protein	14.97
Mtr.42663.1.S1_at	Ferredoxin-dependent glutamate synthase	14.96
Mtr.45315.1.S1_at	SPF1 protein	14.89
Mtr.39065.1.S1_at	Hypothetical protein	14.84
Mtr.4724.1.S1_at	Organic anion transporter	14.83
Mtr.6648.1.S1_s_at	Xyloglucan endotransglycosylase	14.74
Mtr.12663.1.S1_s_at	Patatin-like protein 1	14.72
Mtr.29494.1.S1_at	Pectin methylesterase 9	14.71
Mtr.31589.1.S1_at	Arm repeat protein	14.70
Mtr.37494.1.S1_at	Phi-1 protein	14.68
Mtr.45193.1.S1_at	Hypothetical protein	14.60
Mtr.39563.1.S1_at	Hypothetical protein	14.51

Mtr.14281.1.S1_at	Hypothetical protein	14.49
Mtr.8831.1.S1_at	Patatin-like protein 1	14.46
Mtr.11636.1.S1_at	Dof zinc finger protein	14.46
Mtr.8751.1.S1_at	Hypothetical protein	14.41
Mtr.8975.1.S1_at	Bifunctional lysine-ketoglutarate reductase/saccharopine dehydrogenase	14.37
Msa.1878.1.S1_at	Hypothetical protein	14.37
Mtr.20079.1.S1_at	Lipoxygenase	14.36
Mtr.43561.1.S1_s_at	Calcium/calmodulin-regulated receptor-like kinase	14.36
Mtr.51989.1.S1_s_at	Cyclic nucleotide-binding; K+ channel	14.33
Mtr.9486.1.S1_at	Zinc finger protein CONSTANS-LIKE 10	14.33
Mtr.11713.1.S1_at	BRASSINOSTEROID INSENSITIVE 1-associated receptor kinase 1 (BAK1)	14.29
Mtr.32409.1.S1_at	Galactokinase like protein	14.26
Mtr.41563.1.S1_at	Ring finger E3 ligase	14.25
Mtr.44776.1.S1_at	Hypothetical protein	14.21
Mtr.37757.1.S1_at	Anthranilate N-benzoyltransferase-like protein	14.20
Mtr.35927.1.S1_at	Maturase K	14.19
Mtr.40555.1.S1_at	Osmotin-like protein	14.19
Mtr.28554.1.S1_at	Hypothetical protein	14.13
Mtr.40838.1.S1_at	Electron transfer flavoprotein, alpha subunit	14.12
Msa.2738.1.S1_at	Hypothetical protein	14.11
Mtr.31946.1.S1_at	Hypothetical protein	14.03
Mtr.1356.1.S1_s_at	Hypothetical protein	14.00
Mtr.37578.1.S1_at	Hypothetical protein	13.97
Mtr.37520.1.S1_at	Cinnamoyl CoA reductase	13.89
Mtr.14686.1.S1_at	Tetrapeptide transporter	13.78
Mtr.653.1.S1_s_at	Short-chain dehydrogenase/reductase family protein	13.78
Mtr.12139.1.S1_at	WRKY transcription factor 29	13.72
Mtr.11520.1.S1_s_at	Protein phosphatase 2C	13.71
Mtr.27950.1.S1_at	Hypothetical protein	13.69
Mtr.10557.1.S1_at	PDR-type ABC transporter 2	13.66
Msa.1770.1.S1_at	Hypothetical protein	13.66
Mtr.41282.1.S1_s_at	TMV response-related protein	13.62
Mtr.17903.1.S1_at	Hypothetical protein	13.58
Mtr.44519.1.S1_at	Hypothetical protein	13.57
Mtr.5445.1.S1_at	Hypothetical protein	13.57
Mtr.45912.1.S1_at	Pathogenesis-related transcriptional factor, ERF	13.53
Mtr.33213.1.S1_at	Hypothetical protein	13.52
Mtr.41861.1.S1_at	Acetyl-CoA carboxylase	13.44
Mtr.6049.1.S1_s_at	S-receptor kinase	13.43
Mtr.39298.1.S1_at	Hypothetical protein	13.39
Mtr.11053.1.S1_at	WRKY-type transcription factor	13.38
Mtr.32790.1.S1_at	Hypothetical protein	13.34
Mtr.8962.1.S1_at	Hypothetical protein	13.33
Mtr.11256.1.S1_at	Hypothetical protein	13.32
Mtr.27510.1.S1_at	Blight resistance protein RGA1	13.25
Mtr.1359.1.S1_at	Hypothetical protein	13.23
Mtr.12246.1.S1_at	MtN12 protein	13.21
Mtr.33334.1.S1_at	Exo-1,3-beta-glucanase	13.20
Mtr.6156.1.S1_at	Cytochrome oxidase subunit II	13.17
Mtr.6909.1.S1_at	Hypothetical protein	13.16
Msa.3019.1.S1_at	Hypothetical protein	13.16
Mtr.1331.1.S1_s_at	Hypothetical protein	13.16
Msa.2819.1.S1_at	Hypothetical protein	13.15
Mtr.18113.1.S1_at	Hypothetical protein	13.11

Mtr.38479.1.S1_at	Hypothetical protein	13.07
Mtr.25964.1.S1_s_at	Hypothetical protein	13.02
Mtr.29505.1.S1_s_at	Hypothetical protein	12.99
Mtr.14016.1.S1_at	Hypothetical protein	12.98
Mtr.29902.1.S1_at	WRKY DNA-binding protein	12.98
Mtr.44641.1.S1_at	F-box family protein	12.97
Mtr.10683.1.S1_at	DNA-binding proteim	12.97
Mtr.35697.1.S1_s_at	Hypothetical protein	12.96
Mtr.11572.1.S1_at	Receptor-like protein kinase	12.88
Mtr.9023.1.S1_s_at	MYB transcription factor	12.86
Mtr.43231.1.S1_s_at	Hypothetical protein	12.84
Mtr.22536.1.S1_at	Hypothetical protein	12.77
Mtr.8948.1.S1_at	Cytochrome P450 82A1	12.76
Mtr.35302.1.S1_at	Galactokinase	12.75
Mtr.43392.1.S1_s_at	Cytochrome b	12.73
Mtr.38380.1.S1_at	Nodulin-like protein	12.70
Mtr.48070.1.S1_at	Hypothetical protein	12.68
Mtr.11022.1.S1_at	RING zinc finger protein	12.65
Mtr.40363.1.S1_at	Fiber protein	12.60
Mtr.35178.1.S1_at	Branched-chain alpha keto-acid dehydrogenase E1-alpha	12.58
Mtr.44445.1.S1_at	Syringolide-induced protein B13-1-9	12.51
Mtr.40983.1.S1_at	Hypothetical protein	12.49
Mtr.28045.1.S1_at	Hypothetical protein	12.48
Mtr.47086.1.S1_at	Glucan 1,3-beta-glucosidase	12.48
Mtr.47087.1.S1_at	Glucan 1,3-beta-glucosidase	12.47
Mtr.43734.1.S1_at	Protein kinase C	12.45
Mtr.13850.1.S1_at	Hypothetical protein	12.42
Mtr.20425.1.S1_at	Hypothetical protein	12.41
Mtr.13536.1.S1_at	Bifunctional lysine-ketoglutarate reductase/saccharopine dehydrogenase	12.38
Mtr.12477.1.S1_at	Quinone-oxidoreductase QR1	12.38
Mtr.50729.1.S1_at	Hypothetical protein	12.35
Mtr.39945.1.S1_at	Hypothetical protein	12.32
Mtr.46166.1.S1_at	Isopenicillin N synthase	12.27
Mtr.8688.1.S1_at	Hypothetical protein	12.27
Mtr.39874.1.S1_at	Hypothetical protein	12.19
Mtr.13270.1.S1_at	Hypothetical protein	12.19
Mtr.33024.1.S1_at	Glucosidase-like protein	12.18
Mtr.43987.1.S1_at	Serin carboxypeptidase	12.17
Mtr.6950.1.S1_at	Galactokinase like protein	12.16
Mtr.43716.1.S1_at	Glycosyltransferase	12.14
Mtr.20187.1.S1_x_at	Naringenin-chalcone synthase	12.12
Mtr.32790.1.S1_s_at	Hypothetical protein	12.12
Mtr.41894.1.S1_at	AT-hook DNA-binding protein	12.12
Mtr.11466.1.S1_at	Galactokinase like protein	12.09
Mtr.42141.1.S1_s_at	Class III peroxidase	12.09
Mtr.8599.1.S1_at	Caffeic acid 3-O-methyltransferase 1	12.08
Mtr.35.1.S1_at	Abscisic acid and environmental stress inducible protein	12.08
Mtr.26939.1.S1_at	Hypothetical protein	12.07
Mtr.1757.1.S1_at	Homeodomain-leucine zipper protein	12.07
Mtr.27096.1.S1_at	Cinnamyl-alcohol dehydrogenase-like protein	12.06
Mtr.9450.1.S1_at	Axi 1-like protein	12.02
Mtr.2465.1.S1_at	Hypothetical protein	12.02
Mtr.39450.1.S1_at	Amino acid permease AAP3	12.02
Mtr.10743.1.S1_at	SRG1 protein	11.96

Mtr.40830.1.S1_at	2-hydroxyphytanoyl-CoA lyase	11.95
Mtr.43229.1.S1_at	Syringolide-induced protein 14-1-1	11.95
Mtr.8522.1.S1_at	Syringolide-induced protein 19-1-5	11.93
Mtr.10043.1.S1_at	Wax synthase	11.92
Mtr.19134.1.S1_at	Hypothetical protein	11.86
Mtr.14773.1.S1_at	Hypothetical protein	11.85
Mtr.12155.1.S1_at	WRKY-type DNA binding protein	11.85
Mtr.39431.1.S1_at	Aldehyde reductase	11.84
Msa.2171.1.S1_at	Hypothetical protein	11.81
Mtr.20144.1.S1_at	Multi antimicrobial extrusion protein	11.77
Mtr.12832.1.S1_at	Receptor-like serine/threonine kinase	11.76
Mtr.5684.1.S1_at	Hypothetical protein	11.74
Mtr.36186.1.S1_at	Nitrate transporter NTL1	11.74
Mtr.35243.1.S1_at	Early flowering 3	11.74
Mtr.44775.1.S1_at	G-protein alpha-subunit	11.74
Mtr.9524.1.S1_at	Early flowering 3	11.73
Mtr.25953.1.S1_at	Cytidine deaminase	11.66
Msa.2910.1.S1_at	Hypothetical protein	11.65
Mtr.6341.1.S1_at	Beta-1, 3-glucanase	11.64
Mtr.23311.1.S1_s_at	Glycosyl hydrolase family 3	11.63
Mtr.12456.1.S1_at	AP2-related transcription factor	11.61
Mtr.42141.1.S1_at	Class III peroxidase	11.61
Mtr.38492.1.S1_at	Hypothetical protein	11.55
Mtr.12044.1.S1_at	Cellulose synthase	11.51
Mtr.8934.1.S1_at	Alternative oxidase 3	11.50
Mtr.17288.1.S1_at	Natural resistance-associated macrophage protein	11.50
Mtr.11066.1.S1_s_at	Hypothetical protein	11.49
Mtr.28274.1.S1_at	Avr9/Cf-9 rapidly elicited protein 264	11.46
Mtr.44735.1.S1_at	Hypothetical protein	11.46
Mtr.40168.1.S1_at	Phenylalanine ammonia-lyase 2	11.38
Mtr.37413.1.S1_s_at	Chalcone-flavonone isomerase	11.31
Mtr.7214.1.S1_at	Hypothetical protein	11.24
Mtr.44074.1.S1_at	Strubbelig receptor family 1	11.20
Mtr.28687.1.S1_at	Wax synthase	11.17
Mtr.27221.1.S1_at	Hypothetical protein	11.17
Mtr.45211.1.S1_at	Phosphoenolpyruvate carboxykinase	11.14
Mtr.10815.1.S1_at	Hypothetical protein	11.12
Mtr.10370.1.S1_at	Peroxidase1B	11.12
Mtr.20391.1.S1_at	Hypothetical protein	11.09
Mtr.45320.1.S1_at	Hypothetical protein	11.07
Mtr.21324.1.S1_at	ABC transporter	11.06
Mtr.25509.1.S1_at	Hypothetical protein	11.00
Mtr.43497.1.S1_at	Ethylene-forming-enzyme-like dioxygenase	11.00
Mtr.4464.1.S1_at	Thaumatin-like protein 1	11.00
Mtr.38075.1.S1_at	Hypothetical protein	10.97
Mtr.35852.1.S1_at	Auxin-induced protein	10.96
Msa.2921.1.S1_at	Hypothetical protein	10.89
Mtr.51734.1.S1_at	Hypothetical protein	10.88
Mtr.25838.1.S1_at	Hypothetical protein	10.88
Mtr.42860.1.S1_at	Ferredoxin III	10.85
Mtr.11866.1.S1_at	MFS Superfamily transporter	10.82
Mtr.49572.1.S1_s_at	Naringenin-chalcone synthase	10.79
Mtr.32646.1.S1_at	Hypothetical protein	10.79
Mtr.20623.1.S1_at	Hypothetical protein	10.75

Mtr.9901.1.S1_at	Receptor-like kinase	10.75
Mtr.27985.1.S1_at	Hypothetical protein	10.72
Mtr.11468.1.S1_at	Hypothetical protein	10.71
Mtr.34990.1.S1_at	Cytochrome P450	10.70
Mtr.15568.1.S1_s_at	DNA-binding WRKY	10.69
Mtr.16487.1.S1_s_at	Zn-finger, CCHC type	10.66
Mtr.11991.1.S1_s_at	Strubbelig receptor family 3	10.64
Mtr.6317.1.S1_at	Hypothetical protein	10.63
Mtr.9637.1.S1_at	Myb-related transcription factor	10.63
Mtr.37715.1.S1_at	Hypothetical protein	10.63
Mtr.44104.1.S1_at	Zeatin O-glucosyltransferase	10.62
Mtr.9997.1.S1_at	Glucan endo-1,3-beta-glucosidase	10.60
Msa.2884.1.S1_at	Hypothetical protein	10.58
Mtr.40521.1.S1_at	Adhesive/proline-rich protein	10.53
Mtr.40917.1.S1_at	SPF1 protein	10.52
Mtr.11621.1.S1_at	Receptor-like protein kinase	10.51
Mtr.21790.1.S1_at	Hypothetical protein	10.49
Mtr.43230.1.S1_at	Hypothetical protein	10.48
Mtr.17239.1.S1_at	Metallopeptidases	10.48
Mtr.17180.1.S1_at	Galactose mutarotase	10.45
Mtr.19019.1.S1_at	No apical meristem (NAM) protein	10.44
Mtr.16851.1.S1_at	Serine/threonine protein kinase	10.43
Mtr.4899.1.S1_at	Hypothetical protein	10.37
Mtr.12087.1.S1_at	Hypothetical protein	10.36
Mtr.21276.1.S1_at	TAZ finger	10.34
Mtr.33365.1.S1_s_at	PDR7 ABC transporter	10.33
Mtr.45159.1.S1_at	Hypothetical protein	10.32
Mtr.38607.1.S1_at	Hypothetical protein	10.31
Mtr.32738.1.S1_s_at	Myo-inositol oxygenase	10.31
Mtr.35538.1.S1_at	Avr9/Cf-9 rapidly elicited protein 137	10.30
Mtr.13757.1.S1_at	Hin1-like protein	10.29
Mtr.37714.1.S1_at	Syringolide-induced protein 13-1-1	10.28
Mtr.2157.1.S1_s_at	Hypothetical protein	10.28
Mtr.43746.1.S1_at	Hypothetical protein	10.24
Mtr.31.1.S1_at	Peroxidase 3	10.22
Mtr.6811.1.S1_at	Glutathione S-transferase GST 17	10.20
Mtr.34018.1.S1_at	S-receptor kinase	10.20
Mtr.37718.1.S1_at	Hypothetical protein	10.18
Mtr.51734.1.S1_s_at	Hypothetical protein	10.17
Mtr.35110.1.S1_at	Hypothetical protein	10.16
Mtr.23266.1.S1_at	Protease inhibitor	10.15
Mtr.45444.1.S1_at	Hypothetical protein	10.14
Mtr.40123.1.S1_at	Chalcone synthase 4	10.14
Mtr.44228.1.S1_at	Cyanophycinase	10.14
Mtr.10575.1.S1_at	Hypothetical protein	10.12
Mtr.35357.1.S1_at	Auxin-regulated gene	10.10
Mtr.8853.1.S1_at	Arginase 1	10.09
Mtr.8763.1.S1_at	Thaumatin-like protein PR-5a	10.06
Mtr.45244.1.S1_at	Hypothetical protein	10.06
Mtr.45989.1.S1_at	Xylose isomerase	10.05
Mtr.49688.1.S1_at	Heavy metal-associated domain protein	10.05
Mtr.12291.1.S1_s_at	Proline dehydrogenase	10.04
Mtr.37975.1.S1_at	CBL-interacting protein kinase	10.01
Mtr.42263.1.S1_at	Hypothetical protein	9.98

Mtr.50458.1.S1_at	Hypothetical protein	9.98
Mtr.32564.1.S1_at	Hypothetical protein	9.97
Mtr.15309.1.S1_at	Hypothetical protein	9.91
Mtr.45080.1.S1_at	Retinoic acid receptor alpha	9.91
Msa.1625.1.S1_at	Hypothetical protein	9.91
Mtr.10727.1.S1_at	Hypothetical protein	9.89
Msa.1669.1.S1_at	Hypothetical protein	9.89
Mtr.18222.1.S1_at	Hypothetical protein	9.84
Mtr.11811.1.S1_s_at	Phi-1-like protein	9.81
Mtr.17531.1.S1_at	Hypothetical protein	9.79
Mtr.31118.1.S1_at	Serine /threonine kinase	9.79
Mtr.10416.1.S1_at	3-deoxy-D-arabino-heptulosonate 7-phosphate synthase	9.79
Mtr.38566.1.S1_at	Hypothetical protein	9.78
Mtr.48695.1.S1_at	Hypothetical protein	9.78
Mtr.39456.1.S1_at	Coatomer protein complex, beta prime; beta'-COP protein	9.77
Mtr.41533.1.S1_at	Calcium binding protein	9.76
Mtr.13318.1.S1_at	Hypothetical protein	9.76
Mtr.40277.1.S1_x_at	Isoflavone synthase	9.73
Mtr.32284.1.S1_at	Hypothetical protein	9.73
Mtr.18596.1.S1_at	Zn-finger, RING	9.69
Mtr.13960.1.S1_at	Flavonoid 3'-hydroxylase	9.69
Mtr.44073.1.S1_at	En/Spm-like transposon protein	9.57
Mtr.13722.1.S1_at	Hypothetical protein	9.57
Mtr.47442.1.S1_s_at	Glycosyl hydrolase family 3	9.53
Mtr.4083.1.S1_s_at	Hypothetical protein	9.52
Mtr.7255.1.S1_at	Arm repeat protein	9.48
Mtr.44658.1.S1_at	Hypothetical protein	9.47
Mtr.40277.1.S1_at	Isoflavone synthase	9.47
Msa.2861.1.S1_at	Hypothetical protein	9.47
Mtr.29335.1.S1_at	Receptor-like protein kinase ARK	9.47
Mtr.40399.1.S1_at	CPRD2 protein	9.46
Mtr.20567.1.S1_at	Naringenin-chalcone synthase	9.45
Mtr.35987.1.S1_s_at	Hypothetical protein	9.44
Mtr.20618.1.S1_at	Hypothetical protein	9.44
Mtr.48831.1.S1_at	Peptidase aspartic	9.42
Mtr.7268.1.S1_at	Hypothetical protein	9.41
Msa.2680.1.S1_at	Hypothetical protein	9.40
Mtr.10121.1.S1_at	Limonoid UDP-glucosyltransferase	9.38
Mtr.1746.1.S1_at	Glucosyltransferase-9	9.36
Mtr.8936.1.S1_at	Zinc finger protein	9.36
Mtr.37390.1.S1_at	Pyruvate decarboxylase 1	9.36
Mtr.42202.1.S1_at	Hypothetical protein	9.35
Mtr.17497.1.S1_at	bHLH DNA-binding	9.34
Mtr.29510.1.S1_at	Hypothetical protein	9.32
Mtr.37879.1.S1_at	Hypothetical protein	9.32
Mtr.25476.1.S1_at	Auxin efflux carrier protein family	9.31
Mtr.689.1.S1_at	FAD-linked oxidoreductase	9.31
Mtr.22561.1.S1_at	Hypothetical protein	9.31
Mtr.43827.1.S1_at	Hypothetical protein	9.30
Mtr.5955.1.S1_at	Hypothetical protein	9.25
Mtr.52242.1.S1_at	Galactokinase	9.25
Mtr.49123.1.S1_at	Major intrinsic protein	9.24
Mtr.40194.1.S1_at	Hypothetical protein	9.23
Mtr.40223.1.S1_at	Hypothetical protein	9.23

Mtr.41299.1.S1_s_at	Hypothetical protein	9.22
Mtr.3431.1.S1_at	Hypothetical protein	9.22
Mtr.50225.1.S1_at	Hypothetical protein	9.22
Mtr.25969.1.S1_at	Hypothetical protein	9.21
Mtr.18322.1.S1_s_at	Alternative oxidase	9.21
Mtr.42916.1.S1_at	Hypothetical protein	9.20
Mtr.49481.1.S1_at	Hypothetical protein	9.19
Mtr.22708.1.S1_at	Hypothetical protein	9.18
Mtr.24516.1.S1_at	Basic blue copper protein	9.17
Mtr.5869.1.S1_at	Hexose carrier protein HEX6	9.17
Mtr.12511.1.S1_at	Heat shock factor RHSF2	9.15
Mtr.37878.1.S1_at	Hypothetical protein	9.14
Mtr.28727.1.S1_at	Adenosine monophosphate binding protein 1 AMPBP1	9.13
Mtr.12303.1.S1_at	Hypothetical protein	9.13
Mtr.49055.1.S1_s_at	Hypothetical protein	9.11
Mtr.24370.1.S1_at	3'-5' exonuclease	9.09
Mtr.11712.1.S1_at	NADH dehydrogenase subunit 6	9.07
Mtr.43783.1.S1_at	Hypothetical protein	9.06
Mtr.9364.1.S1_at	Hypothetical protein	9.05
Mtr.25365.1.S1_at	Hypothetical protein	9.05
Mtr.31724.1.S1_at	Hypothetical protein	9.04
Mtr.32367.1.S1_s_at	Peroxidase 3	9.01
Mtr.12025.1.S1_at	Bacterial-induced peroxidase	9.01
Mtr.11502.1.S1_at	Hypothetical protein	9.00
Mtr.1625.1.S1_s_at	Protein kinase	8.99
Mtr.34118.1.S1_at	Cationic peroxidase	8.96
Mtr.20187.1.S1_at	Naringenin-chalcone synthase	8.96
Mtr.3072.1.S1_at	Disease resistance protein Cf-2.1-like	8.96
Mtr.12685.1.S1_at	Calcium/calmodulin-regulated receptor-like kinase	8.91
Mtr.34771.1.S1_at	Hypothetical protein	8.87
Mtr.44219.1.S1_at	Cytokinin oxidase-like protein	8.86
Mtr.6339.1.S1_at	Ripening regulated protein	8.85
Msa.1050.1.S1_at	Hypothetical protein	8.85
Mtr.48873.1.S1_s_at	Plant acid phosphatase	8.82
Mtr.40166.1.S1_s_at	Phenylalanine ammonia-lyase	8.81
Mtr.10492.1.S1_x_at	Isoflavone synthase	8.80
Mtr.44094.1.S1_at	Hypothetical protein	8.78
Mtr.44967.1.S1_at	Hypothetical protein	8.78
Mtr.48304.1.S1_s_at	Hypothetical protein	8.75
Mtr.16822.1.S1_at	Hypothetical protein	8.74
Mtr.10817.1.S1_at	Hypothetical protein	8.73
Mtr.31312.1.S1_at	Receptor ser/thr protein kinase	8.73
Mtr.41039.1.S1_at	Hypothetical protein	8.72
Msa.952.1.S1_at	Hypothetical protein	8.71
Mtr.38202.1.S1_at	WRKY transcription factor 28	8.70
Mtr.12538.1.S1_at	Hypothetical protein	8.69
Mtr.40371.1.S1_at	Avr9/Cf-9 rapidly elicited protein 264	8.69
Mtr.42135.1.S1_at	Photoperiod responsive protein	8.65
Mtr.7906.1.S1_at	Vasopressin V1b receptor	8.65
Mtr.19182.1.S1_at	Hypothetical protein	8.64
Mtr.5738.1.S1_s_at	Hypothetical protein	8.64
Mtr.39665.1.S1_at	Hypothetical protein	8.63
Mtr.4576.1.S1_s_at	Indole-3-acetate beta-glucosyltransferase	8.62
Mtr.28033.1.S1_at	Hypothetical protein	8.62

Mtr.43944.1.S1_at	Glucan 1,3-beta-glucosidase	8.62
Mtr.41518.1.S1_at	Hypothetical protein	8.61
Mtr.28177.1.S1_at	Hypothetical protein	8.61
Mtr.38328.1.S1_at	Phosphatidic acid phosphatase alpha	8.59
Mtr.45000.1.S1_at	Trehalose-6-phosphate synthase/phosphatase	8.59
Mtr.29535.1.S1_x_at	Hypothetical protein	8.57
Mtr.49053.1.S1_s_at	Hypothetical protein	8.55
Mtr.12051.1.S1_at	Hypothetical protein	8.53
Mtr.34126.1.S1_at	Hypothetical protein	8.52
Mtr.12377.1.S1_at	WRKY transcription factor 11	8.49
Mtr.15380.1.S1_at	Hypothetical protein	8.49
Mtr.41367.1.S1_at	Hypothetical protein	8.48
Mtr.6933.1.S1_at	Methylcrotonyl-CoA carboxylase beta chain	8.48
Mtr.37262.1.S1_at	Hypothetical protein	8.47
Mtr.45741.1.S1_at	Cytochrome P450	8.47
Mtr.12708.1.S1_at	Isoflavone reductase	8.46
Mtr.6889.1.S1_at	Hypothetical protein	8.45
Mtr.30703.1.S1_at	Hypothetical protein	8.42
Mtr.35080.1.S1_at	Trehalose-6-phosphate synthase	8.39
Mtr.11349.1.S1_at	Transcription factor WRKY31	8.39
Mtr.23988.1.S1_at	bHLH protein	8.39
Mtr.41551.1.S1_at	SCARECROW-like protein	8.38
Mtr.39741.1.S1_s_at	Hypothetical protein	8.37
Msa.1166.1.S1_at	Hypothetical protein	8.37
Mtr.48109.1.S1_at	WRKY4 transcription factor	8.35
Mtr.13586.1.S1_at	Transcription activator	8.34
Mtr.33124.1.S1_at	Acyl-activating enzyme	8.33
Mtr.35410.1.S1_at	Rho GDP dissociation inhibitor 2	8.33
Msa.1898.1.S1_at	Hypothetical protein	8.33
Mtr.37899.1.S1_at	Hypothetical protein	8.32
Mtr.24820.1.S1_s_at	Fructan 1-exohydrolase	8.32
Mtr.49557.1.S1_at	Hypothetical protein	8.31
Mtr.4233.1.S1_at	Hypothetical protein	8.30
Mtr.16688.1.S1_at	Mitochondrial import inner membrane translocase	8.30
Mtr.51693.1.S1_at	Hypothetical protein	8.30
Mtr.43956.1.S1_at	Zinc finger family protein	8.28
Mtr.40410.1.S1_at	Hypothetical protein	8.25
Mtr.43494.1.S1_at	Hypothetical protein	8.25
Mtr.17422.1.S1_at	Amino acid/polyamine transporter I	8.24
Mtr.19625.1.S1_at	Hypothetical protein	8.24
Mtr.16539.1.S1_s_at	Hypothetical protein	8.24
Mtr.48475.1.S1_at	Hypothetical protein	8.23
Mtr.11488.1.S1_at	Hypothetical protein	8.22
Mtr.22179.1.S1_at	Hypothetical protein	8.22
Mtr.18819.1.S1_at	2OG-Fe(II) oxygenase	8.21
Mtr.29408.1.S1_at	Hypothetical protein	8.20
Mtr.42235.1.S1_at	Beta-glucan binding protein	8.20
Mtr.13918.1.S1_at	Hypothetical protein	8.20
Msa.3160.1.S1_at	Hypothetical protein	8.16
Mtr.10005.1.S1_at	Hypothetical protein	8.16
Mtr.39423.1.S1_at	Hypothetical protein	8.14
Mtr.12648.1.S1_at	Ovary protein induced by treatment with gibberellic acid	8.13
Mtr.696.1.S1_at	Auxin-induced (indole-3-acetic acid induced) protein	8.13
Mtr.28786.1.S1_at	Dihydrodipicolinate synthase	8.12

Mtr.22904.1.S1_s_at	Aux/IAA protein	8.11
Mtr.820.1.S1_at	Hypothetical protein	8.11
Mtr.13641.1.S1_at	Alpha-mannosidase	8.08
Mtr.8813.1.S1_at	Zinc finger protein constans-like 6	8.07
Mtr.39825.1.S1_at	NADH dehydrogenase, subunit 2	8.07
Msa.1267.1.S1_at	Hypothetical protein	8.05
Mtr.32917.1.S1_s_at	Cytochrome oxidase subunit I	8.04
Mtr.13455.1.S1_at	Hypothetical protein	8.03
Msa.1067.1.S1_at	Hypothetical protein	8.02
Mtr.10984.1.S1_at	Hypothetical protein	8.00
Mtr.44194.1.S1_at	CPRD2 protein	8.00
Mtr.3183.1.S1_at	Hypothetical protein	7.99
Mtr.10808.1.S1_at	Prephenate dehydratase	7.98
Mtr.29714.1.S1_at	Hypothetical protein	7.96
Mtr.4083.1.S1_at	Hypothetical protein	7.95
Mtr.20588.1.S1_at	Hypothetical protein	7.95
Mtr.33658.1.S1_at	Hypothetical protein	7.92
Mtr.7741.1.S1_at	Peptide/amino acid transporter	7.91
Mtr.12226.1.S1_at	Hypothetical protein	7.91
Mtr.31572.1.S1_s_at	Inwardly rectifying potassium channel subunit	7.87
Msa.2990.1.S1_at	Hypothetical protein	7.87
Mtr.7638.1.S1_at	Endo-1,3-beta-glucanase	7.87
Mtr.38799.1.S1_s_at	Cytokinin oxidase-like protein	7.86
Mtr.39999.1.S1_at	Syringolide-induced protein B13-1-9	7.86
Mtr.12419.1.S1_s_at	50S ribosomal protein L15	7.85
Mtr.29535.1.S1_at	Hypothetical protein	7.85
Mtr.36136.1.S1_at	Hypothetical protein	7.84
Mtr.8617.1.S1_at	Syringolide-induced protein B15-3-5	7.84
Msa.2259.1.S1_at	Hypothetical protein	7.83
Mtr.32799.1.S1_at	GTP-binding regulatory protein extra-large	7.80
Mtr.46485.1.S1_at	Glutathione S-transferase GST 17	7.78
Mtr.46962.1.S1_at	Hypothetical protein	7.77
Mtr.42039.1.S1_at	Hypothetical protein	7.77
Mtr.21216.1.S1_at	Exo70 exocyst complex subunit	7.76
Mtr.27309.1.S1_at	Hypothetical protein	7.74
Mtr.6545.1.S1_at	Hypothetical protein	7.74
Mtr.45877.1.S1_at	RNA-binding region RNP-1	7.72
Mtr.41986.1.S1_at	Wall-associated kinase 4	7.71
Mtr.43656.1.S1_at	Cysteine protease	7.70
Mtr.5512.1.S1_at	Biotin synthase	7.69
Mtr.41342.1.S1_at	Serine/threonine-protein kinase	7.67
Mtr.19162.1.S1_at	AMP-dependent synthetase and ligase	7.65
Mtr.43887.1.S1_at	Hypothetical protein	7.65
Mtr.45285.1.S1_at	Hypothetical protein	7.62
Mtr.26378.1.S1_at	Hypothetical protein	7.62
Mtr.20804.1.S1_at	Protein kinase	7.61
Mtr.35122.1.S1_at	Hypothetical protein	7.61
Mtr.51571.1.S1_s_at	Amino acid/polyamine transporter II	7.60
Mtr.18295.1.S1_at	Zinc-containing alcohol dehydrogenase superfamily	7.60
Mtr.47077.1.S1_at	Hypothetical protein	7.60
Msa.1103.1.S1_at	Hypothetical protein	7.59
Mtr.49400.1.S1_at	Auxin responsive SAUR protein	7.58
Mtr.43041.1.S1_at	Hexokinase	7.58
Mtr.7638.1.S1_s_at	Endo-1,3-beta-glucanase	7.57

Mtr.24739.1.S1_at	Phosphoserine aminotransferase	7.55
Mtr.51630.1.S1_s_at	Hypothetical protein	7.54
Mtr.27861.1.S1_at	Hypothetical protein	7.54
Mtr.15772.1.S1_at	Glycoside hydrolase	7.52
Mtr.16854.1.S1_x_at	Protein kinase	7.51
Mtr.41506.1.S1_at	Hypothetical protein	7.50
Mtr.12277.1.S1_at	Class 10 PR protein	7.49
Mtr.26988.1.S1_s_at	Hypothetical protein	7.48
Mtr.38185.1.S1_at	Hypothetical protein	7.46
Msa.3062.1.S1_at	Hypothetical protein	7.45
Mtr.52198.1.S1_at	Bacterial regulatory factor	7.45
Mtr.26988.1.S1_at	Hypothetical protein	7.45
Mtr.43134.1.S1_at	Fructose-bisphosphate aldolase	7.44
Mtr.41386.1.S1_at	Hypothetical protein	7.44
Mtr.40724.1.S1_at	Hypothetical protein	7.44
Mtr.14303.1.S1_at	Hydrogenase large subunit	7.44
Mtr.27095.1.S1_at	Hypothetical protein	7.43
Mtr.261.1.S1_at	Hypothetical protein	7.43
Mtr.1797.1.S1_at	MYB-related protein	7.42
Mtr.9577.1.S1_at	Hypothetical protein	7.40
Mtr.49441.1.S1_at	2OG-Fe(II) oxygenase	7.40
Mtr.43353.1.S1_at	Hypothetical protein	7.37
Mtr.38735.1.S1_at	Hypothetical protein	7.36
Mtr.27097.1.S1_at	Hypothetical protein	7.35
Mtr.35833.1.S1_s_at	Hypothetical protein	7.35
Mtr.13643.1.S1_at	Hypothetical protein	7.33
Mtr.15997.1.S1_s_at	Peroxidase	7.33
Mtr.38092.1.S1_at	MAP kinase phosphatase	7.33
Mtr.45989.1.S1_s_at	Xylose isomerase	7.32
Mtr.5715.1.S1_at	Beta-glucan-elicitor receptor	7.32
Mtr.29666.1.S1_at	Hypothetical protein	7.29
Mtr.37676.1.S1_at	Hypothetical protein	7.27
Mtr.17556.1.S1_at	Hypothetical protein	7.24
Mtr.33372.1.S1_at	Hypothetical protein	7.23
Mtr.40048.1.S1_at	Acetylornithine aminotransferase	7.23
Mtr.39403.1.S1_at	Receptor-like serine/threonine kinase	7.23
Mtr.17610.1.S1_at	Hypothetical protein	7.23
Mtr.38022.1.S1_at	LEXYL1 protein	7.21
Mtr.38520.1.S1_s_at	Hypothetical protein	7.21
Mtr.29342.1.S1_at	Hypothetical protein	7.20
Mtr.39132.1.S1_at	Malate synthase	7.20
Mtr.10407.1.S1_at	Ferrochelatase II	7.19
Mtr.44996.1.S1_at	Acid phosphatase	7.19
Mtr.27753.1.S1_at	L-lactate dehydrogenase	7.18
Mtr.42373.1.S1_at	Peroxidase	7.18
Mtr.25889.1.S1_at	Protein kinase	7.18
Mtr.1587.1.S1_at	Calmodulin-related protein	7.17
Mtr.31649.1.S1_at	Hypothetical protein	7.17
Mtr.11087.1.S1_at	Hypothetical protein	7.17
Mtr.40765.1.S1_at	Hypothetical protein	7.16
Mtr.32735.1.S1_at	Hypothetical protein	7.15
Mtr.12958.1.S1_at	Hypothetical protein	7.14
Mtr.44130.1.S1_s_at	Hypothetical protein	7.13
Mtr.12290.1.S1_at	Proline dehydrogenase	7.13

Mtr.48476.1.S1_at	Hypothetical protein	7.11
Mtr.42598.1.S1_at	Hypothetical protein	7.10
Mtr.43119.1.S1_at	Chorismate synthase 1	7.09
Mtr.25887.1.S1_at	bHLH protein	7.08
Mtr.9756.1.S1_at	Rev interacting-like protein	7.05
Mtr.51425.1.S1_at	Serine/threonine protein kinase	7.04
Mtr.10672.1.S1_s_at	Polyphosphoinositide binding protein	7.02
Mtr.9421.1.S1_at	Indole-3-acetate beta-glucosyltransferase	7.02
Mtr.31952.1.S1_at	Hypothetical protein	7.01
Mtr.20668.1.S1_at	Hypothetical protein	7.01
Msa.1129.1.S1_at	Hypothetical protein	7.00
Mtr.40611.1.S1_at	Peroxidase	6.99
Mtr.38347.1.S1_at	Tripartite motif protein	6.96
Mtr.37560.1.S1_at	Pyruvate decarboxylase	6.96
Mtr.1874.1.S1_s_at	Phospholipid-transporting ATPase	6.96
Mtr.12264.1.S1_s_at	Cytochrome P450 monooxygenase	6.95
Mtr.5174.1.S1_at	Hypothetical protein	6.94
Mtr.33184.1.S1_at	Hypothetical protein	6.94
Mtr.13083.1.S1_at	Hypothetical protein	6.93
Mtr.5079.1.S1_at	Syringolide-induced protein 1-3-1B	6.92
Mtr.7063.1.S1_at	Alcohol dehydrogenase	6.90
Mtr.24464.1.S1_at	Receptor-like protein kinase	6.89
Mtr.42683.1.S1_at	Acyl-activating enzyme	6.89
Mtr.15470.1.S1_s_at	Amino acid/polyamine transporter II	6.87
Mtr.43391.1.S1_at	Hypothetical protein	6.87
Mtr.32702.1.S1_at	Hypothetical protein	6.86
Mtr.28496.1.S1_at	Protein kinase	6.85
Mtr.42418.1.S1_at	Phospholipase D	6.85
Mtr.28500.1.S1_at	Outer surface protein C	6.84
Mtr.8880.1.S1_at	B12D-like protein	6.84
Mtr.12423.1.S1_at	Heat shock transcription factor	6.84
Mtr.10681.1.S1_at	Universal stress protein USP1-like protein	6.84
Mtr.23311.1.S1_at	Glycosyl hydrolase family	6.84
Mtr.23026.1.S1_at	Hypothetical protein	6.82
Mtr.28717.1.S1_at	Hypothetical protein	6.81
Msa.3099.1.S1_at	Hypothetical protein	6.81
Mtr.38568.1.S1_at	Kelch repeat-containing F-box	6.79
Mtr.42626.1.S1_at	Hypothetical protein	6.79
Mtr.19224.1.S1_at	bZIP transcription factor	6.78
Mtr.45266.1.S1_at	Phospholipase D	6.77
Msa.1202.1.S1_at	Hypothetical protein	6.77
Mtr.16850.1.S1_at	Curculin-like (mannose-binding) lectin	6.76
Mtr.46462.1.S1_at	AMP-dependent synthetase and ligase family	6.74
Mtr.6934.1.S1_at	Glutaredoxin-related-like protein	6.73
Msa.3172.1.S1_at	Hypothetical protein	6.72
Mtr.49405.1.S1_s_at	Disease resistance protein	6.72
Mtr.37493.1.S1_at	Phi-1 protein	6.71
Mtr.51988.1.S1_at	Pyridine nucleotide-disulphide oxidoreductase	6.70
Mtr.28526.1.S1_at	Hypothetical protein	6.70
Mtr.44386.1.S1_at	Hypothetical protein	6.69
Msa.1009.1.S1_at	Hypothetical protein	6.68
Mtr.51717.1.S1_at	Hypothetical protein	6.68
Mtr.17920.1.S1_s_at	Hypothetical protein	6.68
Mtr.17409.1.S1_s_at	Amino acid/polyamine transporter II	6.67

Mtr.48996.1.S1_at	Heat shock protein DnaJ	6.67
Mtr.37873.1.S1_at	Hypothetical protein	6.66
Mtr.40256.1.S1_at	Hypothetical protein	6.66
Mtr.7637.1.S1_at	Prostatic spermine-binding protein precursor (SBP)	6.66
Mtr.43184.1.S1_at	Hypothetical protein	6.65
Mtr.40073.1.S1_at	Hypothetical protein	6.65
Mtr.46961.1.S1_at	Hypothetical protein	6.65
Mtr.10636.1.S1_at	MYB protein	6.65
Mtr.3151.1.S1_s_at	Hypothetical protein	6.65
Mtr.17506.1.S1_at	Phospholipid-translocating P-type ATPase	6.64
Mtr.10928.1.S1_s_at	Avr9/Cf-9 rapidly elicited protein	6.64
Mtr.43605.1.S1_at	Glycosyl transferase	6.63
Mtr.40680.1.S1_at	Transcription factor MYB	6.63
Mtr.41783.1.S1_at	Hypothetical protein	6.62
Mtr.34448.1.S1_at	UDP-glucose 4-epimerase	6.62
Msa.1124.1.S1_at	Hypothetical protein	6.61
Mtr.8669.1.S1_at	Hypothetical protein	6.61
Mtr.44874.1.S1_at	Hypothetical protein	6.61
Mtr.40278.1.S1_at	Glutathione S-transferase	6.61
Mtr.42299.1.S1_at	Hypothetical protein	6.60
Mtr.11544.1.S1_at	Receptor-like kinase	6.59
Mtr.7943.1.S1_at	Hypothetical protein	6.58
Mtr.4906.1.S1_s_at	1-aminocyclopropane-1-carboxylate oxidase (ACC oxidase)	6.57
Mtr.9897.1.S1_s_at	Glycerol kinase	6.57
Mtr.43924.1.S1_at	Glutaredoxin-related-like protein	6.57
Mtr.34980.1.S1_s_at	Hypothetical protein	6.55
Mtr.33595.1.S1_s_at	Root-specific metal transporter	6.54
Mtr.6892.1.S1_at	S glycoprotein	6.54
Mtr.5692.1.S1_at	Indole-3-acetate beta-glucosyltransferase-like protein	6.53
Mtr.19888.1.S1_at	Glycosyl hydrolases family	6.51
Mtr.50994.1.S1_at	Zn-finger, RING	6.50
Mtr.46208.1.S1_at	Hypothetical protein	6.48
Mtr.10681.1.S1_s_at	Universal stress protein USP1-like protein	6.47
Msa.1569.1.S1_at	Hypothetical protein	6.46
Mtr.37906.1.S1_at	4,5-DOPA dioxygenase extradiol-like protein	6.45
Mtr.44872.1.S1_at	Cytochrome P450 monooxygenase	6.45
Mtr.50290.1.S1_s_at	Hypothetical protein	6.44
Mtr.42673.1.S1_at	Receptor-like serine/threonine kinase	6.44
Mtr.32374.1.S1_at	Serine carboxypeptidase	6.43
Mtr.11516.1.S1_at	Phosphoserine aminotransferase	6.42
Mtr.10063.1.S1_at	Cycloidea protein	6.42
Mtr.16854.1.S1_at	Protein kinase	6.41
Mtr.10165.1.S1_at	Ethylene receptor	6.41
Mtr.41182.1.S1_at	GTP-binding protein	6.40
Mtr.11783.1.S1_at	Hypothetical protein	6.39
Mtr.38735.1.S1_s_at	Hypothetical protein	6.39
Mtr.39468.1.S1_at	Hypothetical protein	6.39
Mtr.5055.1.S1_at	Hypothetical protein	6.39
Mtr.28525.1.S1_at	Hypothetical protein	6.38
Msa.1670.1.S1_at	Hypothetical protein	6.38
Mtr.48899.1.S1_at	Ferritin	6.37
Mtr.22589.1.S1_at	Hypothetical protein	6.36
Mtr.43900.1.S1_at	Hypothetical protein	6.35
Mtr.33265.1.S1_at	Hypothetical protein	6.35

Mtr.37224.1.S1_at	Hydroxyproline-rich glycoprotein	6.35
Mtr.38256.1.S1_s_at	Flavonol synthase	6.35
Mtr.12226.1.S1_x_at	Hypothetical protein	6.35
Mtr.32195.1.S1_at	Urea active transporter	6.34
Msa.686.1.S1_at	Hypothetical protein	6.34
Mtr.39267.1.S1_at	1-aminocyclopropane-1-carboxylate synthase	6.34
Mtr.38662.1.S1_at	Hypothetical protein	6.33
Mtr.38932.1.S1_at	Malate synthase	6.33
Mtr.24465.1.S1_at	Putative receptor-like protein kinase	6.33
Mtr.35704.1.S1_at	Hypothetical protein	6.33
Mtr.29300.1.S1_x_at	Hypothetical protein	6.31
Mtr.11587.1.S1_at	Hypothetical protein	6.30
Mtr.15028.1.S1_at	Transketolase	6.30
Mtr.41419.1.S1_at	Ring domain containing protein	6.30
Mtr.10090.1.S1_at	Homo-phytochelatin synthase	6.29
Mtr.33446.1.S1_at	Sucrose transporter	6.28
Mtr.42915.1.S1_at	Hypothetical protein	6.27
Mtr.35009.1.S1_at	Hypothetical protein	6.26
Mtr.41255.1.S1_at	Amino acid permease	6.25
Mtr.41855.1.S1_at	Hypothetical protein	6.25
Mtr.49771.1.S1_at	Hypothetical protein	6.25
Mtr.19206.1.S1_at	Ras GTPase	6.25
Mtr.1874.1.S1_at	Potential phospholipid-transporting ATPase	6.24
Mtr.9079.1.S1_at	Hypothetical protein	6.24
Mtr.24434.1.S1_at	Hypothetical protein	6.24
Mtr.42185.1.S1_at	Hypothetical protein	6.24
Mtr.12290.1.S1_s_at	Proline dehydrogenase	6.23
Mtr.22675.1.S1_at	Hypothetical protein	6.20
Mtr.10956.1.S1_at	Hypothetical protein	6.20
Mtr.32397.1.S1_s_at	Lipid phosphate phosphatase	6.19
Mtr.42770.1.S1_at	Photosystem II 23 kDa polypeptide	6.19
Mtr.25575.1.S1_at	Hypothetical protein	6.19
Mtr.44823.1.S1_at	Hypothetical protein	6.19
Mtr.37646.1.S1_s_at	Hypothetical protein	6.18
Mtr.6875.1.S1_at	Hypothetical protein	6.17
Mtr.29374.1.S1_at	Hypothetical protein	6.17
Mtr.43023.1.S1_at	Hypothetical protein	6.17
Mtr.27726.1.S1_at	Rod shape-determining protein	6.17
Mtr.8653.1.S1_at	Peroxidase	6.16
Mtr.14785.1.S1_at	Hypothetical protein	6.16
Msa.2848.1.S1_at	Hypothetical protein	6.16
Mtr.33562.1.S1_at	Hypothetical protein	6.15
Mtr.684.1.S1_at	Hypothetical protein	6.14
Mtr.45910.1.S1_at	Hypothetical protein	6.13
Mtr.49234.1.S1_at	Protein kinase	6.11
Msa.1709.1.S1_at	Hypothetical protein	6.11
Mtr.13044.1.S1_at	WRKY transcription factor	6.11
Mtr.43707.1.S1_at	Hypothetical protein	6.11
Mtr.43706.1.S1_at	Hypothetical protein	6.10
Mtr.3345.1.S1_at	Hypothetical protein	6.10
Mtr.51664.1.S1_s_at	Hypothetical protein	6.08
Mtr.10308.1.S1_at	Hydroxyproline-rich glycoprotein	6.08
Mtr.42692.1.S1_at	Glutaredoxin-like protein	6.07
Mtr.38959.1.S1_s_at	GTP-binding protein	6.07

Mtr.12772.1.S1_at	Mlo protein homolog	6.07
Mtr.13150.1.S1_at	Hypothetical protein	6.07
Mtr.11986.1.S1_at	WD40-repeat protein	6.06
Mtr.50585.1.S1_at	Proteinase inhibitor I3	6.06
Mtr.38799.1.S1_at	Cytokinin oxidase-like protein	6.06
Mtr.51916.1.S1_at	Hypothetical protein	6.06
Mtr.25168.1.S1_at	Hypothetical protein	6.06
Mtr.15470.1.S1_at	Amino acid/polyamine transporter II	6.05
Mtr.43416.1.S1_at	Zinc-finger protein	6.05
Mtr.41223.1.S1_at	ATP-dependent phosphoenolpyruvate carboxykinase	6.04
Mtr.37277.1.S1_at	Calcium-dependent protein kinase	6.03
Msa.2329.1.S1_at	Hypothetical protein	6.03
Mtr.10049.1.S1_at	Biotin synthase	6.03
Mtr.39460.1.S1_at	Hypothetical protein	6.02
Mtr.6178.1.S1_at	Alanine racemase	6.02
Mtr.9478.1.S1_at	Laccase-like protein	6.00
Mtr.42353.1.S1_at	NADH dehydrogenase subunit 3	6.00
Mtr.24465.1.S1_x_at	Putative receptor-like protein kinase	5.98
Mtr.16728.1.S1_at	Hypothetical protein	5.98
Mtr.11612.1.S1_at	Hypothetical protein	5.98
Mtr.41599.1.S1_s_at	SNARE-interacting protein KEULE	5.97
Mtr.10655.1.S1_at	Hypothetical protein	5.96
Msa.1241.1.S1_at	Hypothetical protein	5.95
Msa.3111.1.S1_x_at	Hypothetical protein	5.95
Mtr.41427.1.S1_at	Pyruvate, phosphate dikinase	5.95
Mtr.50492.1.S1_s_at	Ribosomal protein L3	5.95
Msa.2831.1.S1_at	Hypothetical protein	5.93
Mtr.18747.1.S1_at	Hypothetical protein	5.93
Mtr.43272.1.S1_at	Hypothetical protein	5.93
Mtr.11112.1.S1_at	Hypothetical protein	5.93
Mtr.32666.1.S1_at	Hypothetical protein	5.92
Mtr.10253.1.S1_at	Cellulose synthase	5.92
Mtr.11385.1.S1_at	Hypothetical protein	5.92
Mtr.5032.1.S1_at	KIC-related protein	5.92
Mtr.40075.1.S1_at	Pprg2 protein	5.91
Mtr.44404.1.S1_s_at	Hypothetical protein	5.91
Mtr.33536.1.S1_at	Sugar transporter-like protein	5.90
Mtr.47936.1.S1_at	Hypothetical protein	5.90
Mtr.912.1.S1_s_at	ATP citrate lyase a-subunit	5.89
Mtr.9214.1.S1_at	Hypothetical protein	5.89
Mtr.48875.1.S1_s_at	Hypothetical protein	5.89
Mtr.21501.1.S1_at	Zn-finger, RING	5.89
Mtr.35960.1.S1_at	1, 3-beta-glucanase-like protein	5.89
Mtr.15172.1.S1_at	Leucine-rich repeat receptor-like protein kinase 1	5.89
Mtr.11788.1.S1_at	Hypothetical protein	5.87
Mtr.13161.1.S1_at	Hypothetical protein	5.87
Mtr.35172.1.S1_at	Hypothetical protein	5.87
Msa.1270.1.S1_at	Hypothetical protein	5.86
Mtr.51256.1.S1_at	Transferase	5.86
Mtr.6526.1.S1_at	Hypothetical protein	5.86
Mtr.26364.1.S1_at	Hypothetical protein	5.86
Mtr.52124.1.S1_s_at	Phosphate-induced protein	5.86
Mtr.47542.1.S1_at	Hypothetical protein	5.86
Mtr.40239.1.S1_at	Phosphoenolpyruvate carboxykinase	5.85

Mtr.37168.1.S1_at	Hypothetical protein	5.85
Mtr.37374.1.S1_at	Patatin-like protein	5.85
Mtr.35662.1.S1_at	Hypothetical protein	5.85
Mtr.12240.1.S1_at	Envelope glycoprotein	5.85
Mtr.37283.1.S1_s_at	Pathogen-inducible alpha-dioxygenase	5.85
Mtr.26459.1.S1_at	Avr9/Cf-9 rapidly elicited protein	5.84
Mtr.40847.1.S1_at	Hypothetical protein	5.84
Mtr.45270.1.S1_at	Hypothetical protein	5.84
Mtr.12783.1.S1_at	Heat shock protein	5.84
Mtr.37678.1.S1_at	Oxoglutarate transaminase I	5.83
Mtr.41962.1.S1_at	Ketol-acid reductoisomerase	5.83
MsaAffx.3537.1.S1_s_at	Hypothetical protein	5.83
Mtr.28547.1.S1_s_at	Glutathione S-transferase GST	5.83
Mtr.13060.1.S1_at	Hypothetical protein	5.82
Mtr.25525.1.S1_at	Hypothetical protein	5.80
Mtr.36359.1.S1_s_at	Hypothetical protein	5.79
Mtr.27828.1.S1_at	Phospholipase D	5.78
Mtr.37831.1.S1_at	Hypothetical protein	5.77
Mtr.11728.1.S1_at	Hypothetical protein	5.77
Mtr.33441.1.S1_s_at	Hypothetical protein	5.77
Mtr.9063.1.S1_at	Glucose/sorbosone dehydrogenases-like protein	5.76
Mtr.1018.1.S1_s_at	GDP-mannose transporter	5.75
Mtr.44925.1.S1_at	Hypothetical protein	5.73
Mtr.52317.1.S1_at	Hypothetical protein	5.73
Mtr.45563.1.S1_at	PDR9 ABC transporter	5.73
Mtr.33142.1.S1_at	Hypothetical protein	5.73
Mtr.42571.1.S1_at	MutT domain protein	5.71
Mtr.35520.1.S1_at	Hypothetical protein	5.71
Mtr.39287.1.S1_at	SGP1 monomeric G-protein	5.71
Mtr.25966.1.S1_at	Hypothetical protein	5.70
Mtr.48874.1.S1_at	Hypothetical protein	5.69
Mtr.11072.1.S1_at	Hypothetical protein	5.69
Mtr.1401.1.S1_at	Hypothetical protein	5.68
Mtr.13407.1.S1_at	Hypothetical protein	5.68
Mtr.40911.1.S1_s_at	Cationic peroxidase 2	5.68
Mtr.35045.1.S1_at	Rab escort protein	5.68
Mtr.47908.1.S1_at	Hypothetical protein	5.67
Mtr.13237.1.S1_at	Hypothetical protein	5.66
Mtr.7249.1.S1_at	N-hydroxycinnamoyl/benzoyltransferase-like protein	5.66
Mtr.50912.1.S1_s_at	Hypothetical protein	5.65
Mtr.985.1.S1_at	Hypothetical protein	5.65
Mtr.8425.1.S1_at	Hypothetical protein	5.65
Mtr.28550.1.S1_at	Hypothetical protein	5.64
Mtr.12589.1.S1_at	Acetyl-CoA carboxylase	5.63
Mtr.40933.1.S1_at	TIR-similar-domain-containing protein	5.63
Mtr.34901.1.S1_s_at	PDR-like ABC transporter	5.63
Mtr.40880.1.S1_at	Electron transfer flavoprotein beta-subunit-like	5.62
Mtr.9429.1.S1_at	Transcription factor RAU1	5.62
Mtr.42984.1.S1_at	Proline dehydrogenase	5.61
Mtr.38375.1.S1_at	Hypothetical protein	5.60
Mtr.23085.1.S1_s_at	Hypothetical protein	5.60
Msa.1414.1.S1_at	Hypothetical protein	5.60
Mtr.12694.1.S1_at	NAC domain protein NAC1	5.59
Mtr.1800.1.S1_at	Cytochrome b	5.59

Mtr.17829.1.S1_at	UDP-glucose 4-epimerase	5.58
Mtr.2486.1.S1_at	Hypothetical protein	5.58
Mtr.12226.1.S1_s_at	Hypothetical protein	5.58
Mtr.31075.1.S1_at	Receptor protein kinase	5.58
Mtr.12034.1.S1_at	ADP/ATP translocase-like protein	5.58
Mtr.43845.1.S1_at	Hypothetical protein	5.58
Mtr.43343.1.S1_at	Hypothetical protein	5.57
Mtr.6926.1.S1_at	Hypothetical protein	5.56
Mtr.41494.1.S1_at	Hypothetical protein	5.56
Mtr.40203.1.S1_at	Aldehyde dehydrogenase (NAD+)	5.56
Mtr.1526.1.S1_a_at	Hypothetical protein	5.54
Mtr.27812.1.S1_at	Hypothetical protein	5.54
Mtr.6918.1.S1_at	Phospholipase PLDb1	5.54
Mtr.40315.1.S1_at	Al-induced protein	5.54
Mtr.4958.1.S1_at	Hypothetical protein	5.54
Mtr.12540.1.S1_s_at	Receptor kinase	5.54
Mtr.48787.1.S1_s_at	WD-40 repeat protein	5.53
Mtr.43583.1.S1_at	Hypothetical protein	5.53
Mtr.19235.1.S1_at	Hypothetical protein	5.53
Mtr.24063.1.S1_at	Hypothetical protein	5.53
Mtr.33188.1.S1_at	Hypothetical protein	5.52
Mtr.32188.1.S1_x_at	Chalcone synthase 9	5.52
Mtr.31327.1.S1_s_at	Protein kinase	5.51
Mtr.12497.1.S1_at	Protein kinase	5.50
Mtr.12412.1.S1_at	Glutamate dehydrogenase 1 (GDH 1)	5.49
Mtr.50046.1.S1_at	Hypothetical protein	5.49
Mtr.38086.1.S1_at	Alpha-N-acetylglucosaminidase	5.49
Mtr.25306.1.S1_at	Prolyl 4-hydroxylase, alpha subunit	5.48
Mtr.8935.1.S1_at	Alternative oxidase 3	5.48
Mtr.41983.1.S1_at	Glucosyltransferase-7	5.48
Mtr.33482.1.S1_s_at	Carbonic anhydrase 2	5.48
Mtr.50205.1.S1_at	Hypothetical protein	5.47
Mtr.38064.1.S1_at	Hypothetical protein	5.47
Mtr.40124.1.S1_at	Peroxidase1B	5.47
Msa.2372.1.S1_at	Hypothetical protein	5.46
Mtr.10619.1.S1_at	Hypothetical protein	5.46
Msa.1774.1.S1_at	Hypothetical protein	5.46
Mtr.9101.1.S1_at	PDR-like ABC transporter	5.46
Msa.3040.1.S1_at	Hypothetical protein	5.45
Mtr.40703.1.S1_at	Hypothetical protein	5.45
Mtr.9613.1.S1_at	TIR-similar-domain-containing protein	5.44
Mtr.11134.1.S1_at	Ornithine aminotransferase	5.44
Mtr.43706.1.S1_s_at	Hypothetical protein	5.44
Mtr.42223.1.S1_at	Hypothetical protein	5.44
Mtr.4967.1.S1_at	GTPase activating protein	5.43
Msa.1639.1.S1_at	Hypothetical protein	5.42
Mtr.51296.1.S1_at	Hypothetical protein	5.42
Mtr.45843.1.S1_at	Hypothetical protein	5.40
Mtr.10408.1.S1_at	Ferrochelatase I	5.39
Msa.2663.1.S1_at	Hypothetical protein	5.39
Mtr.31177.1.S1_at	Ubiquitin-protein ligase	5.38
Msa.3054.1.S1_at	Hypothetical protein	5.38
Mtr.10616.1.S1_at	Hypothetical protein	5.38
Mtr.34926.1.S1_s_at	Hypothetical protein	5.37

Mtr.24888.1.S1_at	Hypothetical protein	5.37
Mtr.11278.1.S1_at	Hypothetical protein	5.37
Mtr.39144.1.S1_at	WD40-repeat protein	5.37
Mtr.32690.1.S1_at	Hypothetical protein	5.36
Mtr.44843.1.S1_at	Hypothetical protein	5.35
Mtr.43170.1.S1_s_at	Glutamate dehydrogenase 1 (GDH 1)	5.35
Mtr.24715.1.S1_at	Glutathione S-transferase GST 14	5.34
Mtr.39919.1.S1_at	Protein kinase	5.34
Mtr.35824.1.S1_at	GATA-1 zinc finger protein	5.33
Mtr.25611.1.S1_at	4-coumarate:CoA ligase	5.33
Mtr.38510.1.S1_at	Hypothetical protein	5.33
Mtr.40682.1.S1_at	Proline/glycine betaine transporter	5.33
Mtr.4702.1.S1_at	Fiber protein Fb1	5.33
Mtr.40789.1.S1_at	DNA-binding protein	5.33
Mtr.44516.1.S1_at	Type IIB calcium ATPase	5.33
Mtr.7608.1.S1_at	50S ribosomal protein L33	5.31
Mtr.33221.1.S1_s_at	acyl-activating enzyme 13	5.31
Mtr.32008.1.S1_at	Hypothetical protein	5.31
Mtr.37347.1.S1_s_at	Hypothetical protein	5.30
Mtr.43291.1.S1_at	Fiber protein Fb1	5.30
Mtr.35086.1.S1_at	Hypothetical protein	5.30
Mtr.44122.1.S1_at	Hypothetical protein	5.30
Mtr.47774.1.S1_at	Hypothetical protein	5.28
Mtr.31335.1.S1_s_at	Alcohol dehydrogenase-like protein	5.28
Mtr.791.1.S1_at	Acyl-activating enzyme	5.28
Mtr.296.1.S1_at	Hypothetical protein	5.28
Mtr.43917.1.S1_at	Hypothetical protein	5.28
Mtr.2124.1.S1_s_at	Hypothetical protein	5.27
Mtr.25841.1.S1_at	Plasma intrinsic protein	5.26
Mtr.1214.1.S1_at	Hypothetical protein	5.26
Mtr.8548.1.S1_at	Proline-rich cell wall protein	5.25
Mtr.5044.1.S1_s_at	Hypothetical protein	5.25
Mtr.10691.1.S1_at	ATP citrate lyase a-subunit	5.25
Mtr.19723.1.S1_at	Serine/threonine protein kinase	5.25
Mtr.11357.1.S1_at	SNARE-interacting protein KEULE	5.24
Mtr.27551.1.S1_at	LOB domain protein 38	5.24
Mtr.42007.1.S1_at	Hypothetical protein	5.24
Mtr.42072.1.S1_at	Nodulin	5.24
Mtr.6811.1.S1_s_at	Glutathione S-transferase GST 17	5.23
Mtr.25059.1.S1_at	WD-40 repeat protein	5.22
Mtr.43266.1.S1_at	Hypothetical protein	5.22
Mtr.13394.1.S1_at	Hypothetical protein	5.21
Mtr.82.1.S1_at	STS14 protein	5.20
Mtr.31406.1.S1_x_at	Isoflavonoid glucosyltransferase	5.20
Mtr.13630.1.S1_at	Hypothetical protein	5.19
Mtr.35775.1.S1_at	Hypothetical protein	5.19
Mtr.37393.1.S1_at	Hypothetical protein	5.19
Mtr.37412.1.S1_s_at	Chalcone-flavonone isomerase	5.19
Mtr.51042.1.S1_at	Hypothetical protein	5.18
Mtr.50280.1.S1_at	Protein kinase	5.18
Mtr.19727.1.S1_at	Hypothetical protein	5.18
Mtr.22601.1.S1_at	Endoxyloglucan transferase	5.17
Mtr.21035.1.S1_at	Sugar transporter	5.17
Mtr.18369.1.S1_at	Glutathione S-transferase	5.17

Mtr.20215.1.S1_s_at	Hypothetical protein	5.16
Mtr.13949.1.S1_at	PLU-1 protein	5.16
Mtr.30853.1.S1_at	Sugar transporter	5.16
Mtr.15228.1.S1_at	Hypothetical protein	5.16
Mtr.20315.1.S1_at	Adenine phosphoribosyltransferase-like protein	5.15
Mtr.15448.1.S1_at	Gibberellin regulated protein	5.14
Mtr.2282.1.S1_at	Hypothetical protein	5.13
Mtr.44257.1.S1_s_at	Hypothetical protein	5.13
Mtr.9897.1.S1_at	Glycerol kinase	5.12
Mtr.39894.1.S1_at	Dermal papilla derived protein	5.12
Mtr.11383.1.S1_at	Hypothetical protein	5.12
Mtr.4702.1.S1_s_at	Fiber protein Fb1	5.12
Mtr.15523.1.S1_at	U box protein	5.12
Mtr.38274.1.S1_at	Steroid 22-alpha-hydroxylase	5.11
Mtr.12525.1.S1_at	Chitinase	5.10
Msa.996.1.S1_at	Hypothetical protein	5.10
Mtr.51562.1.S1_at	No apical meristem (NAM) protein	5.09
Mtr.33428.1.S1_at	Hypothetical protein	5.09
Mtr.37284.1.S1_at	Topoisomerase-like protein	5.09
Mtr.10407.1.S1_s_at	Ferrochelatase II	5.09
Mtr.18646.1.S1_at	Hypothetical protein	5.08
Mtr.37330.1.S1_at	Transcription factor	5.08
Mtr.41526.1.S1_at	Lipoamide dehydrogenase	5.08
Mtr.12175.1.S1_at	Hypothetical protein	5.08
Mtr.42389.1.S1_at	Hypothetical protein	5.07
Mtr.14250.1.S1_at	Arf GTPase activating protein	5.07
Mtr.11251.1.S1_at	Protein integral membrane protein	5.06
Mtr.42067.1.S1_s_at	Hypothetical protein	5.06
Mtr.34806.1.S1_s_at	MLO-like protein 2	5.06
Mtr.38811.1.S1_s_at	Nucleosome assembly protein 1	5.06
Mtr.8884.1.S1_at	Pathogenesis-related protein 4A	5.06
Mtr.37410.1.S1_at	Hypothetical protein	5.05
Mtr.5270.1.S1_at	Hypothetical protein	5.05
Mtr.43241.1.S1_at	Transcription factor WRKY32	5.04
Mtr.34775.1.S1_at	Hypothetical protein	5.04
Mtr.9913.1.S1_at	Hypothetical protein	5.04
Mtr.24211.1.S1_at	Hypothetical protein	5.04
Mtr.43364.1.S1_at	Hypothetical protein	5.03
Mtr.39176.1.S1_at	Hypothetical protein	5.02
Mtr.31291.1.S1_at	Geranyl diphosphate synthase large subunit	5.01
Mtr.43799.1.S1_at	Hypothetical protein	5.00
Mtr.43853.1.S1_at	Hypothetical protein	5.00
Mtr.44780.1.S1_at	Hypothetical protein	5.00
Mtr.13094.1.S1_at	GTP-binding protein	5.00
Msa.884.1.S1_at	Hypothetical protein	5.00
Mtr.32104.1.S1_s_at	Respiratory burst oxidase homolog	4.99
Mtr.49012.1.S1_at	Zn-finger	4.99
Mtr.5195.1.S1_at	Hypothetical protein	4.99
Mtr.9552.1.S1_at	Protein kinase	4.98
Mtr.48688.1.S1_at	DNA-binding WRKY	4.98
Mtr.39742.1.S1_at	Hypothetical protein	4.97
Mtr.47441.1.S1_at	Hypothetical protein	4.96
Mtr.38205.1.S1_at	Mannose transporter	4.96
Mtr.42289.1.S1_at	Hypothetical protein	4.96

Mtr.36847.1.S1_at	Hypothetical protein	4.96
Mtr.43236.1.S1_at	UDP-glucosyltransferase	4.96
Mtr.27895.1.S1_at	Ser/Thr protein phosphatase 2C	4.95
Mtr.12626.1.S1_at	Snakin-1	4.95
Msa.582.1.S1_at	Hypothetical protein	4.94
Mtr.1549.1.S1_at	Hypothetical protein	4.94
Mtr.12688.1.S1_at	Hypothetical protein	4.93
Mtr.34217.1.S1_at	Ss-galactosidase	4.93
Mtr.40685.1.S1_at	Hypothetical protein	4.93
Mtr.2158.1.S1_at	Hypothetical protein	4.93
Mtr.51563.1.S1_at	Threonine synthase	4.92
Mtr.29652.1.S1_at	Hypothetical protein	4.92
Msa.2661.1.S1_at	Hypothetical protein	4.92
Mtr.27222.1.S1_at	Hypothetical protein	4.92
Mtr.9141.1.S1_at	Hypothetical protein	4.91
Mtr.31614.1.S1_at	WD-repeat protein	4.91
Mtr.37965.1.S1_at	Hypothetical protein	4.91
Mtr.45293.1.S1_s_at	Hypothetical protein	4.91
Mtr.8705.1.S1_at	Hypothetical protein	4.90
Msa.1436.1.S1_at	Hypothetical protein	4.90
Msa.2215.1.S1_at	Hypothetical protein	4.90
Mtr.12760.1.S1_at	Hypothetical protein	4.90
Mtr.10976.1.S1_at	WRKY transcription factor	4.89
Mtr.26606.1.S1_at	Hypothetical protein	4.89
Mtr.27313.1.S1_at	Receptor kinase-like protein	4.89
Mtr.13645.1.S1_at	Hypothetical protein	4.89
Mtr.37412.1.S1_at	Chalcone-flavonone isomerase	4.87
Mtr.13139.1.S1_at	Carbonate dehydratase-like protein	4.86
Mtr.9546.1.S1_at	Hypothetical protein	4.86
Mtr.35144.1.S1_s_at	Acyl-activating enzyme	4.86
Mtr.10018.1.S1_at	Hypothetical protein	4.86
Mtr.12946.1.S1_at	Zinc finger (C3HC4-type RING finger) family protein	4.85
Mtr.15895.1.S1_s_at	Hypothetical protein	4.85
Mtr.39774.1.S1_at	Ttg1-like protein	4.85
Mtr.42470.1.S1_x_at	Alpha galactosidase	4.85
Mtr.24755.1.S1_at	Hypothetical protein	4.85
Mtr.47143.1.S1_s_at	Brassinosteroid-regulated protein BRU1	4.85
Mtr.43378.1.S1_at	Beta-1,3 glucanase precursor	4.85
Mtr.17517.1.S1_at	Hypothetical protein	4.84
Mtr.30227.1.S1_at	Hypothetical protein	4.83
Mtr.12220.1.S1_at	Phi-1 protein	4.82
Mtr.30167.1.S1_at	Hypothetical protein	4.82
Mtr.16873.1.S1_s_at	O-methyltransferase	4.81
Msa.3096.1.S1_at	Hypothetical protein	4.81
Mtr.1266.1.S1_at	Protein kinase	4.81
Mtr.27351.1.S1_at	Protein kinase	4.81
Mtr.49053.1.S1_at	Hypothetical protein	4.81
Mtr.37779.1.S1_at	Formate dehydrogenase	4.81
Mtr.10701.1.S1_at	Axi 1 protein-like protein	4.80
Mtr.40651.1.S1_at	Hypothetical protein	4.80
Mtr.10629.1.S1_at	Caffeoyl-CoA O-methyltransferase	4.80
Mtr.6545.1.S1_x_at	Hypothetical protein	4.80
Mtr.930.1.S1_at	Hypothetical protein	4.80
Mtr.11140.1.S1_at	Protein kinase APK1B	4.79

Mtr.246.1.S1_at	DnaJ-like protein	4.79
Mtr.44747.1.S1_at	Secreted glycoprotein	4.79
Mtr.8789.1.S1_s_at	Formate dehydrogenase	4.79
Mtr.42470.1.S1_at	Alpha galactosidase	4.78
Mtr.40284.1.S1_at	Hypothetical protein	4.78
Mtr.44123.1.S1_at	Lipase class 3-like	4.78
Mtr.30474.1.S1_s_at	Hypothetical protein	4.78
Mtr.18117.1.S1_at	Hypothetical protein	4.78
Mtr.31575.1.S1_at	Phosphoribosylanthranilate transferase	4.78
Mtr.10974.1.S1_at	Hypothetical protein	4.78
Mtr.4918.1.S1_at	S-receptor kinase	4.78
Msa.2673.1.S1_at	Hypothetical protein	4.77
Mtr.7708.1.S1_s_at	Hypothetical protein	4.77
Mtr.6696.1.S1_at	Hypothetical protein	4.77
Mtr.42052.1.S1_at	F-box family protein	4.77
Mtr.27992.1.S1_at	Hypothetical protein	4.76
Mtr.5337.1.S1_at	Hypothetical protein	4.76
Msa.1875.1.S1_at	Hypothetical protein	4.76
Mtr.35604.1.S1_at	Lipase family protein	4.74
Mtr.6009.1.S1_at	Hypothetical protein	4.73
Mtr.9913.1.S1_s_at	Hypothetical protein	4.73
Mtr.38424.1.S1_at	Lectin-like receptor kinase	4.72
Mtr.9668.1.S1_at	Hypothetical protein	4.72
Mtr.8990.1.S1_at	Hypothetical protein	4.72
Mtr.6378.1.S1_at	1-aminocyclopropanecarboxylic acid oxidase	4.71
Mtr.2720.1.S1_at	Gyrase A	4.71
Mtr.5765.1.S1_at	Receptor-like protein kinase	4.71
Mtr.9938.1.S1_at	Dihydrodipicolinate synthase	4.71
Mtr.12312.1.S1_at	ATP citrate lyase b-subunit	4.71
Mtr.30446.1.S1_at	Cystinosin	4.70
Mtr.31877.1.S1_at	P-glycoprotein-like	4.70
Mtr.1851.1.S1_s_at	Secreted glycoprotein	4.69
Mtr.37202.1.S1_at	Cyclic nucleotide gated channel	4.68
Mtr.33360.1.S1_at	Hypothetical protein	4.68
Mtr.29044.1.S1_at	Hypothetical protein	4.68
Mtr.2889.1.S1_at	Hypothetical protein	4.68
Msa.1940.1.S1_at	Hypothetical protein	4.68
Mtr.2627.1.S1_at	Hypothetical protein	4.68
Mtr.44816.1.S1_at	Hypothetical protein	4.67
Mtr.41503.1.S1_at	Hypothetical protein	4.67
Mtr.20821.1.S1_at	Zinc finger protein, CONSTANS type	4.67
Mtr.5950.1.S1_s_at	Zinc finger protein	4.66
Mtr.37524.1.S1_at	Hypothetical protein	4.66
Mtr.44393.1.S1_at	Transformer-SR ribonucleoprotein	4.65
Mtr.11388.1.S1_at	Transcription factor	4.65
Mtr.41307.1.S1_at	Hypothetical protein	4.65
Mtr.43258.1.S1_s_at	Hypothetical protein	4.65
Mtr.50859.1.S1_s_at	Hypothetical protein	4.64
Mtr.428.1.S1_at	Leucine rich repeat protein family	4.64
Mtr.8726.1.S1_at	Hypothetical protein	4.64
Mtr.13347.1.S1_at	Lipoxygenase	4.64
Mtr.8621.1.S1_s_at	Hypothetical protein	4.64
Mtr.4342.1.S1_at	Hypothetical protein	4.63
Mtr.21000.1.S1_at	Universal stress protein	4.63

Mtr.33203.1.S1_at	Hypothetical protein	4.63
Mtr.48130.1.S1_at	Hypothetical protein	4.63
Mtr.41380.1.S1_at	Hypothetical protein	4.62
Mtr.43294.1.S1_at	Non-specific lipid transfer-like protein	4.62
Mtr.49745.1.S1_s_at	Tyrosine specific protein phosphatase	4.61
Mtr.20378.1.S1_at	Sugar transporter	4.61
Mtr.9074.1.S1_at	Hypothetical protein	4.61
Mtr.35088.1.S1_at	Hypothetical protein	4.61
Msa.1264.1.S1_at	Hypothetical protein	4.61
Mtr.12004.1.S1_at	Hypothetical protein	4.60
Mtr.33253.1.S1_at	Hypothetical protein	4.60
Mtr.2673.1.S1_s_at	Hypothetical protein	4.60
Mtr.16859.1.S1_at	Calcium-binding EF-hand	4.59
Mtr.13391.1.S1_at	Hypothetical protein	4.59
Mtr.5530.1.S1_s_at	Hypothetical protein	4.58
Mtr.37425.1.S1_at	Transcription factor WRKY10	4.57
Mtr.12209.1.S1_at	Phi-1 protein	4.57
Mtr.42466.1.S1_at	Phosphate transporter	4.57
Mtr.52294.1.S1_at	DNA photolyase	4.57
Mtr.16110.1.S1_at	Ankyrin protein	4.56
Mtr.38461.1.S1_at	Calmodulin-like domain protein kinase	4.56
Msa.937.1.S1_at	Hypothetical protein	4.56
Mtr.44296.1.S1_at	Cellulose synthase-like protein	4.55
Mtr.16849.1.S1_at	Protein kinase	4.55
Mtr.13422.1.S1_at	Hypothetical protein	4.55
Mtr.409.1.S1_at	Hypothetical protein	4.55
Mtr.38581.1.S1_at	Zinc finger protein	4.55
Mtr.43604.1.S1_s_at	Hypothetical protein	4.53
Mtr.12972.1.S1_at	Hypothetical protein	4.53
Mtr.40005.1.S1_at	Hypothetical protein	4.52
Mtr.17135.1.S1_at	Phospholipid-translocating P-type ATPase	4.52
Mtr.41232.1.S1_at	Cytochrome b5	4.52
Mtr.27830.1.S1_at	Arginine/serine-rich protein	4.52
Mtr.38027.1.S1_at	Hypothetical protein	4.52
Mtr.1516.1.S1_at	S-receptor kinase-like protein	4.52
Mtr.2541.1.S1_at	RuvB DNA helicase-like protein	4.52
Msa.1370.1.S1_at	Hypothetical protein	4.52
Mtr.51608.1.S1_at	Plant lipid transfer protein	4.51
Mtr.12703.1.S1_at	Hypothetical protein	4.51
Mtr.42229.1.S1_at	Hypothetical protein	4.51
Mtr.50954.1.S1_s_at	Hypothetical protein	4.51
Mtr.48842.1.S1_at	Hypothetical protein	4.51
Mtr.9195.1.S1_at	Hypothetical protein	4.50
Mtr.33603.1.S1_s_at	Transaldolase	4.50
Mtr.1854.1.S1_at	Hypothetical protein	4.50
Msa.1491.1.S1_at	Hypothetical protein	4.49
Mtr.13605.1.S1_at	Hypothetical protein	4.49
Mtr.15799.1.S1_at	Hypothetical protein	4.49
Mtr.38001.1.S1_at	Hypothetical protein	4.49
Mtr.12550.1.S1_at	Steroid sulfotransferase-like protein	4.49
Mtr.13183.1.S1_at	Hypothetical protein	4.49
Mtr.46642.1.S1_at	Protein kinase family	4.48
Mtr.11560.1.S1_s_at	ADP/ATP translocase-like protein	4.48
Mtr.48288.1.S1_s_at	Senescence-associated protein	4.48

Mtr.17825.1.S1_at	Hypothetical protein	4.47
Mtr.5506.1.S1_at	Protein phosphatase 2C	4.47
Msa.2981.1.S1_at	Hypothetical protein	4.46
Mtr.22270.1.S1_s_at	Hypothetical protein	4.46
Mtr.24411.1.S1_at	Hypothetical protein	4.45
Mtr.13823.1.S1_at	Hypothetical protein	4.45
Mtr.7313.1.S1_at	50S ribosomal protein L3-2	4.45
Mtr.13087.1.S1_at	Hypothetical protein	4.45
Mtr.37651.1.S1_s_at	5-enolpyruvylshikimate-3-phosphate synthase	4.44
Mtr.35231.1.S1_s_at	Pathogenesis-related protein	4.44
Mtr.10807.1.S1_at	Hypothetical protein	4.44
Mtr.41245.1.S1_at	Chorismate mutase	4.44
Msa.820.1.S1_at	Hypothetical protein	4.44
Mtr.32738.1.S1_at	Myo-inositol oxygenase	4.43
Msa.1800.1.S1_at	Hypothetical protein	4.43
Mtr.21266.1.S1_at	Hypothetical protein	4.43
Mtr.39168.1.S1_at	Dihydrodipicolinate synthase	4.43
Mtr.13110.1.S1_at	Fiber protein Fb2	4.42
Mtr.7440.1.S1_at	Hypothetical protein	4.41
Mtr.39664.1.S1_at	Hypothetical protein	4.41
Mtr.5605.1.S1_at	Protein phosphatase 2C	4.41
Mtr.12229.1.S1_s_at	RING zinc finger protein	4.41
Mtr.27180.1.S1_at	Hypothetical protein	4.41
Mtr.318.1.S1_at	OPR3 (12-oxophytodienoic acid 10,11-reductase)	4.41
Mtr.9440.1.S1_at	Hypothetical protein	4.40
Mtr.14950.1.S1_s_at	H+-transporting two-sector ATPase	4.40
Mtr.5260.1.S1_at	Hypothetical protein	4.40
Mtr.31621.1.S1_at	Hypothetical protein	4.40
Mtr.51564.1.S1_at	Glycoside hydrolase	4.40
Mtr.1532.1.S1_at	Hypothetical protein	4.40
Mtr.2945.1.S1_at	NADP+ reductase	4.39
Mtr.7373.1.S1_at	Hypothetical protein	4.39
Mtr.6315.1.S1_at	GCN4-complementing protein (GCP1)	4.39
Mtr.43201.1.S1_s_at	110 kDa 4SNc-Tudor domain protein	4.39
Mtr.23797.1.S1_s_at	Brassinosteroid-regulated protein BRU1	4.39
Mtr.45319.1.S1_at	Hypothetical protein	4.39
Mtr.33759.1.S1_at	F-box family protein	4.39
Mtr.12234.1.S1_s_at	40S ribosomal protein S4	4.38
Msa.821.1.S1_at	Hypothetical protein	4.38
Mtr.9209.1.S1_at	Hypothetical protein	4.38
Mtr.38773.1.S1_at	Protein kinase	4.37
Mtr.34161.1.S1_at	Amino acid permease-like protein	4.37
Mtr.12925.1.S1_at	RING/C3HC4/PHD zinc finger-like protein	4.37
Mtr.42649.1.S1_at	Hypothetical protein	4.37
Mtr.12146.1.S1_at	Hypothetical protein	4.37
Msa.3094.1.S1_at	Hypothetical protein	4.37
Mtr.31998.1.S1_at	Epoxide hydrolase	4.37
Mtr.24000.1.S1_at	Pentatricopeptide (PPR) repeat-containing protein	4.37
Mtr.15773.1.S1_at	Cyclin-like F-box	4.36
Mtr.18680.1.S1_at	Heat shock protein	4.36
Mtr.32924.1.S1_at	Heat shock protein	4.36
Mtr.10798.1.S1_at	Hypothetical protein	4.36
Mtr.30716.1.S1_at	Hypothetical protein	4.36
Mtr.40054.1.S1_at	Hypothetical protein	4.36

Mtr.43097.1.S1_at	Beta-amylase	4.36
Mtr.14781.1.S1_at	Hypothetical protein	4.35
Mtr.43825.1.S1_at	Hypothetical protein	4.35
Mtr.11118.1.S1_at	Calcium-dependent protein kinase	4.35
Mtr.48971.1.S1_at	Cytochrome oxidase	4.35
Msa.3189.1.S1_at	Hypothetical protein	4.35
Mtr.31364.1.S1_at	Phosphoglycerate dehydrogenase	4.35
Mtr.42808.1.S1_at	Myb-related transcription factor	4.35
Mtr.13338.1.S1_at	Hypothetical protein	4.35
Mtr.1356.1.S1_at	Hypothetical protein	4.34
Mtr.49421.1.S1_at	2OG-Fe(II) oxygenase	4.34
Mtr.38026.1.S1_at	Hypothetical protein	4.34
Mtr.43550.1.S1_at	Tyrosine aminotransferase	4.34
Mtr.48718.1.S1_at	Galactose-binding protein	4.34
Mtr.37208.1.S1_at	Hypothetical protein	4.34
Mtr.8633.1.S1_at	Hypothetical protein	4.34
Mtr.12430.1.S1_s_at	Mature anther-specific protein	4.33
Mtr.38197.1.S1_at	Hypothetical protein	4.33
Mtr.42600.1.S1_at	Hypothetical protein	4.33
Mtr.18476.1.S1_at	Hypothetical protein	4.33
Mtr.29063.1.S1_at	Hypothetical protein	4.33
Mtr.41435.1.S1_s_at	Hypothetical protein	4.33
Mtr.41945.1.S1_at	Hypothetical protein	4.32
Mtr.40076.1.S1_at	RNA Binding Protein	4.32
Mtr.7011.1.S1_at	Hypothetical protein	4.32
Mtr.27429.1.S1_at	Homocysteine S-methyltransferase 1	4.31
Mtr.11530.1.S1_at	Hypothetical protein	4.31
Mtr.23414.1.S1_at	Hypothetical protein	4.31
Mtr.18420.1.S1_at	Hypothetical protein	4.30
Mtr.42888.1.S1_s_at	Phi-1 protein	4.30
Mtr.35836.1.S1_at	Hypothetical protein	4.30
Mtr.12835.1.S1_at	Hypothetical protein	4.30
Mtr.44727.1.S1_at	Hypothetical protein	4.30
Msa.901.1.S1_a_at	Hypothetical protein	4.30
Mtr.13824.1.S1_at	Cyclic nucleotide-gated channel A	4.29
Mtr.40696.1.S1_at	Yeast pheromone receptor-like protein	4.29
Mtr.44596.1.S1_at	Hypothetical protein	4.29
Mtr.44341.1.S1_at	BRASSINOSTEROID INSENSITIVE 1-associated receptor kinase 1 precursor (E	4.28
Mtr.48884.1.S1_at	Hypothetical protein	4.27
Mtr.9442.1.S1_at	Hypothetical protein	4.27
Mtr.10732.1.S1_at	Hypothetical protein	4.27
Mtr.48306.1.S1_at	Hypothetical protein	4.27
Mtr.44417.1.S1_at	Probable disease resistance protein	4.27
Msa.1168.1.S1_at	Hypothetical protein	4.27
Mtr.31903.1.S1_at	Stellacyanin	4.27
Mtr.16145.1.S1_s_at	Protein kinase	4.27
Mtr.33855.1.S1_at	Alpha-N-acetylglucosaminidase	4.26
Mtr.39812.1.S1_s_at	Respiratory burst oxidase homolog	4.26
Mtr.42577.1.S1_s_at	Transcription factor WRKY31	4.26
Mtr.11768.1.S1_at	Hypothetical protein	4.26
Mtr.38808.1.S1_at	Class III peroxidase	4.26
Mtr.49473.1.S1_at	Carbamoyl-phosphate synthase L chain	4.26
Mtr.35294.1.S1_at	Protein kinase	4.26
Mtr.13033.1.S1_at	Hypothetical protein	4.26

Mtr.22599.1.S1_at	Histidine decarboxylase	4.25
Mtr.38358.1.S1_s_at	SINA1p	4.25
Mtr.27533.1.S1_s_at	Hypothetical protein	4.24
Mtr.9037.1.S1_at	Glycine-rich protein	4.24
Mtr.12551.1.S1_at	Monothiol glutaredoxin	4.24
Mtr.19308.1.S1_s_at	Cyclin-like F-box	4.23
Mtr.20704.1.S1_at	Nucleosome assembly protein	4.23
Mtr.40569.1.S1_at	Alpha-mannosidase	4.23
Mtr.45398.1.S1_at	Exo-1,3-beta-glucanase	4.23
Mtr.45783.1.S1_at	Short-chain dehydrogenase/reductase	4.23
Mtr.5723.1.S1_at	Hypothetical protein	4.23
Mtr.40272.1.S1_at	Nodulin-like protein	4.23
Mtr.40982.1.S1_at	Yippee-like protein	4.23
Mtr.45031.1.S1_at	Proteinase like protein	4.22
Mtr.24351.1.S1_s_at	Hypothetical protein	4.22
Mtr.50858.1.S1_s_at	Hypothetical protein	4.22
Mtr.32783.1.S1_at	Myosin	4.22
Mtr.40547.1.S1_at	bZIP transcription factor	4.22
Mtr.12916.1.S1_at	Hypothetical protein	4.22
Mtr.43257.1.S1_at	NAC-domain protein	4.22
Mtr.45583.1.S1_at	Hypothetical protein	4.22
Mtr.40615.1.S1_at	Phosphate transporter	4.21
Mtr.11749.1.S1_at	Hypothetical protein	4.21
Mtr.32999.1.S1_at	Hypothetical protein	4.21
Mtr.43908.1.S1_at	Suspensor-specific protein	4.21
Mtr.26369.1.S1_at	Hypothetical protein	4.21
Mtr.44843.1.S1_s_at	Hypothetical protein	4.20
Mtr.4053.1.S1_at	Protein kinase	4.20
Mtr.10424.1.S1_at	Ethylene responsive element binding factor	4.20
Mtr.9826.1.S1_at	Hypothetical protein	4.20
Mtr.40821.1.S1_at	Abscisic acid-activated protein kinase	4.20
Mtr.9407.1.S1_at	Hypothetical protein	4.19
Mtr.10388.1.S1_at	UDP-glucose 4-epimerase	4.19
Mtr.43116.1.S1_at	Hypothetical protein	4.19
Msa.1216.1.S1_at	Hypothetical protein	4.18
Mtr.9043.1.S1_at	LS1-like protein	4.18
Mtr.13028.1.S1_at	Hypothetical protein	4.18
Mtr.8508.1.S1_at	Pectinesterase	4.18
Mtr.40167.1.S1_s_at	Phenylalanine ammonia-lyase	4.18
Mtr.12208.1.S1_at	Pentameric polyubiquitin	4.18
Mtr.37280.1.S1_s_at	Calcium-dependent protein kinase	4.17
Mtr.12990.1.S1_at	Nucleolar histone deacetylase	4.17
Mtr.35141.1.S1_at	Receptor-like serine/threonine kinase	4.17
Mtr.14751.1.S1_at	Ribosome-binding factor	4.17
Mtr.2604.1.S1_x_at	Nodulin precursor	4.17
Mtr.27728.1.S1_s_at	Hypothetical protein	4.17
Mtr.45008.1.S1_at	Receptor protein kinase like protein	4.17
Mtr.21463.1.S1_at	Cys-rich domain protein	4.16
Mtr.49199.1.S1_at	Hypothetical protein	4.16
Mtr.4065.1.S1_at	Hypothetical protein	4.16
Mtr.6366.1.S1_at	Hypothetical protein	4.15
Mtr.11512.1.S1_at	Hypothetical protein	4.15
Mtr.32808.1.S1_at	Hypothetical protein	4.15
Mtr.9474.1.S1_at	MLO-like protein	4.15

Mtr.31327.1.S1_at	Protein kinase	4.14
Mtr.8994.1.S1_s_at	Hypothetical protein	4.14
Mtr.36944.1.S1_at	Hypothetical protein	4.14
Mtr.9291.1.S1_s_at	Hypothetical protein	4.13
Mtr.45278.1.S1_s_at	Hypothetical protein	4.13
Mtr.11485.1.S1_at	Imidazole glycerol phosphate synthase	4.13
Mtr.42387.1.S1_at	Hypothetical protein	4.13
Mtr.39873.1.S1_s_at	Myb-like protein	4.13
Msa.1306.1.S1_at	Hypothetical protein	4.12
Mtr.41196.1.S1_at	Hypothetical protein	4.12
Mtr.35204.1.S1_at	Glycine-rich protein	4.12
Mtr.32311.1.S1_at	Dynein light chain 1	4.12
Mtr.13329.1.S1_at	Hypothetical protein	4.12
Mtr.38757.1.S1_at	Myb-related transcription factor	4.12
Mtr.12293.1.S1_at	Receptor protein kinase PERK-like	4.12
Mtr.16505.1.S1_at	Aldehyde dehydrogenase	4.11
Mtr.8674.1.S1_at	Serine/threonine protein phosphatase	4.11
Mtr.43239.1.S1_at	Avr9 elicitor response protein	4.11
Mtr.39861.1.S1_at	Ethylene response factor ERF1	4.11
Msa.2974.1.S1_at	Hypothetical protein	4.11
Mtr.31491.1.S1_s_at	Cellulose synthase-like protein	4.11
Mtr.42911.1.S1_at	RING zinc finger protein	4.11
Mtr.7658.1.S1_at	Hypothetical protein	4.11
Mtr.42944.1.S1_x_at	Extensin class 1 protein	4.11
Msa.2736.1.S1_at	Hypothetical protein	4.11
Mtr.38956.1.S1_at	Hypothetical protein	4.10
Mtr.49055.1.S1_at	Hypothetical protein	4.10
Mtr.42340.1.S1_at	Importin alpha	4.10
Mtr.12229.1.S1_at	RING zinc finger protein	4.10
Mtr.14421.1.S1_at	Hypothetical protein	4.10
Mtr.6366.1.S1_s_at	Clathrin heavy chain	4.10
Mtr.42712.1.S1_at	Mitochondrial carrier-like protein	4.10
Mtr.38615.1.S1_at	Hypothetical protein	4.09
Mtr.1430.1.S1_at	Hypothetical protein	4.09
Mtr.6559.1.S1_at	Hypothetical protein	4.09
Mtr.11252.1.S1_s_at	Receptor kinase-like protein	4.09
Mtr.36943.1.S1_at	Hypothetical protein	4.08
Mtr.45088.1.S1_at	Vesicle transport v-SNARE 13	4.08
Mtr.4189.1.S1_s_at	Hypothetical protein	4.08
Mtr.49019.1.S1_at	Glycoside hydrolase	4.08
Mtr.24415.1.S1_s_at	Alpha-amylase	4.08
Mtr.21310.1.S1_at	Peptidase aspartic	4.07
Mtr.41557.1.S1_at	Hypothetical protein	4.07
Mtr.37633.1.S1_at	Allene oxide synthase	4.07
Mtr.9830.1.S1_at	Expansin	4.07
Mtr.31741.1.S1_at	WD repeat domain protein	4.07
Msa.3087.1.S1_at	Hypothetical protein	4.06
Mtr.34203.1.S1_at	UDP-glucuronosyltransferase	4.06
Mtr.38660.1.S1_at	Hypothetical protein	4.06
Mtr.36887.1.S1_at	Avr9/Cf-9 rapidly elicited protein	4.06
Mtr.34860.1.S1_s_at	Ubiquitin-protein ligase	4.05
Mtr.8471.1.S1_at	Transport protein subunit	4.05
Mtr.12559.1.S1_s_at	Glutathione S-transferase	4.05
Mtr.40406.1.S1_at	Hypothetical protein	4.05

Mtr.35926.1.S1_s_at	Hypothetical protein	4.05
Mtr.21812.1.S1_at	Hypothetical protein	4.04
Mtr.10766.1.S1_s_at	Amino acid permease	4.04
Mtr.10176.1.S1_at	Hypothetical protein	4.04
Mtr.42547.1.S1_s_at	Respiratory burst oxidase homolog	4.04
Mtr.1398.1.S1_s_at	Hypothetical protein	4.03
Mtr.44974.1.S1_at	Skp1p-like protein	4.03
Mtr.27935.1.S1_s_at	Transcription factor WRKY4	4.03
Mtr.19170.1.S1_at	Hypothetical protein	4.03
Mtr.29990.1.S1_at	MYB-related protein	4.03
Mtr.20316.1.S1_at	Purine/pyrimidine phosphoribosyl transferase	4.03
Mtr.13259.1.S1_at	Methylmalonate semi-aldehyde dehydrogenase	4.03
Mtr.22118.1.S1_s_at	5-enol-pyruvylshikimate-phosphate synthase	4.03
Mtr.8632.1.S1_s_at	Hypothetical protein	4.03
Mtr.38126.1.S1_at	Hypothetical protein	4.03
Msa.2671.1.S1_s_at	Hypothetical protein	4.03
Mtr.11232.1.S1_at	Serine/threonine protein kinase	4.03
Mtr.9106.1.S1_at	Hypothetical protein	4.02
Msa.2542.1.S1_at	Hypothetical protein	4.02
Mtr.39021.1.S1_at	AP2 domain transcription factor	4.02
Mtr.27354.1.S1_s_at	RPM1-interacting protein	4.01
Mtr.19347.1.S1_s_at	Hypothetical protein	4.01
Mtr.20265.1.S1_at	Hypothetical protein	4.01
Mtr.320.1.S1_at	12-oxophytodienoic acid 10, 11-reductase	4.01
Mtr.12449.1.S1_at	Hypothetical protein	4.00
Mtr.29264.1.S1_at	Isp4 like protein,	4.00
Mtr.4870.1.S1_s_at	Hypothetical protein	4.00
Mtr.14494.1.S1_at	Dihydroorotate dehydrogenase 1	4.00
Mtr.12609.1.S1_at	Acyl CoA oxidase	4.00
Mtr.18366.1.S1_at	Curculin-like (mannose-binding) lectin	3.99
Msa.1622.1.S1_at	Hypothetical protein	3.98
Mtr.35611.1.S1_at	Hypothetical protein	3.98
Mtr.40182.1.S1_at	Hypothetical protein	3.98
Mtr.38837.1.S1_s_at	Hypothetical protein	3.98
Mtr.7556.1.S1_at	AP2 domain containing protein	3.98
Mtr.9388.1.S1_at	WRKY transcription factor	3.98
Mtr.31455.1.S1_at	Hypothetical protein	3.97
Mtr.10773.1.S1_at	Wasl protein	3.97
Mtr.9041.1.S1_s_at	Hypothetical protein	3.97
Mtr.12455.1.S1_at	Hypothetical protein	3.97
Mtr.23965.1.S1_s_at	Wound-inducible protein	3.97
Mtr.37900.1.S1_at	Pantothenate kinase 1 (Pantothenic acid kinase 1)	3.97
Mtr.41525.1.S1_at	Hypothetical protein	3.97
Mtr.13594.1.S1_s_at	Lipase-like protein	3.96
Mtr.40005.1.S1_s_at	Hypothetical protein	3.96
Mtr.42525.1.S1_x_at	Hypothetical protein	3.96
Msa.1053.1.S1_at	Hypothetical protein	3.96
Mtr.6963.1.S1_at	Hypothetical protein	3.95
Mtr.46097.1.S1_at	Hypothetical protein	3.95
Mtr.23686.1.S1_at	DNA-directed RNA polymerase subunit	3.95
Mtr.32957.1.S1_at	Hypothetical protein	3.95
Mtr.50704.1.S1_s_at	Aldehyde dehydrogenase	3.95
Mtr.10325.1.S1_at	Chitinase	3.95
Mtr.19407.1.S1_at	Hypothetical protein	3.95

Mtr.15053.1.S1_at	Thaumatin	3.94
Mtr.20850.1.S1_at	Zn-finger, DHHC type	3.94
Mtr.11633.1.S1_at	Hypothetical protein	3.94
Mtr.16691.1.S1_at	Myb, DNA-binding protein	3.94
Mtr.29288.1.S1_x_at	Hypothetical protein	3.94
Mtr.13696.1.S1_at	Hypothetical protein	3.94
Mtr.7569.1.S1_at	Cytosolic fructose-1,6-bisphosphatase	3.94
Msa.1051.1.S1_at	Hypothetical protein	3.94
Mtr.4569.1.S1_at	Hypothetical protein	3.93
Mtr.9112.1.S1_at	Steroid 22-alpha-hydroxylase	3.93
Mtr.28237.1.S1_s_at	Agenet domain-containing protein	3.93
Mtr.35724.1.S1_at	Hypothetical protein	3.93
Msa.1075.1.S1_at	Hypothetical protein	3.93
Mtr.35925.1.S1_at	Hypothetical protein	3.93
Mtr.38129.1.S1_at	RNA-binding protein	3.93
Mtr.6906.1.S1_at	Acyl-activating enzyme	3.92
Mtr.3105.1.S1_at	PDR-type ABC transporter	3.92
Mtr.37020.1.S1_at	NADH-ubiquinone oxidoreductase subunit	3.92
Mtr.41259.1.S1_at	Ring finger E3 ligase	3.92
Mtr.51646.1.S1_at	Carbohydrate-binding, CenC-like protein	3.92
Mtr.46408.1.S1_at	Cytochrome P450	3.92
Msa.3043.1.S1_at	Hypothetical protein	3.91
Mtr.43512.1.S1_at	Hypothetical protein	3.91
Mtr.42782.1.S1_s_at	Hypothetical protein	3.91
Mtr.44169.1.S1_at	Spermatid-specific protein	3.90
Mtr.48644.1.S1_at	Hypothetical protein	3.90
Mtr.6797.1.S1_at	Hypothetical protein	3.90
Mtr.12397.1.S1_at	Cytochrome P450	3.89
Mtr.39871.1.S1_at	Hypothetical protein	3.89
Mtr.31600.1.S1_at	Hypothetical protein	3.89
Mtr.41814.1.S1_at	PRIB5 protein	3.89
Mtr.32948.1.S1_at	Hypothetical protein	3.89
Mtr.457.1.S1_s_at	Hypothetical protein	3.89
Mtr.10061.1.S1_at	Hypothetical protein	3.88
Mtr.36255.1.S1_at	Aquaporin-like transmembrane channel protein	3.88
Mtr.40178.1.S1_at	Hypothetical protein	3.88
Mtr.9139.1.S1_at	Hypothetical protein	3.88
Mtr.11543.1.S1_at	Gamma-glutamyl hydrolase	3.88
Mtr.6515.1.S1_at	Dihydrodipicolinate synthase	3.88
Mtr.11396.1.S1_at	Oxysterol-binding protein	3.88
Mtr.40876.1.S1_at	Hypothetical protein	3.88
Mtr.38871.1.S1_at	Hypothetical protein	3.88
Mtr.12425.1.S1_at	Mitogen-activated protein kinase MMK2	3.88
Mtr.10127.1.S1_at	Phospholipase-like protein	3.87
Mtr.21466.1.S1_at	Serine/threonine protein kinase	3.87
Mtr.18298.1.S1_at	Leucine-rich repeat	3.87
Mtr.1157.1.S1_s_at	Hypothetical protein	3.87
Mtr.13216.1.S1_at	Hypothetical protein	3.87
Mtr.46189.1.S1_s_at	Disease resistance protein	3.86
Mtr.38835.1.S1_at	Cytochrome C biosynthesis heme-carrier protein	3.86
Mtr.11968.1.S1_at	Alpha galactosidase	3.86
Mtr.43024.1.S1_at	6-phosphogluconate dehydrogenase	3.86
Mtr.14019.1.S1_at	LysM domain-containing receptor-like kinase	3.85
Mtr.22597.1.S1_s_at	Serine decarboxylase	3.85

Mtr.43455.1.S1_at	Hypothetical protein	3.85
Mtr.10389.1.S1_s_at	UDP-glucose 4-epimerase	3.85
Mtr.7429.1.S1_s_at	Hypothetical protein	3.85
Mtr.2424.1.S1_at	Nodulin-like protein	3.84
Mtr.39061.1.S1_at	Hypothetical protein	3.84
Mtr.5877.1.S1_at	Thioredoxin	3.84
Mtr.39061.1.S1_s_at	Hypothetical protein	3.84
Mtr.50547.1.S1_at	RHO protein GDP dissociation inhibitor	3.84
Mtr.20739.1.S1_s_at	Protein kinase	3.84
Msa.2600.1.S1_at	Hypothetical protein	3.84
Mtr.28325.1.S1_at	Branched-chain alpha-keto acid decarboxylase E1 beta subunit	3.84
Mtr.39422.1.S1_s_at	Hypothetical protein	3.83
Mtr.12664.1.S1_at	Hypothetical protein	3.83
Mtr.42955.1.S1_at	Cytochrome P450 76C4	3.83
Mtr.12402.1.S1_s_at	Beta-galactosidase	3.82
Mtr.43104.1.S1_at	1-aminocyclopropanecarboxylic acid oxidase	3.82
Mtr.8701.1.S1_at	Leucine rich repeat protein	3.82
Mtr.11018.1.S1_s_at	Hypothetical protein	3.82
Mtr.33274.1.S1_at	Hypothetical protein	3.82
Mtr.18069.1.S1_x_at	Disease resistance protein	3.81
Msa.1174.1.S1_at	Hypothetical protein	3.81
Mtr.17868.1.S1_at	Hypothetical protein	3.81
Mtr.42180.1.S1_s_at	Hypothetical protein	3.81
Mtr.44105.1.S1_at	Nucleolar RNA-binding Nop10p-like protein	3.81
Mtr.10806.1.S1_at	Hypothetical protein	3.81
Mtr.50038.1.S1_at	No apical meristem (NAM) protein	3.81
Mtr.13624.1.S1_at	Hypothetical protein	3.81
Mtr.33257.1.S1_s_at	Stromal membrane-associated protein	3.80
Mtr.35045.1.S1_s_at	Rab escort protein	3.80
Mtr.5717.1.S1_at	Receptor protein kinase	3.80
Mtr.20156.1.S1_s_at	Hypothetical protein	3.80
Msa.2746.1.S1_at	Hypothetical protein	3.80
Mtr.49088.1.S1_at	Rhodanese-like protein	3.79
Mtr.41349.1.S1_s_at	Hydrolase	3.79
Mtr.23865.1.S1_at	Putative disease resistance protein	3.79
Mtr.18642.1.S1_at	Hypothetical protein	3.79
Mtr.12210.1.S1_at	Acyl carrier protein	3.79
Mtr.8642.1.S1_at	Serine palmitoyltransferase	3.79
Mtr.21812.1.S1_s_at	Hypothetical protein	3.79
Mtr.17962.1.S1_at	DNA-binding WRKY	3.78
Mtr.47843.1.S1_at	Monosaccharide transporter	3.78
Mtr.27641.1.S1_at	Hypothetical protein	3.78
Mtr.26307.1.S1_at	GDSL-motif lipase/hydrolase protein	3.77
Mtr.51442.1.S1_at	Protein kinase	3.77
Mtr.40538.1.S1_at	Glutamate decarboxylase	3.77
Mtr.5966.1.S1_at	Class III peroxidase	3.77
Mtr.7323.1.S1_at	Hypothetical protein	3.76
Mtr.37312.1.S1_at	Heat shock protein 70-3	3.76
Msa.2767.1.S1_at	Hypothetical protein	3.76
Mtr.10581.1.S1_at	Hypothetical protein	3.76
Mtr.30738.1.S1_at	Uracil phosphoribosyltransferase	3.76
Mtr.27778.1.S1_at	Serine/threonine protein kinase	3.75
Mtr.9182.1.S1_at	Hypothetical protein	3.75
Mtr.13273.1.S1_at	TINY-like protein	3.75

Mtr.45463.1.S1_at	Xyloglucan endotransglucosylase/hydrolase protein	3.75
Mtr.47022.1.S1_s_at	Wound-inducible protein	3.75
Mtr.43073.1.S1_s_at	Poly(A)-binding protein C-terminal interacting protein	3.74
Mtr.44365.1.S1_s_at	Adenine-specific DNA modification methyltransferase	3.74
Mtr.19347.1.S1_at	Major intrinsic protein	3.74
Mtr.29288.1.S1_at	Hypothetical protein	3.74
Mtr.10514.1.S1_at	Acyl-activating enzyme	3.74
Mtr.33285.1.S1_at	Hypothetical protein	3.74
Mtr.33152.1.S1_at	Hypothetical protein	3.74
Mtr.49556.1.S1_at	RNA polymerase	3.74
Mtr.30654.1.S1_at	Hypothetical protein	3.73
Mtr.37655.1.S1_at	Nucleolar histone deacetylase	3.73
Mtr.43192.1.S1_at	Mature anther-specific protein	3.73
Mtr.44922.1.S1_at	Glycine-rich protein	3.73
Mtr.14729.1.S1_at	RNA-processing protein	3.73
Mtr.37225.1.S1_at	Adenine nucleotide translocator	3.73
Mtr.40099.1.S1_at	Hypothetical protein	3.72
Mtr.42149.1.S1_at	Hypothetical protein	3.72
Mtr.10779.1.S1_at	Chalcone-flavonone isomerase	3.72
Mtr.45140.1.S1_at	ABI3 family regulatory protein	3.72
Mtr.40144.1.S1_at	Glutamate-rich protein	3.72
Mtr.35913.1.S1_at	Integral membrane protein	3.72
Msa.1472.1.S1_at	Hypothetical protein	3.71
Mtr.5571.1.S1_s_at	Hypothetical protein	3.71
Mtr.39083.1.S1_at	Proliferating-cell nucleolar protein	3.71
Mtr.40058.1.S1_x_at	Nodulin	3.71
Mtr.42944.1.S1_a_at	Extensin class 1 protein	3.70
Mtr.7448.1.S1_s_at	Hypothetical protein	3.70
Mtr.33339.1.S1_at	Hypothetical protein	3.70
Mtr.45117.1.S1_at	Guanylate kinase	3.70
Mtr.43355.1.S1_at	Hypothetical protein	3.70
Mtr.32291.1.S1_at	GTP cyclohydrolase II	3.70
Mtr.33361.1.S1_at	Hypothetical protein	3.70
Msa.1942.1.S1_at	Hypothetical protein	3.69
Mtr.6979.1.S1_s_at	Glyceraldehyde-3-phosphate dehydrogenase	3.69
Mtr.40977.1.S1_at	Hypothetical protein	3.69
Mtr.9523.1.S1_at	Hypothetical protein	3.69
Mtr.39540.1.S1_at	ABC transporter	3.69
Mtr.12378.1.S1_at	Serine/threonine-protein kinase	3.69
Mtr.35813.1.S1_at	Hypothetical protein	3.68
Mtr.50388.1.S1_at	TPR repeat protein	3.68
Mtr.41051.1.S1_at	Hypothetical protein	3.68
Mtr.2254.1.S1_at	Hypothetical protein	3.68
Mtr.10338.1.S1_s_at	Hypothetical protein	3.68
Mtr.16703.1.S1_at	Disease resistance protein	3.67
Mtr.9399.1.S1_at	CDPK-related protein kinase	3.67
Mtr.10728.1.S1_at	Hypothetical protein	3.67
Mtr.8658.1.S1_at	Dehydroquinate synthase	3.67
Mtr.12698.1.S1_at	Hypothetical protein	3.67
Mtr.42147.1.S1_at	Intracellular pathogenesis related protein	3.67
Mtr.12597.1.S1_at	TIR-NBS-LRR type R protein	3.67
Mtr.41733.1.S1_at	Hypothetical protein	3.66
Mtr.44772.1.S1_at	Probable elongation factor G	3.66
Mtr.50050.1.S1_at	Isopenicillin N synthetase	3.66

Mtr.45354.1.S1_at	Whitefly-induced gp91-phox	3.66
Mtr.3871.1.S1_at	CCAAT-box-binding transcription factor-like protein	3.66
Mtr.31548.1.S1_at	Metacaspase	3.66
Mtr.37495.1.S1_at	SCOF-1	3.66
Mtr.10565.1.S1_at	Hypothetical protein	3.66
Mtr.18344.1.S1_at	Protein kinase	3.65
Mtr.37260.1.S1_at	Agenet domain-containing protein	3.65
Msa.2783.1.S1_at	Hypothetical protein	3.64
Mtr.50583.1.S1_at	Glycoside hydrolase	3.64
Mtr.45507.1.S1_at	Hypothetical protein	3.64
Mtr.1676.1.S1_at	Hypothetical protein	3.64
Msa.2622.1.S1_at	Hypothetical protein	3.64
Mtr.43169.1.S1_at	Hypothetical protein	3.64
Mtr.23261.1.S1_at	Hypothetical protein	3.63
Mtr.37972.1.S1_at	Hypothetical protein	3.63
Mtr.41809.1.S1_s_at	Non-phototropic hypocotyl protein	3.63
Mtr.37928.1.S1_at	Thiamine pyrophosphokinase	3.63
Mtr.8933.1.S1_at	1,2-dioxygenase	3.63
Mtr.41172.1.S1_s_at	Hypothetical protein	3.62
Mtr.13267.1.S1_at	Hypothetical protein	3.62
Mtr.10621.1.S1_at	Hypothetical protein	3.62
Mtr.13077.1.S1_at	Hypothetical protein	3.62
Mtr.14663.1.S1_at	Major intrinsic protein	3.62
Mtr.9656.1.S1_at	Response regulator protein	3.62
Mtr.44555.1.S1_at	Amino acid permease-like protein	3.62
Mtr.23049.1.S1_at	Hypothetical protein	3.62
Mtr.50422.1.S1_at	Hypothetical protein	3.62
Mtr.29715.1.S1_at	Hypothetical protein	3.62
Mtr.22662.1.S1_at	NADP-dependent malic enzyme	3.62
Mtr.320.1.S1_x_at	12-oxophytodienoic acid 10, 11-reductase	3.62
Mtr.11114.1.S1_at	Hypothetical protein	3.62
Mtr.33096.1.S1_at	Heterogeneous nuclear ribonucleoprotein A2/B1-like protein	3.62
Mtr.14627.1.S1_at	Hypothetical protein	3.61
Mtr.34632.1.S1_s_at	Seven transmembrane helix receptor	3.61
Mtr.35440.1.S1_at	Hypothetical protein	3.61
Mtr.31454.1.S1_s_at	Hypothetical protein	3.61
Mtr.3533.1.S1_at	Hypothetical protein	3.61
Mtr.51889.1.S1_s_at	Peptidase	3.61
Mtr.18402.1.S1_at	Hypothetical protein	3.61
Mtr.43658.1.S1_at	Hypothetical protein	3.61
Mtr.41781.1.S1_at	Cell cycle serine/threonine-protein kinase	3.60
Mtr.35741.1.S1_at	Hypothetical protein	3.60
Mtr.4934.1.S1_s_at	Hypothetical protein	3.60
Mtr.35008.1.S1_at	RNA polymerase	3.60
Mtr.9697.1.S1_at	Hypothetical protein	3.60
Mtr.39049.1.S1_at	WD40-repeat protein	3.60
Mtr.13010.1.S1_at	Xyloglucan endotransglucosylase/hydrolase protein	3.59
Mtr.11175.1.S1_at	Hypothetical protein	3.59
Mtr.36256.1.S1_at	Hypothetical protein	3.59
Mtr.24415.1.S1_at	Alpha-amylase	3.59
Mtr.46918.1.S1_at	Hypothetical protein	3.59
Mtr.4379.1.S1_at	Hypothetical protein	3.59
Mtr.45568.1.S1_at	Phosphate transporter	3.59
Mtr.44462.1.S1_at	Heat shock protein	3.59

Mtr.20117.1.S1_s_at	Peptidyl-prolyl cis-trans isomerase	3.59
Mtr.8589.1.S1_at	Cinnamyl-alcohol dehydrogenase (CAD)	3.58
Mtr.51829.1.S1_at	Response regulator receiver	3.58
Mtr.31755.1.S1_at	Diacylglycerol kinase	3.58
Mtr.36311.1.S1_at	Hypothetical protein	3.58
Mtr.31337.1.S1_at	Serine/threonine kinase	3.58
Mtr.38691.1.S1_s_at	Hypothetical protein	3.58
Mtr.7950.1.S1_at	Receptor protein kinase	3.58
Mtr.15228.1.S1_s_at	Pre-mRNA processing ribonucleoprotein	3.58
Mtr.33544.1.S1_at	Beta-galactosidase	3.58
Mtr.42855.1.S1_s_at	Plastidic ATP/ADP transporter	3.58
Mtr.38362.1.S1_at	Anthranilate N-benzoyltransferase-like protein	3.58
Mtr.25254.1.S1_at	DnaJ-like protein	3.58
Mtr.33133.1.S1_at	IRE homolog; protein kinase-like protein	3.58
Mtr.267.1.S1_at	Hypothetical protein	3.58
Mtr.33601.1.S1_s_at	Nodulin26-like major intrinsic protein	3.57
Mtr.23284.1.S1_at	Calcium-binding EF-hand family protein	3.57
Mtr.8606.1.S1_at	O-methyltransferase	3.57
Mtr.37994.1.S1_at	Protein kinase	3.56
Mtr.42023.1.S1_at	Lipase	3.56
Mtr.41029.1.S1_s_at	Ankyrin repeat-containing protein	3.56
Mtr.41703.1.S1_at	Lipoxygenase	3.55
Mtr.32533.1.S1_s_at	Hypothetical protein	3.55
Mtr.49896.1.S1_at	Hypothetical protein	3.55
Mtr.40241.1.S1_at	Cinnamoyl-CoA reductase	3.55
Mtr.46756.1.S1_s_at	Hypothetical protein	3.55
Mtr.32998.1.S1_s_at	Hypothetical protein	3.55
Mtr.7754.1.S1_s_at	Vestitone reductase	3.55
Mtr.11202.1.S1_s_at	Hypothetical protein	3.55
Mtr.12684.1.S1_at	Hypothetical protein	3.54
Mtr.32531.1.S1_s_at	Hypothetical protein	3.54
Mtr.4454.1.S1_at	Hypothetical protein	3.54
Mtr.9950.1.S1_at	Hypothetical protein	3.54
Mtr.35093.1.S1_at	Hypothetical protein	3.54
Mtr.5263.1.S1_at	Hypothetical protein	3.54
Mtr.11200.1.S1_at	Zinc finger protein	3.54
Mtr.13938.1.S1_at	Mei2-like protein	3.54
Mtr.6484.1.S1_at	Phosphate transporter	3.54
Mtr.47490.1.S1_s_at	Cytochrome P450 71D9	3.54
Mtr.9792.1.S1_at	Phage-type RNA polymerase	3.54
Mtr.9002.1.S1_at	1-aminocyclopropane-1-carboxylic acid oxidase	3.54
Mtr.4815.1.S1_at	Hypothetical protein	3.54
Mtr.30502.1.S1_at	Hypothetical protein	3.54
Mtr.18525.1.S1_at	Pyridoxal-dependent decarboxylase	3.53
Mtr.38894.1.S1_s_at	Hypothetical protein	3.53
Mtr.42610.1.S1_at	Acyl-activating enzyme	3.53
Mtr.9525.1.S1_at	Hypothetical protein	3.53
Mtr.33223.1.S1_x_at	Anionic Peroxidase	3.53
Mtr.46966.1.S1_s_at	Wound-induced GSK-3-like protein	3.53
Mtr.44444.1.S1_at	MADS box interactor-like protein	3.52
Mtr.3066.1.S1_s_at	Hypothetical protein	3.52
Msa.2535.1.S1_at	Hypothetical protein	3.52
Mtr.45145.1.S1_s_at	Lipase class 3-like	3.52
Mtr.16393.1.S1_at	Hypothetical protein	3.52

Mtr.28948.1.S1_at	Hypothetical protein	3.52
Mtr.28840.1.S1_at	Hypothetical protein	3.52
Mtr.41270.1.S1_at	Beta-adaptin-like protein A	3.52
Mtr.40735.1.S1_at	Hypothetical protein	3.52
Mtr.45225.1.S1_at	Ankyrin-like protein	3.52
Msa.1474.1.S1_at	Hypothetical protein	3.52
Msa.2952.1.S1_at	Hypothetical protein	3.51
Mtr.40307.1.S1_at	Citrate synthase	3.51
Mtr.13389.1.S1_at	Receptor protein kinase	3.51
Mtr.8444.1.S1_s_at	Hypothetical protein	3.51
Mtr.46539.1.S1_at	bZIP DNA-binding protein	3.51
Mtr.692.1.S1_at	Hypothetical protein	3.51
Mtr.32061.1.S1_at	Hypothetical protein	3.51
Mtr.41853.1.S1_at	Zinc finger, C3HC4 type protein	3.51
Mtr.10147.1.S1_at	Auxin-induced SAUR-like protein	3.50
Mtr.2533.1.S1_at	Hypothetical protein	3.50
Mtr.9055.1.S1_at	Branched-chain alpha-keto acid decarboxylase E1 beta subunit	3.50
Mtr.28090.1.S1_at	Hypothetical protein	3.50
Mtr.44992.1.S1_s_at	Serine/threonine protein kinase	3.50
Mtr.45647.1.S1_at	Amidase	3.50
Mtr.12962.1.S1_at	Rho GDP dissociation inhibitor 2	3.50
Mtr.37677.1.S1_s_at	Hypothetical protein	3.50
Mtr.44887.1.S1_at	Hypothetical protein	3.50
Mtr.21028.1.S1_at	Hypothetical protein	3.50
Mtr.31290.1.S1_at	NHL repeat-containing protein	3.50
Mtr.50930.1.S1_at	Helix-loop-helix DNA-binding protein	3.50
Mtr.44211.1.S1_at	Hypothetical protein	3.49
Mtr.38258.1.S1_at	Peroxisomal acetoacetyl-coenzyme A thiolase	3.49
Mtr.36196.1.S1_s_at	Lipase-like protein	3.49
Mtr.34124.1.S1_at	ARF GAP-like zinc finger-containing protein	3.49
Mtr.1526.1.S1_at	Hypothetical protein	3.49
Mtr.47932.1.S1_at	Xylulose kinase	3.48
Mtr.10342.1.S1_at	Xyloglucan endotransglucosylase/hydrolase protein	3.48
Mtr.43390.1.S1_at	Hypothetical protein	3.48
Mtr.44614.1.S1_at	Hypothetical protein	3.48
Mtr.44647.1.S1_at	DNA/RNA binding protein	3.47
Mtr.428.1.S1_s_at	Leucine rich repeat protein family	3.47
Mtr.13769.1.S1_at	Exonuclease-like protein	3.47
Mtr.30741.1.S1_at	Allantoin permease	3.46
Msa.1220.1.S1_at	Hypothetical protein	3.46
Msa.3071.1.S1_at	Hypothetical protein	3.46
Mtr.23415.1.S1_at	Hypothetical protein	3.46
Mtr.12342.1.S1_at	bHLH transcription factor	3.46
Mtr.37612.1.S1_at	Hypothetical protein	3.46
Mtr.33353.1.S1_at	Microtubule-associated protein	3.46
Mtr.39204.1.S1_at	Hypothetical protein	3.45
Mtr.9031.1.S1_at	Nucellin-like aspartic protease	3.45
Mtr.38626.1.S1_at	Fumarate hydratase	3.45
Msa.1176.1.S1_at	Hypothetical protein	3.45
Mtr.3889.1.S1_at	Hypothetical protein	3.45
Mtr.7267.1.S1_at	Hypothetical protein	3.45
Mtr.8704.1.S1_at	Hypothetical protein	3.45
Mtr.49932.1.S1_at	Ankyrin protein	3.44
Mtr.45496.1.S1_at	Profilin	3.44

Mtr.41441.1.S1_at	Hypothetical protein	3.44
Mtr.10725.1.S1_at	Basic blue copper protein	3.44
Mtr.6886.1.S1_at	Hypothetical protein	3.44
Mtr.1233.1.S1_at	KAP-2	3.44
Mtr.18695.1.S1_at	Hypothetical protein	3.44
Mtr.37771.1.S1_at	Ser/Thr protein kinase	3.44
Mtr.42269.1.S1_at	Transcription factor (E2F)	3.44
Mtr.51028.1.S1_at	Aspartate/glutamate/uridylate kinase	3.44
Mtr.38070.1.S1_at	Hypothetical protein	3.44
Mtr.49905.1.S1_at	Hypothetical protein	3.43
Mtr.32218.1.S1_s_at	Hypothetical protein	3.43
Mtr.24467.1.S1_s_at	Hypothetical protein	3.43
Mtr.37913.1.S1_at	Acyl-CoA oxidase	3.43
Mtr.5083.1.S1_at	Hypothetical protein	3.43
Mtr.15650.1.S1_s_at	Adenylate kinase	3.43
Mtr.39683.1.S1_at	Hypothetical protein	3.43
Mtr.41760.1.S1_at	Calcium-dependent protein kinase	3.43
Mtr.10536.1.S1_at	Omega-3 fatty acid desaturase	3.42
Mtr.13482.1.S1_at	Ceramide kinase	3.42
Mtr.13565.1.S1_at	Hypothetical protein	3.42
Mtr.34768.1.S1_at	Hypothetical protein	3.42
Mtr.43957.1.S1_s_at	Peroxisomal copper-containing amine oxidase	3.42
Mtr.27800.1.S1_s_at	Synptobrevin-related protein	3.42
Mtr.17544.1.S1_at	Hypothetical protein	3.41
Mtr.17714.1.S1_at	Pentatricopeptide repeat protein	3.41
Mtr.36788.1.S1_at	Hypothetical protein	3.41
Mtr.47465.1.S1_at	Nitrate transporter	3.41
Mtr.41373.1.S1_at	Phi-1-like protein	3.41
Mtr.2735.1.S1_at	Hypothetical protein	3.40
Mtr.7227.1.S1_at	Hypothetical protein	3.40
Mtr.16634.1.S1_s_at	Hypothetical protein	3.40
Mtr.50478.1.S1_at	Phenylalanine/histidine ammonia-lyas	3.40
Mtr.11076.1.S1_at	Hypothetical protein	3.40
Mtr.48868.1.S1_at	Phosphofructokinase	3.40
Mtr.40131.1.S1_at	Peroxidase	3.40
Mtr.42359.1.S1_at	Hypothetical protein	3.40
Mtr.45896.1.S1_at	Translation initiation factor	3.40
Mtr.10349.1.S1_s_at	Hypothetical protein	3.40
Msa.969.1.S1_at	Hypothetical protein	3.39
Mtr.4041.1.S1_at	Nucleic acid binding protein	3.39
Mtr.39314.1.S1_at	Hypothetical protein	3.39
Mtr.38570.1.S1_at	YGL010w-like protein	3.39
Mtr.13667.1.S1_at	Hypothetical protein	3.39
Mtr.10240.1.S1_at	Hypothetical protein	3.39
Mtr.40592.1.S1_at	Ferredoxin III	3.39
Mtr.5343.1.S1_s_at	Type-A response regulator	3.39
Mtr.48412.1.S1_s_at	Beta-galactosidase	3.39
Mtr.10224.1.S1_at	MYB-like DNA-binding domain protein	3.39
Mtr.28849.1.S1_at	Amidase-like protein	3.38
Mtr.49094.1.S1_at	Peptidase aspartic	3.38
Mtr.48411.1.S1_s_at	Beta-galactosidase	3.38
Mtr.43608.1.S1_at	rRNA processing protein	3.38
Mtr.20288.1.S1_at	Transcriptional factor B3	3.38
Mtr.38966.1.S1_at	Ser/Thr kinase	3.38

Mtr.42391.1.S1_at	Hypothetical protein	3.38
Mtr.648.1.S1_at	Peptide transporter	3.38
Mtr.12830.1.S1_at	Hypothetical protein	3.38
Mtr.38304.1.S1_at	Hypothetical protein	3.38
Mtr.35791.1.S1_s_at	Light-induced protein	3.38
Mtr.13806.1.S1_at	Hypothetical protein	3.38
Mtr.45833.1.S1_s_at	Hypothetical protein	3.38
Mtr.47703.1.S1_at	Hypothetical protein	3.38
Mtr.11878.1.S1_at	Receptor protein kinase	3.38
Mtr.45297.1.S1_at	Hypothetical protein	3.37
Mtr.32889.1.S1_at	Hypothetical protein	3.37
Mtr.45545.1.S1_at	Hypothetical protein	3.37
Mtr.40801.1.S1_at	Hypothetical protein	3.37
Mtr.37280.1.S1_at	Calcium-dependent protein kinase	3.37
Mtr.30161.1.S1_s_at	Basic/leucine zipper protein	3.37
Mtr.10542.1.S1_at	Hypothetical protein	3.37
Mtr.28411.1.S1_s_at	Hypothetical protein	3.37
Mtr.16216.1.S1_at	Serine/threonine protein kinase	3.37
Mtr.41875.1.S1_at	Receptor protein kinase	3.37
Mtr.33279.1.S1_at	Aux/IAA	3.37
Mtr.2238.1.S1_s_at	Repetitive proline-rich cell wall protein	3.36
Mtr.44661.1.S1_at	Hypothetical protein	3.36
Mtr.39602.1.S1_at	Receptor protein kinase	3.36
Mtr.42760.1.S1_at	Hypothetical protein	3.36
Mtr.30257.1.S1_at	Hypothetical protein	3.36
Mtr.9028.1.S1_at	Hypothetical protein	3.36
Mtr.12920.1.S1_s_at	MAP kinase-like protein	3.36
Mtr.21815.1.S1_at	2OG-Fe(II) oxygenase	3.36
Mtr.2218.1.S1_at	Ubiquitin-protein ligase	3.36
Mtr.11557.1.S1_at	Hypothetical protein	3.36
Mtr.50292.1.S1_at	Hypothetical protein	3.36
Mtr.45259.1.S1_at	LEM3-like protein	3.36
Mtr.43597.1.S1_at	Hypothetical protein	3.35
Mtr.32122.1.S1_at	Cellulose synthase-like protein	3.35
Mtr.39259.1.S1_at	Hypothetical protein	3.35
Msa.1908.1.S1_at	Hypothetical protein	3.35
Mtr.14930.1.S1_at	Hypothetical protein	3.35
Mtr.47863.1.S1_s_at	Transcription factor WRKY10	3.35
Mtr.2249.1.S1_at	Hypothetical protein	3.35
Mtr.30756.1.S1_s_at	Hypothetical protein	3.35
Mtr.4322.1.S1_at	Cytochrome oxidase subunit III	3.35
Mtr.38935.1.S1_at	Myo-inositol transporter 1	3.35
Mtr.38752.1.S1_at	Geranyl diphosphate synthase small subunit	3.35
Mtr.5770.1.S1_at	Endo-1,3-beta-glucanase	3.34
Mtr.29332.1.S1_s_at	Hypothetical protein	3.34
Mtr.44448.1.S1_at	Poly(A)-binding protein	3.34
Mtr.51701.1.S1_s_at	Cyclin-like F-box protein	3.34
Mtr.39520.1.S1_at	Hypothetical protein	3.34
Mtr.19212.1.S1_at	Hypothetical protein	3.34
Mtr.39646.1.S1_at	Receptor-like protein kinase	3.34
Mtr.20124.1.S1_at	Serine/threonine protein kinase	3.34
Mtr.17399.1.S1_at	Peptidylprolyl isomerase, FKBP-type	3.34
Mtr.40132.1.S1_at	Peroxidase1	3.34
Mtr.21871.1.S1_at	Hypothetical protein	3.34

Mtr.34899.1.S1_at	Hexose carrier protein	3.34
Mtr.50207.1.S1_s_at	Hypothetical protein	3.33
Mtr.13244.1.S1_at	Hypothetical protein	3.33
Mtr.50554.1.S1_at	Hypothetical protein	3.33
Mtr.10425.1.S1_at	Ethylene responsive element binding factor	3.33
Mtr.34874.1.S1_s_at	Dolichyl-phosphate beta-glucosyltransferase	3.33
Mtr.37773.1.S1_at	GDSL-motif lipase/hydrolase-like protein	3.33
Mtr.10867.1.S1_at	Expansin-like protein	3.33
Mtr.10633.1.S1_at	Hypothetical protein	3.32
Mtr.9389.1.S1_at	DNA-binding protein RAV1	3.32
Mtr.27919.1.S1_at	Hypothetical protein	3.32
Mtr.49246.1.S1_at	Zn-finger, MYND type	3.32
Mtr.17691.1.S1_at	Transcriptional factor	3.32
Mtr.29056.1.S1_at	Hypothetical protein	3.32
Mtr.42872.1.S1_at	Hypothetical protein	3.32
Msa.3067.1.S1_at	Hypothetical protein	3.32
Mtr.13557.1.S1_at	Ethylene receptor homolog	3.32
Mtr.17704.1.S1_s_at	Hypothetical protein	3.32
Mtr.13913.1.S1_at	Phytochelatin synthetase-like protein	3.32
Mtr.19330.1.S1_at	Peptidase A1	3.32
Mtr.7084.1.S1_at	Asparagine synthase	3.31
Mtr.33541.1.S1_x_at	Asparagine synthase	3.31
Mtr.7575.1.S1_at	Hypothetical protein	3.31
Mtr.12111.1.S1_s_at	Enhancer of split complex m3 protein	3.31
Mtr.36964.1.S1_at	Hypothetical protein	3.31
Mtr.47588.1.S1_x_at	Hypothetical protein	3.31
Mtr.5072.1.S1_s_at	Hypothetical protein	3.31
Msa.2711.1.S1_at	Hypothetical protein	3.31
Mtr.8944.1.S1_at	Chorismate mutase	3.31
Mtr.16956.1.S1_at	O-methyltransferase	3.31
Mtr.9744.1.S1_at	Hypothetical protein	3.31
Mtr.38709.1.S1_at	Hypothetical protein	3.31
Mtr.14280.1.S1_at	Thioredoxin-related protein	3.30
Mtr.42544.1.S1_at	Nodulin-like protein	3.30
Mtr.5760.1.S1_at	Hypothetical protein	3.30
Mtr.40961.1.S1_at	Hypothetical protein	3.30
Mtr.37730.1.S1_at	Golgi SNARE 11 protein	3.30
Mtr.26632.1.S1_at	Hypothetical protein	3.30
Mtr.17625.1.S1_at	Zn-finger, RING	3.30
Mtr.35346.1.S1_at	Hypothetical protein	3.29
Mtr.40110.1.S1_at	Ribosomal protein S27	3.29
Mtr.13009.1.S1_s_at	Hypothetical protein	3.29
Mtr.39867.1.S1_at	Hypothetical protein	3.29
Mtr.12817.1.S1_at	Hypothetical protein	3.29
Mtr.41306.1.S1_s_at	Hypothetical protein	3.29
Mtr.33563.1.S1_at	Hypothetical protein	3.28
Mtr.43198.1.S1_at	RNA helicase	3.28
Mtr.9362.1.S1_at	Hypothetical protein	3.28
Mtr.15849.1.S1_at	Glycine-rich RNA binding protein	3.28
Mtr.43483.1.S1_at	Hypothetical protein	3.28
Mtr.28563.1.S1_at	Hypothetical protein	3.28
Mtr.15535.1.S1_at	Serine/threonine protein kinase	3.28
Mtr.16912.1.S1_at	WD-40 repeat protein	3.28
Mtr.50185.1.S1_at	Hypothetical protein	3.28

Mtr.43667.1.S1_at	GAST1 protein homolog	3.28
Mtr.39750.1.S1_at	Hypothetical protein	3.28
Mtr.5980.1.S1_at	Hypothetical protein	3.28
Mtr.12771.1.S1_at	Pti1 kinase-like protein	3.28
Mtr.10027.1.S1_at	Hypothetical protein	3.27
Mtr.11991.1.S1_at	Strubbelig receptor family	3.27
Mtr.39440.1.S1_at	Serine/threonine kinase	3.27
Mtr.40050.1.S1_at	Rab escort protein	3.27
Mtr.8259.1.S1_at	Hypothetical protein	3.27
Mtr.18898.1.S1_at	WD-40 repeat	3.27
Mtr.32032.1.S1_at	Extensin-like protein	3.27
Mtr.51599.1.S1_s_at	Serine/threonine protein kinase	3.27
Mtr.51695.1.S1_at	Hypothetical protein	3.27
Mtr.39020.1.S1_at	Ethylene receptor homolog	3.27
Mtr.2685.1.S1_at	Hypothetical protein	3.27
Mtr.21241.1.S1_at	Ribosomal protein L15e	3.27
Mtr.32820.1.S1_s_at	Glucan 1,3-beta-glucosidase	3.27
Mtr.19752.1.S1_at	Hypothetical protein	3.26
Mtr.34834.1.S1_at	Serine/threonine protein kinase	3.26
Mtr.38725.1.S1_s_at	CDPK adapter protein	3.26
Mtr.11064.1.S1_at	DNA-directed RNA polymerase	3.26
Mtr.1174.1.S1_at	Hypothetical protein	3.26
Mtr.43436.1.S1_at	Pantothenate kinase 1	3.26
Mtr.44964.1.S1_at	Hypothetical protein	3.26
Msa.591.1.S1_at	Hypothetical protein	3.26
Mtr.40320.1.S1_at	Hypothetical protein	3.26
Mtr.15443.1.S1_at	Hypothetical protein	3.26
Mtr.41656.1.S1_at	Hypothetical protein	3.26
Mtr.49556.1.S1_s_at	RNA polymerase Rpb1	3.26
Mtr.16204.1.S1_at	Hypothetical protein	3.26
Mtr.43171.1.S1_at	Transcription factor WRKY4	3.25
Mtr.42452.1.S1_at	Hypothetical protein	3.25
Mtr.42375.1.S1_at	Hypothetical protein	3.25
Mtr.13714.1.S1_at	Auxin-induced protein	3.25
Mtr.12959.1.S1_s_at	Auxin response factor	3.25
Mtr.34929.1.S1_at	Zinc finger protein CONSTANS-LIKE	3.25
Mtr.25598.1.S1_at	Delta24-sterol-C-methyltransferase	3.25
Mtr.7519.1.S1_at	IFA-binding protein-like	3.25
Mtr.5080.1.S1_s_at	Extensin (Class I)	3.25
Mtr.377.1.S1_s_at	Hypothetical protein	3.25
Mtr.36790.1.S1_at	Aconitate hydratase	3.25
Mtr.41977.1.S1_at	Hypothetical protein	3.25
Mtr.44989.1.S1_at	Nucleosome assembly protein	3.25
Mtr.39546.1.S1_at	Hypothetical protein	3.25
Mtr.41497.1.S1_at	ARIADNE-like protein	3.24
Mtr.20117.1.S1_at	Peptidyl-prolyl cis-trans isomerase, cyclophilin type	3.24
Mtr.38368.1.S1_at	Hypothetical protein	3.24
Mtr.1871.1.S1_at	Maturase K	3.24
Mtr.50044.1.S1_at	Thioredoxin-related protein	3.23
Mtr.9994.1.S1_at	Hypothetical protein	3.23
Mtr.24721.1.S1_at	Heat shock transcription factor	3.23
Mtr.40786.1.S1_at	40S ribosomal protein S25-1	3.23
Msa.690.1.S1_at	Hypothetical protein	3.23
Mtr.10478.1.S1_at	Cytoplasmic ribosomal protein S15a	3.23

Mtr.43020.1.S1_at	Fructokinase	3.23
Mtr.10164.1.S1_at	Adenylate kinase	3.23
Mtr.51785.1.S1_at	Hypothetical protein	3.23
Mtr.9221.1.S1_at	Salicylic acid glucosyltransferase	3.23
Mtr.37244.1.S1_at	Hypothetical protein	3.22
Mtr.22264.1.S1_at	Phosphoenolpyruvate/phosphate translocator	3.22
Mtr.5578.1.S1_at	Receptor protein kinase	3.22
Mtr.28125.1.S1_s_at	Early nodulin GRP3	3.22
Mtr.11333.1.S1_at	Oxidase-like protein	3.22
Mtr.17858.1.S1_at	Hypothetical protein	3.22
Mtr.24441.1.S1_s_at	Hypothetical protein	3.22
Mtr.13621.1.S1_s_at	Multidrug-resistance related protein	3.21
Mtr.12656.1.S1_at	NADPH:quinone oxidoreductase	3.21
Mtr.9098.1.S1_at	CREG2-protein-like	3.21
Mtr.691.1.S1_at	Hypothetical protein	3.21
Mtr.14439.1.S1_at	Hypothetical protein	3.21
Mtr.8914.1.S1_s_at	SER/THR PROTEIN PHOSPHATASE PP2C HOMOLOG	3.21
Mtr.50247.1.S1_at	Sugar transporter	3.21
Mtr.14934.1.S1_s_at	Gly rich structural protein	3.21
Mtr.46455.1.S1_at	Hypothetical protein	3.21
Mtr.40773.1.S1_at	Hypothetical protein	3.21
Mtr.12514.1.S1_at	NAM-like protein	3.21
Mtr.2071.1.S1_at	Hypothetical protein	3.21
Mtr.39950.1.S1_at	Hypothetical protein	3.20
Mtr.37584.1.S1_at	Hypothetical protein	3.20
Mtr.15716.1.S1_at	WD40	3.20
Mtr.9984.1.S1_at	Acyl-activating enzyme	3.20
Mtr.174.1.S1_at	Response regulator protein	3.20
Mtr.41281.1.S1_s_at	Hypothetical protein	3.20
Mtr.42058.1.S1_s_at	Hypothetical protein	3.20
Mtr.45221.1.S1_at	Hypothetical protein	3.20
Mtr.5567.1.S1_at	Hypothetical protein	3.20
Mtr.41934.1.S1_at	Pupal cuticle protein	3.19
Mtr.4597.1.S1_at	Hypothetical protein	3.19
Mtr.8783.1.S1_at	RNA helicase	3.19
Mtr.44464.1.S1_at	Elongation factor G	3.19
Msa.1311.1.S1_at	Hypothetical protein	3.19
Mtr.42889.1.S1_s_at	Elongation factor EF-2	3.18
Mtr.50746.1.S1_at	Staphylococcus nuclease	3.18
Mtr.19050.1.S1_at	Mitochondrial and plastid RNA polymerase	3.18
Mtr.33699.1.S1_at	Trehalose-6-phosphate synthase like protein	3.18
Mtr.12426.1.S1_at	Dynamin-like protein	3.18
Mtr.44597.1.S1_at	Female-specific transformer protein	3.18
Mtr.35761.1.S1_at	Hypothetical protein	3.18
Mtr.13525.1.S1_at	Hypothetical protein	3.18
Mtr.23686.1.S1_s_at	DNA-directed RNA polymerase subunit	3.17
Mtr.42739.1.S1_at	Protein kinase	3.17
Mtr.17255.1.S1_at	Formyl transferase	3.17
Mtr.44281.1.S1_at	Purple acid phosphatase	3.17
Mtr.32287.1.S1_at	Hypothetical protein	3.17
Mtr.38541.1.S1_at	Receptor-like protein kinase	3.17
Mtr.40416.1.S1_at	Hypothetical protein	3.17
Mtr.42380.1.S1_at	Hypothetical protein	3.17
Mtr.32884.1.S1_s_at	Jasmonic acid 2	3.17

Msa.1342.1.S1_at	Hypothetical protein	3.16
Mtr.12750.1.S1_at	Hypothetical protein	3.16
Mtr.47814.1.S1_at	Hypothetical protein	3.16
Mtr.20243.1.S1_s_at	Hypothetical protein	3.16
Mtr.21478.1.S1_s_at	Hypothetical protein	3.16
Mtr.10714.1.S1_at	Receptor protein kinase	3.16
Mtr.14517.1.S1_at	RNA-binding protein	3.16
Mtr.41454.1.S1_at	Farnesylated protein	3.16
Mtr.33283.1.S1_at	Chitinase	3.16
Mtr.32363.1.S1_at	Phosphatidylinositol/phosphatidylcholine transfer protein	3.16
Mtr.11679.1.S1_at	Hypothetical protein	3.16
Mtr.12784.1.S1_at	Wound-induced GSK-3-like protein	3.15
Mtr.205.1.S1_at	40S ribosomal protein S12	3.15
Mtr.10443.1.S1_at	Glyceraldehyde-3-phosphate dehydrogenase	3.15
Mtr.39338.1.S1_at	DNA-directed RNA polymerase I	3.15
Mtr.9647.1.S1_at	Hypothetical protein	3.15
Mtr.16955.1.S1_at	Ribosomal protein S9	3.15
Msa.1303.1.S1_at	Hypothetical protein	3.15
Mtr.44867.1.S1_at	1,3-fucosyltransferase	3.15
Mtr.5213.1.S1_at	TPR-repeat-containing proteins	3.15
Msa.1271.1.S1_at	Hypothetical protein	3.15
Mtr.45883.1.S1_at	Hypothetical protein	3.15
Mtr.11604.1.S1_s_at	Hypothetical protein	3.14
Mtr.43074.1.S1_at	RPM1-interacting protein	3.14
Mtr.9510.1.S1_at	Proliferating-cell nucleolar protein	3.14
Mtr.13558.1.S1_at	Beta-galactosidase	3.14
Mtr.50504.1.S1_at	Protein kinase	3.14
Mtr.6498.1.S1_at	Hypothetical protein	3.14
Mtr.20508.1.S1_s_at	Pyridine nucleotide-disulphide oxidoreductase, class-II	3.14
Mtr.5392.1.S1_at	Hypothetical protein	3.14
Mtr.45171.1.S1_at	Mitochondrial and chloroplast RNA polymerase	3.14
Mtr.38928.1.S1_at	Choline transporter-like protein	3.14
Mtr.31625.1.S1_at	Receptor kinase	3.14
Mtr.41177.1.S1_at	Mitochondrial carrier protein	3.14
Mtr.38213.1.S1_at	Hypothetical protein	3.14
Mtr.51323.1.S1_s_at	Hypothetical protein	3.14
Mtr.4347.1.S1_s_at	Chalcone-flavonone isomerase	3.13
Mtr.8407.1.S1_at	Hypothetical protein	3.13
Msa.2750.1.S1_at	Hypothetical protein	3.13
Mtr.10349.1.S1_at	Hypothetical protein	3.13
Mtr.33159.1.S1_at	Hypothetical protein	3.13
Msa.1290.1.S1_at	Hypothetical protein	3.13
Mtr.22966.1.S1_s_at	Hypothetical protein	3.13
Mtr.12961.1.S1_at	Hypothetical protein	3.13
Mtr.35231.1.S1_at	Pathogenesis-related protein	3.13
Msa.1965.1.S1_at	Hypothetical protein	3.13
Mtr.6664.1.S1_at	Hypothetical protein	3.13
Mtr.10583.1.S1_at	Root-specific metal transporter	3.13
Mtr.34637.1.S1_at	Anthranilate N-benzoyltransferase-like protein	3.13
Mtr.41732.1.S1_at	Hypothetical protein	3.13
Mtr.51829.1.S1_s_at	Response regulator receiver; CheY-like protein	3.13
Msa.1218.1.S1_at	Hypothetical protein	3.13
Mtr.51636.1.S1_at	Hypothetical protein	3.13
Mtr.20237.1.S1_at	Mitochondrial carrier protein	3.13

Mtr.5863.1.S1_at	Isoflavonoid glucosyltransferase	3.13
Mtr.41634.1.S1_at	Receptor kinase	3.12
Mtr.35571.1.S1_at	Hypothetical protein	3.12
Mtr.41848.1.S1_at	Acyl-CoA oxidase	3.12
Mtr.44471.1.S1_at	NADH dehydrogenase	3.12
Mtr.12154.1.S1_at	Hypothetical protein	3.12
Mtr.4816.1.S1_at	CDPK-like protein	3.12
Mtr.19348.1.S1_x_at	Hypothetical protein	3.12
Mtr.15650.1.S1_at	Adenylate kinase	3.12
Mtr.16016.1.S1_at	Hypothetical protein	3.12
Mtr.14274.1.S1_at	RNA-binding protein	3.12
Mtr.13716.1.S1_at	Hypothetical protein	3.11
Mtr.29305.1.S1_at	Hypothetical protein	3.11
Mtr.40007.1.S1_at	Hypothetical protein	3.11
Mtr.50358.1.S1_at	Hypothetical protein	3.11
Mtr.22834.1.S1_at	Symbiotic ammonium transporter	3.11
Mtr.29620.1.S1_at	Hypothetical protein	3.11
Mtr.39981.1.S1_at	CTR1-like protein kinase	3.11
Mtr.41029.1.S1_at	Ankyrin repeat-containing protein	3.11
Mtr.12917.1.S1_at	Jasmonic acid 2	3.11
Mtr.37122.1.S1_at	Hypothetical protein	3.11
Mtr.3288.1.S1_at	Hypothetical protein	3.11
Mtr.29969.1.S1_at	Hypothetical protein	3.11
Mtr.9032.1.S1_at	Aspartyl protease	3.11
Mtr.39985.1.S1_at	Hypothetical protein	3.11
Mtr.2503.1.S1_s_at	NEP1-interacting protein	3.11
Mtr.13113.1.S1_at	Hypothetical protein	3.11
Mtr.1887.1.S1_at	Gibberellin oxidase-like protein	3.10
Mtr.39710.1.S1_at	Hypothetical protein	3.10
Mtr.43389.1.S1_at	Fiber protein Fb2	3.10
Mtr.5582.1.S1_s_at	DNA-directed RNA polymerase I	3.10
Mtr.5620.1.S1_at	Membrane cofactor protein	3.10
Mtr.37421.1.S1_at	Somatic embryogenesis receptor-like kinase	3.10
Mtr.12392.1.S1_at	Phaseolin G-box binding protein	3.10
Mtr.8594.1.S1_at	Soluble inorganic pyrophosphatase	3.10
Mtr.46085.1.S1_at	Homeodomain-like protein	3.10
Mtr.8716.1.S1_at	Ras-related protein	3.10
Mtr.21025.1.S1_at	Alpha/beta hydrolase	3.09
Mtr.43511.1.S1_at	Nucleic acid binding protein	3.09
Mtr.36679.1.S1_s_at	Hypothetical protein	3.09
Mtr.49129.1.S1_s_at	Puf RNA-binding protein	3.09
Mtr.1692.1.S1_at	Hypothetical protein	3.09
Mtr.41430.1.S1_at	Hypothetical protein	3.09
Mtr.41930.1.S1_at	Hypothetical protein	3.09
Mtr.37423.1.S1_at	Chaperonin CPN60-2, mitochondrial precursor (HSP60-2)	3.09
Mtr.16619.1.S1_at	Ribosomal protein L24E	3.09
Mtr.35606.1.S1_at	Hypothetical protein	3.09
Mtr.6798.1.S1_at	NADH dehydrogenase subunit 5	3.09
Mtr.24341.1.S1_at	Hypothetical protein	3.09
Mtr.5840.1.S1_at	Hypothetical protein	3.09
Mtr.50730.1.S1_at	bZIP transcription factor	3.09
Mtr.48949.1.S1_at	Biopterin transport-related protein	3.09
Mtr.40474.1.S1_at	Ribosomal protein 30S	3.09
Mtr.43306.1.S1_at	Nucleic acid binding protein	3.09

Mtr.31452.1.S1_at	Hypothetical protein	3.08
Msa.1102.1.S1_at	Hypothetical protein	3.08
Mtr.41303.1.S1_at	Hypothetical protein	3.08
Mtr.40130.1.S1_at	Peroxidase	3.08
Mtr.28321.1.S1_s_at	Phloem-specific lectin PP2-like protein	3.08
Mtr.20487.1.S1_at	Hypothetical protein	3.08
Mtr.8495.1.S1_at	Endoplasmic reticulum HSC70-cognate binding protein	3.08
Mtr.42021.1.S1_at	1-aminocyclopropane-1-carboxylate oxidase	3.08
Mtr.37956.1.S1_at	Hypothetical protein	3.08
Mtr.33608.1.S1_at	Hypothetical protein	3.08
Mtr.11602.1.S1_at	Hypothetical protein	3.08
Mtr.28049.1.S1_at	Hypothetical protein	3.08
Mtr.9213.1.S1_at	NIMA-related protein kinase	3.07
Mtr.33201.1.S1_at	Hypothetical protein	3.07
Mtr.34946.1.S1_at	Hypothetical protein	3.07
Mtr.13773.1.S1_at	NADH dehydrogenase subunit 4	3.07
Mtr.44333.1.S1_s_at	Hypothetical protein	3.07
Mtr.41152.1.S1_at	Hypothetical protein	3.07
Mtr.43544.1.S1_at	Covalently-linked cell wall protein 7	3.07
Mtr.8462.1.S1_at	Lipoxygenase	3.07
Mtr.1767.1.S1_at	Hypothetical protein	3.07
Mtr.41578.1.S1_at	Hypothetical protein	3.07
Mtr.12053.1.S1_at	Serine acetyltransferase	3.07
Mtr.374.1.S1_at	Hypothetical protein	3.07
Mtr.38304.1.S1_s_at	Hypothetical protein	3.07
Mtr.4344.1.S1_at	Rapid alkalinization factor 2	3.07
Mtr.44885.1.S1_at	Hypothetical protein	3.07
Mtr.26965.1.S1_at	Hypothetical protein	3.07
Mtr.43865.1.S1_at	Heat shock protein 83	3.07
Mtr.50494.1.S1_s_at	Hypothetical protein	3.07
Msa.2793.1.S1_at	Hypothetical protein	3.07
Mtr.23204.1.S1_x_at	Hypothetical protein	3.06
Msa.2808.1.S1_at	Hypothetical protein	3.06
Mtr.10744.1.S1_at	Hypothetical protein	3.06
Mtr.32827.1.S1_s_at	Hypothetical protein	3.06
Mtr.24032.1.S1_at	Hypothetical protein	3.06
Mtr.13834.1.S1_at	Hypothetical protein	3.06
Mtr.14845.1.S1_s_at	Hypothetical protein	3.06
Mtr.45351.1.S1_s_at	Hypothetical protein	3.06
Mtr.13646.1.S1_at	Hypothetical protein	3.06
Mtr.43847.1.S1_at	Mitochondrial carrier protein	3.06
Mtr.12230.1.S1_s_at	Elongation factor 2	3.06
Mtr.21300.1.S1_at	Small nuclear-like ribonucleoprotein	3.05
Mtr.36034.1.S1_at	Hypothetical protein	3.05
Mtr.50766.1.S1_at	Hypothetical protein	3.05
Mtr.9417.1.S1_at	Beta-galactosidase	3.05
Mtr.6343.1.S1_at	Hydroxyproline-rich glycoprotein	3.05
Mtr.28513.1.S1_at	Phospholipase D	3.05
Mtr.11078.1.S1_at	Hypothetical protein	3.05
Mtr.6418.1.S1_s_at	Hypothetical protein	3.05
Mtr.40782.1.S1_at	Hypothetical protein	3.05
Mtr.43936.1.S1_at	Ser/Thr protein kinase	3.05
Mtr.9031.1.S1_s_at	Nucellin-like aspartic protease	3.05
Mtr.28286.1.S1_at	Hypothetical protein	3.05

Mtr.42839.1.S1_at	Hypothetical protein	3.05
Mtr.50416.1.S1_at	Phosphatidylinositol 3- and 4-kinase	3.05
Mtr.27318.1.S1_at	Fructose-bisphosphate aldolase	3.05
Mtr.42528.1.S1_at	Hypothetical protein	3.04
Mtr.2386.1.S1_at	Hypothetical protein	3.04
Mtr.10736.1.S1_at	Hypothetical protein	3.04
Mtr.28378.1.S1_at	Hypothetical protein	3.04
Mtr.15376.1.S1_at	Hypothetical protein	3.04
Mtr.19399.1.S1_at	Glutathione S-transferase	3.04
Mtr.52344.1.S1_at	Aminotransferase	3.04
Mtr.32578.1.S1_at	Hypothetical protein	3.04
Mtr.38635.1.S1_at	Peroxidase	3.04
Mtr.43265.1.S1_at	Hypothetical protein	3.04
Mtr.45123.1.S1_at	Hypothetical protein	3.04
Mtr.41453.1.S1_at	Hypothetical protein	3.04
Mtr.44587.1.S1_at	Peroxisomal acetoacetyl-coenzyme A thiolase	3.04
Mtr.38970.1.S1_at	Hypothetical protein	3.04
Mtr.51932.1.S1_at	Quinonprotein alcohol dehydrogenase	3.04
Mtr.30728.1.S1_at	SAR DNA-binding protein	3.04
Mtr.5045.1.S1_at	Hypothetical protein	3.04
Mtr.1893.1.S1_at	Hypothetical protein	3.03
Mtr.46098.1.S1_s_at	Zn-finger, DHHC type	3.03
Mtr.23743.1.S1_at	Hypothetical protein	3.03
Msa.1037.1.S1_at	Hypothetical protein	3.03
Mtr.25749.1.S1_at	Copper amine oxidase	3.03
Mtr.6671.1.S1_at	Hypothetical protein	3.03
Mtr.43151.1.S1_at	Beta-galactosidase	3.03
Mtr.43129.1.S1_at	Arginine decarboxylase	3.02
Mtr.25309.1.S1_at	Hypothetical protein	3.02
Mtr.33064.1.S1_at	Calmodulin-binding protein	3.02
Mtr.31446.1.S1_at	Hypothetical protein	3.02
Mtr.13453.1.S1_at	Hypothetical protein	3.02
Mtr.38855.1.S1_at	Hypothetical protein	3.02
Msa.1686.1.S1_s_at	Hypothetical protein	3.02
Mtr.33034.1.S1_at	Chromobox protein	3.02
Mtr.9077.1.S1_at	Hypothetical protein	3.02
Msa.277.1.S1_at	Hypothetical protein	3.02
Mtr.8479.1.S1_at	Cytochrome P450	3.02
Mtr.19346.1.S1_s_at	Hypothetical protein	3.02
Mtr.4284.1.S1_at	Hypothetical protein	3.02
Mtr.40928.1.S1_at	PGPD14 protein	3.02
Mtr.51291.1.S1_at	Pathogenesis-related transcriptional factor	3.02
Mtr.41492.1.S1_at	Phytochrome-associated protein	3.01
Mtr.42907.1.S1_at	Hypothetical protein	3.01
Mtr.29453.1.S1_at	Hypothetical protein	3.01
Mtr.27737.1.S1_at	Hypothetical protein	3.01
Mtr.27653.1.S1_s_at	Hypothetical protein	3.01
Mtr.2692.1.S1_at	Hypothetical protein	3.01
Mtr.11774.1.S1_at	Hypothetical protein	3.01
Mtr.43348.1.S1_at	Isoprenylated protein	3.01
Mtr.43493.1.S1_at	Hypothetical protein	3.01
Mtr.27102.1.S1_at	Hypothetical protein	3.01
Mtr.28295.1.S1_s_at	Female-specific transformer protein	3.01
Mtr.12018.1.S1_at	Hypothetical protein	3.01

Mtr.50704.1.S1_at	Aldehyde dehydrogenase	3.01
Mtr.23789.1.S1_s_at	Glutamate decarboxylase	3.01
Mtr.32877.1.S1_at	Hypothetical protein	3.01
Msa.1002.1.S1_at	Hypothetical protein	3.00
Mtr.12120.1.S1_at	Hypothetical protein	3.00
Mtr.10838.1.S1_at	Leucine-rich repeat protein	3.00

Supplementary Table S2. Downregulated genes at 12 hours after inoculation with soybean rust

Probesets	Target Description	Fold reduction
Mtr.26465.1.S1_s_at	Phosphoethanolamine N-methyltransferase	-886.26
Mtr.13982.1.S1_at	CDP-diacylglycerol-glycerol-3-phosphate 3-phosphatidyltransferase	-687.90
Mtr.35794.1.S1_s_at	Myo-inositol 1-phoshate synthase	-659.64
Mtr.20438.1.S1_at	O-methyltransferase	-639.88
Mtr.20321.1.S1_at	Plant lipid transfer/seed storage/trypsin-alpha amylase inhibitor	-629.87
Mtr.42849.1.S1_at	Myo-inositol-1-phosphate synthase	-520.47
Mtr.8427.1.S1_at	Lipoxygenase	-469.91
Mtr.12069.1.S1_at	Phosphoethanolamine N-methyltransferase	-363.98
Msa.888.1.S1_at	Hypothetical protein	-339.34
Mtr.11085.1.S1_at	LHY protein	-329.74
Mtr.8618.1.S1_at	Cytochrome P450 monooxygenase CYP93D1	-328.21
Mtr.43021.1.S1_at	1-deoxy-D-xylulose 5-phosphate reductoisomerase	-325.67
Msa.2715.1.S1_at	Hypothetical protein	-268.19
Mtr.44581.1.S1_at	Hypothetical protein	-244.04
Mtr.10456.1.S1_at	Early light inducible protein	-213.77
Mtr.12499.1.S1_s_at	Hypothetical protein	-208.32
Mtr.40245.1.S1_s_at	Early light inducible protein	-206.93
Mtr.12393.1.S1_at	Cinnamoyl-CoA reductase	-204.37
Mtr.44731.1.S1_at	Hypothetical protein	-201.56
Mtr.38552.1.S1_s_at	Hypothetical protein	-199.56
Mtr.10369.1.S1_at	Chalcone synthase	-188.66
Mtr.15442.1.S1_at	Hypothetical protein	-188.28
Mtr.8651.1.S1_a_at	Dehydrin-like protein	-173.38
Mtr.773.1.S1_s_at	LHY protein	-169.80
Msa.888.1.S1_s_at	Hypothetical protein	-167.82
Mtr.16402.1.S1_at	NADPH HC toxin reductase	-167.17
Mtr.12272.1.S1_at	Granule-bound glycogen (Starch) synthase	-166.78
Mtr.8651.1.S1_at	Dehydrin-like protein	-166.21
Mtr.42858.1.S1_at	Acid phosphatase	-163.28
Mtr.9320.1.S1_at	Hypothetical protein	-162.56
Mtr.42982.1.S1_s_at	Carbonic anhydrase	-161.97
Mtr.52215.1.S1_at	Lipoxygenase	-159.17
Mtr.10457.1.S1_at	Early light inducible protein	-155.94
Mtr.10659.1.S1_s_at	Hypothetical protein	-152.50
Mtr.26601.1.S1_s_at	FRO1-like protein	-150.56
Mtr.52315.1.S1_at	Heat shock protein DnaJ	-138.14
Mtr.38073.1.S1_at	Dihydroflavanol-4-reductase 1	-134.50
Mtr.44812.1.S1_s_at	Extracellular calcium sensing receptor	-131.73
Msa.3134.1.S1_at	Hypothetical protein	-131.02
Mtr.14290.1.S1_at	Rhodanese-like protein	-130.84
Mtr.43089.1.S1_at	Seed maturation protein LEA 4	-130.56
Mtr.10450.1.S1_at	Hypothetical protein	-130.13
Mtr.46164.1.S1_s_at	Hypothetical protein	-127.64
Mtr.51205.1.S1_at	Hypothetical protein	-125.87
Mtr.4439.1.S1_s_at	Thioredoxin H2	-125.15
Mtr.26602.1.S1_s_at	NADPH oxidase	-121.40
Msa.3155.1.S1_at	Hypothetical protein	-120.72
Mtr.43870.1.S1_at	NADPH oxidase	-118.73
Mtr.46609.1.S1_at	UDP-glycosyltransferase	-116.66
Mtr.11110.1.S1_at	GCN5-related N-acetyltransferase (GNAT) family	-116.56
Mtr.768.1.S1_at	Hypothetical protein	-111.46
Mtr.28774.1.S1_at	Anthocyanidin synthase	-111.08

Mtr.12687.1.S1_at	Hypothetical protein	-107.69
Mtr.13564.1.S1_s_at	Extracellular calcium sensing receptor	-106.98
Mtr.45133.1.S1_at	Amine oxidase	-105.86
Msa.1858.1.S1_at	Hypothetical protein	-105.24
Mtr.35685.1.S1_at	Heat shock protein	-105.09
Mtr.12942.1.S1_at	Late embryogenesis abundant protein	-104.14
Mtr.37221.1.S1_at	Photosystem II type I chlorophyll a/b-binding protein	-103.58
Mtr.13397.1.S1_at	Regulator of chromosome condensation-like protein	-103.53
Mtr.50943.1.S1_at	Peptidase	-103.51
Mtr.32878.1.S1_at	Zeaxanthin epoxidase	-103.43
Msa.3125.1.S1_at	Hypothetical protein	-102.00
Mtr.40719.1.S1_s_at	Tic62 protein	-101.44
Mtr.1186.1.S1_s_at	Hypothetical protein	-100.32
Mtr.16410.1.S1_at	Alpha/beta hydrolase	-98.49
Mtr.13375.1.S1_at	Hypothetical protein	-96.56
Mtr.6142.1.S1_at	Jasmonate O-methyltransferase	-95.18
Mtr.32797.1.S1_s_at	Sphingolipid long chain base delta 8 desaturase	-95.02
Mtr.11284.1.S1_at	Oxygen evolving complex protein	-94.72
Mtr.27879.1.S1_at	Hypothetical protein	-93.52
Mtr.31194.1.S1_at	Cytochrome P450	-92.98
Mtr.26044.1.S1_s_at	Circadian clock associated1	-92.09
Msa.1358.1.S1_at	Hypothetical protein	-90.45
Mtr.26465.1.S1_at	Phosphoethanolamine N-methyltransferase	-89.27
Mtr.13014.1.S1_at	Amine oxidase	-89.20
Mtr.10693.1.S1_at	Cytochrome P450	-87.45
Mtr.35696.1.S1_s_at	Hypothetical protein	-87.40
Mtr.19759.1.S1_at	Zinc-containing alcohol dehydrogenase	-87.05
Mtr.48649.1.S1_at	Cellulose synthase	-86.86
Mtr.32302.1.S1_at	Hypothetical protein	-86.68
Msa.3055.1.S1_at	Hypothetical protein	-85.57
Mtr.37252.1.S1_at	Salt-tolerance protein	-85.29
Mtr.44844.1.S1_at	Chalcone reductase	-84.93
Msa.2980.1.S1_at	Hypothetical protein	-84.83
Mtr.9873.1.S1_at	Thioredoxin H2	-84.73
Mtr.41960.1.S1_at	Hypothetical protein	-83.91
Mtr.39986.1.S1_at	Hypothetical protein	-83.69
Mtr.43296.1.S1_at	Hypothetical protein	-83.56
Mtr.41063.1.S1_at	Nine-cis-epoxycarotenoid dioxygenase4	-83.49
Mtr.38344.1.S1_at	Limonoid UDP-glucosyltransferase	-83.28
Mtr.38769.1.S1_at	Steroid 5alpha-reductase-like protein	-82.98
Mtr.47151.1.S1_at	Glutathione S-transferase	-82.72
Mtr.26597.1.S1_at	Hypothetical protein	-82.01
Mtr.41445.1.S1_at	Hypothetical protein	-81.97
Mtr.8771.1.S1_at	Hypothetical protein	-80.53
Mtr.50035.1.S1_at	Hypothetical protein	-80.37
Mtr.35647.1.S1_at	Hypothetical protein	-80.25
Mtr.9796.1.S1_at	Hypothetical protein	-79.62
Mtr.49244.1.S1_at	NADPH HC toxin reductase	-79.35
Mtr.33852.1.S1_at	CpABA1 protein	-79.16
Mtr.9735.1.S1_a_at	Tic62 protein	-78.96
Mtr.40546.1.S1_at	Hypothetical protein	-78.79
Mtr.7343.1.S1_at	ADP-glucose pyrophosphorylase	-78.45
Mtr.16941.1.S1_at	UDP-glucuronosyl/UDP-glucosyltransferase	-78.26
Mtr.41463.1.S1_at	ADP-glucose pyrophosphorylase	-76.89

Mtr.6707.1.S1_s_at	Hypothetical protein	-76.71
Mtr.40861.1.S1_at	Cytochrome P450	-75.25
Mtr.6048.1.S1_s_at	Hypothetical protein	-74.78
Mtr.12806.1.S1_at	Ascorbate oxidase promoter-binding protein	-74.29
Mtr.8438.1.S1_at	LHCII type I chlorophyll a/b-binding protein	-73.81
Mtr.42981.1.S1_s_at	Hypothetical protein	-73.75
Mtr.12797.1.S1_at	Family II lipase EXL3	-72.40
Mtr.37367.1.S1_at	UDP-glucosyltransferase	-72.23
Mtr.1857.1.S1_at	CpABA1 protein	-72.08
Mtr.37215.1.S1_at	Photosystem II type I chlorophyll a/b-binding protein	-71.98
Mtr.41828.1.S1_at	Thylakoid soluble phosphoprotein	-71.72
Mtr.41387.1.S1_at	Hypothetical protein	-71.63
Mtr.42902.1.S1_s_at	Hypothetical protein	-71.55
Mtr.2065.1.S1_at	Hypothetical protein	-70.87
Mtr.5956.1.S1_s_at	Late embryogenesis abundant protein	-70.49
Mtr.37363.1.S1_at	CPRD46 protein	-69.95
Mtr.37384.1.S1_at	Granule-bound starch synthase	-69.94
Mtr.12682.1.S1_at	Light-harvesting complex protein	-69.88
Mtr.41416.1.S1_at	Drought-induced protein RDI	-69.78
Mtr.34140.1.S1_at	CpABA1 protein	-69.30
Mtr.1274.1.S1_at	Proline-rich protein	-69.27
Mtr.11048.1.S1_at	Hypothetical protein	-68.83
Mtr.40306.1.S1_at	Hypothetical protein	-68.75
Mtr.35240.1.S1_s_at	Xyloglucan endotransglycosylase hydrolase 1	-68.73
Mtr.50430.1.S1_at	Lipoxygenase	-68.30
Msa.927.1.S1_at	Hypothetical protein	-68.24
Mtr.14755.1.S1_at	Hypothetical protein	-67.88
Mtr.26036.1.S1_s_at	Fatty acid elongase 3-ketoacyl-CoA synthase 1 (KCS1)	-67.54
Mtr.11088.1.S1_at	37kDa chloroplast inner envelope membrane polypeptide	-67.08
Mtr.13758.1.S1_at	Hypothetical protein	-65.84
Mtr.12327.1.S1_s_at	Late embryogenesis abundant protein	-65.60
Mtr.241.1.S1_at	Delta-6-desaturase	-65.58
Mtr.43118.1.S1_s_at	Cytochrome P450 72A1	-65.51
Mtr.39290.1.S1_at	Hypothetical protein	-65.50
Mtr.38596.1.S1_at	Amine oxidase	-65.39
Mtr.44988.1.S1_at	Zeaxanthin epoxidase	-64.58
Msa.1690.1.S1_at	Hypothetical protein	-63.94
Mtr.38772.1.S1_at	In2-1 protein	-63.80
Msa.1752.1.S1_s_at	Hypothetical protein	-63.58
Msa.2589.1.S1_at	Hypothetical protein	-62.53
Mtr.8650.1.S1_at	Xyloglucan endotransglycosylase hydrolase 1	-62.46
Mtr.44505.1.S1_at	Glucosyltransferase-9	-61.62
Mtr.8475.1.S1_s_at	Plastidic aldolase	-61.60
Msa.2924.1.S1_at	Hypothetical protein	-61.13
Msa.1578.1.S1_at	Hypothetical protein	-61.10
Mtr.42335.1.S1_at	Hypothetical protein	-61.05
Mtr.13564.1.S1_at	Extracellular calcium sensing receptor	-61.03
Mtr.2129.1.S1_at	Hypothetical protein	-61.01
Mtr.37362.1.S1_at	CPRD46 protein	-60.30
Mtr.253.1.S1_at	Symbiotic ammonium transporter	-59.84
Mtr.13261.1.S1_s_at	Hypothetical protein	-59.78
Mtr.33745.1.S1_s_at	Hypothetical protein	-59.78
Mtr.43886.1.S1_at	RNA polymerase sigma subunit SigE	-59.43
Mtr.43117.1.S1_at	Cytochrome P450	-59.39

Mtr.36069.1.S1_s_at	Hypothetical protein	-58.96
Mtr.18535.1.S1_at	Hypothetical protein	-57.76
Mtr.10604.1.S1_at	Nitrate reductase	-57.21
Mtr.19547.1.S1_s_at	Nucleoside phosphatase	-57.21
Mtr.13242.1.S1_at	Magnesium chelatase	-57.06
Mtr.51618.1.S1_at	Hypothetical protein	-56.50
Mtr.6031.1.S1_at	Lipid transfer protein	-56.38
Mtr.37368.1.S1_at	Hypothetical protein	-56.35
Mtr.12199.1.S1_at	Ascorbate oxidase promoter-binding protein	-55.61
Mtr.50943.1.S1_s_at	Peptidase	-55.20
Msa.2805.1.S1_at	Hypothetical protein	-55.16
Mtr.9236.1.S1_at	ADP-glucose pyrophosphorylase	-55.06
Mtr.41942.1.S1_at	Hypothetical protein	-54.70
Mtr.13484.1.S1_s_at	Golden2-like protein 2	-54.65
Mtr.23405.1.S1_at	Hypothetical protein	-54.52
Mtr.32384.1.S1_s_at	Beta-amyrin synthase	-54.50
Mtr.41393.1.S1_at	Hypothetical protein	-54.42
Mtr.43208.1.S1_at	Thioredoxin F-type	-53.95
Mtr.18630.1.S1_at	Prenyltransferase/squalene oxidase	-53.65
Mtr.10948.1.S1_at	Hypothetical protein	-53.36
Msa.1858.1.S1_x_at	Hypothetical protein	-53.35
Mtr.4951.1.S1_at	Hypothetical protein	-53.00
Mtr.34690.1.S1_at	Lactoylglutathione lyase	-52.99
Mtr.21070.1.S1_at	Hypothetical protein	-52.76
Mtr.2064.1.S1_at	Hypothetical protein	-52.53
Mtr.8554.1.S1_at	Fructose-bisphosphate aldolase	-51.97
Mtr.32679.1.S1_at	Hypothetical protein	-51.87
Msa.1381.1.S1_at	Hypothetical protein	-51.81
Msa.3003.1.S1_at	Hypothetical protein	-51.75
Mtr.14426.1.S1_at	Aldo/keto reductase	-51.48
Msa.1593.1.S1_at	Hypothetical protein	-51.17
Mtr.27305.1.S1_at	S-receptor kinase-like protein 2	-50.98
Msa.1451.1.S1_at	Hypothetical protein	-50.88
Mtr.36333.1.S1_at	Flavonoid 3'-hydroxylase	-50.72
Mtr.40497.1.S1_at	Glucose acyltransferase	-50.71
Mtr.8530.1.S1_s_at	Hypothetical protein	-50.61
Mtr.40432.1.S1_at	Sedoheptulose-1,7-bisphosphatase	-50.45
Mtr.48573.1.S1_at	Haloacid dehalogenase-like hydrolase	-50.26
Mtr.14608.1.S1_at	Heat shock protein Hsp20	-49.87
Mtr.31744.1.S1_at	Anthranilate N-hydroxycinnamoyl/benzoyltransferase	-49.61
Msa.1609.1.S1_at	Hypothetical protein	-49.37
Mtr.40669.1.S1_at	FK506 binding protein 1	-49.31
Mtr.34723.1.S1_at	37 kDa inner envelope membrane protein	-48.76
Mtr.34955.1.S1_at	Hypothetical protein	-48.59
Mtr.44591.1.S1_at	Quinone oxidoreductase	-48.58
Mtr.44991.1.S1_at	Cucumisin-like serine protease	-48.53
Mtr.15717.1.S1_at	Chlorophyll A-B binding protein	-48.26
Mtr.39190.1.S1_at	Cytochrome p450	-48.24
Mtr.43589.1.S1_at	Protease Do-like 1	-48.11
Mtr.5358.1.S1_at	Cellulose synthase	-47.87
Mtr.43911.1.S1_at	Hypothetical protein	-47.70
Mtr.22141.1.S1_at	Glycosyltransferase	-47.63
Mtr.9748.1.S1_at	Peptidyl-prolyl cis-trans isomerase TLP38	-47.62
Mtr.41042.1.S1_s_at	Phosphoethanolamine N-methyltransferase	-47.47

Mtr.49177.1.S1_at	Dienelactone hydrolase	-47.42
Mtr.36457.1.S1_at	RNA polymerase sigma subunit SigE	-47.40
Msa.3075.1.S1_at	Hypothetical protein	-47.28
Msa.1004.1.S1_at	Hypothetical protein	-46.87
Mtr.17090.1.S1_at	Myb, DNA-binding	-46.40
Mtr.3423.1.S1_at	Vesicular glutamate transporter-3	-46.36
Mtr.39853.1.S1_at	Basic PR-1 protein	-46.12
Mtr.5628.1.S1_s_at	Lipoxygenase	-45.95
Mtr.33633.1.S1_at	Subtilisin-like serine protease	-45.75
Mtr.18035.1.S1_at	Protein phosphatase 2C	-45.31
Mtr.8552.1.S1_at	Hypothetical protein	-44.99
Mtr.42226.1.S1_at	Ent-kaurenoic acid oxidase	-44.83
Mtr.9328.1.S1_at	Carboxyl terminal protease	-44.67
Mtr.6748.1.S1_at	Hypothetical protein	-44.34
Mtr.33820.1.S1_at	Beta-galactosidase	-44.13
Mtr.8476.1.S1_at	Plastidic aldolase	-44.10
Mtr.37557.1.S1_at	Nine-cis-epoxycarotenoid dioxygenase1	-43.93
Mtr.17317.1.S1_at	S-adenosyl-methionine-sterol-C-methyltransferase	-43.67
Msa.3154.1.S1_at	Hypothetical protein	-43.67
Mtr.44648.1.S1_at	Hypothetical protein	-43.52
Mtr.34220.1.S1_s_at	Hypothetical protein	-43.38
Mtr.8440.1.S1_at	Salt-tolerance protein	-43.37
Msa.1197.1.S1_at	Hypothetical protein	-43.35
Mtr.27898.1.S1_at	Alanine aminotransferase	-42.69
Mtr.9709.1.S1_at	Hypothetical protein	-42.16
Mtr.39524.1.S1_at	Circadian clock associated1	-42.12
Mtr.16620.1.S1_s_at	Phosphoglycerate/bisphosphoglycerate mutase	-42.04
Mtr.5348.1.S1_at	Hypothetical protein	-41.79
Mtr.11985.1.S1_at	Hypothetical protein	-41.62
Mtr.34856.1.S1_at	Hypothetical protein	-41.59
Mtr.41515.1.S1_at	Phosphate transporter	-41.46
Mtr.41843.1.S1_at	Fimbrin	-41.29
Mtr.46628.1.S1_s_at	Peroxidase	-41.10
Mtr.37533.1.S1_at	Fructose-1,6-bisphosphatase	-41.03
Mtr.22480.1.S1_s_at	Chloroplast mRNA-binding protein	-41.00
Mtr.6704.1.S1_at	Hypothetical protein	-40.71
Mtr.42665.1.S1_at	Hypothetical protein	-40.68
Mtr.31606.1.S1_s_at	Phosphoethanolamine N-methyltransferase	-40.51
Msa.1838.1.S1_s_at	Hypothetical protein	-40.33
Mtr.14013.1.S1_at	Transporter associated with antigen processing-like protein	-40.27
Mtr.40921.1.S1_at	Hypothetical protein	-40.23
Mtr.41715.1.S1_at	Receptor protein-like	-40.18
Mtr.37609.1.S1_s_at	Hypothetical protein	-40.10
Mtr.24578.1.S1_s_at	Hypothetical protein	-40.01
Mtr.9273.1.S1_at	Benzoyl coenzyme A	-39.49
Mtr.40742.1.S1_s_at	Thylakoid membrane phosphoprotein 14 kDa	-39.44
Mtr.50844.1.S1_at	PAP fibrillin	-39.34
Mtr.12138.1.S1_at	Hypothetical protein	-39.03
Mtr.9477.1.S1_at	Hypothetical protein	-39.00
Mtr.10124.1.S1_at	Cytochrome P450	-38.85
Mtr.24918.1.S1_s_at	Receptor-like protein kinase	-38.76
Mtr.34097.1.S1_at	Sucrose-phosphate synthase 2	-38.71
Mtr.37609.1.S1_at	Hypothetical protein	-38.66
Mtr.41305.1.S1_at	CPRD49 protein	-38.57

Mtr.43939.1.S1_s_at	Ribonuclease HII	-38.48
Msa.2951.1.S1_at	Hypothetical protein	-38.41
Mtr.10925.1.S1_at	Hypothetical protein	-38.35
Mtr.13190.1.S1_at	Hypothetical protein	-38.26
Mtr.50426.1.S1_at	Lipoxygenase	-38.23
Mtr.33651.1.S1_at	Hypothetical protein	-38.06
Mtr.5355.1.S1_at	Hypothetical protein	-38.04
Mtr.43717.1.S1_at	Hypothetical protein	-37.99
Mtr.6745.1.S1_s_at	Violaxanthin de-epoxidase	-37.95
Mtr.11810.1.S1_at	Hypothetical protein	-37.86
Mtr.46324.1.S1_at	Hypothetical protein	-37.56
Mtr.10830.1.S1_at	Solanesyl diphosphate synthase	-37.43
Mtr.1073.1.S1_at	Hypothetical protein	-37.38
Mtr.41786.1.S1_at	Hypothetical protein	-37.35
Mtr.44475.1.S1_at	Ultraviolet-B-repressible protein	-37.33
Mtr.8977.1.S1_at	Pathogenesis-related protein	-37.07
Mtr.10820.1.S1_at	Hypothetical protein	-36.87
Mtr.21910.1.S1_at	Trypsin protein inhibitor 2	-36.76
Mtr.32752.1.S1_s_at	Hypothetical protein	-36.73
Mtr.45311.1.S1_at	Hypothetical protein	-36.41
Mtr.11069.1.S1_at	Starch branching enzyme I	-36.20
Mtr.4723.1.S1_at	Hypothetical protein	-36.18
Mtr.40848.1.S1_at	Photosystem II protein X	-35.91
Mtr.42241.1.S1_at	Nodulin 26-like protein	-35.84
Mtr.41360.1.S1_at	Fimbrin-like protein	-35.65
Msa.1383.1.S1_at	Hypothetical protein	-35.60
Mtr.38650.1.S1_at	Anthocyanidin synthase	-35.56
Mtr.10298.1.S1_at	Photosystem II 22 kDa protein	-35.51
Mtr.10789.1.S1_at	Chlorophyll a oxygenase	-35.49
Mtr.47266.1.S1_at	DNA-binding protein 4	-35.48
Mtr.10797.1.S1_at	Thylakoid membrane phosphoprotein 14 kDa	-35.26
Mtr.5344.1.S1_at	Cellulose synthase	-35.07
Mtr.24034.1.S1_s_at	Hypothetical protein	-35.04
Msa.565.1.S1_at	Hypothetical protein	-34.89
Mtr.28829.1.S1_at	Hypothetical protein	-34.88
Mtr.12965.1.S1_at	Hypothetical protein	-34.84
Mtr.12028.1.S1_at	Hypothetical protein	-34.69
Mtr.10816.1.S1_at	Hypothetical protein	-34.64
Mtr.24825.1.S1_s_at	Cytochrome P450	-34.62
Mtr.19547.1.S1_at	Nucleoside phosphatase	-34.61
Mtr.21416.1.S1_at	Ribonuclease	-34.33
Mtr.31205.1.S1_at	Hypothetical protein	-34.28
Mtr.10182.1.S1_at	PREG1-like negative regulator-like protein	-34.23
Mtr.43262.1.S1_at	Hypothetical protein	-34.16
Mtr.6657.1.S1_s_at	Hypothetical protein	-34.13
Mtr.43442.1.S1_at	Hypothetical protein	-33.91
Mtr.13704.1.S1_at	Hypothetical protein	-33.81
Mtr.46512.1.S1_at	Alpha/beta-amyrin synthase	-33.66
Mtr.8799.1.S1_at	Raffinose synthase	-33.58
Mtr.10911.1.S1_at	Phenylpropanoid:glucosyltransferase 1	-33.55
Mtr.37335.1.S1_at	Chlorophyll a/b-binding protein	-33.54
Msa.1851.1.S1_at	Hypothetical protein	-33.51
Mtr.24540.1.S1_s_at	Peroxidase	-33.50
Mtr.14356.1.S1_at	Photosystem I reaction centre subunit IV	-33.33

Mtr.43317.1.S1_s_at	Photosystem II core complex proteins psbY	-33.05
Mtr.10377.1.S1_at	Hypothetical protein	-33.01
Mtr.5633.1.S1_at	Esterase/lipase/thioesterase	-32.98
Mtr.34115.1.S1_at	Hypothetical protein	-32.97
Mtr.20297.1.S1_at	Lipocalin	-32.88
Mtr.22060.1.S1_at	Alanine aminotransferase	-32.80
Mtr.11603.1.S1_at	Non-phototropic hypocotyl-like protein	-32.69
Msa.1752.1.S1_at	Hypothetical protein	-32.66
Mtr.44732.1.S1_at	GDSL-motif lipase/hydrolase	-32.57
Mtr.28584.1.S1_s_at	Hypothetical protein	-32.56
Mtr.49640.1.S1_at	Hypothetical protein	-32.45
Mtr.11545.1.S1_at	Hypothetical protein	-32.43
Mtr.7344.1.S1_at	Hypothetical protein	-32.33
Mtr.16620.1.S1_at	Phosphoglycerate/bisphosphoglycerate mutase	-32.29
Mtr.33459.1.S1_s_at	Hypothetical protein	-32.16
Mtr.9185.1.S1_at	Prephenate dehydratase	-32.08
Mtr.12160.1.S1_at	Mitochondrial carnitine/acylcarnitine carrier-like protein	-32.04
Mtr.40246.1.S1_at	Hypothetical protein	-31.98
Mtr.17364.1.S1_at	D-galactoside/L-rhamnose binding SUEL lectin	-31.85
Msa.1913.1.S1_at	Hypothetical protein	-31.83
Msa.2574.1.S1_at	Hypothetical protein	-31.79
Mtr.7512.1.S1_at	Hypothetical protein	-31.70
Mtr.47629.1.S1_at	Aldo/keto reductase	-31.55
Mtr.43295.1.S1_at	Hypothetical protein	-31.49
Mtr.11664.1.S1_at	MYB transcription factor	-31.30
Mtr.18757.1.S1_at	Sulfate transporter	-31.26
Mtr.18560.1.S1_at	dTDP-glucose 4-6-dehydratase	-31.22
Mtr.34850.1.S1_s_at	Hypothetical protein	-31.20
Mtr.21257.1.S1_at	Late embryogenesis abundant protein	-31.05
Mtr.25935.1.S1_at	Auxin-induced protein 15A	-30.87
Mtr.44534.1.S1_at	Hypothetical protein	-30.77
Mtr.25652.1.S1_at	Cell-wall P4 protein	-30.74
Mtr.44640.1.S1_at	Hypothetical protein	-30.67
Mtr.8659.1.S1_s_at	Beta-galactosidase	-30.66
Msa.1549.1.S1_at	Hypothetical protein	-30.59
Mtr.43381.1.S1_at	Hypothetical protein	-30.58
Mtr.39886.1.S1_at	Hypothetical protein	-30.55
Mtr.21256.1.S1_at	Late embryogenesis abundant protein	-30.51
Mtr.5594.1.S1_at	Ca2+/H+ exchanger	-30.42
Mtr.32904.1.S1_s_at	Phosphoglycerate kinase	-30.39
Mtr.7091.1.S1_at	Zinc-finger-like protein	-30.33
Mtr.47934.1.S1_at	Amino acid permease 6	-30.14
Mtr.40994.1.S1_at	ABC transporter	-30.01
Mtr.24189.1.S1_s_at	Fatty acid desaturase	-29.99
Mtr.10982.1.S1_at	Serine/threonine-specific protein kinase	-29.94
Mtr.32479.1.S1_at	Phosphoribosylaminoimidazolecarboxamide formyltransferase	-29.80
Msa.1550.1.S1_at	Hypothetical protein	-29.62
Mtr.28616.1.S1_at	ABC transporter	-29.32
Msa.1096.1.S1_at	Hypothetical protein	-29.29
Mtr.44488.1.S1_at	Hypothetical protein	-29.27
Mtr.49062.1.S1_at	Heat shock protein DnaJ	-29.26
Mtr.22140.1.S1_at	Glycosyltransferase	-29.18
Mtr.36794.1.S1_at	Hypothetical protein	-28.91
Mtr.39379.1.S1_at	Amino acid permease 6	-28.89

Mtr.9354.1.S1_at	ADP-glucose pyrophosphorylase	-28.78
Msa.3176.1.S1_at	Hypothetical protein	-28.68
Mtr.12305.1.S1_at	Chlorophyll a/b-binding protein CP24	-28.57
Mtr.37297.1.S1_at	Plastidic aldolase	-28.55
Mtr.51080.1.S1_at	Phospholipid/glycerol acyltransferase	-28.45
Mtr.9187.1.S1_at	Hypothetical protein	-28.45
Mtr.6517.1.S1_at	Flavonoid 3'-hydroxylase	-28.45
Mtr.33577.1.S1_at	Starch branching enzyme I	-28.39
Mtr.37529.1.S1_at	Magnesium chelatase	-28.38
Mtr.14782.1.S1_at	Isopenicillin N synthase	-28.29
Mtr.32687.1.S1_at	Hypothetical protein	-28.26
Mtr.27626.1.S1_s_at	Hypothetical protein	-28.23
Mtr.10566.1.S1_at	Hypothetical protein	-28.18
Mtr.36871.1.S1_at	Calcium-dependent protein kinase	-28.16
Mtr.6753.1.S1_at	Hypothetical protein	-28.11
Mtr.3123.1.S1_at	Hypothetical protein	-28.10
Mtr.25935.1.S1_x_at	Auxin-induced protein 15A	-28.10
Mtr.42339.1.S1_at	Hypothetical protein	-28.00
Mtr.10377.1.S1_s_at	Hypothetical protein	-27.96
Mtr.41746.1.S1_at	DnaJ domain protein	-27.95
Mtr.20948.1.S1_s_at	Membrane located receptor-like protein	-27.91
Mtr.37632.1.S1_at	Tetrapyrrole-binding protein	-27.88
Mtr.33008.1.S1_at	Hypothetical protein	-27.88
Mtr.9780.1.S1_at	Subtilisin-like protease	-27.59
Mtr.38920.1.S1_s_at	NFU3 protein	-27.47
Mtr.28243.1.S1_at	Hypothetical protein	-27.47
Mtr.22013.1.S1_s_at	Peroxiredoxin Q	-27.32
Mtr.9248.1.S1_at	Hypothetical protein	-27.27
Mtr.10927.1.S1_at	Regulator of chromosome condensation-like protein	-27.20
Mtr.34779.1.S1_at	GDSL-motif lipase/hydrolase	-27.13
Mtr.34481.1.S1_at	Abnormal spindle-like protein	-27.01
Mtr.1392.1.S1_s_at	Hypothetical protein	-26.95
Mtr.4580.1.S1_at	ABC transporter	-26.86
Mtr.12026.1.S1_at	Hypothetical protein	-26.81
Mtr.38370.1.S1_s_at	Hypothetical protein	-26.65
Mtr.42567.1.S1_at	Phantastica transcription factor	-26.62
Mtr.34852.1.S1_at	Raucaffricine-O-beta-D-glucosidase	-26.59
Mtr.31792.1.S1_at	Hypothetical protein	-26.59
Mtr.41363.1.S1_at	Immunophilin / FKBP-type peptidyl-prolyl cis-trans isomerase	-26.59
Mtr.43702.1.S1_s_at	Thylakoid lumenal protein	-26.43
Mtr.20149.1.S1_at	Hypothetical protein	-26.36
Mtr.26958.1.S1_at	Hypothetical protein	-26.36
Mtr.11283.1.S1_at	Hypothetical protein	-26.36
Mtr.18579.1.S1_at	Myb, DNA-binding	-26.23
Mtr.37883.1.S1_at	Hypothetical protein	-26.15
Mtr.9564.1.S1_at	FKBP like protein	-26.07
Mtr.35826.1.S1_at	Hypothetical protein	-26.05
Mtr.51510.1.S1_at	Hypothetical protein	-25.96
Mtr.11963.1.S1_s_at	Sulphate transporter	-25.91
Mtr.17316.1.S1_at	Generic methyltransferase	-25.87
Mtr.40462.1.S1_at	Hypothetical protein	-25.85
Mtr.4339.1.S1_at	Hypothetical protein	-25.76
Msa.2685.1.S1_at	Hypothetical protein	-25.69
Mtr.32990.1.S1_at	Non-phototropic hypocotyl-like protein	-25.48

Mtr.18023.1.S1_at	Ferredoxin	-25.44
Mtr.4947.1.S1_at	Cytochrome P-450	-25.23
Mtr.38433.1.S1_at	Hypothetical protein	-25.22
Mtr.31215.1.S1_s_at	Hypothetical protein	-25.11
Mtr.44109.1.S1_at	Nitrate transporter	-25.08
Mtr.8637.1.S1_at	Hypothetical protein	-25.08
Mtr.33061.1.S1_at	Hypothetical protein	-25.01
Mtr.14083.1.S1_at	Hypothetical protein	-24.96
Mtr.10840.1.S1_at	Hypothetical protein	-24.76
Mtr.33659.1.S1_at	Hypothetical protein	-24.74
Mtr.2101.1.S1_at	Hypothetical protein	-24.72
Mtr.40092.1.S1_at	Dehydroascorbate reductase	-24.68
Mtr.39747.1.S1_at	UDP-glucose:flavonoid 3-O-glucosyltransferase	-24.62
Mtr.41722.1.S1_at	Hypothetical protein	-24.53
Mtr.21118.1.S1_at	Hypothetical protein	-24.53
Mtr.34490.1.S1_at	Hypothetical protein	-24.45
Mtr.43480.1.S1_at	Hypothetical protein	-24.44
Mtr.36842.1.S1_s_at	Nodulin 26-like protein	-24.34
Mtr.27626.1.S1_at	Hypothetical protein	-24.30
Mtr.11236.1.S1_at	UDP-glucosyltransferase	-24.30
Mtr.12445.1.S1_at	Hypothetical protein	-24.25
Mtr.49762.1.S1_at	Elongation factor g	-24.22
Mtr.42596.1.S1_at	LLS1-like protein	-24.20
Mtr.48257.1.S1_at	Hypothetical protein	-24.20
Msa.2533.1.S1_at	Hypothetical protein	-24.07
Mtr.22279.1.S1_at	Hypothetical protein	-24.05
Mtr.33696.1.S1_at	4-coumarate-CoA ligase	-24.05
Mtr.49764.1.S1_at	Auxin Efflux Carrier	-24.04
Mtr.10013.1.S1_at	Hypothetical protein	-23.71
Mtr.37608.1.S1_at	Hypothetical protein	-23.67
Mtr.42925.1.S1_s_at	Ribulose bisphosphate carboxylase/oxygenase activase	-23.66
Mtr.49641.1.S1_at	Hypothetical protein	-23.63
Mtr.42918.1.S1_at	Protein disulfide isomerase	-23.60
Mtr.37228.1.S1_at	UDP-glycose:flavonoid glycosyltransferase	-23.59
Mtr.34693.1.S1_at	Hypothetical protein	-23.55
Mtr.44580.1.S1_at	Hypothetical protein	-23.53
Mtr.31791.1.S1_at	Hypothetical protein	-23.51
Mtr.38366.1.S1_at	Hypothetical protein	-23.42
Mtr.13968.1.S1_at	GH3-like protein	-23.35
Mtr.49839.1.S1_x_at	Hypothetical protein	-23.31
Mtr.31754.1.S1_x_at	Isoflavonoid glucosyltransferase	-23.30
Mtr.5651.1.S1_at	Hypothetical protein	-23.25
Mtr.13096.1.S1_at	Hypothetical protein	-23.25
Mtr.46159.1.S1_at	bZIP transcription factor	-23.08
Mtr.10441.1.S1_at	mRNA-binding protein	-22.77
Mtr.5310.1.S1_s_at	Light harvesting protein	-22.74
Mtr.33489.1.S1_at	ABC transporter	-22.72
Mtr.13817.1.S1_at	AX110P-like protein	-22.68
Mtr.32665.1.S1_s_at	Myo-inositol-1-phosphate synthase	-22.62
Mtr.41073.1.S1_at	FPF1 protein	-22.60
Mtr.31754.1.S1_at	Isoflavonoid glucosyltransferase	-22.47
Mtr.33723.1.S1_at	Hypothetical protein	-22.43
Mtr.18406.1.S1_at	Hypothetical protein	-22.41
Mtr.35838.1.S1_at	Nitrate transporter	-22.35

Mtr.13203.1.S1_at	Thylakoid lumenal 29.8 kDa protein	-22.32
Mtr.12202.1.S1_at	Ribulose 1,5-bisphosphate carboxylase small subunit	-22.28
Mtr.41275.1.S1_at	Kinesin-like protein	-22.11
Mtr.13996.1.S1_at	Hypothetical protein	-22.02
Mtr.11271.1.S1_at	Hypothetical protein	-22.00
Mtr.48595.1.S1_at	Hypothetical protein	-21.99
Mtr.2073.1.S1_at	Copper/topa quinone amine oxidase	-21.96
Mtr.37662.1.S1_at	Proline-rich family protein	-21.92
Msa.1254.1.S1_at	Hypothetical protein	-21.83
Mtr.29089.1.S1_s_at	Peroxidase	-21.76
Mtr.27575.1.S1_at	Nitrate transporter	-21.68
Mtr.13401.1.S1_at	Hypothetical protein	-21.60
Mtr.37245.1.S1_at	Chlorophyll a-b binding protein	-21.59
Mtr.30739.1.S1_s_at	Hypothetical protein	-21.59
Mtr.10828.1.S1_at	Glucosyltransferase-13	-21.58
Mtr.40632.1.S1_at	Hypothetical protein	-21.53
Mtr.19937.1.S1_at	Elongation factor G	-21.49
Mtr.13356.1.S1_at	Allene oxide cyclase C4	-21.46
Mtr.34625.1.S1_at	Cytochrome P450 71A6	-21.44
Mtr.26786.1.S1_at	Hypothetical protein	-21.27
Mtr.12295.1.S1_at	Inorganic pyrophosphatase-like protein	-21.22
Mtr.42609.1.S1_at	Hypothetical protein	-21.18
Mtr.32336.1.S1_at	Cytochrome P450 71A21	-21.15
Mtr.2893.1.S1_at	Hypothetical protein	-21.15
Mtr.45559.1.S1_at	ARK2 product/receptor-like serine/threonine protein kinase	-21.08
Mtr.36690.1.S1_at	GH3-like protein	-21.06
Mtr.25285.1.S1_at	Symbiotic ammonium transporter	-21.05
Mtr.50822.1.S1_x_at	Chlorophyll A-B binding protein	-21.00
Mtr.21054.1.S1_at	Mitochodrial transcription termination factor	-20.92
Mtr.5804.1.S1_at	Hypothetical protein	-20.90
Mtr.37286.1.S1_at	Furostanol glycoside 26-O-beta-glucosidase	-20.87
Msa.1688.1.S1_at	Hypothetical protein	-20.87
Mtr.12909.1.S1_at	Hypothetical protein	-20.76
Mtr.12056.1.S1_s_at	Cytochrome P450	-20.76
Mtr.47958.1.S1_at	Hypothetical protein	-20.72
Mtr.43727.1.S1_s_at	Squalene monooxygenase 1	-20.70
Mtr.48892.1.S1_at	Hypothetical protein	-20.70
Msa.3166.1.S1_at	Hypothetical protein	-20.68
Msa.3115.1.S1_at	Hypothetical protein	-20.64
Mtr.14704.1.S1_at	Carbamoyl-phosphate synthase	-20.61
Mtr.43628.1.S1_at	Isoflavonoid glucosyltransferase	-20.59
Mtr.27854.1.S1_at	NADP-dependent oxidoreductase P1	-20.55
Mtr.40691.1.S1_at	Lipoxygenase	-20.46
Mtr.46540.1.S1_at	CP12	-20.42
Mtr.27388.1.S1_s_at	Rubisco activase	-20.39
Mtr.11326.1.S1_at	Leaf senescence protein	-20.38
Mtr.43610.1.S1_at	Hypothetical protein	-20.31
Mtr.13324.1.S1_at	Hypothetical protein	-20.30
Mtr.11477.1.S1_at	PsbP protein	-20.28
Mtr.15413.1.S1_at	Haloacid dehalogenase	-20.25
Mtr.6743.1.S1_at	2-oxoglutarate/malate translocator	-20.22
Mtr.10946.1.S1_at	Hypothetical protein	-20.21
Mtr.15285.1.S1_at	Myo-inositol-1-phosphate synthase	-20.20
Msa.1916.1.S1_at	Hypothetical protein	-20.20

Mtr.38371.1.S1_at	Hypothetical protein	-20.18
Msa.1822.1.S1_at	Hypothetical protein	-20.13
Mtr.24508.1.S1_at	Hypothetical protein	-20.10
Mtr.21535.1.S1_at	Serine/threonine protein kinase	-20.04
Mtr.44312.1.S1_at	Hypothetical protein	-20.03
Mtr.43075.1.S1_at	Cyanogenic Beta-Glucosidase	-20.01
Msa.3042.1.S1_s_at	Hypothetical protein	-20.00
Mtr.20943.1.S1_at	Serine/threonine protein kinase	-19.91
Mtr.21006.1.S1_at	Hypothetical protein	-19.84
Mtr.27619.1.S1_at	Hypothetical protein	-19.82
Mtr.32939.1.S1_at	Hypothetical protein	-19.81
Mtr.18997.1.S1_at	Zn-finger, Dof type	-19.81
Mtr.47158.1.S1_s_at	Hypothetical protein	-19.67
Mtr.6714.1.S1_at	Hypothetical protein	-19.66
Mtr.3942.1.S1_at	Hypothetical protein	-19.51
Mtr.41693.1.S1_at	CND41, chloroplast nucleoid DNA binding protein	-19.41
Mtr.20116.1.S1_s_at	Hypothetical protein	-19.39
Mtr.51059.1.S1_x_at	E-class P450	-19.37
Mtr.37042.1.S1_at	Hypothetical protein	-19.36
Mtr.5369.1.S1_at	Nitrate transporter	-19.33
Mtr.48723.1.S1_at	Hypothetical protein	-19.32
Mtr.14762.1.S1_at	Flavoprotein monooxygenase	-19.32
Mtr.11295.1.S1_at	Transport protein	-19.31
Msa.1384.1.S1_at	Hypothetical protein	-19.31
Mtr.45060.1.S1_at	Salt-tolerance protein	-19.25
Mtr.40788.1.S1_at	Hypothetical protein	-19.10
Mtr.11524.1.S1_at	Hypothetical protein	-19.00
Mtr.38923.1.S1_at	Cytoplasmic intermediate filament protein	-18.93
Mtr.20727.1.S1_at	Aldehyde dehydrogenase	-18.91
Mtr.13781.1.S1_at	Hypothetical protein	-18.85
Mtr.43375.1.S1_at	Hypothetical protein	-18.84
Mtr.35200.1.S1_at	Elongation factor 1 alpha	-18.84
Mtr.38889.1.S1_at	Metacaspase 3	-18.81
Mtr.22257.1.S1_s_at	Hypothetical protein	-18.78
Mtr.5368.1.S1_at	Chlorophyll a/b-binding protein	-18.77
Mtr.6033.1.S1_at	Peroxidase	-18.77
Mtr.32922.1.S1_at	Amino acid transporter	-18.76
Mtr.10844.1.S1_at	Peroxiredoxin Q	-18.72
Mtr.14906.1.S1_at	Major intrinsic protein	-18.69
Mtr.5621.1.S1_at	Hypothetical protein	-18.65
Mtr.44339.1.S1_at	Hypothetical protein	-18.64
Mtr.12060.1.S1_at	Annexin	-18.62
Mtr.24823.1.S1_at	Hypothetical protein	-18.55
Mtr.28742.1.S1_at	Very-long-chain fatty acid condensing enzyme CUT1	-18.50
Mtr.14011.1.S1_at	Hypothetical protein	-18.46
Mtr.8269.1.S1_at	Hypothetical protein	-18.43
Mtr.6693.1.S1_s_at	NFU3 protein	-18.42
Mtr.20846.1.S1_at	MtN3 and saliva related transmembrane protein	-18.36
Mtr.8426.1.S1_at	Chlorophyll a/b binding protein	-18.30
Mtr.7085.1.S1_at	Fatty acid elongase	-18.28
Mtr.40597.1.S1_at	Seed maturation protein	-18.27
Mtr.32162.1.S1_at	Hypothetical protein	-18.25
Mtr.51345.1.S1_at	Hypothetical protein	-18.25
Mtr.45678.1.S1_at	SAM (and some other nucleotide) binding protein	-18.24

Mtr.3259.1.S1_at	Hypothetical protein	-18.24
Mtr.20033.1.S1_at	Hypothetical protein	-18.23
Mtr.12900.1.S1_at	Iron reductase	-18.21
Mtr.13434.1.S1_at	Peroxisomal membrane protein	-18.19
Mtr.48150.1.S1_s_at	Hypothetical protein	-18.17
Mtr.42753.1.S1_at	Hypothetical protein	-18.09
Mtr.38087.1.S1_at	Hyoscyamine 6 beta-hydroxylase	-18.06
Mtr.37623.1.S1_at	3-beta-hydroxysteroiddehydrogenase	-18.03
Mtr.31606.1.S1_at	Phosphoethanolamine N-methyltransferase	-18.03
Mtr.43709.1.S1_at	Starch synthase isoform SS III	-17.99
Mtr.14112.1.S1_s_at	PAP fibrillin	-17.94
Mtr.45561.1.S1_at	4-coumarate-CoA ligase	-17.94
Msa.1863.1.S1_at	Hypothetical protein	-17.92
Mtr.37210.1.S1_at	Chlorophyll a-b binding protein	-17.91
Msa.2778.1.S1_at	Hypothetical protein	-17.85
Mtr.43044.1.S1_at	NADP-dependent glyceraldehyde-3-phosphate dehydrogenase	-17.85
Mtr.45032.1.S1_at	Squalene epoxidase	-17.82
Mtr.12543.1.S1_at	Early nodulin-like protein 2	-17.81
Mtr.5960.1.S1_s_at	Hypothetical protein	-17.80
Mtr.6752.1.S1_at	10 kDa photosystem II polypeptide	-17.77
Mtr.27132.1.S1_s_at	Histidine-containing phosphotransfer protein	-17.77
Mtr.14592.1.S1_at	Peroxidase	-17.76
Mtr.9624.1.S1_at	ABC transporter	-17.76
Mtr.13248.1.S1_at	Protein kinase	-17.70
Mtr.39379.1.S1_s_at	Amino acid permease 6	-17.64
Mtr.42369.1.S1_at	Hypothetical protein	-17.62
Mtr.6709.1.S1_at	Hypothetical protein	-17.57
Mtr.35044.1.S1_at	Nine-cis-epoxycarotenoid dioxygenase2	-17.55
Msa.3147.1.S1_at	Hypothetical protein	-17.54
Mtr.44975.1.S1_at	ABC transporter	-17.51
Mtr.40755.1.S1_at	ERD7 protein	-17.50
Mtr.11302.1.S1_at	Glycosyl hydrolase	-17.47
Msa.1086.1.S1_at	Hypothetical protein	-17.47
Msa.941.1.S1_at	Hypothetical protein	-17.44
Mtr.40667.1.S1_at	Abscisic acid-induced-like protein	-17.43
Mtr.49516.1.S1_at	Gigantea protein	-17.43
Mtr.5570.1.S1_at	Plasma membrane sulphate transporter	-17.41
Mtr.10464.1.S1_at	Phosphoribulokinase	-17.38
Mtr.27878.1.S1_at	Isoflavone reductase	-17.33
Mtr.41919.1.S1_s_at	Hypothetical protein	-17.32
Mtr.49786.1.S1_x_at	Glycosyl hydrolases	-17.31
Mtr.39126.1.S1_at	Hypothetical protein	-17.30
Mtr.24883.1.S1_at	Hypothetical protein	-17.29
Mtr.33459.1.S1_at	Hypothetical protein	-17.26
Mtr.13143.1.S1_at	Starch synthase isoform SS III	-17.25
Mtr.4983.1.S1_at	Hypothetical protein	-17.25
Mtr.20386.1.S1_s_at	Hemopexin	-17.25
Mtr.10630.1.S1_at	Nonspecific lipid-transfer protein 2	-17.21
Mtr.9205.1.S1_at	Hypothetical protein	-17.19
Mtr.12964.1.S1_at	Hypothetical protein	-17.17
Mtr.11503.1.S1_at	Cold acclimation protein	-17.17
Mtr.5418.1.S1_at	Magnesium transporter	-17.13
Mtr.7842.1.S1_at	Hypothetical protein	-17.09
Mtr.21616.1.S1_at	Hypothetical protein	-17.02

Mtr.10289.1.S1_at	SANT/MYB domain protein	-17.00
Mtr.38165.1.S1_at	Serine/threonine protein kinase	-16.99
Msa.1809.1.S1_at	Hypothetical protein	-16.99
Mtr.14527.1.S1_at	Hypothetical protein	-16.98
Msa.1732.1.S1_at	Hypothetical protein	-16.94
Mtr.12736.1.S1_at	Hypothetical protein	-16.93
Mtr.29086.1.S1_at	Hypothetical protein	-16.92
Mtr.7389.1.S1_at	Yippee-like protein	-16.89
Mtr.48146.1.S1_s_at	Boron transporter	-16.89
Mtr.20226.1.S1_at	UDP-glucuronosyl/UDP-glucosyltransferase	-16.79
Mtr.12624.1.S1_at	ABC1 family protein	-16.78
Mtr.11536.1.S1_at	SOS2-like protein kinase PKS3	-16.76
Mtr.42075.1.S1_at	Cytochrome P450	-16.72
Mtr.43919.1.S1_at	Two-component response regulator-like APRR7	-16.71
Mtr.37463.1.S1_at	CesA2	-16.69
Mtr.49774.1.S1_at	Auxin responsive SAUR	-16.68
Mtr.43683.1.S1_at	Protein phosphatase 2C	-16.67
Mtr.12722.1.S1_at	Hypothetical protein	-16.65
Mtr.45292.1.S1_at	Hypothetical protein	-16.63
Mtr.37624.1.S1_s_at	3-beta-hydroxysteroiddehydrogenase	-16.60
Mtr.4029.1.S1_at	CTF2A	-16.59
Mtr.26473.1.S1_at	Isoamylase	-16.56
Mtr.8811.1.S1_at	Hypothetical protein	-16.55
Msa.3135.1.S1_at	Hypothetical protein	-16.54
Mtr.10192.1.S1_at	DREB-like protein	-16.53
Mtr.43701.1.S1_at	Thylakoid lumenal protein	-16.50
Msa.2424.1.S1_at	Hypothetical protein	-16.49
Mtr.9467.1.S1_at	Glutaredoxin	-16.48
Mtr.29595.1.S1_at	Hypothetical protein	-16.48
Mtr.20847.1.S1_at	MtN3 and saliva related transmembrane protein	-16.46
Mtr.25633.1.S1_at	2-Cys peroxiredoxin	-16.46
Mtr.46511.1.S1_at	Alpha/beta-amyrin synthase	-16.46
Mtr.50822.1.S1_at	Chlorophyll A-B binding protein	-16.45
Mtr.41759.1.S1_at	Rac GTPase activating protein	-16.43
Mtr.12720.1.S1_at	Lipoxygenase	-16.43
Mtr.16791.1.S1_s_at	Thylakoid lumen protein	-16.42
Mtr.39712.1.S1_at	Rac GTPase activating protein 3	-16.39
Mtr.51399.1.S1_at	Hypothetical protein	-16.31
Mtr.5490.1.S1_at	Hypothetical protein	-16.27
Mtr.6744.1.S1_at	Hypothetical protein	-16.22
Mtr.38438.1.S1_at	APG1	-16.16
Mtr.5325.1.S1_at	Hypothetical protein	-16.12
Mtr.250.1.S1_at	Hypothetical protein	-16.04
Mtr.34428.1.S1_s_at	Glyceraldehyde-3-phosphate dehydrogenase B	-16.03
Mtr.33622.1.S1_at	Dehydration-responsive family protein	-15.99
Mtr.5305.1.S1_at	Cytochrome P450	-15.94
Mtr.4339.1.S1_s_at	Hypothetical protein	-15.82
Mtr.39266.1.S1_at	Cation diffusion facilitator 9	-15.80
Mtr.41681.1.S1_at	Phosphoglycerate kinase	-15.78
Mtr.2200.1.S1_at	Hypothetical protein	-15.77
Mtr.10992.1.S1_at	Arabinogalactan protein-like protein	-15.71
Mtr.40866.1.S1_at	Hydrolase-like protein	-15.71
Mtr.19622.1.S1_s_at	Hypothetical protein	-15.67
Mtr.6878.1.S1_s_at	Hypothetical protein	-15.67

Mtr.44856.1.S1_at	Beta-amylase	-15.64
Mtr.11662.1.S1_at	Hypothetical protein	-15.63
Mtr.20298.1.S1_at	Hypothetical protein	-15.60
Mtr.13958.1.S1_at	Beta-amylase	-15.59
Mtr.41129.1.S1_s_at	Hypothetical protein	-15.58
Mtr.47933.1.S1_at	Amino acid transporter	-15.47
Mtr.6877.1.S1_at	Drought responsive element binding protein	-15.45
Mtr.32662.1.S1_at	Beta-amylase	-15.45
Msa.3182.1.S1_at	Hypothetical protein	-15.44
Mtr.27885.1.S1_s_at	Hypothetical protein	-15.40
Mtr.17489.1.S1_at	Hypothetical protein	-15.39
Mtr.48925.1.S1_at	Hypothetical protein	-15.38
Mtr.42178.1.S1_at	Starch synthase I	-15.38
Mtr.32351.1.S1_s_at	Hypothetical protein	-15.36
Mtr.50516.1.S1_at	Deoxyribodipyrimidine photolyase	-15.36
Mtr.10972.1.S1_at	Hypothetical protein	-15.35
Mtr.33019.1.S1_at	Hypothetical protein	-15.35
Mtr.23337.1.S1_at	Nonphototrophic hypocotyl 1b	-15.31
Mtr.43994.1.S1_at	Hypothetical protein	-15.30
Mtr.32213.1.S1_at	Nitrite transport protein	-15.28
Mtr.9137.1.S1_at	Hypothetical protein	-15.25
Mtr.40706.1.S1_s_at	ADP-glucose pyrophosphorylase large subunit	-15.25
Msa.928.1.S1_at	Hypothetical protein	-15.24
Mtr.37245.1.S1_s_at	Chlorophyll a-b binding protein	-15.23
Mtr.46001.1.S1_s_at	Hypothetical protein	-15.22
Mtr.39718.1.S1_at	Hypothetical protein	-15.20
Msa.1749.1.S1_at	Hypothetical protein	-15.20
Mtr.13595.1.S1_at	Lecithine cholesterol acyltransferase	-15.17
Mtr.34717.1.S1_at	Disease resistance response protein	-15.15
Mtr.38722.1.S1_at	Hypothetical protein	-15.10
Mtr.52116.1.S1_at	Glyceraldehyde 3-phosphate dehydrogenase	-15.09
Mtr.9257.1.S1_at	Starch synthase I	-15.04
Mtr.38471.1.S1_at	Ring finger protein	-15.03
Mtr.27471.1.S1_s_at	Hypothetical protein	-15.02
Mtr.19806.1.S1_at	Hypothetical protein	-15.01
Mtr.42830.1.S1_at	Hypothetical protein	-15.01
Mtr.43077.1.S1_at	Hypothetical protein	-15.00
Mtr.27892.1.S1_at	Hypothetical protein	-14.99
Mtr.11888.1.S1_at	Aldehyde dehydrogenase	-14.97
Mtr.8418.1.S1_s_at	Xylulose kinase	-14.95
Mtr.44199.1.S1_at	Hypothetical protein	-14.95
Mtr.17318.1.S1_at	Magnesium-protoporphyrin IX monomethyl ester aerobic oxidative cyclase	-14.90
Mtr.6738.1.S1_at	Receptor-like protein kinase	-14.88
Mtr.2103.1.S1_at	Hypothetical protein	-14.87
Mtr.51819.1.S1_at	Streptomyces cyclase/dehydrase	-14.87
Mtr.43503.1.S1_at	Hypothetical protein	-14.85
Mtr.40981.1.S1_at	Cellulose synthase	-14.83
Mtr.51040.1.S1_s_at	Cytochrome b-245	-14.78
Msa.1095.1.S1_at	Hypothetical protein	-14.76
Msa.2842.1.S1_at	Hypothetical protein	-14.74
Mtr.21762.1.S1_s_at	Hypothetical protein	-14.72
Mtr.8572.1.S1_at	Thaumatin-like protein	-14.68
Mtr.37731.1.S1_at	PWWP domain protein	-14.66
Mtr.43197.1.S1_at	Hypothetical protein	-14.64

Mtr.10206.1.S1_at	Pentatricopeptide (PPR) repeat-containing protein	-14.63
Mtr.8362.1.S1_at	Hypothetical protein	-14.62
Mtr.43744.1.S1_at	Hypothetical protein	-14.62
Mtr.32865.1.S1_at	Hypothetical protein	-14.59
Mtr.43509.1.S1_at	Cyclic-AMP-dependent transcription factor ATF-5	-14.57
Mtr.34610.1.S1_at	Hypothetical protein	-14.49
Mtr.51216.1.S1_s_at	Homeodomain-like	-14.49
Mtr.12422.1.S1_at	Ferredoxin-dependent glutamate synthase	-14.47
Mtr.14435.1.S1_at	Calcium-binding EF-hand protein	-14.47
Msa.1642.1.S1_at	Hypothetical protein	-14.44
Msa.1363.1.S1_at	Hypothetical protein	-14.39
Mtr.13442.1.S1_at	Hypothetical protein	-14.39
Msa.2278.1.S1_at	Hypothetical protein	-14.34
Mtr.8591.1.S1_at	Hypothetical protein	-14.33
Msa.2800.1.S1_at	Hypothetical protein	-14.30
Msa.1743.1.S1_at	Hypothetical protein	-14.27
Mtr.33666.1.S1_at	Hypothetical protein	-14.26
Mtr.6070.1.S1_at	Histidine kinase	-14.26
Mtr.10593.1.S1_at	Allene oxide cyclase	-14.25
Mtr.10857.1.S1_at	MOLYBDENUM COFACTOR BIOSYNTHESIS PROTEIN C	-14.24
Mtr.18705.1.S1_at	Peptidase C1A	-14.22
Mtr.2960.1.S1_at	Magnesium transporter	-14.18
Msa.2629.1.S1_at	Hypothetical protein	-14.16
Mtr.40587.1.S1_at	Glyoxalase	-14.16
Mtr.51216.1.S1_at	Homeodomain-like	-14.15
Mtr.11387.1.S1_at	Hypothetical protein	-14.13
Mtr.12414.1.S1_at	Apospory-associated protein C	-14.10
Mtr.42854.1.S1_at	Thioredoxin m	-14.07
Msa.2953.1.S1_at	Hypothetical protein	-14.07
Mtr.40697.1.S1_at	Putativepod-specific dehydrogenase	-14.02
Mtr.15310.1.S1_at	Fatty acid desaturase	-14.01
Mtr.38798.1.S1_at	Hypothetical protein	-14.00
Mtr.42587.1.S1_at	Heat shock transcription factor	-13.99
Mtr.32682.1.S1_at	Leucyl aminopeptidase	-13.96
Mtr.21718.1.S1_at	Leucine-rich repeat	-13.94
Mtr.24048.1.S1_at	Hypothetical protein	-13.93
Mtr.13592.1.S1_at	Photomorphogenesis repressor protein	-13.90
Mtr.50001.1.S1_s_at	Glutamate synthase	-13.89
Mtr.41906.1.S1_at	Expansin-related protein 1	-13.88
Mtr.12842.1.S1_at	Hypothetical protein	-13.88
Mtr.2675.1.S1_s_at	Transporter-like protein	-13.88
Msa.1760.1.S1_at	Hypothetical protein	-13.86
Mtr.13567.1.S1_at	Hypothetical protein	-13.86
Mtr.27621.1.S1_at	Hypothetical protein	-13.86
Mtr.20201.1.S1_at	Na+/H+ antiporter-like protein	-13.79
Mtr.5468.1.S1_at	Hypothetical protein	-13.76
Mtr.48866.1.S1_at	Hypothetical protein	-13.74
Mtr.27399.1.S1_at	Small basic membrane integral protein	-13.74
Mtr.41751.1.S1_at	Hypothetical protein	-13.71
Mtr.8900.1.S1_at	Hypothetical protein	-13.71
Mtr.7468.1.S1_at	10 kDa photosystem II polypeptide	-13.66
Mtr.39011.1.S1_at	Auxin-binding protein	-13.64
Mtr.22781.1.S1_at	Photosystem I-N subunit	-13.60
Mtr.30778.1.S1_at	Hypothetical protein	-13.60

Mtr.8207.1.S1_at	Hypothetical protein	-13.59
Mtr.20763.1.S1_s_at	ATP sulfurylase	-13.56
Mtr.37808.1.S1_at	Hypothetical protein	-13.56
Msa.2939.1.S1_at	Hypothetical protein	-13.55
Mtr.2477.1.S1_at	Hypothetical protein	-13.54
Mtr.11734.1.S1_at	Sulfate transporter	-13.54
Mtr.38432.1.S1_at	Temperature stress-induced lipocalin	-13.53
Mtr.27648.1.S1_at	Hypothetical protein	-13.51
Mtr.34177.1.S1_at	Hypothetical protein	-13.47
Mtr.41840.1.S1_at	Cation diffusion facilitator 10	-13.46
Mtr.44755.1.S1_at	LRR-like protein	-13.46
Mtr.36454.1.S1_at	Hypothetical protein	-13.43
Mtr.13959.1.S1_at	SHOOT1 protein	-13.42
Mtr.13851.1.S1_at	Hypothetical protein	-13.40
Msa.1853.1.S1_at	Hypothetical protein	-13.40
Msa.3048.1.S1_at	Hypothetical protein	-13.37
Mtr.9402.1.S1_at	Aldehyde dehydrogenase	-13.36
Mtr.22897.1.S1_s_at	Polygalacturonase	-13.31
Mtr.41070.1.S1_at	Hypothetical protein	-13.30
Msa.1943.1.S1_at	Hypothetical protein	-13.28
Mtr.25313.1.S1_at	Hypothetical protein	-13.27
Mtr.34902.1.S1_s_at	Phosphoenolpyruvate carboxylase	-13.27
Mtr.32681.1.S1_at	Hypothetical protein	-13.25
Msa.1101.1.S1_at	Hypothetical protein	-13.23
Mtr.50758.1.S1_at	Rubredoxin-type Fe(Cys)4 protein	-13.22
Mtr.14524.1.S1_at	Glucose/ribitol dehydrogenase	-13.21
Mtr.39530.1.S1_at	Hypothetical protein	-13.19
Mtr.43807.1.S1_at	Hypothetical protein	-13.17
Mtr.12949.1.S1_at	MURF2 protein	-13.17
Mtr.34703.1.S1_at	Hypothetical protein	-13.15
Mtr.42595.1.S1_at	Hypothetical protein	-13.15
Mtr.15310.1.S1_s_at	Fatty acid desaturase	-13.13
Mtr.6729.1.S1_at	Hypothetical protein	-13.12
Mtr.13134.1.S1_at	ABC1-like protein	-13.09
Mtr.43522.1.S1_at	Plastid-lipid associated protein PAP	-13.06
Mtr.15051.1.S1_s_at	Hypothetical protein	-13.01
Mtr.7544.1.S1_at	Isoflavonoid glucosyltransferase	-12.98
Mtr.20473.1.S1_at	Hypothetical protein	-12.98
Mtr.2086.1.S1_at	Hypothetical protein	-12.98
Mtr.42735.1.S1_at	Hypothetical protein	-12.95
Mtr.12899.1.S1_s_at	Hypothetical protein	-12.95
Mtr.39235.1.S1_at	Hypothetical protein	-12.94
Msa.1787.1.S1_at	Hypothetical protein	-12.94
Mtr.41132.1.S1_at	Hypothetical protein	-12.90
Mtr.13901.1.S1_at	Hypothetical protein	-12.87
Mtr.25013.1.S1_at	2'-hydroxy isoflavone/dihydroflavonol reductase	-12.87
Mtr.11921.1.S1_at	Thylakoid lumen protein	-12.87
Mtr.47369.1.S1_s_at	ABC transporter	-12.86
Mtr.38850.1.S1_at	Hypothetical protein	-12.83
Mtr.43215.1.S1_at	Hypothetical protein	-12.80
Mtr.35794.1.S1_at	Myo-inositol 1-phoshate synthase	-12.77
Mtr.45379.1.S1_at	Hypothetical protein	-12.76
Mtr.40162.1.S1_at	Reversibly glycosylated protein	-12.67
Mtr.42070.1.S1_at	Geminivirus replication protein-interacting protein	-12.66

Mtr.32657.1.S1_s_at	Beta-amylase	-12.60
Mtr.7993.1.S1_s_at	Glyceraldehyde-3-phosphate dehydrogenase	-12.60
Mtr.33484.1.S1_s_at	Hypothetical protein	-12.58
Mtr.29551.1.S1_at	Hypothetical protein	-12.53
Mtr.34799.1.S1_at	Hypothetical protein	-12.53
Mtr.23672.1.S1_at	51 kDa seed maturation protein	-12.53
Mtr.11548.1.S1_at	Starch synthase isoform SS III	-12.52
Mtr.27875.1.S1_at	ABC1	-12.48
Mtr.45376.1.S1_at	Nitrite transporter	-12.47
Mtr.32317.1.S1_s_at	Phototropin	-12.45
Mtr.33044.1.S1_s_at	Hypothetical protein	-12.44
Mtr.39964.1.S1_s_at	Hypothetical protein	-12.41
Msa.1191.1.S1_at	Hypothetical protein	-12.36
Mtr.42897.1.S1_at	Ycf23 protein	-12.34
Mtr.51053.1.S1_at	Hypothetical protein	-12.33
Mtr.27132.1.S1_at	Histidine-containing phosphotransfer protein	-12.31
Mtr.9728.1.S1_at	Hypothetical protein	-12.29
Mtr.12300.1.S1_at	Aminotransferase 2	-12.29
Mtr.39715.1.S1_at	Isopiperitenone reductase	-12.28
Mtr.44703.1.S1_at	Hypothetical protein	-12.27
Mtr.42112.1.S1_at	Hypothetical protein	-12.25
Mtr.36999.1.S1_at	Photomorphogenesis repressor protein	-12.22
Mtr.11736.1.S1_at	Aquaporin NIP-type	-12.21
Mtr.6362.1.S1_at	Aminopeptidase 2	-12.21
Mtr.44317.1.S1_at	ApaG protein	-12.19
Mtr.47226.1.S1_at	CBL-interacting protein kinase 1	-12.19
Mtr.12821.1.S1_s_at	Phosphoglucomutase	-12.18
Mtr.42019.1.S1_at	ClpB heat shock protein	-12.15
Mtr.18614.1.S1_at	Hypothetical protein	-12.14
Msa.1348.1.S1_at	Hypothetical protein	-12.09
Mtr.28079.1.S1_at	Hypothetical protein	-12.07
Mtr.43282.1.S1_at	2,7,4'-trihydroxyisoflavanone 4'-O-methyltransferase	-12.07
Mtr.38676.1.S1_at	Methyltransferase	-12.04
Mtr.12421.1.S1_at	Hypothetical protein	-12.03
Mtr.9582.1.S1_at	Glycogen(starch) synthase isoform II	-12.02
Mtr.15109.1.S1_s_at	Hypothetical protein	-12.02
Mtr.21518.1.S1_at	Cytochrome P450	-12.00
Mtr.15459.1.S1_at	Major intrinsic protein	-12.00
Mtr.5783.1.S1_at	Flavin containing monooxygenase 3	-11.99
Mtr.29826.1.S1_at	Hypothetical protein	-11.99
Mtr.41162.1.S1_at	Hypothetical protein	-11.98
Mtr.33004.1.S1_at	Hypothetical protein	-11.94
Mtr.2703.1.S1_at	Hypothetical protein	-11.91
Mtr.8575.1.S1_at	Glucosyltransferase-13	-11.91
Mtr.11791.1.S1_at	Hypothetical protein	-11.89
Msa.2771.1.S1_at	Hypothetical protein	-11.89
Mtr.44986.1.S1_at	Acyltransferase	-11.86
Mtr.10952.1.S1_at	Sulfate adenylyltransferase	-11.86
Mtr.33891.1.S1_at	Hypothetical protein	-11.85
Mtr.38356.1.S1_s_at	Hypothetical protein	-11.84
Mtr.5039.1.S1_at	Receptor protein	-11.80
Mtr.8715.1.S1_at	UDP-glucose glucosyltransferase	-11.79
Mtr.50874.1.S1_at	Hypothetical protein	-11.78
Mtr.34694.1.S1_at	Hypothetical protein	-11.77

Mtr.13898.1.S1_at	GPI-anchored protein	-11.77
Mtr.43527.1.S1_at	Hypothetical protein	-11.77
Mtr.34745.1.S1_at	Thylakoid lumenal 17.9 kDa protein	-11.76
Mtr.11073.1.S1_at	Short-chain dehydrogenase Tic32	-11.74
Mtr.18086.1.S1_at	RNA-directed DNA polymerase	-11.74
Mtr.21518.1.S1_s_at	Cytochrome P450	-11.73
Mtr.15420.1.S1_at	ABC transporter	-11.73
Mtr.44535.1.S1_at	ClpB heat shock protein	-11.73
Mtr.31456.1.S1_at	FRO1 and FRO2-like protein	-11.72
Mtr.33495.1.S1_at	Hypothetical protein	-11.71
Mtr.44539.1.S1_at	Ent-kaurenoic acid oxidase	-11.69
Mtr.1231.1.S1_s_at	Omega-6 fatty acid desaturase	-11.68
Sme.4919.1.S1_at	Hypothetical protein	-11.67
Mtr.47857.1.S1_at	Beta-amylase	-11.64
Mtr.44674.1.S1_at	Phytochrome A supressor spa1	-11.64
Msa.3133.1.S1_at	Hypothetical protein	-11.63
Mtr.51074.1.S1_at	Inorganic pyrophosphatase	-11.62
Mtr.8670.1.S1_at	Hypothetical protein	-11.62
Mtr.13080.1.S1_at	Hypothetical protein	-11.59
Mtr.33700.1.S1_at	CAX	-11.54
Mtr.41816.1.S1_at	Hypothetical protein	-11.49
Mtr.41407.1.S1_at	Hypothetical protein	-11.49
Mtr.24085.1.S1_at	Hypothetical protein	-11.49
Mtr.35444.1.S1_at	Oxygen evolving complex protein	-11.47
Mtr.5648.1.S1_s_at	Enhanced disease susceptibility 5	-11.47
Mtr.43833.1.S1_at	Hypothetical protein	-11.45
Mtr.28597.1.S1_at	Hypothetical protein	-11.45
Mtr.38250.1.S1_at	Hypothetical protein	-11.44
Mtr.8954.1.S1_at	Hypothetical protein	-11.44
Msa.1239.1.S1_at	Hypothetical protein	-11.43
Mtr.26941.1.S1_at	Hypothetical protein	-11.43
Mtr.12401.1.S1_s_at	Myb-related transcription factor MYB59	-11.43
Mtr.10301.1.S1_s_at	18S ribosomal RNA	-11.41
Msa.2667.1.S1_at	Hypothetical protein	-11.41
Mtr.51445.1.S1_x_at	Hypothetical protein	-11.38
Mtr.24591.1.S1_at	Hypothetical protein	-11.37
Mtr.8517.1.S1_at	Mannitol dehydrogenase	-11.31
Mtr.13436.1.S1_at	Nodule-enhanced protein phosphatase type 2C	-11.28
Mtr.17305.1.S1_at	Peptidase S4	-11.28
Mtr.40393.1.S1_s_at	MYB transcription factor	-11.28
Mtr.19422.1.S1_at	ParB-like nuclease	-11.28
Mtr.40232.1.S1_at	Geranylgeranyl hydrogenase	-11.27
Mtr.45404.1.S1_at	Glycine/D-amino acid oxidases	-11.26
Msa.2585.1.S1_at	Hypothetical protein	-11.24
Mtr.44131.1.S1_at	Hypothetical protein	-11.24
Msa.1080.1.S1_at	Hypothetical protein	-11.21
Mtr.41859.1.S1_at	FRO1 and FRO2-like protein	-11.20
Mtr.40391.1.S1_at	Hypothetical protein	-11.19
Mtr.2712.1.S1_at	Cyclin delta-3	-11.14
Mtr.46740.1.S1_at	Hypothetical protein	-11.12
Mtr.1814.1.S1_at	RING-H2 zinc finger protein	-11.11
Mtr.32128.1.S1_at	Hypothetical protein	-11.10
Mtr.10889.1.S1_at	Hypothetical protein	-11.09
Mtr.28238.1.S1_at	Subtilisin-like protease	-11.07

Mtr.43899.1.S1_at	Hydrolase	-11.06
Mtr.27410.1.S1_at	Hypothetical protein	-11.05
Mtr.18604.1.S1_at	Hypothetical protein	-11.02
Mtr.42306.1.S1_at	Hypothetical protein	-11.00
Mtr.17068.1.S1_at	WD-40 repeat protein	-10.99
Mtr.26042.1.S1_at	Hypothetical protein	-10.99
Mtr.7850.1.S1_s_at	Thaumatin-like protein	-10.98
Msa.1854.1.S1_at	Hypothetical protein	-10.97
Mtr.41565.1.S1_s_at	Hypothetical protein	-10.96
Mtr.39017.1.S1_at	Hypothetical protein	-10.96
Mtr.43350.1.S1_at	Hypothetical protein	-10.95
Mtr.39302.1.S1_at	Translation initiation factor IF-2	-10.95
Mtr.37780.1.S1_at	Alpha-glucan phosphorylase	-10.92
Mtr.9416.1.S1_at	Protease sppA	-10.92
Mtr.13420.1.S1_at	Selenium-binding protein	-10.90
Msa.981.1.S1_at	Hypothetical protein	-10.89
Mtr.8917.1.S1_at	Dehydrogenase	-10.89
Mtr.47901.1.S1_x_at	Glyceraldehyde-3-phosphate dehydrogenase A	-10.87
Mtr.13606.1.S1_at	Hypothetical protein	-10.87
Mtr.37855.1.S1_at	MAP kinase (Ser/Thr kinase)	-10.84
Mtr.13975.1.S1_at	Ycf23 protein	-10.83
Mtr.43498.1.S1_at	Hypothetical protein	-10.83
Mtr.19562.1.S1_at	Zn-finger, RING	-10.82
Mtr.20515.1.S1_at	Ferrochelatase	-10.81
Mtr.17600.1.S1_at	Protein kinase	-10.74
Mtr.25983.1.S1_s_at	Pectinesterase	-10.74
Mtr.46001.1.S1_at	Hypothetical protein	-10.73
Mtr.45397.1.S1_at	Ankyrin repeat-like protein	-10.73
Mtr.6746.1.S1_s_at	LEM3 (Ligand-effect modulator 3)	-10.72
Mtr.8402.1.S1_at	Hypothetical protein	-10.72
Mtr.18769.1.S1_at	Homeobox	-10.71
Mtr.27870.1.S1_at	Hypothetical protein	-10.71
Msa.2712.1.S1_at	Hypothetical protein	-10.69
Mtr.20822.1.S1_at	Hypothetical protein	-10.66
Mtr.39251.1.S1_at	Translation initiation factor IF-2	-10.66
Msa.935.1.S1_at	Hypothetical protein	-10.66
Mtr.35254.1.S1_at	Short chain dehydrogenase/reductase	-10.65
Msa.2885.1.S1_at	Hypothetical protein	-10.65
Mtr.22693.1.S1_s_at	Hypothetical protein	-10.64
Mtr.39104.1.S1_at	Xylulose kinase like protein	-10.63
Mtr.39425.1.S1_s_at	ATP-sulfurylase	-10.61
Mtr.45511.1.S1_at	Histidine kinase	-10.59
Mtr.9262.1.S1_at	Hsc70 protein	-10.59
Mtr.41614.1.S1_at	Hypothetical protein	-10.59
Mtr.47283.1.S1_at	Hypothetical protein	-10.58
Mtr.7832.1.S1_at	Hypothetical protein	-10.58
Mtr.8773.1.S1_s_at	Poly(A) polymerase	-10.57
Mtr.11556.1.S1_at	Carbohydrate oxidase	-10.57
Mtr.5135.1.S1_at	Protein kinase family	-10.55
Mtr.38712.1.S1_at	Hypothetical protein	-10.54
Mtr.39370.1.S1_at	Hypothetical protein	-10.54
Mtr.39821.1.S1_at	Membrane transporter	-10.53
Msa.1471.1.S1_at	Hypothetical protein	-10.52
Mtr.51258.1.S1_at	Annexin	-10.51

Mtr.11001.1.S1_at	Hypothetical protein	-10.50
Mtr.11129.1.S1_at	Hypothetical protein	-10.47
Mtr.41918.1.S1_at	Hypothetical protein	-10.47
Mtr.42982.1.S1_at	Carbonic anhydrase	-10.46
Mtr.22814.1.S1_at	Hypothetical protein	-10.46
Mtr.30958.1.S1_at	Hypothetical protein	-10.46
Mtr.12236.1.S1_at	Bundle sheath defective protein 2	-10.45
Mtr.34178.1.S1_s_at	Hypothetical protein	-10.44
Mtr.33532.1.S1_at	Kinesin heavy chain	-10.44
Mtr.47901.1.S1_at	Glyceraldehyde-3-phosphate dehydrogenase A	-10.44
Mtr.33889.1.S1_at	CER1 protein	-10.41
Mtr.15109.1.S1_at	Hypothetical protein	-10.40
Mtr.41157.1.S1_at	RAC-like GTP binding protein RAC13	-10.39
Msa.1850.1.S1_at	Hypothetical protein	-10.39
Mtr.42315.1.S1_at	Starch synthase isoform	-10.37
Mtr.28644.1.S1_at	Cytokinin receptor	-10.36
Mtr.45729.1.S1_s_at	ATP sulfurylase-related protein	-10.36
Mtr.32983.1.S1_at	Protein kinase	-10.35
Mtr.11495.1.S1_at	Steroid 5alpha-reductase-like protein	-10.34
Msa.1143.1.S1_s_at	Hypothetical protein	-10.33
Mtr.40710.1.S1_at	Malate dehydrogenase	-10.32
Mtr.11816.1.S1_at	Hypothetical protein	-10.31
Mtr.19992.1.S1_at	Phytochrome kinase substrate 1	-10.30
Mtr.47158.1.S1_at	Hypothetical protein	-10.27
Mtr.38724.1.S1_at	Hypothetical protein	-10.26
Mtr.42033.1.S1_at	Hypothetical protein	-10.25
Mtr.45039.1.S1_at	Peptidylprolyl isomerase	-10.25
Mtr.9046.1.S1_at	GAST1	-10.23
Mtr.38550.1.S1_at	Subtilisin-like proteinase	-10.23
Mtr.6551.1.S1_at	Hypothetical protein	-10.21
Mtr.16188.1.S1_at	Hypothetical protein	-10.19
Mtr.40683.1.S1_at	Photosystem II protein W-like protein	-10.19
Mtr.13054.1.S1_at	Wall-associated kinase	-10.17
Mtr.11332.1.S1_at	Hypothetical protein	-10.16
Mtr.41622.1.S1_at	Hypothetical protein	-10.16
Mtr.928.1.S1_at	Hypothetical protein	-10.15
Mtr.22603.1.S1_at	Glyceraldehyde-3-phosphate dehydrogenase A	-10.14
Mtr.37807.1.S1_at	Transporter	-10.12
Mtr.42265.1.S1_at	Transmembrane protein kinase	-10.12
Mtr.13393.1.S1_at	Hypothetical protein	-10.12
Mtr.11744.1.S1_at	Hypothetical protein	-10.12
Mtr.37345.1.S1_at	Subtilisin-like protease	-10.11
Mtr.9192.1.S1_at	Disease resistance protein	-10.11
Mtr.5655.1.S1_s_at	ADP-glucose pyrophosphorylase large subunit	-10.10
Mtr.39352.1.S1_at	Hypothetical protein	-10.10
Mtr.13330.1.S1_at	Hypothetical protein	-10.09
Msa.1307.1.S1_at	Hypothetical protein	-10.07
Mtr.20579.1.S1_at	Hypothetical protein	-10.06
Mtr.43627.1.S1_at	Hypothetical protein	-10.05
Mtr.34708.1.S1_at	Sulfolipid synthase	-10.04
Mtr.17233.1.S1_at	Hypothetical protein	-10.04
Mtr.12382.1.S1_at	Hypothetical protein	-10.04
Mtr.37550.1.S1_at	4-alpha-glucanotransferase	-10.04
Mtr.8588.1.S1_at	Hypothetical protein	-10.04

Mtr.48945.1.S1_s_at	Ferrochelatase	-10.02
Mtr.12448.1.S1_at	IAA-Ala hydrolase (IAR3)	-10.01
Mtr.18105.1.S1_at	Phosphoglycerate kinase	-10.00
Mtr.48351.1.S1_at	Proteinase inhibitor 20	-9.99
Msa.3102.1.S1_at	Hypothetical protein	-9.98
Mtr.44611.1.S1_at	Hypothetical protein	-9.98
Mtr.38403.1.S1_at	Thylakoid lumenal 25.6 kDa protein	-9.95
Mtr.7197.1.S1_at	Inositol 1,3,4-trisphosphate 5/6-kinase	-9.95
Mtr.50016.1.S1_at	Hypothetical protein	-9.94
Mtr.634.1.S1_at	Hypothetical protein	-9.91
Mtr.51188.1.S1_at	Glycine hydroxymethyltransferase	-9.87
Mtr.13458.1.S1_at	3(2),5-BISPHOSPHATE NUCLEOTIDASE	-9.87
Mtr.6728.1.S1_at	Cytochrome P-450	-9.86
Msa.3143.1.S1_at	Hypothetical protein	-9.86
Mtr.43359.1.S1_at	Phytochelatin synthetase	-9.81
Mtr.44198.1.S1_at	Hypothetical protein	-9.81
Mtr.40204.1.S1_at	Aldehyde dehydrogenase 1	-9.80
Mtr.9437.1.S1_at	Hypothetical protein	-9.80
Mtr.42210.1.S1_s_at	Two-component response regulator-like APRR3	-9.79
Mtr.42509.1.S1_at	Hypothetical protein	-9.79
Mtr.41265.1.S1_at	Glutathione S-transferase GST 10	-9.77
Mtr.11668.1.S1_at	Hypothetical protein	-9.76
Mtr.14453.1.S1_at	Rhodanese-like protein	-9.75
Mtr.40872.1.S1_at	Hypothetical protein	-9.74
Mtr.43762.1.S1_at	Hypothetical protein	-9.74
Mtr.19208.1.S1_at	Zn-finger, RING	-9.73
Mtr.47369.1.S1_at	ABC transporter	-9.73
Mtr.51423.1.S1_at	Sugar transporter	-9.72
Mtr.45551.1.S1_at	Hypothetical protein	-9.70
Mtr.16202.1.S1_at	Esterase/lipase/thioesterase	-9.69
Mtr.28675.1.S1_at	Polygalacturonase	-9.69
Mtr.9742.1.S1_at	Geminivirus replication protein-interacting protein	-9.69
Mtr.6694.1.S1_at	Na/Ca,K-exchanger-like protein	-9.68
Mtr.40926.1.S1_at	Thioredoxin-like protein	-9.65
Mtr.42672.1.S1_at	Hypothetical protein	-9.65
Mtr.44653.1.S1_at	Myb-related transcription activator	-9.65
Mtr.9686.1.S1_at	Beta-1,3-glucanase	-9.64
Mtr.36140.1.S1_at	Phosphoenolpyruvate carboxylase	-9.63
Mtr.12493.1.S1_at	Hypothetical protein	-9.63
Mtr.12821.1.S1_at	Phosphoglucomutase	-9.62
Msa.960.1.S1_at	Hypothetical protein	-9.61
Mtr.50122.1.S1_at	Hypothetical protein	-9.60
Mtr.25494.1.S1_at	Galactinol synthase	-9.59
Mtr.33572.1.S1_at	Glucose transporter	-9.56
Mtr.25331.1.S1_at	Glutamate receptor	-9.56
Mtr.43335.1.S1_at	4-coumarate:coenzyme A ligase	-9.55
Mtr.32947.1.S1_at	Hypothetical protein	-9.55
Mtr.4716.1.S1_at	Nitrite transport protein	-9.52
Mtr.12259.1.S1_at	Rubisco activase	-9.51
Mtr.13021.1.S1_at	Lustrin A-like protein	-9.48
Mtr.13843.1.S1_at	Hypothetical protein	-9.46
Mtr.39782.1.S1_at	Ppib protein	-9.44
Mtr.38392.1.S1_at	ClpB heat shock protein	-9.44
Mtr.38458.1.S1_at	FKBP-type peptidyl-prolyl cis-trans isomerase 2	-9.41

Mtr.12582.1.S1_at	Hypothetical protein	-9.41
Mtr.52214.1.S1_at	Hypothetical protein	-9.41
Mtr.46292.1.S1_at	Malate dehydrogenase	-9.40
Mtr.40392.1.S1_s_at	Circadian clock associated1	-9.40
Mtr.25945.1.S1_at	Myb-related	-9.39
Msa.1495.1.S1_at	Hypothetical protein	-9.36
Mtr.38080.1.S1_at	Hypothetical protein	-9.35
Mtr.525.1.S1_at	Hypothetical protein	-9.35
Mtr.33476.1.S1_at	Receptor protein kinase	-9.35
Msa.1805.1.S1_at	Hypothetical protein	-9.34
Mtr.33861.1.S1_at	Hypothetical protein	-9.33
Mtr.32529.1.S1_at	Transcription factor	-9.33
Mtr.8683.1.S1_at	Phosphoenolpyruvate carboxylase	-9.32
Mtr.49583.1.S1_at	Glycoside hydrolase	-9.31
Msa.2871.1.S1_at	Hypothetical protein	-9.31
Mtr.14312.1.S1_at	Hypothetical protein	-9.30
Mtr.7160.1.S1_at	ABC transporter	-9.28
Mtr.15414.1.S1_at	Hypothetical protein	-9.28
Mtr.12770.1.S1_at	Phosphoglucomutase	-9.27
Mtr.36889.1.S1_s_at	NIP3	-9.27
Mtr.8557.1.S1_s_at	MtN5 protein	-9.25
Mtr.30196.1.S1_at	S-adenosyl-L-methionine:salicylic acid carboxyl methyltransferase	-9.25
Mtr.37643.1.S1_at	NADP-dependent oxidoreductase P2	-9.23
Mtr.19310.1.S1_s_at	N-acetyl-gamma-glutamyl-phosphate reductase	-9.23
Mtr.27359.1.S1_at	Isoleucyl-tRNA synthetase	-9.23
Mtr.20397.1.S1_at	Amidase, hydantoinase/carbamoylase	-9.22
Mtr.45068.1.S1_at	Hypothetical protein	-9.22
Mtr.41202.1.S1_at	Hypothetical protein	-9.21
Mtr.45179.1.S1_at	Malate dehydrogenase	-9.21
Mtr.43181.1.S1_at	Thiamine biosynthesis protein	-9.20
Mtr.6132.1.S1_s_at	Subtilisin-like protein protease	-9.17
Mtr.15811.1.S1_at	Hypothetical protein	-9.16
Mtr.6059.1.S1_at	basic Helix Loop Helix protein	-9.16
Mtr.15585.1.S1_at	Copper/Zinc superoxide dismutase	-9.14
Mtr.35945.1.S1_at	UDP-glycose:flavonoid glycosyltransferase	-9.13
Mtr.32638.1.S1_s_at	Hypothetical protein	-9.13
Mtr.20946.1.S1_at	Serine/threonine protein kinase	-9.12
Mtr.48272.1.S1_at	Glycine dehydrogenase	-9.12
Mtr.37552.1.S1_at	RNA- or ssDNA-binding protein	-9.11
Mtr.33674.1.S1_s_at	Glutamyl-tRNA reductase 1	-9.10
Mtr.33011.1.S1_at	Copper transport protein	-9.09
Mtr.10088.1.S1_at	Hypothetical protein	-9.09
Mtr.32723.1.S1_at	Formiminotransferase-cyclodeaminase	-9.08
Msa.3192.1.S1_at	Hypothetical protein	-9.07
Mtr.49256.1.S1_at	Hypothetical protein	-9.07
Msa.3102.1.S1_x_at	Hypothetical protein	-9.06
Mtr.6869.1.S1_s_at	Glutamate receptor 3.6	-9.06
Mtr.22897.1.S1_at	Polygalacturonase	-9.06
Mtr.34700.1.S1_at	Tryptophan synthase beta chain 1	-9.05
Mtr.47706.1.S1_at	Myb family transcription factor	-9.04
Msa.1665.1.S1_at	Hypothetical protein	-9.04
Mtr.37839.1.S1_at	[4Fe-4S] cluster assembly factor	-9.03
Mtr.42261.1.S1_at	Rubisco activase	-9.02
Mtr.5714.1.S1_at	Hypothetical protein	-9.00

Mtr.26801.1.S1_a_at	Hypothetical protein	-9.00
Mtr.49274.1.S1_at	Cupredoxin	-8.99
Msa.989.1.S1_s_at	Hypothetical protein	-8.98
Mtr.3071.1.S1_at	Starch synthase	-8.98
Mtr.40160.1.S1_s_at	Pyrroline-5-carboxylate reductase	-8.97
Mtr.27055.1.S1_s_at	Plastid RNA polymerase	-8.97
Mtr.39530.1.S1_s_at	Hypothetical protein	-8.96
Mtr.46957.1.S1_at	Hypothetical protein	-8.94
Mtr.41940.1.S1_at	Hypothetical protein	-8.94
Msa.985.1.S1_at	Hypothetical protein	-8.93
Mtr.41447.1.S1_at	Hypothetical protein	-8.93
Mtr.8133.1.S1_at	Hypothetical protein	-8.92
Mtr.40620.1.S1_at	Hypothetical protein	-8.92
Mtr.929.1.S1_s_at	Hypothetical protein	-8.91
Mtr.46511.1.S1_x_at	Alpha/beta-amyrin synthase	-8.90
Mtr.40963.1.S1_at	En/Spm-like transposon protein	-8.90
Msa.1377.1.S1_at	Hypothetical protein	-8.90
Mtr.1268.1.S1_at	Hypothetical protein	-8.89
Mtr.20646.1.S1_s_at	Glucose/ribitol dehydrogenase	-8.88
Mtr.12358.1.S1_at	Seed maturation protein LEA 4	-8.87
Mtr.37298.1.S1_at	Cytochrome P450	-8.84
Msa.2727.1.S1_at	Hypothetical protein	-8.83
Mtr.25367.1.S1_at	12-oxophytodienoic acid 10, 11-reductase	-8.80
Mtr.2077.1.S1_at	Hypothetical protein	-8.80
Mtr.3805.1.S1_at	Laccase	-8.78
Mtr.41958.1.S1_at	Hypothetical protein	-8.78
Msa.2888.1.S1_at	Hypothetical protein	-8.77
Mtr.28817.1.S1_at	Hypothetical protein	-8.77
Msa.2550.1.S1_at	Hypothetical protein	-8.76
Msa.2653.1.S1_at	Hypothetical protein	-8.75
Mtr.10039.1.S1_at	Hypothetical protein	-8.75
Mtr.9876.1.S1_at	Nuclear transcription factor Y	-8.74
Mtr.41868.1.S1_at	Tryptophan synthase beta chain 1	-8.74
Mtr.48721.1.S1_at	Cytochrome c	-8.73
Msa.1910.1.S1_at	Hypothetical protein	-8.72
Mtr.28166.1.S1_s_at	Dihydrolipoamide Dehydrogenase	-8.72
Mtr.35488.1.S1_at	Hypothetical protein	-8.72
Mtr.12308.1.S1_at	Photosystem I reaction center subunit psaK	-8.69
Mtr.25657.1.S1_at	Cell-wall P4 protein	-8.69
Mtr.9418.1.S1_s_at	Pathogenesis-related protein	-8.69
Mtr.35142.1.S1_at	Hypothetical protein	-8.66
Mtr.41702.1.S1_at	Hypothetical protein	-8.65
Mtr.15561.1.S1_at	GCN5-related N-acetyltransferase	-8.64
Mtr.40734.1.S1_at	Glucosyltransferase-14	-8.63
Msa.1297.1.S1_at	Hypothetical protein	-8.63
Mtr.19267.1.S1_at	Photosystem I protein PsaD	-8.61
Mtr.47977.1.S1_at	Nodule-specific protein	-8.61
Mtr.6717.1.S1_at	Acyl-activating enzyme 14	-8.60
Mtr.6746.1.S1_at	LEM3 (Ligand-effect modulator 3)	-8.59
Mtr.52053.1.S1_at	Ribosomal protein L35	-8.57
Mtr.6869.1.S1_at	Glutamate receptor 3.6	-8.56
Mtr.48911.1.S1_at	Short-chain dehydrogenase/reductase	-8.56
Mtr.27223.1.S1_s_at	Hypothetical protein	-8.54
Mtr.52150.1.S1_at	UTP--glucose-1-phosphate uridylyltransferase	-8.53

Mtr.1885.1.S1_at	Symbiotic ammonium transporter	-8.53
Mtr.24793.1.S1_at	Blight resistance protein RGA1	-8.53
Mtr.12273.1.S1_s_at	Ribulose 1,5-bisphosphate carboxylase small subunit	-8.52
Mtr.44543.1.S1_at	Transmembrane transport protein	-8.52
Mtr.41590.1.S1_at	Hypothetical protein	-8.51
Mtr.9534.1.S1_at	Hypothetical protein	-8.51
Mtr.37898.1.S1_at	Short-chain dehydrogenase Tic32	-8.49
Mtr.43727.1.S1_at	Squalene monooxygenase 1	-8.49
Mtr.10393.1.S1_at	Thiamin biosynthetic enzyme	-8.47
Mtr.11416.1.S1_at	HMG-box transcription factor 3	-8.46
Mtr.6733.1.S1_at	Rhomboid family protein	-8.46
Mtr.11529.1.S1_at	SOUL heme-binding protein	-8.46
Mtr.41414.1.S1_at	Hypothetical protein	-8.45
Mtr.47452.1.S1_at	Isoflavone 2'-hydroxylase	-8.44
Mtr.6155.1.S1_at	Protein kinase	-8.44
Mtr.3858.1.S1_at	Leucoanthocyanidin dioxygenase	-8.42
Msa.1069.1.S1_at	Hypothetical protein	-8.42
Mtr.40761.1.S1_at	50S ribosomal protein L11	-8.42
Msa.1655.1.S1_at	Hypothetical protein	-8.41
Mtr.40799.1.S1_at	Subtilisin-like protease	-8.41
Mtr.32760.1.S1_at	Hypothetical protein	-8.41
Msa.3120.1.S1_at	Hypothetical protein	-8.40
Mtr.34741.1.S1_at	3(2),5-BISPHOSPHATE NUCLEOTIDASE	-8.39
Mtr.40514.1.S1_at	Ultraviolet-B-repressible protein	-8.38
Mtr.42164.1.S1_at	Hypothetical protein	-8.38
Mtr.38984.1.S1_at	Hypothetical protein	-8.36
Mtr.39735.1.S1_at	Hypothetical protein	-8.36
Mtr.27111.1.S1_at	GTPase engC protein 1	-8.35
Mtr.42914.1.S1_x_at	Ribulose 1,5-bisphosphate carboxylase small subunit	-8.34
Msa.2847.1.S1_at	Hypothetical protein	-8.33
Mtr.8772.1.S1_at	Poly(A) polymerase	-8.32
Mtr.9162.1.S1_at	Carboxypeptidase type III	-8.32
Mtr.16226.1.S1_at	Serine/threonine protein kinase	-8.30
Msa.1349.1.S1_at	Hypothetical protein	-8.30
Mtr.33765.1.S1_s_at	Hypothetical protein	-8.30
Mtr.20818.1.S1_at	Xylulose kinase	-8.30
Mtr.40347.1.S1_at	Omega-3 fatty acid desaturase	-8.29
Mtr.33632.1.S1_s_at	Hypothetical protein	-8.29
Mtr.43393.1.S1_at	Nicotianamine synthase	-8.29
Mtr.2111.1.S1_at	Hypothetical protein	-8.28
Mtr.33426.1.S1_at	MRP-like ABC transporter	-8.27
Mtr.27827.1.S1_at	Hypothetical protein	-8.26
Mtr.7520.1.S1_at	Hypothetical protein	-8.26
Mtr.12555.1.S1_at	Alpha-glucan phosphorylase	-8.26
Mtr.50904.1.S1_at	Hypothetical protein	-8.25
Mtr.37420.1.S1_at	Glycine cleavage system H protein	-8.25
Mtr.33529.1.S1_at	Hypothetical protein	-8.24
Mtr.37749.1.S1_at	Hypothetical protein	-8.24
Mtr.34738.1.S1_s_at	Copper/zinc superoxide dismutase	-8.24
Msa.1844.1.S1_at	Hypothetical protein	-8.22
Mtr.40814.1.S1_at	Chlorophyll synthetase	-8.21
Mtr.10485.1.S1_at	Glucose-6-phosphate isomerase	-8.19
Mtr.12039.1.S1_at	Peptide transporter 1	-8.16
Mtr.24731.1.S1_at	Hypothetical protein	-8.15

Mtr.44491.1.S1_at	Hypothetical protein	-8.14
Msa.858.1.S1_at	Hypothetical protein	-8.13
Mtr.40715.1.S1_at	Mercuric reductase	-8.11
Mtr.45935.1.S1_at	Hypothetical protein	-8.10
Mtr.45384.1.S1_at	Hypothetical protein	-8.09
Mtr.20229.1.S1_at	UDP-glucose:anthocyanin 5-O-glucosyltransferase	-8.08
Mtr.4948.1.S1_at	ABC transporter	-8.08
Mtr.39387.1.S1_at	DCL protein	-8.08
Mtr.8410.1.S1_at	Sulfate transporter	-8.07
Mtr.49461.1.S1_at	Hypothetical protein	-8.07
Mtr.12374.1.S1_at	Protochlorophyllide reductase	-8.07
Mtr.40321.1.S1_at	Hydroperoxide lyase	-8.07
Mtr.29849.1.S1_at	Hypothetical protein	-8.07
Mtr.35625.1.S1_s_at	Hypothetical protein	-8.06
Mtr.32661.1.S1_at	Hypothetical protein	-8.05
Msa.1161.1.S1_at	Hypothetical protein	-8.05
Mtr.38869.1.S1_at	Hypothetical protein	-8.03
Mtr.45313.1.S1_at	Multidrug resistance-associated protein 2	-8.03
Mtr.44782.1.S1_at	Hypothetical protein	-8.03
Mtr.13059.1.S1_at	Hypothetical protein	-8.02
Mtr.17765.1.S1_at	TGF-beta receptor	-8.02
Mtr.40978.1.S1_at	Hypothetical protein	-8.01
Msa.3118.1.S1_at	Hypothetical protein	-8.00
Mtr.43060.1.S1_at	GTP-binding protein	-8.00
Mtr.12258.1.S1_at	Nonspecific lipid-transfer protein precursor (LTP)	-7.99
Mtr.41860.1.S1_at	Leucine-rich repeat receptor-like protein kinase 1	-7.98
Mtr.48501.1.S1_at	Leucine-rich repeat	-7.97
Mtr.10881.1.S1_at	Hypothetical protein	-7.97
Msa.1765.1.S1_at	Hypothetical protein	-7.95
Mtr.8920.1.S1_at	2-oxoglutarate/malate translocator	-7.94
Mtr.44760.1.S1_at	Sulfolipid synthase	-7.94
Mtr.42492.1.S1_at	Carboxylesterase-like protein	-7.92
Mtr.19934.1.S1_at	Cysteine proteinase	-7.91
Mtr.12672.1.S1_at	Cytochrome P-450LXXIA1	-7.91
Mtr.16355.1.S1_at	HSP20-like chaperone	-7.90
Mtr.9712.1.S1_at	Copper/zinc superoxide dismutase	-7.89
Mtr.43316.1.S1_at	Symbiotic ammonium transporter	-7.88
Mtr.49015.1.S1_at	Chlorophyll A-B binding protein	-7.87
Mtr.41408.1.S1_at	Hypothetical protein	-7.87
Msa.1547.1.S1_at	Hypothetical protein	-7.86
Mtr.51462.1.S1_s_at	Zn-finger, RING	-7.86
Msa.3103.1.S1_at	Hypothetical protein	-7.86
Mtr.11698.1.S1_at	Hypothetical protein	-7.85
Mtr.15871.1.S1_at	Hypothetical protein	-7.84
Mtr.49075.1.S1_at	Peptidase aspartic	-7.83
Mtr.21341.1.S1_at	Glutamyl-tRNA reductase	-7.83
Mtr.18021.1.S1_at	Hypothetical protein	-7.82
Mtr.7552.1.S1_at	Hypothetical protein	-7.80
Msa.1517.1.S1_at	Hypothetical protein	-7.80
Mtr.37322.1.S1_at	Hypothetical protein	-7.80
Mtr.5768.1.S1_at	Hypothetical protein	-7.80
Mtr.15326.1.S1_at	ABA/WDS induced protein	-7.79
Mtr.18566.1.S1_at	Pyruvate kinase	-7.79
Mtr.43018.1.S1_at	Cytochrome P450	-7.79

Mtr.6086.1.S1_s_at	Sesquiterpene cyclase	-7.78
Mtr.24929.1.S1_at	Hypothetical protein	-7.78
Mtr.16579.1.S1_x_at	Cytochrome P450	-7.77
Mtr.48959.1.S1_at	Flavoprotein pyridine nucleotide cytochrome reductase	-7.77
Mtr.41227.1.S1_at	Germacrene D synthase	-7.77
Mtr.31169.1.S1_at	Cytochrome P450	-7.76
Mtr.29995.1.S1_at	Hypothetical protein	-7.76
Mtr.9757.1.S1_at	Quinolinate phosphoribosyltransferase	-7.75
Mtr.43061.1.S1_s_at	GTP-binding protein	-7.75
Mtr.3511.1.S1_at	Anthranilate N-hydroxycinnamoyl/benzoyltransferase	-7.75
Mtr.49684.1.S1_at	UDP-glucose glucosyltransferase	-7.74
Mtr.40327.1.S1_at	ADP-glucose pyrophosphorylase	-7.74
Mtr.34803.1.S1_at	Glutathione S-conjugate transporting ATPase	-7.73
Mtr.15289.1.S1_at	Lipoxygenase	-7.73
Mtr.34390.1.S1_at	Hypothetical protein	-7.73
Mtr.27269.1.S1_at	Hypothetical protein	-7.73
Mtr.43912.1.S1_at	Hypothetical protein	-7.72
Mtr.40528.1.S1_s_at	UOS1	-7.71
Mtr.41573.1.S1_at	Double C2-like domain containing protein	-7.70
Msa.1640.1.S1_at	Hypothetical protein	-7.70
Msa.1150.1.S1_at	Hypothetical protein	-7.70
Mtr.15885.1.S1_at	Hypothetical protein	-7.70
Mtr.12260.1.S1_at	2-Cys peroxiredoxin	-7.69
Mtr.31153.1.S1_at	Hypothetical protein	-7.67
Mtr.39289.1.S1_at	Flavonoid 1-2 rhamnosyltransferase	-7.66
Mtr.33844.1.S1_s_at	Allantoinase	-7.65
Mtr.19902.1.S1_at	Isochorismate synthases	-7.64
Mtr.44672.1.S1_at	Glutathione S-transferase GST 7	-7.64
Mtr.9818.1.S1_at	Hypothetical protein	-7.63
Mtr.11082.1.S1_at	Perchloric acid soluble translation inhibitor protein	-7.62
Mtr.13744.1.S1_at	Transmembrane transport protein	-7.61
Mtr.25951.1.S1_at	Hypothetical protein	-7.60
Mtr.18790.1.S1_at	Esterase/lipase/thioesterase	-7.60
Mtr.11025.1.S1_at	Hypothetical protein	-7.59
Mtr.42279.1.S1_at	Protease Do-like 8	-7.59
Mtr.27368.1.S1_at	Hypothetical protein	-7.57
Mtr.1762.1.S1_at	Plastid RNA polymerase sigma-subunit	-7.56
Mtr.44182.1.S1_s_at	LEA protein in group 5	-7.55
Msa.1763.1.S1_at	Hypothetical protein	-7.55
Mtr.2990.1.S1_at	Metal resistance protein	-7.54
Mtr.40152.1.S1_at	Oxygen-evolving enhancer protein 2	-7.53
Mtr.26195.1.S1_at	Hypothetical protein	-7.53
Mtr.30845.1.S1_at	Nodulin-like protein	-7.53
Mtr.40730.1.S1_at	Thylakoid lumenal 16.5 kDa protein	-7.53
Mtr.38437.1.S1_at	Hypothetical protein	-7.52
Mtr.42842.1.S1_s_at	Ribulose 1,5-bisphosphate carboxylase small subunit	-7.52
Mtr.5330.1.S1_at	Serine/threonine protein kinase	-7.51
Mtr.26124.1.S1_at	Hypothetical protein	-7.50
Mtr.37166.1.S1_at	UDP-glucose:indole-3-acetate beta-D-glucosyltransferase	-7.50
Mtr.13956.1.S1_at	2-oxoglutarate/malate translocator	-7.50
Mtr.5357.1.S1_at	ABC transporter	-7.49
Mtr.38608.1.S1_at	Hypothetical protein	-7.49
Mtr.1747.1.S1_at	Hypothetical protein	-7.49
Mtr.31624.1.S1_at	Hypothetical protein	-7.48

Mtr.37792.1.S1_at	Hypothetical protein	-7.47
Mtr.6825.1.S1_at	Hypothetical protein	-7.46
Mtr.37744.1.S1_at	Rieske iron-sulfur protein	-7.46
Mtr.5404.1.S1_at	Receptor protein kinase	-7.45
Mtr.1779.1.S1_at	Hypothetical protein	-7.45
Mtr.1036.1.S1_at	Non-phototropic hypocotyl 3	-7.44
Mtr.43567.1.S1_at	Dimethylaniline monooxygenase	-7.44
Mtr.41524.1.S1_at	Sulfate transporter	-7.43
Msa.2593.1.S1_at	Hypothetical protein	-7.43
Mtr.13396.1.S1_at	Hypothetical protein	-7.43
Mtr.22277.1.S1_s_at	Hypothetical protein	-7.41
Msa.1528.1.S1_at	Hypothetical protein	-7.41
Mtr.50437.1.S1_s_at	Hypothetical protein	-7.40
Msa.3052.1.S1_at	Hypothetical protein	-7.40
Mtr.10136.1.S1_at	Hypothetical protein	-7.39
Mtr.1911.1.S1_s_at	Hypothetical protein	-7.39
Mtr.12188.1.S1_at	Hypothetical protein	-7.38
Mtr.2057.1.S1_s_at	Glutamate/malate translocator	-7.38
Mtr.9168.1.S1_at	Hypothetical protein	-7.37
Mtr.50743.1.S1_at	Hypothetical protein	-7.36
Mtr.45291.1.S1_at	Hypothetical protein	-7.36
Mtr.45562.1.S1_at	Nuclear shuttle interacting protein	-7.36
Mtr.42253.1.S1_at	Hypothetical protein	-7.35
Msa.2813.1.S1_at	Hypothetical protein	-7.35
Mtr.40869.1.S1_at	Hypothetical protein	-7.35
Mtr.49172.1.S1_at	Hypothetical protein	-7.34
Mtr.31750.1.S1_at	Sulfate transporter	-7.34
Mtr.28722.1.S1_at	Peptide transporter-like protein	-7.34
Mtr.13528.1.S1_at	Anthranilate N-hydroxycinnamoyl/benzoyltransferase	-7.34
Mtr.41106.1.S1_at	Hypothetical protein	-7.33
Msa.2963.1.S1_at	Hypothetical protein	-7.33
Mtr.45371.1.S1_at	Small signal peptidase	-7.33
Mtr.45476.1.S1_at	Metal-transporting P-type ATPase	-7.33
Msa.1766.1.S1_at	Hypothetical protein	-7.33
Mtr.34167.1.S1_at	Hypothetical protein	-7.32
Mtr.49627.1.S1_at	Hypothetical protein	-7.32
Mtr.43533.1.S1_at	Maturation polypeptide	-7.31
Mtr.8219.1.S1_at	Hypothetical protein	-7.31
Mtr.2640.1.S1_at	Pullulanase-like protein	-7.30
Mtr.37814.1.S1_at	CONSTANS-like protein	-7.30
Mtr.38747.1.S1_s_at	Sugar transporter	-7.28
Mtr.4407.1.S1_at	Transcription factor, CCAAT-binding	-7.28
Mtr.40379.1.S1_at	Leucoanthocyanidin dioxygenase	-7.27
Mtr.15484.1.S1_at	Peroxidase	-7.27
Mtr.33854.1.S1_at	Hypothetical protein	-7.27
Mtr.46061.1.S1_s_at	Dynamin GTPase effector	-7.26
Mtr.37767.1.S1_at	Hypothetical protein	-7.25
Msa.1320.1.S1_at	Hypothetical protein	-7.25
Mtr.15409.1.S1_at	Protein prenyltransferase	-7.25
Mtr.15245.1.S1_at	ABC transporter	-7.25
Mtr.12256.1.S1_at	Nonspecific lipid-transfer protein precursor (LTP)	-7.25
Mtr.51379.1.S1_at	Basic helix-loop-helix	-7.24
Mtr.48548.1.S1_at	Deoxyxylulose-5-phosphate synthase	-7.23
Msa.1062.1.S1_at	Hypothetical protein	-7.23

Mtr.17441.1.S1_at	Glycoside hydrolase	-7.21
Msa.1741.1.S1_at	Hypothetical protein	-7.20
Mtr.12602.1.S1_at	Mitogen-activated protein kinase	-7.20
Mtr.39163.1.S1_at	Hypothetical protein	-7.20
Mtr.24061.1.S1_at	Hypothetical protein	-7.20
Mtr.43091.1.S1_s_at	Seed maturation protein LEA 4	-7.20
Msa.1561.1.S1_at	Hypothetical protein	-7.20
Mtr.37745.1.S1_at	Hypothetical protein	-7.17
Mtr.9270.1.S1_at	Hypothetical protein	-7.17
Mtr.44282.1.S1_s_at	THA4 protein	-7.17
Mtr.43079.1.S1_at	Farnesyl-diphosphate farnesyltransferase	-7.17
Mtr.31623.1.S1_at	Hypothetical protein	-7.16
Mtr.40190.1.S1_at	Flowering promoting factor	-7.16
Mtr.22096.1.S1_at	EIX receptor 1	-7.15
Mtr.5844.1.S1_at	Ternary complex factor MIP1-like protein	-7.15
Mtr.20397.1.S1_s_at	Amidase, hydantoinase/carbamoylase	-7.14
Mtr.7329.1.S1_at	Ribulose-1,5-bisphosphate carboxylase small subunit	-7.14
Mtr.42820.1.S1_at	RING/C3HC4/PHD zinc finger protein	-7.14
Mtr.32860.1.S1_s_at	Hypothetical protein	-7.14
Mtr.48535.1.S1_at	Hypothetical protein	-7.13
Mtr.44533.1.S1_at	Hypothetical protein	-7.13
Mtr.39390.1.S1_at	Phosphoenolpyruvate carboxylase	-7.12
Mtr.42326.1.S1_s_at	Hypothetical protein	-7.11
Mtr.5845.1.S1_at	Hypothetical protein	-7.11
Msa.1109.1.S1_at	Hypothetical protein	-7.11
Mtr.11185.1.S1_at	SEX1	-7.10
Mtr.11562.1.S1_at	Diphosphonucleotide phosphatase 1	-7.10
Mtr.41412.1.S1_at	Thylakoid lumenal 18 kDa protein	-7.09
Mtr.30722.1.S1_at	Hypothetical protein	-7.08
Mtr.24070.1.S1_at	Plastid-lipid associated protein	-7.08
Mtr.43633.1.S1_at	Hypothetical protein	-7.08
Mtr.12063.1.S1_at	Hypothetical protein	-7.07
Mtr.11016.1.S1_at	Hypothetical protein	-7.07
Mtr.41145.1.S1_at	Peroxisomal copper-containing amine oxidase	-7.07
Mtr.2611.1.S1_at	Protein kinase	-7.06
Mtr.40149.1.S1_at	Thiazole biosynthetic enzyme	-7.06
Mtr.12725.1.S1_at	Mucin-like protein	-7.06
Mtr.11931.1.S1_at	Long chain acyl-CoA synthetase	-7.06
Mtr.47780.1.S1_at	Expansin, putative	-7.05
Mtr.12846.1.S1_at	MAP3K	-7.05
Mtr.34485.1.S1_at	Hypothetical protein	-7.05
Msa.2820.1.S1_at	Hypothetical protein	-7.05
Mtr.848.1.S1_s_at	Hypothetical protein	-7.05
Mtr.42585.1.S1_at	Hypothetical protein	-7.04
Mtr.37783.1.S1_at	Epoxide hydrolase	-7.04
Mtr.18312.1.S1_at	Hypothetical protein	-7.02
Mtr.10402.1.S1_at	Type II chlorophyll a/b binding protein from photosystem I	-7.01
Mtr.8942.1.S1_at	Desiccation protectant protein Lea14	-7.01
Mtr.6756.1.S1_at	ABC transporter	-7.00
Msa.3105.1.S1_at	Hypothetical protein	-7.00
Mtr.10207.1.S1_at	Early E3 20.6 kDa glycoprotein	-6.99
Mtr.46949.1.S1_at	Hypothetical protein	-6.98
Mtr.42902.1.S1_at	VuP5CS protein	-6.97
Mtr.35532.1.S1_at	Bg55 protein	-6.97

Mtr.9264.1.S1_at	Hypothetical protein	-6.97
Mtr.13374.1.S1_at	Protein kinase	-6.97
Mtr.49977.1.S1_at	Hypothetical protein	-6.96
Mtr.41089.1.S1_at	Hypothetical protein	-6.96
Mtr.43655.1.S1_at	Glucosyltransferase	-6.96
Mtr.15886.1.S1_at	Hypothetical protein	-6.96
Mtr.35696.1.S1_at	Hypothetical protein	-6.95
Mtr.44854.1.S1_at	Hypothetical protein	-6.94
Mtr.41481.1.S1_at	Phosphate translocator	-6.94
Mtr.40480.1.S1_at	Allantoinase	-6.94
Mtr.27314.1.S1_s_at	Heat shock transcription factor	-6.94
Mtr.13413.1.S1_at	Transporter like protein	-6.94
Mtr.18237.1.S1_at	Hypothetical protein	-6.92
Mtr.38446.1.S1_s_at	Ferredoxin-thioredoxin reductase	-6.92
Mtr.6771.1.S1_s_at	Hypothetical protein	-6.92
Mtr.50224.1.S1_s_at	FYVE/PHD zinc finger	-6.92
Mtr.31392.1.S1_s_at	Desiccation protectant protein	-6.92
Mtr.43148.1.S1_at	Myb-related transcription factor MYB59	-6.92
Mtr.33613.1.S1_at	Hypothetical protein	-6.91
Mtr.39411.1.S1_at	Sulfate transporter 3.3	-6.90
Mtr.39699.1.S1_at	Hypothetical protein	-6.89
Mtr.27055.1.S1_at	Plastid RNA polymerase	-6.88
Mtr.43037.1.S1_at	Glyoxysomal malate dehydrogenase	-6.87
Mtr.36996.1.S1_at	Hypothetical protein	-6.86
Mtr.42326.1.S1_at	Hypothetical protein	-6.85
Mtr.44150.1.S1_at	Myristoyl-acyl carrier protein thioesterase	-6.84
Mtr.37518.1.S1_at	Nodulin-like protein	-6.84
Mtr.41151.1.S1_s_at	Cer2 protein	-6.84
Mtr.33903.1.S1_s_at	GTP-binding protein	-6.84
Msa.1776.1.S1_at	Hypothetical protein	-6.83
Mtr.37624.1.S1_at	3-beta-hydroxysteroiddehydrogenase	-6.83
Mtr.12899.1.S1_at	Hypothetical protein	-6.83
Mtr.47758.1.S1_at	bHLH protein family	-6.82
Mtr.33055.1.S1_s_at	Hypothetical protein	-6.82
Mtr.14004.1.S1_at	Hypothetical protein	-6.81
Mtr.45016.1.S1_at	Hypothetical protein	-6.80
Mtr.44793.1.S1_at	Hypothetical protein	-6.80
Mtr.11186.1.S1_at	Pyruvate, phosphate dikinase	-6.80
Mtr.11829.1.S1_at	Hypothetical protein	-6.80
Mtr.12884.1.S1_at	Thioredoxin x	-6.80
Mtr.38557.1.S1_at	MAP kinase protein	-6.79
Mtr.43358.1.S1_at	Outer membrane lipoprotein	-6.79
Mtr.33417.1.S1_at	MRP-like ABC transporter	-6.79
Mtr.43087.1.S1_at	Pectin methylesterase	-6.78
Mtr.18020.1.S1_at	Hypothetical protein	-6.78
Msa.1177.1.S1_at	Hypothetical protein	-6.78
Mtr.13801.1.S1_at	Hypothetical protein	-6.78
Mtr.5313.1.S1_s_at	Hypothetical protein	-6.78
Mtr.6780.1.S1_at	Starch synthase IVb	-6.78
Mtr.12203.1.S1_at	Ribulose 1,6-bisphosphate carboxylase small subunit	-6.77
Mtr.24695.1.S1_at	NADH dehydrogenase	-6.77
Mtr.24717.1.S1_s_at	Arabinogalactan protein	-6.77
Mtr.42733.1.S1_at	Histone H1	-6.76
Mtr.10799.1.S1_at	Multi resistance protein	-6.76

Msa.2782.1.S1_at	Hypothetical protein	-6.75
Mtr.38066.1.S1_at	Cinnamyl alcohol dehydrogenase	-6.75
Msa.1744.1.S1_at	Hypothetical protein	-6.74
Msa.1508.1.S1_at	Hypothetical protein	-6.74
Mtr.34597.1.S1_s_at	Hypothetical protein	-6.74
Mtr.45956.1.S1_at	Lipase/lipooxygenase	-6.73
Mtr.8818.1.S1_at	Hypothetical protein	-6.73
Mtr.37429.1.S1_at	Cytosolic aldehyde dehydrogenase	-6.72
Msa.1808.1.S1_at	Hypothetical protein	-6.72
Mtr.42608.1.S1_at	Polysaccharide polymerase	-6.71
Mtr.44836.1.S1_at	Hypothetical protein	-6.71
Mtr.10595.1.S1_s_at	Seed maturation protein PM36	-6.70
Msa.900.1.S1_s_at	Hypothetical protein	-6.69
Mtr.34747.1.S1_at	Hypothetical protein	-6.69
Mtr.12983.1.S1_at	Isp4 like protein	-6.69
Msa.3170.1.S1_at	Hypothetical protein	-6.68
Mtr.37811.1.S1_at	Hypothetical protein	-6.68
Mtr.11408.1.S1_at	Hypothetical protein	-6.67
Mtr.6141.1.S1_s_at	Alpha-expansin	-6.66
Mtr.32580.1.S1_at	Alpha-mannosidase	-6.66
Mtr.16218.1.S1_s_at	Homeodomain-like protein	-6.65
Msa.1421.1.S1_at	Hypothetical protein	-6.64
Mtr.12779.1.S1_at	Photosystem II stability/assembly factor HCF136	-6.63
Mtr.21614.1.S1_at	Hypothetical protein	-6.63
Mtr.10685.1.S1_at	Hypothetical protein	-6.63
Mtr.29366.1.S1_at	Serine/threonine protein kinase	-6.62
Mtr.39748.1.S1_at	Hypothetical protein	-6.62
Mtr.9805.1.S1_at	Hypothetical protein	-6.61
Mtr.37156.1.S1_at	Hypothetical protein	-6.61
Mtr.11795.1.S1_at	Proline-rich protein	-6.60
Mtr.47648.1.S1_at	Hypothetical protein	-6.59
Mtr.34797.1.S1_at	Hypothetical protein	-6.59
Mtr.40422.1.S1_at	Dihydrolipoyl dehydrogenase	-6.59
Mtr.41047.1.S1_at	Hypothetical protein	-6.59
Mtr.6035.1.S1_at	UDP-glycosyltransferase 74G1	-6.58
Mtr.13053.1.S1_s_at	Urophorphyrin III methylase	-6.58
Mtr.38938.1.S1_at	Hypothetical protein	-6.58
Mtr.2641.1.S1_at	Hypothetical protein	-6.57
Msa.2926.1.S1_s_at	Hypothetical protein	-6.56
Mtr.39327.1.S1_at	Hypothetical protein	-6.56
Mtr.10011.1.S1_at	Hypothetical protein	-6.55
Mtr.31127.1.S1_s_at	ERD4 protein	-6.54
Mtr.39005.1.S1_at	Nitrate transporter	-6.53
Mtr.30134.1.S1_at	Serine carboxipeptidase	-6.52
Mtr.40443.1.S1_at	50S ribosomal protein L13	-6.51
Mtr.33027.1.S1_at	Hypothetical protein	-6.51
Mtr.24210.1.S1_at	Hypothetical protein	-6.51
Mtr.22990.1.S1_at	Receptor-like protein kinase 1	-6.50
Mtr.44017.1.S1_at	Seed maturation protein	-6.50
Mtr.49627.1.S1_x_at	Hypothetical protein	-6.50
Mtr.45049.1.S1_at	Hypothetical protein	-6.49
Mtr.9169.1.S1_at	Hypothetical protein	-6.49
Mtr.34752.1.S1_s_at	Hypothetical protein	-6.49
Mtr.44524.1.S1_at	Hypothetical protein	-6.49

Mtr.52193.1.S1_at	Glycoside hydrolase	-6.49
Mtr.33453.1.S1_at	Hypothetical protein	-6.48
Mtr.40236.1.S1_s_at	Hypothetical protein	-6.47
Mtr.21678.1.S1_at	YABBY protein	-6.47
Mtr.16894.1.S1_at	Zn-finger, CCHC type	-6.46
Mtr.50932.1.S1_at	Myb, DNA-binding;	-6.46
Msa.1072.1.S1_at	Hypothetical protein	-6.46
Msa.1845.1.S1_at	Hypothetical protein	-6.46
Mtr.18280.1.S1_at	Serine/threonine protein kinase	-6.45
Mtr.5510.1.S1_at	Hypothetical protein	-6.44
Msa.925.1.S1_at	Hypothetical protein	-6.44
Mtr.15923.1.S1_at	Peptidylprolyl isomerase, FKBP-type	-6.43
Mtr.32475.1.S1_s_at	Hypothetical protein	-6.43
Mtr.1753.1.S1_at	Hypothetical protein	-6.41
Mtr.34827.1.S1_at	Hypothetical protein	-6.41
Mtr.39433.1.S1_at	Xyloglucan endotransglucosylase/hydrolase protein	-6.41
Mtr.33894.1.S1_at	Tetracycline transporter	-6.41
Mtr.41485.1.S1_at	Dynamin-like protein	-6.41
Mtr.48197.1.S1_at	Hypothetical protein	-6.40
Mtr.43132.1.S1_at	CND41, chloroplast nucleoid DNA binding protein	-6.40
Mtr.37673.1.S1_at	Hypothetical protein	-6.39
Mtr.2078.1.S1_at	Cell death regulator	-6.39
Mtr.44745.1.S1_at	Ferredoxin	-6.38
Mtr.41314.1.S1_at	Pyruvate, phosphate dikinase	-6.38
Mtr.44090.1.S1_at	Hypothetical protein	-6.38
Mtr.28080.1.S1_at	Anthocyanin 5-aromatic acyltransferase	-6.38
Mtr.11741.1.S1_at	Hydrolase, alpha/beta fold protein	-6.37
Msa.1632.1.S1_at	Hypothetical protein	-6.37
Mtr.38033.1.S1_at	bZIP transcription factor	-6.37
Mtr.33113.1.S1_at	Hypothetical protein	-6.37
Mtr.44288.1.S1_at	Hypothetical protein	-6.36
Mtr.40991.1.S1_at	Limonoid UDP-glucosyltransferase	-6.36
Mtr.44189.1.S1_at	BEL1-related homeotic protein 14	-6.36
Mtr.33645.1.S1_at	Lipoate-protein ligase	-6.35
Mtr.6081.1.S1_at	Family II lipase EXL2	-6.35
Mtr.42445.1.S1_at	Hypothetical protein	-6.35
Mtr.37531.1.S1_at	Hypothetical protein	-6.34
Msa.3011.1.S1_at	Hypothetical protein	-6.34
Mtr.44654.1.S1_at	Hypothetical protein	-6.33
Mtr.40437.1.S1_at	ABC transporter	-6.33
Mtr.46510.1.S1_x_at	Alpha/beta-amyrin synthase	-6.32
Mtr.37274.1.S1_at	Aquaporin 2	-6.32
Mtr.30437.1.S1_at	Hypothetical protein	-6.32
Mtr.19479.1.S1_at	Glutaredoxin	-6.31
Mtr.919.1.S1_s_at	Hypothetical protein	-6.31
Msa.1122.1.S1_at	Hypothetical protein	-6.31
Mtr.50754.1.S1_at	Hypothetical protein	-6.31
Mtr.7067.1.S1_at	Beta-galactosidase	-6.30
Mtr.21261.1.S1_s_at	Multicopper oxidase	-6.29
Mtr.11208.1.S1_at	Hypothetical protein	-6.29
Mtr.8777.1.S1_at	Hypothetical protein	-6.29
Mtr.12223.1.S1_at	Hypothetical protein	-6.28
Mtr.34832.1.S1_at	Hypothetical protein	-6.27
Mtr.7873.1.S1_at	Ribonuclease HII	-6.27

Mtr.1796.1.S1_at	Hypothetical protein	-6.27
Msa.1357.1.S1_at	Hypothetical protein	-6.26
Mtr.1405.1.S1_at	Hypothetical protein	-6.26
Mtr.32024.1.S1_at	Hypothetical protein	-6.26
Mtr.22041.1.S1_at	Hypothetical protein	-6.26
Mtr.45853.1.S1_at	Esterase/lipase/thioesterase	-6.25
Mtr.45338.1.S1_at	Cytochrome P450	-6.25
Mtr.6757.1.S1_at	Thaumatine-like protein	-6.25
Mtr.34732.1.S1_at	Trehalose-6-phosphate synthase	-6.24
Mtr.40886.1.S1_at	Hypothetical protein	-6.24
Mtr.35957.1.S1_at	Germin-like protein	-6.22
Mtr.7068.1.S1_at	Threonyl-tRNA synthetase	-6.22
Mtr.26499.1.S1_at	Lateral organ boundaries (LOB) domain protein	-6.22
Mtr.15385.1.S1_at	UbiA prenyltransferase	-6.20
Mtr.10423.1.S1_at	Hypothetical protein	-6.19
Mtr.37389.1.S1_at	Triose phosphate/phosphate translocator	-6.19
Mtr.1639.1.S1_at	Hypothetical protein	-6.19
Mtr.38919.1.S1_at	Hypothetical protein	-6.18
Mtr.20794.1.S1_at	Plant lipid transfer/seed storage/trypsin-alpha amylase inhibitor	-6.18
Msa.3151.1.S1_at	Hypothetical protein	-6.17
Mtr.20801.1.S1_at	Homeodomain-like protein	-6.17
Mtr.4048.1.S1_at	Hypothetical protein	-6.17
Msa.3097.1.S1_at	Hypothetical protein	-6.16
Mtr.44620.1.S1_at	Polygalacturonase	-6.16
Mtr.13967.1.S1_at	Phosphoenolpyruvate carboxylase	-6.15
Mtr.24075.1.S1_at	Mannitol dehydrogenase	-6.14
Msa.3084.1.S1_at	Hypothetical protein	-6.13
Mtr.25648.1.S1_at	Amino acid transporter	-6.12
Mtr.8972.1.S1_at	Hypothetical protein	-6.12
Mtr.11162.1.S1_at	GTP-binding protein LepA-like protein	-6.12
Mtr.8953.1.S1_s_at	bZIP transcription factor	-6.12
Mtr.51597.1.S1_at	Hypothetical protein	-6.12
Mtr.9831.1.S1_at	Mechanosensitive ion channel domain-containing protein	-6.12
Mtr.3101.1.S1_at	Hypothetical protein	-6.11
Mtr.16311.1.S1_at	Hypothetical protein	-6.11
Mtr.35600.1.S1_at	Pyrroline-5-carboxylate reductase	-6.11
Mtr.41133.1.S1_at	Bell-like homeodomain protein 3	-6.11
Mtr.1429.1.S1_s_at	Hypothetical protein	-6.10
Mtr.43001.1.S1_at	Aminotransferase 2,	-6.10
Mtr.13575.1.S1_at	Hypothetical protein	-6.09
Mtr.34896.1.S1_at	Receptor-like protein kinase	-6.09
Mtr.11988.1.S1_at	Tryptophanyl-tRNA synthetase	-6.08
Msa.3002.1.S1_at	Hypothetical protein	-6.08
Mtr.11265.1.S1_at	Glycosyl hydrolase	-6.08
Mtr.43636.1.S1_at	Fructose-6-phosphate 2-kinase/fructose-2,6-bisphosphatase	-6.08
Mtr.11421.1.S1_at	Ripening-related protein	-6.07
Mtr.42133.1.S1_at	Protein kinase	-6.06
Mtr.41850.1.S1_at	UOS1	-6.06
Msa.3188.1.S1_at	Hypothetical protein	-6.06
Mtr.10615.1.S1_at	Cellulose synthase catalytic subunit	-6.06
Mtr.37547.1.S1_at	Hypothetical protein	-6.05
Mtr.13545.1.S1_at	Hypothetical protein	-6.05
Mtr.13979.1.S1_at	Lipid transfer protein	-6.04
Mtr.41668.1.S1_at	Ribulose-bisphosphate carboxylase activase large isoform protein	-6.04

Mtr.41251.1.S1_at	Hypothetical protein	-6.03
Msa.1523.1.S1_at	Hypothetical protein	-6.03
Msa.2534.1.S1_at	Hypothetical protein	-6.02
Mtr.46448.1.S1_s_at	Hypothetical protein	-6.02
Msa.1434.1.S1_at	Hypothetical protein	-6.02
Mtr.30692.1.S1_at	Receptor-protein kinase	-6.01
Mtr.11739.1.S1_s_at	Homogentisate phytylprenyltransferase	-5.99
Mtr.39943.1.S1_at	Hypothetical protein	-5.99
Mtr.40676.1.S1_at	CND41, chloroplast nucleoid DNA binding protein	-5.99
Mtr.51925.1.S1_at	Hypothetical protein	-5.98
Mtr.44020.1.S1_at	Brn1-like protein	-5.98
Mtr.10648.1.S1_at	UOS1	-5.97
Mtr.40473.1.S1_at	Lipid transfer protein	-5.97
Mtr.5586.1.S1_s_at	Beta-carotene hydroxylase	-5.97
Msa.3148.1.S1_at	Hypothetical protein	-5.96
Mtr.39095.1.S1_at	Alcohol oxidase	-5.96
Mtr.23402.1.S1_at	Aldehyde dehydrogenase	-5.95
Mtr.10231.1.S1_at	Hypothetical protein	-5.95
Mtr.4994.1.S1_at	Hypothetical protein	-5.95
Mtr.28667.1.S1_at	Hypothetical protein	-5.95
Mtr.12410.1.S1_at	Fructose-1,6-bisphosphatase	-5.95
Mtr.12349.1.S1_at	Hypothetical protein	-5.95
Mtr.13232.1.S1_at	Hypothetical protein	-5.95
Mtr.41624.1.S1_at	SEC14 cytosolic factor	-5.94
Mtr.45139.1.S1_at	Sulfate transporter 4.1	-5.94
Mtr.52045.1.S1_at	Hypothetical protein	-5.94
Mtr.50562.1.S1_s_at	Zn-finger, Dof type	-5.94
Mtr.5741.1.S1_s_at	Lipoate-protein ligase	-5.93
Mtr.35894.1.S1_at	Hypothetical protein	-5.93
Mtr.44852.1.S1_at	Glu-tRNA(Gln) amidotransferase subunit B	-5.93
Mtr.50952.1.S1_at	Ribosomal protein L9	-5.92
Mtr.39387.1.S1_s_at	DCL protein	-5.91
Mtr.44282.1.S1_at	THA4 protein	-5.91
Mtr.34494.1.S1_at	Hypothetical protein	-5.91
Mtr.52185.1.S1_at	Hypothetical protein	-5.90
Mtr.26161.1.S1_at	Peroxisomal membrane protein	-5.90
Mtr.43371.1.S1_at	Peroxidase 55	-5.90
Mtr.875.1.S1_s_at	Thioredoxin x	-5.89
Msa.1112.1.S1_at	Hypothetical protein	-5.89
Mtr.13533.1.S1_at	Hypothetical protein	-5.88
Mtr.17374.1.S1_at	C4-dicarboxylate transporter/malic acid transport protein	-5.88
Mtr.33398.1.S1_s_at	Hypothetical protein	-5.88
Mtr.9952.1.S1_at	Hypothetical protein	-5.88
Mtr.12037.1.S1_at	Hypothetical protein	-5.88
Mtr.7911.1.S1_s_at	ER1-like receptor kinase	-5.87
Mtr.46060.1.S1_at	Hypothetical protein	-5.87
Mtr.51204.1.S1_at	Amine oxidase	-5.86
Mtr.5242.1.S1_at	Cellulose synthase	-5.86
Mtr.8497.1.S1_at	PSI light-harvesting antenna chlorophyll a/b-binding protein	-5.86
Msa.1689.1.S1_at	Hypothetical protein	-5.85
Mtr.36469.1.S1_at	Hypothetical protein	-5.85
Mtr.9315.1.S1_at	Hypothetical protein	-5.84
Mtr.32674.1.S1_at	Alr1013 protein	-5.83
Mtr.11359.1.S1_at	Hypothetical protein	-5.83

Mtr.44847.1.S1_s_at	Glycosyltransferase family protein	-5.83
Mtr.41877.1.S1_at	Hypothetical protein	-5.83
Mtr.32411.1.S1_a_at	Hypothetical protein	-5.82
Mtr.8844.1.S1_at	Mitogen-activated protein kinase	-5.82
Mtr.38019.1.S1_at	Lil3 protein	-5.81
Mtr.37759.1.S1_at	Hypothetical protein	-5.81
Mtr.33522.1.S1_at	Hypothetical protein	-5.81
Mtr.46046.1.S1_at	Lipolytic enzyme	-5.80
Mtr.36804.1.S1_at	Hypothetical protein	-5.79
Mtr.38983.1.S1_at	Hypothetical protein	-5.79
Mtr.31199.1.S1_s_at	Cytochrome P450	-5.79
Mtr.39220.1.S1_at	Methyltransferase	-5.78
Mtr.27862.1.S1_s_at	Hypothetical protein	-5.77
Mtr.35361.1.S1_at	Fructose-6-phosphate 2-kinase/fructose-2,6-bisphosphatase	-5.77
Mtr.37372.1.S1_at	Dehydroascorbate reductase	-5.76
Mtr.9532.1.S1_at	Hypothetical protein	-5.76
Mtr.10480.1.S1_at	Glutamine synthetase	-5.75
Mtr.38436.1.S1_at	Cyclin-like protein	-5.75
Mtr.8509.1.S1_at	Hypothetical protein	-5.74
Msa.1462.1.S1_at	Hypothetical protein	-5.74
Mtr.5120.1.S1_at	Hypothetical protein	-5.74
Mtr.34697.1.S1_at	THA4 protein	-5.74
Mtr.13951.1.S1_at	Hypothetical protein	-5.73
Mtr.19984.1.S1_at	Hypothetical protein	-5.73
Mtr.38441.1.S1_at	Hypothetical protein	-5.73
Mtr.17358.1.S1_at	Protein synthesis factor	-5.73
Mtr.44687.1.S1_at	Hypothetical protein	-5.73
Msa.3149.1.S1_at	Hypothetical protein	-5.72
Mtr.38693.1.S1_at	Hypothetical protein	-5.72
Mtr.51752.1.S1_at	Short-chain dehydrogenase/reductase	-5.72
Mtr.20636.1.S1_at	NAD-dependent epimerase/dehydratase	-5.71
Mtr.39164.1.S1_at	Hypothetical protein	-5.71
Mtr.12363.1.S1_at	Hypothetical protein	-5.71
Msa.1866.1.S1_at	Hypothetical protein	-5.71
Mtr.17798.1.S1_at	GNS1/SUR4 membrane protein	-5.71
Mtr.12306.1.S1_at	Hypothetical protein	-5.71
Mtr.40844.1.S1_at	Diphosphonucleotide phosphatase 1	-5.70
Mtr.42974.1.S1_at	Hydroxyisourate hydrolase	-5.70
Mtr.13202.1.S1_at	Cellulose synthase CesA-1	-5.69
Mtr.44411.1.S1_at	Peptide methionine sulfoxide reductase	-5.69
Mtr.28737.1.S1_at	Homeodomain protein	-5.68
Mtr.45813.1.S1_at	Hypothetical protein	-5.68
Msa.2963.1.S1_s_at	Hypothetical protein	-5.68
Mtr.8730.1.S1_at	Hypothetical protein	-5.68
Mtr.45567.1.S1_at	Fatty aldehyde dehydrogenase 1	-5.67
Mtr.40897.1.S1_at	Hypothetical protein	-5.67
Mtr.48097.1.S1_at	Hypothetical protein	-5.67
Mtr.13053.1.S1_at	Urophorphyrin III methylase	-5.65
Msa.1083.1.S1_at	Hypothetical protein	-5.64
Mtr.8478.1.S1_at	Cytochrome P450	-5.63
Mtr.38047.1.S1_at	Monogalactosyldiacylglycerol synthase	-5.63
Mtr.29983.1.S1_s_at	Beta-carotene hydroxylase	-5.62
Mtr.11489.1.S1_at	RedA protein	-5.62
Mtr.44607.1.S1_at	Pyrroline-5-carboxylate synthetase	-5.62

Mtr.51862.1.S1_at	Hypothetical protein	-5.61
Mtr.31764.1.S1_at	Alpha-mannosidase	-5.60
Mtr.31300.1.S1_at	Disease resistance response protein	-5.60
Mtr.12447.1.S1_at	Proline transport protein	-5.60
Mtr.4424.1.S1_at	Hypothetical protein	-5.60
Mtr.11363.1.S1_at	Glycosyltransferase	-5.60
Msa.1866.1.S1_x_at	Hypothetical protein	-5.60
Mtr.6980.1.S1_at	Multidrug resistance-associated protein 2	-5.60
Mtr.41896.1.S1_s_at	Hypothetical protein	-5.59
Mtr.37982.1.S1_at	Twist related protein 1	-5.59
Mtr.41829.1.S1_at	Hypothetical protein	-5.59
Mtr.33664.1.S1_at	Ferredoxin-dependent glutamate synthase	-5.58
Mtr.20947.1.S1_x_at	Serine/threonine protein kinase	-5.58
Mtr.18153.1.S1_at	Hypothetical protein	-5.58
Mtr.20560.1.S1_at	Purple acid phosphatase	-5.57
Mtr.40508.1.S1_at	Magnesium-chelatase subunit chlI	-5.57
Mtr.32495.1.S1_at	Protein kinase	-5.57
Mtr.15775.1.S1_at	Glycoside hydrolase	-5.56
Msa.2997.1.S1_at	Hypothetical protein	-5.56
Mtr.27754.1.S1_at	Hypothetical protein	-5.56
Mtr.32386.1.S1_at	Strubbelig receptor	-5.56
Mtr.9529.1.S1_at	Hypothetical protein	-5.56
Mtr.13614.1.S1_at	Pyrimidine 5'-nucleotidase	-5.55
Mtr.14230.1.S1_at	Hypothetical protein	-5.54
Mtr.5847.1.S1_at	Zinc finger protein	-5.53
Mtr.15418.1.S1_at	Histone deacetylase	-5.53
Mtr.8416.1.S1_s_at	Cytochrome b	-5.53
Mtr.5115.1.S1_s_at	Hypothetical protein	-5.53
Mtr.40775.1.S1_at	Tyrosine phosphatase	-5.52
Mtr.38697.1.S1_at	Plant viral-response family protein	-5.52
Mtr.10814.1.S1_at	Epoxide hydrolase	-5.52
Mtr.9586.1.S1_at	Hypothetical protein	-5.52
Mtr.44730.1.S1_at	Nitrate transporter	-5.52
Mtr.42840.1.S1_at	Germin-like protein	-5.52
Mtr.17357.1.S1_at	Hypothetical protein	-5.51
Mtr.11222.1.S1_at	Hypothetical protein	-5.51
Mtr.33475.1.S1_s_at	Proline-rich protein	-5.49
Mtr.33440.1.S1_at	30S ribosomal protein S16	-5.49
Mtr.28675.1.S1_s_at	Polygalacturonase	-5.49
Mtr.38254.1.S1_at	Ubiquitin-conjugating enzyme	-5.49
Mtr.37882.1.S1_at	Nucleotide pyrophosphatase/phosphodiesterase	-5.48
Mtr.40230.1.S1_at	Photosystem I reaction center subunit V	-5.48
Mtr.43622.1.S1_s_at	NADH dehydrogenase	-5.48
Mtr.1621.1.S1_s_at	Hypothetical protein	-5.47
Mtr.34189.1.S1_at	Hypothetical protein	-5.47
Mtr.9853.1.S1_at	Hypothetical protein	-5.47
Mtr.25389.1.S1_at	Vestitone reductase	-5.46
Mtr.43459.1.S1_at	Hypothetical protein	-5.46
Mtr.44783.1.S1_at	Hypothetical protein	-5.46
Mtr.5795.1.S1_at	Hypothetical protein	-5.46
Mtr.2343.1.S1_at	Hypothetical protein	-5.46
Mtr.43923.1.S1_at	Calcium-transporting ATPase 9	-5.45
Mtr.25344.1.S1_at	Hypothetical protein	-5.45
Mtr.15345.1.S1_at	Multi antimicrobial extrusion protein	-5.45

Mtr.43771.1.S1_at	Hypothetical protein	-5.44
Mtr.38263.1.S1_at	Hypothetical protein	-5.44
Mtr.41079.1.S1_at	NADH dehydrogenase	-5.44
Mtr.11929.1.S1_at	Beta-1,4-xylosidase	-5.43
Msa.1921.1.S1_at	Hypothetical protein	-5.43
Mtr.40744.1.S1_at	Hypothetical protein	-5.43
Mtr.13813.1.S1_s_at	Alkaline/neutral invertase	-5.42
Mtr.37436.1.S1_at	Hypersensitive-induced response protein	-5.42
Mtr.31160.1.S1_at	En/Spm-like transposon protein	-5.42
Mtr.8754.1.S1_at	Rhodanese-like family protein	-5.41
Mtr.38447.1.S1_at	Cell Wall Invertase Inhibitor	-5.41
Mtr.929.1.S1_at	Hypothetical protein	-5.41
Mtr.27397.1.S1_at	Hypothetical protein	-5.40
Mtr.10605.1.S1_at	Hypothetical protein	-5.40
Mtr.12922.1.S1_at	Hypothetical protein	-5.40
Mtr.40249.1.S1_at	ATP synthase delta chain	-5.40
Mtr.38054.1.S1_at	Hypothetical protein	-5.39
Mtr.13612.1.S1_at	Hypothetical protein	-5.39
Msa.1928.1.S1_at	Hypothetical protein	-5.39
Mtr.1617.1.S1_s_at	Basic blue copper protein	-5.38
Mtr.43681.1.S1_at	CYP1	-5.37
Mtr.4126.1.S1_at	Laccase	-5.36
Mtr.41835.1.S1_at	Hypothetical protein	-5.36
Mtr.31727.1.S1_at	Hypothetical protein	-5.35
Mtr.12886.1.S1_at	Hypothetical protein	-5.34
Mtr.13388.1.S1_s_at	Hypothetical protein	-5.34
Mtr.38481.1.S1_at	Hypothetical protein	-5.34
Msa.1574.1.S1_at	Hypothetical protein	-5.33
Mtr.27146.1.S1_s_at	Cyclin-like protein	-5.33
Msa.2909.1.S1_at	Hypothetical protein	-5.33
Mtr.19280.1.S1_at	Multi antimicrobial extrusion protein	-5.33
Mtr.9891.1.S1_at	Hypothetical protein	-5.33
Mtr.25786.1.S1_at	Hypothetical protein	-5.32
Msa.1209.1.S1_at	Hypothetical protein	-5.32
Mtr.32796.1.S1_at	Sodium transport protein	-5.32
Mtr.37806.1.S1_at	Cellulose synthase	-5.32
Mtr.7062.1.S1_at	Hypothetical protein	-5.30
Mtr.8981.1.S1_at	Hypothetical protein	-5.30
Mtr.8575.1.S1_x_at	Glucosyltransferase-13	-5.29
Mtr.44825.1.S1_at	POZ domain protein	-5.29
Mtr.32634.1.S1_s_at	Heat shock protein	-5.28
Mtr.13969.1.S1_at	Glucosyltransferase	-5.28
Mtr.11645.1.S1_at	Methionine aminopeptidase	-5.27
Mtr.1431.1.S1_at	Hypothetical protein	-5.27
Mtr.39502.1.S1_at	Hypothetical protein	-5.27
Mtr.22606.1.S1_at	Pentatricopeptide (PPR) repeat-containing protein	-5.27
Mtr.14192.1.S1_at	Inositol monophosphatase	-5.27
Msa.2613.1.S1_at	Hypothetical protein	-5.27
Mtr.31331.1.S1_s_at	Hypothetical protein	-5.26
Mtr.35779.1.S1_at	Seed maturation protein	-5.26
Mtr.32040.1.S1_at	Hypothetical protein	-5.26
Mtr.16356.1.S1_s_at	Phospholipid/glycerol acyltransferase	-5.26
Mtr.38390.1.S1_at	Hypothetical protein	-5.25
Mtr.18797.1.S1_at	Proteinase inhibitor I3	-5.25

Mtr.29383.1.S1_at	Hypothetical protein	-5.24
Mtr.11798.1.S1_at	Cellulose synthase	-5.24
Msa.1890.1.S1_at	Hypothetical protein	-5.23
Mtr.18703.1.S1_at	Serine/threonine protein kinase	-5.22
Mtr.40561.1.S1_at	Hypothetical protein	-5.22
Mtr.38245.1.S1_at	Transcription factor RAU1	-5.22
Mtr.12143.1.S1_at	Hypothetical protein	-5.22
Mtr.37688.1.S1_at	NtPRp27-like protein	-5.21
Mtr.30695.1.S1_at	Hypothetical protein	-5.21
Mtr.33840.1.S1_at	Hypothetical protein	-5.21
Mtr.44855.1.S1_at	Urophorphyrin III methylase	-5.21
Mtr.7101.1.S1_at	C2H2 zinc-finger protein	-5.21
Mtr.50278.1.S1_at	Small GTP-binding protein	-5.20
Mtr.38455.1.S1_at	Hypothetical protein	-5.20
Mtr.35540.1.S1_at	Hypothetical protein	-5.19
Mtr.10800.1.S1_s_at	Hypothetical protein	-5.19
Mtr.44860.1.S1_at	MADS5 protein	-5.19
Msa.2839.1.S1_at	Hypothetical protein	-5.18
Mtr.35557.1.S1_s_at	Hypothetical protein	-5.17
Mtr.28757.1.S1_at	Hypothetical protein	-5.17
Mtr.4792.1.S1_at	Leaf ubiquitous urease	-5.16
Mtr.15024.1.S1_at	Hypothetical protein	-5.16
Mtr.14157.1.S1_at	Gibberellin regulated protein	-5.16
Mtr.8420.1.S1_at	Hypothetical protein	-5.16
Mtr.37448.1.S1_at	Photosystem II	-5.16
Mtr.41539.1.S1_at	Hypothetical protein	-5.16
Mtr.27915.1.S1_at	Hypothetical protein	-5.15
Mtr.31928.1.S1_at	Peroxidase 25	-5.15
Msa.2814.1.S1_at	Hypothetical protein	-5.14
Mtr.34376.1.S1_at	Hypothetical protein	-5.14
Mtr.9427.1.S1_at	Hypothetical protein	-5.14
Mtr.41215.1.S1_at	Germin-like protein	-5.14
Mtr.49776.1.S1_x_at	Auxin responsive SAUR	-5.14
Mtr.40565.1.S1_at	GTP-binding protein LepA	-5.14
Mtr.32399.1.S1_at	Hypothetical protein	-5.13
Mtr.18486.1.S1_at	Chlorophyll A-B binding protein	-5.13
Mtr.32044.1.S1_at	Hypothetical protein	-5.12
Mtr.33655.1.S1_at	CYP71A10	-5.12
Mtr.50907.1.S1_at	Glycoside hydrolase	-5.11
Mtr.27855.1.S1_at	Heat shock protein	-5.11
Mtr.36941.1.S1_at	Hypothetical protein	-5.10
Msa.1658.1.S1_at	Hypothetical protein	-5.10
Mtr.24601.1.S1_at	Receptor-related protein kinase	-5.10
Msa.1380.1.S1_at	Hypothetical protein	-5.09
Mtr.40444.1.S1_at	Hypothetical protein	-5.09
Mtr.12904.1.S1_at	Pectinacetylesterase	-5.08
Mtr.15246.1.S1_at	ABC transporter	-5.08
Mtr.12989.1.S1_at	Fructosamine-3-kinase	-5.08
Mtr.37361.1.S1_at	Chlorophyll a-b binding protein CP29.3	-5.08
Mtr.9810.1.S1_at	Hypothetical protein	-5.08
Mtr.36338.1.S1_at	Hypothetical protein	-5.07
Mtr.5489.1.S1_at	Hypothetical protein	-5.07
Mtr.44737.1.S1_at	Hypothetical protein	-5.07
Mtr.6148.1.S1_x_at	Serine carboxypeptidase II-2	-5.06

Mtr.49448.1.S1_at	Glycoside hydrolase	-5.06
Mtr.37642.1.S1_at	NADP-dependent oxidoreductase P2	-5.06
Mtr.15949.1.S1_at	Hypothetical protein	-5.06
Mtr.6532.1.S1_s_at	3-ketoacyl-ACP synthase	-5.06
Mtr.28938.1.S1_at	Hypothetical protein	-5.06
Mtr.25447.1.S1_at	Hypothetical protein	-5.05
Mtr.12572.1.S1_at	Hypothetical protein	-5.05
Mtr.10567.1.S1_s_at	Hypothetical protein	-5.04
Mtr.8993.1.S1_at	Hypothetical protein	-5.04
Mtr.37192.1.S1_at	Hypothetical protein	-5.04
Mtr.23551.1.S1_at	Late embryogenesis abundant protein D-34	-5.04
Mtr.17195.1.S1_at	RNA-binding region RNP-1	-5.04
Mtr.50015.1.S1_at	Hypothetical protein	-5.04
Mtr.41900.1.S1_at	Hypothetical protein	-5.03
Mtr.51598.1.S1_at	NUDIX hydrolase	-5.03
Mtr.34765.1.S1_at	Hypothetical protein	-5.03
Mtr.9540.1.S1_at	RING finger	-5.03
Mtr.40908.1.S1_at	Ribosomal protein L6	-5.02
Mtr.32540.1.S1_at	Hypothetical protein	-5.02
Mtr.37681.1.S1_s_at	Hypothetical protein	-5.02
Mtr.32821.1.S1_at	Hypothetical protein	-5.02
Msa.3056.1.S1_at	Hypothetical protein	-5.01
Mtr.45264.1.S1_at	Hypothetical protein	-5.01
Mtr.33045.1.S1_at	Hypothetical protein	-5.01
Mtr.37517.1.S1_at	Basic blue protein	-4.99
Mtr.40184.1.S1_at	FtsH protease (VAR2)	-4.99
Mtr.38468.1.S1_at	Proline-rich protein	-4.99
Mtr.17550.1.S1_at	Plant lipid transfer protein	-4.99
Mtr.44455.1.S1_at	Hypothetical protein	-4.98
Mtr.44897.1.S1_at	Hypothetical protein	-4.98
Mtr.5646.1.S1_at	Glutathione S-transferase	-4.97
Mtr.47127.1.S1_at	Far-red impaired response protein	-4.97
Mtr.16200.1.S1_at	Esterase/lipase/thioesterase	-4.97
Mtr.45183.1.S1_at	Hypothetical protein	-4.95
Msa.2747.1.S1_at	Hypothetical protein	-4.94
Mtr.23616.1.S1_at	DNA-binding protein	-4.94
Mtr.42933.1.S1_s_at	Nonspecific lipid-transfer protein precursor (LTP)	-4.94
Mtr.12880.1.S1_at	Serine/threonine protein kinase	-4.93
Mtr.34711.1.S1_at	C-type cytochrome biogenesis protein Ccs1	-4.93
Mtr.13309.1.S1_at	Hypothetical protein	-4.93
Mtr.38453.1.S1_at	Proline-rich protein	-4.93
Mtr.38021.1.S1_at	Hypothetical protein	-4.92
Mtr.24898.1.S1_at	Magnesium/proton exchanger	-4.92
Mtr.32928.1.S1_s_at	Hypothetical protein	-4.92
Mtr.6093.1.S1_s_at	Subtilisin-like protease	-4.92
Mtr.46514.1.S1_x_at	Alpha/beta-amyrin synthase	-4.92
Mtr.40332.1.S1_at	5,10-methylenetetrahydrofolate dehydrogenase	-4.91
Mtr.18220.1.S1_s_at	Hypothetical protein	-4.91
Mtr.44805.1.S1_at	Hypothetical protein	-4.91
Mtr.17452.1.S1_at	Glycoside transferase	-4.91
Mtr.14245.1.S1_at	Hypothetical protein	-4.91
Mtr.18022.1.S1_at	Hypothetical protein	-4.90
Mtr.45276.1.S1_at	LIM domain containing protein	-4.90
Mtr.34829.1.S1_at	Hypothetical protein	-4.90

Mtr.21855.1.S1_at	Hypothetical protein	-4.90
Mtr.42899.1.S1_a_at	Carboxylesterase	-4.89
Mtr.39210.1.S1_at	Hypothetical protein	-4.89
Mtr.24083.1.S1_at	Uroporphyrinogen decarboxylase	-4.89
Mtr.33770.1.S1_at	Hypothetical protein	-4.88
Mtr.12907.1.S1_at	Hypothetical protein	-4.88
Mtr.51302.1.S1_at	Ras small GTPase	-4.88
Mtr.38595.1.S1_at	Hypothetical protein	-4.88
Mtr.2090.1.S1_at	Cation transport ATPase	-4.88
Mtr.34734.1.S1_at	Potassium transporter 2	-4.87
Mtr.50716.1.S1_s_at	Glycosyl transferase	-4.87
Mtr.50857.1.S1_at	Peptidase S10	-4.87
Mtr.43323.1.S1_at	Polygalacturonase-like protein	-4.87
Mtr.38114.1.S1_at	Thioredoxin M-type 4	-4.87
Mtr.12848.1.S1_at	Berberine bridge enzyme-like protein	-4.87
Mtr.32711.1.S1_at	Hypothetical protein	-4.87
Mtr.994.1.S1_at	Hypothetical protein	-4.86
Mtr.21060.1.S1_at	Peptidyl-prolyl cis-trans isomerase, cyclophilin type	-4.86
Mtr.43551.1.S1_at	Allyl alcohol dehydrogenase	-4.85
Mtr.33116.1.S1_at	Receptor protein kinase	-4.85
Mtr.7095.1.S1_at	Na+/H+ antiporter NHX6	-4.85
Mtr.26278.1.S1_s_at	Pyrroline-5-carboxylate reductase	-4.85
Mtr.28730.1.S1_at	En/Spm-like transposon protein	-4.84
Mtr.42457.1.S1_at	Hypothetical protein	-4.84
Mtr.28324.1.S1_at	Hypothetical protein	-4.84
Mtr.42933.1.S1_x_at	Nonspecific lipid-transfer protein precursor (LTP)	-4.84
Mtr.27107.1.S1_at	MYC1	-4.84
Mtr.8609.1.S1_at	Photosystem II reaction center	-4.83
Mtr.34447.1.S1_at	Hypothetical protein	-4.83
Mtr.37387.1.S1_s_at	Inhibitor of apoptosis-like protein	-4.83
Mtr.33127.1.S1_at	Hypothetical protein	-4.83
Mtr.45322.1.S1_at	Hypothetical protein	-4.82
Mtr.31359.1.S1_at	Hypothetical protein	-4.82
Mtr.11554.1.S1_at	Alpha-amylase	-4.82
Mtr.20268.1.S1_at	Hypothetical protein	-4.82
Mtr.44100.1.S1_at	Hypothetical protein	-4.82
Mtr.31115.1.S1_at	Hypothetical protein	-4.82
Msa.2646.1.S1_at	Hypothetical protein	-4.81
Mtr.24815.1.S1_at	Glutaredoxin protein	-4.81
Mtr.8504.1.S1_at	Hypothetical protein	-4.81
Mtr.41127.1.S1_at	Pherophorin	-4.81
Mtr.24829.1.S1_at	Hypothetical protein	-4.80
Mtr.9072.1.S1_at	Acyltransferase	-4.80
Mtr.14419.1.S1_s_at	Purple acid phosphatase	-4.80
Mtr.27207.1.S1_s_at	Hypothetical protein	-4.80
Mtr.12724.1.S1_at	PDI-like protein	-4.80
Mtr.41992.1.S1_at	Endo-beta-N-acetylglucosaminidase	-4.80
Mtr.5274.1.S1_s_at	Cellulose synthase	-4.79
Mtr.52072.1.S1_at	Hypothetical protein	-4.79
Mtr.44552.1.S1_at	Hypothetical protein	-4.79
Mtr.8435.1.S1_at	Lipoxygenase	-4.79
Mtr.27854.1.S1_s_at	NADP-dependent oxidoreductase P1	-4.79
Mtr.44578.1.S1_at	RAB1X	-4.78
Mtr.44600.1.S1_at	Hypothetical protein	-4.78

Mtr.10293.1.S1_at	Alanyl-tRNA synthetase	-4.77
Mtr.43506.1.S1_at	Hypothetical protein	-4.77
Mtr.44395.1.S1_at	Hypothetical protein	-4.77
Mtr.6724.1.S1_at	Hypothetical protein	-4.77
Mtr.13288.1.S1_at	ADP-glucose pyrophosphorylase large subunit	-4.76
Mtr.5792.1.S1_at	Hypothetical protein	-4.76
Mtr.37342.1.S1_at	Mannitol dehydrogenase	-4.76
Mtr.34540.1.S1_s_at	Hypothetical protein	-4.76
Mtr.39373.1.S1_at	Alanyl-tRNA synthetase	-4.75
Mtr.42357.1.S1_at	Hypothetical protein	-4.75
Mtr.12774.1.S1_s_at	Bg55 protein	-4.75
Mtr.28309.1.S1_at	Acyl-CoA thioesterase	-4.75
Mtr.50826.1.S1_at	Beta-ketoacyl synthase	-4.74
Mtr.9717.1.S1_at	WAK-like kinase	-4.73
Mtr.29094.1.S1_s_at	Hypothetical protein	-4.73
Mtr.5882.1.S1_at	Hypothetical protein	-4.73
Msa.1391.1.S1_at	Hypothetical protein	-4.73
Mtr.34614.1.S1_at	Hypothetical protein	-4.73
Mtr.16895.1.S1_at	Hypothetical protein	-4.72
Mtr.10347.1.S1_at	Receptor-like protein kinase	-4.72
Mtr.33436.1.S1_at	Hypothetical protein	-4.72
Mtr.13198.1.S1_at	PHAP2B protein	-4.72
Mtr.43806.1.S1_at	Hypothetical protein	-4.72
Mtr.34714.1.S1_at	Cyclin-dependent kinase 3	-4.71
Mtr.19940.1.S1_at	Hypothetical protein	-4.71
Mtr.10198.1.S1_at	Phosphoenolpyruvate carboxylase	-4.71
Mtr.50974.1.S1_at	Hypothetical protein	-4.71
Mtr.4037.1.S1_at	Hypothetical protein	-4.70
Mtr.40774.1.S1_s_at	Tyrosine phosphatase	-4.70
Mtr.8790.1.S1_at	Peripheral-type benzodiazepine receptor	-4.69
Mtr.40121.1.S1_at	Peroxidase1C	-4.69
Mtr.40723.1.S1_at	Hypothetical protein	-4.69
Mtr.35108.1.S1_at	NAP	-4.69
Mtr.40534.1.S1_at	Sulfate transporter protein	-4.69
Mtr.12418.1.S1_at	Hypothetical protein	-4.68
Mtr.44321.1.S1_at	Hypothetical protein	-4.68
Mtr.29279.1.S1_at	Lipoxygenase	-4.68
Mtr.8859.1.S1_at	Proline rich protein	-4.68
Mtr.11440.1.S1_at	Thylakoid lumenal 21.5 kDa protein	-4.67
Msa.1818.1.S1_at	Hypothetical protein	-4.67
Msa.1114.1.S1_at	Hypothetical protein	-4.67
Mtr.34452.1.S1_at	Hypothetical protein	-4.67
Mtr.9495.1.S1_at	Sugar transporter-like protein	-4.67
Mtr.35849.1.S1_at	Hypothetical protein	-4.67
Mtr.34640.1.S1_at	Hypothetical protein	-4.66
Mtr.1715.1.S1_at	Aminoalcoholphosphotransferase	-4.66
Mtr.26672.1.S1_s_at	Hypothetical protein	-4.65
Mtr.41274.1.S1_at	Hypothetical protein	-4.64
Mtr.6731.1.S1_at	PSII D1 protein processing enzyme	-4.64
Mtr.38321.1.S1_at	Transfactor-like protein	-4.64
AFFX-Mtr-gsta-5_at	2,4-D inducible glutathione S-transferase	-4.64
Mtr.5745.1.S1_at	Receptor-like protein kinase	-4.64
Mtr.16200.1.S1_x_at	Esterase/lipase/thioesterase	-4.64
Mtr.44721.1.S1_at	Rust resistance kinase	-4.63

Mtr.10882.1.S1_at	Hypothetical protein	-4.62
Mtr.42374.1.S1_at	Hypothetical protein	-4.62
Mtr.32709.1.S1_at	Hypothetical protein	-4.62
Mtr.37602.1.S1_at	Proline-rich protein	-4.62
Mtr.37299.1.S1_at	Hypothetical protein	-4.61
Mtr.6322.1.S1_s_at	Cytochrome P450	-4.61
Mtr.13786.1.S1_at	Hypothetical protein	-4.61
Mtr.22750.1.S1_at	Hypothetical protein	-4.61
Mtr.19517.1.S1_at	Ribulose bisphosphate carboxylase	-4.61
Mtr.39315.1.S1_s_at	Hypothetical protein	-4.60
Mtr.51909.1.S1_at	Phenylalanine/histidine ammonia-lyase	-4.59
Mtr.13472.1.S1_at	Interleukin-1 receptor-associated kinase-3	-4.59
Mtr.38647.1.S1_at	WAK-like kinase	-4.59
Mtr.22791.1.S1_at	UDP-glucose pyrophosphorylase	-4.59
Mtr.35618.1.S1_at	Multi resistance protein	-4.58
Mtr.12931.1.S1_at	Hypothetical protein	-4.58
Mtr.22414.1.S1_s_at	Hypothetical protein	-4.58
Mtr.4818.1.S1_s_at	Gluthamine synthetase	-4.58
Mtr.43042.1.S1_at	LEDI-5c protein	-4.57
Mtr.15494.1.S1_at	Cinnamyl alcohol dehydrogenase	-4.57
Mtr.41489.1.S1_at	Hypothetical protein	-4.57
Mtr.34709.1.S1_at	Ser-thr protein kinase	-4.57
Mtr.42095.1.S1_at	Receptor like protein	-4.57
Mtr.8212.1.S1_at	Protein kinase	-4.57
Msa.1877.1.S1_at	Hypothetical protein	-4.57
Mtr.13167.1.S1_at	Acyl CoA synthetase	-4.57
Mtr.37311.1.S1_at	Chlorophyll a/b-binding protein type I	-4.57
Mtr.31555.1.S1_at	Beta-1,3-glucanase	-4.57
Mtr.5962.1.S1_s_at	Nonspecific lipid-transfer protein	-4.56
Mtr.31155.1.S1_at	Hypothetical protein	-4.56
Msa.1022.1.S1_at	Hypothetical protein	-4.56
Mtr.24912.1.S1_at	Hypothetical protein	-4.56
Mtr.27682.1.S1_at	NS5 protein	-4.55
Mtr.713.1.S1_s_at	Nucleotide pyrophosphatase	-4.55
Mtr.40668.1.S1_at	Hypothetical protein	-4.55
Mtr.35857.1.S1_at	Hypothetical protein	-4.55
Mtr.39989.1.S1_at	Proteinase IV	-4.55
Mtr.6658.1.S1_at	Auxin-induced protein	-4.55
Mtr.39482.1.S1_at	Hypothetical protein	-4.54
Mtr.13421.1.S1_at	Cytochrome b-561	-4.54
Mtr.50308.1.S1_at	Hypothetical protein	-4.54
Mtr.50307.1.S1_at	Haloacid dehalogenase-like hydrolase	-4.54
Mtr.31565.1.S1_s_at	Hypothetical protein	-4.53
Mtr.41288.1.S1_at	Hypothetical protein	-4.53
Mtr.25043.1.S1_at	Hypothetical protein	-4.52
Mtr.8496.1.S1_at	Hypothetical protein	-4.52
Mtr.45389.1.S1_at	Hypothetical protein	-4.51
Mtr.42104.1.S1_at	Heterotrimeric G-protein gamma subunit 2	-4.51
Mtr.40027.1.S1_at	CBL-interacting protein kinase	-4.51
Mtr.8858.1.S1_at	Biogenesis protein	-4.51
Mtr.35867.1.S1_at	Hypothetical protein	-4.51
Mtr.37563.1.S1_at	Outer envelope membrane protein	-4.51
Mtr.44590.1.S1_s_at	Glycuronosyltransferase	-4.50
Mtr.29095.1.S1_at	Respiratory burst oxidase homolog	-4.50

Mtr.28777.1.S1_at	Hypothetical protein	-4.50
Msa.579.1.S1_at	Hypothetical protein	-4.50
Mtr.8501.1.S1_s_at	Glycolate oxidase	-4.50
Mtr.33506.1.S1_at	Hypothetical protein	-4.50
Mtr.8925.1.S1_s_at	Signal recognition particle 54 kDa subunit	-4.50
Mtr.11999.1.S1_at	Hypothetical protein	-4.49
Mtr.25990.1.S1_at	Hypothetical protein	-4.49
Mtr.38367.1.S1_at	Hypothetical protein	-4.49
Mtr.45269.1.S1_at	NADP dependent malic enzyme	-4.49
Mtr.20299.1.S1_at	U box protein	-4.49
Mtr.37015.1.S1_at	Hydroxymethylglutaryl-CoA lyase	-4.48
Mtr.8824.1.S1_at	CONSTANS-like protein 2	-4.48
Mtr.51973.1.S1_at	Seed maturation protein	-4.48
Mtr.28632.1.S1_at	Hypothetical protein	-4.48
Mtr.6008.1.S1_at	Hypothetical protein	-4.48
Mtr.40550.1.S1_at	Hypothetical protein	-4.47
Mtr.38746.1.S1_at	Heat shock transcription factor	-4.47
Mtr.46876.1.S1_at	Hypothetical protein	-4.47
Mtr.9679.1.S1_at	Thioredoxin 3	-4.47
Mtr.52127.1.S1_at	2OG-Fe(II) oxygenase	-4.47
Mtr.38138.1.S1_at	Hypothetical protein	-4.47
Mtr.1427.1.S1_at	Hypothetical protein	-4.46
Mtr.13972.1.S1_at	Thioredoxin m	-4.46
Msa.2749.1.S1_at	Hypothetical protein	-4.45
Mtr.40684.1.S1_at	Anthocyanin 5-aromatic acyltransferase/benzoyltransferase	-4.45
Mtr.10656.1.S1_at	Sucrose-phosphate synthase	-4.45
Mtr.32848.1.S1_at	Hypothetical protein	-4.45
Mtr.19577.1.S1_s_at	Aldehyde decarbonylase	-4.45
Mtr.9501.1.S1_at	Lysine decarboxylase	-4.44
Mtr.20207.1.S1_at	Thioredoxin	-4.44
Msa.1725.1.S1_at	Hypothetical protein	-4.44
Mtr.34453.1.S1_at	1-aminocyclopropane-1-carboxylate synthase	-4.44
Mtr.3869.1.S1_at	Hypothetical protein	-4.44
Msa.3191.1.S1_s_at	Hypothetical protein	-4.44
Mtr.43269.1.S1_at	30S ribosomal protein S1	-4.44
Mtr.32057.1.S1_at	Hypothetical protein	-4.43
Mtr.48712.1.S1_s_at	Facilitated glucose transporter	-4.43
Mtr.41066.1.S1_at	Hypothetical protein	-4.43
Mtr.2062.1.S1_at	Hypothetical protein	-4.43
Mtr.10212.1.S1_at	Hypothetical protein	-4.43
Mtr.38223.1.S1_at	Acyltransferase	-4.42
Mtr.29529.1.S1_at	Hypothetical protein	-4.42
Mtr.13184.1.S1_at	Prolyl carboxypeptidase like protein	-4.42
Mtr.5954.1.S1_at	ABC transporter	-4.42
Mtr.33686.1.S1_at	Hypothetical protein	-4.42
Mtr.10936.1.S1_at	Transcriptional regulator, LysR family	-4.42
Mtr.50527.1.S1_at	Hypothetical protein	-4.41
Mtr.49348.1.S1_s_at	Proteinase inhibitor I25	-4.41
Mtr.1424.1.S1_at	Hypothetical protein	-4.41
Mtr.4814.1.S1_at	AROGP2	-4.41
Mtr.43889.1.S1_at	Beta-1,3-glucanase	-4.41
Msa.1230.1.S1_at	Hypothetical protein	-4.41
Mtr.32117.1.S1_at	Hypothetical protein	-4.40
Msa.1490.1.S1_at	Hypothetical protein	-4.40

Mtr.38303.1.S1_at	Hypothetical protein	-4.40
Mtr.44684.1.S1_at	Nucleotide pyrophosphatase	-4.39
Mtr.9616.1.S1_at	Hypothetical protein	-4.39
Msa.1588.1.S1_at	Hypothetical protein	-4.39
Mtr.10607.1.S1_at	PS60 protein	-4.39
Mtr.12858.1.S1_at	Hypothetical protein	-4.39
Msa.1651.1.S1_at	Hypothetical protein	-4.39
Mtr.14519.1.S1_a_at	RNA-binding region RNP-1	-4.38
Mtr.41249.1.S1_at	30S ribosomal protein S13	-4.38
Mtr.43590.1.S1_at	Hypothetical protein	-4.38
Mtr.44918.1.S1_at	Lysophospholipase 1	-4.38
Mtr.33007.1.S1_at	Hypothetical protein	-4.38
Mtr.5253.1.S1_s_at	Hypothetical protein	-4.38
Mtr.28159.1.S1_at	AP2 domain transcription factor	-4.38
Mtr.31078.1.S1_at	CPD photolyase	-4.38
Mtr.44666.1.S1_at	Carboxyl terminal protease	-4.37
Mtr.43826.1.S1_at	Hypothetical protein	-4.37
Mtr.20860.1.S1_at	Hypothetical protein	-4.37
Mtr.1559.1.S1_s_at	Calcineurin B-like protein 10	-4.37
Mtr.32683.1.S1_at	Tetratricopeptide repeat (TPR)-containing protein	-4.37
Mtr.19693.1.S1_at	Zinc-containing alcohol dehydrogenase	-4.37
Mtr.40232.1.S1_s_at	Geranylgeranyl hydrogenase	-4.37
Mtr.9173.1.S1_at	Hypothetical protein	-4.37
Mtr.40525.1.S1_at	Hypothetical protein	-4.36
Mtr.22479.1.S1_at	Anthocyanin 1	-4.36
Mtr.34131.1.S1_at	NADP dependent malic enzyme	-4.36
Mtr.47321.1.S1_s_at	DNA-directed RNA polymerase beta chain	-4.36
Mtr.48754.1.S1_at	Dihydrodipicolinate reductase	-4.35
Mtr.10104.1.S1_at	Ornithine decarboxylase	-4.35
Mtr.8397.1.S1_at	Anthranilate synthase alpha subunit	-4.35
Mtr.37955.1.S1_at	Hypothetical protein	-4.35
Mtr.40305.1.S1_at	Hypothetical protein	-4.35
Mtr.49527.1.S1_at	Lipolytic enzyme	-4.35
Msa.2740.1.S1_at	Hypothetical protein	-4.35
Mtr.37930.1.S1_at	Hypothetical protein	-4.35
Mtr.11591.1.S1_at	Constitutively photomorphogenic 1 protein	-4.35
Mtr.7633.1.S1_at	Hypothetical protein	-4.35
Mtr.28691.1.S1_at	Nine-cis-epoxycarotenoid dioxygenase3	-4.34
Mtr.39369.1.S1_at	33 kd chloroplast ribonucleoprotein	-4.34
Mtr.40558.1.S1_at	Short-chain alcohol dehydrogenase	-4.34
Mtr.50015.1.S1_s_at	Hypothetical protein	-4.34
Mtr.44759.1.S1_at	E-beta-ocimene synthase	-4.34
Mtr.35494.1.S1_at	Hypothetical protein	-4.33
Mtr.16234.1.S1_at	Hypothetical protein	-4.33
Msa.3079.1.S1_at	Hypothetical protein	-4.33
Mtr.42503.1.S1_at	Isoflavonoid glucosyltransferase	-4.33
Msa.1903.1.S1_at	Hypothetical protein	-4.33
Mtr.10800.1.S1_at	Hypothetical protein	-4.33
Mtr.9062.1.S1_at	Hypothetical protein	-4.33
Mtr.5351.1.S1_at	Cuticle protein (Faceless pollen-1) (YORE-YORE protein)	-4.33
Mtr.48631.1.S1_at	Sugar transporter	-4.33
Msa.3021.1.S1_at	Hypothetical protein	-4.32
Mtr.49348.1.S1_at	Proteinase inhibitor I25	-4.32
Mtr.40297.1.S1_at	Cytochrome b6f complex subunit	-4.32

Mtr.37072.1.S1_at	Hypothetical protein	-4.32
Mtr.3201.1.S1_at	LysM domain-containing receptor-like kinase 1	-4.31
Msa.1138.1.S1_at	Hypothetical protein	-4.31
Mtr.31183.1.S1_x_at	Long-chain-fatty-acid--CoA ligase 6	-4.31
Msa.3039.1.S1_at	Hypothetical protein	-4.31
Mtr.38360.1.S1_at	Hypothetical protein	-4.31
Mtr.41054.1.S1_at	UDP-glucose glucosyltransferase	-4.31
Mtr.49132.1.S1_at	Ferredoxin	-4.31
Mtr.762.1.S1_at	2OG-Fe(II) oxygenase	-4.30
Mtr.21135.1.S1_at	2OG-Fe(II) oxygenase	-4.30
Mtr.8254.1.S1_at	Hypothetical protein	-4.30
Mtr.13924.1.S1_at	Glucosyltransferase like protein	-4.29
Mtr.9820.1.S1_at	MADS box protein	-4.29
Mtr.20814.1.S1_at	Zn-finger, CCHC type	-4.29
Mtr.44890.1.S1_at	Calcium binding protein	-4.29
Mtr.42512.1.S1_at	Sugar transporter	-4.28
Mtr.41591.1.S1_at	3-ketoacyl-ACP synthase	-4.28
Mtr.24188.1.S1_at	Hypothetical protein	-4.28
Mtr.19007.1.S1_at	Barwin-related endoglucanase	-4.28
Mtr.3319.1.S1_s_at	Hypothetical protein	-4.28
Mtr.37341.1.S1_at	Mannitol dehydrogenase	-4.28
Mtr.34220.1.S1_at	Hypothetical protein	-4.28
Mtr.8830.1.S1_at	Hypothetical protein	-4.28
Mtr.50857.1.S1_s_at	Peptidase S10	-4.28
Mtr.1962.1.S1_at	MRP-like ABC transporter	-4.28
Mtr.11881.1.S1_at	Hypothetical protein	-4.27
Mtr.9049.1.S1_at	Sbp65a protein	-4.27
Mtr.40759.1.S1_at	TatC	-4.26
Mtr.5456.1.S1_at	Hypothetical protein	-4.26
Mtr.44147.1.S1_at	Lipid transfer protein	-4.26
Mtr.37091.1.S1_at	Hypothetical protein	-4.26
Mtr.21709.1.S1_at	Hypothetical protein	-4.26
Mtr.11319.1.S1_at	Hypothetical protein	-4.26
Mtr.35041.1.S1_s_at	Glutamate receptor 3.3	-4.26
Mtr.8775.1.S1_at	Hypothetical protein	-4.25
Msa.1739.1.S1_at	Hypothetical protein	-4.24
Mtr.21146.1.S1_at	Zn-finger, RING	-4.24
Msa.1400.1.S1_at	Hypothetical protein	-4.24
Mtr.24083.1.S1_s_at	Uroporphyrinogen decarboxylase	-4.24
Mtr.43288.1.S1_at	Diphenol oxidase	-4.24
Mtr.44223.1.S1_at	Mitochondrial aspartate-glutamate carrier protein	-4.23
Msa.1977.1.S1_at	Hypothetical protein	-4.23
Mtr.33530.1.S1_at	Hypothetical protein	-4.22
Mtr.25580.1.S1_at	Hypothetical protein	-4.22
Mtr.20125.1.S1_at	Glycoside hydrolase	-4.22
Mtr.12617.1.S1_at	Hypothetical protein	-4.22
Mtr.27444.1.S1_at	Hypothetical protein	-4.22
Msa.3181.1.S1_at	Hypothetical protein	-4.22
Mtr.39481.1.S1_at	Hypothetical protein	-4.21
Mtr.48937.1.S1_at	Hypothetical protein	-4.21
Mtr.38815.1.S1_at	Pair-rule protein	-4.21
Mtr.40653.1.S1_at	Hypothetical protein	-4.21
Mtr.1068.1.S1_s_at	Hypothetical protein	-4.21
Mtr.43598.1.S1_at	Polygalacturonase-like protein	-4.20

Mtr.42284.1.S1_at	Hypothetical protein	-4.20
Mtr.47389.1.S1_at	Hypothetical protein	-4.20
Mtr.3096.1.S1_at	Glycosyl hydrolase	-4.19
Mtr.38410.1.S1_at	bZIP50 protein	-4.19
Mtr.42658.1.S1_at	Ski protein	-4.19
Mtr.41864.1.S1_at	Hypothetical protein	-4.19
Mtr.34410.1.S1_at	Hypothetical protein	-4.19
Mtr.8908.1.S1_at	Hypothetical protein	-4.18
Msa.2828.1.S1_at	Hypothetical protein	-4.18
Mtr.31151.1.S1_at	Hypothetical protein	-4.17
Mtr.24230.1.S1_at	Isoflavone reductase	-4.17
Mtr.21515.1.S1_at	Cellulose synthase	-4.16
Mtr.33410.1.S1_at	Hypothetical protein	-4.16
Mtr.18494.1.S1_at	ZF-HD homeobox protein	-4.16
Mtr.10248.1.S1_at	Peroxidase precursor	-4.16
Mtr.35286.1.S1_at	Twist related protein 1	-4.16
Mtr.50199.1.S1_at	Hypothetical protein	-4.16
Mtr.17661.1.S1_at	Polyprenyl synthetase	-4.16
Mtr.12407.1.S1_at	Glutamate decarboxylase	-4.15
Mtr.32828.1.S1_at	Nodulin-like protein	-4.15
Mtr.39920.1.S1_at	Receptor like protein	-4.15
Mtr.13998.1.S1_at	Hypothetical protein	-4.14
Mtr.10045.1.S1_at	Hypothetical protein	-4.14
Mtr.6279.1.S1_at	LIM domain containing protein	-4.14
Mtr.13942.1.S1_at	Polyphosphoinositide binding protein	-4.14
Mtr.44469.1.S1_at	Prolyl carboxypeptidase	-4.14
Mtr.45048.1.S1_s_at	Hypothetical protein	-4.13
Mtr.48994.1.S1_at	Hypothetical protein	-4.13
Mtr.10312.1.S1_at	Phytochelatin synthetase-like protein	-4.13
Msa.2987.1.S1_at	Hypothetical protein	-4.13
Mtr.17362.1.S1_at	Beta-Ig-H3/fasciclin	-4.12
Mtr.43311.1.S1_at	BURP domain-containing protein	-4.12
Mtr.13829.1.S1_at	Hypothetical protein	-4.12
Mtr.43596.1.S1_at	Plastid ribosomal protein L19	-4.11
Mtr.35162.1.S1_at	Hypothetical protein	-4.11
Mtr.51697.1.S1_at	Beta-lactamase	-4.11
Mtr.31658.1.S1_s_at	GAI-like protein	-4.11
Mtr.11542.1.S1_s_at	Hypothetical protein	-4.11
Mtr.20633.1.S1_at	General substrate transporter	-4.11
Mtr.6765.1.S1_at	Hypothetical protein	-4.11
Mtr.9912.1.S1_at	Hypothetical protein	-4.11
Mtr.8443.1.S1_at	Hypothetical protein	-4.11
Mtr.34112.1.S1_at	Ferredoxin-thioredoxin reductase	-4.11
Mtr.4867.1.S1_s_at	Hypothetical protein	-4.11
Mtr.24418.1.S1_at	Cytochrome P450	-4.11
Mtr.15549.1.S1_at	Hypothetical protein	-4.10
Mtr.40695.1.S1_s_at	Hypothetical protein	-4.10
Mtr.43518.1.S1_at	Plastidic cysteine synthase 1	-4.10
Mtr.36903.1.S1_at	C1C-Nt1 protein	-4.10
Mtr.7553.1.S1_at	Alanine acetyl transferase	-4.10
Mtr.44280.1.S1_at	Avr9/Cf-9 induced kinase 1	-4.10
Mtr.45399.1.S1_at	Hypothetical protein	-4.10
Mtr.6706.1.S1_at	Hypothetical protein	-4.10
Mtr.4975.1.S1_s_at	SRG1 protein	-4.10

Msa.473.1.S1_at	Hypothetical protein	-4.09
Mtr.9483.1.S1_at	Hypothetical protein	-4.09
Mtr.10331.1.S1_at	Orcinol O-methyltransferase	-4.08
Mtr.19679.1.S1_s_at	Phospholipase/Carboxylesterase	-4.08
Mtr.13051.1.S1_at	Glucose-6-phosphate 1-dehydrogenase 1	-4.08
Mtr.37175.1.S1_at	Chlorophyll a/b binding protein	-4.08
Mtr.37696.1.S1_at	Scarecrow-like 6	-4.07
Mtr.43082.1.S1_at	Omega-6 fatty acid desaturase	-4.07
Mtr.35210.1.S1_at	Kinase like protein	-4.06
Mtr.45418.1.S1_at	Hypothetical protein	-4.06
Mtr.32736.1.S1_at	Hypothetical protein	-4.06
Mtr.13236.1.S1_at	Glucose-6-phosphate dehydrogenase	-4.06
Mtr.10705.1.S1_at	GPI-anchored protein	-4.05
AFFX-Mtr-gsta-M_x_at	2,4-D inducible glutathione S-transferase	-4.05
Mtr.43200.1.S1_at	Adenosine 5'-phosphosulfate reductase	-4.05
Mtr.49874.1.S1_at	Zn-finger, RING	-4.05
Mtr.9857.1.S1_at	Hypothetical protein	-4.05
Mtr.24535.1.S1_at	Peroxidase	-4.05
Mtr.37893.1.S1_s_at	Cytochrome P450 monooxygenase	-4.05
Mtr.37615.1.S1_at	Hypothetical protein	-4.05
Mtr.6751.1.S1_at	Hypothetical protein	-4.05
Mtr.31344.1.S1_at	Oxidoreductase	-4.04
Mtr.32315.1.S1_at	Hypothetical protein	-4.04
Mtr.18567.1.S1_at	Pyruvate kinase	-4.04
Mtr.13750.1.S1_at	Eent-kaurenoic acid hydroxylase	-4.04
Mtr.27829.1.S1_at	Heat shock factor protein hsf8	-4.04
Mtr.43479.1.S1_at	Hypothetical protein	-4.03
Mtr.51015.1.S1_at	Hypothetical protein	-4.03
Mtr.9347.1.S1_at	Hypothetical protein	-4.03
Mtr.33661.1.S1_at	Subtilisin-like serine proteinase	-4.03
Mtr.38643.1.S1_s_at	Sucrose-phosphate synthase	-4.03
Mtr.11974.1.S1_at	Hypothetical protein	-4.03
Mtr.12889.1.S1_at	ATPase-like protein	-4.03
Msa.1696.1.S1_at	Hypothetical protein	-4.03
Mtr.4862.1.S1_at	Oxidoreductase	-4.03
Mtr.7840.1.S1_at	Hypothetical protein	-4.03
Msa.1612.1.S1_at	Hypothetical protein	-4.02
Mtr.2337.1.S1_at	Hypothetical protein	-4.02
Mtr.14519.1.S1_at	RNA-binding region RNP-1	-4.02
Mtr.40308.1.S1_at	Monofunctional aspartokinase	-4.02
Mtr.36799.1.S1_s_at	Hypothetical protein	-4.02
Mtr.31405.1.S1_at	CDR1	-4.02
Mtr.31183.1.S1_at	Long-chain-fatty-acid--CoA ligase 6	-4.02
Mtr.223.1.S1_at	Photolyase/blue-light receptor	-4.01
Mtr.8707.1.S1_at	Hypothetical protein	-4.01
Mtr.12803.1.S1_at	Pyridoxamine 5-phosphate oxidase	-4.01
Mtr.37164.1.S1_at	Heat shock factor	-4.01
Mtr.24495.1.S1_at	Photosystem II 32 kDa protein	-4.01
Mtr.43000.1.S1_at	Hypothetical protein	-4.01
Mtr.6500.1.S1_at	Alcohol NADP+ oxidoreductase	-4.01
Mtr.27067.1.S1_at	DNA binding with one finger	-4.01
Mtr.51659.1.S1_at	Hypothetical protein	-4.01
Mtr.43904.1.S1_at	Cyclase-like protein	-4.01
Mtr.41461.1.S1_at	Hypothetical protein	-4.00

Mtr.45126.1.S1_at	Hypothetical protein	-4.00
Mtr.37315.1.S1_at	Glycolate oxidase	-4.00
Mtr.49120.1.S1_s_at	Cysteine synthase A	-4.00
Mtr.32523.1.S1_at	Peptide transporter	-4.00
Mtr.13752.1.S1_at	Calcineurin B-like protein 10	-4.00
Mtr.14341.1.S1_at	Calponin-like actin-binding protein	-4.00
Mtr.15256.1.S1_at	RNA-binding region RNP-1	-4.00
Msa.1866.1.S1_s_at	Hypothetical protein	-3.99
Mtr.50712.1.S1_at	Hypothetical protein	-3.99
Mtr.42622.1.S1_s_at	Maturase	-3.99
Msa.1405.1.S1_at	Hypothetical protein	-3.99
Mtr.15321.1.S1_at	Serine/threonine protein phosphatase and bis(5-nucleosyl)-tetraphosphatase	-3.99
Mtr.7389.1.S1_s_at	Yippee-like protein	-3.99
Mtr.21847.1.S1_at	Hypothetical protein	-3.99
Mtr.37666.1.S1_at	Hypothetical protein	-3.99
Msa.2217.1.S1_at	Hypothetical protein	-3.99
Mtr.25510.1.S1_at	Glycosyl hydrolase	-3.99
Mtr.44402.1.S1_at	Hypothetical protein	-3.99
Mtr.26041.1.S1_at	Hypothetical protein	-3.98
Mtr.36799.1.S1_at	Hypothetical protein	-3.98
Mtr.4063.1.S1_at	Hypothetical protein	-3.98
Mtr.39361.1.S1_at	Glucanase like protein	-3.98
Mtr.43749.1.S1_s_at	Hypothetical protein	-3.98
Mtr.1770.1.S1_at	Protein kinase	-3.98
Mtr.6062.1.S1_at	Deoxyribonuclease II	-3.98
Mtr.39386.1.S1_at	Hypothetical protein	-3.98
Mtr.9652.1.S1_at	Hypothetical protein	-3.98
Mtr.43995.1.S1_at	Hypothetical protein	-3.97
Mtr.9808.1.S1_at	Hypothetical protein	-3.97
Mtr.10767.1.S1_at	Agmatine Iminohydrolase	-3.97
Mtr.12679.1.S1_at	Peroxiredoxin-like protein	-3.97
Msa.1951.1.S1_s_at	Hypothetical protein	-3.97
Mtr.42054.1.S1_at	Glu-tRNA(Gln) amidotransferase subunit A	-3.97
Mtr.43600.1.S1_at	Hypothetical protein	-3.97
Mtr.7035.1.S1_at	Hypothetical protein	-3.97
Mtr.7071.1.S1_at	Alcohol oxidase	-3.97
Mtr.33925.1.S1_at	Hypothetical protein	-3.97
Mtr.51665.1.S1_s_at	Photosynthetic reaction centre protein	-3.97
Mtr.21631.1.S1_at	Peptide chain release factor 2	-3.96
Mtr.39702.1.S1_at	Hypothetical protein	-3.96
Mtr.8968.1.S1_at	Hypothetical protein	-3.96
Mtr.9665.1.S1_at	Oikosin 4A protein	-3.96
Mtr.10227.1.S1_at	Cytochrome P450-like protein	-3.96
Mtr.10332.1.S1_at	Class Ib chitinase	-3.96
Mtr.43824.1.S1_at	Thioredoxin M-type 3	-3.96
Msa.1751.1.S1_at	Hypothetical protein	-3.96
Msa.1814.1.S1_at	Hypothetical protein	-3.95
Mtr.115.1.S1_at	Hypothetical protein	-3.95
Mtr.16995.1.S1_at	Hypothetical protein	-3.95
Mtr.42539.1.S1_at	Hypothetical protein	-3.95
Mtr.34879.1.S1_at	Hypothetical protein	-3.95
Mtr.5449.1.S1_at	Hypothetical protein	-3.95
Mtr.34451.1.S1_at	Hypothetical protein	-3.95
Mtr.5356.1.S1_s_at	Beta-mannosidase	-3.95

Mtr.31794.1.S1_s_at	Hypothetical protein	-3.95
Mtr.10926.1.S1_at	Thylakoid lumenal 17.4 kDa protein	-3.94
Mtr.40998.1.S1_at	Hypothetical protein	-3.94
Mtr.1621.1.S1_at	Hypothetical protein	-3.94
Mtr.27403.1.S1_at	Hypothetical protein	-3.94
Mtr.38209.1.S1_at	Polysaccharide ABC transporter	-3.94
Mtr.31189.1.S1_at	Trehalose-6-phosphate phosphatase	-3.94
Mtr.6074.1.S1_at	Valine-tRNA ligase	-3.94
Mtr.33779.1.S1_at	Threonyl-tRNA synthetase	-3.93
Mtr.5452.1.S1_at	ABC transporter	-3.93
Mtr.33726.1.S1_at	Sugar transporter	-3.93
Msa.1835.1.S1_at	Hypothetical protein	-3.93
Mtr.9095.1.S1_at	30S ribosomal protein S9	-3.93
Mtr.5125.1.S1_s_at	Isoflavone reductase	-3.93
Mtr.12359.1.S1_at	enod40 mRNA for non-translatable RNA	-3.93
Mtr.31487.1.S1_s_at	Agmatine Iminohydrolase	-3.93
Msa.2707.1.S1_at	Hypothetical protein	-3.93
Mtr.10025.1.S1_at	Phosphoribosylanthranilate transferase-like protein	-3.93
Mtr.34499.1.S1_at	Hypothetical protein	-3.92
Mtr.31664.1.S1_at	Hypothetical protein	-3.92
Mtr.11249.1.S1_s_at	Calcineurin B-like protein 10	-3.92
Mtr.43492.1.S1_at	Hypothetical protein	-3.92
Mtr.6699.1.S1_at	Lhcb2 protein (Light harvesting chlorophyll a/b-binding protein)	-3.92
Mtr.40976.1.S1_at	Transcription factor Hap5a	-3.92
Mtr.9854.1.S1_at	Hypothetical protein	-3.91
Mtr.33742.1.S1_at	Acyl-CoA thioesterase	-3.91
Mtr.50217.1.S1_s_at	AFG1-like ATPase	-3.91
Mtr.42769.1.S1_at	Hypothetical protein	-3.91
Mtr.15971.1.S1_at	Glycine cleavage T protein	-3.90
Mtr.33769.1.S1_s_at	Prolyl carboxypeptidase	-3.90
Mtr.9861.1.S1_at	Hypothetical protein	-3.90
Mtr.49014.1.S1_at	SNO glutamine amidotransferase	-3.90
Mtr.9263.1.S1_at	Glycine-tRNA ligase	-3.90
Mtr.2661.1.S1_at	Serine acetyltransferase	-3.90
Msa.1021.1.S1_at	Hypothetical protein	-3.90
Mtr.44584.1.S1_at	Transfactor-like protein	-3.90
Mtr.16432.1.S1_at	Myb, DNA-binding	-3.89
Mtr.25689.1.S1_at	UDP-glucose 6-dehydrogenase	-3.89
Mtr.10665.1.S1_at	Hypothetical protein	-3.89
Mtr.24561.1.S1_s_at	UDP-glycosyltransferase	-3.89
Mtr.10873.1.S1_at	Hypothetical protein	-3.89
Mtr.9105.1.S1_at	Coproporphyrinogen oxidase	-3.89
Mtr.42749.1.S1_at	Late embryogenesis abundant protein 1	-3.88
Mtr.24331.1.S1_s_at	Hypothetical protein	-3.88
Mtr.6750.1.S1_at	Serine carboxypeptidase	-3.88
Mtr.5788.1.S1_s_at	Hypothetical protein	-3.88
Mtr.34815.1.S1_at	Hypothetical protein	-3.88
Mtr.44320.1.S1_at	Hypothetical protein	-3.87
Mtr.29176.1.S1_at	Metal-dependent phosphohydrolase HD domain-containing protein	-3.87
Mtr.31537.1.S1_at	Hypothetical protein	-3.87
Mtr.9268.1.S1_at	Hypothetical protein	-3.87
Mtr.32795.1.S1_s_at	Hypothetical protein	-3.86
Mtr.32230.1.S1_at	Glycine-sarcosine methyltransferase	-3.86
Mtr.27907.1.S1_at	Pectate lyase	-3.86

Mtr.13579.1.S1_at	Photoreceptor-interacting transcription factor	-3.86
Mtr.3013.1.S1_at	UDP-glucuronosyltransferase	-3.86
Msa.1661.1.S1_at	Hypothetical protein	-3.86
Mtr.4981.1.S1_at	Dehydration-responsive protein	-3.86
Mtr.33111.1.S1_at	Hypothetical protein	-3.86
Mtr.32670.1.S1_at	UDP-glucose:salicylic acid glucosyltransferase	-3.86
Mtr.2881.1.S1_at	Hypothetical protein	-3.86
Mtr.42999.1.S1_at	Photosystem I psaH	-3.85
Mtr.34594.1.S1_s_at	Thylakoid lumenal 17.4 kDa protein	-3.85
Mtr.40506.1.S1_at	Serine carboxypeptidase II	-3.85
Mtr.33014.1.S1_s_at	Hypothetical protein	-3.85
Mtr.5656.1.S1_at	Vacuolar H+-ATPase B subunit	-3.85
Mtr.52119.1.S1_at	Protein phosphatase 2C	-3.84
Mtr.2385.1.S1_at	Hypothetical protein	-3.84
Mtr.19691.1.S1_at	Hypothetical protein	-3.84
Mtr.48864.1.S1_at	Zn-finger, Dof type	-3.84
Mtr.37352.1.S1_at	Trypsin protein inhibitor 3	-3.84
Msa.2735.1.S1_at	Hypothetical protein	-3.84
Mtr.38283.1.S1_at	Hypothetical protein	-3.84
Mtr.37927.1.S1_at	Hypothetical protein	-3.84
Mtr.11721.1.S1_at	Lycopene epsilon-cyclase	-3.84
Mtr.34395.1.S1_at	Hypothetical protein	-3.83
Mtr.46510.1.S1_at	Alpha/beta-amyrin synthase	-3.83
Mtr.36918.1.S1_at	Hypothetical protein	-3.83
Msa.1716.1.S1_at	Hypothetical protein	-3.83
Mtr.40695.1.S1_at	Hypothetical protein	-3.83
Mtr.43250.1.S1_at	Subtilisin-like protease	-3.83
Mtr.28655.1.S1_at	Hypothetical protein	-3.83
Mtr.29249.1.S1_at	Hypothetical protein	-3.83
Mtr.44847.1.S1_at	Glycosyltransferase family protein	-3.83
Mtr.12787.1.S1_at	Membrane-associated 30 kDa protein	-3.82
Mtr.28729.1.S1_at	Serine protease	-3.82
Mtr.20942.1.S1_x_at	Serine/threonine protein kinase	-3.82
Mtr.32655.1.S1_at	Hypothetical protein	-3.82
Mtr.37821.1.S1_at	Hypothetical protein	-3.82
Mtr.2587.1.S1_at	Membrane-associated salt-inducible protein	-3.82
Mtr.33734.1.S1_at	Phosphoglycerate mutase	-3.82
Mtr.50700.1.S1_s_at	Serine/threonine protein kinase	-3.82
Mtr.9977.1.S1_at	Hypothetical protein	-3.81
Mtr.31819.1.S1_at	UDP rhamnose	-3.81
Mtr.7957.1.S1_at	Hypothetical protein	-3.81
Mtr.50814.1.S1_at	Hypothetical protein	-3.81
Mtr.41311.1.S1_at	Hypothetical protein	-3.81
Mtr.27078.1.S1_at	Selenium-binding protein	-3.81
Mtr.51464.1.S1_at	Hypothetical protein	-3.81
Mtr.26276.1.S1_at	CER1 protein	-3.81
Mtr.13501.1.S1_at	Alanyl-tRNA synthetase	-3.81
Mtr.14002.1.S1_at	Calcium binding protein	-3.81
Mtr.10876.1.S1_at	Hypothetical protein	-3.80
Mtr.27872.1.S1_at	ATPase	-3.80
Mtr.4684.1.S1_s_at	Hypothetical protein	-3.80
Mtr.39477.1.S1_at	Myosin-like protein	-3.80
Mtr.22763.1.S1_at	Hypothetical protein	-3.79
Mtr.17972.1.S1_at	Glycoside hydrolase	-3.79

Mtr.13783.1.S1_at	Hypothetical protein	-3.79
Mtr.3440.1.S1_at	Hypothetical protein	-3.79
Mtr.19629.1.S1_at	Hypothetical protein	-3.79
Mtr.44465.1.S1_at	Amino acid transporter	-3.79
Mtr.26277.1.S1_at	Lycopene beta-cyclase	-3.79
Mtr.14580.1.S1_at	Zn-finger, RING	-3.79
Mtr.8926.1.S1_at	Alpha-D-xylosidase	-3.79
Mtr.36687.1.S1_at	Hypothetical protein	-3.78
Mtr.40975.1.S1_at	Nitrite transport protein	-3.78
Mtr.44765.1.S1_at	Lycopene epsilon cyclase	-3.78
Mtr.38225.1.S1_at	Arm repeat-containing protein	-3.78
Mtr.24451.1.S1_at	Hypothetical protein	-3.78
Mtr.11518.1.S1_at	Hypothetical protein	-3.78
Mtr.39462.1.S1_at	Glu-tRNA(Gln) amidotransferase subunit A	-3.78
Mtr.17822.1.S1_at	Cytochrome P450	-3.78
Msa.1046.1.S1_at	Hypothetical protein	-3.77
Mtr.9325.1.S1_at	Hypothetical protein	-3.77
Mtr.15048.1.S1_s_at	HAD-superfamily subfamily IIA hydrolase	-3.77
Mtr.6354.1.S1_at	Tetratricopeptide repeat (TPR)-containing protein	-3.77
Mtr.39039.1.S1_at	Ankyrin like protein	-3.77
Mtr.29065.1.S1_at	GTP cyclohydrolase I	-3.77
Mtr.40639.1.S1_at	Hypothetical protein	-3.77
Mtr.5058.1.S1_at	Cryptochrome 1	-3.77
Mtr.43528.1.S1_at	Fructokinase 3	-3.76
Mtr.5171.1.S1_at	Hypothetical protein	-3.76
Mtr.37464.1.S1_at	Hypothetical protein	-3.76
Mtr.32351.1.S1_at	Hypothetical protein	-3.76
Mtr.5236.1.S1_s_at	Hypothetical protein	-3.76
Mtr.5850.1.S1_at	Protein kinase	-3.76
Mtr.12644.1.S1_at	Fiber protein Fb34	-3.76
Mtr.10288.1.S1_at	Kinase like protein	-3.76
Mtr.39152.1.S1_at	Mitochondrial half-ABC transporter	-3.76
Mtr.42188.1.S1_at	Yippee-like protein	-3.76
Mtr.31137.1.S1_at	Hypothetical protein	-3.76
Mtr.15596.1.S1_s_at	C2 calcium/lipid-binding protein	-3.76
Mtr.16273.1.S1_at	Proteinase inhibitor I12	-3.75
Mtr.33519.1.S1_at	Cold acclimation responsive protein	-3.75
Mtr.8447.1.S1_s_at	Hypothetical protein	-3.75
Mtr.8728.1.S1_at	Hypothetical protein	-3.75
Mtr.23870.1.S1_at	Hypothetical protein	-3.75
Mtr.11939.1.S1_at	RelA-SpoT like protein RSH1	-3.75
Mtr.6773.1.S1_at	Myosin heavy chain-like protein	-3.75
Msa.399.1.S1_at	Hypothetical protein	-3.74
Mtr.6087.1.S1_at	Hypothetical protein	-3.74
Mtr.5638.1.S1_at	Hypothetical protein	-3.74
Mtr.50799.1.S1_s_at	Cupredoxin	-3.74
Mtr.8778.1.S1_at	Hypothetical protein	-3.74
Mtr.13885.1.S1_at	Actin-like protein	-3.74
Mtr.40987.1.S1_at	Hypothetical protein	-3.74
Mtr.42200.1.S1_s_at	Protein kinase APK1B	-3.74
Mtr.10186.1.S1_at	Hypothetical protein	-3.74
Mtr.42216.1.S1_at	RanGAP1 interacting protein	-3.73
Mtr.27440.1.S1_at	Hypothetical protein	-3.73
Mtr.23946.1.S1_at	Receptor kinase	-3.73

Mtr.11841.1.S1_at	Lecithine cholesterol acyltransferase	-3.73
Mtr.13573.1.S1_at	Cleavage stimulation factor 50K	-3.73
Mtr.39047.1.S1_at	RNA helicase-like protein	-3.73
Mtr.41057.1.S1_at	Hypothetical protein	-3.73
Mtr.48462.1.S1_s_at	Hypothetical protein	-3.73
Mtr.22047.1.S1_at	Hypothetical protein	-3.72
Mtr.44031.1.S1_at	Hypothetical protein	-3.72
Mtr.2596.1.S1_at	Protein kinase	-3.72
Mtr.18659.1.S1_s_at	Hly-III related proteins	-3.72
Mtr.18268.1.S1_at	Serine/threonine protein kinase	-3.72
Mtr.45093.1.S1_at	Beta-carotene hydroxylase	-3.72
Mtr.8750.1.S1_at	Homeobox protein knotted-1 like 3 (KNAP3)	-3.72
Mtr.1035.1.S1_at	Heat shock protein	-3.72
Mtr.44377.1.S1_at	Ferric leghemoglobin reductase	-3.72
Mtr.23937.1.S1_at	Hypothetical protein	-3.72
Mtr.13194.1.S1_s_at	RanGAP1 interacting protein	-3.72
Mtr.38268.1.S1_at	Hypothetical protein	-3.72
Mtr.27203.1.S1_at	Hypothetical protein	-3.71
Mtr.18673.1.S1_at	Esterase/lipase/thioesterase	-3.71
Mtr.40151.1.S1_at	Cellulose synthase	-3.71
Mtr.8220.1.S1_at	Hypothetical protein	-3.71
Msa.1378.1.S1_at	Hypothetical protein	-3.71
Mtr.37287.1.S1_s_at	Beta-glucosidase	-3.71
Mtr.7932.1.S1_s_at	Allyl alcohol dehydrogenase	-3.71
Mtr.17456.1.S1_at	Hypothetical protein	-3.71
Mtr.19730.1.S1_at	Microtubule-associated protein	-3.70
Mtr.6163.1.S1_at	Hypothetical protein	-3.70
Mtr.11277.1.S1_at	Hydroxymethylglutaryl-CoA lyase	-3.70
Mtr.12978.1.S1_at	3-hydroxyisobutyrate dehydrogenase	-3.70
Msa.1654.1.S1_at	Hypothetical protein	-3.70
Mtr.44678.1.S1_at	Hypothetical protein	-3.70
Msa.1619.1.S1_at	Hypothetical protein	-3.70
Mtr.2656.1.S1_at	Serine/threonine protein kinase	-3.70
Mtr.8254.1.S1_s_at	Hypothetical protein	-3.70
Msa.2287.1.S1_at	Hypothetical protein	-3.69
Mtr.16219.1.S1_at	Zn-finger, B-box	-3.69
Mtr.41837.1.S1_s_at	Hypothetical protein	-3.69
Mtr.45013.1.S1_at	Hypothetical protein	-3.69
Mtr.34418.1.S1_at	Hypothetical protein	-3.69
Mtr.35108.1.S1_s_at	NAP	-3.69
Mtr.14257.1.S1_at	Magnesium-protoporphyrin IX methyltransferase	-3.69
Mtr.40395.1.S1_at	MtN29 protein	-3.69
Mtr.37525.1.S1_at	Multifunctional aquaporin	-3.69
Mtr.38754.1.S1_at	Chloride channel protein	-3.69
Mtr.11364.1.S1_at	Cytochrome P450	-3.69
Mtr.40906.1.S1_at	Quinone oxidoreductase	-3.68
Mtr.15596.1.S1_at	C2 calcium/lipid-binding protein	-3.68
Mtr.1902.1.S1_at	Hypothetical protein	-3.68
Mtr.41950.1.S1_at	ABC transporter	-3.67
Mtr.28056.1.S1_at	Hypothetical protein	-3.67
Mtr.8275.1.S1_at	Calcium-transporting ATPase 9	-3.67
Mtr.5622.1.S1_at	Hypothetical protein	-3.67
Mtr.28571.1.S1_at	Phosphoribosylanthranilate transferase	-3.67
Mtr.35868.1.S1_at	Hypothetical protein	-3.67

Mtr.9685.1.S1_at	CAAX amino terminal protease	-3.67
Mtr.2083.1.S1_s_at	Glutamate decarboxylase 1	-3.67
Mtr.8967.1.S1_s_at	Hypothetical protein	-3.67
Mtr.44368.1.S1_at	Hypothetical protein	-3.67
Mtr.50278.1.S1_s_at	Small GTP-binding protein	-3.66
Mtr.42552.1.S1_at	Anthocyanidin-3-glucoside rhamnosyltransferase	-3.66
Mtr.28405.1.S1_at	General negative transcription regulator	-3.66
Mtr.23112.1.S1_at	Chloride channel-like (CLC) protein	-3.66
Mtr.13141.1.S1_at	Chloroplast nucleoid DNA binding protein	-3.66
Mtr.44038.1.S1_at	Hypothetical protein	-3.65
Mtr.11805.1.S1_at	Hypothetical protein	-3.65
Mtr.27970.1.S1_at	Hypothetical protein	-3.65
Mtr.23994.1.S1_at	Homeodomain transcription factor	-3.65
Mtr.9451.1.S1_at	Hypothetical protein	-3.65
Mtr.12148.1.S1_s_at	Hypothetical protein	-3.65
Mtr.12741.1.S1_at	Hypothetical protein	-3.65
Mtr.12001.1.S1_at	Imidazole-4-carboxamide isomerase	-3.65
Mtr.42432.1.S1_at	Hypothetical protein	-3.65
Mtr.43748.1.S1_s_at	Hypothetical protein	-3.65
Mtr.19564.1.S1_s_at	Hypothetical protein	-3.65
Mtr.10321.1.S1_at	Glycerophosphoryl diester phosphodiesterase 2	-3.64
Mtr.51597.1.S1_s_at	Hypothetical protein	-3.64
Mtr.29098.1.S1_at	Long chain acyl-CoA synthetase 5	-3.64
Mtr.712.1.S1_at	Thioredoxin	-3.64
Mtr.33880.1.S1_s_at	Hypothetical protein	-3.64
Mtr.28688.1.S1_at	Hypothetical protein	-3.64
Mtr.11631.1.S1_at	Chloroplast alpha-glucan water dikinase	-3.64
Mtr.33457.1.S1_s_at	Galactinol synthase	-3.64
Mtr.13230.1.S1_at	Glycosyl transferase	-3.64
Mtr.9018.1.S1_at	Tic20	-3.63
Mtr.38700.1.S1_at	Hypothetical protein	-3.63
Mtr.35622.1.S1_at	Hypothetical protein	-3.63
Mtr.41140.1.S1_at	Hypothetical protein	-3.63
Mtr.38761.1.S1_s_at	Cell division protein FtsH-like	-3.63
Mtr.49013.1.S1_at	Hypothetical protein	-3.63
Mtr.6112.1.S1_at	Cytochrome p450	-3.63
Mtr.36969.1.S1_at	Beta-galactosidase	-3.63
Mtr.42232.1.S1_at	Hypothetical protein	-3.63
Mtr.28232.1.S1_s_at	RNA helicase	-3.63
Mtr.6854.1.S1_at	Two-component system sensor histidine kinase	-3.63
Mtr.10887.1.S1_at	Hypothetical protein	-3.63
Mtr.12798.1.S1_at	Benzoyl coenzyme A: benzyl alcohol benzoyl transferase	-3.63
Mtr.785.1.S1_s_at	Chloroplast initiation factor 3	-3.63
Mtr.17598.1.S1_at	Hypothetical protein	-3.62
Mtr.13200.1.S1_at	Hypothetical protein	-3.62
Mtr.39445.1.S1_at	Polygalacturonase isoenzyme 1 beta subunit	-3.62
Mtr.28068.1.S1_at	Hypothetical protein	-3.62
Mtr.49845.1.S1_at	Hypothetical protein	-3.62
Mtr.39696.1.S1_at	Hypothetical protein	-3.62
Mtr.37613.1.S1_at	Annexin-like protein	-3.62
Mtr.43677.1.S1_at	50S ribosomal protein L5	-3.61
Mtr.9455.1.S1_at	Hypothetical protein	-3.61
Mtr.35247.1.S1_at	Aquaporin-like transmembrane channel protein	-3.61
Mtr.9554.1.S1_at	Histone H4	-3.61

Msa.1661.1.S1_s_at	Hypothetical protein	-3.61
Mtr.5029.1.S1_at	Hypothetical protein	-3.60
Mtr.30232.1.S1_at	Hypothetical protein	-3.60
Mtr.38649.1.S1_at	Seed maturation-like protein	-3.60
Mtr.13889.1.S1_at	Hypothetical protein	-3.60
Mtr.33046.1.S1_at	Hypothetical protein	-3.60
Mtr.10065.1.S1_at	Hypothetical protein	-3.60
Mtr.42344.1.S1_at	Hypothetical protein	-3.60
Mtr.37359.1.S1_at	50S ribosomal protein L18	-3.60
Mtr.4368.1.S1_at	Light harvesting protein	-3.59
Msa.2822.1.S1_at	Hypothetical protein	-3.59
Mtr.28082.1.S1_s_at	Hypothetical protein	-3.59
Mtr.45091.1.S1_at	ABC transporter	-3.59
Mtr.10403.1.S1_at	Photosystem I reaction center subunit IV A	-3.59
Mtr.48199.1.S1_at	Hypothetical protein	-3.59
Mtr.40397.1.S1_at	Cytochrome P450 78A3	-3.59
Mtr.2204.1.S1_at	Hypothetical protein	-3.59
Mtr.6937.1.S1_at	Hypothetical protein	-3.59
Mtr.45509.1.S1_at	Hypothetical protein	-3.59
Mtr.47145.1.S1_at	C2H2-type zinc finger protein	-3.59
Mtr.11444.1.S1_at	Receptor-like protein kinase	-3.59
Mtr.7796.1.S1_at	Hypothetical protein	-3.58
Mtr.11994.1.S1_at	ABC transporter	-3.58
Mtr.2197.1.S1_at	Hypothetical protein	-3.58
Mtr.12080.1.S1_s_at	Hypothetical protein	-3.58
Msa.2137.1.S1_at	Hypothetical protein	-3.58
Mtr.28234.1.S1_at	Hypothetical protein	-3.58
Mtr.14574.1.S1_at	Syntaxin	-3.58
Mtr.44971.1.S1_at	Hypothetical protein	-3.57
Mtr.7450.1.S1_at	Hypothetical protein	-3.57
Mtr.13869.1.S1_at	Calcium-activated outward-rectifying potassium channel 5	-3.57
Mtr.45329.1.S1_at	Protein kinase	-3.57
Mtr.43925.1.S1_at	Histone H2A	-3.57
Mtr.43749.1.S1_at	Hypothetical protein	-3.57
Mtr.9855.1.S1_at	Hypothetical protein	-3.57
Mtr.45240.1.S1_at	Hypothetical protein	-3.56
Mtr.34726.1.S1_at	Photosystem I reaction center subunit II	-3.56
Mtr.41782.1.S1_at	Hypothetical protein	-3.56
Mtr.44813.1.S1_at	Aromatic rich glycoprotein	-3.56
Mtr.10412.1.S1_at	Oxygen-evolving enhancer protein 3	-3.56
Mtr.22161.1.S1_at	Hypothetical protein	-3.56
Mtr.4282.1.S1_at	HAP3-like transcriptional-activator	-3.56
Mtr.29292.1.S1_at	Hypothetical protein	-3.56
Mtr.16358.1.S1_at	Glyoxalase/extradiol ring-cleavage dioxygenase	-3.55
Mtr.5808.1.S1_at	UDP-glycosyltransferase 89B2	-3.55
Mtr.33744.1.S1_s_at	Hypothetical protein	-3.55
Mtr.5919.1.S1_at	Hypothetical protein	-3.55
Mtr.49327.1.S1_at	Hypothetical protein	-3.55
Mtr.42606.1.S1_at	Hypothetical protein	-3.54
Mtr.10826.1.S1_at	Rho GDP dissociation inhibitor 1, complete	-3.54
Mtr.25077.1.S1_at	Hypothetical protein	-3.54
Mtr.9877.1.S1_at	Short-chain dehydrogenase/reductase	-3.54
Mtr.20947.1.S1_at	Serine/threonine protein kinase	-3.54
Mtr.39345.1.S1_at	Oligopeptidase B	-3.54

Mtr.11785.1.S1_at	Hypothetical protein	-3.54
Mtr.42121.1.S1_at	1-aminocyclopropane-1-carboxylate synthase	-3.54
Mtr.13372.1.S1_at	Hypothetical protein	-3.53
Mtr.152.1.S1_at	Vestitone reductase	-3.53
Mtr.36792.1.S1_at	Hypothetical protein	-3.53
Mtr.18262.1.S1_at	Hypothetical protein	-3.53
Mtr.28569.1.S1_s_at	Remorin	-3.53
Mtr.9163.1.S1_at	Hypothetical protein	-3.53
Mtr.12805.1.S1_at	Hypothetical protein	-3.53
Mtr.784.1.S1_s_at	Translation initiation factor	-3.53
Mtr.13736.1.S1_at	Senescence-associated protein SAG102	-3.53
Mtr.37692.1.S1_at	Glutathione-dependent formaldehyde dehydrogenase	-3.52
Mtr.8848.1.S1_at	Alpha-xylosidase	-3.52
Mtr.41151.1.S1_at	Cer2 protein	-3.52
Mtr.14183.1.S1_at	Annexin	-3.52
Msa.1301.1.S1_at	Hypothetical protein	-3.52
Mtr.40863.1.S1_at	Hypothetical protein	-3.52
Mtr.43488.1.S1_at	Hypothetical protein	-3.51
Mtr.5041.1.S1_s_at	Alcohol dehydrogenase 1	-3.51
Mtr.36992.1.S1_at	Seed maturation-like protein	-3.51
Msa.1697.1.S1_at	Hypothetical protein	-3.51
Mtr.25499.1.S1_s_at	NifU-related metallocluster assembly factor	-3.51
Mtr.12375.1.S1_at	Photosystem I reaction center subunit III	-3.51
Mtr.46659.1.S1_at	Hypothetical protein	-3.51
Mtr.18628.1.S1_at	Lipid-binding START	-3.51
Mtr.9599.1.S1_at	Hypothetical protein	-3.51
Msa.3017.1.S1_at	Hypothetical protein	-3.51
Mtr.27391.1.S1_at	N-acylethanolamine amidohydrolase	-3.51
Mtr.32192.1.S1_at	Hypothetical protein	-3.50
Mtr.50756.1.S1_at	Hypothetical protein	-3.50
Mtr.6108.1.S1_at	Hypothetical protein	-3.50
Mtr.11148.1.S1_at	Multi resistance protein	-3.50
Mtr.38545.1.S1_s_at	Kinesin 1 (Kinesin-like protein A)	-3.50
Mtr.40247.1.S1_at	Salicylic acid glucosyltransferase	-3.50
Mtr.42622.1.S1_at	Maturase	-3.50
Mtr.43815.1.S1_at	Hypothetical protein	-3.49
Msa.1941.1.S1_at	Hypothetical protein	-3.48
Mtr.37355.1.S1_at	GAI-like protein	-3.48
Mtr.15042.1.S1_at	Histidine kinase A	-3.48
Mtr.27421.1.S1_s_at	NADPH HC toxin reductase	-3.48
Mtr.26358.1.S1_at	Hypothetical protein	-3.48
Mtr.20815.1.S1_at	Hypothetical protein	-3.48
Mtr.31577.1.S1_at	Imidazole-4-carboxamide isomerase	-3.48
Mtr.29219.1.S1_at	Alcohol oxidase	-3.48
Mtr.39786.1.S1_at	Beta-carotene hydroxylase	-3.48
Mtr.40922.1.S1_at	Hypothetical protein	-3.48
Mtr.38947.1.S1_s_at	Hypothetical protein	-3.48
Mtr.41644.1.S1_at	Hypothetical protein	-3.48
Mtr.7486.1.S1_s_at	Zinc finger protein	-3.47
Mtr.41014.1.S1_at	Hypothetical protein	-3.47
Mtr.13136.1.S1_at	Fasciclin-like AGP 10	-3.47
Mtr.12218.1.S1_at	Cytochrome B6-F complex iron-sulfur subunit	-3.47
Mtr.25643.1.S1_at	B-type cyclin	-3.47
Mtr.45352.1.S1_s_at	Glutathione transporter	-3.47

Mtr.42446.1.S1_at	Nitrate reductase	-3.47
Mtr.43423.1.S1_at	Hypothetical protein	-3.47
Msa.2836.1.S1_at	Hypothetical protein	-3.47
Mtr.5291.1.S1_at	Hypothetical protein	-3.47
Mtr.10483.1.S1_at	Esterase D	-3.47
Mtr.8265.1.S1_at	Pectinacetylesterase-like protein	-3.47
Mtr.37908.1.S1_at	Peroxidase	-3.47
Mtr.16086.1.S1_at	Hypothetical protein	-3.47
Mtr.39543.1.S1_at	DNAJ heat shock N-terminal domain-containing protein	-3.46
Msa.926.1.S1_at	Hypothetical protein	-3.46
Mtr.33025.1.S1_s_at	Salt-inducible protein	-3.46
Mtr.28642.1.S1_at	Hypothetical protein	-3.46
Mtr.5862.1.S1_at	Photosystem II 22 kDa protein	-3.46
Mtr.45513.1.S1_at	Hypothetical protein	-3.46
Mtr.13209.1.S1_at	Hypothetical protein	-3.46
Mtr.26048.1.S1_at	Hypothetical protein	-3.46
Mtr.31119.1.S1_at	MRP-like ABC transporter	-3.46
Mtr.23330.1.S1_at	Mitochondrial carrier protein	-3.46
Mtr.29047.1.S1_at	Hypothetical protein	-3.45
Mtr.831.1.S1_at	Hypothetical protein	-3.45
Mtr.27418.1.S1_at	Hypothetical protein	-3.45
Mtr.27110.1.S1_at	C2 domain-containing protein	-3.45
Mtr.31536.1.S1_at	Hypothetical protein	-3.45
Mtr.29088.1.S1_at	Hypothetical protein	-3.45
Mtr.13378.1.S1_at	Zinc finger protein 4	-3.45
Mtr.6912.1.S1_at	Hypothetical protein	-3.45
Mtr.45252.1.S1_s_at	Calcium-binding protein CAST	-3.44
Mtr.8870.1.S1_at	Cytochrome P450 monooxygenase	-3.44
Mtr.2599.1.S1_at	Hypothetical protein	-3.44
Mtr.43402.1.S1_at	Ankyrin-like protein	-3.44
Mtr.19917.1.S1_at	Auxin responsive SAUR	-3.44
Mtr.38020.1.S1_at	Monodehydroascorbate reductase	-3.44
Mtr.8839.1.S1_at	Gamma-tocopherol methyltransferase	-3.44
Mtr.13551.1.S1_at	mRNA cleavage factor subunit	-3.44
Mtr.27901.1.S1_at	Hypothetical protein	-3.44
Mtr.13256.1.S1_at	Protein phosphatase-2C	-3.44
Mtr.7496.1.S1_at	Hypothetical protein	-3.43
Mtr.16049.1.S1_at	Hypothetical protein	-3.43
Mtr.32484.1.S1_at	Hypothetical protein	-3.43
Mtr.41768.1.S1_at	Verticillium wilt disease resistance protein	-3.43
Mtr.44814.1.S1_at	Ycf49-like protein	-3.42
Mtr.44526.1.S1_at	Hypothetical protein	-3.42
Mtr.9363.1.S1_s_at	Diphosphonucleotide phosphatase	-3.42
Mtr.24519.1.S1_at	Globulin-like protein	-3.42
Mtr.36341.1.S1_s_at	Tetracycline transporter protein	-3.42
Mtr.15511.1.S1_at	Hypothetical protein	-3.42
Mtr.15646.1.S1_at	Hypothetical protein	-3.42
Mtr.28982.1.S1_at	Hypothetical protein	-3.42
Msa.1025.1.S1_at	Hypothetical protein	-3.42
Mtr.2226.1.S1_at	Hypothetical protein	-3.42
Mtr.44853.1.S1_at	Constitutively photomorphogenic 1 protein	-3.41
Mtr.39493.1.S1_at	Squamosa promoter binding protein	-3.41
Mtr.37597.1.S1_s_at	Hypothetical protein	-3.41
Mtr.22801.1.S1_at	Polynucleotide phosphorylase	-3.41

Mtr.7841.1.S1_at	Hypothetical protein	-3.41
Mtr.27040.1.S1_s_at	Hypothetical protein	-3.41
Mtr.41982.1.S1_at	Sulfate transporter ATST1	-3.41
Mtr.18250.1.S1_at	Nuclear protein SET	-3.41
Mtr.10608.1.S1_at	Glutamine cyclotransferase	-3.41
Mtr.43427.1.S1_at	Hypothetical protein	-3.41
Mtr.52183.1.S1_at	Concanavalin A-like lectin	-3.41
Mtr.34788.1.S1_at	Glyceraldehyde-3-phosphate dehydrogenase	-3.40
Mtr.40458.1.S1_at	Hypothetical protein	-3.40
Mtr.12262.1.S1_at	Extensin	-3.40
Mtr.39936.1.S1_at	Spermidine synthase 2	-3.40
Mtr.13189.1.S1_at	Hypothetical protein	-3.40
Msa.1600.1.S1_at	Hypothetical protein	-3.40
Mtr.13662.1.S1_s_at	Protein kinase	-3.40
Mtr.50467.1.S1_at	Serine/threonine protein kinase	-3.40
Mtr.43891.1.S1_at	Hypothetical protein	-3.40
Mtr.27411.1.S1_s_at	Ribonucleoprotein	-3.40
Mtr.38333.1.S1_at	Hypothetical protein	-3.40
Msa.3107.1.S1_at	Hypothetical protein	-3.39
Mtr.49166.1.S1_at	Short-chain dehydrogenase/reductase	-3.39
Mtr.20335.1.S1_at	Pectin acetylesterase	-3.39
Mtr.25320.1.S1_at	Hypothetical protein	-3.39
Mtr.38913.1.S1_at	Hypothetical protein	-3.39
Mtr.32078.1.S1_at	Hypothetical protein	-3.39
Mtr.8418.1.S1_at	Xylulose kinase	-3.39
Mtr.38132.1.S1_a_at	Homeobox 2 protein	-3.39
Mtr.20398.1.S1_at	Hypothetical protein	-3.38
Mtr.18268.1.S1_s_at	Serine/threonine protein kinase	-3.38
Mtr.19083.1.S1_at	ABC transporter	-3.38
Mtr.24082.1.S1_at	Haloacid dehalogenase-like hydrolase	-3.38
Mtr.11510.1.S1_at	Hypothetical protein	-3.38
Mtr.20332.1.S1_at	Pectin acetylesterase	-3.38
Mtr.33744.1.S1_at	Hypothetical protein	-3.38
Mtr.37742.1.S1_s_at	Methionine sulfoxide reductase A	-3.38
Mtr.44708.1.S1_at	Hypothetical protein	-3.38
Mtr.28673.1.S1_at	Hypothetical protein	-3.37
Mtr.42768.1.S1_at	Fasciclin-like arabinogalactan protein 1	-3.37
Mtr.2081.1.S1_at	Hypothetical protein	-3.37
Mtr.35648.1.S1_at	bHLH transcription factor	-3.37
Mtr.41025.1.S1_at	Hypothetical protein	-3.37
Mtr.46334.1.S1_at	Serine/threonine protein kinase	-3.37
Mtr.17396.1.S1_s_at	Cellulose synthase	-3.37
Mtr.41183.1.S1_at	Growth regulator-related protein	-3.36
Mtr.5178.1.S1_s_at	Hydroxyisourate hydrolase	-3.36
Mtr.32826.1.S1_at	Hypothetical protein	-3.36
Mtr.17339.1.S1_at	50s ribosomal protein l18	-3.36
Msa.2895.1.S1_at	Hypothetical protein	-3.36
Mtr.39076.1.S1_at	Hypothetical protein	-3.36
Mtr.36865.1.S1_s_at	Hypothetical protein	-3.36
Mtr.52115.1.S1_s_at	Hypothetical protein	-3.35
Mtr.41659.1.S1_at	Chloroplast carotenoid epsilon-ring hydroxylase	-3.35
Mtr.20939.1.S1_at	Hypothetical protein	-3.35
Mtr.45091.1.S1_x_at	ABC transporter	-3.35
Mtr.41480.1.S1_at	Polygalacturonase isoenzyme 1 beta subunit	-3.35

Mtr.9768.1.S1_at	Hypothetical protein	-3.35
Mtr.47251.1.S1_at	Hypothetical protein	-3.35
Mtr.9109.1.S1_at	Paa2 P-type ATPase	-3.34
Mtr.16766.1.S1_at	Hypothetical protein	-3.34
Mtr.9284.1.S1_at	Hypothetical protein	-3.34
Mtr.8249.1.S1_at	Hypothetical protein	-3.34
Mtr.5545.1.S1_at	Hypothetical protein	-3.34
Mtr.46514.1.S1_s_at	Alpha/beta-amyrin synthase	-3.34
Mtr.22379.1.S1_s_at	Hypothetical protein	-3.34
Mtr.26297.1.S1_at	Hypothetical protein	-3.34
Mtr.20499.1.S1_at	Phosphatidylinositol-4-phosphate 5-kinase	-3.34
Mtr.29161.1.S1_at	Serine/threonine protein kinase	-3.34
Mtr.8916.1.S1_at	Farnesylated protein ATFP6	-3.34
Mtr.44868.1.S1_at	UPF0187 protein	-3.34
Mtr.14636.1.S1_s_at	RNA polymerase beta subunit	-3.34
Mtr.18807.1.S1_at	Flavoprotein monooxygenase	-3.34
Mtr.29418.1.S1_at	Hypothetical protein	-3.33
Mtr.10265.1.S1_at	Hydrolase-like protein	-3.33
Mtr.9521.1.S1_at	Hypothetical protein	-3.33
Mtr.40864.1.S1_at	Acyl-peptide hydrolase	-3.33
Mtr.32688.1.S1_at	Hypothetical protein	-3.33
Mtr.13826.1.S1_at	Hypothetical protein	-3.33
Mtr.50411.1.S1_at	DNA glycosylase	-3.32
Mtr.42285.1.S1_at	Phosphoglycerate mutase	-3.32
Mtr.21612.1.S1_at	Hypothetical protein	-3.32
Mtr.4733.1.S1_s_at	Peroxidase	-3.32
Mtr.48816.1.S1_at	Phosphoglycerate mutase	-3.32
Mtr.6710.1.S1_at	MYB transcription factor	-3.32
Mtr.28728.1.S1_at	Hypothetical protein	-3.32
Mtr.34205.1.S1_at	Hypothetical protein	-3.32
Mtr.24581.1.S1_s_at	1,2-diacylglycerol 3-beta-galactosyltransferase	-3.31
Mtr.36800.1.S1_at	Elicitor-inducible LRR receptor-like protein EILP	-3.31
Mtr.9816.1.S1_at	Hypothetical protein	-3.31
Mtr.49018.1.S1_at	30S ribosomal protein S31	-3.31
Mtr.38346.1.S1_at	Hypothetical protein	-3.31
Mtr.12206.1.S1_at	Ribulose 1,7-bisphosphate carboxylase small subunit	-3.31
Mtr.38925.1.S1_s_at	Hypothetical protein	-3.31
Mtr.45380.1.S1_at	Hypothetical protein	-3.31
Mtr.41803.1.S1_at	Hypothetical protein	-3.31
Mtr.39136.1.S1_at	Hypothetical protein	-3.31
Mtr.15660.1.S1_at	Peptidase M	-3.31
Mtr.5617.1.S1_at	Hypothetical protein	-3.31
Mtr.11306.1.S1_at	ATP-dependent RNA helicase	-3.31
Mtr.46678.1.S1_at	Metalloprotease	-3.30
Mtr.6340.1.S1_at	Cyclic nucleotide-gated ion channel 2	-3.30
Mtr.50484.1.S1_s_at	Aminopeptidase	-3.30
Mtr.33344.1.S1_at	Disease resistance protein	-3.30
Mtr.44447.1.S1_at	Hypothetical protein	-3.30
Mtr.23748.1.S1_s_at	C3HC4-type zinc finger protein	-3.30
Mtr.37988.1.S1_at	Hypothetical protein	-3.30
Mtr.36764.1.S1_at	Photosystem I psaH protein	-3.30
Mtr.26951.1.S1_at	Hypothetical protein	-3.30
Mtr.13927.1.S1_at	Hypothetical protein	-3.30
Mtr.13163.1.S1_at	Hypothetical protein	-3.30

Mtr.40495.1.S1_at	Beta-mannosidase	-3.30
Mtr.21822.1.S1_at	Hypothetical protein	-3.29
Mtr.45146.1.S1_at	Apolipoprotein D	-3.29
Mtr.6831.1.S1_at	UDP-glucose glucosyltransferase	-3.29
Mtr.15888.1.S1_at	Serine/threonine protein kinase	-3.29
Mtr.8503.1.S1_at	Photosystem II reaction center W protein	-3.29
Msa.1784.1.S1_at	Hypothetical protein	-3.29
Mtr.21944.1.S1_s_at	CYP83D1p	-3.29
Mtr.27607.1.S1_at	Hypothetical protein	-3.29
Mtr.4623.1.S1_at	WRKY transcription factor 32	-3.29
Mtr.8748.1.S1_at	Methionine synthase	-3.29
Mtr.11113.1.S1_at	Hypothetical protein	-3.29
Mtr.18240.1.S1_at	Auxin responsive SAUR protein	-3.28
Msa.1757.1.S1_at	Hypothetical protein	-3.28
Mtr.6734.1.S1_s_at	Hypothetical protein	-3.28
Mtr.50173.1.S1_at	Hypothetical protein	-3.28
Mtr.39618.1.S1_at	Prolyl tRNA synthetase	-3.28
Mtr.36351.1.S1_at	Cytochrome oxidase II	-3.28
Mtr.50819.1.S1_s_at	Hypothetical protein	-3.28
Mtr.40199.1.S1_at	Hypothetical protein	-3.28
Mtr.44644.1.S1_s_at	Hydrolase	-3.28
Mtr.18574.1.S1_at	3,4-Dihydroxy-2-butanone 4-phosphate synthase	-3.28
Mtr.38216.1.S1_at	Hypothetical protein	-3.28
Msa.897.1.S1_s_at	Hypothetical protein	-3.28
Mtr.2306.1.S1_at	Trehalose-6-phosphate phosphatase	-3.28
Mtr.6063.1.S1_at	Glucosyltransferase	-3.27
Mtr.48824.1.S1_at	tRNA isopentenyltransferase	-3.27
Mtr.11718.1.S1_at	Hypothetical protein	-3.27
Mtr.15765.1.S1_at	Hypothetical protein	-3.27
Mtr.10428.1.S1_at	NADP-specific isocitrate dehydrogenase	-3.27
Mtr.23750.1.S1_at	Hypothetical protein	-3.27
Mtr.10009.1.S1_at	Hypothetical protein	-3.27
Mtr.35064.1.S1_at	Cucumisin-like serine protease	-3.27
Mtr.8663.1.S1_at	Calcium/calmodulin-dependent protein kinase CaMK3	-3.26
Mtr.27917.1.S1_s_at	Yippee-like protein	-3.26
Mtr.8330.1.S1_at	Hypothetical protein	-3.26
Mtr.25726.1.S1_s_at	Hypothetical protein	-3.26
Mtr.40716.1.S1_at	WAP four-disulfide core domain protein 1	-3.26
Mtr.5818.1.S1_at	Myosin heavy chain	-3.25
Mtr.9004.1.S1_at	Hypothetical protein	-3.25
Mtr.11205.1.S1_at	Hypothetical protein	-3.25
Mtr.39292.1.S1_at	Hypothetical protein	-3.25
Mtr.28150.1.S1_at	Recombination protein	-3.24
Mtr.41696.1.S1_at	Hypothetical protein	-3.24
Mtr.6192.1.S1_at	Hypothetical protein	-3.24
Msa.2547.1.S1_at	Hypothetical protein	-3.23
Mtr.25499.1.S1_at	NifU-related metallocluster assembly factor	-3.23
Mtr.15297.1.S1_at	Hypothetical protein	-3.23
Mtr.35461.1.S1_at	Hypothetical protein	-3.23
Mtr.12327.1.S1_at	Late embryogenesis abundant protein	-3.23
Mtr.6371.1.S1_s_at	GDSL-motif lipase/hydrolase-like protein	-3.23
Mtr.37336.1.S1_at	RNA-binding protein 2	-3.23
Mtr.13351.1.S1_at	ABC transporter	-3.23
Mtr.33858.1.S1_at	Metal-transporting P-type ATPase	-3.23

Mtr.9332.1.S1_at	Hypothetical protein	-3.23
Mtr.43439.1.S1_at	Hypothetical protein	-3.23
Mtr.51065.1.S1_at	Cytochrome P450	-3.22
Mtr.35998.1.S1_at	Cinnamoyl-CoA reductase	-3.22
Msa.1840.1.S1_at	Hypothetical protein	-3.22
Mtr.618.1.S1_s_at	Hypothetical protein	-3.22
Mtr.15378.1.S1_at	Hypothetical protein	-3.22
Mtr.28621.1.S1_at	Hypothetical protein	-3.22
Mtr.12179.1.S1_at	Hypothetical protein	-3.22
Mtr.51607.1.S1_at	Beta-Ig-H3/fasciclin	-3.22
Msa.1262.1.S1_at	Hypothetical protein	-3.22
Mtr.5617.1.S1_s_at	Hypothetical protein	-3.22
Mtr.42030.1.S1_at	Hypothetical protein	-3.21
Mtr.12953.1.S1_at	Hydroperoxide lyase	-3.21
Mtr.10790.1.S1_at	Lysosomal Pro-X carboxypeptidase	-3.21
Msa.2936.1.S1_at	Hypothetical protein	-3.21
Mtr.46674.1.S1_at	Hypothetical protein	-3.21
Mtr.11230.1.S1_at	Laccase (Diphenol oxidase)	-3.21
Mtr.34132.1.S1_s_at	Hypothetical protein	-3.21
Mtr.29554.1.S1_s_at	Hypothetical protein	-3.21
Mtr.42619.1.S1_at	Hypothetical protein	-3.21
Mtr.16219.1.S1_s_at	Zn-finger, B-box	-3.21
Mtr.50233.1.S1_at	Initiation factor 3	-3.21
Msa.1448.1.S1_at	Hypothetical protein	-3.21
Mtr.28077.1.S1_at	Hypothetical protein	-3.20
Mtr.17048.1.S1_at	Metallo-dependent hydrolase	-3.20
Msa.1148.1.S1_at	Hypothetical protein	-3.20
Mtr.36413.1.S1_at	Hypothetical protein	-3.20
Mtr.41476.1.S1_at	Cytochrome P450 71D11	-3.20
Mtr.41933.1.S1_at	Hypothetical protein	-3.20
Mtr.44149.1.S1_at	Aquaporin	-3.20
Mtr.24954.1.S1_s_at	Protein kinase	-3.20
Mtr.5133.1.S1_at	Hypothetical protein	-3.19
Mtr.41953.1.S1_at	Hypothetical protein	-3.19
Mtr.40105.1.S1_at	Hypothetical protein	-3.19
Mtr.41929.1.S1_at	Phenylalanine-tRNA synthetase	-3.19
Mtr.12473.1.S1_at	Isoflavonoid glucosyltransferase	-3.19
Mtr.24881.1.S1_at	Hypothetical protein	-3.19
Mtr.33445.1.S1_at	Plakoglobin/armadillo/beta-catenin-like protein	-3.19
Mtr.26216.1.S1_at	Auxin response factor 5	-3.19
Msa.2619.1.S1_at	Hypothetical protein	-3.19
Mtr.40237.1.S1_at	Hypothetical protein	-3.19
Mtr.34826.1.S1_at	Hypothetical protein	-3.19
Mtr.7486.1.S1_at	Zinc finger protein	-3.19
Mtr.38545.1.S1_at	Kinesin 1 (Kinesin-like protein A)	-3.18
Mtr.43725.1.S1_at	50S ribosomal protein L24	-3.18
Mtr.29445.1.S1_at	Hypothetical protein	-3.18
Mtr.40545.1.S1_at	HSP associated protein	-3.18
Mtr.38127.1.S1_at	Hypothetical protein	-3.18
Mtr.10170.1.S1_at	Hypothetical protein	-3.18
Msa.3012.1.S1_at	Hypothetical protein	-3.18
Mtr.29302.1.S1_at	Hypothetical protein	-3.18
Mtr.16385.1.S1_s_at	Hypothetical protein	-3.17
Mtr.36189.1.S1_at	Hypothetical protein	-3.17

Mtr.42744.1.S1_at	Hypothetical protein	-3.17
Mtr.12714.1.S1_at	Hypothetical protein	-3.17
Mtr.51453.1.S1_at	Hypothetical protein	-3.17
Mtr.41113.1.S1_at	Lectin-like receptor kinase	-3.17
Mtr.15800.1.S1_s_at	Zn-binding protein	-3.17
Mtr.48940.1.S1_at	Protein phosphatase 2C	-3.17
Mtr.6056.1.S1_at	Serine protease	-3.17
Mtr.41264.1.S1_at	Zinc finger protein	-3.17
Mtr.12701.1.S1_at	Om(1E) protein	-3.17
Mtr.48366.1.S1_at	Hypothetical protein	-3.17
Mtr.15000.1.S1_at	Transferase	-3.17
Mtr.15932.1.S1_at	Hypothetical protein	-3.17
Mtr.43458.1.S1_s_at	Hypothetical protein	-3.16
Mtr.30783.1.S1_at	Cytochrome P450	-3.16
Mtr.33076.1.S1_at	Actin binding protein	-3.16
Msa.1681.1.S1_at	Hypothetical protein	-3.16
Mtr.41315.1.S1_at	Mechanosensitive ion channel domain-containing protein	-3.16
Mtr.11805.1.S1_s_at	Hypothetical protein	-3.16
Msa.2543.1.S1_at	Hypothetical protein	-3.16
Mtr.38498.1.S1_at	Mitochondrial transcription termination factor	-3.16
Mtr.32433.1.S1_at	Aminotransferase ACS10	-3.16
Mtr.40894.1.S1_at	SF16 protein	-3.15
Mtr.25541.1.S1_at	Hypothetical protein	-3.15
Mtr.48305.1.S1_x_at	Hypothetical protein	-3.15
Mtr.13460.1.S1_at	Chloroplast ribosomal protein L10	-3.15
Mtr.6127.1.S1_s_at	ADP-ribosylation factor	-3.15
Mtr.41083.1.S1_at	Hypothetical protein	-3.15
Mtr.41620.1.S1_at	Hypothetical protein	-3.15
Mtr.43742.1.S1_at	bZIP protein BZO2H2	-3.15
Mtr.41201.1.S1_at	50S ribosomal protein L28	-3.15
Mtr.50620.1.S1_at	16S rRNA processing protein RimM	-3.15
Mtr.8568.1.S1_at	Nitrite reductase	-3.14
Msa.1329.1.S1_at	Hypothetical protein	-3.14
Mtr.8500.1.S1_at	Hypothetical protein	-3.14
Mtr.41658.1.S1_at	Glucose transporter 8	-3.14
Mtr.769.1.S1_at	Harpin-induced protein 1 family (HIN1)	-3.14
Mtr.16583.1.S1_at	Hypothetical protein	-3.14
Mtr.40827.1.S1_at	Hypothetical protein	-3.14
Mtr.443.1.S1_at	Resistance protein candidate RGC2K	-3.13
Mtr.11430.1.S1_s_at	Extensin-like protein	-3.13
Mtr.44328.1.S1_at	Hypothetical protein	-3.13
Mtr.39436.1.S1_at	RelA-SpoT like protein	-3.13
Mtr.37970.1.S1_at	Cis,cis-muconate cycloisomerase	-3.13
Mtr.33677.1.S1_s_at	Diphosphonucleotide phosphatase	-3.13
Mtr.45471.1.S1_at	Hypothetical protein	-3.13
Mtr.12270.1.S1_at	Alternative oxidase	-3.13
Mtr.9623.1.S1_at	Hypothetical protein	-3.13
Mtr.28227.1.S1_at	Hypothetical protein	-3.13
Mtr.49033.1.S1_s_at	Serine/threonine protein kinase	-3.13
Mtr.29262.1.S1_at	3-hydroxy-3-methylglutaryl-coenzyme A reductase 2	-3.13
Mtr.51747.1.S1_at	Hypothetical protein	-3.13
Mtr.43643.1.S1_at	Sucrose-phosphatase	-3.12
Mtr.22822.1.S1_at	Hypothetical protein	-3.12
Mtr.37929.1.S1_at	Inositol phosphatase	-3.12

Mtr.12588.1.S1_at	Hypothetical protein	-3.12
Mtr.32155.1.S1_at	Hypothetical protein	-3.12
Mtr.39594.1.S1_at	Trehalose-6-phosphate phosphatase	-3.12
Mtr.37876.1.S1_at	Protein disulfide isomerase	-3.12
Mtr.11399.1.S1_at	Proline-rich protein	-3.12
Mtr.32558.1.S1_at	N-acylethanolamine amidohydrolase	-3.12
Mtr.43812.1.S1_at	Hypothetical protein	-3.12
Mtr.15226.1.S1_at	bZIP transcription factor	-3.11
Mtr.8392.1.S1_at	4-alpha-glucanotransferase	-3.11
Mtr.4206.1.S1_at	Hypothetical protein	-3.11
Mtr.38029.1.S1_at	Chloroplast 50S ribosomal protein L31	-3.11
Mtr.32171.1.S1_x_at	Aquaporin protein	-3.11
Mtr.11558.1.S1_at	SERK1 protein	-3.11
Mtr.48326.1.S1_at	60S ribosomal protein L13A	-3.11
Mtr.39009.1.S1_at	Serine-rich protein	-3.11
Mtr.11023.1.S1_at	Ethylene-responsive family protein	-3.11
Mtr.42951.1.S1_at	Hypothetical protein	-3.11
Msa.1635.1.S1_at	Hypothetical protein	-3.11
Mtr.39517.1.S1_at	Hypothetical protein	-3.11
Mtr.13613.1.S1_s_at	Actin-related protein 8B	-3.10
Mtr.29307.1.S1_at	Multi resistance protein	-3.10
Mtr.29108.1.S1_at	Hypothetical protein	-3.10
Mtr.5084.1.S1_at	Hypothetical protein	-3.10
Mtr.5904.1.S1_at	Salt-inducible protein	-3.10
Mtr.10265.1.S1_s_at	Hydrolase-like protein	-3.10
Mtr.11081.1.S1_at	Hypothetical protein	-3.10
Msa.1684.1.S1_at	Hypothetical protein	-3.10
Mtr.13670.1.S1_at	Hypothetical protein	-3.10
Mtr.39924.1.S1_at	Ethylene-responsive family protein	-3.10
Mtr.13733.1.S1_at	Hypothetical protein	-3.10
Mtr.41817.1.S1_at	Sulfate transporter ATST1	-3.09
Mtr.14184.1.S1_s_at	Annexin	-3.09
Mtr.44423.1.S1_at	Laccase	-3.09
Mtr.16346.1.S1_at	Prefoldin	-3.09
Mtr.47312.1.S1_at	Hydrolase	-3.09
Mtr.51178.1.S1_at	Hypothetical protein	-3.09
Mtr.44052.1.S1_at	Cryptochrome 1 apoprotein	-3.09
Mtr.29352.1.S1_at	Peptide transporter	-3.09
Mtr.47027.1.S1_at	Ubiquitin-conjugating enzyme	-3.09
Mtr.15800.1.S1_at	Zn-binding protein	-3.09
Mtr.8825.1.S1_at	Hypothetical protein	-3.09
Mtr.25491.1.S1_at	Hypothetical protein	-3.09
Mtr.42024.1.S1_at	Hypothetical protein	-3.09
Mtr.33841.1.S1_at	Hypothetical protein	-3.09
Msa.1269.1.S1_at	Hypothetical protein	-3.09
Mtr.7078.1.S1_s_at	Peroxidase	-3.09
Mtr.927.1.S1_at	Purple acid phosphatase	-3.09
Mtr.5295.1.S1_at	GA protein	-3.09
Mtr.28065.1.S1_at	Aromatic rich glycoprotein	-3.09
Mtr.41209.1.S1_at	Hypothetical protein	-3.08
Mtr.40865.1.S1_at	Acyl-peptide hydrolase	-3.08
Mtr.2293.1.S1_at	Hypothetical protein	-3.08
Mtr.8973.1.S1_at	Hypothetical protein	-3.08
Mtr.12933.1.S1_at	Chloroplast Release Factor 1	-3.08

Mtr.41769.1.S1_at	Hypothetical protein	-3.08
Mtr.39884.1.S1_at	Hypothetical protein	-3.08
Mtr.42487.1.S1_at	Alpha-mannosidase	-3.08
Mtr.40589.1.S1_at	Symbiosis-related protein	-3.08
Mtr.2580.1.S1_at	Leptin receptor	-3.08
Mtr.11062.1.S1_at	Hypothetical protein	-3.08
Mtr.13218.1.S1_at	Hypothetical protein	-3.08
Mtr.26285.1.S1_s_at	Hypothetical protein	-3.07
Mtr.9770.1.S1_at	Hypothetical protein	-3.07
Mtr.43462.1.S1_at	Hypothetical protein	-3.07
Mtr.45728.1.S1_s_at	Pentatricopeptide repeat protein	-3.07
Mtr.29496.1.S1_at	H+-ATPase	-3.07
Mtr.5522.1.S1_at	Urease	-3.07
Mtr.35486.1.S1_at	Hypothetical protein	-3.07
Mtr.28186.1.S1_at	Hypothetical protein	-3.07
Mtr.40545.1.S1_a_at	HSP associated protein	-3.07
Mtr.28002.1.S1_at	Hypothetical protein	-3.07
Mtr.23279.1.S1_at	Geraniol synthase	-3.07
Mtr.12200.1.S1_at	Hypothetical protein	-3.07
Mtr.9045.1.S1_at	Carbonate dehydratase	-3.07
Mtr.16430.1.S1_at	Hypothetical protein	-3.07
Mtr.20400.1.S1_at	Hypothetical protein	-3.06
Mtr.39931.1.S1_at	Hypothetical protein	-3.06
Mtr.43856.1.S1_at	Protein kinase	-3.06
Mtr.13247.1.S1_at	Hypothetical protein	-3.06
Mtr.40674.1.S1_at	Ribosome-like protein	-3.06
Mtr.33689.1.S1_at	Hypothetical protein	-3.06
Msa.2419.1.S1_at	Hypothetical protein	-3.06
Mtr.9050.1.S1_at	Hypothetical protein	-3.06
Mtr.21755.1.S1_at	Hypothetical protein	-3.06
Mtr.10835.1.S1_s_at	Hypothetical protein	-3.06
Msa.2803.1.S1_at	Hypothetical protein	-3.06
Mtr.40816.1.S1_at	Thylakoid lumenal 15 kDa protein	-3.05
Mtr.13656.1.S1_at	Galactomannan galactosyltransferase	-3.05
Mtr.32980.1.S1_at	Hypothetical protein	-3.05
Mtr.29147.1.S1_at	Hypothetical protein	-3.05
Mtr.33737.1.S1_at	Hypothetical protein	-3.05
Mtr.33407.1.S1_s_at	Sophorol reductase	-3.05
Mtr.37847.1.S1_at	UDP-glycosyltransferase 85A8	-3.05
Mtr.42323.1.S1_at	Hypothetical protein	-3.05
Mtr.5346.1.S1_at	Receptor-like protein kinase	-3.05
Mtr.12366.1.S1_s_at	Transcription factor Myb1	-3.05
Mtr.11116.1.S1_at	Hypothetical protein	-3.05
Mtr.20619.1.S1_x_at	Auxin responsive SAUR	-3.05
Mtr.43210.1.S1_at	Hypothetical protein	-3.05
Mtr.28467.1.S1_s_at	Aspartyl aminopeptidase	-3.04
Mtr.50208.1.S1_at	Protein kinase	-3.04
Mtr.46498.1.S1_at	Hypothetical protein	-3.04
Mtr.5649.1.S1_at	Serine/threonine protein kinase	-3.04
Mtr.13516.1.S1_s_at	ZF-HD homeobox protein	-3.04
Mtr.30128.1.S1_at	Hypothetical protein	-3.04
Mtr.41688.1.S1_at	Hypothetical protein	-3.04
Mtr.28734.1.S1_at	Hypothetical protein	-3.03
Mtr.47245.1.S1_s_at	Hypothetical protein	-3.03

Mtr.43324.1.S1_s_at	Polygalacturonase-like protein	-3.03
Mtr.27813.1.S1_at	6-4 photolyase	-3.03
Mtr.11004.1.S1_s_at	Hydrolase-like protein	-3.03
Mtr.38379.1.S1_at	Egr1 protein	-3.03
Mtr.18798.1.S1_at	Proteinase inhibitor I3	-3.03
Mtr.26982.1.S1_at	Hypothetical protein	-3.03
Mtr.8454.1.S1_at	Hypothetical protein	-3.03
Mtr.32338.1.S1_at	CLC-e chloride channel protein	-3.03
Mtr.11040.1.S1_s_at	Nodulin-like protein	-3.03
Mtr.40343.1.S1_s_at	Dihydrofolate reductase-thymidylate synthase	-3.03
Msa.1945.1.S1_at	Hypothetical protein	-3.03
Mtr.47227.1.S1_s_at	RelA-SpoT like protein	-3.03
Msa.1756.1.S1_at	Hypothetical protein	-3.02
Mtr.3652.1.S1_at	Hypothetical protein	-3.02
Mtr.33547.1.S1_at	Cellulose synthase	-3.02
Mtr.37427.1.S1_at	Aquaporin TIP4.1 (Tonoplast intrinsic protein 4.1)	-3.02
Mtr.2022.1.S1_at	Receptor-like protein kinase	-3.02
Mtr.26160.1.S1_at	Hypothetical protein	-3.02
Mtr.26115.1.S1_at	Protein kinase	-3.02
Mtr.553.1.S1_at	Hypothetical protein	-3.02
Mtr.38269.1.S1_at	Arabinogalactan protein	-3.01
Mtr.39248.1.S1_at	Hypothetical protein	-3.01
Mtr.51652.1.S1_at	Cytochrome P450	-3.01
Mtr.15273.1.S1_at	Peptidase M14	-3.01
Mtr.44327.1.S1_at	Adenylate kinase 2	-3.01
Mtr.9426.1.S1_at	Hypothetical protein	-3.01
Mtr.14599.1.S1_at	Hypothetical protein	-3.01
Mtr.12706.1.S1_at	Hypothetical protein	-3.01
Mtr.37510.1.S1_s_at	Hypothetical protein	-3.01
Mtr.43099.1.S1_at	Transcription factor Myb1	-3.01
Mtr.12288.1.S1_at	2,4-D inducible glutathione S-transferase	-3.01
Mtr.40352.1.S1_at	SNF1 like protein kinase	-3.01
Mtr.34479.1.S1_at	Hypothetical protein	-3.01
Mtr.17706.1.S1_at	Hypothetical protein	-3.00
Mtr.43893.1.S1_at	Immunophilin-like protein	-3.00
Mtr.29388.1.S1_s_at	Hypothetical protein	-3.00
Mtr.40612.1.S1_at	Myo-inositol monophosphatase 2	-3.00
Mtr.40583.1.S1_at	Polygalacturonase-like protein	-3.00
Mtr.45321.1.S1_at	Diphosphonucleotide phosphatase 1	-3.00
Mtr.34769.1.S1_at	Hypothetical protein	-3.00

Supplementary Table S3. Upregulated genes at 24 hours after inoculation with soybean rust

Probesets	Target Description	Fold induction
Mtr.37395.1.S1_at	Deoxycytidine deaminase	1033.01
Mtr.43146.1.S1_at	Branched-chain-amino-acid aminotransferase 2	952.58
Mtr.8417.1.S1_at	Mitochondrial uncoupling protein	582.90
Mtr.18650.1.S1_at	Glycoside hydrolase	534.19
Mtr.15537.1.S1_at	Hypothetical protein	527.57
Mtr.22656.1.S1_s_at	Deoxycytidylate deaminase	517.66
Mtr.8498.1.S1_s_at	Asparagine synthetase	511.70
Mtr.19796.1.S1_at	Hypothetical protein	489.28
Mtr.18127.1.S1_at	Glyoxalase	462.67
Mtr.16278.1.S1_at	Proteinase inhibitor I12	459.19
Mtr.37375.1.S1_at	Hypothetical protein	454.82
Mtr.5486.1.S1_s_at	Hypothetical protein	338.47
Msa.1736.1.S1_at	Hypothetical protein	326.44
Mtr.43467.1.S1_at	Hypothetical protein	316.53
Mtr.40564.1.S1_at	Cyanogenic Beta-Glucosidase	311.69
Mtr.35334.1.S1_s_at	Hypothetical protein	298.89
Mtr.44900.1.S1_at	Hypothetical protein	298.87
Mtr.22592.1.S1_at	Cytosolic fructose-1,6-bisphosphatase	297.52
Mtr.8498.1.S1_at	Asparagine synthetase	270.05
Mtr.40120.1.S1_at	Cationic peroxidase 1	244.97
Mtr.44559.1.S1_at	Hypothetical protein	217.66
Mtr.34416.1.S1_at	Pyruvate,orthophosphate dikinase	216.42
Mtr.40147.1.S1_s_at	Class 10 PR protein	212.17
Mtr.13321.1.S1_at	Pyruvate, phosphate dikinase	210.61
Mtr.40553.1.S1_at	Hypothetical protein	207.64
Mtr.1538.1.S1_at	Hypothetical protein	206.45
Mtr.45497.1.S1_s_at	Geraniol synthase	203.73
Mtr.45667.1.S1_x_at	Type III polyketide synthase	203.63
Msa.1060.1.S1_at	Hypothetical protein	200.99
Mtr.42416.1.S1_at	Hypothetical protein	181.65
Mtr.21662.1.S1_at	Hypothetical protein	168.93
Mtr.43830.1.S1_at	Hypothetical protein	161.65
Mtr.43942.1.S1_s_at	Hypothetical protein	158.34
Mtr.39214.1.S1_at	Hypothetical protein	158.11
Mtr.11656.1.S1_at	Hypothetical protein	153.11
Mtr.10434.1.S1_at	SRG1 protein	151.47
Mtr.42989.1.S1_at	Thaumatin-like protein PR-5b	148.87
Mtr.20464.1.S1_x_at	Naringenin-chalcone synthase	147.54
Mtr.41238.1.S1_at	Hypothetical protein	147.29
Mtr.11055.1.S1_at	SRG1 protein	144.35
Mtr.42268.1.S1_at	Inositol oxygenase	135.99
Mtr.43008.1.S1_at	Hypothetical protein	134.21
Msa.1879.1.S1_s_at	Hypothetical protein	133.10
Mtr.331.1.S1_at	Chitinase	132.80
Mtr.23418.1.S1_s_at	AP2 domain transcription factor	130.97
Mtr.37358.1.S1_s_at	Cytochrome P450 71B13	130.60
Mtr.37393.1.S1_at	Attacin A	128.37
Mtr.34968.1.S1_at	Nucellin-like protein	127.13
Msa.2901.1.S1_s_at	Hypothetical protein	126.96
Mtr.17404.1.S1_s_at	Aldo/keto reductase	125.77
Mtr.15261.1.S1_s_at	Curculin-like (mannose-binding) lectin	125.70
Mtr.39844.1.S1_at	Hypothetical protein	121.56

Mtr.11267.1.S1_at	Protein kinase-like protein	120.16
Mtr.15678.1.S1_s_at	Aldo/keto reductase	119.18
Mtr.11104.1.S1_s_at	Hypothetical protein	118.17
Mtr.34114.1.S1_s_at	Pathogenesis related protein	116.08
Mtr.40563.1.S1_at	Cyanogenic Beta-Glucosidase	114.28
Mtr.37356.1.S1_at	Cytochrome P450 71B25	113.58
Mtr.17526.1.S1_at	Hypothetical protein	112.27
Mtr.43254.1.S1_at	Hypothetical protein	112.13
Mtr.23578.1.S1_s_at	Polygalacturonase-inhibiting protein	111.97
Mtr.33212.1.S1_s_at	Beta-glucosidase	110.12
Mtr.12748.1.S1_at	Hypothetical protein	108.16
Mtr.11851.1.S1_at	Hypothetical protein	105.43
Mtr.45373.1.S1_at	Hypothetical protein	102.11
Msa.2530.1.S1_s_at	Hypothetical protein	101.63
Mtr.12675.1.S1_at	Integral membrane protein	101.14
Mtr.4663.1.S1_at	Light-inducible protein	99.03
Mtr.10391.1.S1_at	Class 10 PR protein	98.15
Mtr.1299.1.S1_s_at	Cytochrome P450 93B1	96.31
Mtr.44354.1.S1_at	Cytochrome P450 monooxygenase	95.66
Mtr.43279.1.S1_at	Proline dehydrogenase	94.67
Mtr.33545.1.S1_s_at	Non-cyanogenic beta-glucosidase	93.91
Mtr.14224.1.S1_at	Proteinase inhibitor I13	93.34
Mtr.35755.1.S1_at	Hypothetical protein	92.21
Mtr.1402.1.S1_at	Hypothetical protein	91.89
Mtr.10573.1.S1_at	Hypothetical protein	91.83
Mtr.43196.1.S1_at	Hypothetical protein	91.44
Mtr.32248.1.S1_at	3-hydroxyisobutyrate dehydrogenase	91.32
Mtr.10502.1.S1_s_at	Glutathione S-transferase parA (Auxin-regulated protein parA)	89.64
Mtr.24901.1.S1_at	Phospholipase	88.82
Mtr.8831.1.S1_at	Patatin-like protein 1	88.71
Mtr.18521.1.S1_at	Thioredoxin-related protein	88.62
Mtr.10435.1.S1_s_at	SRG1 protein	88.57
Mtr.8599.1.S1_s_at	Caffeic acid 3-O-methyltransferase 1	88.33
Mtr.21790.1.S1_at	Hypothetical protein	87.54
Mtr.51826.1.S1_at	Hypothetical protein	87.50
Mtr.28311.1.S1_s_at	Hypothetical protein	85.01
Mtr.44104.1.S1_at	Zeatin O-glucosyltransferase	84.82
Mtr.46391.1.S1_at	Hypothetical protein	83.46
Mtr.2105.1.S1_at	Hypothetical protein	82.01
Mtr.410.1.S1_s_at	Isoflavone reductase	81.83
Mtr.16207.1.S1_at	Hypothetical protein	81.19
Mtr.28242.1.S1_at	Beta-glucan-elicitor receptor	79.70
Mtr.38035.1.S1_at	S-locus-like receptor protein kinase	78.05
Mtr.28801.1.S1_at	Caffeoyl-CoA O-methyltransferase	78.03
Mtr.41059.1.S1_at	Hypothetical protein	76.55
Mtr.34158.1.S1_s_at	Hypothetical protein	75.50
Mtr.43594.1.S1_at	Hypothetical protein	74.96
Mtr.23863.1.S1_at	Glucan 1,3-beta-glucosidase	72.29
Mtr.15010.1.S1_s_at	Myb DNA-binding protein	71.05
Mtr.35325.1.S1_at	Hypothetical protein	69.73
Mtr.13317.1.S1_at	Hypothetical protein	69.47
Mtr.42129.1.S1_at	AP2 domain transcription factor	69.26
Mtr.40555.1.S1_at	Osmotin-like protein	68.96
Mtr.40399.1.S1_at	CPRD2 protein	68.15

Mtr.8599.1.S1_at	Caffeic acid 3-O-methyltransferase 1	68.09
Mtr.48534.1.S1_at	Hypothetical protein	66.32
Mtr.40377.1.S1_at	Cysteine proteinase inhibitor	65.99
Mtr.43852.1.S1_at	Acidic glucanase	65.97
Mtr.12246.1.S1_at	MtN12 protein	65.01
Mtr.40412.1.S1_at	Hypothetical protein	63.95
Mtr.41670.1.S1_at	F-box protein	63.94
Mtr.34634.1.S1_at	Epoxide hydrolase	62.72
Mtr.41053.1.S1_at	AT-hook DNA-binding protein	62.06
Mtr.15831.1.S1_s_at	Hypothetical protein	61.58
Mtr.22423.1.S1_at	Hin1 like protein	60.27
Mtr.37973.1.S1_at	Hypothetical protein	59.44
Mtr.42966.1.S1_at	Disease resistance response protein Pi49 (PR10)	59.42
Mtr.6667.1.S1_at	Cytochrome P450 monooxygenase	59.09
Mtr.37275.1.S1_at	Pathogen-inducible alpha-dioxygenase	57.81
Mtr.12616.1.S1_at	Cytochrome P450-like protein	57.78
Mtr.38234.1.S1_at	Epoxide hydrolase	56.95
Mtr.29026.1.S1_at	Hypothetical protein	55.07
Msa.2530.1.S1_at	Hypothetical protein	54.86
Mtr.40733.1.S1_at	Hypothetical protein	54.77
Mtr.17616.1.S1_x_at	Naringenin-chalcone synthase	54.17
Mtr.12193.1.S1_at	RCa12	53.65
Mtr.8507.1.S1_at	Pathogenesis related protein	53.57
Mtr.8763.1.S1_at	Thaumatin-like protein PR-5a	53.55
Mtr.28274.1.S1_at	Avr9/Cf-9 rapidly elicited protein	53.12
Mtr.37718.1.S1_at	Hypothetical protein	53.03
Mtr.17621.1.S1_x_at	Naringenin-chalcone synthase	52.63
Mtr.12502.1.S1_at	Laccase	52.01
Mtr.37984.1.S1_at	Adenosine nucleotide translocator	51.59
Mtr.51082.1.S1_at	Plant lipid transfer/seed storage/trypsin-alpha amylase inhibitor	51.50
Mtr.25838.1.S1_at	Hypothetical protein	51.27
Mtr.45666.1.S1_at	Glycoside hydrolase	49.95
Mtr.48647.1.S1_s_at	Hypothetical protein	49.92
Msa.1231.1.S1_at	Hypothetical protein	48.32
Mtr.17612.1.S1_x_at	Naringenin-chalcone synthase	47.98
Mtr.30508.1.S1_at	Isoflavone reductase	47.97
Mtr.44384.1.S1_at	PDR7 ABC transporter	47.80
Mtr.10692.1.S1_at	Cytochrome P450 76C4	47.57
Mtr.5486.1.S1_at	Hypothetical protein	47.16
Mtr.12485.1.S1_at	Hypothetical protein	46.81
Mtr.43363.1.S1_at	Syringolide-induced protein 13-1-1	46.81
Mtr.42255.1.S1_at	Hypothetical protein	46.78
Mtr.27937.1.S1_at	Nodulin-like protein	46.59
Mtr.11725.1.S1_at	Isoflavone synthase	46.27
Mtr.1493.1.S1_at	Hypothetical protein	46.13
Mtr.25575.1.S1_x_at	Hypothetical protein	45.89
Mtr.38241.1.S1_at	Hypothetical protein	45.86
Mtr.48996.1.S1_at	3Fe-4S ferredoxin	45.47
Mtr.49999.1.S1_at	Phloem-specific lectin PP2-like protein	44.77
Mtr.44487.1.S1_at	Hypothetical protein	44.53
Msa.2942.1.S1_s_at	Hypothetical protein	44.19
Mtr.38764.1.S1_at	Cytochrome P450	43.75
Mtr.40363.1.S1_at	Fiber protein Fb19	43.70
Mtr.10760.1.S1_at	N-hydroxycinnamoyl/benzoyltransferase-like protein	42.83

Mtr.46788.1.S1_at	L-allo-threonine aldolase	42.31
Mtr.5060.1.S1_at	Cyanogenic Beta-Glucosidase	42.30
Mtr.41478.1.S1_at	Class 10 PR protein	42.29
Mtr.10466.1.S1_at	Hypothetical protein	42.14
Mtr.49411.1.S1_at	Hypothetical protein	41.85
Mtr.36013.1.S1_at	Cryptosporidium parvum external transcribed spacer	41.72
Mtr.689.1.S1_at	FAD-linked oxidoreductase	41.56
Mtr.17910.1.S1_at	Serine/threonine protein kinase	41.43
Mtr.12653.1.S1_at	Hypothetical protein	41.37
Mtr.44326.1.S1_at	Ephrin type-A receptor 5	41.37
Msa.2910.1.S1_at	Hypothetical protein	40.48
Mtr.6645.1.S1_at	Lysine and histidine specific transporter	40.29
Mtr.1156.1.S1_at	Rhicadhesin receptor (Germin-like protein)	40.00
Mtr.40613.1.S1_at	Hypothetical protein	39.75
Mtr.10127.1.S1_s_at	Phospholipase	39.36
Mtr.10689.1.S1_at	Translation factor EF-1 alpha	39.16
Mtr.48768.1.S1_at	Gibberellin regulated protein	38.94
Mtr.38043.1.S1_at	Glucosyltransferase-10	38.55
Mtr.10368.1.S1_at	Chalcone synthase 8	38.32
Mtr.25021.1.S1_at	Vestitone reductase	38.30
Mtr.8652.1.S1_at	6a-hydroxymaackiain methyltransferase	38.29
Mtr.43644.1.S1_at	Homeodomain transcription factor	37.85
Mtr.8558.1.S1_at	Hypothetical protein	37.81
Mtr.11959.1.S1_at	Serine/threonine protein phosphatase 2A	37.78
Mtr.13239.1.S1_at	Hypothetical protein	37.66
Mtr.38079.1.S1_at	Calcium/calmodulin protein kinase 1	37.60
Mtr.22459.1.S1_at	Hypothetical protein	37.53
Mtr.4441.1.S1_at	Hypothetical protein	37.46
Mtr.40763.1.S1_at	Hypothetical protein	37.42
Mtr.39322.1.S1_at	UDP-glucuronosyltransferase	37.29
Mtr.8666.1.S1_s_at	Hypothetical protein	37.18
Mtr.38957.1.S1_at	Hypothetical protein	36.84
Mtr.8725.1.S1_at	Hypothetical protein	36.83
Msa.3005.1.S1_at	Hypothetical protein	36.79
Mtr.25008.1.S1_x_at	Vestitone reductase	36.71
Mtr.38121.1.S1_at	Avr9/Cf-9 rapidly elicited protein	36.19
Mtr.12141.1.S1_at	Hypothetical protein	35.93
Mtr.43189.1.S1_at	Disease resistance response protein	35.92
Mtr.45394.1.S1_at	Synaptobrevin-like protein	35.84
Mtr.37033.1.S1_s_at	Metalloproteinase	35.70
Mtr.11773.1.S1_s_at	Hypothetical protein	35.49
Mtr.37316.1.S1_at	NAD(P)H dependent 6'-deoxychalcone synthase	35.17
Mtr.49123.1.S1_at	Major intrinsic protein	34.66
Mtr.44496.1.S1_at	AP2 domain-containing protein	34.36
Mtr.34762.1.S1_s_at	coII intron protein	34.14
Mtr.10319.1.S1_at	Pprg2 protein	33.92
Mtr.43259.1.S1_at	Hypothetical protein	33.63
Mtr.10311.1.S1_at	Pprg2 protein	33.39
Mtr.13532.1.S1_at	Cytochrome P450	33.39
Mtr.10272.1.S1_at	Calmodulin	33.32
Mtr.10898.1.S1_at	Hypothetical protein	32.97
Mtr.37579.1.S1_at	Branched chain alpha-keto acid dehydrogenase E2 subunit	32.67
Mtr.8667.1.S1_at	Hypothetical protein	32.58
Msa.2919.1.S1_at	Hypothetical protein	31.95

Mtr.43769.1.S1_at	Seed imbibition protein	31.63
Mtr.49341.1.S1_at	Peptidase, metallopeptidases	31.31
Mtr.10427.1.S1_at	Hypothetical protein	31.21
Mtr.43569.1.S1_at	Medicago nodulin N21-like protein	31.19
Mtr.49305.1.S1_at	Fatty acid elongase	31.12
Mtr.42451.1.S1_at	Bimodular protein	31.09
Mtr.5445.1.S1_at	Hypothetical protein	30.92
Mtr.8975.1.S1_at	Bifunctional lysine-ketoglutarate reductase/saccharopine dehydrogenase	30.70
Mtr.23266.1.S1_at	Protease inhibitor	30.61
Mtr.10941.1.S1_at	Hypothetical protein	30.58
Mtr.13960.1.S1_at	Flavonoid 3'-hydroxylase	30.19
Mtr.47295.1.S1_x_at	Polygalacturonase inhibitor protein	30.17
Mtr.18422.1.S1_at	Hypothetical protein	30.17
Mtr.20618.1.S1_s_at	Transferase	30.01
Mtr.41495.1.S1_at	Hypothetical protein	29.96
Mtr.47992.1.S1_at	Hypothetical protein	29.96
Mtr.43280.1.S1_x_at	Beta-Glucosidase	29.83
Mtr.51557.1.S1_at	Chitin-binding protein	29.77
Msa.2552.1.S1_at	Hypothetical protein	29.61
Mtr.10317.1.S1_at	Pprg2 protein	29.54
Mtr.18.1.S1_at	Hypothetical protein	29.52
Mtr.49557.1.S1_at	Cys-rich protein	29.30
Mtr.6543.1.S1_at	Hypothetical protein	29.29
Mtr.8442.1.S1_at	Hydroxyproline-rich glycoprotein	29.28
Mtr.42815.1.S1_at	N-hydroxycinnamoyl/benzoyltransferase-like protein	29.25
Mtr.51252.1.S1_s_at	Transferase	29.04
Mtr.31314.1.S1_at	NAM	28.97
Mtr.24415.1.S1_s_at	Alpha-amylase	28.95
Mtr.32790.1.S1_at	Drm3	28.93
Mtr.42263.1.S1_s_at	Hypothetical protein	28.86
Mtr.9846.1.S1_at	Hypothetical protein	28.38
Mtr.44391.1.S1_at	Cytochrome P450 82A2	28.28
Mtr.13782.1.S1_s_at	Hypothetical protein	28.15
Mtr.51693.1.S1_at	Hypothetical protein	28.07
Mtr.6639.1.S1_s_at	Hypothetical protein	28.01
Mtr.19594.1.S1_at	Cyclin-like F-box	27.87
Mtr.42540.1.S1_at	Elicitor-inducible cytochrome P450	27.69
Mtr.49688.1.S1_at	Hypothetical protein	27.66
Mtr.42916.1.S1_at	Hypothetical protein	27.60
Mtr.12772.1.S1_at	Mlo protein	27.45
Mtr.38513.1.S1_at	Syringolide-induced protein 14-1-1	27.44
Mtr.8425.1.S1_at	Hypothetical protein	27.43
Mtr.10438.1.S1_at	Kunitz proteinase inhibitor-1	27.37
Mtr.13536.1.S1_at	Bifunctional lysine-ketoglutarate reductase/saccharopine dehydrogenase	27.09
Mtr.7260.1.S1_at	Ethylene-induced esterase	27.08
Mtr.50682.1.S1_at	14-3-3 protein	26.96
Mtr.12569.1.S1_at	Hypothetical protein	26.89
Mtr.27711.1.S1_at	Hypothetical protein	26.85
Mtr.35677.1.S1_at	Zinc finger protein	26.65
Mtr.43513.1.S1_at	Syringolide-induced protein 19-1-5	26.47
Mtr.41944.1.S1_at	Hypothetical protein	26.22
Mtr.44504.1.S1_at	Beta-glucan-elicitor receptor	26.07
Mtr.34867.1.S1_s_at	Hypothetical protein	25.80
Mtr.6154.1.S1_s_at	Wall-associated kinase	25.61

Mtr.45211.1.S1_at	Phosphoenolpyruvate carboxykinase	25.53
Mtr.9372.1.S1_at	DnaJ protein	25.52
Mtr.32409.1.S1_at	Galactokinase	25.23
Msa.2861.1.S1_at	Hypothetical protein	25.23
Mtr.38270.1.S1_at	ABA-responsive protein	25.12
Mtr.32790.1.S1_s_at	Drm3	25.12
Mtr.24415.1.S1_at	Alpha-amylase	25.10
Mtr.34185.1.S1_at	Hypothetical protein	25.08
Mtr.6666.1.S1_at	Hypothetical protein	24.93
Mtr.43193.1.S1_at	Glutamate dehydrogenase 3	24.87
Mtr.38347.1.S1_at	Tripartite motif protein	24.57
Mtr.37585.1.S1_a_at	Extensin	24.53
Mtr.43514.1.S1_s_at	Syringolide-induced protein 19-1-5	24.34
Mtr.37715.1.S1_at	Hypothetical protein	24.28
Mtr.40481.1.S1_at	Isovaleryl-CoA Dehydrogenase	24.26
Mtr.26218.1.S1_s_at	Isovaleryl-CoA Dehydrogenase	24.19
Mtr.45003.1.S1_at	Non-cyanogenic beta-glucosidase	23.95
Mtr.9107.1.S1_at	Hypothetical protein	23.91
Mtr.13150.1.S1_at	Hypothetical protein	23.90
Mtr.22561.1.S1_at	Hypothetical protein	23.85
Mtr.5785.1.S1_s_at	PDR7 ABC transporter	23.83
Mtr.41268.1.S1_at	Serine/threonine-specific protein kinase APK2a	23.61
Mtr.34806.1.S1_s_at	MLO-like protein	23.51
Mtr.25953.1.S1_at	Cytidine deaminase	23.37
Mtr.27985.1.S1_at	Hypothetical protein	23.30
Mtr.11066.1.S1_at	AKIN gamma	23.19
Mtr.43284.1.S1_at	Alcohol dehydroge	23.19
Mtr.8751.1.S1_at	Hypothetical protein	23.17
Mtr.79.1.S1_at	Hypothetical protein	23.08
Mtr.5385.1.S1_at	AP2 domain transcription factor	22.86
Msa.2701.1.S1_at	Hypothetical protein	22.85
Mtr.25362.1.S1_s_at	Hypothetical protein	22.82
Mtr.14283.1.S1_at	Aminotransferase class-III	22.81
Mtr.12487.1.S1_at	Cyanogenic Beta-Glucosidase	22.78
Mtr.6339.1.S1_at	Ripening regulated protein	22.71
Mtr.46286.1.S1_at	Small GTP-binding protein	22.65
Mtr.16615.1.S1_at	Prephenate dehydratase	22.58
Mtr.17288.1.S1_at	Hypothetical protein	22.41
Mtr.12764.1.S1_at	AMP-binding protein	22.39
Mtr.38765.1.S1_at	LOB domain protein 38	22.39
Mtr.39424.1.S1_at	Serine/threonine protein kinase	22.36
Mtr.46130.1.S1_at	Synaptobrevin	22.29
Mtr.38997.1.S1_at	Serine/threonine protein kinase	22.28
Mtr.6950.1.S1_at	Galactokinase like protein	22.26
Mtr.31952.1.S1_at	Hypothetical protein	22.20
Mtr.8559.1.S1_at	Hypothetical protein	22.15
Mtr.33508.1.S1_s_at	Nodulin N21-like protein	22.09
Mtr.40504.1.S1_at	O-methyltransferase	22.04
Mtr.41772.1.S1_at	AMP-binding protein	22.03
Mtr.29505.1.S1_s_at	Hypothetical protein	22.00
Mtr.10895.1.S1_at	Hypothetical protein	21.99
Mtr.40339.1.S1_s_at	Hypothetical protein	21.98
Mtr.8696.1.S1_at	Zinc finger DNA-binding protein	21.74
Mtr.49090.1.S1_at	GCN5-related N-acetyltransferase	21.66

Mtr.43009.1.S1_s_at	Isoflavone 2'-hydroxylase	21.62
Mtr.37376.1.S1_s_at	Hypothetical protein	21.57
Mtr.6976.1.S1_at	Hypothetical protein	21.49
Mtr.39569.1.S1_at	Hypothetical protein	21.45
Msa.886.1.S1_s_at	Hypothetical protein	21.44
Mtr.11047.1.S1_at	Mitogen-activated protein kinase 4	21.35
Mtr.45398.1.S1_at	Exo-1,3-beta-glucanase	21.29
Mtr.11520.1.S1_at	Protein phosphatase 2C	21.25
Mtr.40340.1.S1_at	Alkaline alpha galactosidase I	21.23
Mtr.4593.1.S1_at	Histidine amino acid transporter	21.09
Mtr.12723.1.S1_at	Nt-gh3 deduced protein	20.97
Mtr.45331.1.S1_at	Hypothetical protein	20.90
Mtr.50585.1.S1_at	Proteinase inhibitor I3	20.80
Mtr.43746.1.S1_at	Hypothetical protein	20.66
Mtr.35994.1.S1_at	Hypothetical protein	20.64
Mtr.40293.1.S1_at	Glutathione S-transferase	20.50
Mtr.38067.1.S1_at	Hypothetical protein	20.42
Mtr.40882.1.S1_at	Aldehyde dehydrogenase	20.41
Mtr.28496.1.S1_at	Protein kinase	20.36
Mtr.32279.1.S1_at	SPF1 protein	20.36
Mtr.23311.1.S1_s_at	Glycosyl hydrolase family 4	20.31
Mtr.10683.1.S1_at	DNA-binding proteim	20.28
Mtr.50458.1.S1_at	Hypothetical protein	20.18
Mtr.11947.1.S1_at	Early growth response protein	20.09
Mtr.39160.1.S1_at	Hypothetical protein	19.97
Mtr.35302.1.S1_at	Galactokinase	19.97
Mtr.38233.1.S1_at	DAZ-associated protein 1	19.94
Mtr.40890.1.S1_at	WRKY transcription factor 40	19.93
Mtr.35178.1.S1_at	Branched-chain alpha keto-acid dehydrogenase E1-alpha	19.74
Mtr.45356.1.S1_at	Glutamate dehydrogenase	19.68
Mtr.47795.1.S1_at	Hypothetical protein	19.63
Mtr.13171.1.S1_at	Hypothetical protein	19.60
Mtr.12424.1.S1_at	Disease resistance response protein	19.55
Mtr.5797.1.S1_at	Hypothetical protein	19.49
Mtr.28696.1.S1_at	Hypothetical protein	19.39
Mtr.11466.1.S1_at	Galactokinase like protein	19.34
Mtr.43199.1.S1_at	Hypothetical protein	19.29
Mtr.42470.1.S1_at	Alpha galactosidase	19.19
Mtr.27728.1.S1_s_at	Hypothetical protein	19.04
Mtr.51996.1.S1_at	Hypothetical protein	18.98
Mtr.24422.1.S1_at	Hypothetical protein	18.97
Mtr.16487.1.S1_s_at	Zn-finger, CCHC type	18.90
Mtr.43280.1.S1_at	Cyanogenic Beta-Glucosidase	18.75
Mtr.37578.1.S1_at	Hypothetical protein	18.71
Mtr.21276.1.S1_at	TAZ finger	18.67
Mtr.4996.1.S1_at	Hypothetical protein	18.65
Mtr.10221.1.S1_at	Sugar transporter-like protein	18.63
Mtr.43716.1.S1_at	Glycosyltransferase	18.61
Mtr.34720.1.S1_at	Hypothetical protein	18.54
Mtr.48829.1.S1_at	Peptidase aspartic	18.50
Mtr.42470.1.S1_x_at	Alpha galactosidase	18.49
Mtr.35802.1.S1_at	Hypothetical protein	18.48
Mtr.39059.1.S1_at	Hypothetical protein	18.46
Mtr.50729.1.S1_at	Hypothetical protein	18.39

Msa.2738.1.S1_at	Hypothetical protein	18.38
Mtr.35987.1.S1_s_at	Hypothetical protein	18.36
Mtr.20803.1.S1_at	Hypothetical protein	18.33
Msa.2622.1.S1_at	Hypothetical protein	18.32
Mtr.41237.1.S1_at	Nt-gh3 deduced protein	18.22
Mtr.27713.1.S1_at	Cytochrome P450 71A8	18.17
Mtr.19224.1.S1_at	bZIP transcription factor	18.16
Mtr.32898.1.S1_at	Protein phosphatase 2C	18.15
Mtr.38907.1.S1_at	Alcohol dehydrogenase	18.15
Mtr.43203.1.S1_at	Hypothetical protein	18.14
Mtr.32486.1.S1_at	Strubbelig receptor	18.12
Mtr.12615.1.S1_at	Pathogenesis-related protein PR10A	18.12
Mtr.35.1.S1_at	Abscisic acid and environmental stress inducible protein	18.09
Mtr.32917.1.S1_s_at	Cytochrome oxidase subunit I	17.92
Mtr.12925.1.S1_at	RING/C3HC4/PHD zinc finger-like protein	17.85
Mtr.33150.1.S1_s_at	Syringolide-induced protein 19-1-5	17.77
Mtr.45768.1.S1_at	Serine/threonine protein kinase	17.70
Mtr.23575.1.S1_x_at	Polygalacturonase inhibitor protein	17.69
Mtr.38479.1.S1_at	Hypothetical protein	17.66
Mtr.40838.1.S1_at	Electron transfer flavoprotein, alpha subunit	17.63
Mtr.21098.1.S1_at	Oxysterol-binding protein	17.59
Mtr.40410.1.S1_at	Hypothetical protein	17.57
Mtr.8601.1.S1_at	Hypothetical protein	17.56
Mtr.12277.1.S1_at	Class 10 PR protein	17.56
Mtr.43159.1.S1_at	Bacterial-induced peroxidase	17.49
Mtr.8851.1.S1_at	Hypothetical protein	17.44
Mtr.7063.1.S1_at	Alcohol dehydrogenase	17.43
Mtr.40588.1.S1_at	Glutathione S-transferase GST 13	17.34
Mtr.48557.1.S1_at	Plant integral membrane protein	17.31
Mtr.48287.1.S1_s_at	Hypothetical protein	17.27
Mtr.37377.1.S1_at	Hypothetical protein	17.25
Mtr.41427.1.S1_at	Pyruvate, phosphate dikinase	17.23
Mtr.1214.1.S1_at	Hypothetical protein	17.21
Mtr.48070.1.S1_at	Hypothetical protein	17.21
Mtr.29902.1.S1_at	WRKY DNA-binding protein	17.16
Mtr.5512.1.S1_at	Biotin synthase	17.13
Mtr.5791.1.S1_at	Anthocyanin 3-O-glucoside-6"-O-malonyltransferase	17.12
Mtr.43231.1.S1_s_at	Hypothetical protein	17.03
Mtr.11118.1.S1_at	Calcium-dependent protein kinase 2	16.98
Mtr.43656.1.S1_at	Cysteine protease	16.91
Mtr.43115.1.S1_at	Pheromone receptor-like protein	16.87
Mtr.20215.1.S1_s_at	Hypothetical protein	16.86
Mtr.20486.1.S1_at	Hypothetical protein	16.85
Mtr.18629.1.S1_at	Hypothetical protein	16.84
Mtr.11285.1.S1_at	WAK-like kinase	16.81
Mtr.1886.1.S1_at	Hypothetical protein	16.79
Mtr.45989.1.S1_at	Xylose isomerase	16.78
Mtr.8987.1.S1_at	Cytochrome P450 monooxygenase	16.74
Mtr.10079.1.S1_at	Hypothetical protein	16.74
Mtr.18819.1.S1_at	2OG-Fe(II) oxygenase	16.62
Mtr.10494.1.S1_x_at	Glutathione S-transferase GST 7	16.59
Msa.1965.1.S1_at	Hypothetical protein	16.59
Mtr.40122.1.S1_s_at	Chalcone synthase 4	16.52
Mtr.13389.1.S1_at	Receptor protein kinase	16.51

Mtr.38564.1.S1_at	Nodulin-like protein	16.46
MsaAffx.3537.1.S1_s_at	Hypothetical protein	16.45
Mtr.9661.1.S1_at	Scarecrow-like 14	16.44
Mtr.40498.1.S1_at	Hypothetical protein	16.41
Mtr.9223.1.S1_at	AP2 domain transcription factor	16.36
Mtr.3130.1.S1_at	Hypothetical protein	16.29
Mtr.20391.1.S1_at	Hypothetical protein	16.25
Mtr.39762.1.S1_at	Chlorophyllase 1	16.15
Mtr.39563.1.S1_at	Hypothetical protein	16.10
Mtr.49012.1.S1_at	Zn-finger, RING	16.03
Mtr.5578.1.S1_at	Receptor protein kinase	16.02
Mtr.26939.1.S1_at	Hypothetical protein	16.01
Mtr.19134.1.S1_at	Hypothetical protein	15.99
Mtr.8538.1.S1_at	Common plant regulatory factor 7	15.93
Mtr.6769.1.S1_at	Hypothetical protein	15.91
Mtr.41091.1.S1_at	Mitogen-activated protein kinase MMK2	15.88
Mtr.44416.1.S1_at	WRKY-type DNA binding protein	15.87
Mtr.9474.1.S1_at	MLO-like protein 2	15.85
Mtr.47442.1.S1_s_at	Glycosyl hydrolase	15.77
Mtr.28255.1.S1_at	Cytochrome P450	15.76
Mtr.7741.1.S1_at	Peptide/amino acid transporter	15.73
Mtr.38492.1.S1_at	AX110P-like protein	15.68
Mtr.49730.1.S1_at	Hypothetical protein	15.68
Mtr.24820.1.S1_s_at	Fructan 1-exohydrolase	15.67
Mtr.48695.1.S1_at	Hypothetical protein	15.65
Mtr.653.1.S1_s_at	Short-chain dehydrogenase	15.64
Mtr.13850.1.S1_at	Hypothetical protein	15.61
Mtr.11022.1.S1_at	RING zinc finger protein	15.59
Mtr.31521.1.S1_at	Lysine and histidine specific transporter	15.50
Mtr.10122.1.S1_at	Nine-cis-epoxycarotenoid dioxygenase1	15.48
Msa.3122.1.S1_at	Hypothetical protein	15.48
Mtr.39165.1.S1_at	Cobalamin 5-phosphate synthase	15.44
Mtr.4724.1.S1_at	Organic anion transporter-like protein	15.30
Mtr.12139.1.S1_at	WRKY transcription factor 29	15.19
Mtr.37378.1.S1_at	Hypothetical protein	15.18
Mtr.49400.1.S1_at	Auxin responsive SAUR protein	15.16
Mtr.15309.1.S1_at	K+ channel tetramerisation	15.09
Mtr.28727.1.S1_at	Adenosine monophosphate binding protein 1	15.05
Mtr.37676.1.S1_at	Hypothetical protein	15.05
Mtr.1587.1.S1_at	Calmodulin-related protein	15.03
Msa.1042.1.S1_at	Hypothetical protein	15.02
Mtr.281.1.S1_at	Trehalose-6-phosphate phosphatase	14.99
Mtr.46962.1.S1_at	Hypothetical protein	14.98
Mtr.6648.1.S1_s_at	Xyloglucan endotransglycosylase	14.98
Mtr.9254.1.S1_at	Phe ammonia lyase	14.89
Mtr.11788.1.S1_at	Hypothetical protein	14.86
Mtr.6909.1.S1_at	Hypothetical protein	14.81
Mtr.10592.1.S1_at	Isoflavone reductase	14.79
Mtr.40605.1.S1_at	Xyloglucan-specific fungal endoglucanase inhibitor protein	14.76
Mtr.32991.1.S1_at	Hypothetical protein	14.73
Mtr.28125.1.S1_s_at	Early nodulin GRP3	14.72
Mtr.42620.1.S1_at	Organic anion transporter-like protein	14.72
Mtr.34990.1.S1_at	Cytochrome P450	14.68
Mtr.40195.1.S1_at	Pectin methylesterase 5	14.63

Mtr.10896.1.S1_s_at	WIZZ	14.63
Mtr.35324.1.S1_at	Peroxidase 2	14.61
Mtr.31288.1.S1_at	Isoflavonoid glucosyltransferase	14.54
Mtr.16848.1.S1_at	Harpin-induced 1	14.50
Mtr.11468.1.S1_at	Hypothetical protein	14.50
Mtr.37975.1.S1_at	CBL-interacting protein kinase	14.49
Mtr.11066.1.S1_s_at	AKIN gamma	14.47
Msa.2680.1.S1_at	Hypothetical protein	14.44
Mtr.48791.1.S1_at	Cys-rich domain protein	14.43
Mtr.32629.1.S1_s_at	Thaumatin-like protein PR-5a	14.39
Mtr.50276.1.S1_at	Phosphoinositide-binding clathrin adaptor protein	14.31
Mtr.14773.1.S1_at	Hypothetical protein	14.27
Mtr.43921.1.S1_at	Hypothetical protein	14.24
Mtr.25007.1.S1_at	Vestitone reductase	14.23
Mtr.28194.1.S1_at	Flavonol 3-O-glucosyltransferase 5	14.19
Mtr.11520.1.S1_s_at	Protein phosphatase 2C, partial (49%)	14.08
Mtr.43772.1.S1_at	Hypothetical protein	14.05
Mtr.6654.1.S1_at	Hypothetical protein	14.04
Mtr.51533.1.S1_at	Myb, DNA-binding protein	14.01
Mtr.28717.1.S1_at	Hypothetical protein	13.98
Mtr.5695.1.S1_at	Hypothetical protein	13.98
Mtr.25967.1.S1_at	Hypothetical protein	13.96
Mtr.11007.1.S1_at	Avr9/Cf-9 rapidly elicited protein	13.96
Mtr.12321.1.S1_at	MtN19-like protein	13.96
Mtr.38181.1.S1_at	Expansin-like protein B	13.88
Mtr.27510.1.S1_at	light resistance protein RGA1	13.87
Mtr.32646.1.S1_at	Hypothetical protein	13.87
Mtr.25889.1.S1_at	Protein kinase	13.86
Mtr.6933.1.S1_at	Methylcrotonyl-CoA carboxylase	13.85
Mtr.40048.1.S1_at	Acetylornithine aminotransferase	13.81
Mtr.10049.1.S1_at	Cycloidea protein	13.79
Mtr.39450.1.S1_at	Amino acid permease AAP3	13.79
Mtr.8522.1.S1_at	Syringolide-induced protein 19-1-5	13.68
Mtr.38075.1.S1_at	Hypothetical protein	13.65
Mtr.25575.1.S1_at	Hypothetical protein	13.64
Mtr.39710.1.S1_at	Hypothetical protein	13.60
Msa.1770.1.S1_at	Hypothetical protein	13.56
Mtr.32738.1.S1_s_at	Myo-inositol oxygenase	13.54
Mtr.33024.1.S1_at	Glucosidase-like protein	13.53
Mtr.38380.1.S1_at	Nodulin-like protein	13.48
Mtr.10971.1.S1_s_at	Serine/threonine protein kinase	13.42
Mtr.43230.1.S1_at	Hypothetical protein	13.37
Mtr.38612.1.S1_at	Phe ammonia lyase	13.27
Mtr.25969.1.S1_at	Hypothetical protein	13.27
Mtr.22854.1.S1_at	bHLH protein	13.23
Mtr.42202.1.S1_at	Hypothetical protein	13.22
Mtr.39140.1.S1_at	ABC transporter (PDR5-like)	13.14
Mtr.19367.1.S1_at	Zn-finger, C2H2 type	13.13
Mtr.43646.1.S1_at	Hypothetical protein	13.13
Mtr.44074.1.S1_at	Strubbelig receptor family	13.11
Mtr.8884.1.S1_at	Pathogenesis-related protein 4A	13.09
Mtr.34728.1.S1_at	Hypothetical protein	13.08
Mtr.43827.1.S1_at	Hypothetical protein	13.04
Mtr.11991.1.S1_s_at	Strubbelig receptor family	13.02

Mtr.6926.1.S1_at	Hypothetical protein	13.01
Mtr.15380.1.S1_at	Hypothetical protein	13.01
Mtr.27533.1.S1_s_at	Hypothetical protein	12.95
Mtr.17239.1.S1_at	Peptidase	12.95
Mtr.1331.1.S1_s_at	Hypothetical protein	12.93
Mtr.18596.1.S1_at	Zn-finger, RING	12.92
Mtr.11684.1.S1_at	Beta-D-glucosidase	12.85
Mtr.20187.1.S1_x_at	Naringenin-chalcone synthase	12.83
Mtr.35410.1.S1_at	Rho GDP dissociation inhibitor 2	12.75
Mtr.820.1.S1_at	Hypothetical protein	12.73
Mtr.27309.1.S1_at	Hypothetical protein	12.63
Mtr.42808.1.S1_at	Myb-related transcription factor	12.63
Mtr.12511.1.S1_at	Heat shock factor RHSF2	12.62
Mtr.18222.1.S1_at	Hypothetical protein	12.60
Mtr.48304.1.S1_s_at	Hypothetical protein	12.60
Mtr.33892.1.S1_at	Zinc finger protein constans-like 6	12.59
Mtr.6769.1.S1_s_at	Hypothetical protein	12.54
Mtr.19019.1.S1_at	No apical meristem (NAM) protein	12.50
Mtr.22179.1.S1_at	Hypothetical protein	12.49
Mtr.18040.1.S1_at	Hypothetical protein	12.48
Mtr.4967.1.S1_at	GTPase activating protein	12.48
Mtr.35852.1.S1_at	Auxin-induced protein	12.47
Mtr.41223.1.S1_at	ATP-dependent phosphoenolpyruvate carboxykinase	12.47
Mtr.19182.1.S1_at	Hypothetical protein	12.43
Msa.1404.1.S1_at	Hypothetical protein	12.42
Mtr.13722.1.S1_at	Hypothetical protein	12.41
Mtr.15332.1.S1_at	Myb DNA-binding protein	12.39
Msa.3124.1.S1_at	Hypothetical protein	12.33
Mtr.15470.1.S1_s_at	Amino acid/polyamine transporter II	12.31
Mtr.44194.1.S1_at	CPRD2 protein	12.29
Mtr.50317.1.S1_at	Zn-finger, RING	12.25
Mtr.32982.1.S1_at	MutT-like protein	12.23
Mtr.37455.1.S1_at	Glucosyltransferase-13	12.17
Mtr.8853.1.S1_at	Arginase 1	12.13
Mtr.33191.1.S1_at	Hypothetical protein	12.13
Mtr.39384.1.S1_at	Hypothetical protein	12.12
Mtr.52242.1.S1_at	Galactokinase	12.10
Mtr.10696.1.S1_at	Syntaxin-related protein	12.08
Mtr.41031.1.S1_at	4-coumarate-CoA ligase	12.03
Msa.1264.1.S1_at	Hypothetical protein	12.00
Mtr.33206.1.S1_s_at	Lustrin A-like protein	11.99
Mtr.32702.1.S1_at	Hypothetical protein	11.97
Mtr.39195.1.S1_at	Nitrate transporter NTL1	11.95
Mtr.31575.1.S1_at	Phosphoribosylanthranilate transferase	11.94
Mtr.28033.1.S1_at	Hypothetical protein	11.94
Mtr.23311.1.S1_at	Glycosyl hydrolase family 3	11.93
Mtr.12466.1.S1_at	Chitinase	11.91
Mtr.15470.1.S1_at	Amino acid/polyamine transporter II	11.86
Mtr.22536.1.S1_at	Hypothetical protein	11.86
Mtr.19407.1.S1_at	Octicosapeptide/Phox/Bem1p	11.85
Mtr.35833.1.S1_s_at	Hypothetical protein	11.85
Mtr.46961.1.S1_at	Hypothetical protein	11.84
Mtr.18113.1.S1_at	Hypothetical protein	11.83
Mtr.35110.1.S1_at	Hypothetical protein	11.81

Mtr.9997.1.S1_at	Glucan endo-1,3-beta-glucosidase	11.78
Mtr.1854.1.S1_at	Hypothetical protein	11.76
Mtr.12097.1.S1_at	MtN19-like protein	11.76
Mtr.11543.1.S1_at	Gamma-glutamyl hydrolase	11.75
Mtr.22769.1.S1_at	Hypothetical protein	11.71
Mtr.34126.1.S1_at	Hypothetical protein	11.69
Mtr.27695.1.S1_at	Auxin response factor	11.68
Mtr.35294.1.S1_at	Protein kinase	11.65
Mtr.41894.1.S1_at	AT-hook DNA-binding protein	11.55
Mtr.26378.1.S1_at	Hypothetical protein	11.52
Mtr.30776.1.S1_at	Patatin-like protein 1	11.50
Mtr.37879.1.S1_at	Hypothetical protein	11.44
Mtr.17180.1.S1_at	Galactose mutarotase	11.42
Mtr.28177.1.S1_at	RAB11F	11.40
Mtr.46462.1.S1_at	AMP-dependent synthetase and ligase	11.38
Mtr.40601.1.S1_s_at	Hypothetical protein	11.38
Mtr.7214.1.S1_at	Hypothetical protein	11.37
Mtr.11728.1.S1_at	Hypothetical protein	11.37
Mtr.43900.1.S1_at	Hypothetical protein	11.37
Mtr.40611.1.S1_at	Peroxidase	11.36
Mtr.10557.1.S1_at	PDR-type ABC transporter 2	11.33
Msa.2755.1.S1_at	Hypothetical protein	11.29
Mtr.43862.1.S1_at	Hypothetical protein	11.25
Mtr.37878.1.S1_at	Hypothetical protein	11.25
Mtr.50290.1.S1_s_at	Hypothetical protein	11.22
Mtr.50994.1.S1_at	CHY zinc finger protein	11.21
Mtr.14737.1.S1_at	Protein kinase	11.20
Mtr.35045.1.S1_at	Rab escort protein	11.20
Mtr.29408.1.S1_at	Hypothetical protein	11.18
Mtr.9819.1.S1_at	Hypothetical protein	11.18
Mtr.9258.1.S1_at	Receptor-like protein kinase	11.15
Mtr.24370.1.S1_at	3'-5' exonuclease	11.13
Mtr.46408.1.S1_at	Hypothetical protein	11.12
Mtr.39132.1.S1_at	Malate synthase	11.12
Mtr.33334.1.S1_at	Exo-1,3-beta-glucanase	11.09
Mtr.12087.1.S1_at	Hypothetical protein	11.07
Mtr.29374.1.S1_at	AKIN gamma	11.07
Mtr.43294.1.S1_at	Non-specific lipid transfer-like protein	11.07
Mtr.696.1.S1_at	Auxin-induced (indole-3-acetic acid induced) protein	11.06
Msa.2329.1.S1_at	Hypothetical protein	11.05
Mtr.43916.1.S1_at	Isoflavone reductase	11.04
Mtr.8531.1.S1_at	Chalcone-flavonone isomerase 1	11.01
Mtr.35413.1.S1_at	O-methyltransferase	11.00
Mtr.1203.1.S1_at	CCR4-associated factor-related protein	10.99
Mtr.8688.1.S1_at	Hypothetical protein	10.94
Msa.2990.1.S1_at	Hypothetical protein	10.94
Mtr.12710.1.S1_at	Syringolide-induced protein B13-1-1	10.90
Mtr.27221.1.S1_at	Hypothetical protein	10.87
Mtr.18369.1.S1_at	Glutathione S-transferase	10.86
Mtr.43392.1.S1_s_at	Cytochrome b	10.83
Mtr.23988.1.S1_at	bHLH protein	10.82
Mtr.15568.1.S1_s_at	DNA-binding WRKY	10.80
Mtr.3431.1.S1_at	Hypothetical protein	10.80
Mtr.11968.1.S1_at	Alpha galactosidase	10.76

Mtr.49931.1.S1_at	E-class P450	10.75
Msa.3054.1.S1_at	Hypothetical protein	10.72
Mtr.44823.1.S1_at	Hypothetical protein	10.72
Mtr.34771.1.S1_at	Hypothetical protein	10.72
Mtr.34448.1.S1_at	UDP-glucose 4-epimerase	10.70
Mtr.10018.1.S1_at	Hypothetical protein	10.67
Mtr.42663.1.S1_at	Ferredoxin-dependent glutamate synthase	10.66
Mtr.43783.1.S1_at	Hypothetical protein	10.65
Mtr.9388.1.S1_at	WRKY transcription factor 23	10.63
Mtr.42072.1.S1_at	Nodulin	10.62
Mtr.35080.1.S1_at	Trehalose-6-phosphate synthase	10.59
Mtr.10220.1.S1_s_at	Isoliquiritigenin 2'-O-methyltransferase	10.59
Mtr.28687.1.S1_at	Wax synthase	10.52
Mtr.49572.1.S1_s_at	Naringenin-chalcone synthase	10.51
Mtr.45989.1.S1_s_at	Xylose isomerase	10.48
Mtr.40223.1.S1_at	Hypothetical protein	10.44
Mtr.11134.1.S1_at	Ornithine aminotransferase	10.40
Mtr.11636.1.S1_at	Dof zinc finger protein	10.39
Mtr.40619.1.S1_at	Deltex protein	10.39
Msa.1051.1.S1_at	Hypothetical protein	10.36
Mtr.6317.1.S1_at	DUR3	10.36
Mtr.40983.1.S1_at	Hypothetical protein	10.36
Mtr.19471.1.S1_at	Hypothetical protein	10.35
Mtr.32374.1.S1_at	Serine carboxypeptidase	10.34
Mtr.13455.1.S1_at	Hypothetical protein	10.33
Mtr.45000.1.S1_at	Trehalose-6-phosphate synthase/phosphatase	10.32
Mtr.41039.1.S1_at	Hypothetical protein	10.31
Mtr.1746.1.S1_at	Glucosyltransferase-9	10.31
Msa.1267.1.S1_at	Hypothetical protein	10.30
Mtr.12866.1.S1_at	Hypothetical protein	10.30
Mtr.6489.1.S1_at	Glutathione S-transferase GST 15	10.29
Mtr.16167.1.S1_at	Hypothetical protein	10.27
Mtr.6916.1.S1_s_at	Hypothetical protein	10.27
Mtr.43887.1.S1_at	Hypothetical protein	10.25
Mtr.43494.1.S1_at	Hypothetical protein	10.25
Mtr.37168.1.S1_at	Hypothetical protein	10.24
Mtr.40371.1.S1_at	Avr9/Cf-9 rapidly elicited protein	10.21
Mtr.35775.1.S1_at	Hypothetical protein	10.20
Mtr.41783.1.S1_at	Hypothetical protein	10.20
Mtr.20344.1.S1_at	Seven in absentia protein	10.19
Mtr.24851.1.S1_at	Hypothetical protein	10.19
Mtr.28790.1.S1_at	Hypothetical protein	10.18
Msa.1669.1.S1_at	Hypothetical protein	10.16
Mtr.2844.1.S1_at	Hypothetical protein	10.13
Mtr.3072.1.S1_at	Disease resistance protein	10.12
Mtr.38568.1.S1_at	Kelch repeat-containing F-box-like protein	10.11
Mtr.45350.1.S1_at	Hypothetical protein	10.11
Mtr.40194.1.S1_at	Hypothetical protein	10.07
Mtr.37520.1.S1_at	Cinnamoyl CoA reductase	10.07
Mtr.7569.1.S1_at	Cytosolic fructose-1,6-bisphosphatase	10.06
Mtr.40680.1.S1_at	Transcription factor MYBS3	10.05
Mtr.12105.1.S1_at	Receptor protein kinase	10.05
Mtr.38086.1.S1_at	Alpha-N-acetylglucosaminidase	10.04
Mtr.49572.1.S1_x_at	Naringenin-chalcone synthase	10.04

Msa.2171.1.S1_at	Hypothetical protein	10.02
Mtr.41299.1.S1_at	Hypothetical protein	10.01
Msa.3019.1.S1_at	Hypothetical protein	9.99
Mtr.35088.1.S1_at	Hypothetical protein	9.99
Mtr.10396.1.S1_at	Syringolide-induced protein	9.99
Msa.2259.1.S1_at	Hypothetical protein	9.96
Msa.1129.1.S1_at	Hypothetical protein	9.89
Mtr.33213.1.S1_at	Hypothetical protein	9.88
Mtr.27726.1.S1_at	Rod shape-determining protein	9.88
Mtr.3602.1.S1_at	Hypothetical protein	9.82
Msa.3062.1.S1_at	Hypothetical protein	9.81
Mtr.40724.1.S1_at	Hypothetical protein	9.78
Mtr.40531.1.S1_at	O-methyltransferase	9.77
Mtr.38202.1.S1_at	WRKY transcription factor 28	9.76
Mtr.38093.1.S1_at	Hypothetical protein	9.74
Mtr.49862.1.S1_at	Isoflavone-7-o-methytransferase 9	9.74
Mtr.18747.1.S1_at	Carbamoyl-phosphate synthase	9.74
Mtr.43561.1.S1_s_at	Calcium/calmodulin-regulated receptor-like kinase	9.70
Mtr.8617.1.S1_at	Syringolide-induced protein B15-3-5	9.69
Mtr.21324.1.S1_at	ABC transporter	9.68
Mtr.12684.1.S1_at	Hypothetical protein	9.67
Mtr.42782.1.S1_s_at	Hypothetical protein	9.67
Mtr.19625.1.S1_at	Ribonuclease III	9.64
Mtr.37262.1.S1_at	Hypothetical protein	9.64
Mtr.31589.1.S1_at	Arm repeat protein	9.62
Mtr.23085.1.S1_s_at	Hypothetical protein	9.61
Mtr.9897.1.S1_s_at	Glycerol kinase	9.61
Mtr.8606.1.S1_at	O-methyltransferase	9.61
Mtr.5605.1.S1_at	Protein phosphatase 2C	9.61
Mtr.8813.1.S1_at	Zinc finger protein	9.56
Msa.1879.1.S1_at	Hypothetical protein	9.55
Mtr.46208.1.S1_at	Hypothetical protein	9.53
Mtr.13161.1.S1_at	Hypothetical protein	9.53
Mtr.37873.1.S1_at	Hypothetical protein	9.53
Mtr.12291.1.S1_s_at	Proline dehydrogenase	9.51
Mtr.7608.1.S1_at	50S ribosomal protein L33	9.50
Mtr.42739.1.S1_at	Protein kinase	9.47
Mtr.40177.1.S1_at	Hypothetical protein	9.43
Mtr.35086.1.S1_at	Hypothetical protein	9.42
Mtr.24896.1.S1_at	Hypothetical protein	9.42
Mtr.1951.1.S1_s_at	Zinc finger protein	9.42
Mtr.20187.1.S1_at	Naringenin-chalcone synthase	9.41
Mtr.9524.1.S1_at	Early flowering 3	9.40
Mtr.8827.1.S1_at	Cytochrome P450	9.40
Mtr.40521.1.S1_at	Adhesive/proline-rich protein	9.38
Mtr.7556.1.S1_at	AP2 domain containing protein	9.36
Mtr.12477.1.S1_at	Quinone-oxidoreductase QR1	9.33
Mtr.31649.1.S1_at	Hypothetical protein	9.31
Mtr.43605.1.S1_at	Glycosyl transferase	9.31
Mtr.38932.1.S1_at	Malate synthase	9.27
Mtr.11414.1.S1_at	PDR-like ABC transporter	9.25
Mtr.42084.1.S1_at	Hypothetical protein	9.25
Mtr.25509.1.S1_at	Hypothetical protein	9.24
Mtr.12648.1.S1_at	Hypothetical protein	9.21

Mtr.43278.1.S1_at	Cellulose synthase	9.18
Mtr.35430.1.S1_s_at	Hypothetical protein	9.17
Mtr.39945.1.S1_at	Hypothetical protein	9.16
Mtr.17570.1.S1_at	Zn-finger, RING	9.16
Mtr.31913.1.S1_at	Hypothetical protein	9.16
Mtr.39267.1.S1_at	1-aminocyclopropane-1-carboxylate synthase	9.14
Mtr.42078.1.S1_at	Anthocyanin 5-aromatic acyltransferase	9.14
Mtr.8645.1.S1_at	Riboflavin biosynthesis protein	9.13
Mtr.3060.1.S1_s_at	Hypothetical protein	9.11
Mtr.10616.1.S1_at	Hypothetical protein	9.11
Mtr.42703.1.S1_at	Hypothetical protein	9.05
Mtr.33446.1.S1_at	Sucrose transporter	9.04
Mtr.41419.1.S1_at	Ring domain containing protein	9.04
Mtr.36186.1.S1_at	Nitrate transporter NTL1	9.01
Mtr.10743.1.S1_at	SRG1 protein	9.00
Mtr.45572.1.S1_at	Hypothetical protein	8.99
Mtr.35243.1.S1_at	Hypothetical protein	8.97
Mtr.23580.1.S1_at	Polygalacturonase inhibitor protein	8.96
Mtr.38844.1.S1_at	Hypothetical protein	8.95
Mtr.36847.1.S1_at	Hypothetical protein	8.94
Mtr.48884.1.S1_at	Hypothetical protein	8.93
Mtr.51571.1.S1_s_at	Amino acid/polyamine transporter II	8.93
Mtr.474.1.S1_s_at	Peroxisomal membrane protein	8.92
Mtr.19162.1.S1_at	AMP-dependent synthetase and ligase	8.92
Mtr.26898.1.S1_at	Hypothetical protein	8.91
Mtr.15997.1.S1_s_at	Haem peroxidase	8.91
Mtr.45912.1.S1_at	Pathogenesis-related transcriptional factor and ERF	8.86
Mtr.5817.1.S1_at	Phospholipid-transporting ATPase 1	8.85
Mtr.35172.1.S1_at	Hypothetical protein	8.83
Mtr.11385.1.S1_at	Hypothetical protein	8.80
Mtr.8876.1.S1_at	Short-chain dehydrogenase Tic32	8.79
Mtr.11811.1.S1_s_at	Phi-1-like protein	8.78
Mtr.42692.1.S1_at	Glutaredoxin-like protein	8.78
Mtr.1625.1.S1_s_at	Protein kinase	8.76
Mtr.45193.1.S1_at	Hypothetical protein	8.73
Mtr.4576.1.S1_s_at	Indole-3-acetate beta-glucosyltransferase	8.72
Mtr.18822.1.S1_at	Heavy metal transport/detoxification protein	8.71
Mtr.43944.1.S1_at	Sporulation-specific glucan 1,3-beta-glucosidase	8.71
Mtr.24063.1.S1_at	F-box protein	8.70
Mtr.38330.1.S1_at	Hypothetical protein	8.69
Mtr.14303.1.S1_at	Iron hydrogenase	8.69
Mtr.20425.1.S1_at	Hypothetical protein	8.66
Msa.686.1.S1_at	Hypothetical protein	8.66
Mtr.9637.1.S1_at	Myb-related transcription factor	8.65
Mtr.10370.1.S1_at	Peroxidase1B	8.64
Mtr.12044.1.S1_at	Cellulose synthase	8.64
Mtr.15849.1.S1_at	Glycine-rich RNA binding protein	8.63
Mtr.44130.1.S1_s_at	Hypothetical protein	8.63
Mtr.35231.1.S1_s_at	Pathogenesis-related protein	8.62
Mtr.14494.1.S1_at	Dihydroorotate dehydrogenase 1	8.59
Mtr.12664.1.S1_at	Hypothetical protein	8.55
Mtr.39298.1.S1_at	Hypothetical protein	8.54
Mtr.15772.1.S1_at	Glycoside hydrolase	8.53
Mtr.47441.1.S1_at	Hypothetical protein	8.53

Mtr.29300.1.S1_s_at	Hypothetical protein	8.53
Mtr.35813.1.S1_at	Hypothetical protein	8.51
Mtr.11783.1.S1_at	Hypothetical protein	8.51
Mtr.16822.1.S1_at	Hypothetical protein	8.50
Mtr.43585.1.S1_at	1-deoxy-D-xylulose 5-phosphate synthase 2	8.50
Mtr.27830.1.S1_at	Arginine/serine-rich protein	8.49
Mtr.34199.1.S1_at	2-oxoglutarate-dependent oxygenase	8.46
Mtr.10575.1.S1_at	Hypothetical protein	8.45
Mtr.27129.1.S1_at	Zinc finger protein	8.44
Mtr.13757.1.S1_at	Hin1-like protein	8.43
Mtr.40333.1.S1_x_at	Uclacyanin II precursor	8.43
Mtr.20804.1.S1_at	Serine/threonine protein kinase	8.42
Mtr.33361.1.S1_at	Hypothetical protein	8.42
Mtr.15250.1.S1_at	ABC transporter	8.41
Mtr.44775.1.S1_at	G-protein alpha-subunit	8.40
Mtr.40928.1.S1_at	PGPD14 protein	8.40
Mtr.17788.1.S1_at	Ankyrin	8.37
Mtr.33365.1.S1_s_at	PDR7 ABC transporter	8.37
Mtr.40076.1.S1_at	RNA Binding Protein 45	8.36
Mtr.9523.1.S1_at	Hypothetical protein	8.36
Mtr.27095.1.S1_at	Hypothetical protein	8.36
Mtr.12832.1.S1_at	Receptor-like serine/threonine kinase	8.32
Mtr.19206.1.S1_at	Ras GTPase	8.31
Mtr.9392.1.S1_at	Flavonoid 3'-hydroxylase	8.28
Mtr.238.1.S1_at	Metal transport protein	8.23
Mtr.31946.1.S1_at	Hypothetical protein	8.21
Mtr.38485.1.S1_at	Hypothetical protein	8.21
Mtr.42373.1.S1_at	Peroxidase 40	8.18
Mtr.9079.1.S1_at	Hypothetical protein	8.18
Mtr.29368.1.S1_at	Receptor-like protein kinase	8.17
Msa.1878.1.S1_at	Hypothetical protein	8.16
Mtr.49680.1.S1_at	Hypothetical protein	8.15
Mtr.5044.1.S1_s_at	Hypothetical protein	8.15
Mtr.49578.1.S1_at	Hypothetical protein	8.15
Mtr.41930.1.S1_at	Hypothetical protein	8.14
Mtr.43416.1.S1_at	Zinc-finger protein	8.11
Mtr.22903.1.S1_at	Chitinase (Class II)	8.09
Mtr.43734.1.S1_at	Protein kinase C	8.09
Mtr.10127.1.S1_at	Phospholipase-like protein	8.08
Mtr.13044.1.S1_at	WRKY transcription factor 5	8.07
Mtr.44516.1.S1_at	Type IIB calcium ATPase	8.05
Mtr.44349.1.S1_at	Zinc transporter	8.04
Mtr.33142.1.S1_at	Hypothetical protein	8.04
Mtr.8654.1.S1_at	Peroxidase	8.04
Msa.1604.1.S1_at	Hypothetical protein	8.03
Mtr.38064.1.S1_at	Hypothetical protein	8.03
Mtr.25476.1.S1_at	Auxin efflux carrier protein	8.01
Mtr.14090.1.S1_at	AUX/IAA protein	8.00
Mtr.9063.1.S1_at	Glucose/sorbosone dehydrogenases	8.00
Mtr.9668.1.S1_at	Hypothetical protein	7.99
Mtr.43236.1.S1_at	UDP-glucosyltransferase HRA25	7.97
Msa.952.1.S1_at	Hypothetical protein	7.97
Mtr.11087.1.S1_at	Hypothetical protein	7.96
Mtr.12377.1.S1_at	WRKY transcription factor 11	7.92

Mtr.11256.1.S1_at	Hypothetical protein	7.91
Mtr.32555.1.S1_s_at	Vitellogenin A	7.91
Mtr.10681.1.S1_at	Universal stress protein USP1-like protein	7.90
Mtr.4934.1.S1_s_at	Hypothetical protein	7.90
Mtr.41809.1.S1_s_at	Non-phototropic hypocotyl 3	7.90
Mtr.39431.1.S1_at	Aldehyde reductase	7.88
Mtr.15773.1.S1_at	Cyclin-like F-box	7.87
Mtr.32284.1.S1_at	Hypothetical protein	7.87
Mtr.41342.1.S1_at	Serine/threonine-protein kinase 24	7.86
Mtr.11621.1.S1_at	Hypothetical protein	7.85
Mtr.39423.1.S1_at	Hypothetical protein	7.85
Mtr.40277.1.S1_at	Isoflavone synthase	7.84
Mtr.42263.1.S1_at	Hypothetical protein	7.84
Mtr.41149.1.S1_at	Glutathione S-transferase GST 11	7.84
Mtr.13586.1.S1_at	Transcription activator	7.83
Mtr.49896.1.S1_at	Non-cell-autonomous protein	7.81
Mtr.38443.1.S1_at	Hypothetical protein	7.80
Mtr.37253.1.S1_at	Amino acid transporter	7.79
Mtr.49441.1.S1_at	2OG-Fe(II) oxygenase	7.79
Mtr.39741.1.S1_s_at	Hypothetical protein	7.79
Mtr.30654.1.S1_at	Hypothetical protein	7.77
Msa.2372.1.S1_at	Hypothetical protein	7.77
Msa.1067.1.S1_at	Hypothetical protein	7.76
Mtr.44144.1.S1_at	Lap1 protein	7.75
Mtr.21216.1.S1_at	Exo70 exocyst complex subunit	7.72
Mtr.27096.1.S1_at	Cinnamyl-alcohol dehydrogenase	7.72
Mtr.49085.1.S1_at	GCN5-related N-acetyltransferase	7.71
Mtr.40277.1.S1_x_at	Isoflavone synthase	7.70
Mtr.3066.1.S1_s_at	Hypothetical protein	7.69
Mtr.13470.1.S1_s_at	Hypothetical protein	7.69
Mtr.15053.1.S1_at	Thaumatin	7.67
Mtr.12290.1.S1_at	Proline dehydrogenase	7.67
Mtr.42955.1.S1_at	Cytochrome P450	7.67
Mtr.51195.1.S1_at	AAA ATPase	7.67
Mtr.20618.1.S1_at	Transferase	7.66
Mtr.51997.1.S1_at	Hypothetical protein	7.66
Mtr.11565.1.S1_at	Patatin-like protein	7.65
Mtr.25887.1.S1_at	bHLH protein	7.65
Mtr.14686.1.S1_at	Peptidase aspartic	7.64
Mtr.11716.1.S1_at	Methionine sulfoxide reductase A	7.64
Mtr.9421.1.S1_at	Indole-3-acetate beta-glucosyltransferase	7.63
Mtr.47412.1.S1_at	Hypothetical protein	7.62
Mtr.6834.1.S1_at	Zeatin O-glucosyltransferase	7.61
Mtr.48874.1.S1_at	Hypothetical protein	7.61
Mtr.33482.1.S1_s_at	Carbonic anhydrase 2	7.59
Mtr.1401.1.S1_at	Hypothetical protein	7.56
Mtr.10681.1.S1_s_at	Universal stress protein USP1-like protein	7.52
Mtr.7267.1.S1_at	Hypothetical protein	7.50
Mtr.2157.1.S1_s_at	Hypothetical protein	7.50
Mtr.45320.1.S1_at	Hypothetical protein	7.49
Mtr.44941.1.S1_s_at	Glyoxysomal fatty acid beta-oxidation multifunctional protein	7.48
Mtr.17409.1.S1_s_at	Amino acid/polyamine transporter II	7.48
Mtr.33221.1.S1_s_at	acyl-activating enzyme	7.48
Mtr.10308.1.S1_at	Hydroxyproline-rich glycoprotein	7.48

Msa.2831.1.S1_at	Hypothetical protein	7.48
Mtr.42626.1.S1_at	Hypothetical protein	7.47
Mtr.10655.1.S1_at	Hypothetical protein	7.47
Mtr.34766.1.S1_at	Zinc finger protein	7.44
Mtr.8444.1.S1_s_at	Hypothetical protein	7.43
Mtr.51734.1.S1_at	Hypothetical protein	7.43
Mtr.22675.1.S1_at	Hypothetical protein	7.43
Mtr.15895.1.S1_s_at	Dormancyauxin associated protein	7.43
Mtr.39679.1.S1_at	Hypothetical protein	7.42
Mtr.28572.1.S1_at	Cytochrome b	7.42
Mtr.47292.1.S1_at	Hypothetical protein	7.40
Mtr.12540.1.S1_s_at	Receptor kinase-like protein	7.37
Mtr.18344.1.S1_at	Serine/threonine protein kinase	7.37
Mtr.12694.1.S1_at	NAC domain protein NAC1	7.37
Mtr.31177.1.S1_at	Ubiquitin-protein ligase	7.36
Mtr.35741.1.S1_at	Hypothetical protein	7.35
Mtr.10701.1.S1_at	Axi 1 protein-like protein	7.35
Mtr.17829.1.S1_at	UDP-glucose 4-epimerase	7.35
Mtr.42855.1.S1_s_at	Plastidic ATP/ADP transporter	7.34
Mtr.51734.1.S1_s_at	Hypothetical protein	7.34
Mtr.11388.1.S1_at	Transcription factor	7.33
Mtr.41533.1.S1_at	Calcium binding protein	7.32
Mtr.3151.1.S1_s_at	Hypothetical protein	7.31
Mtr.33855.1.S1_at	Alpha-N-acetylglucosaminidase	7.30
Mtr.37757.1.S1_at	Anthranilate N-benzoyltransferase-like protein	7.30
Mtr.20508.1.S1_s_at	Pyridine nucleotide-disulphide oxidoreductase	7.30
Mtr.43550.1.S1_at	Tyrosine aminotransferase	7.28
Mtr.17497.1.S1_at	bHLH protein	7.28
Mtr.42299.1.S1_at	Hypothetical protein	7.26
Mtr.28796.1.S1_at	Cytochrome P450 monooxygenase	7.25
Mtr.44122.1.S1_at	Hypothetical protein	7.24
Mtr.50492.1.S1_s_at	Translation factor	7.23
Mtr.41506.1.S1_at	Hypothetical protein	7.22
Mtr.11156.1.S1_at	Leucine-rich repeat receptor-like protein kinase 1	7.21
Mtr.45877.1.S1_at	RNA-binding region RNP-1	7.20
Mtr.38072.1.S1_at	Hypothetical protein	7.18
Mtr.8948.1.S1_at	Cytochrome P450 82A1	7.18
Msa.2506.1.S1_at	Hypothetical protein	7.18
Mtr.12708.1.S1_at	Isoflavone reductase	7.17
Mtr.6009.1.S1_at	Hypothetical protein	7.16
Mtr.17610.1.S1_at	Hypothetical protein	7.16
Mtr.28550.1.S1_at	Hypothetical protein	7.16
Mtr.38022.1.S1_at	LEXYL1 protein	7.16
Mtr.49091.1.S1_at	Hypothetical protein	7.14
Mtr.2735.1.S1_at	Hypothetical protein	7.14
Mtr.37708.1.S1_at	Sulfate transporter-like protein	7.12
Mtr.6049.1.S1_s_at	S-receptor kinase	7.12
Mtr.17940.1.S1_at	U box protein	7.12
Mtr.15228.1.S1_at	Pre-mRNA processing ribonucleoprotein	7.11
Mtr.10027.1.S1_at	Hypothetical protein	7.09
Mtr.43364.1.S1_at	Hypothetical protein	7.08
Mtr.9897.1.S1_at	Glycerol kinase	7.08
Mtr.48873.1.S1_s_at	Acid phosphatase (Class B)	7.07
Mtr.16539.1.S1_s_at	Hypothetical protein	7.07

Mtr.6875.1.S1_at	Hypothetical protein	7.06
Mtr.29535.1.S1_x_at	Hypothetical protein	7.06
Mtr.34958.1.S1_at	Hypothetical protein	7.06
Mtr.8621.1.S1_s_at	Hypothetical protein	7.06
Mtr.37823.1.S1_at	CPRD49 protein	7.06
Mtr.38247.1.S1_at	Seven in absentia-like protein	7.05
Mtr.38959.1.S1_s_at	GTP-binding protein	7.05
Mtr.11202.1.S1_s_at	Hypothetical protein	7.04
Mtr.12749.1.S1_at	Hypothetical protein	7.03
Mtr.39742.1.S1_at	Hypothetical protein	7.03
Mtr.42673.1.S1_at	Receptor-like serine/threonine kinase	7.03
Mtr.35144.1.S1_s_at	acyl-activating enzyme 13	7.03
Mtr.10090.1.S1_at	Homo-phytochelatin synthase	7.02
Mtr.4702.1.S1_at	Fiber protein Fb1	7.02
Mtr.40880.1.S1_at	Electron transfer flavoprotein beta-subunit	7.00
Mtr.43144.1.S1_at	NtEIG-E80 protein	6.97
Mtr.49088.1.S1_at	Rhodanese-like protein	6.96
Mtr.46200.1.S1_at	CCCH-type zinc finger protein	6.96
Mtr.41563.1.S1_s_at	Ring finger E3 ligase	6.96
Mtr.7255.1.S1_at	Arm repeat protein	6.94
Mtr.52294.1.S1_at	DNA photolyase	6.93
Mtr.12378.1.S1_at	Serine/threonine-protein kinase	6.93
Mtr.7397.1.S1_at	Heat shock transcription factor 34	6.92
Mtr.40125.1.S1_at	Peroxidase1B	6.91
Mtr.49055.1.S1_s_at	Hypothetical protein	6.91
Mtr.21028.1.S1_at	Hypothetical protein	6.90
Mtr.38349.1.S1_at	NAM (no apical meristem)-like protein	6.89
Mtr.50766.1.S1_at	Hypothetical protein	6.88
Mtr.43825.1.S1_at	Hypothetical protein	6.87
Mtr.29335.1.S1_at	Receptor-like protein kinase ARK1	6.87
Mtr.8933.1.S1_at	Homogentisate 1,2-dioxygenase	6.87
Mtr.11053.1.S1_at	WRKY-type transcription factor	6.87
Mtr.7906.1.S1_at	Vasopressin V1b receptor	6.87
Mtr.4906.1.S1_s_at	1-aminocyclopropane-1-carboxylate oxidase	6.87
Mtr.23572.1.S1_at	Hypothetical protein	6.86
Mtr.48787.1.S1_s_at	WD-40 repeat protein	6.85
Mtr.19871.1.S1_at	Calcium-binding EF-hand	6.85
Mtr.13594.1.S1_s_at	Lipase-like protein	6.84
Mtr.34980.1.S1_s_at	Hypothetical protein	6.84
Mtr.45910.1.S1_at	Hypothetical protein	6.83
Mtr.41255.1.S1_at	Amino acid permease AAP3	6.82
Mtr.32195.1.S1_at	Urea active transporter	6.82
Mtr.43455.1.S1_at	Hypothetical protein	6.81
Mtr.24411.1.S1_at	Hypothetical protein	6.81
Mtr.4083.1.S1_s_at	Hypothetical protein	6.81
Msa.3071.1.S1_at	Hypothetical protein	6.80
Mtr.5781.1.S1_at	Hypothetical protein	6.79
Mtr.246.1.S1_at	DnaJ-like protein	6.79
Mtr.12685.1.S1_at	Calcium/calmodulin-regulated receptor-like kinase	6.78
Mtr.45080.1.S1_at	Retinoic acid receptor alpha (RAR-alpha)	6.78
Mtr.33275.1.S1_s_at	Hypothetical protein	6.77
Mtr.48109.1.S1_at	WRKY4 transcription factor	6.76
Mtr.8726.1.S1_at	Hypothetical protein	6.76
Mtr.50358.1.S1_at	Hypothetical protein	6.74

Mtr.5869.1.S1_at	Hexose carrier protein	6.74
Mtr.43291.1.S1_at	Fiber protein Fb1	6.72
Mtr.40066.1.S1_at	Hydroxyproline-rich glycoprotein	6.72
Mtr.37347.1.S1_s_at	Hypothetical protein	6.71
Mtr.6889.1.S1_at	Hypothetical protein	6.67
Mtr.11488.1.S1_at	Hypothetical protein	6.67
Mtr.31452.1.S1_at	Hypothetical protein	6.65
Mtr.38358.1.S1_s_at	Hypothetical protein	6.65
Mtr.40847.1.S1_at	Heat shock transcription factor HSF30	6.65
Mtr.10325.1.S1_at	Chitinase	6.63
Mtr.24481.1.S1_at	Hypothetical protein	6.63
Mtr.29298.1.S1_at	Hypothetical protein	6.63
Mtr.39754.1.S1_at	NAM (No apical meristem)-like protein	6.63
Mtr.26988.1.S1_s_at	Hypothetical protein	6.62
Mtr.43497.1.S1_at	Ethylene-forming-enzyme-like dioxygenase	6.62
Mtr.35704.1.S1_at	Hypothetical protein	6.62
Mtr.49246.1.S1_at	Zn-finger, MYND type	6.62
Mtr.35662.1.S1_at	Hypothetical protein	6.62
Mtr.22599.1.S1_at	Histidine decarboxylase	6.60
Mtr.17467.1.S1_at	Serine/threonine protein kinase	6.58
Mtr.26988.1.S1_at	Hypothetical protein	6.57
Mtr.32312.1.S1_at	WRKY transcription factor	6.57
Mtr.28554.1.S1_at	Hypothetical protein	6.57
Mtr.27753.1.S1_at	L-lactate dehydrogenase	6.57
Mtr.11712.1.S1_at	NADH dehydrogenase subunit 6	6.56
Mtr.13139.1.S1_at	Carbonate dehydratase-like protein	6.56
Mtr.18037.1.S1_at	Hypothetical protein	6.55
Mtr.9091.1.S1_at	Hypothetical protein	6.54
Mtr.27812.1.S1_at	Hypothetical protein	6.54
Mtr.33188.1.S1_at	Hypothetical protein	6.54
Mtr.13318.1.S1_at	Hypothetical protein	6.53
Mtr.13630.1.S1_at	Membrane spanning protein	6.52
Mtr.14785.1.S1_at	Hypothetical protein	6.51
Mtr.49053.1.S1_s_at	Esterase/lipase/thioesterase	6.51
Msa.1241.1.S1_at	Hypothetical protein	6.50
Mtr.22444.1.S1_at	Calcium-binding EF-hand family protein	6.50
Mtr.4702.1.S1_s_at	Fiber protein Fb1	6.49
Mtr.29535.1.S1_at	Hypothetical protein	6.49
Mtr.14950.1.S1_s_at	Hypothetical protein	6.48
Mtr.35122.1.S1_at	Hypothetical protein	6.45
Mtr.51646.1.S1_at	Carbohydrate-binding protein	6.44
Mtr.44270.1.S1_at	Homocysteine S-methyltransferase 3	6.42
Mtr.40782.1.S1_at	Hypothetical protein	6.41
Mtr.40765.1.S1_at	Hypothetical protein	6.41
Msa.996.1.S1_at	Hypothetical protein	6.39
Mtr.35204.1.S1_at	Glycine-rich protein	6.36
Mtr.40256.1.S1_at	Hypothetical protein	6.36
Mtr.33678.1.S1_at	Hypothetical protein	6.35
Mtr.2627.1.S1_at	Hypothetical protein	6.35
Mtr.49481.1.S1_at	Hypothetical protein	6.34
Mtr.43956.1.S1_at	Zinc finger family protein	6.33
Msa.1875.1.S1_at	Hypothetical protein	6.32
Mtr.16851.1.S1_at	Serine/threonine protein kinase	6.32
Mtr.261.1.S1_at	Hypothetical protein	6.31

Mtr.12419.1.S1_s_at	50S ribosomal protein L15	6.30
Mtr.44843.1.S1_at	Hypothetical protein	6.30
Mtr.13643.1.S1_at	Hypothetical protein	6.30
Mtr.28498.1.S1_at	S-ribonuclease binding protein	6.28
Mtr.40005.1.S1_at	Hypothetical protein	6.27
Mtr.45833.1.S1_s_at	Hypothetical protein	6.27
Mtr.35960.1.S1_at	1, 3-beta-glucanase	6.27
Mtr.38547.1.S1_at	Tuber-specific and sucrose-responsive element binding factor	6.25
Mtr.12512.1.S1_at	Glutathione S-transferase GST 24	6.24
Mtr.38711.1.S1_at	GT-1 like transcription factor	6.23
Mtr.9034.1.S1_at	Hypothetical protein	6.22
Mtr.45270.1.S1_at	Hypothetical protein	6.22
Mtr.11502.1.S1_at	Hypothetical protein	6.22
Mtr.36679.1.S1_s_at	Hypothetical protein	6.20
Mtr.44996.1.S1_at	Acid phosphatase	6.19
Msa.1414.1.S1_at	Hypothetical protein	6.19
Msa.2935.1.S1_at	Hypothetical protein	6.18
Mtr.31312.1.S1_at	Receptor ser/thr protein kinase	6.18
Mtr.26304.1.S1_at	Hypothetical protein	6.18
Mtr.37277.1.S1_at	Calcium-dependent protein kinase	6.17
Mtr.31273.1.S1_at	Hypothetical protein	6.17
Mtr.37284.1.S1_at	Topoisomerase-like protein	6.17
Mtr.24000.1.S1_at	PPR repeat-containing protein	6.17
Mtr.6373.1.S1_at	Acetyl-CoA carboxylase	6.16
Mtr.4083.1.S1_at	Hypothetical protein	6.16
Mtr.43987.1.S1_at	Serin carboxypeptidase-like protein	6.16
Mtr.25168.1.S1_at	Leucine zipper-containing protein	6.15
Mtr.40982.1.S1_at	Yippee-like protein	6.14
Mtr.31327.1.S1_s_at	Protein kinase	6.14
Mtr.42340.1.S1_at	Importin alpha 2	6.14
Mtr.5270.1.S1_at	Hypothetical protein	6.13
Mtr.18602.1.S1_at	Pathogenesis-related transcriptional factor	6.12
Mtr.38258.1.S1_at	Peroxisomal acetoacetyl-coenzyme A thiolase	6.12
Mtr.11530.1.S1_at	Hypothetical protein	6.12
Mtr.2340.1.S1_s_at	Hypothetical protein	6.12
Mtr.42770.1.S1_at	Photosystem II 23 kDa polypeptide	6.11
Mtr.20185.1.S1_at	Naringenin-chalcone synthase	6.11
Mtr.31290.1.S1_at	NHL repeat-containing protein	6.11
Mtr.33360.1.S1_at	Hypothetical protein	6.11
Mtr.12225.1.S1_at	Plastidic ATP/ADP transporter	6.10
Mtr.46756.1.S1_s_at	Hypothetical protein	6.09
Mtr.6637.1.S1_s_at	Hypothetical protein	6.09
Mtr.40751.1.S1_at	Hypothetical protein	6.09
Mtr.13087.1.S1_at	Hypothetical protein	6.09
Mtr.12290.1.S1_s_at	Proline dehydrogenase	6.09
Mtr.13605.1.S1_at	Hypothetical protein	6.08
Mtr.29063.1.S1_at	Hypothetical protein	6.08
Mtr.12412.1.S1_at	Glutamate dehydrogenase 1	6.07
Mtr.7658.1.S1_at	Hypothetical protein	6.06
Mtr.41386.1.S1_at	Hypothetical protein	6.05
Mtr.40821.1.S1_at	Abscisic acid-activated protein kinase	6.05
Mtr.32799.1.S1_at	GTP-binding regulatory protein	6.05
Mtr.44393.1.S1_at	Transformer-SR ribonucleoprotein	6.05
Mtr.17422.1.S1_at	Amino acid/polyamine transporter I	6.04

Mtr.27551.1.S1_at	LOB domain protein 38	6.04
Mtr.10581.1.S1_at	Hypothetical protein	6.03
Mtr.42389.1.S1_at	Hypothetical protein	6.03
Mtr.40320.1.S1_at	Hypothetical protein	6.03
Mtr.33391.1.S1_at	Glycine-rich RNA binding protein	6.01
Mtr.23204.1.S1_x_at	Hypothetical protein	6.00
Mtr.50912.1.S1_s_at	Hypothetical protein	5.99
Mtr.8995.1.S1_at	Hypothetical protein	5.99
Msa.896.1.S1_at	Hypothetical protein	5.99
Mtr.11354.1.S1_at	Bacterial-induced peroxidase	5.98
Mtr.42039.1.S1_at	Hypothetical protein	5.97
Mtr.47936.1.S1_at	Hypothetical protein	5.97
Mtr.32211.1.S1_at	Asparagine synthetase	5.96
Mtr.13407.1.S1_at	Hypothetical protein	5.96
Mtr.7440.1.S1_at	Hypothetical protein	5.94
Mtr.44257.1.S1_s_at	Hypothetical protein	5.94
Mtr.18642.1.S1_at	Hypothetical protein	5.94
Mtr.11333.1.S1_at	Oxidase-like protein	5.94
Mtr.44658.1.S1_at	Hypothetical protein	5.94
Mtr.25841.1.S1_at	Plasma intrinsic protein	5.93
Mtr.13259.1.S1_at	Methylmalonate semi-aldehyde dehydrogenase	5.93
Mtr.38185.1.S1_at	Hypothetical protein	5.92
Mtr.41367.1.S1_at	Hypothetical protein	5.92
Mtr.22601.1.S1_at	Endoxyloglucan transferase	5.91
Mtr.20185.1.S1_x_at	Naringenin-chalcone synthase	5.91
Mtr.3345.1.S1_at	Hypothetical protein	5.91
Mtr.41961.1.S1_at	Hypothetical protein	5.90
Mtr.4870.1.S1_s_at	Hypothetical protein	5.90
Mtr.35009.1.S1_at	Hypothetical protein	5.89
Mtr.10165.1.S1_at	Ethylene receptor	5.89
Mtr.27950.1.S1_at	Hypothetical protein	5.89
Mtr.28325.1.S1_at	Branched-chain alpha-keto acid decarboxylase E1 beta subunit	5.88
Msa.2746.1.S1_at	Hypothetical protein	5.87
Mtr.21729.1.S1_at	Serine/threonine protein kinase	5.87
Mtr.30703.1.S1_at	Hypothetical protein	5.86
Mtr.12946.1.S1_at	Zinc finger (C3HC4-type RING finger) family protein	5.86
Mtr.46085.1.S1_at	Homeobox protein	5.86
Mtr.38662.1.S1_at	Hypothetical protein	5.85
Mtr.16703.1.S1_at	Disease resistance protein	5.85
Mtr.43971.1.S1_at	Hypothetical protein	5.85
Mtr.39919.1.S1_at	Protein kinase	5.85
Mtr.35045.1.S1_s_at	Rab escort protein	5.84
Mtr.37736.1.S1_at	Hypothetical protein	5.83
Mtr.10043.1.S1_at	Wax synthase	5.83
Mtr.22708.1.S1_at	Uridylyltransferase	5.82
Mtr.38092.1.S1_at	MAP kinase phosphatase	5.82
Mtr.50494.1.S1_s_at	Hypothetical protein	5.82
Mtr.43845.1.S1_at	Hypothetical protein	5.82
Mtr.18067.1.S1_at	O-methyltransferase	5.81
Mtr.33339.1.S1_at	Hypothetical protein	5.81
Mtr.31018.1.S1_at	Hypothetical protein	5.81
Mtr.9569.1.S1_at	Thaumatin-like protein 1	5.80
Mtr.7058.1.S1_at	Hypothetical protein	5.80
Mtr.38811.1.S1_s_at	Nucleosome assembly protein 1-like protein 4	5.80

Mtr.4284.1.S1_at	Hypothetical protein	5.80
Mtr.29602.1.S1_at	Hypothetical protein	5.78
Mtr.43170.1.S1_s_at	Glutamate dehydrogenase 1	5.78
Mtr.43272.1.S1_at	Hypothetical protein	5.77
Mtr.2282.1.S1_at	Hypothetical protein	5.77
Mtr.11713.1.S1_at	BRASSINOSTEROID INSENSITIVE 1-associated receptor kinase 1	5.77
Mtr.43705.1.S1_at	Light repressible receptor protein kinase	5.74
Mtr.29666.1.S1_at	Hypothetical protein	5.74
Mtr.11080.1.S1_s_at	Glutathione S-transferase	5.73
Mtr.8646.1.S1_at	Nine-cis-epoxycarotenoid dioxygenase1	5.73
Mtr.50704.1.S1_s_at	Aldehyde dehydrogenase	5.73
Mtr.11986.1.S1_at	WD40-repeat protein	5.73
Mtr.12209.1.S1_at	Phi-1 protein	5.73
Mtr.44816.1.S1_at	Hypothetical protein	5.73
Mtr.52317.1.S1_at	Hypothetical protein	5.72
Mtr.35520.1.S1_at	Hypothetical protein	5.72
Mtr.15448.1.S1_at	Gibberellin regulated protein	5.72
Msa.1311.1.S1_at	Hypothetical protein	5.72
Mtr.9098.1.S1_at	CREG2-protein	5.71
Mtr.37301.1.S1_at	Cytochrome P450	5.70
Mtr.39874.1.S1_at	Hypothetical protein	5.70
Mtr.16145.1.S1_s_at	Serine/threonine protein kinase	5.69
Mtr.51630.1.S1_s_at	Hypothetical protein	5.69
Msa.1569.1.S1_at	Hypothetical protein	5.68
Mtr.13823.1.S1_at	Hypothetical protein	5.68
Mtr.13094.1.S1_at	GTP-binding protein	5.67
Mtr.42757.1.S1_at	Hypothetical protein	5.67
Mtr.31998.1.S1_at	Epoxide hydrolase	5.66
Mtr.10773.1.S1_at	Wasl protein	5.66
Mtr.35231.1.S1_at	Pathogenesis-related protein	5.66
Mtr.32690.1.S1_at	Nucleolar autoantigen-like protein	5.66
Mtr.36136.1.S1_at	Hypothetical protein	5.65
Mtr.10619.1.S1_at	Hypothetical protein	5.65
Mtr.33372.1.S1_at	Hypothetical protein	5.64
Mtr.44882.1.S1_at	Hypothetical protein	5.64
Mtr.14016.1.S1_at	Hypothetical protein	5.64
Mtr.16688.1.S1_at	Mitochondrial import inner membrane translocase	5.63
Mtr.43938.1.S1_at	Protein kinase	5.63
Mtr.2541.1.S1_at	RuvB DNA helicase	5.63
Mtr.35357.1.S1_at	Auxin-regulated gene	5.63
Mtr.47086.1.S1_s_at	Glucan 1,3-beta-glucosidase	5.62
Mtr.38375.1.S1_at	Hypothetical protein	5.62
Mtr.46410.1.S1_at	Hypothetical protein	5.62
Mtr.20704.1.S1_at	Nucleosome assembly protein	5.61
Mtr.22597.1.S1_s_at	Serine decarboxylase	5.61
Mtr.930.1.S1_at	Hypothetical protein	5.61
Mtr.21463.1.S1_at	Hypothetical protein	5.60
Mtr.43184.1.S1_at	Hypothetical protein	5.59
Mtr.18228.1.S1_at	Gibberellin regulated protein	5.59
Mtr.9522.1.S1_at	Hypothetical protein	5.58
Mtr.9486.1.S1_at	Zinc finger protein CONSTANS-LIKE 10	5.58
Mtr.5517.1.S1_at	Hypothetical protein	5.57
Mtr.39558.1.S1_at	Gamma-gliadin precursor	5.57
Mtr.51889.1.S1_s_at	Peptidase	5.57

Mtr.11357.1.S1_at	SNARE-interacting protein KEULE	5.56
Mtr.39751.1.S1_at	SRG1-like protein	5.56
Mtr.41599.1.S1_s_at	SNARE-interacting protein KEULE	5.56
Mtr.24530.1.S1_s_at	Amino acid transporter	5.56
Mtr.44519.1.S1_at	Hypothetical protein	5.55
Mtr.42984.1.S1_at	Proline dehydrogenase	5.55
Mtr.38520.1.S1_s_at	Hypothetical protein	5.55
Mtr.37208.1.S1_at	Hypothetical protein	5.55
Msa.2921.1.S1_at	Hypothetical protein	5.55
Mtr.34860.1.S1_s_at	Ubiquitin-protein ligase	5.54
Mtr.13641.1.S1_at	Alpha-mannosidase	5.54
Mtr.44228.1.S1_at	Cyanophycinase	5.54
Msa.3094.1.S1_at	Hypothetical protein	5.53
Mtr.5976.1.S1_at	ERD1 protein	5.52
Mtr.33096.1.S1_at	Heterogeneous nuclear ribonucleoprotein	5.52
Mtr.49473.1.S1_at	Aldehyde dehydrogenase	5.51
Mtr.16634.1.S1_s_at	Hypothetical protein	5.50
Mtr.24464.1.S1_at	Receptor-like protein kinase	5.50
Mtr.44587.1.S1_at	Peroxisomal acetoacetyl-coenzyme A thiolase	5.50
Mtr.37501.1.S1_at	Isoliquiritigenin 2'-O-methyltransferase	5.49
Mtr.11544.1.S1_at	Receptor-like kinase	5.49
Mtr.32566.1.S1_s_at	DEAD box protein	5.49
Mtr.20751.1.S1_at	bZIP transcription factor	5.48
Mtr.33428.1.S1_at	Hypothetical protein	5.48
Mtr.42907.1.S1_at	Hypothetical protein	5.47
Mtr.30257.1.S1_at	Hypothetical protein	5.47
Msa.3040.1.S1_at	Hypothetical protein	5.47
Mtr.42269.1.S1_at	Transcription factor (E2F)	5.46
Mtr.38566.1.S1_at	Hypothetical protein	5.46
Mtr.1851.1.S1_s_at	Secreted glycoprotein 3	5.46
Mtr.39065.1.S1_at	Hypothetical protein	5.45
Mtr.8407.1.S1_at	Hypothetical protein	5.45
Mtr.41839.1.S1_at	Hypothetical protein	5.45
Mtr.9901.1.S1_at	Receptor-like kinase	5.45
Mtr.7373.1.S1_at	Hypothetical protein	5.45
Mtr.12538.1.S1_at	Hypothetical protein	5.44
Mtr.15339.1.S1_at	Homeodomain-like proein	5.44
Mtr.12609.1.S1_at	Acyl CoA oxidase	5.43
Mtr.40002.1.S1_at	Hydroxymethylglutaryl-CoA lyase	5.43
Mtr.12456.1.S1_at	AP2-related transcription factor	5.43
Mtr.51562.1.S1_at	No apical meristem (NAM) protein	5.42
Mtr.10147.1.S1_at	Auxin-induced SAUR-like protein	5.42
Msa.1621.1.S1_at	Hypothetical protein	5.42
Mtr.30853.1.S1_at	Sugar transporter	5.41
Mtr.25254.1.S1_at	DnaJ-like protein	5.41
Mtr.19347.1.S1_at	Major intrinsic protein	5.40
Mtr.13769.1.S1_at	Exonuclease	5.40
Mtr.9041.1.S1_s_at	Hypothetical protein	5.40
Mtr.40284.1.S1_at	Hypothetical protein	5.40
Mtr.6811.1.S1_at	Glutathione S-transferase GST 17	5.39
Mtr.13682.1.S1_at	Alpha-mannosidase	5.39
Mtr.25966.1.S1_at	Hypothetical protein	5.39
Mtr.51296.1.S1_at	SPla/RYanodine receptor	5.38
Mtr.40203.1.S1_at	Aldehyde dehydrogenase (NAD+)	5.38

Mtr.27318.1.S1_at	Fructose-bisphosphate aldolase	5.37
Mtr.6545.1.S1_at	Hypothetical protein	5.37
Mtr.32188.1.S1_x_at	Chalcone synthase 9	5.37
Mtr.48688.1.S1_at	DNA-binding WRKY	5.37
Mtr.13223.1.S1_at	Alpha-mannosidase	5.36
Mtr.48718.1.S1_at	Galactose-binding protein	5.36
Mtr.13645.1.S1_at	Hypothetical protein	5.36
Mtr.10805.1.S1_at	Avr9/Cf-9 rapidly elicited protein	5.35
Mtr.20378.1.S1_at	Sugar transporter	5.35
Mtr.42600.1.S1_at	Hypothetical protein	5.35
Mtr.48353.1.S1_at	Alpha fucosidase	5.35
Mtr.43706.1.S1_at	Hypothetical protein	5.35
Mtr.43917.1.S1_at	Hypothetical protein	5.35
Mtr.7313.1.S1_at	50S ribosomal protein L3-2	5.35
Mtr.37169.1.S1_at	Hypothetical protein	5.34
Mtr.34217.1.S1_at	Ss-galactosidase precursor	5.34
Mtr.43784.1.S1_at	Hypothetical protein	5.34
Msa.2542.1.S1_at	Hypothetical protein	5.34
Mtr.5840.1.S1_at	Hypothetical protein	5.33
Mtr.18295.1.S1_at	Zinc-containing alcohol dehydrogenase	5.33
Msa.1009.1.S1_at	Hypothetical protein	5.33
Mtr.3156.1.S1_at	Hypothetical protein	5.32
Mtr.12470.1.S1_at	Syringolide-induced protein	5.32
Msa.937.1.S1_at	Hypothetical protein	5.31
Mtr.30738.1.S1_at	Uracil phosphoribosyltransferase 1	5.31
Mtr.10703.1.S1_at	Kinesin-like protein KIF13B	5.31
Mtr.29510.1.S1_at	Hypothetical protein	5.31
Mtr.17966.1.S1_at	Leucine-rich repeat protein	5.31
Mtr.11572.1.S1_at	Receptor-like protein kinase	5.31
Mtr.51042.1.S1_at	Hypothetical protein	5.31
Mtr.9214.1.S1_at	Hypothetical protein	5.30
Mtr.44713.1.S1_at	Amino acid transporter-like protein	5.30
Mtr.9055.1.S1_at	Branched-chain alpha-keto acid decarboxylase E1	5.30
Mtr.42023.1.S1_at	Lipase SIL1	5.30
Mtr.42185.1.S1_at	Hypothetical protein	5.30
Mtr.44573.1.S1_at	GPI-anchored protein	5.30
Mtr.41983.1.S1_at	Glucosyltransferase-7	5.29
Mtr.10514.1.S1_at	Acyl-activating enzyme	5.29
Mtr.45031.1.S1_at	Proteinase	5.29
Mtr.6156.1.S1_at	Cytochrome oxidase subunit II	5.29
Mtr.26629.1.S1_at	CCR4-associated factor 1-related protein	5.29
Mtr.21708.1.S1_at	lysophospholipase	5.29
Mtr.38635.1.S1_at	Peroxidase 10	5.28
Mtr.13033.1.S1_at	Hypothetical protein	5.28
Mtr.9032.1.S1_at	Aspartyl protease	5.28
Msa.1202.1.S1_at	Hypothetical protein	5.28
Mtr.52124.1.S1_s_at	Phosphate-induced protein 1	5.27
Mtr.29714.1.S1_at	Hypothetical protein	5.26
Mtr.38945.1.S1_at	Hypothetical protein	5.26
Mtr.6352.1.S1_s_at	Alternative oxidase 3	5.25
Mtr.42223.1.S1_at	Hypothetical protein	5.25
Msa.3172.1.S1_at	Hypothetical protein	5.25
Mtr.24341.1.S1_at	Hypothetical protein	5.25
Mtr.43229.1.S1_at	Syringolide-induced protein 14-1-1	5.25

Mtr.37224.1.S1_at	Hydroxyproline-rich glycoprotein	5.25
Mtr.45563.1.S1_at	PDR9 ABC transporter	5.24
Msa.820.1.S1_at	Hypothetical protein	5.24
Mtr.11512.1.S1_at	Hypothetical protein	5.24
Mtr.43707.1.S1_at	Hypothetical protein	5.24
Mtr.33785.1.S1_at	Hypothetical protein	5.24
Mtr.5950.1.S1_s_at	Zinc finger protein	5.24
Mtr.12652.1.S1_s_at	Hypothetical protein	5.23
Mtr.21035.1.S1_at	Sugar transporter	5.23
Mtr.43346.1.S1_at	Nodulin-like protein	5.23
Mtr.34775.1.S1_at	Hypothetical protein	5.22
Mtr.41503.1.S1_at	Hypothetical protein	5.21
Mtr.11219.1.S1_at	Hypothetical protein	5.21
Mtr.32564.1.S1_at	Hypothetical protein	5.21
Mtr.9106.1.S1_at	RuvB DNA helicase	5.20
Mtr.17135.1.S1_at	ATPase, E1-E2 type	5.20
Mtr.14439.1.S1_at	Hypothetical protein	5.19
Mtr.48475.1.S1_at	Growth regulator like protein	5.19
Mtr.34121.1.S1_at	Hypothetical protein	5.19
Mtr.9031.1.S1_at	Nucellin-like aspartic protease	5.18
Mtr.40627.1.S1_at	Hypothetical protein	5.18
Mtr.45053.1.S1_at	Hypothetical protein	5.17
Mtr.10388.1.S1_at	UDP-glucose 4-epimerase	5.17
Msa.1505.1.S1_at	Hypothetical protein	5.17
Mtr.39454.1.S1_at	Enod8-like protein	5.16
Mtr.49418.1.S1_s_at	Hypothetical protein	5.16
Mtr.21016.1.S1_at	Hypothetical protein	5.16
Mtr.7966.1.S1_at	Hypothetical protein	5.15
Mtr.35681.1.S1_at	Metal-dependent amidase/aminoacylase/carboxypeptidase	5.15
Mtr.2218.1.S1_at	Ubiquitin-protein ligase	5.14
Mtr.18117.1.S1_at	Hypothetical protein	5.13
Mtr.5932.1.S1_at	Hypothetical protein	5.13
Mtr.40239.1.S1_at	Phosphoenolpyruvate carboxykinase	5.13
Mtr.7011.1.S1_at	Hypothetical protein	5.13
Mtr.44444.1.S1_at	MADS box interactor	5.13
Mtr.23414.1.S1_at	Hypothetical protein	5.13
Mtr.17255.1.S1_at	Formyl transferase	5.12
Mtr.41441.1.S1_at	Hypothetical protein	5.12
Mtr.50038.1.S1_at	No apical meristem (NAM) protein	5.12
Mtr.12703.1.S1_at	Hypothetical protein	5.12
Mtr.27180.1.S1_at	Ntdin	5.11
Mtr.10005.1.S1_at	Hypothetical protein	5.11
Mtr.2502.1.S1_s_at	Hypothetical protein	5.11
Mtr.11704.1.S1_at	Protein phosphatase 2C	5.11
Mtr.29342.1.S1_at	Hypothetical protein	5.10
Mtr.10063.1.S1_at	Cycloidea protein	5.10
Mtr.1800.1.S1_at	Cytochrome b	5.10
Mtr.40005.1.S1_s_at	Hypothetical protein	5.09
Mtr.15180.1.S1_at	Glucose-6-phosphate dehydrogenase	5.09
Mtr.28045.1.S1_at	Hypothetical protein	5.09
Mtr.82.1.S1_at	STS14 protein	5.08
Mtr.44073.1.S1_at	En/Spm-like transposon protein	5.08
Mtr.19347.1.S1_s_at	Major intrinsic protein	5.08
Mtr.1526.1.S1_a_at	Hypothetical protein	5.08

Mtr.21501.1.S1_at	Zn-finger, RING	5.08
Mtr.15228.1.S1_s_at	Pre-mRNA processing ribonucleoprotein	5.08
Mtr.43343.1.S1_at	Hypothetical protein	5.08
Mtr.36359.1.S1_s_at	Hypothetical protein	5.08
Mtr.40917.1.S1_at	SPF1 protein	5.07
Mtr.2604.1.S1_x_at	Nodulin	5.07
Mtr.43391.1.S1_at	Hypothetical protein	5.06
Mtr.41551.1.S1_at	SCARECROW-like protein	5.06
Msa.2722.1.S1_at	Hypothetical protein	5.06
Mtr.38570.1.S1_at	YGL010w-like protein	5.06
Mtr.25922.1.S1_at	Hypothetical protein	5.05
Mtr.34967.1.S1_at	ERD1 protein	5.04
Mtr.40789.1.S1_at	DNA-binding protein	5.04
Mtr.40168.1.S1_at	Phenylalanine ammonia-lyase 2	5.03
Mtr.45088.1.S1_at	Vesicle transport v-SNARE 13	5.02
Mtr.32566.1.S1_at	DEAD box protein	5.02
Mtr.10930.1.S1_at	Caffeoyl-CoA O-methyltransferase 5	5.01
Mtr.37779.1.S1_at	Formate dehydrogenase	5.01
Mtr.41029.1.S1_s_at	Ankyrin repeat-containing protein	5.01
Mtr.7014.1.S1_at	Peptide transporter 1	5.00
Mtr.35824.1.S1_at	GATA-1 zinc finger protein	5.00
Mtr.12958.1.S1_at	Hypothetical protein	5.00
Mtr.20288.1.S1_at	Transcriptional factor	5.00
Mtr.40050.1.S1_at	Rab escort protein	4.99
Mtr.45159.1.S1_at	Hypothetical protein	4.99
Mtr.6892.1.S1_at	S glycoprotein	4.98
Mtr.41497.1.S1_at	ARIADNE-like protein	4.97
Mtr.39144.1.S1_at	WD40-repeat protein	4.97
Mtr.1676.1.S1_at	Hypothetical protein	4.95
Mtr.42222.1.S1_at	Hypothetical protein	4.95
Mtr.42418.1.S1_at	Phospholipase D	4.95
Mtr.33541.1.S1_x_at	Asparagine synthase (glutamine-hydrolysing)	4.95
Mtr.26369.1.S1_at	Hypothetical protein	4.94
Mtr.9031.1.S1_s_at	Nucellin-like aspartic protease	4.94
Mtr.7227.1.S1_at	Hypothetical protein	4.94
Mtr.8789.1.S1_s_at	Formate dehydrogenase	4.94
Mtr.10565.1.S1_at	Hypothetical protein	4.93
Mtr.7253.1.S1_at	Snakin-1	4.93
Mtr.12662.1.S1_at	Patatin-like protein 1	4.93
Mtr.48506.1.S1_at	Hypothetical protein	4.93
Mtr.25059.1.S1_at	WD-40 repeat protein	4.93
Mtr.39176.1.S1_at	Hypothetical protein	4.93
Mtr.1952.1.S1_at	Hypothetical protein	4.93
Mtr.40278.1.S1_at	Glutathione S-transferase	4.92
Mtr.9101.1.S1_at	PDR-like ABC transporter	4.92
Mtr.17625.1.S1_at	Zn-finger, RING	4.92
Mtr.16195.1.S1_at	Hypothetical protein	4.91
Mtr.22834.1.S1_at	Symbiotic ammonium transporter	4.91
Mtr.1430.1.S1_at	Hypothetical protein	4.91
Mtr.40547.1.S1_at	bZIP transcription factor	4.91
Mtr.47187.1.S1_at	Glyoxysomal fatty acid beta-oxidation multifunctional protein	4.90
Mtr.14250.1.S1_at	Arf GTPase activating protein	4.90
Mtr.13273.1.S1_at	TINY-like protein	4.90
Mtr.49791.1.S1_at	Auxin responsive SAUR protein	4.90

Mtr.24888.1.S1_at	Hypothetical protein	4.90
Mtr.29620.1.S1_at	Hypothetical protein	4.89
Mtr.47932.1.S1_at	Xylulose kinase	4.89
Mtr.27992.1.S1_at	Hypothetical protein	4.88
Mtr.13270.1.S1_at	Hypothetical protein	4.88
Mtr.45741.1.S1_at	Cytochrome P450	4.88
Mtr.16691.1.S1_at	Myb, DNA-binding protein	4.88
Mtr.35927.1.S1_at	Maturase K	4.87
Mtr.27097.1.S1_at	Hypothetical protein	4.87
Mtr.42872.1.S1_at	Hypothetical protein	4.87
Mtr.39139.1.S1_at	Pathogenesis-related protein 4A	4.87
Mtr.42610.1.S1_at	Acyl-activating enzyme 17	4.87
Mtr.11677.1.S1_at	ERD1 protein	4.87
Mtr.28295.1.S1_s_at	Female-specific transformer protein	4.87
Mtr.9440.1.S1_at	Hypothetical protein	4.86
Mtr.43134.1.S1_at	Fructose-bisphosphate aldolase-like protein	4.86
Msa.2848.1.S1_at	Hypothetical protein	4.86
Mtr.44386.1.S1_at	Hypothetical protein	4.86
Mtr.31327.1.S1_at	Protein kinase	4.85
Mtr.20008.1.S1_at	Ankyrin Repeat Protein	4.85
Mtr.13110.1.S1_at	Fiber protein Fb2	4.84
Mtr.2465.1.S1_at	Hypothetical protein	4.84
Mtr.45583.1.S1_at	Hypothetical protein	4.84
Mtr.37425.1.S1_at	Transcription factor WRKY10	4.84
Mtr.37928.1.S1_at	Thiamin pyrophosphokinase 1	4.84
Mtr.43834.1.S1_at	DEAD-Box RNA helicase-like protein	4.84
Msa.1103.1.S1_at	Hypothetical protein	4.83
Mtr.17513.1.S1_at	bZIP transcription factor	4.83
Msa.2554.1.S1_at	Hypothetical protein	4.83
Mtr.42795.1.S1_at	Glutamate synthase [NADH]	4.83
Mtr.10416.1.S1_at	3-deoxy-D-arabino-heptulosonate 7-phosphate synthase	4.83
Mtr.31944.1.S1_at	Hypothetical protein	4.83
Mtr.28526.1.S1_at	Hypothetical protein	4.83
Mtr.233.1.S1_at	Patatin-related protein	4.82
Mtr.7448.1.S1_s_at	Hypothetical protein	4.82
Mtr.38097.1.S1_at	Hypothetical protein	4.82
Mtr.35968.1.S1_at	UDP-glucosyltransferase	4.81
Mtr.33601.1.S1_s_at	Nodulin26-like major intrinsic protein	4.81
Mtr.14627.1.S1_at	Hypothetical protein	4.81
Mtr.14281.1.S1_at	Hypothetical protein	4.81
Mtr.44727.1.S1_at	Timing of CAB expression 1	4.80
Mtr.9417.1.S1_at	Beta-galactosidase	4.79
Mtr.45463.1.S1_at	Xyloglucan endotransglucosylase/hydrolase	4.78
Mtr.39092.1.S1_at	Cytochrome oxidase subunit III	4.78
Mtr.44597.1.S1_at	Female-specific transformer protein	4.78
Mtr.25525.1.S1_at	Hypothetical protein	4.78
Mtr.40433.1.S1_at	Hypothetical protein	4.78
Mtr.16824.1.S1_at	Heavy metal transport/detoxification protein	4.78
Mtr.49791.1.S1_x_at	Auxin responsive SAUR protein	4.77
Mtr.1343.1.S1_at	Hypothetical protein	4.77
Msa.2952.1.S1_at	Hypothetical protein	4.76
Mtr.17517.1.S1_at	Hypothetical protein	4.76
Mtr.24465.1.S1_at	Receptor-like protein kinase	4.76
Mtr.12018.1.S1_at	Hypothetical protein	4.76

Mtr.16783.1.S1_at	Hypothetical protein	4.75
Mtr.36311.1.S1_at	Hypothetical protein	4.75
Mtr.38129.1.S1_at	RNA-binding protein	4.75
Mtr.44417.1.S1_at	Disease resistance protein	4.74
Mtr.15278.1.S1_s_at	Zn-finger, C2H2 type	4.74
Mtr.37771.1.S1_at	Ser/Thr protein kinase	4.74
Mtr.16301.1.S1_at	Hypothetical protein	4.74
Mtr.48476.1.S1_at	Hypothetical protein	4.74
Mtr.10817.1.S1_at	Hypothetical protein	4.74
Mtr.1955.1.S1_at	Protein phosphatase 2C	4.73
Mtr.43499.1.S1_at	Hypothetical protein	4.73
Mtr.10628.1.S1_at	Aldehyde 5-hydroxylase	4.73
Mtr.41861.1.S1_at	Acetyl-CoA carboxylase	4.72
Mtr.38368.1.S1_at	Hypothetical protein	4.72
Mtr.8669.1.S1_at	Hypothetical protein	4.72
Mtr.5877.1.S1_at	Thioredoxin 2	4.72
Mtr.10798.1.S1_at	Hypothetical protein	4.72
Mtr.49047.1.S1_at	Glycoside hydrolase	4.71
Mtr.13491.1.S1_at	Beta xylosidase	4.71
Mtr.41182.1.S1_at	GTP-binding protein-like (Ras-related GTP-binding protein)	4.71
Mtr.40058.1.S1_x_at	Nodulin	4.70
Mtr.27194.1.S1_at	Hypothetical protein	4.69
Mtr.41299.1.S1_s_at	Hypothetical protein	4.69
Mtr.8701.1.S1_at	Leucine rich repeat protein	4.69
Mtr.11112.1.S1_at	Hypothetical protein	4.69
Msa.2665.1.S1_at	Hypothetical protein	4.68
Mtr.34901.1.S1_s_at	PDR-like ABC transporter	4.68
Mtr.18646.1.S1_at	Hypothetical protein	4.68
Mtr.7638.1.S1_at	Endo-1,3-beta-glucanase	4.68
Mtr.21466.1.S1_at	Serine/threonine protein kinase	4.68
Mtr.37996.1.S1_at	Serine/threonine-specific receptor protein kinase	4.67
Mtr.12123.1.S1_at	Hypothetical protein	4.67
Mtr.21119.1.S1_at	Phytochrome kinase	4.67
Mtr.40272.1.S1_at	Nodulin-like protein	4.67
Mtr.10959.1.S1_at	Nodulin-like protein	4.67
Mtr.12835.1.S1_at	Hypothetical protein	4.66
Mtr.34018.1.S1_at	S-receptor kinase	4.66
Mtr.37913.1.S1_at	Acyl-CoA oxidase	4.66
Mtr.13391.1.S1_at	Hypothetical protein	4.64
Mtr.22558.1.S1_s_at	Hydroxymethylglutaryl-CoA lyase	4.64
Mtr.41607.1.S1_at	Hypothetical protein	4.64
Msa.2783.1.S1_at	Hypothetical protein	4.64
Mtr.51656.1.S1_at	Ubiquitin-associated protein	4.63
Mtr.17903.1.S1_at	Hypothetical protein	4.63
Mtr.6178.1.S1_at	Alanine racemase	4.62
Mtr.51564.1.S1_at	Galactose-binding protein	4.62
Mtr.3183.1.S1_at	Hypothetical protein	4.62
Msa.2215.1.S1_at	Hypothetical protein	4.62
Mtr.10974.1.S1_at	Hypothetical protein	4.62
Mtr.16912.1.S1_at	WD-40 repeat	4.62
Mtr.11550.1.S1_at	Diacylglycerol kinase	4.62
Mtr.11633.1.S1_at	Transducin-like protein	4.62
Mtr.8936.1.S1_at	Zinc finger protein	4.62
Mtr.9577.1.S1_at	Hypothetical protein	4.61

Mtr.20630.1.S1_at	Glycoside hydrolase	4.61
Mtr.34632.1.S1_s_at	Seven transmembrane helix receptor	4.61
Mtr.37677.1.S1_s_at	Hypothetical protein	4.61
Mtr.41977.1.S1_at	Hypothetical protein	4.60
Mtr.12525.1.S1_at	Chitinase	4.60
Mtr.11287.1.S1_at	Hypothetical protein	4.60
Mtr.16728.1.S1_at	Fibrillarin	4.60
Mtr.15054.1.S1_at	Thaumatin	4.60
Msa.2884.1.S1_at	Hypothetical protein	4.60
Mtr.27919.1.S1_at	Hypothetical protein	4.60
Mtr.41745.1.S1_at	General negative transcription regulator	4.60
Mtr.44169.1.S1_at	Spermatid-specific protein T1	4.60
Mtr.38993.1.S1_at	ERD1 protein	4.59
Mtr.32458.1.S1_at	Hypothetical protein	4.59
Mtr.10976.1.S1_at	WRKY transcription factor	4.59
Mtr.15850.1.S1_at	Glycine-rich RNA binding protein	4.59
Mtr.42215.1.S1_at	Hypothetical protein	4.59
Mtr.52198.1.S1_at	Bacterial regulatory factor	4.58
Mtr.33658.1.S1_at	Hypothetical protein	4.58
Mtr.12051.1.S1_at	Hypothetical protein	4.57
Mtr.8674.1.S1_at	Serine/threonine protein phosphatase PP1	4.57
Msa.1485.1.S1_at	Hypothetical protein	4.57
Mtr.40110.1.S1_at	Ribosomal protein S27	4.56
Mtr.50547.1.S1_at	RHO protein GDP dissociation inhibitor	4.56
Mtr.44843.1.S1_s_at	Hypothetical protein	4.55
Mtr.38894.1.S1_s_at	Hypothetical protein	4.55
Mtr.45221.1.S1_at	Hypothetical protein	4.55
Mtr.29044.1.S1_at	Hypothetical protein	4.55
Mtr.39305.1.S1_at	Heat shock transcription factor 34	4.54
Mtr.457.1.S1_s_at	Hypothetical protein	4.54
Mtr.2673.1.S1_s_at	Hypothetical protein	4.54
Mtr.13646.1.S1_at	Hypothetical protein	4.54
Msa.1821.1.S1_at	Hypothetical protein	4.54
Mtr.39403.1.S1_at	Receptor-like serine/threonine kinase	4.53
Mtr.10389.1.S1_s_at	UDP-glucose 4-epimerase	4.53
Mtr.47087.1.S1_at	Glucan 1,3-beta-glucosidase	4.53
Mtr.27396.1.S1_at	Isoliquiritigenin 2'-O-methyltransferase	4.53
Mtr.46097.1.S1_at	Hypothetical protein	4.53
Mtr.1018.1.S1_s_at	GDP-mannose transporter	4.53
Mtr.31455.1.S1_at	Hypothetical protein	4.52
Mtr.28840.1.S1_at	TFIIE-beta	4.52
Mtr.46013.1.S1_at	4Fe-4S ferredoxin, iron-sulfur binding protein	4.52
Mtr.6104.1.S1_at	Short chain alcohol dehydrogenase	4.52
Mtr.42571.1.S1_at	MutT domain protein	4.52
Mtr.1921.1.S1_at	Hypothetical protein	4.51
Mtr.42067.1.S1_s_at	Hypothetical protein	4.51
Mtr.21478.1.S1_s_at	Hypothetical protein	4.51
Mtr.38615.1.S1_at	Hypothetical protein	4.51
Mtr.41789.1.S1_at	Hypothetical protein	4.50
Mtr.10984.1.S1_at	Hypothetical protein	4.50
Mtr.9984.1.S1_at	Acyl-activating enzyme 17	4.50
Mtr.43041.1.S1_at	Hexokinase	4.50
Mtr.43706.1.S1_s_at	Hypothetical protein	4.50
Msa.1940.1.S1_at	Hypothetical protein	4.50

Mtr.24434.1.S1_at	Hypothetical protein	4.49
Mtr.35865.1.S1_at	Cdc2MsC protein	4.48
Mtr.51989.1.S1_s_at	EAG/ELK/ERG potassium channel	4.48
Mtr.6197.1.S1_s_at	Root nodule extensin	4.48
Mtr.12208.1.S1_at	Pentameric polyubiquitin	4.48
Mtr.49405.1.S1_s_at	TIR; Disease resistance protein	4.48
Mtr.16854.1.S1_x_at	Protein kinase	4.48
Mtr.4958.1.S1_at	Hypothetical protein	4.47
Mtr.21905.1.S1_at	Hypothetical protein	4.47
Mtr.19752.1.S1_at	Hypothetical protein	4.47
Mtr.41841.1.S1_at	Hypothetical protein	4.47
Mtr.12817.1.S1_at	Hypothetical protein	4.46
Mtr.1969.1.S1_at	Hypothetical protein	4.46
Mtr.12962.1.S1_at	Rho GDP dissociation inhibitor 2	4.46
Mtr.42171.1.S1_at	Ankyrin repeat-containing protein	4.46
Msa.2671.1.S1_s_at	Hypothetical protein	4.45
Mtr.10807.1.S1_at	Hypothetical protein	4.45
Mtr.44974.1.S1_at	Kinetechore (Skp1p-like) protein	4.45
Mtr.11140.1.S1_at	Protein kinase APK1B	4.45
Mtr.28497.1.S1_at	Hypothetical protein	4.44
Mtr.33595.1.S1_s_at	Root-specific metal transporter	4.44
Mtr.45783.1.S1_at	Short-chain dehydrogenase/reductase SDR	4.44
Mtr.33152.1.S1_at	Hypothetical protein	4.44
Mtr.6341.1.S1_at	Beta-1, 3-glucanase	4.44
Mtr.49872.1.S1_at	Hypothetical protein	4.44
Mtr.47863.1.S1_s_at	Transcription factor WRKY10	4.43
Mtr.32287.1.S1_at	Hypothetical protein	4.43
Mtr.43389.1.S1_at	Fiber protein Fb2	4.43
Mtr.40523.1.S1_at	General negative transcription regulator	4.43
Mtr.12990.1.S1_at	Nucleolar histone deacetylase	4.43
Mtr.45247.1.S1_at	Pre-mRNA splicing factor ATP-dependent RNA helicase	4.43
Mtr.34917.1.S1_at	Hypothetical protein	4.43
Mtr.7503.1.S1_at	Hypothetical protein	4.43
Mtr.37469.1.S1_s_at	Hypothetical protein	4.42
Mtr.46176.1.S1_at	Hypothetical protein	4.42
Mtr.51608.1.S1_at	Plant lipid transfer protein	4.42
Mtr.2945.1.S1_at	Ferredoxin-NADP+ reductase	4.42
Mtr.37730.1.S1_at	Golgi SNARE 11 protein	4.42
Mtr.45351.1.S1_s_at	Hypothetical protein	4.42
Mtr.10176.1.S1_at	Hypothetical protein	4.41
Mtr.11106.1.S1_at	Vernalization 2 protein	4.41
Mtr.25964.1.S1_s_at	Hypothetical protein	4.40
Mtr.8464.1.S1_s_at	Cysteine synthase	4.39
Mtr.50050.1.S1_at	Isopenicillin N synthetase	4.39
Mtr.23261.1.S1_at	Hypothetical protein	4.39
Mtr.1536.1.S1_s_at	Hypothetical protein	4.39
Mtr.40094.1.S1_at	GH3 like protein	4.39
Mtr.13394.1.S1_at	Surfeit 1	4.39
Mtr.28786.1.S1_at	Dihydrodipicolinate synthase	4.39
Mtr.21708.1.S1_s_at	lysophospholipase	4.38
Mtr.35538.1.S1_at	Avr9/Cf-9 rapidly elicited protein	4.38
Mtr.16110.1.S1_at	Ankyrin	4.38
Mtr.41875.1.S1_at	Receptor protein kinase	4.38
Mtr.7135.1.S1_at	Low temperature-responsive RNA-binding protein	4.38

Msa.1800.1.S1_at	Hypothetical protein	4.38
Msa.1598.1.S1_at	Hypothetical protein	4.37
Mtr.42229.1.S1_at	Hypothetical protein	4.37
Mtr.29376.1.S1_at	Hypothetical protein	4.36
Msa.1124.1.S1_at	Hypothetical protein	4.36
Mtr.9542.1.S1_at	SET-domain transcriptional regulator	4.36
Mtr.9496.1.S1_at	Proline rich protein	4.36
Mtr.43362.1.S1_at	Serine/threonine protein phosphatase PP2A	4.36
Msa.1032.1.S1_at	Hypothetical protein	4.35
Mtr.27641.1.S1_at	Hypothetical protein	4.35
Mtr.38801.1.S1_at	TGA-type basic leucine zipper protein TGA1.1	4.35
Mtr.45266.1.S1_at	Phospholipase D	4.35
Mtr.11338.1.S1_at	Hypothetical protein	4.35
Mtr.26632.1.S1_at	Pathogen-related protein	4.34
Mtr.44094.1.S1_at	Hypothetical protein	4.34
Mtr.15525.1.S1_at	Dynein light chain	4.34
Mtr.13495.1.S1_at	Epithelial keratin 2e	4.34
Mtr.10968.1.S1_at	Osmotin-like protein	4.34
Mtr.37526.1.S1_at	Aluminum-induced protein	4.33
Mtr.9327.1.S1_at	Pre-mRNA splicing factor ATP-dependent RNA helicase	4.33
Mtr.41307.1.S1_at	Hypothetical protein	4.33
Mtr.41986.1.S1_at	Wall-associated kinase 4	4.32
Msa.821.1.S1_at	Hypothetical protein	4.32
Mtr.40416.1.S1_at	Hypothetical protein	4.32
Mtr.12229.1.S1_s_at	RING zinc finger protein	4.32
Mtr.43025.1.S1_at	Histone H1	4.32
Mtr.38027.1.S1_at	Hypothetical protein	4.31
Mtr.21000.1.S1_at	Universal stress protein	4.31
Mtr.12155.1.S1_at	WRKY-type DNA binding protein	4.31
Mtr.13334.1.S1_at	Acetoacetyl-CoA thiolase	4.31
Mtr.7653.1.S1_at	Hypothetical protein	4.31
Msa.963.1.S1_at	Hypothetical protein	4.31
Mtr.2846.1.S1_at	Hypothetical protein	4.31
Mtr.9291.1.S1_s_at	Hypothetical protein	4.30
Mtr.32787.1.S1_at	ARIADNE-like protein	4.30
Mtr.38881.1.S1_at	Hypothetical protein	4.30
Mtr.37524.1.S1_at	Hypothetical protein	4.29
Mtr.47843.1.S1_at	Monosaccharide transporter	4.29
Msa.2981.1.S1_at	Hypothetical protein	4.29
Mtr.9182.1.S1_at	Hypothetical protein	4.29
Mtr.28948.1.S1_at	Hypothetical protein	4.29
Mtr.41187.1.S1_at	bZip transcription factor	4.29
Mtr.38026.1.S1_at	Hypothetical protein	4.29
Mtr.32914.1.S1_at	Ethylene-induced esterase	4.29
Mtr.38205.1.S1_at	GDP-Mannose transporter	4.28
Mtr.5684.1.S1_at	Hypothetical protein	4.28
Mtr.47077.1.S1_at	Hypothetical protein	4.28
Mtr.2617.1.S1_s_at	Hypothetical protein	4.28
Msa.2535.1.S1_at	Hypothetical protein	4.28
Mtr.32999.1.S1_at	Hypothetical protein	4.27
Mtr.7519.1.S1_at	IFA-binding protein	4.27
Mtr.48842.1.S1_at	Wound-induced protein	4.27
Mtr.33699.1.S1_at	Trehalose-6-phosphate synthase	4.27
Mtr.9697.1.S1_at	Hypothetical protein	4.26

Mtr.24465.1.S1_x_at	Receptor-like protein kinase		4.26
Msa.1515.1.S1_at	Hypothetical protein		4.26
Mtr.39883.1.S1_at	ARIADNE-like protein ARI7		4.26
Msa.1479.1.S1_at	Hypothetical protein		4.25
Mtr.13667.1.S1_at	Hypothetical protein		4.25
Mtr.33608.1.S1_at	Hypothetical protein		4.25
Mtr.43583.1.S1_at	Hypothetical protein		4.25
Mtr.40099.1.S1_at	Hypothetical protein		4.25
Mtr.1398.1.S1_s_at	Hypothetical protein		4.25
Mtr.11911.1.S1_at	Hypothetical protein		4.25
Mtr.40933.1.S1_at	TIR-similar-domain-containing protein		4.25
Mtr.31.1.S1_at	Peroxidase 3		4.25
Mtr.46098.1.S1_s_at	Zn-finger, DHHC type		4.24
Mtr.14729.1.S1_at	RNA-processing protein		4.24
Mtr.13773.1.S1_at	NADH dehydrogenase subunit 4		4.24
Mtr.6918.1.S1_at	Phospholipase PLDb1		4.24
Mtr.42289.1.S1_at	Hypothetical protein		4.24
Mtr.12490.1.S1_at	Hypothetical protein		4.24
Mtr.31741.1.S1_at	WD repeat domain		4.24
Mtr.35611.1.S1_at	Hypothetical protein		4.24
Mtr.43612.1.S1_at	WRKY transcription factor 65		4.24
Mtr.40144.1.S1_at	Glutamate-rich protein		4.24
Mtr.11878.1.S1_at	Receptor protein kinase		4.23
Mtr.32533.1.S1_s_at	Hypothetical protein		4.23
Mtr.40021.1.S1_at	Germin-like protein		4.23
Mtr.40178.1.S1_at	Hypothetical protein		4.23
Mtr.43355.1.S1_at	Hypothetical protein		4.22
Mtr.7268.1.S1_at	Hypothetical protein		4.22
Mtr.43483.1.S1_at	Hypothetical protein		4.22
Mtr.16414.1.S1_s_at	Ubiquitin system component Cue		4.22
Mtr.27346.1.S1_at	Hypothetical protein		4.22
Mtr.17962.1.S1_at	DNA-binding WRKY		4.22
Mtr.38070.1.S1_at	Hypothetical protein		4.22
Mtr.47086.1.S1_at	Glucan 1,3-beta-glucosidase		4.22
Mtr.13392.1.S1_at	Protein kinase		4.22
Mtr.900.1.S1_at	PPR repeat-containing protein		4.21
Mtr.41029.1.S1_at	Ankyrin repeat-containing protein		4.21
Mtr.17691.1.S1_at	Transcriptional factor		4.21
Mtr.12449.1.S1_at	Hypothetical protein		4.21
Mtr.9139.1.S1_at	Hypothetical protein		4.21
Mtr.38655.1.S1_at	Hypothetical protein		4.20
Mtr.51932.1.S1_at	Quinonprotein alcohol dehydrogenase		4.20
Msa.1174.1.S1_at	Hypothetical protein		4.20
Mtr.6343.1.S1_at	Hydroxyproline-rich glycoprotein		4.20
Mtr.34454.1.S1_at	Hypothetical protein		4.19
Mtr.32738.1.S1_at	Myo-inositol oxygenase		4.19
Mtr.45883.1.S1_at	Hypothetical protein		4.19
Mtr.44614.1.S1_at	Auxilin-like protein		4.19
Mtr.50730.1.S1_at	bZIP transcription factor		4.18
Mtr.2533.1.S1_at	Hypothetical protein		4.18
Mtr.12497.1.S1_at	Protein kinase		4.18
Msa.1370.1.S1_at	Hypothetical protein		4.18
Mtr.15199.1.S1_at	Hypothetical protein		4.17
Mtr.34874.1.S1_s_at	Dolichyl-phosphate beta-glucosyltransferase homolog	At2g39630 -	4.17

Mtr.37684.1.S1_at	Actin-depolymerizing factor 5	4.17
Mtr.38752.1.S1_at	Geranyl diphosphate synthase small subunit	4.17
Mtr.18069.1.S1_x_at	Disease resistance protein	4.17
Mtr.12229.1.S1_at	RING zinc finger protein	4.16
Mtr.40631.1.S1_at	Protein kinase	4.16
Mtr.5750.1.S1_at	EREBP-3-like protein	4.16
Mtr.40315.1.S1_at	Al-induced protein	4.16
Mtr.39023.1.S1_at	Hypothetical protein	4.16
Mtr.32808.1.S1_at	Hypothetical protein	4.16
Mtr.29855.1.S1_at	Hypothetical protein	4.15
Mtr.42598.1.S1_at	Hypothetical protein	4.15
Mtr.5723.1.S1_at	Hypothetical protein	4.15
Mtr.52078.1.S1_s_at	Hypothetical protein	4.15
Mtr.39031.1.S1_at	Hypothetical protein	4.15
Mtr.7637.1.S1_at	Prostatic spermine-binding protein	4.15
Mtr.9362.1.S1_at	Hypothetical protein	4.14
Mtr.41578.1.S1_at	Hypothetical protein	4.14
Mtr.5079.1.S1_at	Syringolide-induced protein 1-3-1B	4.14
Mtr.12878.1.S1_at	Nucleoid DNA-binding-like protein	4.14
Mtr.39540.1.S1_at	AttA2-like ABC transporter	4.13
Mtr.41752.1.S1_at	Geraniol 10-hydroxylase	4.13
Mtr.47433.1.S1_at	Hypothetical protein	4.13
Mtr.41733.1.S1_at	Hypothetical protein	4.13
Mtr.23052.1.S1_at	AAA-type ATPase	4.13
Mtr.52169.1.S1_at	Hypothetical protein	4.13
Mtr.4342.1.S1_at	Hypothetical protein	4.12
Mtr.43909.1.S1_at	RNA-binding region RNP-1	4.12
Mtr.11114.1.S1_at	Hypothetical protein	4.12
Mtr.12146.1.S1_at	Hypothetical protein	4.12
Mtr.45293.1.S1_s_at	Hypothetical protein	4.12
Mtr.27620.1.S1_at	Hypothetical protein	4.12
Mtr.4829.1.S1_s_at	Lipoate protein ligase	4.12
Mtr.34899.1.S1_at	Hexose carrier protein	4.12
Mtr.40696.1.S1_at	Hypothetical protein	4.11
Mtr.39259.1.S1_at	Hypothetical protein	4.11
Mtr.45285.1.S1_at	Hypothetical protein	4.11
Msa.2736.1.S1_at	Hypothetical protein	4.11
Mtr.15856.1.S1_x_at	RNA-binding region RNP-1	4.10
Mtr.13660.1.S1_at	ERD3 protein	4.09
Mtr.40876.1.S1_at	Hypothetical protein	4.09
Mtr.44123.1.S1_at	Lipase class 3	4.09
Mtr.42359.1.S1_at	Hypothetical protein	4.09
Mtr.9135.1.S1_at	Homeobox-leucine zipper protein	4.09
Mtr.11991.1.S1_at	Strubbelig receptor family	4.09
Mtr.28237.1.S1_s_at	Agenet domain-containing protein	4.09
Mtr.1874.1.S1_s_at	Potential phospholipid-transporting ATPase 9	4.08
Msa.2660.1.S1_at	Hypothetical protein	4.08
Mtr.43097.1.S1_at	Beta-amylase	4.08
Mtr.9188.1.S1_at	Hypothetical protein	4.08
Mtr.45020.1.S1_at	Beta-glucosidase-like protein	4.08
Mtr.5663.1.S1_at	Beta-galactosidase	4.08
Mtr.13156.1.S1_at	Hypothetical protein	4.08
Mtr.51565.1.S1_s_at	Galactose-binding protein	4.07
Mtr.7084.1.S1_at	Asparagine synthase	4.07

Mtr.32367.1.S1_s_at	Peroxidase 3	4.07
Mtr.38484.1.S1_at	Yippee-like protein	4.07
Mtr.23415.1.S1_at	Hypothetical protein	4.07
Mtr.20173.1.S1_at	Nucleoporin interacting component	4.07
Mtr.5913.1.S1_at	LIM domain containing protein	4.07
Mtr.51323.1.S1_s_at	Hypothetical protein	4.07
Mtr.11749.1.S1_at	Hypothetical protein	4.06
Mtr.44780.1.S1_at	Hypothetical protein	4.06
Mtr.38256.1.S1_s_at	Flavonol synthase	4.06
Mtr.11497.1.S1_at	Hypothetical protein	4.06
Mtr.12826.1.S1_s_at	Hypothetical protein	4.06
Mtr.8633.1.S1_at	Hypothetical protein	4.05
Mtr.30513.1.S1_s_at	Hypothetical protein	4.05
Mtr.12034.1.S1_at	ADP/ATP translocase-like protein	4.05
Mtr.22270.1.S1_s_at	Hypothetical protein	4.05
Mtr.12402.1.S1_s_at	Beta-galactosidase	4.05
Mtr.7638.1.S1_s_at	Endo-1,3-beta-glucanase	4.05
Mtr.39602.1.S1_at	Receptor protein kinase	4.05
Mtr.39985.1.S1_at	Hypothetical protein	4.05
Mtr.1757.1.S1_at	Homeodomain-leucine zipper protein	4.05
Mtr.18366.1.S1_at	Curculin-like (mannose-binding) lectin	4.04
Mtr.37522.1.S1_at	Ribosomal protein L7	4.04
Mtr.31337.1.S1_at	Serine/threonine protein kinase	4.03
Mtr.24777.1.S1_s_at	Hypothetical protein	4.03
Mtr.35926.1.S1_s_at	Hypothetical protein	4.03
Mtr.9613.1.S1_at	TIR-similar-domain-containing protein	4.03
Mtr.7416.1.S1_at	Hypothetical protein	4.03
Mtr.40735.1.S1_at	Hypothetical protein	4.03
Mtr.26549.1.S1_at	Cathepsin B-like cysteine protease	4.03
Mtr.5184.1.S1_at	Pre-mRNA splicing factor ATP-dependent RNA helicase	4.02
Mtr.7760.1.S1_at	Cdc24	4.02
Mtr.42696.1.S1_at	Nam-like protein 8	4.02
Mtr.6191.1.S1_at	Hypothetical protein	4.02
Mtr.19399.1.S1_at	Glutathione S-transferase	4.02
Mtr.13083.1.S1_at	Hypothetical protein	4.02
Mtr.40830.1.S1_at	2-hydroxyphytanoyl-CoA lyase	4.02
Mtr.43073.1.S1_s_at	Poly(A)-binding protein C-terminal interacting protein 6	4.01
Mtr.48130.1.S1_at	Hypothetical protein	4.01
Mtr.41634.1.S1_at	Receptor kinase-like protein	4.01
Mtr.6671.1.S1_at	Hypothetical protein	4.01
Mtr.29273.1.S1_at	Hypothetical protein	4.00
Mtr.50416.1.S1_at	Phosphatidylinositol 3- and 4-kinase	4.00
Mtr.8319.1.S1_at	Class III peroxidase 5	4.00
Mtr.12120.1.S1_at	Hypothetical protein	4.00
Mtr.32104.1.S1_s_at	Respiratory burst oxidase homolog	4.00
Mtr.41920.1.S1_at	Hypothetical protein	4.00
Msa.1168.1.S1_at	Hypothetical protein	3.99
Mtr.16733.1.S1_s_at	Protein kinase	3.99
Mtr.44989.1.S1_at	Nucleosome assembly protein 1-like protein 2	3.99
Mtr.45902.1.S1_at	Hypothetical protein	3.99
Mtr.42911.1.S1_at	RING zinc finger protein	3.99
Mtr.39307.1.S1_at	Hypothetical protein	3.99
Mtr.34450.1.S1_at	Hypothetical protein	3.99
Mtr.51636.1.S1_at	Hypothetical protein	3.99

Mtr.39665.1.S1_at	Hypothetical protein	3.99
Mtr.37685.1.S1_at	Actin-depolymerizing factor 5	3.99
Mtr.13525.1.S1_at	Hypothetical protein	3.99
Mtr.43170.1.S1_at	Glutamate dehydrogenase 1	3.99
Mtr.31335.1.S1_s_at	Alcohol dehydrogenase	3.99
Mtr.40805.1.S1_at	Hypothetical protein	3.98
Mtr.12948.1.S1_at	Dehydration responsive element binding protein	3.98
Mtr.45225.1.S1_at	Ankyrin-like protein	3.98
Mtr.9826.1.S1_at	Hypothetical protein	3.98
Mtr.50554.1.S1_at	Hypothetical protein	3.98
Mtr.50859.1.S1_s_at	Hypothetical protein	3.98
Mtr.40114.1.S1_s_at	Soluble inorganic pyrophosphatase	3.98
Mtr.10781.1.S1_at	Nuclease	3.98
Mtr.32930.1.S1_at	Plasma membrane ATPase 3	3.98
Mtr.38139.1.S1_s_at	Zinc finger protein	3.98
Mtr.45315.1.S1_at	SPF1 protein	3.98
Mtr.37972.1.S1_at	Hypothetical protein	3.97
Mtr.47631.1.S1_s_at	Transposase	3.97
Mtr.37260.1.S1_at	Agenet domain-containing protein	3.97
Mtr.38221.1.S1_at	Hypothetical protein	3.97
Mtr.413.1.S1_at	CLAVATA1 receptor kinase (CLV1)-like protein	3.97
Mtr.51664.1.S1_s_at	Natural resistance-associated macrophage protein	3.97
Mtr.32717.1.S1_at	Hypothetical protein	3.97
Mtr.205.1.S1_at	40S ribosomal protein S12	3.96
Mtr.38928.1.S1_at	Choline transporter	3.96
Mtr.49053.1.S1_at	Esterase/lipase/thioesterase	3.96
Mtr.39999.1.S1_at	Syringolide-induced protein B13-1-9	3.96
Mtr.44627.1.S1_at	Hypothetical protein	3.96
Mtr.32566.1.S1_x_at	DEAD box protein	3.96
Mtr.3600.1.S1_at	Two-component response regulator ARR2	3.96
Mtr.10806.1.S1_at	Hypothetical protein	3.96
Mtr.12240.1.S1_at	Envelope glycoprotein	3.96
Mtr.38171.1.S1_at	Monooxygenase 2	3.95
Mtr.51425.1.S1_at	Protein kinase	3.95
Mtr.41380.1.S1_at	Hypothetical protein	3.95
Mtr.1871.1.S1_at	Hypothetical protein	3.95
Mtr.32666.1.S1_at	Hypothetical protein	3.95
Mtr.44219.1.S1_at	Cytokinin oxidase-like protein	3.95
Mtr.10956.1.S1_at	Hypothetical protein	3.95
Mtr.39788.1.S1_at	P12 I protein	3.95
Mtr.44772.1.S1_at	Elongation factor G	3.94
Mtr.11377.1.S1_at	Glutathione S-transferase GST 7	3.94
Mtr.26949.1.S1_at	Hypothetical protein	3.94
Mtr.8990.1.S1_at	Hypothetical protein	3.93
Mtr.39643.1.S1_at	Hypothetical protein	3.93
Mtr.32783.1.S1_at	Myosin	3.93
Mtr.33585.1.S1_at	Hypothetical protein	3.93
Mtr.12961.1.S1_at	Hypothetical protein	3.93
Mtr.2249.1.S1_at	Hypothetical protein	3.93
Mtr.13237.1.S1_at	Protein integral membrane protein	3.93
Mtr.43732.1.S1_at	Hypothetical protein	3.92
Mtr.2720.1.S1_at	Gyrase A	3.92
Mtr.6315.1.S1_at	GCN4-complementing protein	3.92
Mtr.30749.1.S1_s_at	Hypothetical protein	3.92

Mtr.50704.1.S1_at	Aldehyde dehydrogenase	3.92
Mtr.44157.1.S1_at	C3HC4-type RING zinc finger protein	3.92
Mtr.33563.1.S1_at	Hypothetical protein	3.91
Mtr.27324.1.S1_s_at	Xyloglucan endo-1,4-beta-D-glucanase	3.91
Mtr.30728.1.S1_at	SAR DNA-binding protein	3.91
Mtr.44885.1.S1_at	Hypothetical protein	3.91
Mtr.4569.1.S1_at	Hypothetical protein	3.90
Mtr.37468.1.S1_at	Hypothetical protein	3.90
Mtr.40166.1.S1_s_at	Phenylalanine ammonia-lyase	3.90
Mtr.33274.1.S1_at	Hypothetical protein	3.90
Mtr.42007.1.S1_at	Hypothetical protein	3.90
Mtr.29833.1.S1_s_at	Hypothetical protein	3.90
Mtr.27130.1.S1_s_at	Hypothetical protein	3.90
Mtr.38461.1.S1_at	Calmodulin-like domain protein kinase	3.90
Mtr.9348.1.S1_s_at	Ubiquitin-protein ligase	3.89
Mtr.41339.1.S1_at	Hypothetical protein	3.89
Mtr.26514.1.S1_s_at	F-box protein	3.89
Mtr.42649.1.S1_at	Hypothetical protein	3.89
Mtr.37153.1.S1_at	Light repressible receptor protein kinase	3.89
Mtr.5061.1.S1_at	ABC transporter-like protein	3.89
Mtr.16955.1.S1_at	Ribosomal protein S9	3.89
Mtr.27222.1.S1_at	Hypothetical protein	3.89
Mtr.27738.1.S1_at	ATP-dependent molecular chaperone HSP82	3.88
Mtr.1874.1.S1_at	Potential phospholipid-transporting ATPase 9	3.88
Msa.1037.1.S1_at	Hypothetical protein	3.88
Mtr.8934.1.S1_at	Alternative oxidase 3	3.88
Mtr.31852.1.S1_s_at	GATA-binding transcription factor	3.88
Mtr.39487.1.S1_s_at	Lipoxygenase	3.88
Mtr.33544.1.S1_at	Beta-galactosidase	3.88
Mtr.39950.1.S1_at	Hypothetical protein	3.88
Mtr.40801.1.S1_at	Hypothetical protein	3.88
Mtr.7429.1.S1_s_at	Hypothetical protein	3.88
Mtr.34946.1.S1_at	Hypothetical protein	3.87
Mtr.9141.1.S1_at	Hypothetical protein	3.87
Mtr.4233.1.S1_at	Hypothetical protein	3.87
Mtr.24351.1.S1_s_at	Hypothetical protein	3.87
Mtr.14442.1.S1_at	Hypothetical protein	3.87
Mtr.44992.1.S1_s_at	Serine/threonine protein kinase	3.87
Mtr.37020.1.S1_at	NADH-ubiquinone oxidoreductase subunit 8	3.86
Mtr.43657.1.S1_at	Hypothetical protein	3.86
Mtr.29988.1.S1_at	ABC transporter	3.86
Mtr.8468.1.S1_at	Cysteine synthase	3.86
Mtr.38837.1.S1_s_at	Hypothetical protein	3.86
Mtr.38935.1.S1_at	Myo-inositol transporter 1	3.86
Mtr.13559.1.S1_s_at	LOB domain protein 38	3.86
Mtr.2124.1.S1_s_at	Hypothetical protein	3.85
Mtr.20985.1.S1_at	acyl-CoA oxidase	3.85
Mtr.32298.1.S1_at	QbsK	3.85
Mtr.24374.1.S1_at	Fiber protein Fb2	3.85
Msa.3087.1.S1_at	Hypothetical protein	3.85
Mtr.12430.1.S1_s_at	Mature anther-specific protein	3.85
Mtr.43353.1.S1_at	Hypothetical protein	3.85
Mtr.12636.1.S1_at	Fructokinase	3.85
Mtr.46843.1.S1_at	F-box protein	3.84

Mtr.25309.1.S1_at	Hypothetical protein	3.84
Mtr.5571.1.S1_s_at	Hypothetical protein	3.84
Mtr.2503.1.S1_s_at	NEP1-interacting protein	3.84
Mtr.41096.1.S1_at	Tubby-like protein	3.84
Mtr.44922.1.S1_at	Glycine-rich protein 2	3.84
Mtr.14517.1.S1_at	RNA-binding region RNP-1	3.84
Mtr.44365.1.S1_s_at	Adenine-specific DNA modification methyltransferase	3.84
Mtr.10588.1.S1_at	Hypothetical protein	3.84
Mtr.42452.1.S1_at	Hypothetical protein	3.84
Mtr.7575.1.S1_at	Hypothetical protein	3.84
Mtr.12168.1.S1_at	Diamine oxidase (Copper amino oxidase)	3.83
Mtr.4050.1.S1_at	Hypothetical protein	3.83
Mtr.52112.1.S1_s_at	RNA-binding region RNP-1	3.83
Mtr.39422.1.S1_s_at	Hypothetical protein	3.83
Mtr.18340.1.S1_at	Hypothetical protein	3.83
Mtr.31877.1.S1_at	P-glycoprotein	3.83
Mtr.10621.1.S1_at	Hypothetical protein	3.82
Msa.1041.1.S1_at	Hypothetical protein	3.82
Mtr.13453.1.S1_at	Hypothetical protein	3.82
Mtr.37222.1.S1_at	Hypothetical protein	3.82
Mtr.35818.1.S1_at	Hypothetical protein	3.82
Mtr.8994.1.S1_s_at	Hypothetical protein	3.82
Mtr.11606.1.S1_at	Hypothetical protein	3.81
Mtr.41411.1.S1_at	Hypothetical protein	3.81
Mtr.9913.1.S1_at	Hypothetical protein	3.81
Mtr.11493.1.S1_at	Hypothetical protein	3.81
Mtr.40100.1.S1_at	Soluble inorganic pyrophosphatase	3.81
Mtr.41152.1.S1_at	Hypothetical protein	3.80
Mtr.11252.1.S1_s_at	Receptor kinase-like protein	3.80
Mtr.27102.1.S1_at	Hypothetical protein	3.80
Mtr.22662.1.S1_at	NADP-dependent malic enzyme	3.80
Mtr.9290.1.S1_at	Hypothetical protein	3.80
Mtr.13010.1.S1_at	Xyloglucan endotransglucosylase/hydrolase protein 22	3.80
Mtr.13696.1.S1_at	Hypothetical protein	3.79
Mtr.9028.1.S1_at	Hypothetical protein	3.79
Mtr.9177.1.S1_at	Hypothetical protein	3.79
Mtr.3871.1.S1_at	CCAAT-box-binding transcription factor	3.79
Mtr.9272.1.S1_at	Hypothetical protein	3.79
Mtr.8592.1.S1_at	Hypothetical protein	3.79
Mtr.38966.1.S1_at	Ser/Thr protein kinase	3.79
Mtr.39812.1.S1_s_at	Respiratory burst oxidase homolog	3.79
Mtr.33788.1.S1_at	Hypothetical protein	3.78
Mtr.39578.1.S1_at	A-agglutinin attachment subunit precursor	3.78
Mtr.42029.1.S1_s_at	Metallo-beta-lactamase	3.78
Mtr.38970.1.S1_at	Hypothetical protein	3.78
Mtr.10037.1.S1_at	GDSL-motif lipase/hydrolase-like protein	3.78
Mtr.22904.1.S1_s_at	Aux/IAA protein	3.78
Mtr.34203.1.S1_at	UDP-glucuronosyltransferase	3.77
Mtr.43237.1.S1_at	TFIIE-beta 2	3.77
Mtr.37374.1.S1_at	Patatin-like protein	3.77
Mtr.44688.1.S1_at	Hypothetical protein	3.77
Mtr.52010.1.S1_at	Myb, DNA-binding	3.77
Mtr.51695.1.S1_at	Hypothetical protein	3.77
Mtr.13329.1.S1_at	Hypothetical protein	3.77

Mtr.33699.1.S1_x_at	Trehalose-7-phosphate synthase	3.77
Mtr.45244.1.S1_at	Hypothetical protein	3.77
Msa.2711.1.S1_at	Hypothetical protein	3.77
Mtr.46485.1.S1_at	Glutathione S-transferase GST 17	3.77
Mtr.11018.1.S1_s_at	Hypothetical protein	3.76
Mtr.17513.1.S1_s_at	bZIP transcription factor	3.76
Mtr.50280.1.S1_at	Protein kinase	3.76
Mtr.41853.1.S1_at	Zinc finger, C3HC4 type (RING finger)	3.76
Mtr.37655.1.S1_at	Nucleolar histone deacetylase	3.76
Msa.1013.1.S1_at	Hypothetical protein	3.75
Mtr.3073.1.S1_s_at	Type IIA calcium ATPase	3.75
Mtr.13695.1.S1_at	Hypothetical protein	3.75
Mtr.11240.1.S1_at	DR2R protein	3.75
Mtr.43466.1.S1_at	Ethylene receptor	3.75
Mtr.50858.1.S1_s_at	Hypothetical protein	3.74
Mtr.10672.1.S1_s_at	Polyphosphoinositide binding protein	3.74
Mtr.31538.1.S1_at	Nam-like protein	3.74
Mtr.2864.1.S1_at	Hypothetical protein	3.74
Mtr.18298.1.S1_at	Serine/threonine protein kinase	3.74
Mtr.21266.1.S1_at	Hypothetical protein	3.74
Mtr.2158.1.S1_at	Hypothetical protein	3.74
Mtr.32889.1.S1_at	Hypothetical protein	3.74
Mtr.37560.1.S1_at	Pyruvate decarboxylase	3.74
Mtr.44718.1.S1_at	Hypothetical protein	3.73
Msa.884.1.S1_at	Hypothetical protein	3.73
Mtr.6540.1.S1_at	Lipoxygenase	3.73
Mtr.24211.1.S1_at	Hypothetical protein	3.73
Mtr.42547.1.S1_s_at	Respiratory burst oxidase homolog	3.72
Mtr.2617.1.S1_at	Hypothetical protein	3.72
Mtr.10529.1.S1_at	ARF GAP-like zinc finger-containing protein	3.72
Mtr.8642.1.S1_at	Serine palmitoyltransferase	3.72
Mtr.41656.1.S1_at	Hypothetical protein	3.72
Mtr.38654.1.S1_at	Hypothetical protein	3.72
Mtr.27949.1.S1_at	Internalin-related protein	3.72
Mtr.17721.1.S1_s_at	Esterase/lipase/thioesterase	3.72
Mtr.46896.1.S1_s_at	Calcium-dependent protein kinase CPK1 adapter protein 2	3.72
Mtr.29231.1.S1_at	Alpha-expansin	3.72
Mtr.43169.1.S1_at	Hypothetical protein	3.72
Mtr.40685.1.S1_at	Hypothetical protein	3.72
Mtr.43116.1.S1_at	RAV2 protein	3.72
Mtr.17714.1.S1_at	Protein prenyltransferase	3.72
Mtr.12238.1.S1_at	Nam-like protein	3.71
Mtr.2238.1.S1_s_at	Repetitive proline-rich cell wall protein	3.71
Mtr.50954.1.S1_s_at	Hypothetical protein	3.71
Mtr.52341.1.S1_at	Cyclin-like F-box protein	3.71
Mtr.41245.1.S1_at	Chorismate mutase CM2	3.71
Mtr.44335.1.S1_at	Glucosidase SCW10	3.71
Mtr.13183.1.S1_at	Hypothetical protein	3.71
Mtr.45199.1.S1_at	Hypothetical protein	3.71
Mtr.11232.1.S1_at	Serine/threonine specific protein kinase	3.70
Mtr.41051.1.S1_at	Hypothetical protein	3.70
Mtr.20124.1.S1_at	Serine/threonine protein kinase	3.70
Mtr.13005.1.S1_at	Phytochrome A specific signal transduction component PAT3	3.70
Mtr.8632.1.S1_s_at	Hypothetical protein	3.70

Mtr.13155.1.S1_at	Hypothetical protein	3.69
Mtr.19727.1.S1_at	Hypothetical protein	3.69
Mtr.9074.1.S1_at	Hypothetical protein	3.69
Mtr.41494.1.S1_at	Hypothetical protein	3.69
Mtr.43604.1.S1_s_at	Hypothetical protein	3.69
Mtr.12004.1.S1_at	Hypothetical protein	3.69
Mtr.41349.1.S1_s_at	Hydrolase, alpha/beta fold family protein	3.69
Mtr.3000.1.S1_s_at	DEAD-Box RNA helicase	3.69
Mtr.42544.1.S1_at	Nodulin-like protein	3.69
Mtr.45383.1.S1_at	Hypothetical protein	3.69
Mtr.43493.1.S1_at	Hypothetical protein	3.68
Mtr.34629.1.S1_at	Protein transport protein	3.68
Mtr.34149.1.S1_at	Hypothetical protein	3.68
Mtr.43487.1.S1_at	Hypothetical protein	3.68
Mtr.24373.1.S1_at	Fiber protein Fb2	3.68
Mtr.14781.1.S1_at	Hypothetical protein	3.68
Mtr.13058.1.S1_at	Hypothetical protein	3.68
Mtr.14663.1.S1_at	Major intrinsic protein	3.67
Mtr.9680.1.S1_at	Zinc finger protein	3.67
Mtr.50378.1.S1_s_at	AAA ATPas	3.67
Mtr.8479.1.S1_at	Cytochrome P450	3.67
Mtr.51785.1.S1_at	Hypothetical protein	3.67
Mtr.9478.1.S1_at	Laccase-like protein	3.66
Mtr.37418.1.S1_s_at	NuM1 protein	3.66
Mtr.9348.1.S1_at	Ubiquitin-protein ligase	3.66
Mtr.27778.1.S1_at	Serine/threonine protein kinase	3.66
Mtr.13338.1.S1_at	Hypothetical protein	3.66
Mtr.33754.1.S1_at	Hypothetical protein	3.66
Mtr.6498.1.S1_at	Hypothetical protein	3.66
Mtr.33907.1.S1_at	Hypothetical protein	3.66
Mtr.28680.1.S1_at	Hypothetical protein	3.65
Mtr.20079.1.S1_at	Lipoxygenase	3.65
Mtr.9043.1.S1_at	LS1-like protein	3.65
Mtr.36255.1.S1_at	Aquaporin-like transmembrane channel protein	3.65
Mtr.39204.1.S1_at	Hypothetical protein	3.65
Mtr.10636.1.S1_at	MYB protein	3.65
Mtr.12226.1.S1_at	Hypothetical protein	3.65
Mtr.12750.1.S1_at	Hypothetical protein	3.65
Mtr.2386.1.S1_at	Hypothetical protein	3.65
Mtr.19793.1.S1_at	Hypothetical protein	3.65
Mtr.6526.1.S1_at	Hypothetical protein	3.65
Mtr.39287.1.S1_at	SGP1 monomeric G-protein	3.65
Mtr.23284.1.S1_at	Calcium-binding EF-hand family protein	3.64
Msa.1303.1.S1_at	Hypothetical protein	3.64
Mtr.13714.1.S1_at	Auxin-induced protein 22D	3.64
Mtr.791.1.S1_at	Acyl-activating enzyme 18	3.64
Mtr.31446.1.S1_at	Hypothetical protein	3.64
Mtr.47761.1.S1_at	Hypothetical protein	3.64
Mtr.6934.1.S1_at	Glutaredoxin-related-like protein	3.63
Mtr.37300.1.S1_at	Cytochrome P450	3.63
Mtr.48357.1.S1_at	Trypsin protein inhibitor 2	3.63
Mtr.7578.1.S1_at	Hypothetical protein	3.63
Mtr.2889.1.S1_at	Hypothetical protein	3.63
Mtr.24715.1.S1_at	Glutathione S-transferase GST 14	3.63

Mtr.38725.1.S1_s_at	CDPK adapter protein 1	3.63
Mtr.11290.1.S1_at	Hypothetical protein	3.62
Mtr.34834.1.S1_at	Serine/threonine protein kinase	3.62
Mtr.24319.1.S1_at	Hypothetical protein	3.62
Mtr.6545.1.S1_x_at	Hypothetical protein	3.62
Mtr.37417.1.S1_s_at	NuM1 protein	3.62
Mtr.41563.1.S1_at	Ring finger E3 ligase	3.62
Mtr.11369.1.S1_at	Hypothetical protein	3.62
Mtr.37278.1.S1_at	Cysteine synthase	3.62
Mtr.4899.1.S1_at	Hypothetical protein	3.62
Mtr.45415.1.S1_a_at	Deetiolated 1-like protein	3.62
Mtr.10747.1.S1_at	Adenosine monophosphate binding protein	3.61
Mtr.23686.1.S1_at	DNA-directed RNA polymerase	3.61
Mtr.38306.1.S1_at	Choline transporter-like protein	3.61
Mtr.51256.1.S1_at	Transferase	3.61
Mtr.23653.1.S1_at	Hypothetical protein	3.61
Mtr.14656.1.S1_at	Hypothetical protein	3.61
Mtr.21943.1.S1_s_at	CYP83D1p	3.61
Mtr.4402.1.S1_at	Hypothetical protein	3.61
Mtr.36943.1.S1_at	Hypothetical protein	3.61
Mtr.16854.1.S1_at	Protein kinase	3.61
Mtr.43954.1.S1_at	Hypothetical protein	3.61
Mtr.43258.1.S1_s_at	Hypothetical protein	3.60
Mtr.37645.1.S1_at	Hypothetical protein	3.60
Mtr.10728.1.S1_at	Hypothetical protein	3.60
Mtr.43192.1.S1_at	Mature anther-specific protein LAT61	3.60
Mtr.28411.1.S1_s_at	Hypothetical protein	3.60
Mtr.12531.1.S1_s_at	Hypothetical protein	3.60
Mtr.27354.1.S1_s_at	RPM1-interacting protein 4	3.60
Mtr.10700.1.S1_at	KCBP interacting Ca2+-binding protein	3.60
Mtr.38128.1.S1_s_at	Aldehyde dehydrogenase	3.60
Mtr.37422.1.S1_at	Hypothetical protein	3.59
Mtr.41435.1.S1_s_at	Hypothetical protein	3.59
Mtr.37508.1.S1_at	4- Hydroxyphenylpyruvate Dioxygenases	3.59
Mtr.50583.1.S1_at	Glycoside hydrolase	3.59
Mtr.42149.1.S1_at	Hypothetical protein	3.59
Mtr.25204.1.S1_s_at	Calmodulin-binding protein	3.59
Mtr.15543.1.S1_at	Peptidylprolyl isomerase, FKBP-type	3.58
Mtr.5620.1.S1_at	Membrane cofactor protein	3.58
Mtr.33373.1.S1_at	Hypothetical protein	3.58
Mtr.42860.1.S1_at	Ferredoxin III	3.58
Mtr.44291.1.S1_at	Hypothetical protein	3.58
Mtr.51366.1.S1_s_at	RNA-binding region RNP-1	3.58
Mtr.40888.1.S1_at	E3 ubiquitin ligase	3.58
Mtr.31724.1.S1_at	Hypothetical protein	3.58
Mtr.6886.1.S1_at	Hypothetical protein	3.58
Mtr.37900.1.S1_at	Pantothenate kinase 1	3.58
Mtr.12154.1.S1_at	Hypothetical protein	3.58
Mtr.10320.1.S1_at	Uricase	3.57
Mtr.26364.1.S1_at	Hypothetical protein	3.57
Mtr.17461.1.S1_at	Hypothetical protein	3.56
Msa.1667.1.S1_at	Hypothetical protein	3.56
Mtr.39837.1.S1_at	Hypothetical protein	3.56
Mtr.16850.1.S1_at	Curculin-like (mannose-binding) lectin	3.56

Mtr.11604.1.S1_s_at	Hypothetical protein	3.56
Mtr.16393.1.S1_at	Hypothetical protein	3.56
Mtr.38482.1.S1_at	Hypothetical protein	3.56
Mtr.13244.1.S1_at	Hypothetical protein	3.56
Mtr.4546.1.S1_s_at	E3 ubiquitin ligase	3.56
Mtr.44290.1.S1_at	Hypothetical protein	3.56
Mtr.36788.1.S1_at	Hypothetical protein	3.56
Msa.1673.1.S1_at	Hypothetical protein	3.56
Mtr.15716.1.S1_at	WD40-like protein	3.56
Mtr.5457.1.S1_at	Transducin-like protein	3.55
Mtr.19469.1.S1_at	Hypothetical protein	3.55
Mtr.42760.1.S1_at	Hypothetical protein	3.55
Mtr.27895.1.S1_at	Ser/Thr protein phosphatase 2C	3.55
Mtr.24817.1.S1_at	Fructan 1-exohydrolase	3.55
Mtr.35606.1.S1_at	Hypothetical protein	3.55
Mtr.38799.1.S1_s_at	Cytokinin oxidase	3.55
Mtr.15257.1.S1_s_at	Hypothetical protein	3.55
Mtr.42336.1.S1_at	WRKY transcription factor 48	3.55
Mtr.31731.1.S1_at	Eukaryotic initiation factor	3.55
Mtr.31446.1.S1_x_at	Hypothetical protein	3.54
Mtr.41041.1.S1_at	Notchless protein homolog	3.54
Mtr.43547.1.S1_at	Hypothetical protein	3.54
Mtr.21943.1.S1_x_at	CYP83D1p	3.54
Mtr.13414.1.S1_at	ABC-type transporter	3.54
Mtr.10424.1.S1_at	Ethylene responsive element binding factor	3.54
Mtr.15744.1.S1_at	Hypothetical protein	3.54
Mtr.34197.1.S1_at	Hypothetical protein	3.54
Mtr.36809.1.S1_at	Hypothetical protein	3.53
Msa.1038.1.S1_at	Hypothetical protein	3.53
Mtr.428.1.S1_at	Leucine rich repeat protein	3.53
Mtr.32968.1.S1_at	Mitochondrial solute carrier protein	3.52
Mtr.43953.1.S1_s_at	Hypothetical protein	3.52
Mtr.5572.1.S1_s_at	CREB-like protein	3.52
Mtr.19044.1.S1_at	Hypothetical protein	3.52
Mtr.44176.1.S1_at	Squalene monooxygenase 1	3.52
Mtr.2676.1.S1_at	Hypothetical protein	3.52
Mtr.37901.1.S1_at	Hypothetical protein	3.52
Mtr.51291.1.S1_at	Pathogenesis-related transcriptional factor and ERF	3.52
Mtr.10425.1.S1_at	Ethylene responsive element binding factor	3.51
Mtr.17506.1.S1_at	ATPase, E1-E2	3.51
Mtr.33247.1.S1_at	Hypothetical protein	3.51
Msa.1220.1.S1_at	Hypothetical protein	3.51
Mtr.13077.1.S1_at	Hypothetical protein	3.51
Mtr.8704.1.S1_at	Hypothetical protein	3.51
Mtr.3225.1.S1_at	Hypothetical protein	3.50
Mtr.35440.1.S1_at	Hypothetical protein	3.50
Mtr.2486.1.S1_at	Hypothetical protein	3.50
Mtr.33034.1.S1_at	Chromobox protein	3.50
Mtr.10360.1.S1_s_at	ER6 protein	3.50
Mtr.33389.1.S1_at	Hypothetical protein	3.50
Mtr.4041.1.S1_at	Nucleic acid binding protein	3.50
Mtr.12973.1.S1_at	Prohibitin 1-like protein	3.50
Mtr.21812.1.S1_at	Hypothetical protein	3.49
Mtr.44464.1.S1_at	Elongation factor G	3.49

Mtr.13657.1.S1_at	Hypothetical protein	3.49
Mtr.2537.1.S1_a_at	Heterogeneous nuclear ribonucleoprotein	3.49
Mtr.32940.1.S1_at	N-hydroxycinnamoyl/benzoyltransferase	3.49
Mtr.12047.1.S1_at	Hypothetical protein	3.49
Mtr.44333.1.S1_s_at	Hypothetical protein	3.49
Mtr.13103.1.S1_at	Hypothetical protein	3.49
Mtr.48306.1.S1_at	Hypothetical protein	3.49
Mtr.5955.1.S1_at	Hypothetical protein	3.49
Mtr.33536.1.S1_at	Sugar transporter-like protein	3.49
Mtr.45548.1.S1_at	Hypothetical protein	3.49
Mtr.9872.1.S1_at	Hypothetical protein	3.49
Mtr.37899.1.S1_at	Hypothetical protein	3.49
Mtr.34150.1.S1_at	Hypothetical protein	3.48
Mtr.39206.1.S1_at	Hypothetical protein	3.48
Mtr.29990.1.S1_at	MYB-related protein	3.48
Mtr.17531.1.S1_at	Hypothetical protein	3.48
Msa.690.1.S1_at	Hypothetical protein	3.48
Mtr.49199.1.S1_at	Hypothetical protein	3.48
Mtr.11804.1.S1_s_at	Hypothetical protein	3.48
Mtr.8797.1.S1_at	60S ribosomal protein L34	3.48
Mtr.44619.1.S1_at	Hypothetical protein	3.48
Mtr.33204.1.S1_at	Hypothetical protein	3.48
Mtr.39641.1.S1_at	Protein kinase	3.48
Mtr.35791.1.S1_s_at	Light-induced protein	3.48
Mtr.7449.1.S1_at	GDSL-motif lipase/hydrolase-like protein	3.47
Mtr.394.1.S1_s_at	Pre-mRNA splicing factor SF2	3.47
Msa.432.1.S1_at	Hypothetical protein	3.47
Mtr.34926.1.S1_s_at	Hypothetical protein	3.47
Mtr.51725.1.S1_at	Hypothetical protein	3.47
Mtr.37513.1.S1_at	RelA-SpoT like protein	3.47
Mtr.48411.1.S1_s_at	Beta-galactosidase	3.47
Mtr.9399.1.S1_at	CDPK-related protein kinase	3.47
Mtr.39404.1.S1_at	Hypothetical protein	3.47
Msa.1723.1.S1_at	Hypothetical protein	3.46
Mtr.39464.1.S1_s_at	Hypothetical protein	3.46
Mtr.40132.1.S1_at	Peroxidase1A	3.46
Mtr.50087.1.S1_at	Hypothetical protein	3.46
Mtr.34957.1.S1_at	Beta-glucosidase	3.46
Mtr.13078.1.S1_s_at	Hypothetical protein	3.46
Mtr.46539.1.S1_at	bZIP DNA-binding protein	3.45
Mtr.4600.1.S1_at	Guanylyl cyclase alpha	3.45
Mtr.9552.1.S1_at	Protein kinase	3.45
Mtr.41653.1.S1_at	Mitochondrial phosphate translocator	3.45
Msa.3090.1.S1_at	Hypothetical protein	3.45
Mtr.21812.1.S1_s_at	Hypothetical protein	3.45
Mtr.24633.1.S1_at	Hypothetical protein	3.45
Mtr.41934.1.S1_at	Pupal cuticle protein	3.45
Mtr.44172.1.S1_at	Hypothetical protein	3.45
Mtr.28723.1.S1_at	Hypothetical protein	3.45
Mtr.47542.1.S1_at	Hydrolase, alpha/beta fold family	3.45
Msa.2283.1.S1_at	Hypothetical protein	3.44
Mtr.12417.1.S1_s_at	Hypothetical protein	3.44
Mtr.5557.1.S1_at	Hypothetical protein	3.44
Mtr.44735.1.S1_at	Hypothetical protein	3.44

Mtr.39304.1.S1_at	Hypothetical protein	3.44
Mtr.8782.1.S1_at	Hypothetical protein	3.44
Mtr.44964.1.S1_at	Hypothetical protein	3.44
Mtr.18004.1.S1_at	Cwf15/Cwc15 cell cycle control protein	3.44
Mtr.37413.1.S1_s_at	Chalcone-flavonone isomerase	3.44
Mtr.40096.1.S1_at	ER6 protein	3.44
Mtr.40417.1.S1_at	Hypothetical protein	3.43
Mtr.7249.1.S1_at	N-hydroxycinnamoyl/benzoyltransferase-like protein	3.43
Mtr.6589.1.S1_at	Mitochondrial solute carrier protein	3.43
Mtr.1853.1.S1_at	Hypothetical protein	3.43
Mtr.41180.1.S1_at	Hypothetical protein	3.43
Mtr.32998.1.S1_s_at	MtN3	3.43
Mtr.5996.1.S1_at	Hypothetical protein	3.43
Mtr.7647.1.S1_at	EF-1-alpha-related GTP-binding protein	3.43
Mtr.8957.1.S1_at	Hypothetical protein	3.43
Mtr.5069.1.S1_at	MYB transcription factor	3.42
Mtr.48412.1.S1_s_at	Beta-galactosidase	3.42
Mtr.12415.1.S1_at	Hypothetical protein	3.42
Mtr.27828.1.S1_at	Phospholipase D delta isoform 1a	3.42
Msa.591.1.S1_at	Hypothetical protein	3.42
Mtr.29585.1.S1_at	Hypothetical protein	3.42
Mtr.6936.1.S1_at	Mitogen-activated protein kinase 7	3.42
Mtr.18691.1.S1_s_at	Hypothetical protein	3.42
Mtr.1533.1.S1_at	Hypothetical protein	3.41
Mtr.9940.1.S1_at	Hypothetical protein	3.41
Mtr.11250.1.S1_at	Disease resistance protein	3.41
Mtr.691.1.S1_at	Hypothetical protein	3.41
Mtr.51701.1.S1_s_at	Cyclin-like F-box protein	3.41
Mtr.10508.1.S1_at	Mitosis inhibitor protein kinase wee1	3.41
Mtr.35305.1.S1_at	Hypothetical protein	3.40
Mtr.40185.1.S1_at	FtsH-like protein	3.40
Mtr.43451.1.S1_at	Adenosine monophosphate binding protein 7	3.40
Mtr.4322.1.S1_at	Cytochrome oxidase subunit III	3.40
Mtr.37960.1.S1_at	PRL1-associated protein	3.40
Mtr.33159.1.S1_at	Hypothetical protein	3.40
Mtr.38936.1.S1_at	Acyl-CoA oxidase	3.40
Mtr.5527.1.S1_at	PHD zinc finger protein	3.39
Mtr.37580.1.S1_at	Brix domain containing protein 1	3.39
Mtr.43936.1.S1_at	Ser/Thr protein kinase	3.39
Mtr.40054.1.S1_at	J8-like protein	3.39
Mtr.41945.1.S1_at	Hypothetical protein	3.39
Mtr.49019.1.S1_at	Glycoside hydrolase	3.39
Msa.277.1.S1_at	Hypothetical protein	3.39
Msa.3160.1.S1_at	Hypothetical protein	3.38
Mtr.2361.1.S1_s_at	MAP kinase activating protein	3.38
Mtr.31259.1.S1_s_at	Plasma membrane intrinsic polypeptide	3.38
Mtr.6345.1.S1_at	DnaJ-like protein	3.38
Mtr.43957.1.S1_s_at	Peroxisomal copper-containing amine oxidase	3.38
Mtr.39253.1.S1_at	Hypothetical protein	3.38
Mtr.41678.1.S1_at	Hypothetical protein	3.38
Mtr.5174.1.S1_at	Hypothetical protein	3.38
Mtr.40474.1.S1_at	Ribosomal protein 30S	3.38
Mtr.38510.1.S1_at	Hypothetical protein	3.38
Mtr.41732.1.S1_at	Hypothetical protein	3.38

Mtr.5164.1.S1_at	Hypothetical protein	3.37
Mtr.37502.1.S1_at	Dermal glycoprotein	3.37
Mtr.15028.1.S1_at	Transketolase	3.37
Mtr.39546.1.S1_at	Hypothetical protein	3.37
Mtr.48875.1.S1_s_at	Hypothetical protein	3.37
Mtr.39626.1.S1_at	MAP kinase activating protein	3.36
Mtr.12264.1.S1_s_at	Cytochrome P450 monooxygenase	3.36
Mtr.42399.1.S1_at	Type IIA calcium ATPase	3.36
Mtr.39049.1.S1_at	WD40-repeat protein	3.36
Mtr.37798.1.S1_at	Hypothetical protein	3.36
Msa.1774.1.S1_at	Hypothetical protein	3.36
Mtr.39873.1.S1_s_at	Myb-like protein	3.36
Mtr.49932.1.S1_at	Ankyrin	3.36
Mtr.45488.1.S1_at	Calmodulin-binding protein	3.36
Mtr.12917.1.S1_at	Jasmonic acid 2	3.36
Mtr.7126.1.S1_s_at	Bat3 protein	3.35
Mtr.49671.1.S1_at	Hypothetical protein	3.35
Mtr.8978.1.S1_at	Hypothetical protein	3.35
Mtr.42930.1.S1_at	Hypothetical protein	3.35
Mtr.12906.1.S1_at	Glutamate decarboxylase 4a	3.35
Mtr.34601.1.S1_s_at	SOS2-like protein kinase	3.35
Mtr.38607.1.S1_at	Hypothetical protein	3.35
Mtr.6016.1.S1_at	Hypothetical protein	3.35
Mtr.781.1.S1_at	SKP1 interacting partner 1	3.35
Mtr.9656.1.S1_at	Response regulator protein	3.35
Mtr.9221.1.S1_at	Salicylic acid glucosyltransferase	3.35
Mtr.28490.1.S1_at	Hypothetical protein	3.35
Mtr.5072.1.S1_s_at	Hypothetical protein	3.35
Mtr.13558.1.S1_at	Beta-galactosidase	3.34
Mtr.2544.1.S1_s_at	Hypothetical protein	3.34
Mtr.38691.1.S1_s_at	Hypothetical protein	3.34
Mtr.44967.1.S1_at	Hypothetical protein	3.34
Mtr.11984.1.S1_s_at	Hypothetical protein	3.33
Mtr.37833.1.S1_at	Hypothetical protein	3.33
Mtr.27313.1.S1_at	Receptor kinase-like protein	3.33
Mtr.16505.1.S1_at	Aldehyde dehydrogenase	3.33
Mtr.50478.1.S1_at	Phenylalanine/histidine ammonia-lyase	3.33
Mtr.3533.1.S1_at	Hypothetical protein	3.33
Mtr.31759.1.S1_at	Homeobox-leucine zipper protein	3.33
Mtr.11772.1.S1_at	Beta-galactosidase	3.33
Mtr.52065.1.S1_at	WD-40 repeat protein	3.33
Mtr.44059.1.S1_at	SAR DNA-binding protein-1	3.32
Mtr.40101.1.S1_s_at	40S ribosomal protein S6	3.32
Mtr.14280.1.S1_at	Thioredoxin-related protein	3.32
Mtr.5567.1.S1_at	Hypothetical protein	3.32
Mtr.35717.1.S1_at	Sugar transporter	3.32
Mtr.9201.1.S1_at	Salt-induced AAA-Type ATPase	3.32
Mtr.14428.1.S1_at	Naringenin-chalcone synthase	3.32
Mtr.377.1.S1_s_at	Hypothetical protein	3.32
Mtr.2859.1.S1_at	Disease resistance protein	3.32
Mtr.12614.1.S1_at	Resistance protein MG23	3.32
Mtr.43924.1.S1_at	Glutaredoxin-related protein	3.32
Mtr.386.1.S1_at	D-xylose proton-symporter	3.32
Mtr.41654.1.S1_at	Single-strand DNA binding protein	3.32

Mtr.4790.1.S1_at	Hypothetical protein	3.32
Mtr.15172.1.S1_at	Leucine-rich repeat protein	3.31
Mtr.40406.1.S1_at	Hypothetical protein	3.31
Mtr.28378.1.S1_at	Hypothetical protein	3.31
Mtr.38381.1.S1_at	Hypothetical protein	3.31
Mtr.12085.1.S1_at	Serine/threonine-protein kinase-like protein, partial (23%)	3.31
Mtr.32685.1.S1_at	Hypothetical protein	3.31
Mtr.39867.1.S1_at	Hypothetical protein	3.31
Mtr.14111.1.S1_at	Plant lipid transfer/seed storage/trypsin-alpha amylase inhibitor	3.31
Mtr.8962.1.S1_at	Hypothetical protein	3.31
Mtr.2811.1.S1_at	Hypothetical protein	3.31
Mtr.45462.1.S1_at	Monosaccharide transporter	3.30
Mtr.41518.1.S1_at	Hypothetical protein	3.30
Mtr.39656.1.S1_s_at	Hypothetical protein	3.30
Mtr.36256.1.S1_at	Hypothetical protein	3.30
Mtr.33189.1.S1_s_at	AML1	3.30
Mtr.45278.1.S1_s_at	Hypothetical protein	3.30
Mtr.52180.1.S1_at	Zn-finger, C2H2 type	3.30
Mtr.16016.1.S1_at	Hypothetical protein	3.30
Mtr.28463.1.S1_at	Myosin-like protein	3.29
Mtr.12698.1.S1_at	Hypothetical protein	3.29
Mtr.11251.1.S1_at	Protein integral membrane protein	3.29
Mtr.43601.1.S1_at	50S ribosomal protein L3-2	3.29
Mtr.43669.1.S1_at	Dolichyl-phosphate beta-glucosyltransferase	3.29
Mtr.44404.1.S1_s_at	Hypothetical protein	3.29
Mtr.4897.1.S1_at	Hypothetical protein	3.29
Msa.2526.1.S1_s_at	Hypothetical protein	3.29
Mtr.44920.1.S1_at	FtsH protease (VAR2)	3.29
Mtr.13765.1.S1_at	Sphingosine-1-phosphate lyase	3.29
Msa.3057.1.S1_s_at	Hypothetical protein	3.28
Mtr.40053.1.S1_at	Serine/threonine protein kinase	3.28
Mtr.44647.1.S1_at	DNA/RNA binding protein	3.28
Mtr.10634.1.S1_at	MONOSACCHARIDE cotransporter	3.28
Mtr.32004.1.S1_at	Pelota-like protein	3.28
Mtr.35786.1.S1_at	Hypothetical protein	3.28
Msa.3020.1.S1_at	Hypothetical protein	3.28
Mtr.11064.1.S1_at	DNA-directed RNA polymerase	3.28
Mtr.13840.1.S1_at	MYB transcription factor	3.28
Mtr.5738.1.S1_s_at	Hypothetical protein	3.28
Mtr.50347.1.S1_at	ATP-dependent helicase	3.28
Mtr.31406.1.S1_x_at	Isoflavonoid glucosyltransferase	3.27
Mtr.43217.1.S1_at	Hypothetical protein	3.27
Mtr.7377.1.S1_at	Germin-like protein	3.27
Mtr.43084.1.S1_at	Hypothetical protein	3.27
Mtr.6988.1.S1_at	Hypothetical protein	3.27
Mtr.25306.1.S1_at	Prolyl 4-hydroxylase	3.27
Mtr.12901.1.S1_at	Hypothetical protein	3.27
Msa.3111.1.S1_x_at	Hypothetical protein	3.27
Mtr.42711.1.S1_at	Scarecrow-like 1 protein	3.27
Mtr.10618.1.S1_x_at	Hypothetical protein	3.27
Mtr.10594.1.S1_at	Xyloglucan endo-1,4-beta-D-glucanase	3.27
Msa.1937.1.S1_at	Hypothetical protein	3.27
Mtr.17499.1.S1_at	tRNA intron endonuclease	3.27
Mtr.39534.1.S1_at	Hypothetical protein	3.27

Mtr.17501.1.S1_at	Pectin lyase	3.27
Mtr.42712.1.S1_at	Mitochondrial carrier-like protein	3.27
Mtr.35896.1.S1_at	Chitinase	3.26
Mtr.3520.1.S1_s_at	Exo-beta-glucanase	3.26
Mtr.12152.1.S1_at	Protein phosphatase 2C	3.26
Msa.1472.1.S1_at	Hypothetical protein	3.26
Mtr.44947.1.S1_at	TFIIE-alpha 2	3.26
Mtr.38799.1.S1_at	Cytokinin oxidase	3.26
Mtr.29867.1.S1_at	Hypothetical protein	3.26
Mtr.42126.1.S1_at	DNA-binding protein	3.26
Mtr.42101.1.S1_at	Hypothetical protein	3.26
Mtr.43608.1.S1_at	rRNA processing protein EBP2	3.25
Msa.1290.1.S1_at	Hypothetical protein	3.25
Mtr.11031.1.S1_s_at	Hypothetical protein	3.25
Mtr.17368.1.S1_at	Galactose oxidase	3.25
Mtr.9119.1.S1_at	Phloem-specific lectin-like protein	3.25
Mtr.41067.1.S1_at	Hypothetical protein	3.25
Mtr.37573.1.S1_at	ADR6 protein	3.25
Mtr.29891.1.S1_at	Hypothetical protein	3.25
Mtr.6418.1.S1_s_at	Hypothetical protein	3.25
Mtr.50388.1.S1_at	Hypothetical protein	3.24
Mtr.42915.1.S1_at	Hypothetical protein	3.24
Mtr.50504.1.S1_at	Protein kinase	3.24
Mtr.11078.1.S1_at	Hypothetical protein	3.24
Mtr.24721.1.S1_at	Heat shock transcription factor	3.24
Mtr.51222.1.S1_at	Disease resistance protein	3.24
Mtr.35836.1.S1_at	Hypothetical protein	3.24
Mtr.35925.1.S1_at	Hypothetical protein	3.24
Mtr.12432.1.S1_at	NADH-dependent glutamate synthase	3.24
Mtr.48971.1.S1_at	Cytochrome oxidase	3.24
Mtr.32912.1.S1_at	Hypothetical protein	3.23
Mtr.51148.1.S1_at	Transferase	3.23
Msa.464.1.S1_at	Hypothetical protein	3.23
Mtr.32496.1.S1_at	Hypothetical protein	3.23
Mtr.50156.1.S1_at	Hypothetical protein	3.23
Mtr.12913.1.S1_at	Hypothetical protein	3.23
Mtr.4879.1.S1_at	Methylesterase	3.23
Mtr.1082.1.S1_at	Aldose 1-epimerase	3.23
Mtr.7570.1.S1_at	Hypothetical protein	3.23
Mtr.39020.1.S1_at	Ethylene receptor homolog	3.23
Msa.1153.1.S1_at	Hypothetical protein	3.23
Mtr.7350.1.S1_at	Hypothetical protein	3.23
Mtr.48736.1.S1_at	Disease resistance protein	3.23
Mtr.9830.1.S1_at	Expansin	3.23
Mtr.27185.1.S1_at	Cytochrome P450	3.23
Mtr.30173.1.S1_at	WRKY transcription factor	3.23
Mtr.49556.1.S1_at	RNA polymerase Rpb1	3.23
Mtr.43011.1.S1_at	Ubiquitin-conjugating enzyme E2	3.23
Mtr.44341.1.S1_at	BRASSINOSTEROID INSENSITIVE 1-associated receptor kinase 1	3.23
Mtr.1526.1.S1_at	Hypothetical protein	3.23
Mtr.27676.1.S1_x_at	Hypothetical protein	3.23
Mtr.50225.1.S1_at	Hypothetical protein	3.22
Mtr.3061.1.S1_s_at	Hypothetical protein	3.22
Mtr.6696.1.S1_at	Hypothetical protein	3.22

Mtr.43175.1.S1_s_at	Hypothetical protein	3.22
Mtr.41694.1.S1_s_at	AP2 domain transcription factor	3.22
Mtr.42375.1.S1_at	Hypothetical protein	3.22
Mtr.12859.1.S1_at	Hypothetical protein	3.21
Mtr.35758.1.S1_at	NONA protein	3.21
Mtr.45586.1.S1_at	Hypothetical protein	3.21
Mtr.17492.1.S1_at	Hypothetical protein	3.21
Mtr.39500.1.S1_at	Avr9/Cf-9 rapidly elicited protein	3.21
Mtr.28524.1.S1_at	Hypothetical protein	3.21
Mtr.38609.1.S1_at	Hypothetical protein	3.21
Mtr.37250.1.S1_at	Hypothetical protein	3.21
Mtr.42141.1.S1_s_at	Class III peroxidase	3.21
Mtr.33923.1.S1_at	TRANSMEMBRANE PROTEIN	3.21
Mtr.4454.1.S1_at	Hypothetical protein	3.20
Mtr.4734.1.S1_at	Hypothetical protein	3.20
Mtr.12226.1.S1_s_at	Hypothetical protein	3.20
Mtr.52344.1.S1_at	Aminotransferase	3.20
Mtr.42391.1.S1_at	Hypothetical protein	3.20
Mtr.28454.1.S1_at	Hypothetical protein	3.20
Mtr.10717.1.S1_at	Twin LOV protein 1	3.20
Mtr.16726.1.S1_at	Leucine-rich repeat protein	3.20
Mtr.9938.1.S1_at	Dihydrodipicolinate synthase	3.20
Mtr.40911.1.S1_s_at	Cationic peroxidase 2	3.20
Mtr.43953.1.S1_at	Hypothetical protein	3.20
Mtr.9077.1.S1_at	Hypothetical protein	3.20
Mtr.787.1.S1_at	Hypothetical protein	3.20
Mtr.19608.1.S1_s_at	Hypothetical protein	3.20
Mtr.17645.1.S1_at	Hypothetical protein	3.20
Mtr.13479.1.S1_at	Transducin-like protein	3.19
Mtr.37977.1.S1_at	Cyclophilin	3.19
Mtr.45405.1.S1_at	Hypothetical protein	3.19
Mtr.9120.1.S1_at	Hypothetical protein	3.19
Mtr.38518.1.S1_at	Hypothetical protein	3.19
Mtr.18420.1.S1_at	Adenylate cyclase	3.19
Mtr.288.1.S1_at	Hypothetical protein	3.19
Msa.1280.1.S1_at	Hypothetical protein	3.19
Mtr.47143.1.S1_s_at	Brassinosteroid-regulated protein BRU1	3.19
Mtr.2952.1.S1_at	Branched-chain alpha keto-acid dehydrogenase E1 alpha subunit	3.19
Mtr.36196.1.S1_s_at	Lipase	3.19
Mtr.13483.1.S1_at	13-lipoxygenase	3.19
Mtr.49460.1.S1_s_at	RNA-binding region RNP-1	3.19
Mtr.48899.1.S1_at	Ferritin/ribonucleotide reductase	3.19
Mtr.10253.1.S1_at	Cellulose synthase	3.19
Mtr.5245.1.S1_at	Hypothetical protein	3.19
Mtr.10408.1.S1_at	Ferrochelatase I	3.19
Mtr.38325.1.S1_at	Sphingosine-1-phosphate lyase	3.19
Mtr.40193.1.S1_at	Hypothetical protein	3.18
Mtr.25749.1.S1_at	Copper amine oxidase	3.18
Mtr.34833.1.S1_s_at	Hypothetical protein	3.18
Mtr.2087.1.S1_at	Hypothetical protein	3.18
Mtr.32750.1.S1_at	Hypothetical protein	3.18
Mtr.46966.1.S1_s_at	Wound-induced GSK-3-like protein	3.18
Mtr.10474.1.S1_at	Autophagy protein 8	3.18
Mtr.31085.1.S1_at	Hypothetical protein	3.18

Mtr.9783.1.S1_at	Zinc finger-like protein	3.18
Mtr.42879.1.S1_at	Light-induced protein	3.18
Mtr.37801.1.S1_at	Protein kinase	3.18
Mtr.41884.1.S1_at	Mei2-like protein	3.17
Mtr.7199.1.S1_at	Hypothetical protein	3.17
Mtr.9218.1.S1_at	Hypothetical protein	3.17
Mtr.9429.1.S1_at	Transcription factor RAU1	3.17
Mtr.45108.1.S1_at	ABC-type cobalamin/Fe3+-siderophores transport	3.17
Mtr.49142.1.S1_at	Hypothetical protein	3.17
Mtr.23686.1.S1_s_at	DNA-directed RNA polymerase	3.17
Mtr.11323.1.S1_at	F-box protein	3.17
Mtr.45354.1.S1_at	Whitefly-induced gp91-phox	3.16
Msa.1740.1.S1_at	Hypothetical protein	3.16
Mtr.39437.1.S1_at	MRPS17 protein	3.16
Mtr.28538.1.S1_at	Hypothetical protein	3.16
Mtr.33245.1.S1_at	Hypothetical protein	3.16
Mtr.1563.1.S1_at	SRG1 protein	3.16
Mtr.41373.1.S1_at	Phi-1-like protein	3.16
Mtr.50948.1.S1_at	Hypothetical protein	3.16
Mtr.43688.1.S1_at	Hypothetical protein	3.16
Mtr.29288.1.S1_x_at	Hypothetical protein	3.16
Msa.1670.1.S1_at	Hypothetical protein	3.15
Mtr.32311.1.S1_at	Dynein light chain 1	3.15
Mtr.37171.1.S1_at	Hypothetical protein	3.15
Mtr.43611.1.S1_at	WRKY transcription factor 65	3.15
Mtr.15702.1.S1_s_at	Zn-finger protein	3.15
Mtr.13158.1.S1_at	S-receptor kinase	3.15
Mtr.50884.1.S1_s_at	Hypothetical protein	3.15
Mtr.35604.1.S1_at	Lipase family protein	3.15
Msa.3101.1.S1_at	Hypothetical protein	3.15
Mtr.37419.1.S1_at	NuM1 protein	3.15
Mtr.3292.1.S1_at	Hypothetical protein	3.15
Mtr.11560.1.S1_s_at	ADP/ATP translocase-like protein	3.15
Mtr.38140.1.S1_at	Zinc finger protein	3.15
Mtr.40007.1.S1_at	Hypothetical protein	3.15
Mtr.10462.1.S1_at	Non-symbiotic hemoglobin 1	3.14
Mtr.41454.1.S1_at	Farnesylated protein	3.14
Mtr.45545.1.S1_at	Hypothetical protein	3.14
Mtr.41247.1.S1_at	Hypothetical protein	3.14
Mtr.25327.1.S1_at	Hypothetical protein	3.14
Mtr.12589.1.S1_at	Acetyl-CoA carboxylase	3.14
Mtr.41270.1.S1_at	Beta-adaptin-like protein A	3.14
Mtr.6811.1.S1_s_at	Glutathione S-transferase GST 17	3.14
Mtr.40736.1.S1_s_at	STAM binding protein	3.14
Mtr.40569.1.S1_at	Alpha-mannosidase	3.14
Mtr.24032.1.S1_at	Hypothetical protein	3.14
Mtr.32884.1.S1_s_at	Jasmonic acid 2	3.14
Mtr.44195.1.S1_at	Hypothetical protein	3.14
Mtr.10342.1.S1_at	Xyloglucan endotransglucosylase/hydrolase protein	3.13
Mtr.38956.1.S1_at	Hypothetical protein	3.13
Mtr.5980.1.S1_at	Hypothetical protein	3.13
Mtr.17399.1.S1_at	Peptidylprolyl isomerase, FKBP-type	3.13
Msa.1761.1.S1_at	Hypothetical protein	3.13
Mtr.13482.1.S1_at	Ceramide kinase	3.13

Mtr.48831.1.S1_at	Peptidase aspartic	3.13
Mtr.594.1.S1_s_at	Functional candidate resistance protein KR1	3.12
Mtr.10407.1.S1_at	Ferrochelatase II	3.12
Mtr.19308.1.S1_s_at	Cyclin-like F-box	3.12
Mtr.7061.1.S1_at	Hypothetical protein	3.12
Mtr.32008.1.S1_at	Hypothetical protein	3.12
Mtr.22123.1.S1_at	Hypothetical protein	3.12
Mtr.10224.1.S1_at	MYB-like DNA-binding domain protein	3.12
Mtr.4893.1.S1_at	Hypothetical protein	3.12
Mtr.14125.1.S1_at	Phytochrome	3.12
Mtr.9754.1.S1_at	Hypothetical protein	3.12
Mtr.45896.1.S1_at	Translation initiation factor IF5	3.12
Mtr.20676.1.S1_at	Hypothetical protein	3.12
Mtr.20265.1.S1_at	Hypothetical protein	3.12
Mtr.37283.1.S1_s_at	Pathogen-inducible alpha-dioxygenase	3.12
Mtr.44105.1.S1_at	Nucleolar RNA-binding Nop10p-like protein	3.12
Mtr.43799.1.S1_at	Hypothetical protein	3.12
Mtr.1356.1.S1_s_at	Hypothetical protein	3.11
Mtr.12000.1.S1_at	Hypothetical protein	3.11
Mtr.39348.1.S1_at	F-box protein ORE9	3.11
Msa.1709.1.S1_at	Hypothetical protein	3.11
Mtr.30161.1.S1_s_at	Basic/leucine zipper protein	3.11
Mtr.49129.1.S1_s_at	Pumilio/Puf RNA-binding protein	3.11
Mtr.1511.1.S1_at	UDP-glucosyl transferase	3.11
Msa.959.1.S1_at	Hypothetical protein	3.11
Mtr.4918.1.S1_at	S-receptor kinase	3.11
Mtr.20784.1.S1_s_at	Lipase	3.10
Mtr.9913.1.S1_s_at	Hypothetical protein	3.10
Mtr.40725.1.S1_at	Mannose-6-phosphate isomerase	3.10
Mtr.35057.1.S1_at	1-aminocyclopropane-1-carboxylate synthase 1	3.10
Mtr.14252.1.S1_at	Ribosomal protein L25	3.10
Mtr.33493.1.S1_at	Hypothetical protein	3.10
Mtr.42251.1.S1_at	Hypothetical protein	3.10
Mtr.27351.1.S1_at	Protein kinase	3.10
Mtr.18677.1.S1_s_at	Hypothetical protein	3.09
Mtr.39529.1.S1_at	Tetratricopeptide repeat (TPR)-containing protein	3.09
Mtr.43227.1.S1_at	52 kDa Ro protein	3.09
Mtr.15268.1.S1_at	ADP-ribosylation factor	3.09
Mtr.24827.1.S1_at	Hypothetical protein	3.09
Mtr.5689.1.S1_at	Syntaxin-related protein	3.09
Mtr.11873.1.S1_at	Ethylene-overproduction protein	3.09
Mtr.10732.1.S1_at	Hypothetical protein	3.09
Mtr.46823.1.S1_at	Calmodulin-binding protein	3.09
Mtr.34611.1.S1_at	ZF-HD homeobox protein	3.09
Mtr.38531.1.S1_at	Hypothetical protein	3.08
Mtr.39976.1.S1_s_at	AAA-type ATPase	3.08
Msa.1686.1.S1_s_at	Hypothetical protein	3.08
Mtr.43257.1.S1_at	NAC-domain protein	3.08
Mtr.40988.1.S1_at	Hypothetical protein	3.08
Mtr.19570.1.S1_at	Pathogenesis-related transcriptional factor	3.08
Mtr.40598.1.S1_at	Hypothetical protein	3.08
Mtr.43265.1.S1_at	Hypothetical protein	3.08
Mtr.42431.1.S1_at	Hypothetical protein	3.08
Mtr.6559.1.S1_at	Nucleoporin	3.08

Mtr.9090.1.S1_at	Hypothetical protein	3.08
Mtr.48818.1.S1_at	bZIP transcription factor	3.08
Mtr.17250.1.S1_at	Spectrin repeat protein	3.08
Mtr.21025.1.S1_at	Esterase/lipase/thioesterase	3.08
Mtr.33257.1.S1_s_at	Stromal membrane-associated protein	3.08
Mtr.38328.1.S1_at	Phosphatidic acid phosphatase alpha	3.08
Mtr.8932.1.S1_at	Geranylgeranylated protein	3.08
Mtr.28443.1.S1_s_at	Hypothetical protein	3.08
Mtr.20407.1.S1_at	Hypothetical protein	3.08
Mtr.44887.1.S1_at	Hypothetical protein	3.07
Mtr.4658.1.S1_at	Hypothetical protein	3.07
Mtr.41303.1.S1_at	Hypothetical protein	3.07
Mtr.17083.1.S1_at	Light chain 3 (LC3)	3.07
Mtr.43104.1.S1_at	1-aminocyclopropanecarboxylic acid oxidase	3.07
Mtr.22601.1.S1_s_at	Endoxyloglucan transferase	3.07
Mtr.37225.1.S1_at	Adenine nucleotide translocator	3.07
Mtr.32578.1.S1_at	Hypothetical protein	3.07
Mtr.10793.1.S1_at	STAM binding protein	3.07
Mtr.39860.1.S1_at	Hypothetical protein	3.07
Mtr.42237.1.S1_at	Hypothetical protein	3.07
Mtr.38213.1.S1_at	Hypothetical protein	3.07
Mtr.11587.1.S1_at	Hypothetical protein	3.07
Mtr.49745.1.S1_s_at	Tyrosine specific protein phosphatase	3.07
Mtr.15535.1.S1_at	Serine/threonine protein kinase	3.06
Mtr.30324.1.S1_at	Hypothetical protein	3.06
Mtr.6307.1.S1_at	Drm3	3.06
Mtr.13157.1.S1_at	Hypothetical protein	3.06
Mtr.10803.1.S1_at	Small nuclear riboprotein	3.06
Mtr.16958.1.S1_s_at	Proteinase inhibitor I9	3.06
Msa.2767.1.S1_at	Hypothetical protein	3.06
Mtr.38773.1.S1_at	Protein kinase	3.06
Mtr.30756.1.S1_s_at	Hypothetical protein	3.06
Mtr.38660.1.S1_at	Hypothetical protein	3.06
Mtr.41990.1.S1_at	Nodulin-like protein	3.06
Msa.2923.1.S1_at	Hypothetical protein	3.06
Mtr.14271.1.S1_at	Calcium-binding EF-hand	3.06
Mtr.20117.1.S1_s_at	Peptidyl-prolyl cis-trans isomerase, cyclophilin type	3.06
Msa.2396.1.S1_at	Hypothetical protein	3.06
Mtr.40703.1.S1_at	Hypothetical protein	3.06
Mtr.39780.1.S1_at	Hypothetical protein	3.06
Mtr.18911.1.S1_s_at	Serine/threonine protein kinase	3.06
Mtr.8301.1.S1_at	BRI1-KD interacting protein	3.06
Mtr.2526.1.S1_at	Hypothetical protein	3.06
Mtr.39119.1.S1_at	Timing of CAB expression 1	3.06
Mtr.18027.1.S1_at	Ankyrin	3.05
Mtr.21406.1.S1_s_at	Ankyrin	3.05
Mtr.33080.1.S1_at	Hypothetical protein	3.05
Mtr.8828.1.S1_at	Cytochrome P450	3.05
Mtr.33207.1.S1_at	Hypothetical protein	3.05
Mtr.44938.1.S1_at	Hypothetical protein	3.05
Mtr.7077.1.S1_at	Amino acid permease AAP4	3.05
Msa.3073.1.S1_s_at	Hypothetical protein	3.05
Mtr.6755.1.S1_at	Xanthine dehydrogenase 1	3.05
Mtr.41453.1.S1_at	Hypothetical protein	3.04

Mtr.10204.1.S1_at	NADH dehydrogenase subunit 4L	3.04
Mtr.50869.1.S1_at	Hypothetical protein	3.04
Mtr.5080.1.S1_s_at	Extensin (Class I)	3.04
Mtr.9076.1.S1_at	Hypothetical protein	3.04
Mtr.43853.1.S1_at	Hypothetical protein	3.04
Mtr.18418.1.S1_at	Ankyrin	3.04
Mtr.49055.1.S1_at	Hypothetical protein	3.04
Mtr.19888.1.S1_at	Glycosyl hydrolases family 17	3.04
Mtr.7491.1.S1_at	Hypothetical protein	3.04
Mtr.42683.1.S1_at	Acyl-activating enzyme 18	3.03
Mtr.46591.1.S1_at	Hypothetical protein	3.03
Mtr.12226.1.S1_x_at	Hypothetical protein	3.03
Mtr.11293.1.S1_at	Polygalacturonase inhibitor-like protein	3.03
Mtr.39368.1.S1_at	Ethylene receptor homolog	3.03
Mtr.19285.1.S1_s_at	Hypothetical protein	3.03
Mtr.35059.1.S1_at	Hypothetical protein	3.03
Mtr.44445.1.S1_at	Syringolide-induced protein B13-1-9	3.03
Mtr.10144.1.S1_at	Disease resistance protein	3.03
Mtr.14274.1.S1_at	RNA-binding region RNP-1	3.03
Mtr.51890.1.S1_s_at	Proteinase inhibitor I9	3.03
Mtr.45295.1.S1_s_at	Hypothetical protein	3.03
Mtr.23763.1.S1_s_at	DEAH box helicase	3.03
Mtr.34681.1.S1_at	Hypothetical protein	3.03
Mtr.37748.1.S1_at	Defense-related protein	3.02
Mtr.42794.1.S1_at	Hypothetical protein	3.02
Mtr.44296.1.S1_at	Cellulose synthase-like protein D4	3.02
Mtr.44925.1.S1_at	Hypothetical protein	3.02
Mtr.24441.1.S1_s_at	Hypothetical protein	3.02
Mtr.27676.1.S1_at	Hypothetical protein	3.02
Mtr.25961.1.S1_s_at	Hypothetical protein	3.02
Mtr.7469.1.S1_at	Hypothetical protein	3.02
Mtr.42273.1.S1_at	Hypothetical protein	3.02
Mtr.41147.1.S1_at	Hypothetical protein	3.02
Mtr.37179.1.S1_at	Hypothetical protein	3.02
Mtr.23037.1.S1_at	Hypothetical protein	3.01
Mtr.44641.1.S1_at	F-box family protein	3.01
Mtr.5708.1.S1_at	RING zinc finger protein	3.01
Mtr.37622.1.S1_at	KH domain-containing protein	3.01
Mtr.11870.1.S1_at	ER66 protein	3.01
Msa.1300.1.S1_at	Hypothetical protein	3.01
Mtr.6549.1.S1_at	Hypothetical protein	3.01
Mtr.45921.1.S1_at	DNA-directed RNA polymerase	3.01
Mtr.35552.1.S1_at	Hypothetical protein	3.01
Mtr.26606.1.S1_at	Hypothetical protein	3.01
Mtr.4402.1.S1_x_at	Hypothetical protein	3.01
Mtr.13557.1.S1_at	Ethylene receptor homolog	3.01
Mtr.27159.1.S1_at	Hypothetical protein	3.01
Mtr.3548.1.S1_at	Hypothetical protein	3.01
Mtr.31135.1.S1_at	Hypothetical protein	3.01
Mtr.12791.1.S1_at	Hypothetical protein	3.01
Mtr.17167.1.S1_at	Methyladenine glycosylase	3.01
Mtr.20107.1.S1_at	Expansin	3.01
Mtr.13267.1.S1_at	Hypothetical protein	3.00
Mtr.12524.1.S1_at	Eukaryotic cap-binding protein	3.00

Mtr.36886.1.S1_at	Hypothetical protein	3.00
Mtr.12455.1.S1_at	Hypothetical protein	3.00
Mtr.12484.1.S1_at	Branched-chain-amino-acid aminotransferase 5	3.00

Supplementary Table S4. Downregulated genes at 24 hours after inoculation with soybean rust

Probesets	Target Description	Fold reduction
Mtr.26465.1.S1_s_at	Phosphoethanolamine N-methyltransferase	-865.11
Mtr.13982.1.S1_at	CDP-diacylglycerol-glycerol-3-phosphate 3-phosphatidyltransferase	-768.01
Mtr.35794.1.S1_s_at	Myo-inositol 2-phoshate synthase	-667.21
Mtr.20438.1.S1_at	O-methyltransferase	-566.21
Mtr.20321.1.S1_at	Plant lipid transfer/seed storage/trypsin-alpha amylase inhibitor	-545.29
Mtr.8618.1.S1_at	Cytochrome P450 monooxygenase CYP93D1	-537.70
Mtr.8427.1.S1_at	Lipoxygenase	-456.09
Mtr.1186.1.S1_s_at	Ferric reductase-like transmembrane component family	-449.36
Mtr.26601.1.S1_s_at	FRO1-like protein	-434.76
Mtr.42849.1.S1_at	Myo-inositol-1-phosphate synthase	-390.84
Mtr.26602.1.S1_s_at	NADPH oxidase	-368.11
Mtr.12069.1.S1_at	Phosphoethanolamine	-350.54
Mtr.43870.1.S1_at	FRO1-like protein; NADPH oxidas	-345.88
Mtr.44581.1.S1_at	Hypothetical protein	-343.94
Msa.2715.1.S1_at	Hypothetical protein	-315.40
Mtr.44812.1.S1_s_at	Extracellular calcium sensing receptor	-311.90
Mtr.13564.1.S1_s_at	Extracellular calcium sensing receptor	-294.11
Mtr.42858.1.S1_at	Hypothetical protein	-286.76
Mtr.10604.1.S1_at	Nitrate reductase	-281.26
Mtr.41746.1.S1_at	DnaJ domain family	-275.74
Mtr.12393.1.S1_at	Cinnamoyl-CoA reductase-like protein	-275.26
Mtr.12682.1.S1_at	Light-harvesting complex protein	-248.43
Mtr.44591.1.S1_at	Quinone oxidoreductase	-240.41
Mtr.9320.1.S1_at	Hypothetical protein	-238.68
Mtr.19547.1.S1_s_at	Nucleoside phosphatase GDA1/CD39	-237.47
Mtr.11284.1.S1_at	Oxygen evolving complex protein	-233.39
Mtr.10369.1.S1_at	Chalcone synthase	-225.35
Mtr.14290.1.S1_at	Rhodanese-like protein	-224.58
Mtr.10456.1.S1_at	Early light inducible protein	-222.50
Msa.888.1.S1_at	Hypothetical protein	-220.26
Mtr.5348.1.S1_at	Hypothetical protein	-216.22
Mtr.41942.1.S1_at	Hypothetical protein	-210.45
Mtr.10298.1.S1_at	Photosystem II 22 kDa protein	-210.25
Mtr.44731.1.S1_at	Hypothetical protein	-209.63
Msa.1913.1.S1_at	Hypothetical protein	-192.35
Mtr.41828.1.S1_at	Thylakoid soluble phosphoprotein	-186.32
Mtr.9873.1.S1_at	Thioredoxin H2	-185.89
Mtr.13190.1.S1_at	Hypothetical protein	-184.82
Mtr.11110.1.S1_at	GCN5-related N-acetyltransferase (GNAT)	-181.75
Mtr.8476.1.S1_at	Plastidic aldolase	-177.94
Mtr.40245.1.S1_s_at	Early light inducible protein	-172.99
Mtr.49177.1.S1_at	Dienelactone hydrolase	-171.50
Mtr.10659.1.S1_s_at	Hypothetical protein	-171.49
Mtr.10450.1.S1_at	Hypothetical protein	-169.63
Msa.3125.1.S1_at	Hypothetical protein	-169.59
Mtr.46609.1.S1_at	UDP-glycosyltransferase	-164.63
Mtr.6704.1.S1_at	Hypothetical protein	-159.30
Mtr.50430.1.S1_at	Lipoxygenase	-157.13
Mtr.15442.1.S1_at	Hypothetical protein	-155.90
Mtr.52215.1.S1_at	Lipoxygenase	-155.36
Msa.888.1.S1_s_at	Hypothetical protein	-154.67
Mtr.40848.1.S1_at	Photosystem II protein X	-149.55

Mtr.8637.1.S1_at	Hypothetical protein	-147.41
Mtr.41463.1.S1_at	ADP-glucose pyrophosphorylase	-145.46
Mtr.19547.1.S1_at	Nucleoside phosphatase GDA1/CD39	-145.18
Mtr.35685.1.S1_at	Heat shock protein	-143.89
Mtr.8475.1.S1_s_at	Plastidic aldolase	-143.82
Mtr.4439.1.S1_s_at	Thioredoxin H2	-140.05
Mtr.40742.1.S1_s_at	Thylakoid membrane phosphoprotein 14 kDa	-139.95
Mtr.8651.1.S1_a_at	Dehydrin-like protein	-139.91
Mtr.12272.1.S1_at	Granule-bound glycogen	-139.18
Mtr.28774.1.S1_at	Anthocyanidin synthase	-137.01
Mtr.25935.1.S1_at	Auxin-induced protein 15A	-135.38
Mtr.51205.1.S1_at	Hypothetical protein	-132.51
Mtr.22257.1.S1_s_at	Hypothetical protein	-131.72
Mtr.34220.1.S1_s_at	Hypothetical protein	-129.88
Mtr.51510.1.S1_at	Hypothetical protein	-126.43
Mtr.38073.1.S1_at	Dihydroflavanol-4-reductase 1	-126.42
Mtr.38344.1.S1_at	Limonoid UDP-glucosyltransferase	-125.60
Mtr.8651.1.S1_at	Dehydrin-like protein	-125.37
Msa.3055.1.S1_at	Hypothetical protein	-125.04
Msa.3134.1.S1_at	Hypothetical protein	-124.47
Mtr.10457.1.S1_at	Early light inducible protein	-123.42
Mtr.40669.1.S1_at	FK506 binding protein 1	-123.05
Mtr.42982.1.S1_s_at	Carbonic anhydrase	-121.36
Mtr.26036.1.S1_s_at	3-ketoacyl-CoA synthase 1 (KCS1)	-121.23
Mtr.40719.1.S1_s_at	Tic62 protein	-120.83
Mtr.9735.1.S1_a_at	Tic62 protein	-119.81
Mtr.25935.1.S1_x_at	Auxin-induced protein 15A	-118.89
Msa.1004.1.S1_at	Hypothetical protein	-117.08
Mtr.9236.1.S1_at	ADP-glucose pyrophosphorylase	-116.73
Msa.1451.1.S1_at	Hypothetical protein	-116.24
Mtr.37363.1.S1_at	CPRD46 protein	-115.96
Msa.3075.1.S1_at	Hypothetical protein	-114.23
Mtr.10797.1.S1_at	Thylakoid membrane phosphoprotein 14 kDa	-114.10
Mtr.12687.1.S1_at	Hypothetical protein	-113.52
Mtr.19759.1.S1_at	Zinc-containing alcohol dehydrogenase	-112.15
Mtr.5628.1.S1_s_at	Lipoxygenase	-111.69
Mtr.32797.1.S1_s_at	Sphingolipid long chain base delta 8 desaturase	-109.08
Msa.2574.1.S1_at	Hypothetical protein	-107.23
Mtr.37533.1.S1_at	Fructose-1,6-bisphosphatase	-107.16
Mtr.9564.1.S1_at	FKBP like protein	-106.44
Mtr.43317.1.S1_s_at	Photosystem II core complex proteins psbY	-105.72
Mtr.16402.1.S1_at	NADPH HC toxin reductase	-105.64
Msa.1690.1.S1_at	Hypothetical protein	-105.20
Mtr.35240.1.S1_s_at	Xyloglucan endotransglycosylase hydrolase	-104.50
Mtr.7343.1.S1_at	ADP-glucose pyrophosphorylase	-104.36
Msa.1383.1.S1_at	Hypothetical protein	-103.70
Mtr.13242.1.S1_at	Magnesium chelatase	-103.31
Mtr.12273.1.S1_s_at	Ribulose 1,5-bisphosphate carboxylase small subunit	-103.16
Msa.2951.1.S1_at	Hypothetical protein	-102.66
Mtr.37632.1.S1_at	Tetrapyrrole-binding protein	-102.43
Mtr.39986.1.S1_at	Hypothetical protein	-97.15
Mtr.41786.1.S1_at	Hypothetical protein	-96.65
Mtr.31194.1.S1_at	Cytochrome P450	-95.86
Mtr.12160.1.S1_at	Mitochondrial carnitine/acylcarnitine carrier-like protein	-93.86

Mtr.24578.1.S1_s_at	Hypothetical protein	-93.05
Mtr.38769.1.S1_at	Steroid 5alpha-reductase	-92.25
Mtr.35696.1.S1_s_at	Hypothetical protein	-90.82
Mtr.42842.1.S1_s_at	Ribulose 1,5-bisphosphate carboxylase small subunit	-90.80
Mtr.43089.1.S1_at	Seed maturation protein LEA 4	-90.57
Mtr.40432.1.S1_at	Sedoheptulose-1,7-bisphosphatase	-89.79
Msa.3155.1.S1_at	Hypothetical protein	-89.20
Mtr.26465.1.S1_at	Phosphoethanolamine N-methyltransferase	-88.57
Mtr.11283.1.S1_at	Hypothetical protein	-87.53
Mtr.22060.1.S1_at	Alanine aminotransferase	-87.50
Mtr.24034.1.S1_s_at	Hypothetical protein	-87.26
Mtr.6142.1.S1_at	Jasmonate O-methyltransferase	-86.62
Mtr.13375.1.S1_at	Hypothetical protein	-86.53
Msa.3176.1.S1_at	Hypothetical protein	-86.50
Mtr.26597.1.S1_at	Hypothetical protein	-86.11
Mtr.10377.1.S1_at	Hypothetical protein	-85.81
Mtr.16941.1.S1_at	UDP-glucuronosyl/UDP-glucosyltransferase	-85.24
Mtr.40546.1.S1_at	Hypothetical protein	-84.09
Mtr.44844.1.S1_at	Chalcone reductase	-83.46
Mtr.8650.1.S1_at	Xyloglucan endotransglycosylase hydrolase 1	-83.08
Msa.1197.1.S1_at	Hypothetical protein	-82.70
Mtr.41445.1.S1_at	Hypothetical protein	-82.66
Mtr.47151.1.S1_at	Glutathione S-transferase	-81.82
Msa.1381.1.S1_at	Hypothetical protein	-81.76
Mtr.48649.1.S1_at	Cellulose synthase	-81.47
Msa.1752.1.S1_s_at	Hypothetical protein	-79.68
Mtr.37367.1.S1_at	UDP-glucosyltransferase	-79.55
Mtr.35647.1.S1_at	Hypothetical protein	-79.07
Mtr.33852.1.S1_at	Hypothetical protein	-78.78
Mtr.9748.1.S1_at	Peptidyl-prolyl cis-trans isomerase TLP38	-78.35
Mtr.5621.1.S1_at	Hypothetical protein	-77.29
Mtr.13564.1.S1_at	Extracellular calcium sensing receptor	-76.83
Mtr.37384.1.S1_at	Granule-bound starch synthase	-76.14
Mtr.37215.1.S1_at	Photosystem II type I chlorophyll a/b-binding protein	-76.07
Mtr.42902.1.S1_s_at	VuP6CS protein	-76.04
Mtr.44991.1.S1_at	Cucumisin-like serine protease	-74.24
Mtr.40861.1.S1_at	Cytochrome P450	-73.99
Mtr.33459.1.S1_s_at	Hypothetical protein	-73.91
Mtr.41387.1.S1_at	Hypothetical protein	-73.90
Mtr.4948.1.S1_at	Hypothetical protein	-73.81
Mtr.4951.1.S1_at	Hypothetical protein	-73.63
Mtr.37221.1.S1_at	Photosystem II type I chlorophyll a/b-binding protein	-72.51
Mtr.40497.1.S1_at	Glucose acyltransferase	-72.47
Mtr.50426.1.S1_at	Lipoxygenase	-72.43
Mtr.32384.1.S1_s_at	Beta-amyrin synthase	-72.07
Mtr.10377.1.S1_s_at	Hypothetical protein	-72.04
Mtr.2065.1.S1_at	Ent-kaurenoic acid oxidase	-71.06
Mtr.41515.1.S1_at	Phosphate transporter	-70.99
Mtr.37368.1.S1_at	Hypothetical protein	-70.80
Mtr.6745.1.S1_s_at	Violaxanthin de-epoxidase	-70.10
Msa.1358.1.S1_at	Hypothetical protein	-69.56
Msa.2589.1.S1_at	Hypothetical protein	-69.47
Mtr.37362.1.S1_at	CPRD46 protein	-69.36
Mtr.11545.1.S1_at	Hypothetical protein	-69.22

Mtr.38772.1.S1_at	Hypothetical protein	-68.72
Mtr.10948.1.S1_at	Hypothetical protein	-68.28
Mtr.32302.1.S1_at	Hypothetical protein	-68.20
Mtr.21070.1.S1_at	Hypothetical protein	-68.17
Mtr.33633.1.S1_at	Subtilisin-like serine protease	-67.81
Mtr.43442.1.S1_at	Hypothetical protein	-67.47
Msa.1851.1.S1_at	Hypothetical protein	-67.29
Mtr.32878.1.S1_at	Zeaxanthin epoxidase	-66.47
Mtr.37662.1.S1_at	Proline-rich family protein	-66.31
Mtr.42335.1.S1_at	Hypothetical protein	-65.98
Mtr.10830.1.S1_at	Solanesyl diphosphate synthase	-65.88
Mtr.6707.1.S1_s_at	Hypothetical protein	-65.25
Mtr.1857.1.S1_at	Hypothetical protein	-64.99
Mtr.31744.1.S1_at	Anthranilate N-hydroxycinnamoyl/benzoyltransferase	-64.85
Mtr.13758.1.S1_at	Hypothetical protein	-64.43
Msa.3154.1.S1_at	Hypothetical protein	-64.34
Mtr.2064.1.S1_at	Hypothetical protein	-64.17
Mtr.43118.1.S1_s_at	Cytochrome P450 72A1	-63.83
Mtr.12722.1.S1_at	Hypothetical protein	-63.73
Mtr.37252.1.S1_at	Salt-tolerance protein	-63.69
Mtr.44640.1.S1_at	Hypothetical protein	-63.68
Mtr.2129.1.S1_at	Hypothetical protein	-63.58
Mtr.44580.1.S1_at	Hypothetical protein	-63.33
Mtr.46324.1.S1_at	Hypothetical protein	-62.69
Mtr.8207.1.S1_at	Hypothetical protein	-62.62
Mtr.43480.1.S1_at	Hypothetical protein	-62.34
Mtr.48573.1.S1_at	Haloacid dehalogenase-like hydrolase	-61.83
Mtr.36069.1.S1_s_at	Hypothetical protein	-61.68
Mtr.51618.1.S1_at	Hypothetical protein	-61.19
Mtr.12942.1.S1_at	Late embryogenesis abundant protein	-61.11
Mtr.41042.1.S1_s_at	Phosphoethanolamine N-methyltransferase	-61.00
Mtr.49244.1.S1_at	NADPH HC toxin reductase	-60.73
Mtr.22013.1.S1_s_at	Peroxiredoxin Q	-60.45
Mtr.18630.1.S1_at	Prenyltransferase/squalene oxidase	-60.35
Mtr.241.1.S1_at	Delta-6-desaturase	-60.23
Mtr.27898.1.S1_at	Alanine aminotransferase	-60.21
Mtr.46540.1.S1_at	Hypothetical protein	-59.22
Mtr.11088.1.S1_at	37kDa chloroplast inner envelope membrane polypeptide	-58.97
Mtr.6753.1.S1_at	Hypothetical protein	-58.52
Mtr.39290.1.S1_at	Hypothetical protein	-57.96
Mtr.22480.1.S1_s_at	Chloroplast mRNA-binding protein CSP41	-57.81
Mtr.6657.1.S1_s_at	Hypothetical protein	-57.18
Mtr.9187.1.S1_at	Hypothetical protein	-57.17
Mtr.11603.1.S1_at	Non-phototropic hypocotyl-like protein	-57.11
Mtr.10464.1.S1_at	Phosphoribulokinase	-57.05
Msa.1578.1.S1_at	Hypothetical protein	-57.00
Mtr.38920.1.S1_s_at	NFU3 protein	-56.97
Mtr.34115.1.S1_at	Hypothetical protein	-56.81
Msa.941.1.S1_at	Hypothetical protein	-56.78
Mtr.49640.1.S1_at	Hypothetical protein	-56.73
Mtr.17090.1.S1_at	Myb, DNA-binding	-56.53
Mtr.6756.1.S1_at	ABC transporter	-56.34
Mtr.14608.1.S1_at	Heat shock protein Hsp20	-55.83
Mtr.43117.1.S1_at	Cytochrome P450	-55.81

Mtr.6748.1.S1_at	Hypothetical protein	-55.47
Mtr.46628.1.S1_s_at	Peroxidase	-55.42
Mtr.6031.1.S1_at	Lipid transfer protein	-55.35
Mtr.23405.1.S1_at	Hypothetical protein	-55.11
Mtr.42830.1.S1_at	Hypothetical protein	-55.11
Mtr.27626.1.S1_s_at	Hypothetical protein	-55.05
Mtr.5956.1.S1_s_at	Late embryogenesis abundant protein	-54.95
Msa.2805.1.S1_at	Hypothetical protein	-54.61
Msa.565.1.S1_at	Hypothetical protein	-54.42
Mtr.43375.1.S1_at	Hypothetical protein	-54.06
Msa.927.1.S1_at	Hypothetical protein	-53.95
Mtr.20297.1.S1_at	Lipocalin; Calycin; Violaxanthin de-epoxidase	-53.59
Mtr.14755.1.S1_at	Hypothetical protein	-53.58
Mtr.18535.1.S1_at	Hypothetical protein	-53.50
Mtr.37297.1.S1_at	Plastidic aldolase	-53.12
Mtr.16620.1.S1_s_at	Phosphoglycerate/bisphosphoglycerate mutase	-53.11
Mtr.13096.1.S1_at	Hypothetical protein	-52.73
Mtr.41393.1.S1_at	Hypothetical protein	-52.69
Mtr.34955.1.S1_at	Hypothetical protein	-52.68
Mtr.13261.1.S1_s_at	Hypothetical protein	-52.64
Msa.2842.1.S1_at	Hypothetical protein	-52.54
Mtr.8438.1.S1_at	LHCII type I chlorophyll a/b-binding protein	-52.36
Mtr.18757.1.S1_at	Sulfate transporter	-52.13
Mtr.34428.1.S1_s_at	Glyceraldehyde-3-phosphate dehydrogenase	-52.12
Mtr.21535.1.S1_at	Serine/threonine protein kinase	-51.92
Mtr.48257.1.S1_at	Hypothetical protein	-51.73
Msa.2885.1.S1_at	Hypothetical protein	-51.71
Mtr.12797.1.S1_at	Family II lipase EXL3	-51.39
Mtr.41843.1.S1_at	Fimbrin	-51.39
Mtr.8771.1.S1_at	Hypothetical protein	-51.19
Mtr.17317.1.S1_at	S-adenosyl-methionine-sterol-C-methyltransferase	-50.99
Mtr.42981.1.S1_s_at	Hypothetical protein	-50.84
Mtr.24825.1.S1_s_at	Cytochrome P450	-50.43
Mtr.12327.1.S1_s_at	Late embryogenesis abundant protein 1	-49.89
Mtr.41416.1.S1_at	Drought-induced protein RDI	-49.75
Mtr.31606.1.S1_s_at	Phosphoethanolamine N-methyltransferase	-49.62
Mtr.27626.1.S1_at	Hypothetical protein	-49.45
Mtr.253.1.S1_at	Symbiotic ammonium transporter	-49.31
Mtr.50844.1.S1_at	Hypothetical protein	-49.27
Mtr.43208.1.S1_at	Thioredoxin F-type	-48.44
Mtr.24540.1.S1_s_at	Peroxidase	-48.44
Mtr.10816.1.S1_at	Hypothetical protein	-48.16
Mtr.44674.1.S1_at	Phytochrome A supressor spa1	-48.00
Mtr.27305.1.S1_at	S-receptor kinase-like protein 2	-47.91
Mtr.42665.1.S1_at	Hypothetical protein	-47.85
Mtr.39853.1.S1_at	Basic PR-1 protein	-47.85
Mtr.45311.1.S1_at	Hypothetical protein	-47.75
Mtr.49641.1.S1_at	Hypothetical protein	-47.69
Mtr.10844.1.S1_at	Peroxiredoxin Q	-47.69
Mtr.18023.1.S1_at	Ferredoxin	-47.61
Mtr.41360.1.S1_at	Fimbrin-like protein	-47.24
Mtr.43021.1.S1_at	1-deoxy-D-xylulose 5-phosphate reductoisomerase	-47.20
Mtr.40921.1.S1_at	Hypothetical protein	-47.09
Mtr.51216.1.S1_s_at	Homeodomain-like	-47.03

Mtr.34690.1.S1_at	Lactoylglutathione lyase	-46.87
Mtr.50943.1.S1_at	Peptidase	-46.32
Mtr.37847.1.S1_at	UDP-glycosyltransferase 85A8	-46.26
Mtr.11069.1.S1_at	Starch branching enzyme I	-46.23
Mtr.52116.1.S1_at	Glyceraldehyde 3-phosphate dehydrogenase	-46.01
Mtr.33820.1.S1_at	Beta-galactosidase	-45.92
Mtr.12295.1.S1_at	Inorganic pyrophosphatase-like protein	-45.83
Mtr.24918.1.S1_s_at	Receptor-like protein kinase	-45.78
Mtr.13817.1.S1_at	AX110P-like protein	-45.76
Mtr.51216.1.S1_at	Homeodomain-like	-45.74
Mtr.10182.1.S1_at	PREG1-like negative regulator	-45.71
Mtr.41816.1.S1_at	Hypothetical protein	-45.70
Mtr.10124.1.S1_at	Cytochrome P450	-45.67
Mtr.34723.1.S1_at	37 kDa inner envelope membrane protein	-45.54
Mtr.13484.1.S1_s_at	Golden2-like protein 2	-45.49
Mtr.39190.1.S1_at	Cytochrome p450	-45.41
Mtr.10828.1.S1_at	Glucosyltransferase-13	-45.40
Mtr.8552.1.S1_at	Hypothetical protein	-45.21
Mtr.42609.1.S1_at	Hypothetical protein	-45.08
Mtr.43886.1.S1_at	RNA polymerase sigma subunit SigE	-44.95
Mtr.14426.1.S1_at	Aldo/keto reductase	-44.51
Mtr.47158.1.S1_s_at	Hypothetical protein	-44.29
Mtr.8554.1.S1_at	Fructose-bisphosphate aldolase	-44.07
Mtr.11810.1.S1_at	Hypothetical protein	-43.99
Mtr.42226.1.S1_at	Ent-kaurenoic acid oxidase	-43.95
Mtr.18035.1.S1_at	Protein phosphatase 2C	-43.89
Mtr.43939.1.S1_s_at	Ribonuclease HII	-43.76
Mtr.33651.1.S1_at	Hypothetical protein	-43.70
Mtr.8530.1.S1_s_at	Hypothetical protein	-43.66
Mtr.39964.1.S1_s_at	Hypothetical protein	-43.63
Mtr.14013.1.S1_at	Transporter associated with antigen processing-like protein	-43.62
Mtr.5594.1.S1_at	Ca2+/H+ exchanger	-43.53
Mtr.7512.1.S1_at	Hypothetical protein	-43.45
Mtr.49062.1.S1_at	Heat shock protein DnaJ	-43.19
Mtr.34097.1.S1_at	Sucrose-phosphate synthase 2	-43.06
Mtr.29551.1.S1_at	Hypothetical protein	-43.05
Mtr.40306.1.S1_at	Hypothetical protein	-42.74
Mtr.35444.1.S1_at	Oxygen evolving complex protein	-42.17
Mtr.16791.1.S1_s_at	Thylakoid lumen protein	-41.82
Mtr.41715.1.S1_at	Receptor protein	-41.67
Mtr.42241.1.S1_at	Nodulin 26-like protein	-41.48
Mtr.32687.1.S1_at	Hypothetical protein	-41.07
Mtr.20822.1.S1_at	Hypothetical protein	-40.85
Mtr.37529.1.S1_at	Magnesium chelatase subunit	-40.84
Mtr.13704.1.S1_at	Hypothetical protein	-40.66
Mtr.1073.1.S1_at	Hypothetical protein	-40.52
Mtr.6709.1.S1_at	Hypothetical protein	-40.50
Msa.935.1.S1_at	Hypothetical protein	-40.35
Mtr.13324.1.S1_at	Hypothetical protein	-40.35
Mtr.18560.1.S1_at	dTDP-glucose 4-6-dehydratase	-40.31
Mtr.44648.1.S1_at	Hypothetical protein	-40.19
Mtr.9354.1.S1_at	ADP-glucose pyrophosphorylase	-40.19
Mtr.25285.1.S1_at	Symbiotic ammonium transporter	-39.68
Mtr.12202.1.S1_at	Ribulose 1,5-bisphosphate carboxylase small subunit	-39.47

Mtr.6693.1.S1_s_at	NFU3 protein	-39.47
Mtr.12028.1.S1_at	Hypothetical protein	-39.45
Mtr.38403.1.S1_at	Thylakoid lumenal 25.6 kDa protein	-39.26
Mtr.43702.1.S1_s_at	Thylakoid lumenal protein	-39.18
Mtr.34856.1.S1_at	Hypothetical protein	-39.16
Mtr.6744.1.S1_at	Hypothetical protein	-39.16
Mtr.49762.1.S1_at	Elongation factor g	-39.00
Mtr.6048.1.S1_s_at	Hypothetical protein	-39.00
Mtr.26941.1.S1_at	Hypothetical protein	-38.95
Mtr.10925.1.S1_at	Hypothetical protein	-38.40
Mtr.38650.1.S1_at	Anthocyanidin synthase	-38.28
Mtr.17316.1.S1_at	Generic methyltransferase	-38.26
Mtr.6743.1.S1_at	2-oxoglutarate/malate translocator	-38.07
Mtr.43075.1.S1_at	Cyanogenic Beta-Glucosidase	-38.05
Mtr.7344.1.S1_at	Hypothetical protein	-37.84
Mtr.8440.1.S1_at	Salt-tolerance protein	-37.68
Mtr.27621.1.S1_at	Hypothetical protein	-37.67
Mtr.12965.1.S1_at	Hypothetical protein	-37.19
Mtr.37624.1.S1_s_at	3-beta-hydroxysteroiddehydrogenase	-36.95
Mtr.32990.1.S1_at	Non-phototropic hypocotyl-like protein	-36.91
Mtr.35200.1.S1_at	Elongation factor 1 alpha	-36.88
Mtr.13203.1.S1_at	Thylakoid lumenal 29.8 kDa protein	-36.82
Mtr.47958.1.S1_at	Hypothetical protein	-36.63
Mtr.14083.1.S1_at	Hypothetical protein	-36.51
Mtr.16620.1.S1_at	Phosphoglycerate/bisphosphoglycerate mutase	-36.39
Mtr.12949.1.S1_at	MURF2 protein	-36.33
Mtr.5355.1.S1_at	Hypothetical protein	-36.29
Msa.1348.1.S1_at	Hypothetical protein	-36.19
Mtr.13248.1.S1_at	Protein kinase	-36.15
Mtr.11985.1.S1_at	Hypothetical protein	-36.13
Msa.1863.1.S1_at	Hypothetical protein	-36.10
Mtr.10946.1.S1_at	Hypothetical protein	-36.10
Mtr.43018.1.S1_at	Cytochrome P450	-36.09
Mtr.4947.1.S1_at	Hypothetical protein	-35.98
Mtr.37420.1.S1_at	Glycine cleavage system H protein	-35.79
Mtr.30778.1.S1_at	Hypothetical protein	-35.79
Msa.981.1.S1_at	Hypothetical protein	-35.70
Mtr.10820.1.S1_at	Hypothetical protein	-35.66
Mtr.10693.1.S1_at	Cytochrome P450	-35.66
Msa.2924.1.S1_at	Hypothetical protein	-35.64
Mtr.2200.1.S1_at	Hypothetical protein	-35.52
Mtr.19937.1.S1_at	Elongation factor G	-35.33
Mtr.34850.1.S1_s_at	Hypothetical protein	-35.21
Mtr.40632.1.S1_at	Hypothetical protein	-35.19
Mtr.11963.1.S1_s_at	Sulphate transporter	-35.11
Mtr.9273.1.S1_at	Benzoyl coenzyme A: benzyl alcohol benzoyl transferase	-35.10
Mtr.10982.1.S1_at	Serine/threonine protein kinase	-35.09
Mtr.36457.1.S1_at	RNA polymerase sigma subunit SigE	-35.05
Mtr.37609.1.S1_at	Hypothetical protein	-35.04
Mtr.20763.1.S1_s_at	ATP sulfurylase	-35.03
Mtr.40092.1.S1_at	Dehydroascorbate reductase	-34.99
Mtr.35838.1.S1_at	Nitrate transporter	-34.83
Mtr.44488.1.S1_at	Hypothetical protein	-34.55
Mtr.27575.1.S1_at	Nitrate transporter	-34.52

Mtr.37557.1.S1_at	Nine-cis-epoxycarotenoid dioxygenase1	-34.41
Mtr.33696.1.S1_at	4-coumarate-CoA ligase	-34.38
Msa.1752.1.S1_at	Hypothetical protein	-34.24
Mtr.17364.1.S1_at	D-galactoside/L-rhamnose binding SUEL lectin	-34.18
Mtr.37623.1.S1_at	3-beta-hydroxysteroiddehydrogenase	-34.17
Mtr.26473.1.S1_at	Isoamylase	-34.01
Mtr.22279.1.S1_at	Hypothetical protein	-34.00
Mtr.34693.1.S1_at	Hypothetical protein	-33.99
Mtr.40866.1.S1_at	Hydrolase-like protein	-33.82
Mtr.47266.1.S1_at	DNA-binding protein 4	-33.62
Mtr.34490.1.S1_at	Hypothetical protein	-33.61
Mtr.9780.1.S1_at	Subtilisin-like protease	-33.60
Mtr.44199.1.S1_at	Hypothetical protein	-33.56
Msa.2533.1.S1_at	Hypothetical protein	-33.22
Mtr.12421.1.S1_at	Hypothetical protein	-33.11
Mtr.40160.1.S1_s_at	Pyrroline-5-carboxylate reductase	-33.09
Mtr.26044.1.S1_s_at	Circadian clock associated1	-33.08
Mtr.44732.1.S1_at	GDSL-motif lipase/hydrolase-like protein	-33.06
Mtr.20948.1.S1_s_at	Membrane located receptor-like protein	-33.01
Mtr.19806.1.S1_at	Hypothetical protein	-32.79
Msa.1609.1.S1_at	Hypothetical protein	-32.69
Mtr.39886.1.S1_at	Hypothetical protein	-32.64
Mtr.4723.1.S1_at	Hypothetical protein	-32.50
Msa.2939.1.S1_at	Hypothetical protein	-32.45
Mtr.3123.1.S1_at	Hypothetical protein	-32.41
Mtr.28829.1.S1_at	Hypothetical protein	-32.17
Mtr.37839.1.S1_at	Hypothetical protein	-32.15
Mtr.13595.1.S1_at	Lecithine cholesterol acyltransferase-like protein	-32.15
Mtr.12138.1.S1_at	Hypothetical protein	-32.12
Mtr.10630.1.S1_at	Nonspecific lipid-transfer protein 2	-32.02
Mtr.47629.1.S1_at	Aldo/keto reductase	-31.90
Mtr.21256.1.S1_at	Late embryogenesis abundant protein	-31.89
Mtr.13606.1.S1_at	Hypothetical protein	-31.85
Mtr.34779.1.S1_at	GDSL-motif lipase/hydrolase	-31.82
Mtr.31456.1.S1_at	FRO1 and FRO2-like protein	-31.64
Mtr.46512.1.S1_at	Alpha/beta-amyrin synthase	-31.57
Mtr.33577.1.S1_at	Starch branching enzyme I	-31.54
Mtr.41251.1.S1_at	Hypothetical protein	-31.46
Msa.1096.1.S1_at	Hypothetical protein	-31.37
Mtr.8799.1.S1_at	Raffinose synthase	-31.33
Mtr.6714.1.S1_at	Hypothetical protein	-31.27
Mtr.43262.1.S1_at	Hypothetical protein	-31.17
Mtr.24189.1.S1_s_at	Fatty acid desaturase	-31.08
Mtr.41363.1.S1_at	Immunophilin / FKBP-type peptidyl-prolyl cis-trans isomerase	-31.05
Mtr.40683.1.S1_at	Photosystem II protein W	-30.93
Mtr.10911.1.S1_at	Phenylpropanoid:glucosyltransferase 1	-30.92
Mtr.10039.1.S1_at	Hypothetical protein	-30.84
Mtr.21910.1.S1_at	Trypsin protein inhibitor 2	-30.82
Mtr.24508.1.S1_at	Hypothetical protein	-30.74
Mtr.38250.1.S1_at	Hypothetical protein	-30.64
Mtr.51074.1.S1_at	Inorganic pyrophosphatase	-30.60
Mtr.42339.1.S1_at	Hypothetical protein	-30.58
Mtr.25313.1.S1_at	Hypothetical protein	-30.56
Mtr.13434.1.S1_at	Peroxisomal membrane protein	-30.53

Mtr.34481.1.S1_at	Abnormal spindle-like protein	-30.52
Mtr.40691.1.S1_at	Lipoxygenase	-30.40
Mtr.10441.1.S1_at	mRNA-binding protein	-30.30
Msa.1822.1.S1_at	Hypothetical protein	-30.28
Mtr.7091.1.S1_at	Zinc-finger-like protein	-30.22
Mtr.6517.1.S1_at	Flavonoid 3'-hydroxylase	-30.14
Mtr.773.1.S1_s_at	LHY protein	-29.95
Mtr.1274.1.S1_at	Proline-rich protein	-29.94
Mtr.45559.1.S1_at	Receptor-like serine/threonine protein kinase ARK2	-29.88
Mtr.40246.1.S1_at	Hypothetical protein	-29.82
Msa.1854.1.S1_at	Hypothetical protein	-29.69
Mtr.18614.1.S1_at	Hypothetical protein	-29.45
Mtr.43911.1.S1_at	Hypothetical protein	-29.20
Mtr.42854.1.S1_at	Thioredoxin m	-29.11
Mtr.42567.1.S1_at	Phantastica transcription factor	-28.98
Mtr.1392.1.S1_s_at	Hypothetical protein	-28.98
Mtr.4580.1.S1_at	ABC transporter	-28.89
Mtr.11477.1.S1_at	PsbP protein	-28.74
Msa.1239.1.S1_at	Hypothetical protein	-28.65
Mtr.43628.1.S1_at	Isoflavonoid glucosyltransferase	-28.62
Mtr.21257.1.S1_at	Late embryogenesis abundant protein	-28.62
Mtr.42261.1.S1_at	Rubisco activase	-28.59
Mtr.9248.1.S1_at	Hypothetical protein	-28.53
Mtr.25652.1.S1_at	Cell-wall P4 protein	-28.49
Mtr.48150.1.S1_s_at	Hypothetical protein	-28.46
Mtr.37883.1.S1_at	Hypothetical protein	-28.31
Mtr.28616.1.S1_at	ABC transporter	-28.29
Mtr.41693.1.S1_at	CND41, chloroplast nucleoid DNA binding protein	-28.13
Mtr.12300.1.S1_at	Aminotransferase 2	-28.10
Mtr.50943.1.S1_s_at	Peptidase	-28.00
Mtr.41305.1.S1_at	CPRD49 protein	-28.00
Mtr.34717.1.S1_at	Disease resistance response protein	-27.97
Mtr.12445.1.S1_at	Hypothetical protein	-27.92
Mtr.31205.1.S1_at	Hypothetical protein	-27.91
Msa.1384.1.S1_at	Hypothetical protein	-27.88
Mtr.40620.1.S1_at	Hypothetical protein	-27.78
Mtr.8773.1.S1_s_at	Poly(A) polymerase	-27.78
Mtr.8977.1.S1_at	Pathogenesis-related protein 1	-27.66
Mtr.11524.1.S1_at	Hypothetical protein	-27.53
Mtr.40994.1.S1_at	ABC transporter	-27.45
Mtr.32213.1.S1_at	Nitrite transpor	-27.43
Mtr.51080.1.S1_at	Phospholipid/glycerol acyltransferase	-27.40
Mtr.42446.1.S1_at	Nitrate reductase	-27.37
Mtr.32479.1.S1_at	Phosphoribosylaminoimidazolecarboxamide formyltransferase	-27.34
Msa.1550.1.S1_at	Hypothetical protein	-27.27
Mtr.41275.1.S1_at	Kinesin-like protein	-27.24
Mtr.9185.1.S1_at	Prephenate dehydratase	-27.18
Mtr.45729.1.S1_s_at	Hypothetical protein	-27.16
Msa.2685.1.S1_at	Hypothetical protein	-27.11
Mtr.43589.1.S1_at	Protease Do-like 1	-27.10
Mtr.48146.1.S1_s_at	Boron transporter	-27.04
Mtr.34852.1.S1_at	Raucaffricine-O-beta-D-glucosidase	-26.96
Mtr.33459.1.S1_at	Hypothetical protein	-26.86
Mtr.2101.1.S1_at	Hypothetical protein	-26.68

Mtr.12736.1.S1_at	Hypothetical protein	-26.52
Mtr.36871.1.S1_at	Calcium-dependent protein kinase	-26.51
Mtr.47901.1.S1_x_at	Glyceraldehyde-3-phosphate dehydrogenase	-26.51
Mtr.3423.1.S1_at	Vesicular glutamate transporter-3	-26.49
Mtr.44988.1.S1_at	Zeaxanthin epoxidase	-26.49
Mtr.21054.1.S1_at	Mitochodrial transcription termination factor	-26.47
Mtr.45397.1.S1_at	Ankyrin repeat-like protein	-26.42
Mtr.16410.1.S1_at	Epoxide hydrolase	-26.28
Mtr.24085.1.S1_at	Hypothetical protein	-26.25
Mtr.41447.1.S1_at	Hypothetical protein	-26.24
Mtr.25633.1.S1_at	2-Cys peroxiredoxin	-26.23
Mtr.43701.1.S1_at	Thylakoid lumenal protein	-26.23
Mtr.39747.1.S1_at	UDP-glucose:flavonoid 3-O-glucosyltransferase	-26.21
Mtr.12720.1.S1_at	Lipoxygenase	-26.16
Mtr.33008.1.S1_at	Hypothetical protein	-26.02
Mtr.43727.1.S1_s_at	Squalene monooxygenase 1	-26.02
Mtr.7842.1.S1_at	Hypothetical protein	-26.01
Mtr.41722.1.S1_at	Hypothetical protein	-25.96
Mtr.44475.1.S1_at	Ultraviolet-B-repressible protein	-25.83
Mtr.11888.1.S1_at	Aldehyde dehydrogenase	-25.82
Mtr.37609.1.S1_s_at	Hypothetical protein	-25.80
Mtr.14762.1.S1_at	Flavoprotein monooxygenase	-25.68
Mtr.50758.1.S1_at	Rubredoxin-type Fe(Cys)4 protein	-25.61
Msa.928.1.S1_at	Hypothetical protein	-25.56
Mtr.36842.1.S1_s_at	Nodulin 26-like protein	-25.51
Mtr.13567.1.S1_at	Hypothetical protein	-25.50
Msa.1363.1.S1_at	Hypothetical protein	-25.49
Mtr.42735.1.S1_at	Hypothetical protein	-25.47
Mtr.45292.1.S1_at	Hypothetical protein	-25.45
Mtr.14782.1.S1_at	2OG-Fe(II) oxygenase	-25.29
Mtr.12063.1.S1_at	Hypothetical protein	-25.03
Mtr.51399.1.S1_at	Hypothetical protein	-25.00
Mtr.33632.1.S1_s_at	Hypothetical protein	-24.98
Mtr.10972.1.S1_at	Hypothetical protein	-24.88
Msa.1838.1.S1_s_at	Hypothetical protein	-24.75
Mtr.21416.1.S1_at	Ribonuclease T2	-24.66
Mtr.10927.1.S1_at	Regulator of chromosome condensation-like protein	-24.66
Mtr.11236.1.S1_at	UDP-glucosyltransferase	-24.65
Mtr.33659.1.S1_at	Hypothetical protein	-24.61
Msa.1593.1.S1_at	Hypothetical protein	-24.60
Mtr.39712.1.S1_at	Rac GTPase activating protein 3	-24.53
Mtr.48892.1.S1_at	Hypothetical protein	-24.51
Mtr.27854.1.S1_at	NADP-dependent oxidoreductase P1	-24.46
Mtr.38370.1.S1_s_at	Hypothetical protein	-24.43
Mtr.10289.1.S1_at	SANT/MYB domain protein	-24.40
Mtr.12026.1.S1_at	Hypothetical protein	-24.36
Mtr.47901.1.S1_at	Glyceraldehyde-3-phosphate dehydrogenase	-24.36
Mtr.42925.1.S1_s_at	Ribulose bisphosphate carboxylase/oxygenase activase	-24.32
Mtr.13393.1.S1_at	Hypothetical protein	-24.20
Msa.3042.1.S1_s_at	Hypothetical protein	-24.16
Mtr.38722.1.S1_at	Hypothetical protein	-24.11
Mtr.39425.1.S1_s_at	ATP-sulfurylase	-23.94
Mtr.36333.1.S1_at	Flavonoid 3'-hydroxylase	-23.89
Mtr.9624.1.S1_at	ABC transporter	-23.82

Msa.1549.1.S1_at	Hypothetical protein	-23.80
Mtr.42112.1.S1_at	Hypothetical protein	-23.79
Mtr.5633.1.S1_at	Esterase/lipase/thioesterase	-23.77
Mtr.46159.1.S1_at	bZIP transcription factor	-23.71
Mtr.1431.1.S1_at	Hypothetical protein	-23.62
Mtr.43350.1.S1_at	Hypothetical protein	-23.49
Mtr.10952.1.S1_at	Sulfate adenylyltransferase	-23.49
Mtr.43627.1.S1_at	Hypothetical protein	-23.40
Mtr.36690.1.S1_at	GH3-like protein	-23.39
Mtr.44534.1.S1_at	Hypothetical protein	-23.38
Mtr.2073.1.S1_at	Copper/topa quinone amine oxidase	-23.37
Msa.3048.1.S1_at	Hypothetical protein	-23.31
Mtr.10857.1.S1_at	MOLYBDENUM COFACTOR BIOSYNTHESIS PROTEIN C	-23.17
Mtr.10593.1.S1_at	Allene oxide cyclase	-23.13
Mtr.42369.1.S1_at	Hypothetical protein	-23.09
Mtr.49256.1.S1_at	Hypothetical protein	-23.07
Mtr.43295.1.S1_at	Hypothetical protein	-23.02
Mtr.22603.1.S1_at	Glyceraldehyde-3-phosphate dehydrogenase A	-22.93
Mtr.13996.1.S1_at	Hypothetical protein	-22.92
Mtr.12493.1.S1_at	Hypothetical protein	-22.88
Mtr.28243.1.S1_at	Hypothetical protein	-22.85
Mtr.14906.1.S1_at	Major intrinsic protein	-22.82
Mtr.43683.1.S1_at	Protein phosphatase 2C	-22.82
Mtr.38087.1.S1_at	Hyoscyamine 6 beta-hydroxylase	-22.72
Mtr.33723.1.S1_at	Hypothetical protein	-22.66
Mtr.37042.1.S1_at	Hypothetical protein	-22.64
Msa.3133.1.S1_at	Hypothetical protein	-22.60
Mtr.45551.1.S1_at	Hypothetical protein	-22.58
Mtr.11326.1.S1_at	Leaf senescence protein	-22.46
Mtr.12258.1.S1_at	Nonspecific lipid-transfer protein precursor (LTP)	-22.45
Mtr.29089.1.S1_s_at	Peroxidase	-22.45
Mtr.10881.1.S1_at	Hypothetical protein	-22.42
Mtr.48925.1.S1_at	Hypothetical protein	-22.42
Mtr.48595.1.S1_at	Hypothetical protein	-22.38
Mtr.15717.1.S1_at	Chlorophyll A-B binding protein	-22.35
Mtr.12842.1.S1_at	Hypothetical protein	-22.33
Mtr.20116.1.S1_s_at	Hypothetical protein	-22.31
Mtr.5369.1.S1_at	Nitrate transporter	-22.31
Mtr.37808.1.S1_at	Hypothetical protein	-22.27
Msa.1787.1.S1_at	Hypothetical protein	-22.27
Mtr.12056.1.S1_s_at	Cytochrome P450	-22.25
Mtr.33019.1.S1_at	Hypothetical protein	-22.25
Mtr.45032.1.S1_at	Squalene epoxidase	-22.24
Mtr.14112.1.S1_s_at	PAP fibrillin	-22.13
Mtr.37286.1.S1_at	Furostanol glycoside 26-O-beta-glucosidase	-22.08
Mtr.43994.1.S1_at	Hypothetical protein	-22.06
Mtr.34625.1.S1_at	Cytochrome P450 71A6	-21.98
Mtr.24823.1.S1_at	Hypothetical protein	-21.95
Mtr.31754.1.S1_at	Isoflavonoid glucosyltransferase	-21.92
Mtr.12499.1.S1_s_at	Hypothetical protein	-21.90
Mtr.41070.1.S1_at	Hypothetical protein	-21.79
Mtr.39524.1.S1_at	Circadian clock associated1	-21.55
Mtr.33666.1.S1_at	Hypothetical protein	-21.47
Mtr.33061.1.S1_at	Hypothetical protein	-21.40

Msa.1743.1.S1_at	Hypothetical protein	-21.36
Mtr.12414.1.S1_at	Apospory-associated protein C	-21.35
Mtr.25367.1.S1_at	12-oxophytodienoic acid 10, 11-reductase	-21.34
Mtr.27471.1.S1_s_at	Reversibly glycosylated protein	-21.34
Mtr.43296.1.S1_at	Hypothetical protein	-21.32
Mtr.20943.1.S1_at	Serine/threonine protein kinase	-21.31
Mtr.5804.1.S1_at	Hypothetical protein	-21.30
Mtr.32752.1.S1_s_at	Hypothetical protein	-21.29
Mtr.40149.1.S1_at	Thiazole biosynthetic enzyme	-21.28
Mtr.36794.1.S1_at	Hypothetical protein	-21.27
Mtr.22141.1.S1_at	Gycosyltransferase	-21.24
Mtr.34140.1.S1_at	CpABA1 protein	-21.16
Mtr.250.1.S1_at	Hypothetical protein	-21.15
Mtr.9477.1.S1_at	Hypothetical protein	-21.13
Mtr.31791.1.S1_at	Hypothetical protein	-21.07
Mtr.45376.1.S1_at	Nitrite transporter	-21.04
Mtr.13968.1.S1_at	GH3-like protein	-21.01
Mtr.18105.1.S1_at	Phosphoglycerate kinase	-20.98
Mtr.51059.1.S1_x_at	Cytochrome P450	-20.97
Mtr.6752.1.S1_at	10 kDa photosystem II polypeptide	-20.89
Mtr.4339.1.S1_at	Hypothetical protein	-20.87
Mtr.11921.1.S1_at	Thylakoid lumen protein	-20.83
Mtr.2703.1.S1_at	Hypothetical protein	-20.78
Mtr.40788.1.S1_at	Hypothetical protein	-20.77
Mtr.39821.1.S1_at	Hypothetical protein	-20.70
Mtr.49839.1.S1_x_at	Hypothetical protein	-20.66
Mtr.17318.1.S1_at	Magnesium-protoporphyrin IX monomethyl ester aerobic oxidative cyclase	-20.65
Mtr.6059.1.S1_at	bHLH protein	-20.63
Mtr.32922.1.S1_at	Amino acid transporter	-20.62
Mtr.20397.1.S1_at	Amidase, hydantoinase/carbamoylase	-20.59
Mtr.12374.1.S1_at	Protochlorophyllide reductase	-20.51
Mtr.14435.1.S1_at	Calcium-binding EF-hand protein	-20.50
Mtr.37731.1.S1_at	PWWP domain protein	-20.45
Mtr.37245.1.S1_at	Chlorophyll a-b binding protein 215	-20.38
Mtr.40249.1.S1_at	ATP synthase delta chain	-20.37
Mtr.42596.1.S1_at	LLS1-like protein	-20.34
Mtr.45179.1.S1_at	Malate dehydrogenase	-20.33
Mtr.5960.1.S1_s_at	Hypothetical protein	-20.31
Mtr.44505.1.S1_at	Glucosyltransferase-9	-20.30
Mtr.45678.1.S1_at	Hypothetical protein	-20.28
Mtr.32351.1.S1_s_at	Hypothetical protein	-20.28
Mtr.12964.1.S1_at	Hypothetical protein	-20.25
Mtr.13080.1.S1_at	Hypothetical protein	-20.22
Mtr.49764.1.S1_at	Auxin Efflux Carrier	-20.20
Mtr.32865.1.S1_at	Hypothetical protein	-20.17
Mtr.10013.1.S1_at	Hypothetical protein	-20.15
Mtr.13356.1.S1_at	Allene oxide cyclase C4	-20.12
Mtr.21614.1.S1_at	Hypothetical protein	-20.11
Mtr.37228.1.S1_at	UDP-glycose:flavonoid glycosyltransferase	-20.08
Mtr.4029.1.S1_at	CTF2A	-20.03
Mtr.13592.1.S1_at	Photomorphogenesis repressor protein	-20.02
Mtr.19622.1.S1_s_at	Hypothetical protein	-20.02
Mtr.52053.1.S1_at	Ribosomal protein L35	-19.98
Msa.3166.1.S1_at	Hypothetical protein	-19.96

Mtr.2675.1.S1_s_at	Transporter-like protein	-19.95
Mtr.12900.1.S1_at	Iron reductase	-19.93
Mtr.21616.1.S1_at	Hypothetical protein	-19.89
Msa.1297.1.S1_at	Hypothetical protein	-19.82
Mtr.41759.1.S1_at	Rac GTPase activating protein	-19.80
Msa.3003.1.S1_at	Hypothetical protein	-19.79
Mtr.40587.1.S1_at	Glyoxalase family protein	-19.76
Mtr.42178.1.S1_at	Starch synthase I	-19.72
Mtr.22814.1.S1_at	Hypothetical protein	-19.70
Mtr.41106.1.S1_at	Hypothetical protein	-19.65
Mtr.11085.1.S1_at	LHY protein	-19.60
Mtr.2893.1.S1_at	Hypothetical protein	-19.54
Mtr.31792.1.S1_at	Hypothetical protein	-19.54
Mtr.8772.1.S1_at	Poly(A) polymerase	-19.50
Mtr.18406.1.S1_at	Hypothetical protein	-19.42
Mtr.43335.1.S1_at	4-coumarate:coenzyme A ligase	-19.40
Mtr.43709.1.S1_at	Starch synthase isoform SS III	-19.40
Mtr.43899.1.S1_at	Hydrolase	-19.39
Mtr.8133.1.S1_at	Hypothetical protein	-19.38
Mtr.11048.1.S1_at	Hypothetical protein	-19.34
Msa.3147.1.S1_at	Hypothetical protein	-19.29
Mtr.45379.1.S1_at	Hypothetical protein	-19.22
Mtr.6869.1.S1_s_at	Glutamate receptor 3.6	-19.21
Mtr.25331.1.S1_at	Glutamate receptor 3.6	-19.18
Msa.1688.1.S1_at	Hypothetical protein	-19.15
Mtr.27388.1.S1_s_at	Rubisco activase	-19.11
Mtr.43610.1.S1_at	Hypothetical protein	-19.10
Msa.1086.1.S1_at	Hypothetical protein	-19.09
Mtr.27132.1.S1_s_at	Histidine-containing phosphotransfer protein	-19.09
Mtr.38869.1.S1_at	Hypothetical protein	-19.08
Mtr.38366.1.S1_at	Hypothetical protein	-19.03
Mtr.40236.1.S1_s_at	Hypothetical protein	-19.02
Mtr.43181.1.S1_at	Thiamine biosynthesis protein thiC	-19.00
Mtr.12236.1.S1_at	Bundle sheath defective protein 2	-18.89
Mtr.41265.1.S1_at	Glutathione S-transferase GST 10	-18.79
Mtr.9137.1.S1_at	Hypothetical protein	-18.79
Mtr.12060.1.S1_at	Annexin	-18.77
Mtr.52315.1.S1_at	Heat shock protein DnaJ	-18.75
Mtr.43381.1.S1_at	Hypothetical protein	-18.72
Mtr.44975.1.S1_at	ABC transporter	-18.66
Mtr.40981.1.S1_at	Cellulose synthase	-18.66
Mtr.35826.1.S1_at	Hypothetical protein	-18.64
Mtr.29595.1.S1_at	Hypothetical protein	-18.64
Mtr.28742.1.S1_at	Very-long-chain fatty acid condensing enzyme CUT1	-18.64
Mtr.22140.1.S1_at	Glycosyltransferase	-18.63
Msa.2771.1.S1_at	Hypothetical protein	-18.61
Mtr.9416.1.S1_at	Protease sppA	-18.57
Mtr.31754.1.S1_x_at	Isoflavonoid glucosyltransferase	-18.57
Msa.1732.1.S1_at	Hypothetical protein	-18.54
Msa.2424.1.S1_at	Hypothetical protein	-18.51
Mtr.27878.1.S1_at	Isoflavone reductase	-18.50
Mtr.9467.1.S1_at	Glutaredoxin	-18.49
Mtr.39011.1.S1_at	Auxin-binding protein ABP19b	-18.44
Mtr.7468.1.S1_at	10 kDa photosystem II polypeptide	-18.40

Mtr.40162.1.S1_at	Reversibly glycosylated protein	-18.32
Msa.1910.1.S1_at	Hypothetical protein	-18.28
Msa.1254.1.S1_at	Hypothetical protein	-18.28
Mtr.14011.1.S1_at	Hypothetical protein	-18.26
Msa.2800.1.S1_at	Hypothetical protein	-18.25
Mtr.928.1.S1_at	Hypothetical protein	-18.23
Mtr.44339.1.S1_at	Hypothetical protein	-18.21
Mtr.44611.1.S1_at	Hypothetical protein	-18.19
Mtr.47158.1.S1_at	Hypothetical protein	-18.14
Mtr.46292.1.S1_at	Malate dehydrogenase	-18.11
Mtr.34703.1.S1_at	Hypothetical protein	-18.09
Msa.1858.1.S1_x_at	Hypothetical protein	-18.03
Mtr.32336.1.S1_at	Cytochrome P450 71A21	-18.00
Mtr.14592.1.S1_at	Peroxidase	-17.98
Mtr.43197.1.S1_at	Hypothetical protein	-17.96
Mtr.34178.1.S1_s_at	Hypothetical protein	-17.90
Mtr.40761.1.S1_at	50S ribosomal protein L11	-17.90
Mtr.12821.1.S1_s_at	Phosphoglucomutase	-17.89
Mtr.32679.1.S1_at	Hypothetical protein	-17.87
Mtr.40814.1.S1_at	G4 protein (Chlorophyll synthetase)	-17.86
Mtr.41960.1.S1_at	Hypothetical protein	-17.85
Mtr.27827.1.S1_at	Hypothetical protein	-17.85
Mtr.12305.1.S1_at	Chlorophyll a/b-binding protein CP24	-17.85
Mtr.6039.1.S1_at	Hypothetical protein	-17.82
Mtr.31215.1.S1_s_at	Hypothetical protein	-17.81
Mtr.47283.1.S1_at	Hypothetical protein	-17.81
Mtr.1617.1.S1_s_at	Basic blue copper protein	-17.77
Mtr.9402.1.S1_at	Aldehyde dehydrogenase	-17.75
Mtr.43174.1.S1_at	Germin-like protein	-17.73
Mtr.20298.1.S1_at	Hypothetical protein	-17.64
Mtr.20201.1.S1_at	Na+/H+ antiporter	-17.63
Mtr.31606.1.S1_at	Phosphoethanolamine N-methyltransferase	-17.63
Mtr.25945.1.S1_at	Myb-related	-17.62
Mtr.5305.1.S1_at	Cytochrome P450	-17.61
Mtr.5368.1.S1_at	Chlorophyll a/b-binding protein	-17.60
Mtr.3942.1.S1_at	Hypothetical protein	-17.59
Mtr.25013.1.S1_at	2'-hydroxy isoflavone/dihydroflavonol reductase	-17.54
Mtr.20149.1.S1_at	Hypothetical protein	-17.49
Mtr.39235.1.S1_at	Hypothetical protein	-17.44
Mtr.27885.1.S1_s_at	Hypothetical protein	-17.43
Mtr.20226.1.S1_at	UDP-glucuronosyl/UDP-glucosyltransferase	-17.43
Mtr.50516.1.S1_at	Deoxyribodipyrimidine photolyase	-17.43
Mtr.37345.1.S1_at	Subtilisin-like protease	-17.40
Mtr.40597.1.S1_at	Seed maturation protein PM39	-17.38
Mtr.2477.1.S1_at	Hypothetical protein	-17.29
Mtr.38712.1.S1_at	Hypothetical protein	-17.28
Msa.1916.1.S1_at	Hypothetical protein	-17.27
Mtr.40734.1.S1_at	Glucosyltransferase-14	-17.26
Mtr.47857.1.S1_at	1,4-alpha-D-glucan maltohydrolase	-17.24
Mtr.39379.1.S1_at	Amino acid permease 6	-17.21
Mtr.20033.1.S1_at	Hypothetical protein	-17.21
Mtr.6141.1.S1_s_at	Alpha-expansin	-17.20
Mtr.51345.1.S1_at	Hypothetical protein	-17.16
Mtr.28584.1.S1_s_at	Hypothetical protein	-17.13

Mtr.13959.1.S1_at	SHOOT1 protein	-17.13
Msa.1805.1.S1_at	Hypothetical protein	-17.10
Mtr.5468.1.S1_at	Hypothetical protein	-17.08
Mtr.8418.1.S1_s_at	Xylulose kinase	-17.07
Msa.1858.1.S1_at	Hypothetical protein	-17.04
Mtr.46511.1.S1_at	Alpha/beta-amyrin synthase	-17.03
Mtr.38923.1.S1_at	Cytoplasmic intermediate filament protein	-17.02
Mtr.26958.1.S1_at	Hypothetical protein	-17.01
Mtr.37608.1.S1_at	Hypothetical protein	-16.95
Mtr.9257.1.S1_at	Starch synthase I	-16.93
Mtr.41132.1.S1_at	Hypothetical protein	-16.81
Mtr.41919.1.S1_s_at	Hypothetical protein	-16.80
Msa.1760.1.S1_at	Hypothetical protein	-16.80
Mtr.20386.1.S1_s_at	D-tyrosyl-tRNA(Tyr) deacylase	-16.74
Mtr.13143.1.S1_at	Starch synthase isoform SS III	-16.72
Mtr.7544.1.S1_at	Isoflavonoid glucosyltransferase	-16.71
Msa.960.1.S1_at	Hypothetical protein	-16.69
Mtr.9205.1.S1_at	Hypothetical protein	-16.68
Mtr.40926.1.S1_at	Thioredoxin-like protein	-16.67
Mtr.2712.1.S1_at	Cyclin delta-3	-16.65
Mtr.10992.1.S1_at	Arabinogalactan protein	-16.65
Mtr.40558.1.S1_at	Short-chain alcohol dehydrogenase	-16.63
Mtr.27410.1.S1_at	Hypothetical protein	-16.60
Mtr.5490.1.S1_at	Hypothetical protein	-16.59
Mtr.41859.1.S1_at	FRO1 and FRO2-like protein	-16.54
Mtr.39251.1.S1_at	Translation initiation factor IF-2	-16.51
Mtr.44109.1.S1_at	Nitrate transporter	-16.51
Mtr.44533.1.S1_at	Hypothetical protein	-16.47
Mtr.30958.1.S1_at	Hypothetical protein	-16.45
Mtr.40755.1.S1_at	ERD7 protein	-16.40
Msa.2888.1.S1_at	Hypothetical protein	-16.35
Mtr.10840.1.S1_at	Hypothetical protein	-16.35
Mtr.5741.1.S1_s_at	Hypothetical protein	-16.34
Mtr.5648.1.S1_s_at	Enhanced disease susceptibility 5 (Eds5)	-16.30
Mtr.27399.1.S1_at	Small basic membrane integral protein	-16.26
Mtr.36999.1.S1_at	Photomorphogenesis repressor protein	-16.24
Mtr.31199.1.S1_s_at	Cytochrome P450	-16.20
Mtr.43316.1.S1_at	Symbiotic ammonium transporter	-16.20
Mtr.7085.1.S1_at	Fatty acid elongase	-16.17
Mtr.8591.1.S1_at	Hypothetical protein	-16.16
Mtr.51040.1.S1_s_at	Cytochrome b-245	-16.14
Mtr.32904.1.S1_s_at	Phosphoglycerate kinase	-16.11
Mtr.43509.1.S1_at	Hypothetical protein	-16.08
Mtr.32128.1.S1_at	Hypothetical protein	-16.07
Mtr.12899.1.S1_s_at	Hypothetical protein	-16.04
Mtr.11503.1.S1_at	Cold acclimation protein	-16.00
Mtr.10789.1.S1_at	Chlorophyll a oxygenase	-15.97
Mtr.42753.1.S1_at	Hypothetical protein	-15.95
Mtr.3259.1.S1_at	Hypothetical protein	-15.92
Mtr.27648.1.S1_at	Hypothetical protein	-15.88
Mtr.49977.1.S1_at	Transketolase	-15.88
Mtr.21135.1.S1_at	2OG-Fe(II) oxygenase	-15.87
Mtr.32939.1.S1_at	Hypothetical protein	-15.83
Mtr.20397.1.S1_s_at	Amidase, hydantoinase/carbamoylase	-15.81

Mtr.9262.1.S1_at	Hsc70 protein	-15.80
Mtr.7850.1.S1_s_at	Thaumatin-like protein	-15.79
Mtr.40232.1.S1_at	Geranylgeranyl hydrogenase	-15.78
Mtr.6717.1.S1_at	Acyl-activating enzyme 14	-15.75
Mtr.42070.1.S1_at	Geminivirus replication protein-interacting protein	-15.71
Mtr.39126.1.S1_at	Hypothetical protein	-15.71
Msa.1080.1.S1_at	Hypothetical protein	-15.68
Mtr.45013.1.S1_at	Hypothetical protein	-15.67
Msa.2550.1.S1_at	Hypothetical protein	-15.66
Mtr.40667.1.S1_at	Hypothetical protein	-15.59
Mtr.38550.1.S1_at	Subtilisin-like proteinase	-15.55
Mtr.6132.1.S1_s_at	Subtilisin-like protein protease	-15.50
Mtr.39017.1.S1_at	Hypothetical protein	-15.50
Mtr.43522.1.S1_at	Plastid-lipid associated protein PAP/fibrillin	-15.49
Mtr.38371.1.S1_at	Hypothetical protein	-15.47
Mtr.37517.1.S1_at	Basic blue protein	-15.47
Mtr.6877.1.S1_at	Drought responsive element binding protein	-15.47
Mtr.41702.1.S1_at	Hypothetical protein	-15.42
Mtr.51053.1.S1_at	Hypothetical protein	-15.41
Mtr.919.1.S1_s_at	Hypothetical protein	-15.41
Mtr.37463.1.S1_at	CesA2	-15.39
Mtr.8588.1.S1_at	Hypothetical protein	-15.39
Mtr.12779.1.S1_at	Photosystem II stability/assembly factor HCF136	-15.36
Mtr.37342.1.S1_at	Mannitol dehydrogenase	-15.32
Mtr.33645.1.S1_at	Lipoate-protein ligase	-15.32
Mtr.11387.1.S1_at	Hypothetical protein	-15.30
Mtr.28597.1.S1_at	Hypothetical protein	-15.28
Mtr.11416.1.S1_at	HMG-box transcription factor 3	-15.25
Mtr.8219.1.S1_at	Hypothetical protein	-15.22
Mtr.6551.1.S1_at	Hypothetical protein	-15.18
Msa.1943.1.S1_at	Hypothetical protein	-15.16
Mtr.51188.1.S1_at	Glycine hydroxymethyltransferase	-15.08
Mtr.9437.1.S1_at	Hypothetical protein	-15.07
Mtr.36889.1.S1_s_at	Hypothetical protein	-15.03
Mtr.43807.1.S1_at	Hypothetical protein	-14.97
Mtr.6155.1.S1_at	Protein kinase	-14.94
Msa.1307.1.S1_at	Hypothetical protein	-14.94
Mtr.18797.1.S1_at	Proteinase inhibitor I3	-14.86
Mtr.6738.1.S1_at	Receptor-like protein kinase	-14.86
Mtr.15561.1.S1_at	GCN5-related N-acetyltransferase	-14.82
Mtr.10192.1.S1_at	DREB-like protein	-14.81
Mtr.12806.1.S1_at	Ascorbate oxidase promoter-binding protein	-14.79
Mtr.6869.1.S1_at	Glutamate receptor 3.6	-14.79
Mtr.11302.1.S1_at	Glycosyl hydrolase	-14.79
Mtr.44317.1.S1_at	ApaG protein	-14.78
Mtr.4339.1.S1_s_at	Hypothetical protein	-14.71
Mtr.35254.1.S1_at	Short chain dehydrogenase/reductase	-14.69
Msa.1808.1.S1_at	Hypothetical protein	-14.69
Mtr.11082.1.S1_at	Perchloric acid soluble translation inhibitor protein	-14.65
Mtr.12582.1.S1_at	Hypothetical protein	-14.65
Mtr.15585.1.S1_at	Copper/Zinc superoxide dismutase	-14.63
Mtr.33572.1.S1_at	Glucose transporter HXT5	-14.62
Msa.2980.1.S1_at	Hypothetical protein	-14.62
Mtr.28817.1.S1_at	Hypothetical protein	-14.60

Mtr.32682.1.S1_at	Leucyl aminopeptidase	-14.60
Mtr.2077.1.S1_at	Hypothetical protein	-14.59
Mtr.17600.1.S1_at	Protein kinase	-14.55
Mtr.11295.1.S1_at	Transport protein	-14.47
Msa.1357.1.S1_at	Hypothetical protein	-14.46
Mtr.39718.1.S1_at	Hypothetical protein	-14.46
Mtr.8410.1.S1_at	Sulfate transporter	-14.44
Mtr.33861.1.S1_at	Hypothetical protein	-14.43
Mtr.14704.1.S1_at	Carbamoyl-phosphate synthase	-14.41
Mtr.11073.1.S1_at	Short-chain dehydrogenase Tic32	-14.40
Mtr.14524.1.S1_at	Glucose/ribitol dehydrogenase	-14.39
Mtr.48866.1.S1_at	Hypothetical protein	-14.39
Mtr.8811.1.S1_at	Hypothetical protein	-14.38
Mtr.34376.1.S1_at	Hypothetical protein	-14.33
Mtr.7197.1.S1_at	Inositol 1,3,4-trisphosphate 5/6-kinase	-14.32
Mtr.52150.1.S1_at	UTP-glucose-1-phosphate uridylyltransferase	-14.31
Mtr.44805.1.S1_at	Hypothetical protein	-14.29
Mtr.45039.1.S1_at	Peptidylprolyl isomerase	-14.28
Mtr.1747.1.S1_at	Hypothetical protein	-14.23
Mtr.43077.1.S1_at	Hypothetical protein	-14.22
Mtr.42306.1.S1_at	Hypothetical protein	-14.21
Mtr.46164.1.S1_s_at	Hypothetical protein	-14.21
Mtr.44854.1.S1_at	Hypothetical protein	-14.20
Mtr.37749.1.S1_at	Hypothetical protein	-14.20
Mtr.9046.1.S1_at	GAST1 protein	-14.19
Mtr.38889.1.S1_at	Metacaspase 3	-14.18
Mtr.50822.1.S1_x_at	Chlorophyll A-B binding protein	-14.17
Msa.3192.1.S1_at	Hypothetical protein	-14.15
Mtr.39266.1.S1_at	Cation diffusion facilitator 9	-14.14
Mtr.38438.1.S1_at	Hypothetical protein	-14.14
Mtr.38471.1.S1_at	Ring finger protein	-14.10
Msa.1471.1.S1_at	Hypothetical protein	-14.10
Mtr.42897.1.S1_at	Ycf23 protein	-14.10
Mtr.19992.1.S1_at	Phytochrome kinase substrate 1	-14.09
Mtr.33891.1.S1_at	Hypothetical protein	-14.09
Msa.1744.1.S1_at	Hypothetical protein	-14.01
Mtr.9686.1.S1_at	Beta-1,3-glucanase	-14.00
Mtr.26499.1.S1_at	Lateral organ boundaries (LOB) domain family protein	-13.96
Mtr.15459.1.S1_at	Major intrinsic protein	-13.94
Mtr.35044.1.S1_at	Nine-cis-epoxycarotenoid dioxygenase2	-13.93
Mtr.49786.1.S1_x_at	Glycosyl hydrolases family 17	-13.93
Mtr.16202.1.S1_at	Esterase/lipase/thioesterase	-13.92
Mtr.33004.1.S1_at	Hypothetical protein	-13.91
Mtr.33489.1.S1_at	ABC transporter	-13.89
Mtr.39715.1.S1_at	Isopiperitenone reductase	-13.86
Mtr.38724.1.S1_at	Hypothetical protein	-13.85
Mtr.50016.1.S1_at	Hypothetical protein	-13.84
Mtr.18579.1.S1_at	Myb, DNA-binding	-13.84
Mtr.39302.1.S1_at	Translation initiation factor IF-2	-13.76
Mtr.44703.1.S1_at	Hypothetical protein	-13.76
Msa.1547.1.S1_at	Hypothetical protein	-13.76
Mtr.38356.1.S1_s_at	Hypothetical protein	-13.73
Mtr.27619.1.S1_at	Hypothetical protein	-13.72
Mtr.12770.1.S1_at	Phosphoglucomutase	-13.70

Mtr.13545.1.S1_at	Hypothetical protein	-13.66
Mtr.41958.1.S1_at	Hypothetical protein	-13.64
Mtr.1639.1.S1_at	Hypothetical protein	-13.63
Mtr.20515.1.S1_at	Ferrochelatase	-13.61
Mtr.42075.1.S1_at	Cytochrome P450	-13.61
Mtr.13134.1.S1_at	Hypothetical protein	-13.59
Mtr.42019.1.S1_at	ClpB heat shock protein	-13.59
Mtr.32665.1.S1_s_at	Myo-inositol-1-phosphate synthase	-13.57
Msa.1850.1.S1_at	Hypothetical protein	-13.56
Mtr.27875.1.S1_at	Hypothetical protein	-13.56
Mtr.43498.1.S1_at	Hypothetical protein	-13.55
Mtr.4983.1.S1_at	Hypothetical protein	-13.53
Mtr.12256.1.S1_at	Nonspecific lipid-transfer protein precursor (LTP)	-13.50
Msa.3182.1.S1_at	Hypothetical protein	-13.49
Msa.1421.1.S1_at	Hypothetical protein	-13.46
Mtr.41524.1.S1_at	Sulfate transporter	-13.46
Mtr.31300.1.S1_at	Disease resistance response protein	-13.45
Mtr.11662.1.S1_at	Hypothetical protein	-13.43
Mtr.8575.1.S1_at	Glucosyltransferase-13	-13.42
Mtr.12199.1.S1_at	Ascorbate oxidase promoter-binding protein	-13.41
Mtr.8517.1.S1_at	Mannitol dehydrogenase	-13.41
Mtr.38850.1.S1_at	Hypothetical protein	-13.39
Mtr.33674.1.S1_s_at	Glutamyl-tRNA reductase 1	-13.34
Mtr.8972.1.S1_at	Hypothetical protein	-13.25
Mtr.8900.1.S1_at	Hypothetical protein	-13.25
Mtr.29086.1.S1_at	Hypothetical protein	-13.24
Msa.3188.1.S1_at	Hypothetical protein	-13.23
Mtr.41681.1.S1_at	Phosphoglycerate kinase	-13.22
Mtr.15285.1.S1_at	Myo-inositol-1-phosphate synthase	-13.22
Mtr.29826.1.S1_at	Hypothetical protein	-13.21
Mtr.21718.1.S1_at	Leucine-rich repeat protein	-13.21
Mtr.42914.1.S1_x_at	Ribulose 1,5-bisphosphate carboxylase small subunit	-13.20
Mtr.44535.1.S1_at	ClpB heat shock protein	-13.17
Mtr.41840.1.S1_at	Cation diffusion facilitator 10	-13.17
Mtr.46740.1.S1_at	Hypothetical protein	-13.12
Mtr.48959.1.S1_at	Flavoprotein pyridine nucleotide cytochrome reductase	-13.10
Mtr.5325.1.S1_at	Hypothetical protein	-13.09
Mtr.45561.1.S1_at	4-coumarate-CoA ligase-like protein	-13.08
Mtr.27132.1.S1_at	Histidine-containing phosphotransfer protein	-13.06
Mtr.28079.1.S1_at	Hypothetical protein	-13.02
Msa.2629.1.S1_at	Hypothetical protein	-13.01
Mtr.32723.1.S1_at	Formiminotransferase-cyclodeaminase	-12.98
Mtr.33044.1.S1_s_at	Hypothetical protein	-12.98
Mtr.34177.1.S1_at	Hypothetical protein	-12.96
Mtr.5651.1.S1_at	Hypothetical protein	-12.95
Mtr.28166.1.S1_s_at	Dihydrolipoamide Dehydrogenase	-12.95
Mtr.11364.1.S1_at	Cytochrome P450	-12.95
Mtr.45068.1.S1_at	Hypothetical protein	-12.94
Mtr.37335.1.S1_at	Chlorophyll a/b-binding protein	-12.94
Mtr.15109.1.S1_s_at	Hypothetical protein	-12.93
Mtr.2086.1.S1_at	Hypothetical protein	-12.91
Mtr.26786.1.S1_at	Hypothetical protein	-12.91
Msa.1109.1.S1_at	Hypothetical protein	-12.90
Mtr.13330.1.S1_at	Hypothetical protein	-12.87

Mtr.5783.1.S1_at	Flavin containing monooxygenase 3	-12.87
Mtr.15413.1.S1_at	Haloacid dehalogenase/epoxide hydrolase	-12.86
Mtr.38984.1.S1_at	Hypothetical protein	-12.85
Mtr.43037.1.S1_at	Glyoxysomal malate dehydrogenase	-12.84
Msa.1749.1.S1_at	Hypothetical protein	-12.84
Mtr.21762.1.S1_s_at	Hypothetical protein	-12.79
Mtr.22693.1.S1_s_at	Hypothetical protein	-12.78
Msa.1101.1.S1_at	Hypothetical protein	-12.77
Mtr.13053.1.S1_s_at	Urophorphyrin III methylase	-12.75
Mtr.15923.1.S1_at	Peptidylprolyl isomerase, FKBP-type	-12.75
Mtr.4716.1.S1_at	Nitrite transport protein	-12.75
Mtr.17489.1.S1_at	Hypothetical protein	-12.74
Mtr.18086.1.S1_at	RNA-directed DNA polymerase	-12.73
Mtr.35894.1.S1_at	Hypothetical protein	-12.72
Mtr.8670.1.S1_at	Hypothetical protein	-12.71
Mtr.39411.1.S1_at	Sulfate transporter 3.3	-12.67
Mtr.11332.1.S1_at	Hypothetical protein	-12.67
Mtr.15310.1.S1_at	Fatty acid desaturase	-12.65
Mtr.33113.1.S1_at	Hypothetical protein	-12.63
Mtr.18220.1.S1_s_at	Hypothetical protein	-12.61
Mtr.40204.1.S1_at	Aldehyde dehydrogenase 1	-12.61
Mtr.40710.1.S1_at	Malate dehydrogenase	-12.61
Mtr.848.1.S1_s_at	Hypothetical protein	-12.58
Mtr.26042.1.S1_at	Hypothetical protein	-12.58
Mtr.48548.1.S1_at	Deoxyxylulose-5-phosphate synthase	-12.56
Mtr.19517.1.S1_at	Ribulose bisphosphate carboxylase	-12.56
Mtr.19562.1.S1_at	Zn-finger, RING	-12.55
Mtr.43200.1.S1_at	Adenosine 5'-phosphosulfate reductase	-12.55
Mtr.51819.1.S1_at	Hypothetical protein	-12.53
Sme.4919.1.S1_at	Hypothetical protein	-12.53
Mtr.38552.1.S1_s_at	Hypothetical protein	-12.50
Mtr.39104.1.S1_at	Xylulose kinase	-12.46
Mtr.11001.1.S1_at	Hypothetical protein	-12.44
Msa.2871.1.S1_at	Hypothetical protein	-12.43
Mtr.50743.1.S1_at	Hypothetical protein	-12.42
Mtr.34745.1.S1_at	Thylakoid lumenal 17.9 kDa protein	-12.38
Mtr.34827.1.S1_at	Hypothetical protein	-12.37
Mtr.37681.1.S1_s_at	Hypothetical protein	-12.37
Mtr.42595.1.S1_at	Hypothetical protein	-12.37
Mtr.33011.1.S1_at	Copper transport protein	-12.36
Mtr.18705.1.S1_at	Peptidase C1A	-12.32
Mtr.50874.1.S1_at	Calcium-binding EF-hand protein	-12.31
Mtr.8715.1.S1_at	UDP-glucose glucosyltransferase	-12.29
Mtr.12821.1.S1_at	Phosphoglucomutase	-12.28
Mtr.25657.1.S1_at	Cell wall protein	-12.27
Mtr.41129.1.S1_s_at	Hypothetical protein	-12.27
Mtr.1814.1.S1_at	RING-H2 zinc finger protein RHA4a	-12.26
Mtr.33495.1.S1_at	Hypothetical protein	-12.25
Mtr.33532.1.S1_at	Kinesin heavy chain	-12.25
Mtr.43503.1.S1_at	Hypothetical protein	-12.23
Mtr.11556.1.S1_at	Carbohydrate oxidase	-12.23
Mtr.39352.1.S1_at	Hypothetical protein	-12.21
Msa.3102.1.S1_x_at	Hypothetical protein	-12.19
Mtr.33622.1.S1_at	Dehydration-responsive family protein	-12.19

Mtr.33700.1.S1_at	Hypothetical protein	-12.17
Msa.1818.1.S1_at	Hypothetical protein	-12.17
Mtr.9742.1.S1_at	Geminivirus replication protein-interacting protein	-12.16
Mtr.23672.1.S1_at	51 kDa seed maturation protein	-12.16
Mtr.13744.1.S1_at	Transmembrane transport protein	-12.07
Mtr.33476.1.S1_at	Receptor protein kinase	-12.07
Mtr.36454.1.S1_at	Hypothetical protein	-12.05
Msa.2727.1.S1_at	Hypothetical protein	-12.03
Mtr.13059.1.S1_at	Hypothetical protein	-12.02
Mtr.13053.1.S1_at	Urophorphyrin III methylase	-12.02
Mtr.37855.1.S1_at	MAP kinase	-12.01
Mtr.38437.1.S1_at	Hypothetical protein	-12.00
Mtr.51445.1.S1_x_at	Hypothetical protein	-11.98
Mtr.10206.1.S1_at	Pentatricopeptide (PPR) repeat-containing protein	-11.97
Mtr.51423.1.S1_at	Sugar transporter	-11.97
Mtr.6362.1.S1_at	Aminopeptidase 2	-11.96
Mtr.25983.1.S1_s_at	Pectinesterase 2	-11.90
Msa.1191.1.S1_at	Hypothetical protein	-11.90
Mtr.5845.1.S1_at	Hypothetical protein	-11.90
Mtr.39095.1.S1_at	Alcohol oxidase	-11.89
Mtr.41063.1.S1_at	Nine-cis-epoxycarotenoid dioxygenase4	-11.87
Mtr.48945.1.S1_s_at	Ferrochelatase	-11.86
Mtr.38798.1.S1_at	Hypothetical protein	-11.85
Mtr.42326.1.S1_s_at	Hypothetical protein	-11.85
Mtr.9418.1.S1_s_at	Pathogenesis-related protein	-11.85
Mtr.13975.1.S1_at	Ycf23 protein	-11.84
Mtr.8362.1.S1_at	Hypothetical protein	-11.80
Mtr.42918.1.S1_at	Protein disulfide isomerase	-11.80
Msa.2278.1.S1_at	Hypothetical protein	-11.79
Mtr.44654.1.S1_at	Hypothetical protein	-11.79
Mtr.15310.1.S1_s_at	Fatty acid desaturase	-11.78
Mtr.33529.1.S1_at	Hypothetical protein	-11.77
Mtr.48351.1.S1_at	Proteinase inhibitor 20	-11.77
Mtr.21341.1.S1_at	Glutamyl-tRNA reductase	-11.75
Mtr.11548.1.S1_at	Starch synthase isoform SS III	-11.75
Mtr.33522.1.S1_at	Hypothetical protein	-11.74
Mtr.5344.1.S1_at	Cellulose synthase	-11.72
Mtr.12260.1.S1_at	2-Cys peroxiredoxin	-11.70
Mtr.43282.1.S1_at	S-adenosyl-L-methionine: 2,7,4'-trihydroxyisoflavanone 4'-O-methyltransferase	-11.70
Mtr.50015.1.S1_at	Hypothetical protein	-11.67
Mtr.38432.1.S1_at	Temperature stress-induced lipocalin	-11.66
Mtr.43717.1.S1_at	Hypothetical protein	-11.65
Mtr.35794.1.S1_at	Myo-inositol 1-phoshate synthase	-11.60
Mtr.33765.1.S1_s_at	Hypothetical protein	-11.59
Msa.1095.1.S1_at	Hypothetical protein	-11.57
Mtr.19902.1.S1_at	Isochorismate synthases	-11.55
Mtr.39370.1.S1_at	Hypothetical protein	-11.55
Mtr.48272.1.S1_at	Glycine dehydrogenase	-11.55
Mtr.13458.1.S1_at	3(2),5-BISPHOSPHATE NUCLEOTIDASE-like protein	-11.54
Mtr.41202.1.S1_at	Hypothetical protein	-11.54
Mtr.40443.1.S1_at	50S ribosomal protein L13	-11.47
Mtr.5418.1.S1_at	Magnesium transporter protein	-11.46
Mtr.11931.1.S1_at	Long chain acyl-CoA synthetase	-11.42
Mtr.10301.1.S1_s_at	18S ribosomal RNA	-11.39

Mtr.41906.1.S1_at	Hypothetical protein	-11.37
Mtr.37783.1.S1_at	Epoxide hydrolase	-11.35
Mtr.11791.1.S1_at	Hypothetical protein	-11.32
Mtr.33484.1.S1_s_at	Hypothetical protein	-11.31
Mtr.18604.1.S1_at	Hypothetical protein	-11.29
Mtr.45935.1.S1_at	Hypothetical protein	-11.29
Mtr.47934.1.S1_at	Amino acid permease 6 (AAP6)	-11.23
Mtr.40697.1.S1_at	Putativepod-specific dehydrogenase SAC25	-11.23
Mtr.40730.1.S1_at	Thylakoid lumenal 16.5 kDa protein	-11.22
Mtr.46511.1.S1_x_at	Alpha/beta-amyrin synthase	-11.20
Mtr.44020.1.S1_at	Brn1-like protein	-11.19
Mtr.46060.1.S1_at	Hypothetical protein	-11.19
Mtr.38392.1.S1_at	ClpB heat shock protein	-11.18
Msa.2820.1.S1_at	Hypothetical protein	-11.18
Mtr.10088.1.S1_at	Hypothetical protein	-11.18
Mtr.34189.1.S1_at	Hypothetical protein	-11.17
Mtr.1268.1.S1_at	Hypothetical protein	-11.15
Msa.1161.1.S1_at	Hypothetical protein	-11.14
Mtr.1231.1.S1_s_at	Omega-6 fatty acid desaturase	-11.13
Mtr.5039.1.S1_at	Receptor protein	-11.13
Mtr.41940.1.S1_at	Hypothetical protein	-11.12
Msa.2646.1.S1_at	Hypothetical protein	-11.11
Msa.858.1.S1_at	Hypothetical protein	-11.10
Mtr.12983.1.S1_at	Isp4 like protein	-11.09
Mtr.34700.1.S1_at	Tryptophan synthase beta chain 1	-11.08
Mtr.22897.1.S1_s_at	Polygalacturonase	-11.08
Mtr.9582.1.S1_at	Glycogen(starch) synthase	-11.07
Msa.3052.1.S1_at	Hypothetical protein	-11.07
Mtr.29995.1.S1_at	Hypothetical protein	-11.07
Mtr.875.1.S1_s_at	Thioredoxin x	-11.02
Mtr.42253.1.S1_at	Hypothetical protein	-11.00
Mtr.1429.1.S1_s_at	Hypothetical protein	-10.99
Mtr.20473.1.S1_at	Hypothetical protein	-10.98
Mtr.11668.1.S1_at	Hypothetical protein	-10.96
Mtr.5358.1.S1_at	Cellulose synthase	-10.96
Mtr.44986.1.S1_at	Acyltransferase-like protein	-10.94
Mtr.42587.1.S1_at	Heat shock transcription factor	-10.92
Mtr.12259.1.S1_at	Rubisco activase	-10.92
Mtr.24070.1.S1_at	Plastid-lipid associated protein PAP/fibrillin family	-10.92
Mtr.41590.1.S1_at	Hypothetical protein	-10.90
Mtr.19310.1.S1_s_at	N-acetyl-gamma-glutamyl-phosphate reductase	-10.90
Mtr.44282.1.S1_s_at	THA4 protein	-10.89
Mtr.37780.1.S1_at	Alpha-glucan phosphorylase	-10.88
Mtr.40444.1.S1_at	Hypothetical protein	-10.88
Mtr.28722.1.S1_at	Peptide transporter	-10.85
Mtr.41751.1.S1_at	Hypothetical protein	-10.84
Mtr.16188.1.S1_at	Hypothetical protein	-10.84
Msa.3102.1.S1_at	Hypothetical protein	-10.83
Mtr.13843.1.S1_at	Hypothetical protein	-10.82
Mtr.17233.1.S1_at	Hypothetical protein	-10.80
Mtr.44543.1.S1_at	Transmembrane transport protein	-10.80
Mtr.10480.1.S1_at	Glutamine synthetase	-10.79
Mtr.5768.1.S1_at	Tab2 protein	-10.78
Mtr.38433.1.S1_at	Hypothetical protein	-10.75

Mtr.15109.1.S1_at	Hypothetical protein	-10.75
Mtr.11664.1.S1_at	MYB transcription factor	-10.74
Mtr.5646.1.S1_at	Glutathione S-transferase	-10.73
Mtr.41073.1.S1_at	FPF1 protein	-10.72
Mtr.8920.1.S1_at	2-oxoglutarate/malate translocator	-10.72
Mtr.15051.1.S1_s_at	Hypothetical protein	-10.70
Mtr.13956.1.S1_at	2-oxoglutarate/malate translocator	-10.69
Mtr.45404.1.S1_at	Glycine/D-amino acid oxidases	-10.69
Mtr.12884.1.S1_at	Thioredoxin x	-10.69
Mtr.40462.1.S1_at	Hypothetical protein	-10.68
Mtr.10889.1.S1_at	Hypothetical protein	-10.67
Mtr.38676.1.S1_at	Methyltransferase	-10.67
Mtr.23337.1.S1_at	Nonphototrophic hypocotyl 1b	-10.66
Mtr.39379.1.S1_s_at	Amino acid permease 6	-10.63
Mtr.50822.1.S1_at	Chlorophyll A-B binding protein	-10.62
Mtr.41868.1.S1_at	Tryptophan synthase beta chain 1	-10.61
Mtr.17765.1.S1_at	TGF-beta receptor	-10.58
Mtr.50035.1.S1_at	Hypothetical protein	-10.57
Mtr.42933.1.S1_s_at	Nonspecific lipid-transfer protein precursor	-10.56
Mtr.42982.1.S1_at	Carbonic anhydrase	-10.55
Mtr.41668.1.S1_at	Ribulose-bisphosphate carboxylase activase large isoform protein	-10.55
Mtr.38283.1.S1_at	Hypothetical protein	-10.54
Msa.1928.1.S1_at	Hypothetical protein	-10.52
Mtr.48721.1.S1_at	Cytochrome c	-10.50
Mtr.25494.1.S1_at	Galactinol synthase	-10.50
Mtr.41054.1.S1_at	UDP-glucose glucosyltransferase	-10.48
Mtr.26672.1.S1_s_at	Hypothetical protein	-10.47
Mtr.6694.1.S1_at	Na/Ca,K-exchanger-like protein	-10.44
Mtr.34902.1.S1_s_at	Phosphoenolpyruvate carboxylase	-10.44
Mtr.12909.1.S1_at	Hypothetical protein	-10.44
Mtr.43681.1.S1_at	CYP1	-10.43
Mtr.32760.1.S1_at	Hypothetical protein	-10.41
Msa.3105.1.S1_at	Hypothetical protein	-10.41
Mtr.28238.1.S1_at	Subtilisin-like protease	-10.40
Msa.1143.1.S1_s_at	Hypothetical protein	-10.39
Msa.1655.1.S1_at	Hypothetical protein	-10.39
Mtr.24929.1.S1_at	Hypothetical protein	-10.39
Mtr.38458.1.S1_at	FKBP-type peptidyl-prolyl cis-trans isomerase 2	-10.38
Mtr.20818.1.S1_at	Xylulose kinase	-10.38
Mtr.37298.1.S1_at	Cytochrome P450	-10.36
Mtr.35945.1.S1_at	UDP-glycose:flavonoid glycosyltransferase	-10.35
Mtr.42315.1.S1_at	Starch synthase isoform SS III	-10.34
Mtr.37930.1.S1_at	Hypothetical protein	-10.32
Msa.989.1.S1_s_at	Hypothetical protein	-10.31
Mtr.41089.1.S1_at	Hypothetical protein	-10.30
Mtr.10485.1.S1_at	Glucose-6-phosphate isomerase	-10.28
Mtr.44724.1.S1_at	Anthocyanin acyltransferase	-10.28
Mtr.37389.1.S1_at	Triose phosphate/phosphate translocator	-10.28
Mtr.41162.1.S1_at	Hypothetical protein	-10.26
Mtr.21118.1.S1_at	Hypothetical protein	-10.25
Mtr.32529.1.S1_at	Transcription factor	-10.20
Mtr.12543.1.S1_at	Early nodulin-like protein 2	-10.17
Mtr.44653.1.S1_at	Myb-related transcription activator	-10.17
Mtr.19267.1.S1_at	Photosystem I protein PsaD	-10.16

Mtr.2960.1.S1_at	Magnesium transporter protein	-10.14
Msa.1508.1.S1_at	Hypothetical protein	-10.14
Mtr.24883.1.S1_at	Hypothetical protein	-10.13
Mtr.9192.1.S1_at	Putativedisease resistance protein	-10.12
Mtr.34741.1.S1_at	3(2),5-BISPHOSPHATE NUCLEOTIDASE	-10.11
Mtr.7329.1.S1_at	Ribulose-1,5-bisphosphate carboxylase small subunit	-10.09
Mtr.12448.1.S1_at	IAA-Ala hydrolase (IAR3)	-10.09
Msa.1640.1.S1_at	Hypothetical protein	-10.08
Mtr.17357.1.S1_at	Hypothetical protein	-10.07
Mtr.5120.1.S1_at	Hypothetical protein	-10.07
Mtr.11734.1.S1_at	Sulfate transporter 2.1	-10.04
Mtr.14356.1.S1_at	Photosystem I reaction centre subunit IV/PsaE	-10.03
Mtr.8730.1.S1_at	Hypothetical protein	-10.02
Mtr.6746.1.S1_s_at	LEM3 (Ligand-effect modulator 3)	-10.02
Mtr.43527.1.S1_at	Hypothetical protein	-10.00
Mtr.34711.1.S1_at	C-type cytochrome biogenesis protein	-9.99
Mtr.41079.1.S1_at	NADH dehydrogenase subunit 1	-9.98
Mtr.12422.1.S1_at	Ferredoxin-dependent glutamate synthase	-9.97
Mtr.42033.1.S1_at	Hypothetical protein	-9.96
Mtr.45384.1.S1_at	Hypothetical protein	-9.96
Mtr.39387.1.S1_at	DCL protein	-9.96
Mtr.39699.1.S1_at	Hypothetical protein	-9.93
Mtr.39164.1.S1_at	Hypothetical protein	-9.91
Mtr.36996.1.S1_at	Hypothetical protein	-9.91
Mtr.9952.1.S1_at	Hypothetical protein	-9.91
Mtr.32580.1.S1_at	Alpha-mannosidase	-9.90
Mtr.37643.1.S1_at	NADP-dependent oxidoreductase P2	-9.88
Mtr.24731.1.S1_at	Hypothetical protein	-9.88
Mtr.33417.1.S1_at	MRP-like ABC transporter	-9.88
Mtr.3511.1.S1_at	Anthranilate N-hydroxycinnamoyl/benzoyltransferase	-9.88
Mtr.2057.1.S1_s_at	Glutamate/malate translocator	-9.86
Mtr.34799.1.S1_at	Hypothetical protein	-9.85
Mtr.50122.1.S1_at	Hypothetical protein	-9.82
Mtr.11129.1.S1_at	Hypothetical protein	-9.82
Mtr.12203.1.S1_at	Ribulose 1,5-bisphosphate carboxylase small subunit	-9.82
Mtr.12846.1.S1_at	MAP3K-like protein kinase	-9.81
Msa.1377.1.S1_at	Hypothetical protein	-9.78
Mtr.27359.1.S1_at	Isoleucyl-tRNA synthetase	-9.78
Mtr.18566.1.S1_at	Pyruvate kinase	-9.78
Mtr.44971.1.S1_at	Hypothetical protein	-9.75
Mtr.40422.1.S1_at	Dihydrolipoyl dehydrogenase	-9.73
Mtr.33745.1.S1_s_at	Hypothetical protein	-9.73
Mtr.24793.1.S1_at	Blight resistance protein RGA1	-9.73
Mtr.37427.1.S1_at	Aquaporin TIP4.1 (Tonoplast intrinsic protein 4.1)	-9.73
Mtr.9757.1.S1_at	Quinolinate phosphoribosyltransferase	-9.72
Mtr.7389.1.S1_at	Yippee-like protein	-9.72
Mtr.44198.1.S1_at	Hypothetical protein	-9.71
Mtr.30762.1.S1_at	Anthocyanin acyltransferase	-9.70
Mtr.42326.1.S1_at	Hypothetical protein	-9.70
Mtr.5135.1.S1_at	Protein kinase	-9.70
Mtr.27055.1.S1_s_at	Plastid RNA polymerase sigma-subunit	-9.69
Mtr.43061.1.S1_s_at	GTP-binding protein TypA	-9.69
Mtr.49274.1.S1_at	Cupredoxin	-9.68
Mtr.40347.1.S1_at	Omega-3 fatty acid desaturase	-9.67

Mtr.44539.1.S1_at	Ent-kaurenoic acid oxidase	-9.67
Mtr.12188.1.S1_at	Hypothetical protein	-9.67
Mtr.13898.1.S1_at	GPI-anchored protein	-9.65
Mtr.44897.1.S1_at	Hypothetical protein	-9.60
Msa.1495.1.S1_at	Hypothetical protein	-9.59
Mtr.26801.1.S1_a_at	Hypothetical protein	-9.57
Mtr.17305.1.S1_at	Peptidase S41	-9.57
Mtr.39782.1.S1_at	Ppib protein	-9.55
Mtr.2103.1.S1_at	Hypothetical protein	-9.54
Mtr.31624.1.S1_at	Hypothetical protein	-9.54
Mtr.525.1.S1_at	Hypothetical protein	-9.53
Mtr.45133.1.S1_at	Amine oxidase	-9.53
Mtr.7520.1.S1_at	Hypothetical protein	-9.51
Mtr.44150.1.S1_at	Myristoyl-acyl carrier protein thioesterase	-9.50
Mtr.41860.1.S1_at	Leucine-rich repeat receptor-like protein kinase 1	-9.49
Mtr.47369.1.S1_at	ABC transporter	-9.45
Mtr.9168.1.S1_at	Hypothetical protein	-9.45
Mtr.25951.1.S1_at	Hypothetical protein	-9.44
Mtr.30196.1.S1_at	Salicylic acid carboxyl methyltransferase	-9.42
Mtr.50952.1.S1_at	Ribosomal protein L9	-9.40
Mtr.44491.1.S1_at	Hypothetical protein	-9.38
Mtr.21678.1.S1_at	YABBY protein	-9.35
Mtr.37550.1.S1_at	4-alpha-glucanotransferase	-9.35
Mtr.22897.1.S1_at	Polygalacturonase	-9.34
Mtr.34747.1.S1_at	Hypothetical protein	-9.34
Mtr.37624.1.S1_at	3-beta-hydroxysteroiddehydrogenase	-9.33
Mtr.49172.1.S1_at	Hypothetical protein	-9.33
Mtr.44760.1.S1_at	Sulfolipid synthase	-9.32
Mtr.47977.1.S1_at	Nodule-specific protein Nlj70	-9.31
Mtr.40508.1.S1_at	Magnesium-chelatase	-9.31
Mtr.8954.1.S1_at	Hypothetical protein	-9.30
Mtr.39433.1.S1_at	Xyloglucan endotransglucosylase/hydrolase	-9.29
Mtr.40886.1.S1_at	Hypothetical protein	-9.29
Mtr.49015.1.S1_at	Chlorophyll A-B binding protein	-9.29
Msa.2534.1.S1_at	Hypothetical protein	-9.28
Mtr.34708.1.S1_at	Sulfolipid synthase	-9.27
Mtr.36804.1.S1_at	Hypothetical protein	-9.27
Mtr.50754.1.S1_at	Hypothetical protein	-9.26
Mtr.19984.1.S1_at	Hypothetical protein	-9.25
Mtr.50001.1.S1_s_at	Glutamate synthase	-9.24
Mtr.1885.1.S1_at	Symbiotic ammonium transporter	-9.23
Mtr.33426.1.S1_at	MRP-like ABC transporter	-9.22
Mtr.42265.1.S1_at	Transmembrane protein kinase	-9.19
Mtr.43762.1.S1_at	Hypothetical protein	-9.18
Mtr.37525.1.S1_at	Multifunctional aquaporin	-9.17
Mtr.14312.1.S1_at	Hypothetical protein	-9.16
Mtr.51597.1.S1_at	Hypothetical protein	-9.14
Mtr.41408.1.S1_at	Hypothetical protein	-9.14
Mtr.17068.1.S1_at	WD-40 repeat protein	-9.13
Mtr.929.1.S1_s_at	Hypothetical protein	-9.12
Mtr.14453.1.S1_at	Rhodanese-like protein	-9.12
Mtr.51258.1.S1_at	Annexin	-9.08
Mtr.15871.1.S1_at	Hypothetical protein	-9.08
Mtr.768.1.S1_at	Hypothetical protein	-9.08

Mtr.10707.1.S1_at	Indole-3-acetic acid induced protein ARG2	-9.07
Mtr.40799.1.S1_at	Subtilisin-like protease	-9.07
Mtr.27862.1.S1_s_at	Hypothetical protein	-9.06
Mtr.45368.1.S1_at	Acetolactate synthase	-9.05
Mtr.40230.1.S1_at	Photosystem I reaction center subunit V	-9.04
Mtr.28675.1.S1_at	Polygalacturonase	-9.04
Mtr.43060.1.S1_at	GTP-binding protein TypA	-9.04
Mtr.5844.1.S1_at	Ternary complex factor MIP1-like protein	-9.04
Mtr.20646.1.S1_s_at	Glucose/ribitol dehydrogenase	-9.03
Mtr.32317.1.S1_s_at	Phototropin	-9.03
Mtr.39210.1.S1_at	Hypothetical protein	-9.02
Mtr.24535.1.S1_at	Peroxidase	-9.02
Mtr.20636.1.S1_at	NAD-dependent epimerase/dehydratase	-9.01
Mtr.3101.1.S1_at	Hypothetical protein	-9.00
Mtr.12401.1.S1_s_at	Myb-related transcription factor MYB59	-9.00
Mtr.20579.1.S1_at	Hypothetical protein	-9.00
Mtr.1753.1.S1_at	Hypothetical protein	-9.00
Mtr.19422.1.S1_at	ParB-like nuclease	-8.98
Mtr.1762.1.S1_at	Plastid RNA polymerase sigma-subunit	-8.97
Mtr.9853.1.S1_at	Hypothetical protein	-8.93
Mtr.6033.1.S1_at	Peroxidase	-8.93
Mtr.9805.1.S1_at	Hypothetical protein	-8.93
Mtr.25510.1.S1_at	Glycosyl hydrolase	-8.92
Mtr.13189.1.S1_at	Hypothetical protein	-8.92
Mtr.31727.1.S1_at	Hypothetical protein	-8.91
Mtr.11529.1.S1_at	SOUL heme-binding protein	-8.90
Mtr.12358.1.S1_at	Seed maturation protein LEA 4	-8.90
Mtr.13388.1.S1_s_at	Hypothetical protein	-8.89
Mtr.3071.1.S1_at	Starch synthase, isoform V	-8.88
Mtr.28757.1.S1_at	Hypothetical protein	-8.87
Mtr.41157.1.S1_at	RAC-like GTP binding protein RAC13	-8.86
Mtr.44855.1.S1_at	Urophorphyrin III methylase	-8.86
Mtr.16226.1.S1_at	Serine/threonine protein kinase	-8.86
Mtr.11271.1.S1_at	Hypothetical protein	-8.85
Mtr.26124.1.S1_at	Hypothetical protein	-8.83
Mtr.50015.1.S1_s_at	Hypothetical protein	-8.83
Mtr.27107.1.S1_at	MYC1	-8.82
Mtr.13979.1.S1_at	Lipid transfer protein	-8.81
Mtr.41622.1.S1_at	Hypothetical protein	-8.80
Mtr.33844.1.S1_s_at	Allantoinase	-8.79
Mtr.15414.1.S1_at	Hypothetical protein	-8.78
Mtr.40514.1.S1_at	Ultraviolet-B-repressible protein	-8.77
Mtr.12349.1.S1_at	Hypothetical protein	-8.77
Mtr.42164.1.S1_at	Hypothetical protein	-8.76
Mtr.5954.1.S1_at	ABC transporter	-8.76
Mtr.6070.1.S1_at	Histidine kinase	-8.75
Mtr.20727.1.S1_at	Aldehyde dehydrogenase	-8.75
Mtr.42672.1.S1_at	Hypothetical protein	-8.74
Mtr.47758.1.S1_at	bHLH protein	-8.74
Mtr.12848.1.S1_at	Berberine bridge enzyme-like protein	-8.73
Mtr.40872.1.S1_at	Hypothetical protein	-8.73
Mtr.38476.1.S1_at	Cytochrome c	-8.72
Msa.3002.1.S1_at	Hypothetical protein	-8.71
Mtr.43633.1.S1_at	Hypothetical protein	-8.68

Mtr.47369.1.S1_s_at	ABC transporter	-8.68
Mtr.38596.1.S1_at	Amine oxidase	-8.67
Mtr.49461.1.S1_at	Hypothetical protein	-8.66
Mtr.12042.1.S1_at	Farnesylated protein ATFP6-like protein	-8.66
Mtr.39530.1.S1_at	Hypothetical protein	-8.66
Mtr.37322.1.S1_at	Hypothetical protein	-8.65
Mtr.15289.1.S1_at	Lipoxygenase	-8.64
Mtr.18020.1.S1_at	Hypothetical protein	-8.64
Mtr.30739.1.S1_s_at	Hypothetical protein	-8.64
Mtr.37552.1.S1_at	RNA- or ssDNA-binding protein	-8.62
Mtr.15971.1.S1_at	Glycine cleavage T protein	-8.62
Mtr.13951.1.S1_at	Hypothetical protein	-8.61
Msa.1776.1.S1_at	Hypothetical protein	-8.60
Mtr.40706.1.S1_s_at	ADP-glucose pyrophosphorylase large subunit	-8.60
Mtr.18237.1.S1_at	Hypothetical protein	-8.60
Mtr.7832.1.S1_at	Hypothetical protein	-8.60
Mtr.41918.1.S1_at	Hypothetical protein	-8.59
Mtr.16218.1.S1_s_at	AAA ATPase	-8.58
Mtr.42933.1.S1_x_at	Nonspecific lipid-transfer protein precursor	-8.57
Mtr.31487.1.S1_s_at	Agmatine Iminohydrolase	-8.56
Mtr.37274.1.S1_at	Aquaporin 2	-8.55
Mtr.11741.1.S1_at	Hydrolase	-8.54
Mtr.44282.1.S1_at	THA4 protein	-8.54
Msa.2653.1.S1_at	Hypothetical protein	-8.53
Mtr.52214.1.S1_at	SOUL heme-binding protein	-8.53
Mtr.35532.1.S1_at	Bg55 protein	-8.53
Mtr.35625.1.S1_s_at	Hypothetical protein	-8.52
Msa.925.1.S1_at	Hypothetical protein	-8.52
Mtr.33903.1.S1_s_at	GTP-binding protein TypA	-8.51
Mtr.51379.1.S1_at	bHLH protein	-8.51
Mtr.31299.1.S1_at	MYB transcription factor	-8.51
Msa.1741.1.S1_at	Hypothetical protein	-8.50
Mtr.33055.1.S1_s_at	Hypothetical protein	-8.50
Mtr.39163.1.S1_at	Hypothetical protein	-8.47
Mtr.45049.1.S1_at	Hypothetical protein	-8.46
Mtr.51752.1.S1_at	Short-chain dehydrogenase/reductase	-8.45
Mtr.41047.1.S1_at	Hypothetical protein	-8.44
Mtr.41151.1.S1_s_at	Cer2 protein	-8.44
Mtr.52185.1.S1_at	Hypothetical protein	-8.44
Msa.1766.1.S1_at	Hypothetical protein	-8.43
Mtr.34697.1.S1_at	THA4 protein	-8.43
Mtr.41573.1.S1_at	Double C2-like domain containing protein	-8.43
Mtr.29529.1.S1_at	Hypothetical protein	-8.42
Mtr.33889.1.S1_at	CER1 protein	-8.42
Mtr.44782.1.S1_at	Hypothetical protein	-8.41
Mtr.12899.1.S1_at	Hypothetical protein	-8.41
Mtr.18280.1.S1_at	Serine/threonine protein kinase	-8.39
Mtr.27055.1.S1_at	Plastid RNA polymerase sigma-subunit	-8.38
Mtr.34879.1.S1_at	Hypothetical protein	-8.38
Mtr.49684.1.S1_at	UDP-glucose glucosyltransferase	-8.37
Mtr.39289.1.S1_at	Flavonoid 1-2 rhamnosyltransferase	-8.36
Mtr.13575.1.S1_at	Hypothetical protein	-8.36
Mtr.35488.1.S1_at	Hypothetical protein	-8.35
Mtr.37767.1.S1_at	Hypothetical protein	-8.34

Mtr.34167.1.S1_at	Hypothetical protein	-8.33
Mtr.20229.1.S1_at	UDP-glucose:anthocyanin 5-O-glucosyltransferase	-8.33
Mtr.43132.1.S1_at	Chloroplast nucleoid DNA binding protein	-8.32
Mtr.9534.1.S1_at	Hypothetical protein	-8.31
Mtr.38019.1.S1_at	Lil3 protein	-8.29
Mtr.31623.1.S1_at	Hypothetical protein	-8.28
Mtr.9529.1.S1_at	Hypothetical protein	-8.28
Mtr.2990.1.S1_at	Metal resistance protein	-8.27
Mtr.38481.1.S1_at	Hypothetical protein	-8.26
Mtr.34485.1.S1_at	Hypothetical protein	-8.24
Mtr.45476.1.S1_at	Metal-transporting P-type ATPase	-8.24
Mtr.41624.1.S1_at	SEC14 cytosolic factor	-8.23
Mtr.23945.1.S1_at	Receptor-like kinase	-8.22
Mtr.14257.1.S1_at	Magnesium-protoporphyrin IX methyltransferase	-8.21
Mtr.44524.1.S1_at	Hypothetical protein	-8.18
Mtr.42733.1.S1_at	Histone H1	-8.18
Mtr.12555.1.S1_at	Alpha-glucan phosphorylase	-8.18
Mtr.45371.1.S1_at	Small signal peptidase	-8.17
Mtr.37811.1.S1_at	Hypothetical protein	-8.16
Mtr.6746.1.S1_at	LEM3 (Ligand-effect modulator 3)	-8.16
Mtr.7160.1.S1_at	MRP-like ABC transporter	-8.16
Mtr.44745.1.S1_at	Ferredoxin	-8.15
Mtr.24695.1.S1_at	NADH dehydrogenase	-8.15
Mtr.30722.1.S1_at	Hypothetical protein	-8.15
Mtr.32947.1.S1_at	Hypothetical protein	-8.12
Mtr.9616.1.S1_at	Hypothetical protein	-8.12
Mtr.34597.1.S1_s_at	Hypothetical protein	-8.12
Mtr.49583.1.S1_at	Glycoside hydrolase	-8.12
Mtr.12989.1.S1_at	Fructosamine-3-kinase	-8.11
Mtr.27403.1.S1_at	Hypothetical protein	-8.11
Mtr.9264.1.S1_at	Hypothetical protein	-8.10
Mtr.38747.1.S1_s_at	Sugar transporter	-8.09
Mtr.47933.1.S1_at	Amino acid transporter a	-8.08
Mtr.41565.1.S1_s_at	Hypothetical protein	-8.07
Mtr.19479.1.S1_at	Glutaredoxin	-8.07
Mtr.43087.1.S1_at	Pectin methylesterase	-8.06
Mtr.25320.1.S1_at	Hypothetical protein	-8.04
Mtr.40480.1.S1_at	Allantoinase	-8.02
Mtr.14192.1.S1_at	Inositol monophosphatase	-8.02
Mtr.4818.1.S1_s_at	Gluthamine synthetase	-8.01
Mtr.4048.1.S1_at	Hypothetical protein	-8.00
Mtr.24591.1.S1_at	Hypothetical protein	-8.00
Mtr.44672.1.S1_at	Glutathione S-transferase GST 7	-7.99
Mtr.38938.1.S1_at	Hypothetical protein	-7.99
Mtr.13969.1.S1_at	Glucosyltransferase	-7.96
Mtr.15811.1.S1_at	Hypothetical protein	-7.96
Mtr.38983.1.S1_at	Hypothetical protein	-7.96
Mtr.45956.1.S1_at	Lipase/lipooxygenase	-7.96
Mtr.18021.1.S1_at	Hypothetical protein	-7.96
Mtr.21518.1.S1_s_at	Cytochrome P450	-7.96
Mtr.26161.1.S1_at	Peroxisomal membrane protein	-7.95
Mtr.45562.1.S1_at	Nuclear shuttle interacting protein	-7.95
Msa.2667.1.S1_at	Hypothetical protein	-7.95
Mtr.32681.1.S1_at	Hypothetical protein	-7.94

Mtr.52193.1.S1_at	Glycoside hydrolase	-7.94
Mtr.35142.1.S1_at	Hypothetical protein	-7.92
Mtr.27269.1.S1_at	Hypothetical protein	-7.91
Mtr.32683.1.S1_at	TPR-containing protein	-7.91
Mtr.24912.1.S1_at	Hypothetical protein	-7.91
Mtr.10207.1.S1_at	Early E3 20.6 kDa glycoprotein	-7.90
Mtr.44090.1.S1_at	Hypothetical protein	-7.90
Mtr.13014.1.S1_at	Amine oxidase	-7.90
Mtr.33357.1.S1_at	MutT domain protein	-7.89
Mtr.49787.1.S1_s_at	Glycosyl hydrolases family 17	-7.88
Mtr.47226.1.S1_at	CBL-interacting protein kinase 1	-7.88
Msa.1763.1.S1_at	Hypothetical protein	-7.87
Msa.2712.1.S1_at	Hypothetical protein	-7.86
Mtr.31119.1.S1_at	MRP-like ABC transporter	-7.85
Mtr.5357.1.S1_at	ABC transporter	-7.84
Msa.1528.1.S1_at	Hypothetical protein	-7.84
Mtr.23616.1.S1_at	DNA-binding protein 4	-7.84
Mtr.37745.1.S1_at	Hypothetical protein	-7.82
Mtr.38080.1.S1_at	Hypothetical protein	-7.82
Mtr.33613.1.S1_at	Hypothetical protein	-7.82
Mtr.34610.1.S1_at	Hypothetical protein	-7.79
Mtr.28639.1.S1_at	Acetolactate synthase	-7.79
Mtr.22801.1.S1_at	Polynucleotide phosphorylase	-7.78
Mtr.43749.1.S1_s_at	Hypothetical protein	-7.77
Mtr.17798.1.S1_at	GNS1/SUR4 membrane protein	-7.76
Mtr.5962.1.S1_s_at	Nonspecific lipid-transfer protein	-7.75
Mtr.8917.1.S1_at	Dehydrogenase-like protein	-7.75
Mtr.11408.1.S1_at	Hypothetical protein	-7.72
Mtr.41412.1.S1_at	Thylakoid lumenal 18 kDa protein	-7.72
Mtr.31750.1.S1_at	Sulfate transporter 3.4	-7.71
Mtr.11363.1.S1_at	Glycosyltransferase	-7.70
Mtr.8497.1.S1_at	PSI light-harvesting antenna chlorophyll a/b-binding protein	-7.70
Mtr.45313.1.S1_at	Multidrug resistance-associated protein 2	-7.69
Mtr.38693.1.S1_at	Hypothetical protein	-7.69
Mtr.2111.1.S1_at	Hypothetical protein	-7.67
Mtr.48501.1.S1_at	Leucine-rich repeat	-7.66
Mtr.20946.1.S1_at	Serine/threonine protein kinase	-7.65
Mtr.15409.1.S1_at	Protein prenyltransferase	-7.64
Mtr.42509.1.S1_at	Hypothetical protein	-7.63
Mtr.41659.1.S1_at	Chloroplast carotenoid epsilon-ring hydroxylase	-7.63
Mtr.8659.1.S1_s_at	Beta-galactosidase	-7.63
Mtr.6086.1.S1_s_at	Sesquiterpene cyclase	-7.60
Mtr.8754.1.S1_at	Rhodanese-like family protein	-7.59
Mtr.40991.1.S1_at	Limonoid UDP-glucosyltransferase	-7.59
Mtr.43091.1.S1_s_at	Seed maturation protein LEA 4	-7.59
Mtr.8420.1.S1_at	Hypothetical protein	-7.59
Mtr.18790.1.S1_at	Esterase/lipase/thioesterase	-7.59
Mtr.49627.1.S1_at	Hypothetical protein	-7.54
Mtr.28644.1.S1_at	Cytokinin receptor, partial (26%)	-7.53
Mtr.13528.1.S1_at	Anthranilate N-hydroxycinnamoyl/benzoyltransferase	-7.52
Mtr.31331.1.S1_s_at	Hypothetical protein	-7.52
Mtr.47706.1.S1_at	Myb family transcription factor	-7.50
Mtr.27397.1.S1_at	Hypothetical protein	-7.50
Mtr.5638.1.S1_at	Hypothetical protein	-7.50

Mtr.24075.1.S1_at	Mannitol dehydrogenase	-7.50
Mtr.38608.1.S1_at	Hypothetical protein	-7.49
Msa.2926.1.S1_s_at	Hypothetical protein	-7.47
Msa.3149.1.S1_at	Hypothetical protein	-7.47
Mtr.11816.1.S1_at	Hypothetical protein	-7.46
Mtr.39315.1.S1_s_at	Hypothetical protein	-7.45
Mtr.41227.1.S1_at	Germacrene D synthase	-7.45
Mtr.27368.1.S1_at	Hypothetical protein	-7.45
Mtr.12037.1.S1_at	Hypothetical protein	-7.44
Mtr.33686.1.S1_at	Hypothetical protein	-7.44
Msa.1574.1.S1_at	Hypothetical protein	-7.42
Msa.1022.1.S1_at	Hypothetical protein	-7.42
Mtr.20207.1.S1_at	Thioredoxin	-7.42
Mtr.1715.1.S1_at	Aminoalcoholphosphotransferase	-7.42
Mtr.38746.1.S1_at	Heat shock transcription factor	-7.41
Msa.3097.1.S1_at	Hypothetical protein	-7.41
Mtr.11536.1.S1_at	SOS2-like protein kinase PKS3	-7.41
Mtr.8426.1.S1_at	Chlorophyll a/b binding protein	-7.41
Mtr.43744.1.S1_at	Hypothetical protein	-7.40
Mtr.50904.1.S1_at	Hypothetical protein	-7.39
Mtr.1911.1.S1_s_at	Hypothetical protein	-7.39
Mtr.11025.1.S1_at	Hypothetical protein	-7.39
Mtr.22990.1.S1_at	Receptor-like protein kinase 1	-7.39
Mtr.10887.1.S1_at	Hypothetical protein	-7.38
Mtr.10136.1.S1_at	Hypothetical protein	-7.37
Mtr.43079.1.S1_at	Farnesyl-diphosphate farnesyltransferase	-7.36
Mtr.43748.1.S1_s_at	Hypothetical protein	-7.35
Mtr.15484.1.S1_at	Peroxidase	-7.35
Mtr.37982.1.S1_at	Twist related protein 1	-7.34
Mtr.48911.1.S1_at	Short-chain dehydrogenase/reductase	-7.34
Mtr.38647.1.S1_at	WAK-like kinase	-7.34
Mtr.33894.1.S1_at	Tetracycline transporter protein	-7.33
Mtr.38441.1.S1_at	Hypothetical protein	-7.33
Mtr.17358.1.S1_at	Protein synthesis factor	-7.32
Mtr.1036.1.S1_at	Non-phototropic hypocotyl 3	-7.32
Mtr.48723.1.S1_at	Hypothetical protein	-7.32
Mtr.20794.1.S1_at	Plant lipid transfer/seed storage/trypsin-alpha amylase inhibitor	-7.30
Mtr.38066.1.S1_at	Cinnamyl alcohol dehydrogenase	-7.30
Mtr.9169.1.S1_at	Hypothetical protein	-7.29
Mtr.44847.1.S1_s_at	Glycosyltransferase family protein 47	-7.29
Mtr.11562.1.S1_at	Diphosphonucleotide phosphatase 1	-7.27
Mtr.37927.1.S1_at	Hypothetical protein	-7.27
Mtr.5313.1.S1_s_at	Hypothetical protein	-7.27
Mtr.17735.1.S1_at	Hypothetical protein	-7.25
Msa.2847.1.S1_at	Hypothetical protein	-7.23
Mtr.40963.1.S1_at	En/Spm-like transposon protein	-7.23
Mtr.43359.1.S1_at	Phytochelatin synthetase	-7.22
Mtr.22096.1.S1_at	EIX receptor 1	-7.22
Mtr.762.1.S1_at	Hypothetical protein	-7.21
Msa.1122.1.S1_at	Hypothetical protein	-7.21
Mtr.9163.1.S1_at	Hypothetical protein	-7.21
Mtr.23402.1.S1_at	Aldehyde dehydrogenase	-7.20
Mtr.13420.1.S1_at	Selenium-binding protein	-7.19
Mtr.52183.1.S1_at	Concanavalin A-like lectin/glucanase	-7.19

Mtr.3858.1.S1_at	Leucoanthocyanidin dioxygenase	-7.18
Mtr.16579.1.S1_x_at	Cytochrome P450	-7.18
Mtr.13533.1.S1_at	Hypothetical protein	-7.18
Mtr.51909.1.S1_at	Phenylalanine/histidine ammonia-lyase	-7.16
Mtr.40676.1.S1_at	Chloroplast nucleoid DNA binding protein	-7.14
Mtr.38021.1.S1_at	Hypothetical protein	-7.13
Mtr.50756.1.S1_at	Hypothetical protein	-7.13
Mtr.18798.1.S1_at	Proteinase inhibitor I3	-7.12
Msa.1561.1.S1_at	Hypothetical protein	-7.12
Mtr.44836.1.S1_at	Hypothetical protein	-7.12
Mtr.15420.1.S1_at	ABC transporter	-7.11
Mtr.32661.1.S1_at	Hypothetical protein	-7.10
Mtr.40684.1.S1_at	Anthocyanin 5-aromatic acyltransferase/benzoyltransferase	-7.10
Mtr.13801.1.S1_at	Hypothetical protein	-7.10
Mtr.40975.1.S1_at	Ntrite transport protein	-7.09
Mtr.44860.1.S1_at	MADS5 protein	-7.09
Mtr.27111.1.S1_at	GTPase engC protein 1	-7.08
Msa.1517.1.S1_at	Hypothetical protein	-7.08
Mtr.32044.1.S1_at	Hypothetical protein	-7.08
Mtr.11016.1.S1_at	Hypothetical protein	-7.08
Mtr.13397.1.S1_at	Regulator of chromosome condensation-like protein	-7.07
Mtr.34803.1.S1_at	Multidrug resistance-associated protein	-7.07
Mtr.16311.1.S1_at	Hypothetical protein	-7.07
Mtr.30437.1.S1_at	Hypothetical protein	-7.07
Mtr.31764.1.S1_at	Alpha-mannosidase	-7.06
Msa.1177.1.S1_at	Hypothetical protein	-7.05
Mtr.42492.1.S1_at	Carboxylesterase-like protein	-7.05
Mtr.32983.1.S1_at	Protein kinase	-7.04
Mtr.13614.1.S1_at	Pyrimidine 5'-nucleotidase	-7.04
Mtr.10595.1.S1_s_at	Seed maturation protein PM36	-7.02
Mtr.41215.1.S1_at	Germin-like protein	-7.00
Mtr.32475.1.S1_s_at	Hypothetical protein	-7.00
Mtr.19777.1.S1_at	Hypothetical protein	-7.00
Mtr.9808.1.S1_at	Hypothetical protein	-6.99
Mtr.13167.1.S1_at	Acyl CoA synthetase	-6.98
Mtr.5330.1.S1_at	Serine /threonine protein kinase	-6.98
Msa.1845.1.S1_at	Hypothetical protein	-6.98
Mtr.50932.1.S1_at	Myb, DNA-binding	-6.97
Mtr.13851.1.S1_at	Hypothetical protein	-6.95
Mtr.48535.1.S1_at	Hypothetical protein	-6.94
Mtr.34826.1.S1_at	Hypothetical protein	-6.94
Mtr.41481.1.S1_at	Phosphate translocator	-6.94
Mtr.35957.1.S1_at	Germin-like protein	-6.94
Mtr.38165.1.S1_at	Serine/threonine protein kinase	-6.94
Mtr.11495.1.S1_at	Steroid 5alpha-reductase	-6.93
Mtr.45389.1.S1_at	Hypothetical protein	-6.92
Mtr.12039.1.S1_at	Peptide transporter 1	-6.91
Mtr.11929.1.S1_at	Beta-1,4-xylosidase	-6.90
Mtr.34815.1.S1_at	Hypothetical protein	-6.90
Mtr.11148.1.S1_at	Multi resistance protein	-6.88
Mtr.42374.1.S1_at	Hypothetical protein	-6.88
Mtr.39387.1.S1_s_at	DCL protein	-6.88
Msa.1890.1.S1_at	Hypothetical protein	-6.87
Mtr.38446.1.S1_s_at	Ferredoxin-thioredoxin reductase catalytic chain	-6.86

Mtr.8968.1.S1_at	Hypothetical protein	-6.86
Mtr.41414.1.S1_at	Hypothetical protein	-6.86
Mtr.34752.1.S1_s_at	Hypothetical protein	-6.86
Mtr.7101.1.S1_at	C2H2 zinc-finger protein	-6.85
Mtr.15042.1.S1_at	Histidine kinase A	-6.85
Mtr.8683.1.S1_at	Phosphoenolpyruvate carboxylase	-6.85
Msa.1062.1.S1_at	Hypothetical protein	-6.85
Mtr.42902.1.S1_at	VuP5CS protein	-6.85
Mtr.23946.1.S1_at	Receptor kinase LRK10	-6.84
Mtr.20268.1.S1_at	Hypothetical protein	-6.83
Mtr.35540.1.S1_at	Hypothetical protein	-6.83
Mtr.11988.1.S1_at	Tryptophanyl-tRNA synthetase	-6.83
Mtr.38595.1.S1_at	Hypothetical protein	-6.82
Mtr.10331.1.S1_at	Orcinol O-methyltransferase	-6.82
Mtr.11829.1.S1_at	Hypothetical protein	-6.82
Mtr.40897.1.S1_at	Hypothetical protein	-6.81
Msa.900.1.S1_s_at	Hypothetical protein	-6.81
Mtr.26278.1.S1_s_at	Pyrroline-5-carboxylate reductase	-6.81
Mtr.46046.1.S1_at	Lipolytic enzyme	-6.81
Mtr.32540.1.S1_at	Hypothetical protein	-6.81
Mtr.11421.1.S1_at	Ripening-related protein	-6.81
Mtr.40775.1.S1_at	Protein tyrosine phosphatase	-6.80
Mtr.40473.1.S1_at	Lipid transfer protein	-6.80
Mtr.634.1.S1_at	Hypothetical protein	-6.79
Mtr.39482.1.S1_at	Hypothetical protein	-6.79
Mtr.37673.1.S1_at	Hypothetical protein	-6.78
Mtr.42445.1.S1_at	Hypothetical protein	-6.78
Mtr.6035.1.S1_at	UDP-glycosyltransferase 74G1	-6.78
Mtr.37448.1.S1_at	Photosystem II	-6.77
Mtr.28667.1.S1_at	Hypothetical protein	-6.77
Mtr.9876.1.S1_at	CCAAT-binding transcription factor subunit C	-6.77
Mtr.39530.1.S1_s_at	Hypothetical protein	-6.77
Mtr.21518.1.S1_at	Cytochrome P450	-6.76
Msa.1654.1.S1_at	Hypothetical protein	-6.76
Mtr.22041.1.S1_at	Hypothetical protein	-6.76
Mtr.47780.1.S1_at	Expansin	-6.75
Mtr.8777.1.S1_at	Plastoglobule associated protein PG1	-6.75
Mtr.37443.1.S1_at	Alcohol dehydrogenase 1	-6.75
Mtr.5850.1.S1_at	Protein kinase	-6.74
Mtr.7068.1.S1_at	Threonyl-tRNA synthetase	-6.74
Mtr.41485.1.S1_at	Dynamin-like protein	-6.73
Mtr.29286.1.S1_at	22 kDa peroxisomal membrane protein	-6.73
Mtr.9810.1.S1_at	Hypothetical protein	-6.72
Msa.1665.1.S1_at	Hypothetical protein	-6.72
Mtr.3805.1.S1_at	Laccase	-6.70
Msa.1462.1.S1_at	Hypothetical protein	-6.70
Msa.3140.1.S1_at	Hypothetical protein	-6.70
Mtr.37361.1.S1_at	Chlorophyll a-b binding protein CP29.3	-6.69
Mtr.10011.1.S1_at	Hypothetical protein	-6.69
Mtr.27915.1.S1_at	Hypothetical protein	-6.68
Mtr.42608.1.S1_at	Polysaccharide polymerase	-6.68
Mtr.48754.1.S1_at	Dihydrodipicolinate reductase	-6.67
Mtr.31137.1.S1_at	Hypothetical protein	-6.67
Mtr.40327.1.S1_at	ADP-glucose pyrophosphorylase	-6.67

Mtr.27314.1.S1_s_at	Heat shock transcription factor	-6.66
Mtr.39477.1.S1_at	Myosin-like protein	-6.66
Mtr.10936.1.S1_at	Transcriptional regulator, LysR	-6.66
Mtr.9717.1.S1_at	WAK-like kinase	-6.65
Mtr.16086.1.S1_at	Hypothetical protein	-6.65
Mtr.13413.1.S1_at	Transporter like protein	-6.64
Mtr.10825.1.S1_at	Hypothetical protein	-6.64
Mtr.27223.1.S1_s_at	Hypothetical protein	-6.63
Mtr.29276.1.S1_s_at	Hypothetical protein	-6.62
Mtr.6729.1.S1_at	Hypothetical protein	-6.62
Mtr.44017.1.S1_at	Seed maturation protein PM24	-6.61
Mtr.43727.1.S1_at	Squalene monooxygenase 1	-6.60
Mtr.34738.1.S1_s_at	Copper/zinc superoxide dismutase	-6.60
Mtr.13401.1.S1_at	Hypothetical protein	-6.60
Mtr.35600.1.S1_at	Pyrroline-5-carboxylate reductase	-6.60
Mtr.38254.1.S1_at	Ubiquitin-conjugating enzyme	-6.59
Mtr.32709.1.S1_at	Hypothetical protein	-6.59
Mtr.24210.1.S1_at	Hypothetical protein	-6.59
Mtr.40908.1.S1_at	Ribosomal protein L6	-6.59
Mtr.21261.1.S1_s_at	Hypothetical protein	-6.59
Mtr.40715.1.S1_at	Mercuric reductase	-6.59
Mtr.10767.1.S1_at	Agmatine Iminohydrolase	-6.58
Mtr.15775.1.S1_at	Glycoside hydrolase	-6.58
Mtr.37807.1.S1_at	Transporter, LysE family	-6.57
Mtr.16200.1.S1_at	Esterase/lipase/thioesterase	-6.57
Mtr.32795.1.S1_s_at	Hypothetical protein	-6.57
Mtr.43655.1.S1_at	Glucosyltransferase	-6.56
Mtr.5404.1.S1_at	Receptor protein kinase	-6.55
Mtr.43044.1.S1_at	NADP-dependent glyceraldehyde-3-phosphate dehydrogenase	-6.55
Mtr.44147.1.S1_at	Lipid transfer protein	-6.55
Mtr.38410.1.S1_at	bZIP50 protein	-6.55
Msa.1380.1.S1_at	Hypothetical protein	-6.54
Msa.2613.1.S1_at	Hypothetical protein	-6.54
Mtr.32796.1.S1_at	Sodium transport protein	-6.54
Mtr.44852.1.S1_at	Glu-tRNA(Gln) amidotransferase subunit B	-6.53
Mtr.22277.1.S1_s_at	Hypothetical protein	-6.53
Mtr.43148.1.S1_at	Myb-related transcription factor MYB59	-6.53
Mtr.45291.1.S1_at	Hypothetical protein	-6.53
Msa.3143.1.S1_at	Hypothetical protein	-6.52
Mtr.38919.1.S1_at	Hypothetical protein	-6.51
Mtr.42840.1.S1_at	Germin-like protein	-6.51
Mtr.37352.1.S1_at	Trypsin protein inhibitor 3	-6.50
Mtr.40528.1.S1_s_at	Hypothetical protein	-6.50
Mtr.5847.1.S1_at	Zinc finger protein 1	-6.50
Mtr.39369.1.S1_at	33 kd chloroplast ribonucleoprotein	-6.50
Mtr.31169.1.S1_at	Cytochrome P450	-6.48
Mtr.44868.1.S1_at	Hypothetical protein	-6.48
Mtr.38054.1.S1_at	Hypothetical protein	-6.47
Mtr.40987.1.S1_at	Hypothetical protein	-6.47
Msa.3021.1.S1_at	Hypothetical protein	-6.47
Mtr.48631.1.S1_at	Major facilitator superfamily	-6.46
Mtr.32386.1.S1_at	Strubbelig receptor family 8	-6.46
Mtr.33744.1.S1_s_at	Hypothetical protein	-6.45
Mtr.35696.1.S1_at	Hypothetical protein	-6.43

Mtr.12308.1.S1_at	Photosystem I reaction center subunit psaK	-6.43
Mtr.5655.1.S1_s_at	ADP-glucose pyrophosphorylase large subunit	-6.41
Mtr.12886.1.S1_at	Hypothetical protein	-6.41
Mtr.47452.1.S1_at	Cytochrome P450 81E1	-6.41
Mtr.12223.1.S1_at	Hypothetical protein	-6.41
Mtr.46900.1.S1_at	Hypothetical protein	-6.40
Mtr.13901.1.S1_at	Hypothetical protein	-6.39
Mtr.37166.1.S1_at	UDP-glucose:indole-3-acetate beta-D-glucosyltransferase	-6.39
Msa.3011.1.S1_at	Hypothetical protein	-6.39
Mtr.8759.1.S1_at	Quinone reductase	-6.39
Mtr.40774.1.S1_s_at	Protein tyrosine phosphatase	-6.38
Mtr.5351.1.S1_at	Cuticle protein (Faceless pollen-1) (YORE-YORE protein)	-6.38
Msa.2585.1.S1_at	Hypothetical protein	-6.37
Mtr.6771.1.S1_s_at	Hypothetical protein	-6.37
Mtr.6780.1.S1_at	Starch synthase IVb precursor	-6.37
Mtr.43889.1.S1_at	Beta-1,3-glucanase-like protein	-6.36
Mtr.14419.1.S1_s_at	Purple acid phosphatase	-6.36
Mtr.5586.1.S1_s_at	Beta-carotene hydroxylase	-6.36
Mtr.25043.1.S1_at	Hypothetical protein	-6.35
Mtr.27146.1.S1_s_at	Cyclin-like protein	-6.35
Mtr.11359.1.S1_at	Hypothetical protein	-6.34
Mtr.39748.1.S1_at	Hypothetical protein	-6.33
Mtr.40506.1.S1_at	Serine carboxypeptidase II	-6.33
Mtr.42279.1.S1_at	Protease Do-like 8	-6.33
Mtr.10393.1.S1_at	Thiamin biosynthetic enzyme	-6.33
Mtr.11795.1.S1_at	Proline-rich protein	-6.32
Mtr.13021.1.S1_at	Hypothetical protein	-6.32
Mtr.37929.1.S1_at	Inositol phosphatase	-6.31
Mtr.13958.1.S1_at	Beta-amylase	-6.31
Mtr.24418.1.S1_at	Cytochrome P450	-6.30
Mtr.34896.1.S1_at	Receptor-like protein kinase	-6.28
Mtr.43622.1.S1_s_at	NADH dehydrogenase	-6.27
Msa.1751.1.S1_at	Hypothetical protein	-6.27
Mtr.6500.1.S1_at	Alcohol NADP+ oxidoreductase	-6.26
Mtr.16200.1.S1_x_at	Esterase/lipase/thioesterase	-6.26
Mtr.13436.1.S1_at	Nodule-enhanced protein phosphatase type 2C	-6.25
Mtr.929.1.S1_at	Hypothetical protein	-6.25
Mtr.4862.1.S1_at	Oxidoreductase	-6.24
Mtr.13612.1.S1_at	Hypothetical protein	-6.24
Mtr.33045.1.S1_at	Hypothetical protein	-6.23
Mtr.39390.1.S1_at	Phosphoenolpyruvate carboxylase	-6.23
Mtr.19940.1.S1_at	Hypothetical protein	-6.23
Mtr.37443.1.S1_x_at	Alcohol dehydrogenase 1	-6.22
Mtr.39989.1.S1_at	Hypothetical protein	-6.22
Msa.1642.1.S1_at	Hypothetical protein	-6.20
Mtr.20560.1.S1_at	Purple acid phosphatase	-6.19
Mtr.40152.1.S1_at	Oxygen-evolving enhancer protein 2	-6.19
Mtr.12418.1.S1_at	Hypothetical protein	-6.18
Mtr.10423.1.S1_at	Hypothetical protein	-6.18
Mtr.38672.1.S1_at	Hypothetical protein	-6.18
Mtr.38436.1.S1_at	Cyclin-like protein, partial (18%)	-6.17
Mtr.43856.1.S1_at	Protein kinase	-6.17
Mtr.51598.1.S1_at	NUDIX hydrolase	-6.16
Mtr.33014.1.S1_s_at	Hypothetical protein	-6.16

Mtr.50974.1.S1_at	Hypothetical protein	-6.16
Mtr.45016.1.S1_at	Hypothetical protein	-6.16
Mtr.36140.1.S1_at	Phosphoenolpyruvate carboxylase	-6.15
Mtr.5346.1.S1_at	Receptor-like protein kinase 2	-6.15
Mtr.44131.1.S1_at	Hypothetical protein	-6.15
Mtr.8818.1.S1_at	Hypothetical protein	-6.15
Mtr.13232.1.S1_at	Hypothetical protein	-6.15
Mtr.25689.1.S1_at	UDP-glucose 6-dehydrogenase	-6.14
Mtr.42240.1.S1_at	Hypothetical protein	-6.14
Mtr.22606.1.S1_at	PPR repeat-containing protein	-6.13
Mtr.11999.1.S1_at	Hypothetical protein	-6.13
Mtr.40190.1.S1_at	Flowering promoting factor-like 1	-6.13
Msa.1977.1.S1_at	Hypothetical protein	-6.13
Msa.1651.1.S1_at	Hypothetical protein	-6.13
Mtr.6878.1.S1_s_at	Hypothetical protein	-6.12
Mtr.12306.1.S1_at	Hypothetical protein	-6.11
Mtr.38379.1.S1_at	Egr1 protein	-6.10
Mtr.50307.1.S1_at	Haloacid dehalogenase-like hydrolase	-6.10
Mtr.18997.1.S1_at	IMGZn-finger, Dof type	-6.10
Mtr.2640.1.S1_at	Pullulanase-like protein	-6.09
Mtr.36351.1.S1_at	Cytochrome oxidase II	-6.08
Mtr.16273.1.S1_at	Proteinase inhibitor I12	-6.07
Mtr.34447.1.S1_at	Hypothetical protein	-6.07
Mtr.45567.1.S1_at	Fatty aldehyde dehydrogenase 1	-6.07
Mtr.43533.1.S1_at	Maturation polypeptide	-6.07
Mtr.6980.1.S1_at	Multidrug resistance-associated protein 2	-6.06
Mtr.49627.1.S1_x_at	Hypothetical protein	-6.06
Mtr.45060.1.S1_at	Salt-tolerance protein	-6.06
Mtr.16356.1.S1_s_at	Phospholipid/glycerol acyltransferase	-6.06
Mtr.33664.1.S1_at	Ferredoxin-dependent glutamate synthase	-6.06
Mtr.18659.1.S1_s_at	Hly-III related proteins	-6.06
Msa.1114.1.S1_at	Hypothetical protein	-6.05
Mtr.17661.1.S1_at	Polyprenyl synthetase	-6.04
Mtr.32411.1.S1_a_at	Hypothetical protein	-6.04
Mtr.6081.1.S1_at	Family II lipase EXL2	-6.04
Mtr.9105.1.S1_at	Coproporphyrinogen oxidase	-6.02
Mtr.36338.1.S1_at	Hypothetical protein	-6.01
Mtr.5714.1.S1_at	Hypothetical protein	-6.01
Mtr.43215.1.S1_at	Hypothetical protein	-6.00
Mtr.20947.1.S1_x_at	Serine/threonine protein kinase	-5.99
Mtr.18022.1.S1_at	Hypothetical protein	-5.97
Mtr.28068.1.S1_at	Hypothetical protein	-5.96
Mtr.41407.1.S1_at	Hypothetical protein	-5.96
Mtr.24061.1.S1_at	Hypothetical protein	-5.96
Mtr.32634.1.S1_s_at	Heat shock protein	-5.95
Mtr.5115.1.S1_s_at	Hypothetical protein	-5.95
Msa.1661.1.S1_at	Hypothetical protein	-5.95
Msa.2137.1.S1_at	Hypothetical protein	-5.95
Msa.3118.1.S1_at	Hypothetical protein	-5.94
Msa.1921.1.S1_at	Hypothetical protein	-5.93
Mtr.50199.1.S1_at	Hypothetical protein	-5.93
Mtr.11440.1.S1_at	Thylakoid lumenal 21.5 kDa protein	-5.93
Msa.1853.1.S1_at	Hypothetical protein	-5.92
Mtr.27754.1.S1_at	Hypothetical protein	-5.92

Mtr.48197.1.S1_at	Hypothetical protein	-5.92
Mtr.13421.1.S1_at	Cytochrome b-561	-5.91
Mtr.46510.1.S1_x_at	Alpha/beta-amyrin synthase	-5.91
Mtr.20299.1.S1_at	U box protein	-5.91
Msa.3191.1.S1_s_at	Hypothetical protein	-5.91
Mtr.45511.1.S1_at	Histidine kinase	-5.90
Mtr.2611.1.S1_at	Protein kinase	-5.90
Mtr.46957.1.S1_at	Hypothetical protein	-5.89
Mtr.37164.1.S1_at	Heat shock factor RHSF6	-5.88
Mtr.8567.1.S1_at	Alcohol dehydrogenase 1	-5.87
Mtr.9712.1.S1_at	Copper/zinc superoxide dismutase	-5.87
Mtr.7552.1.S1_at	Hypothetical protein	-5.87
Mtr.44223.1.S1_at	Mitochondrial aspartate-glutamate carrier protein	-5.87
Mtr.19934.1.S1_at	Cysteine proteinase	-5.87
Mtr.36469.1.S1_at	Hypothetical protein	-5.87
Mtr.2661.1.S1_at	Hypothetical protein	-5.87
Mtr.39373.1.S1_at	Alanyl-tRNA synthetase	-5.86
Mtr.43565.1.S1_at	PAR-1b protein	-5.86
Mtr.33854.1.S1_at	Hypothetical protein	-5.85
Mtr.41274.1.S1_at	Hypothetical protein	-5.84
Mtr.13472.1.S1_at	Interleukin-1 receptor-associated kinase-3 (IRAK-3)	-5.84
Mtr.12907.1.S1_at	Hypothetical protein	-5.83
Mtr.13972.1.S1_at	Thioredoxin m	-5.83
Mtr.37955.1.S1_at	Hypothetical protein	-5.82
Mtr.43749.1.S1_at	Hypothetical protein	-5.82
Mtr.34131.1.S1_at	NADP dependent malic enzyme	-5.82
Mtr.14527.1.S1_at	Hypothetical protein	-5.82
Mtr.15340.1.S1_at	Malate dehydrogenase	-5.81
Mtr.28309.1.S1_at	Acyl-CoA thioesterase	-5.80
Mtr.8967.1.S1_s_at	Hypothetical protein	-5.80
Mtr.34640.1.S1_at	Hypothetical protein	-5.80
Mtr.10293.1.S1_at	Alanyl-tRNA synthetase	-5.79
Mtr.32821.1.S1_at	Hypothetical protein	-5.79
Mtr.37341.1.S1_at	Mannitol dehydrogenase	-5.78
Mtr.31127.1.S1_s_at	ERD4 protein	-5.78
Mtr.49783.1.S1_at	Glycosyl hydrolases family 17	-5.77
Mtr.45269.1.S1_at	NADP dependent malic enzyme	-5.77
Mtr.47648.1.S1_at	Hypothetical protein	-5.76
Mtr.13200.1.S1_at	Hypothetical protein	-5.76
Mtr.6825.1.S1_at	Hypothetical protein	-5.76
Mtr.11542.1.S1_s_at	Hypothetical protein	-5.75
Msa.1113.1.S1_at	Hypothetical protein	-5.75
Mtr.39327.1.S1_at	Hypothetical protein	-5.74
Msa.1809.1.S1_at	Hypothetical protein	-5.74
Msa.1661.1.S1_s_at	Hypothetical protein	-5.74
Mtr.39005.1.S1_at	Nitrate transporter	-5.74
Mtr.34797.1.S1_at	Hypothetical protein	-5.74
Mtr.38390.1.S1_at	Hypothetical protein	-5.73
Mtr.31078.1.S1_at	CPD photolyase	-5.73
Mtr.44890.1.S1_at	Calcium binding protein	-5.73
Mtr.16355.1.S1_at	HSP20-like chaperone	-5.73
Mtr.14230.1.S1_at	Hypothetical protein	-5.72
Mtr.11744.1.S1_at	Hypothetical protein	-5.72
Mtr.46061.1.S1_s_at	Dynamin GTPase effector	-5.72

Mtr.13813.1.S1_s_at	Alkaline/neutral invertase	-5.72
Msa.1434.1.S1_at	Hypothetical protein	-5.71
Mtr.27892.1.S1_at	Hypothetical protein	-5.71
Mtr.42133.1.S1_at	Protein kinase	-5.71
Mtr.51464.1.S1_at	Thiolase	-5.70
Msa.3115.1.S1_at	Hypothetical protein	-5.70
Mtr.42337.1.S1_at	4-coumarate:CoA ligase isoenzyme 2	-5.70
Mtr.14519.1.S1_a_at	RNA-binding region RNP-1	-5.70
Mtr.12774.1.S1_s_at	Bg55 protein	-5.70
Msa.3163.1.S1_at	Hypothetical protein	-5.70
Mtr.9072.1.S1_at	Acyltransferase	-5.70
Mtr.25389.1.S1_at	Vestitone reductase	-5.69
Msa.1069.1.S1_at	Hypothetical protein	-5.68
Mtr.19730.1.S1_at	Microtubule-associated protein	-5.68
Msa.1619.1.S1_at	Hypothetical protein	-5.68
Msa.1746.1.S1_at	Hypothetical protein	-5.68
Mtr.31115.1.S1_at	Hypothetical protein	-5.67
Mtr.39936.1.S1_at	Spermidine synthase 2	-5.67
Msa.1112.1.S1_at	Hypothetical protein	-5.67
Mtr.24717.1.S1_s_at	Arabinogalactan protein	-5.66
Mtr.24083.1.S1_at	Uroporphyrinogen decarboxylase	-5.65
Mtr.28737.1.S1_at	Homeodomain protein GhHOX1	-5.64
Mtr.37792.1.S1_at	Hypothetical protein	-5.64
Msa.2814.1.S1_at	Hypothetical protein	-5.64
Mtr.22781.1.S1_at	Photosystem I-N subunit	-5.64
Mtr.49075.1.S1_at	Peptidase M24	-5.64
Mtr.37372.1.S1_at	Dehydroascorbate reductase	-5.63
Mtr.33475.1.S1_s_at	Proline-rich protein	-5.63
Msa.3138.1.S1_a_at	Hypothetical protein	-5.63
Mtr.15245.1.S1_at	ABC transporter	-5.62
Mtr.13613.1.S1_s_at	Actin-related protein 8B	-5.62
Mtr.32848.1.S1_at	Hypothetical protein	-5.61
Mtr.5310.1.S1_s_at	Light harvesting protein	-5.60
Mtr.14519.1.S1_at	RNA-binding region RNP-1	-5.59
Mtr.51925.1.S1_at	Hypothetical protein	-5.59
Mtr.44320.1.S1_at	Hypothetical protein	-5.59
Mtr.20801.1.S1_at	Homeodomain-like protein	-5.59
Mtr.21006.1.S1_at	Hypothetical protein	-5.58
Mtr.38321.1.S1_at	Transfactor-like protein	-5.58
Mtr.8728.1.S1_at	Hypothetical protein	-5.58
Mtr.17441.1.S1_at	Glycoside hydrolase	-5.58
Mtr.30695.1.S1_at	Hypothetical protein	-5.57
Mtr.1779.1.S1_at	Hypothetical protein	-5.57
Msa.1021.1.S1_at	Hypothetical protein	-5.57
Mtr.10648.1.S1_at	UOS1	-5.57
Mtr.11208.1.S1_at	Hypothetical protein	-5.57
Mtr.44814.1.S1_at	Ycf49-like protein	-5.56
Mtr.8844.1.S1_at	Mitogen-activated protein kinase	-5.56
Mtr.48097.1.S1_at	Hypothetical protein	-5.55
Mtr.51204.1.S1_at	Amine oxidase	-5.55
Mtr.48937.1.S1_at	Hypothetical protein	-5.55
Mtr.10412.1.S1_at	Oxygen-evolving enhancer protein 3	-5.55
Mtr.34732.1.S1_at	Trehalose-6-phosphate synthase	-5.55
Mtr.13374.1.S1_at	Protein kinase	-5.54

Mtr.34614.1.S1_at	Hypothetical protein	-5.54
Mtr.5618.1.S1_at	Zinc finger protein	-5.54
Msa.1072.1.S1_at	Hypothetical protein	-5.52
Mtr.2306.1.S1_at	Trehalose-6-phosphate phosphatase	-5.52
Mtr.32674.1.S1_at	Hypothetical protein	-5.51
Mtr.5426.1.S1_at	Receptor protein kinase	-5.50
Mtr.41877.1.S1_at	Hypothetical protein	-5.50
Mtr.51462.1.S1_s_at	Zn-finger, RING	-5.50
Mtr.41896.1.S1_s_at	Hypothetical protein	-5.50
Mtr.12262.1.S1_at	Extensin 3	-5.49
Msa.3103.1.S1_at	Hypothetical protein	-5.49
Mtr.47272.1.S1_at	Elongation factor P (EF-P)	-5.49
Mtr.39696.1.S1_at	Hypothetical protein	-5.49
Mtr.43833.1.S1_at	Hypothetical protein	-5.48
Mtr.39943.1.S1_at	Hypothetical protein	-5.48
Mtr.42768.1.S1_at	Hypothetical protein	-5.48
Mtr.6731.1.S1_at	PSII D1 protein processing enzyme	-5.48
Mtr.40187.1.S1_at	Serine hydroxymethyltransferase	-5.47
Mtr.19691.1.S1_at	Hypothetical protein	-5.46
Mtr.40561.1.S1_at	Hypothetical protein	-5.45
Mtr.11939.1.S1_at	RelA-SpoT like protein RSH1	-5.45
Mtr.41489.1.S1_at	Hypothetical protein	-5.45
Mtr.4994.1.S1_at	Hypothetical protein	-5.45
Mtr.37015.1.S1_at	Hydroxymethylglutaryl-CoA lyase	-5.45
Mtr.50308.1.S1_at	Hypothetical protein	-5.45
Mtr.30232.1.S1_at	Hypothetical protein	-5.44
Mtr.24601.1.S1_at	Receptor-related protein kinase	-5.44
Mtr.32162.1.S1_at	Hypothetical protein	-5.44
Mtr.21855.1.S1_at	Hypothetical protein	-5.44
Mtr.25985.1.S1_at	L-asparaginase	-5.44
Mtr.31928.1.S1_at	Peroxidase 25	-5.43
Mtr.25648.1.S1_at	Amino acid transporter	-5.43
Mtr.43000.1.S1_at	Hypothetical protein	-5.43
Mtr.7095.1.S1_at	Na+/H+ antiporter NHX6	-5.43
Mtr.4424.1.S1_at	Hypothetical protein	-5.42
Mtr.5882.1.S1_at	Hypothetical protein	-5.42
Mtr.9162.1.S1_at	Carboxypeptidase type III	-5.40
Mtr.44395.1.S1_at	Hypothetical protein	-5.40
Mtr.32559.1.S1_at	Pherophorin	-5.40
Mtr.11162.1.S1_at	GTP-binding protein LepA	-5.39
Mtr.37476.1.S1_at	Bimodular protein	-5.38
Mtr.13378.1.S1_at	Zinc finger protein 4	-5.38
Mtr.33770.1.S1_at	Hypothetical protein	-5.38
Mtr.9483.1.S1_at	Hypothetical protein	-5.38
Mtr.43824.1.S1_at	Thioredoxin M-type 3	-5.38
Mtr.9796.1.S1_at	Hypothetical protein	-5.37
Mtr.5041.1.S1_s_at	Alcohol dehydrogenase	-5.37
Mtr.35108.1.S1_at	Hypothetical protein	-5.37
Mtr.11736.1.S1_at	Aquaporin NIP-type	-5.37
Msa.2747.1.S1_at	Hypothetical protein	-5.36
Msa.3120.1.S1_at	Hypothetical protein	-5.36
Mtr.13054.1.S1_at	Wall-associated kinase	-5.35
Mtr.46001.1.S1_s_at	Hypothetical protein	-5.35
Mtr.39027.1.S1_s_at	Hypothetical protein	-5.35

Mtr.2881.1.S1_at	Hypothetical protein	-5.34
Mtr.9540.1.S1_at	RING finger protein	-5.34
Mtr.10799.1.S1_at	Multi resistance protein	-5.34
Mtr.29262.1.S1_at	3-hydroxy-3-methylglutaryl-coenzyme A reductase 2	-5.34
Mtr.52072.1.S1_at	Hypothetical protein	-5.34
Mtr.11265.1.S1_at	Glycosyl hydrolase	-5.33
Mtr.9508.1.S1_at	Leucine rich repeat-like protein	-5.33
Msa.3151.1.S1_at	Hypothetical protein	-5.33
Mtr.35849.1.S1_at	Hypothetical protein	-5.32
Mtr.4814.1.S1_at	AROGP2	-5.32
Msa.1835.1.S1_at	Hypothetical protein	-5.32
Msa.1588.1.S1_at	Hypothetical protein	-5.32
Mtr.40105.1.S1_at	Hypothetical protein	-5.32
Mtr.9532.1.S1_at	Hypothetical protein	-5.32
Mtr.40716.1.S1_at	WAP four-disulfide core domain protein 1	-5.31
Mtr.11721.1.S1_at	Lycopene epsilon-cyclase	-5.31
Mtr.45322.1.S1_at	Hypothetical protein	-5.31
Mtr.8501.1.S1_s_at	Glycolate oxidase	-5.30
Msa.1877.1.S1_at	Hypothetical protein	-5.30
Mtr.8575.1.S1_x_at	Glucosyltransferase-13	-5.30
Mtr.19596.1.S1_at	TPR repeat protein	-5.30
Mtr.41539.1.S1_at	Hypothetical protein	-5.30
Mtr.27682.1.S1_at	NS5 protein	-5.30
Mtr.39009.1.S1_at	Serine-rich protein	-5.29
Mtr.13736.1.S1_at	Senescence-associated protein SAG102	-5.29
Mtr.40668.1.S1_at	Hypothetical protein	-5.29
Mtr.2587.1.S1_at	Membrane-associated salt-inducible protein	-5.29
Mtr.14004.1.S1_at	Hypothetical protein	-5.28
Mtr.37696.1.S1_at	Hypothetical protein	-5.28
Mtr.29983.1.S1_s_at	Beta-carotene hydroxylase	-5.28
Mtr.6706.1.S1_at	Hypothetical protein	-5.28
Mtr.9045.1.S1_at	Carbonate dehydratase	-5.27
Mtr.40379.1.S1_at	Leucoanthocyanidin dioxygenase	-5.27
Mtr.41850.1.S1_at	Hypothetical protein	-5.27
Mtr.9347.1.S1_at	Hypothetical protein	-5.27
Mtr.37762.1.S1_at	Glutathione S-transferase GST 9	-5.27
Mtr.32117.1.S1_at	Hypothetical protein	-5.26
Mtr.13579.1.S1_at	Photoreceptor-interacting transcription factor	-5.26
Msa.3148.1.S1_at	Hypothetical protein	-5.26
Mtr.8435.1.S1_at	Lipoxygenase	-5.26
Mtr.12179.1.S1_at	Hypothetical protein	-5.26
Mtr.34494.1.S1_at	Hypothetical protein	-5.25
Mtr.32495.1.S1_at	Protein kinase	-5.25
Mtr.26217.1.S1_at	Auxin response factor 5	-5.24
Mtr.31555.1.S1_at	Beta-1,3-glucanase	-5.24
Mtr.8925.1.S1_s_at	Signal recognition particle 54 kDa subunit	-5.24
Mtr.38360.1.S1_at	Hypothetical protein	-5.24
Mtr.37072.1.S1_at	Hypothetical protein	-5.23
Mtr.43001.1.S1_at	Aminotransferase 2	-5.23
Mtr.1749.1.S1_s_at	MutT domain protein	-5.23
Mtr.12080.1.S1_s_at	Hypothetical protein	-5.22
Mtr.5510.1.S1_at	Hypothetical protein	-5.21
Mtr.39594.1.S1_at	Trehalose-6-phosphate phosphatase	-5.21
Mtr.44856.1.S1_at	Beta-amylase	-5.20

Mtr.4407.1.S1_at	Transcription factor, CCAAT-binding	-5.20
Mtr.35779.1.S1_at	Seed maturation protein PM27	-5.20
Mtr.29279.1.S1_at	Lipoxygenase	-5.20
Mtr.19280.1.S1_at	Multi antimicrobial extrusion protein MatE	-5.20
Msa.2593.1.S1_at	Hypothetical protein	-5.20
Mtr.5570.1.S1_at	Plasma membrane sulphate transporter	-5.19
Mtr.7911.1.S1_s_at	ER1-like receptor kinase	-5.19
Mtr.40297.1.S1_at	Cytochrome b6f complex subunit	-5.18
Msa.926.1.S1_at	Hypothetical protein	-5.18
Mtr.22047.1.S1_at	Hypothetical protein	-5.18
Mtr.25643.1.S1_at	B-type cyclin	-5.18
Mtr.37192.1.S1_at	Hypothetical protein	-5.18
Msa.473.1.S1_at	Hypothetical protein	-5.18
Mtr.17452.1.S1_at	Glycoside transferase	-5.18
Mtr.41066.1.S1_at	Hypothetical protein	-5.17
Mtr.9062.1.S1_at	Hypothetical protein	-5.17
Mtr.17195.1.S1_at	RNA-binding region RNP-1	-5.17
Mtr.39734.1.S1_at	Glutathione S-transferase	-5.16
Mtr.18673.1.S1_at	Esterase/lipase/thioesterase	-5.15
Mtr.12679.1.S1_at	Peroxiredoxin-like protein	-5.15
Mtr.40437.1.S1_at	ABC transporter	-5.15
Mtr.32399.1.S1_at	Hypothetical protein	-5.14
Mtr.6734.1.S1_s_at	Hypothetical protein	-5.14
Mtr.50716.1.S1_s_at	Glycosyl transferase	-5.13
Mtr.37688.1.S1_at	Hypothetical protein	-5.13
Mtr.2641.1.S1_at	Hypothetical protein	-5.13
Msa.1523.1.S1_at	Hypothetical protein	-5.12
Mtr.40232.1.S1_s_at	Geranylgeranyl hydrogenase	-5.12
Mtr.44687.1.S1_at	Hypothetical protein	-5.12
Mtr.35286.1.S1_at	Twist related protein 1	-5.11
Mtr.29418.1.S1_at	Hypothetical protein	-5.11
Msa.2895.1.S1_at	Hypothetical protein	-5.10
Mtr.43042.1.S1_at	LEDI-5c protein	-5.09
Mtr.10615.1.S1_at	Cellulose synthase catalytic subunit	-5.09
Mtr.39209.1.S1_at	Hypothetical protein	-5.09
Mtr.6365.1.S1_at	Acetolactate synthase	-5.09
Mtr.43323.1.S1_at	Polygalacturonase-like protein	-5.09
Mtr.10685.1.S1_at	Hypothetical protein	-5.09
Mtr.39386.1.S1_at	Hypothetical protein	-5.09
Mtr.39345.1.S1_at	Oligopeptidase B	-5.08
Mtr.40321.1.S1_at	Hydroperoxide lyase	-5.08
Msa.1503.1.S1_at	Hypothetical protein	-5.08
Mtr.21060.1.S1_at	Peptidyl-prolyl cis-trans isomerase, cyclophilin type	-5.08
Mtr.15949.1.S1_at	Hypothetical protein	-5.07
Mtr.24898.1.S1_at	Magnesium/proton exchanger (MHX1)	-5.07
Mtr.12831.1.S1_at	NBD-like protein	-5.07
Mtr.8447.1.S1_s_at	Hypothetical protein	-5.07
Mtr.14157.1.S1_at	Gibberellin regulated protein	-5.06
Mtr.27855.1.S1_at	Heat shock protein	-5.06
Mtr.44031.1.S1_at	Hypothetical protein	-5.06
Mtr.32711.1.S1_at	Hypothetical protein	-5.06
Mtr.34769.1.S1_at	Hypothetical protein	-5.06
Mtr.22479.1.S1_at	Anthocyanin 1	-5.05
Mtr.15921.1.S1_at	Hypothetical protein	-5.05

Mtr.52045.1.S1_at	Hypothetical protein	-5.05
Mtr.43891.1.S1_at	Hypothetical protein	-5.05
Mtr.40723.1.S1_at	Hypothetical protein	-5.04
Mtr.12672.1.S1_at	Cytochrome P-450 71A1	-5.04
Mtr.31344.1.S1_at	Oxidoreductase-like protein	-5.04
Mtr.4077.1.S1_at	Hypothetical protein	-5.04
Mtr.37359.1.S1_at	50S ribosomal protein L18	-5.04
Msa.1282.1.S1_at	Hypothetical protein	-5.04
Mtr.34452.1.S1_at	Hypothetical protein	-5.04
Mtr.8397.1.S1_at	Anthranilate synthase alpha subunit	-5.03
Mtr.12931.1.S1_at	Hypothetical protein	-5.03
Mtr.48824.1.S1_at	tRNA isopentenyltransferase	-5.02
Mtr.13781.1.S1_at	Hypothetical protein	-5.02
Mtr.44321.1.S1_at	Hypothetical protein	-5.02
Mtr.13442.1.S1_at	Hypothetical protein	-5.01
Mtr.13038.1.S1_at	Hypothetical protein	-5.01
Msa.3079.1.S1_at	Hypothetical protein	-5.01
Mtr.42974.1.S1_at	Hydroxyisourate hydrolase	-5.01
Mtr.9427.1.S1_at	Hypothetical protein	-5.01
Mtr.785.1.S1_s_at	Hypothetical protein	-5.01
Mtr.32662.1.S1_at	Beta-amylase	-5.00
Mtr.31160.1.S1_at	En/Spm-like transposon protein	-5.00
Mtr.51697.1.S1_at	Hypothetical protein	-4.99
Mtr.44918.1.S1_at	Lysophospholipase 1 precursor	-4.99
Mtr.44756.1.S1_at	Hypothetical protein	-4.99
Mtr.38455.1.S1_at	Hypothetical protein	-4.99
Mtr.12359.1.S1_at	Hypothetical protein	-4.99
Mtr.44620.1.S1_at	Polygalacturonase	-4.99
Mtr.4684.1.S1_s_at	Hypothetical protein	-4.98
Mtr.44847.1.S1_at	Glycosyltransferase family protein 47	-4.98
Mtr.1796.1.S1_at	Hypothetical protein	-4.98
Mtr.4037.1.S1_at	Hypothetical protein	-4.98
Mtr.43771.1.S1_at	Hypothetical protein	-4.98
Mtr.28729.1.S1_at	Serine protease-like protein	-4.98
Mtr.13670.1.S1_at	Hypothetical protein	-4.97
Mtr.44765.1.S1_at	Lycopene epsilon cyclase	-4.97
Mtr.3869.1.S1_at	Hypothetical protein	-4.97
Mtr.49874.1.S1_at	Zn-finger, RING	-4.97
Mtr.6322.1.S1_s_at	Cytochrome P450	-4.96
Mtr.43371.1.S1_at	Peroxidase	-4.96
Mtr.8269.1.S1_at	Hypothetical protein	-4.96
Mtr.12754.1.S1_at	Hypothetical protein	-4.95
Mtr.10170.1.S1_at	Hypothetical protein	-4.95
Mtr.40305.1.S1_at	Hypothetical protein	-4.95
Mtr.15024.1.S1_at	Hypothetical protein	-4.95
Mtr.3201.1.S1_at	LysM domain-containing receptor-like kinase 1	-4.95
Mtr.24230.1.S1_at	Isoflavone reductase	-4.95
Msa.2963.1.S1_at	Hypothetical protein	-4.94
Msa.1405.1.S1_at	Hypothetical protein	-4.94
Mtr.21631.1.S1_at	Peptide chain release factor 2	-4.94
Mtr.31392.1.S1_s_at	Desiccation protectant protein Lea14	-4.94
Mtr.15326.1.S1_at	ABA/WDS induced protein	-4.94
Mtr.8478.1.S1_at	Cytochrome P450	-4.94
Mtr.27870.1.S1_at	Hypothetical protein	-4.93

Mtr.30845.1.S1_at	Nodulin-like protein	-4.93
Mtr.44755.1.S1_at	LRR-like protein	-4.93
Msa.1150.1.S1_at	Hypothetical protein	-4.93
Msa.1689.1.S1_at	Hypothetical protein	-4.93
Mtr.13829.1.S1_at	Hypothetical protein	-4.93
Mtr.29249.1.S1_at	Hypothetical protein	-4.92
Mtr.7993.1.S1_s_at	Glyceraldehyde-3-phosphate dehydrogenase	-4.92
Mtr.50857.1.S1_at	Peptidase S10	-4.92
Mtr.14245.1.S1_at	Hypothetical protein	-4.92
Mtr.5792.1.S1_at	Hypothetical protein	-4.91
Mtr.38815.1.S1_at	Hypothetical protein	-4.91
Mtr.5786.1.S1_at	MAP kinase	-4.91
Msa.2707.1.S1_at	Hypothetical protein	-4.91
Msa.1083.1.S1_at	Hypothetical protein	-4.90
Mtr.1621.1.S1_s_at	Hypothetical protein	-4.90
Msa.985.1.S1_at	Hypothetical protein	-4.90
Mtr.38245.1.S1_at	Transcription factor RAU1	-4.90
Mtr.20901.1.S1_s_at	Heat shock protein Hsp70	-4.89
Mtr.38047.1.S1_at	Monogalactosyldiacylglycerol synthase	-4.89
Mtr.28777.1.S1_at	Hypothetical protein	-4.89
Mtr.33440.1.S1_at	30S ribosomal protein S16	-4.88
Mtr.44455.1.S1_at	Hypothetical protein	-4.88
Mtr.41614.1.S1_at	Hypothetical protein	-4.88
Mtr.9382.1.S1_at	Serine carboxypeptidase II	-4.88
Mtr.8504.1.S1_at	Hypothetical protein	-4.88
Mtr.9861.1.S1_at	Hypothetical protein	-4.88
Mtr.9495.1.S1_at	Sugar transporter	-4.87
Mtr.34855.1.S1_at	Receptor-like protein kinase 2	-4.87
Mtr.40550.1.S1_at	Hypothetical protein	-4.87
Mtr.33840.1.S1_at	Hypothetical protein	-4.87
Mtr.25447.1.S1_at	Hypothetical protein	-4.87
Mtr.40978.1.S1_at	Hypothetical protein	-4.87
Mtr.39502.1.S1_at	Hypothetical protein	-4.87
Mtr.223.1.S1_at	Photolyase/blue-light receptor	-4.86
Mtr.831.1.S1_at	Hypothetical protein	-4.86
Mtr.8926.1.S1_at	Alpha-D-xylosidase	-4.86
Mtr.41057.1.S1_at	Hypothetical protein	-4.85
Mtr.27040.1.S1_s_at	Hypothetical protein	-4.85
Mtr.7486.1.S1_s_at	Zinc finger protein	-4.85
Mtr.34765.1.S1_at	Hypothetical protein	-4.85
Mtr.8572.1.S1_at	Thaumatin-like protein	-4.84
Msa.3056.1.S1_at	Hypothetical protein	-4.84
Mtr.42503.1.S1_at	Isoflavonoid glucosyltransferase	-4.84
Mtr.784.1.S1_s_at	Hypothetical protein	-4.84
Mtr.11698.1.S1_at	APETALA3	-4.84
Mtr.10231.1.S1_at	Hypothetical protein	-4.84
Mtr.49132.1.S1_at	Ferredoxin	-4.84
Mtr.37806.1.S1_at	Cellulose synthase	-4.83
Mtr.42284.1.S1_at	Hypothetical protein	-4.83
Mtr.9325.1.S1_at	Hypothetical protein	-4.83
Mtr.30134.1.S1_at	Serine carboxipeptidase	-4.83
Mtr.6087.1.S1_at	Hypothetical protein	-4.83
Mtr.50278.1.S1_s_at	Ras GTPase	-4.83
Mtr.27879.1.S1_at	Hypothetical protein	-4.82

Mtr.16894.1.S1_at	Zn-finger, CCHC type	-4.82
Msa.341.1.S1_at	Hypothetical protein	-4.82
Mtr.41288.1.S1_at	Hypothetical protein	-4.82
Mtr.40565.1.S1_at	GTP-binding protein	-4.81
Mtr.40744.1.S1_at	Hypothetical protein	-4.81
Mtr.38175.1.S1_at	Hypothetical protein	-4.81
Mtr.33530.1.S1_at	Hypothetical protein	-4.81
Mtr.43506.1.S1_at	Hypothetical protein	-4.81
Mtr.1424.1.S1_at	Hypothetical protein	-4.80
Mtr.11739.1.S1_s_at	Homogentisate phytylprenyltransferase	-4.80
Mtr.27440.1.S1_at	Hypothetical protein	-4.80
Mtr.19208.1.S1_at	Zn-finger, RING	-4.80
Mtr.31664.1.S1_at	Hypothetical protein	-4.79
Mtr.37156.1.S1_at	Hypothetical protein	-4.79
Mtr.42585.1.S1_at	Hypothetical protein	-4.79
Mtr.8942.1.S1_at	Desiccation protectant protein Lea14	-4.79
Mtr.25990.1.S1_at	Hypothetical protein	-4.79
Mtr.37299.1.S1_at	Hypothetical protein	-4.79
Mtr.12856.1.S1_at	Hypothetical protein	-4.79
Mtr.28080.1.S1_at	Anthocyanin 5-aromatic acyltransferase/benzoyltransferase-like protein	-4.79
Mtr.6765.1.S1_at	Hypothetical protein	-4.79
Mtr.31565.1.S1_s_at	Hypothetical protein	-4.78
Mtr.45813.1.S1_at	Hypothetical protein	-4.78
Mtr.40184.1.S1_at	FtsH protease (VAR2)	-4.78
Mtr.11186.1.S1_at	Pyruvate, phosphate dikinase	-4.78
Msa.2997.1.S1_at	Hypothetical protein	-4.78
Mtr.15246.1.S1_at	ABC transporter	-4.78
Mtr.10800.1.S1_s_at	Hypothetical protein	-4.78
Mtr.1405.1.S1_at	Hypothetical protein	-4.78
Mtr.8848.1.S1_at	Alpha-xylosidase	-4.77
Mtr.38223.1.S1_at	Acyltransferase	-4.77
Mtr.41591.1.S1_at	3-ketoacyl-ACP synthase	-4.77
Mtr.51232.1.S1_at	Multi antimicrobial extrusion protein MatE	-4.76
Mtr.17267.1.S1_at	Hypothetical protein	-4.76
Mtr.44793.1.S1_at	Hypothetical protein	-4.76
Mtr.45471.1.S1_at	Hypothetical protein	-4.76
Mtr.45264.1.S1_at	Hypothetical protein	-4.76
Mtr.38761.1.S1_s_at	Cell division protein FtsH-like	-4.76
Msa.1739.1.S1_at	Hypothetical protein	-4.76
Mtr.8878.1.S1_at	Hypothetical protein	-4.76
Mtr.44396.1.S1_at	Hypothetical protein	-4.75
Mtr.11645.1.S1_at	Methionine aminopeptidase	-4.75
Mtr.6008.1.S1_at	Hypothetical protein	-4.75
Mtr.44189.1.S1_at	BEL1-related homeotic protein 14	-4.75
Mtr.29172.1.S1_at	Hypothetical protein	-4.74
Mtr.13501.1.S1_at	Alanyl-tRNA synthetase	-4.74
Mtr.43269.1.S1_at	30S ribosomal protein S1	-4.74
Mtr.37893.1.S1_s_at	Cytochrome P450 monooxygenase	-4.74
Mtr.6354.1.S1_at	TPR-containing protein	-4.73
Mtr.37429.1.S1_at	Cytosolic aldehyde dehydrogenase	-4.73
Mtr.20847.1.S1_at	MtN3 and saliva related transmembrane protein	-4.73
Mtr.20846.1.S1_at	MtN3 and saliva related transmembrane protein	-4.73
Mtr.50278.1.S1_at	Ras GTPase	-4.73
Mtr.713.1.S1_s_at	Nucleotide pyrophosphatase	-4.73

Mtr.8877.1.S1_at	Hypothetical protein	-4.73
Mtr.35108.1.S1_s_at	Hypothetical protein	-4.72
Mtr.28002.1.S1_at	Hypothetical protein	-4.72
Mtr.10212.1.S1_at	Hypothetical protein	-4.72
Mtr.7389.1.S1_s_at	Yippee-like protein	-4.72
Mtr.13786.1.S1_at	Hypothetical protein	-4.72
Msa.911.1.S1_at	Hypothetical protein	-4.71
Mtr.9270.1.S1_at	Hypothetical protein	-4.71
Mtr.15385.1.S1_at	UbiA prenyltransferase	-4.71
Mtr.8460.1.S1_s_at	Pathogen-inducible alpha-dioxygenase	-4.70
Mtr.41249.1.S1_at	30S ribosomal protein S13	-4.70
Mtr.25580.1.S1_at	Hypothetical protein	-4.70
Mtr.44600.1.S1_at	Hypothetical protein	-4.70
Mtr.41953.1.S1_at	Hypothetical protein	-4.70
Mtr.6062.1.S1_at	Hypothetical protein	-4.70
Msa.2749.1.S1_at	Hypothetical protein	-4.70
Mtr.45294.1.S1_at	Methionine synthase	-4.70
Mtr.24815.1.S1_at	Glutaredoxin protein	-4.70
Mtr.36918.1.S1_at	Hypothetical protein	-4.69
Mtr.33410.1.S1_at	Hypothetical protein	-4.69
Mtr.14223.1.S1_at	bHLH DNA-binding	-4.69
Mtr.42658.1.S1_at	Ski protein	-4.69
Mtr.50224.1.S1_s_at	Hypothetical protein	-4.68
Mtr.45146.1.S1_at	Apolipoprotein D	-4.68
Mtr.32895.1.S1_at	Hypothetical protein	-4.68
Mtr.46498.1.S1_at	Hypothetical protein	-4.68
Mtr.39361.1.S1_at	Glucanase like protein	-4.67
Mtr.28688.1.S1_at	Hypothetical protein	-4.67
Mtr.31359.1.S1_at	Hypothetical protein	-4.67
Mtr.9831.1.S1_at	Mechanosensitive ion channel domain-containing protein	-4.66
Mtr.11222.1.S1_at	Hypothetical protein	-4.66
Mtr.39292.1.S1_at	Hypothetical protein	-4.66
Mtr.9268.1.S1_at	Hypothetical protein	-4.65
Mtr.20398.1.S1_at	Hypothetical protein	-4.65
Mtr.35557.1.S1_s_at	Hypothetical protein	-4.65
Mtr.37441.1.S1_s_at	Alcohol dehydrogenase 1	-4.65
Mtr.32351.1.S1_at	Hypothetical protein	-4.64
Mtr.29176.1.S1_at	Metal-dependent phosphohydrolase	-4.64
Mtr.40976.1.S1_at	Transcription factor Hap5a	-4.64
Mtr.42512.1.S1_at	Sugar transporter-like protein	-4.64
Mtr.44149.1.S1_at	Aquaporin	-4.64
Mtr.12572.1.S1_at	Hypothetical protein	-4.64
Mtr.13967.1.S1_at	Phosphoenolpyruvate carboxylase	-4.64
Mtr.43992.1.S1_at	Methionine synthase	-4.64
Mtr.44100.1.S1_at	Hypothetical protein	-4.64
Msa.1844.1.S1_at	Hypothetical protein	-4.63
Mtr.37759.1.S1_at	ERD4 protein	-4.63
Mtr.44012.1.S1_at	Hypothetical protein	-4.63
Mtr.8416.1.S1_s_at	Cytochrome b	-4.63
Msa.399.1.S1_at	Hypothetical protein	-4.63
Mtr.37531.1.S1_at	Hypothetical protein	-4.62
Mtr.28232.1.S1_s_at	RNA helicase-like protein	-4.62
Msa.1765.1.S1_at	Hypothetical protein	-4.62
Mtr.34734.1.S1_at	High affinity potassium transporter 2	-4.62

Mtr.39922.1.S1_at	Hypothetical protein	-4.62
Mtr.44759.1.S1_at	E-beta-ocimene synthase	-4.62
Mtr.8392.1.S1_at	4-alpha-glucanotransferase	-4.61
Mtr.43806.1.S1_at	Hypothetical protein	-4.61
Mtr.8609.1.S1_at	Photosystem II reaction center	-4.61
Mtr.12532.1.S1_at	Patatin-like protein 1	-4.59
Mtr.41127.1.S1_at	Pherophorin	-4.59
Mtr.16995.1.S1_at	Hypothetical protein	-4.59
Mtr.52127.1.S1_at	2OG-Fe(II) oxygenase	-4.59
Mtr.34680.1.S1_at	Actin-related protein 8B	-4.59
Mtr.10882.1.S1_at	Hypothetical protein	-4.58
Mtr.10607.1.S1_at	PS60 protein	-4.58
Mtr.44063.1.S1_at	Hypothetical protein	-4.57
Mtr.5456.1.S1_at	Valine-tRNA ligase	-4.57
Mtr.18567.1.S1_at	Pyruvate kinase	-4.57
Mtr.40869.1.S1_at	Hypothetical protein	-4.56
Mtr.44730.1.S1_at	Nitrate transporter	-4.56
Mtr.50181.1.S1_s_at	Vitamin B6 biosynthesis protein	-4.56
Mtr.42104.1.S1_at	Heterotrimeric G-protein gamma subunit 2	-4.56
Mtr.50437.1.S1_s_at	Hypothetical protein	-4.56
Mtr.9652.1.S1_at	Hypothetical protein	-4.56
Mtr.7062.1.S1_at	Hypothetical protein	-4.56
Mtr.41933.1.S1_at	Kexin	-4.56
Mtr.16129.1.S1_at	Hypothetical protein	-4.55
Msa.1589.1.S1_at	Hypothetical protein	-4.55
Mtr.41133.1.S1_at	Bell-like homeodomain protein 3	-4.55
Mtr.2656.1.S1_at	Serine/threonine protein kinase	-4.55
Msa.3170.1.S1_at	Hypothetical protein	-4.55
Mtr.42432.1.S1_at	Hypothetical protein	-4.55
Mtr.14334.1.S1_at	Cytochrome b561	-4.54
Mtr.11185.1.S1_at	SEX1	-4.54
Mtr.31151.1.S1_at	Hypothetical protein	-4.54
Mtr.37091.1.S1_at	Hypothetical protein	-4.54
Mtr.20860.1.S1_at	Hypothetical protein	-4.54
Mtr.41900.1.S1_at	Hypothetical protein	-4.54
Mtr.46514.1.S1_x_at	Alpha/beta-amyrin synthase	-4.53
Mtr.9709.1.S1_at	Hypothetical protein	-4.53
Msa.2217.1.S1_at	Hypothetical protein	-4.53
Mtr.39889.1.S1_at	Pherophorin - like protein	-4.53
Mtr.8981.1.S1_at	Hypothetical protein	-4.53
Mtr.12904.1.S1_at	Pectinacetylesterase	-4.53
Mtr.37315.1.S1_at	Glycolate oxidase	-4.53
Mtr.31155.1.S1_at	Neutral invertase	-4.52
Mtr.36792.1.S1_at	Hypothetical protein	-4.52
Mtr.41434.1.S1_at	Threonine synthase	-4.52
Mtr.49018.1.S1_at	30S ribosomal protein S31	-4.52
Mtr.46510.1.S1_at	Alpha/beta-amyrin synthase	-4.52
Mtr.13573.1.S1_at	Cleavage stimulation factor 50	-4.52
Mtr.13885.1.S1_at	Actin-like protein	-4.52
Mtr.44737.1.S1_at	Hypothetical protein	-4.51
Mtr.44783.1.S1_at	Hypothetical protein	-4.51
Mtr.33734.1.S1_at	Phosphoglycerate mutase	-4.51
Msa.1903.1.S1_at	Hypothetical protein	-4.51
Mtr.10566.1.S1_at	Hypothetical protein	-4.51

Msa.2882.1.S1_at	Hypothetical protein	-4.51
Mtr.37256.1.S1_at	Hypothetical protein	-4.51
Mtr.40913.1.S1_at	Hypothetical protein	-4.51
Mtr.13198.1.S1_at	PHAP2B protein	-4.51
Mtr.8830.1.S1_at	Hypothetical protein	-4.51
AFFX-Mtr-gsta-5_at	2,4-D inducible glutathione S-transferase	-4.50
Mtr.27901.1.S1_at	Hypothetical protein	-4.50
Msa.2125.1.S1_at	Hypothetical protein	-4.50
Mtr.43912.1.S1_at	Hypothetical protein	-4.50
Mtr.23937.1.S1_at	Hypothetical protein	-4.49
Mtr.20125.1.S1_at	Glycoside hydrolase	-4.49
Mtr.28675.1.S1_s_at	Polygalacturonase	-4.49
Mtr.33742.1.S1_at	Acyl-CoA thioesterase	-4.49
Mtr.11277.1.S1_at	Hydroxymethylglutaryl-CoA lyase	-4.49
Mtr.39481.1.S1_at	Hypothetical protein	-4.49
Mtr.30692.1.S1_at	Receptor-protein kinase	-4.49
Mtr.10045.1.S1_at	Hypothetical protein	-4.49
Msa.2782.1.S1_at	Hypothetical protein	-4.48
Mtr.5904.1.S1_at	Salt-inducible protein	-4.48
Mtr.44684.1.S1_at	Nucleotide pyrophosphatase-like protein	-4.48
Msa.3039.1.S1_at	Hypothetical protein	-4.48
Mtr.50233.1.S1_at	Initiation factor 3	-4.47
Mtr.4684.1.S1_at	Hypothetical protein	-4.47
Mtr.40458.1.S1_at	Hypothetical protein	-4.47
Mtr.44377.1.S1_at	Ferric leghemoglobin reductase	-4.47
Mtr.10065.1.S1_at	Hypothetical protein	-4.47
Mtr.32860.1.S1_s_at	Hypothetical protein	-4.47
Mtr.45556.1.S1_at	Actin-related protein 8A	-4.46
Mtr.6093.1.S1_s_at	Subtilisin-like protease	-4.46
Mtr.44182.1.S1_s_at	LEA protein	-4.46
Mtr.41835.1.S1_at	Hypothetical protein	-4.46
Mtr.29095.1.S1_at	Respiratory burst oxidase homolog	-4.46
Mtr.47127.1.S1_at	Far-red impaired response protein	-4.46
Mtr.38114.1.S1_at	Thioredoxin M-type 4	-4.46
Mtr.26195.1.S1_at	Hypothetical protein	-4.46
Mtr.43826.1.S1_at	Hypothetical protein	-4.45
Mtr.27418.1.S1_at	Hypothetical protein	-4.45
Mtr.42095.1.S1_at	Receptor like protein	-4.45
Mtr.33779.1.S1_at	Threonyl-tRNA synthetase	-4.44
Mtr.9679.1.S1_at	Thioredoxin 3	-4.44
Mtr.1092.1.S1_at	ABC transporter	-4.43
Mtr.45276.1.S1_at	LIM domain containing protein	-4.43
Mtr.15418.1.S1_at	Histone deacetylase	-4.43
Mtr.10656.1.S1_at	Sucrose-phosphate synthase	-4.43
Mtr.33111.1.S1_at	Hypothetical protein	-4.43
Mtr.5125.1.S1_s_at	Isoflavone reductase	-4.42
Mtr.43995.1.S1_at	Hypothetical protein	-4.42
Mtr.33105.1.S1_at	Hypothetical protein	-4.42
Mtr.22791.1.S1_at	UDP-glucose pyrophosphorylase	-4.42
Mtr.47389.1.S1_at	Hypothetical protein	-4.41
Mtr.45509.1.S1_at	Hypothetical protein	-4.41
Mtr.10186.1.S1_at	Hypothetical protein	-4.41
Mtr.40332.1.S1_at	5,10-methylenetetrahydrofolate dehydrogenase	-4.41
Mtr.9095.1.S1_at	30S ribosomal protein S9	-4.41

Mtr.12803.1.S1_at	Pyridoxamine 5-phosphate oxidase	-4.41
Mtr.35932.1.S1_at	Na+/H+ antiporter	-4.40
Mtr.49926.1.S1_at	Mitochondrial carrier protein	-4.40
Mtr.49527.1.S1_at	Lipolytic enzyme	-4.40
Mtr.4981.1.S1_at	Dehydration-responsive protein	-4.40
Mtr.37849.1.S1_at	Fe-superoxide dismutase	-4.40
Mtr.6699.1.S1_at	Lhcb2 protein (Light harvesting chlorophyll a/b-binding protein)	-4.40
Mtr.49774.1.S1_at	Auxin responsive SAUR protein	-4.38
Msa.1658.1.S1_at	Hypothetical protein	-4.38
Mtr.32040.1.S1_at	Hypothetical protein	-4.38
Mtr.553.1.S1_at	Hypothetical protein	-4.38
Mtr.43492.1.S1_at	Hypothetical protein	-4.37
Mtr.45329.1.S1_at	Protein kinase	-4.37
Mtr.12143.1.S1_at	Hypothetical protein	-4.37
Mtr.9855.1.S1_at	Hypothetical protein	-4.37
Mtr.9284.1.S1_at	Hypothetical protein	-4.37
Msa.3138.1.S1_at	Hypothetical protein	-4.37
Mtr.19329.1.S1_at	Hypothetical protein	-4.37
Mtr.43427.1.S1_at	Hypothetical protein	-4.37
Mtr.9651.1.S1_at	Transcription factor	-4.36
Mtr.38029.1.S1_at	Chloroplast 50S ribosomal protein L31	-4.36
Mtr.28730.1.S1_at	En/Spm-like transposon protein	-4.36
Msa.2936.1.S1_at	Hypothetical protein	-4.35
Mtr.18153.1.S1_at	Hypothetical protein	-4.35
Msa.2836.1.S1_at	Hypothetical protein	-4.35
Mtr.51659.1.S1_at	Hypothetical protein	-4.35
Mtr.4792.1.S1_at	Leaf ubiquitous urease	-4.35
Mtr.37175.1.S1_at	Chlorophyll a/b binding protein	-4.35
Mtr.33726.1.S1_at	Sugar transporter-like protein	-4.35
Mtr.37336.1.S1_at	RNA-binding protein 2	-4.34
Mtr.35494.1.S1_at	Hypothetical protein	-4.34
Mtr.13750.1.S1_at	Ent-kaurenoic acid hydroxylase	-4.34
Mtr.9793.1.S1_at	4-coumarate-CoA ligase	-4.34
Mtr.43083.1.S1_s_at	Phosphoglucomutase	-4.34
Mtr.20942.1.S1_x_at	Serine/threonine protein kinase	-4.34
Mtr.10248.1.S1_at	Peroxidase	-4.34
Mtr.17374.1.S1_at	C4-dicarboxylate transporter/malic acid transport protein	-4.33
Msa.3084.1.S1_at	Hypothetical protein	-4.33
Mtr.5253.1.S1_s_at	Hypothetical protein	-4.33
Mtr.27203.1.S1_at	Hypothetical protein	-4.32
Mtr.6724.1.S1_at	Hypothetical protein	-4.32
Mtr.9728.1.S1_at	Hypothetical protein	-4.32
Mtr.19007.1.S1_at	Barwin-related endoglucanase	-4.32
Mtr.19679.1.S1_s_at	Phospholipase/Carboxylesterase	-4.32
Mtr.49120.1.S1_s_at	Cysteine synthase A	-4.32
Mtr.12363.1.S1_at	Hypothetical protein	-4.32
Mtr.11841.1.S1_at	Lecithine cholesterol acyltransferase	-4.31
Mtr.10198.1.S1_at	Phosphoenolpyruvate carboxylase	-4.31
Mtr.10917.1.S1_at	Cytochrome P450 77A3	-4.31
Mtr.34451.1.S1_at	Hypothetical protein	-4.31
Mtr.40408.1.S1_at	Photosystem II	-4.31
Mtr.27207.1.S1_s_at	Hypothetical protein	-4.30
Mtr.42357.1.S1_at	Hypothetical protein	-4.30
Mtr.5029.1.S1_at	Hypothetical protein	-4.30

Mtr.27078.1.S1_at	Selenium-binding protein	-4.30
Mtr.19577.1.S1_s_at	Aldehyde decarbonylase	-4.30
Mtr.48940.1.S1_at	Protein phosphatase 2C	-4.30
Mtr.27854.1.S1_s_at	NADP-dependent oxidoreductase P1	-4.29
Mtr.48994.1.S1_at	Hypothetical protein	-4.29
Mtr.43518.1.S1_at	Plastidic cysteine synthase 1	-4.29
Mtr.44721.1.S1_at	Rust resistance kinase Lr10	-4.29
Mtr.49166.1.S1_at	Short-chain dehydrogenase/reductase	-4.28
Mtr.38453.1.S1_at	Proline-rich protein	-4.28
Mtr.34176.1.S1_at	Hypothetical protein	-4.28
Mtr.46448.1.S1_s_at	Hypothetical protein	-4.28
Mtr.25077.1.S1_at	Hypothetical protein	-4.28
Mtr.13648.1.S1_at	Hypothetical protein	-4.28
Mtr.41052.1.S1_at	RING zinc finger protein	-4.28
Mtr.15256.1.S1_at	RNA-binding region RNP-1	-4.27
Mtr.33506.1.S1_at	Hypothetical protein	-4.27
Mtr.41461.1.S1_at	Hypothetical protein	-4.27
Mtr.43677.1.S1_at	50S ribosomal protein L5	-4.27
Mtr.37442.1.S1_at	Alcohol dehydrogenase 1	-4.27
Mtr.33027.1.S1_at	Hypothetical protein	-4.26
Mtr.44561.1.S1_at	Threonine synthase	-4.26
Mtr.32024.1.S1_at	Hypothetical protein	-4.26
Mtr.10800.1.S1_at	Hypothetical protein	-4.26
Mtr.24083.1.S1_s_at	Uroporphyrinogen decarboxylase	-4.26
Mtr.11319.1.S1_at	Hypothetical protein	-4.25
Mtr.31577.1.S1_at	Phosphoribosylformimino-5-aminoimidazole carboxamide ribotide isomerase	-4.25
Mtr.7450.1.S1_at	Hypothetical protein	-4.25
Mtr.32688.1.S1_at	Hypothetical protein	-4.25
Mtr.13372.1.S1_at	Hypothetical protein	-4.25
Mtr.37642.1.S1_at	NADP-dependent oxidoreductase P2	-4.25
Mtr.9263.1.S1_at	Glycine-tRNA ligase	-4.25
Mtr.43678.1.S1_at	Hypothetical protein	-4.24
Mtr.5745.1.S1_at	Receptor-like protein kinase	-4.24
Mtr.37436.1.S1_at	Hypersensitive-induced response protein	-4.24
Mtr.44590.1.S1_s_at	Glycuronosyltransferase-like protein	-4.24
Msa.2909.1.S1_at	Hypothetical protein	-4.23
Mtr.9820.1.S1_at	MADS box protein GHMADS-2	-4.23
Mtr.38346.1.S1_at	Hypothetical protein	-4.23
Mtr.44280.1.S1_at	Avr9/Cf-9 induced kinase 1	-4.23
Msa.2963.1.S1_s_at	Hypothetical protein	-4.23
Mtr.6751.1.S1_at	Hypothetical protein	-4.22
Mtr.1035.1.S1_at	Heat shock protein	-4.22
Mtr.37406.1.S1_at	Hypothetical protein	-4.22
Mtr.39220.1.S1_at	Methyltransferase	-4.22
Mtr.8748.1.S1_at	Methionine synthase	-4.22
Mtr.34318.1.S1_at	Receptor-like protein kinase	-4.22
Mtr.6063.1.S1_at	Glucosyltransferase	-4.21
Mtr.5242.1.S1_at	Cellulose synthase	-4.21
Msa.1378.1.S1_at	Hypothetical protein	-4.21
Mtr.7932.1.S1_s_at	Allyl alcohol dehydrogenase	-4.21
Mtr.43636.1.S1_at	Fructose-6-phosphate 2-kinase	-4.21
Mtr.43250.1.S1_at	Subtilisin-like protease	-4.21
Mtr.9857.1.S1_at	Hypothetical protein	-4.21
Mtr.39618.1.S1_at	Prolyl tRNA synthetase	-4.21

Mtr.13924.1.S1_at	Glucosyltransferase like protein	-4.20
Mtr.8908.1.S1_at	Hypothetical protein	-4.20
Msa.3181.1.S1_at	Hypothetical protein	-4.20
Mtr.32057.1.S1_at	Hypothetical protein	-4.20
Mtr.1427.1.S1_at	Hypothetical protein	-4.19
Mtr.40545.1.S1_at	HSP associated protein	-4.18
Mtr.7633.1.S1_at	Hypothetical protein	-4.18
Mtr.8503.1.S1_at	Photosystem II reaction center W protein	-4.18
Mtr.16895.1.S1_at	Hypothetical protein	-4.18
Mtr.6854.1.S1_at	Two-component system sensor histidine kinase	-4.18
Mtr.8870.1.S1_at	Cytochrome P450 monooxygenase	-4.18
Msa.984.1.S1_at	Hypothetical protein	-4.18
Mtr.33880.1.S1_s_at	Hypothetical protein	-4.18
Mtr.38980.1.S1_at	Isopiperitenone reductase	-4.17
Mtr.42121.1.S1_at	1-aminocyclopropane-1-carboxylate synthase	-4.17
Mtr.40121.1.S1_at	Peroxidase1C	-4.17
Mtr.45183.1.S1_at	Hyaluronan mediated motility receptor	-4.17
Mtr.34408.1.S1_s_at	Hypothetical protein	-4.17
Mtr.13869.1.S1_at	Calcium-activated outward-rectifying potassium channel 5	-4.17
Mtr.13202.1.S1_at	Cellulose synthase CesA-1	-4.17
Mtr.32657.1.S1_s_at	Beta-amylase	-4.17
Mtr.28982.1.S1_at	Hypothetical protein	-4.16
Mtr.20868.1.S1_at	Glycoside hydrolase	-4.16
Mtr.34220.1.S1_at	Hypothetical protein	-4.15
Mtr.42820.1.S1_at	RING/C3HC4/PHD zinc finger	-4.15
Mtr.38478.1.S1_at	Hypothetical protein	-4.15
Msa.1138.1.S1_at	Hypothetical protein	-4.15
Mtr.31405.1.S1_at	CDR1	-4.15
Mtr.51973.1.S1_at	Seed maturation protein	-4.15
Mtr.41864.1.S1_at	Hypothetical protein	-4.14
Mtr.33046.1.S1_at	Hypothetical protein	-4.14
Mtr.28632.1.S1_at	Hypothetical protein	-4.14
Mtr.11798.1.S1_at	Cellulose synthase	-4.14
Mtr.27903.1.S1_at	High mobility group protein 1	-4.14
Mtr.20900.1.S1_at	Hypothetical protein	-4.14
Mtr.43596.1.S1_at	Plastid ribosomal protein L19	-4.13
Mtr.39493.1.S1_at	Squamosa promoter binding protein-like 8	-4.12
Mtr.16430.1.S1_at	Hypothetical protein	-4.12
Mtr.9623.1.S1_at	Hypothetical protein	-4.12
Mtr.24954.1.S1_s_at	Protein kinase	-4.12
Mtr.29094.1.S1_s_at	Hypothetical protein	-4.11
Mtr.34499.1.S1_at	Hypothetical protein	-4.11
Mtr.12407.1.S1_at	Glutamate decarboxylase	-4.11
Mtr.24235.1.S1_at	Hypothetical protein	-4.11
Mtr.39152.1.S1_at	Mitochondrial half-ABC transporter	-4.10
Mtr.3096.1.S1_at	Glycosyl hydrolase	-4.10
Mtr.6112.1.S1_at	Cytochrome P450	-4.10
Mtr.42749.1.S1_at	Late embryogenesis abundant protein 1	-4.10
Mtr.9891.1.S1_at	Hypothetical protein	-4.10
Mtr.42054.1.S1_at	Glu-tRNA(Gln) amidotransferase subunit A	-4.10
Mtr.40151.1.S1_at	Cellulose synthase	-4.10
Mtr.31183.1.S1_x_at	Long-chain acyl-CoA synthetase 6	-4.09
Mtr.49014.1.S1_at	SNO glutamine amidotransferase	-4.09
Mtr.41015.1.S1_at	Hypothetical protein	-4.09

Msa.2740.1.S1_at	Hypothetical protein	-4.09
Msa.579.1.S1_at	Hypothetical protein	-4.09
Mtr.51597.1.S1_s_at	Hypothetical protein	-4.08
Mtr.10452.1.S1_at	Hypothetical protein	-4.08
Mtr.28467.1.S1_s_at	Aspartyl aminopeptidase	-4.08
Mtr.6148.1.S1_x_at	Serine carboxypeptidase II-2	-4.08
Mtr.40395.1.S1_at	MtN29 protein	-4.08
Mtr.8454.1.S1_at	Hypothetical protein	-4.08
Mtr.43725.1.S1_at	50S ribosomal protein L24	-4.08
Mtr.24829.1.S1_at	Hypothetical protein	-4.08
Mtr.12114.1.S1_at	Cyclophilin-40	-4.08
Mtr.32928.1.S1_s_at	Hypothetical protein	-4.08
Mtr.49845.1.S1_at	Hypothetical protein	-4.08
Mtr.34714.1.S1_at	Hypothetical protein	-4.07
Mtr.40653.1.S1_at	Hypothetical protein	-4.07
Mtr.14580.1.S1_at	Zn-finger, RING	-4.07
Mtr.36941.1.S1_at	Hypothetical protein	-4.07
Mtr.7035.1.S1_at	Hypothetical protein	-4.07
Mtr.38413.1.S1_at	Myb-related transcription factor	-4.07
Mtr.41142.1.S1_at	ABC transporter	-4.07
Mtr.39702.1.S1_at	Hypothetical protein	-4.06
Mtr.41992.1.S1_at	Endo-beta-N-acetylglucosaminidase	-4.06
Mtr.10835.1.S1_s_at	Hypothetical protein	-4.06
Mtr.12644.1.S1_at	Fiber protein Fb34	-4.05
Mtr.27917.1.S1_s_at	Yippee-like protein	-4.05
Mtr.2385.1.S1_at	Hypothetical protein	-4.05
Mtr.22750.1.S1_at	Hypothetical protein	-4.05
Mtr.51607.1.S1_at	Hypothetical protein	-4.05
Mtr.46659.1.S1_at	Hypothetical protein	-4.04
Mtr.1962.1.S1_at	MRP-like ABC transporter	-4.04
Mtr.8807.1.S1_at	Phospholipase-like protein	-4.03
Mtr.10312.1.S1_at	Phytochelatin synthetase	-4.03
Mtr.13998.1.S1_at	Hypothetical protein	-4.03
Mtr.18312.1.S1_at	Lipase	-4.03
Mtr.40545.1.S1_a_at	HSP associated protein	-4.03
Mtr.50857.1.S1_s_at	Peptidase S10	-4.03
Mtr.41769.1.S1_at	Hypothetical protein	-4.03
Mtr.40998.1.S1_at	Hypothetical protein	-4.02
Mtr.6074.1.S1_at	Valine-tRNA ligase	-4.02
Mtr.43590.1.S1_at	Hypothetical protein	-4.02
Mtr.41929.1.S1_at	Phenylalanine-tRNA synthetase	-4.02
Mtr.20309.1.S1_at	H+-transporting two-sector ATPase	-4.02
Mtr.35857.1.S1_at	Hypothetical protein	-4.02
Mtr.5656.1.S1_at	Vacuolar H+-ATPase B subunit	-4.02
Msa.1632.1.S1_at	Hypothetical protein	-4.02
Mtr.43488.1.S1_at	Hypothetical protein	-4.02
Mtr.38754.1.S1_at	Chloride channel protein	-4.02
Mtr.41025.1.S1_at	Hypothetical protein	-4.02
Mtr.50562.1.S1_s_at	Zn-finger, Dof type	-4.02
Mtr.50527.1.S1_at	Hypothetical protein	-4.01
Mtr.12805.1.S1_at	Hypothetical protein	-4.01
Mtr.43459.1.S1_at	Hypothetical protein	-4.01
Msa.2987.1.S1_at	Hypothetical protein	-4.01
Mtr.7078.1.S1_s_at	Peroxidase	-4.01

Mtr.24451.1.S1_at	Hypothetical protein	-4.00
Mtr.44402.1.S1_at	Hypothetical protein	-4.00
Msa.1614.1.S1_at	Hypothetical protein	-4.00
Mtr.21847.1.S1_at	Hypothetical protein	-4.00
Mtr.41151.1.S1_at	Cer2 protein	-3.99
Mtr.37912.1.S1_at	Allyl alcohol dehydrogenase	-3.99
Mtr.4063.1.S1_at	Hypothetical protein	-3.99
Mtr.40525.1.S1_at	Hypothetical protein	-3.99
Mtr.33744.1.S1_at	Hypothetical protein	-3.99
Mtr.29088.1.S1_at	Hypothetical protein	-3.99
Mtr.28159.1.S1_at	AP2 domain transcription factor	-3.98
Mtr.39884.1.S1_at	Hypothetical protein	-3.98
Mtr.12922.1.S1_at	Hypothetical protein	-3.98
Mtr.12725.1.S1_at	Mucin-like protein	-3.98
Mtr.7159.1.S1_at	Hypothetical protein	-3.98
Mtr.18014.1.S1_at	L-lactate dehydrogenase	-3.98
Mtr.37814.1.S1_at	CONSTANS-like protein	-3.97
Mtr.50712.1.S1_at	Hypothetical protein	-3.97
Mtr.10510.1.S1_s_at	Cytochrome b6f complex subunit	-3.97
Mtr.27444.1.S1_at	Hypothetical protein	-3.97
Mtr.40191.1.S1_at	Transcription factor EREBP-like protein	-3.97
Mtr.6728.1.S1_at	Cytochrome P450 protein	-3.96
Mtr.31537.1.S1_at	Hypothetical protein	-3.96
Mtr.50173.1.S1_at	Hypothetical protein	-3.96
Mtr.17362.1.S1_at	Beta-Ig-H3/fasciclin	-3.96
Mtr.35162.1.S1_at	Hypothetical protein	-3.96
Msa.1600.1.S1_at	Hypothetical protein	-3.96
Mtr.50484.1.S1_s_at	Peptidase M18	-3.96
Mtr.7553.1.S1_at	Alanine acetyl transferase	-3.95
Mtr.5862.1.S1_at	Photosystem II 22 kDa protein	-3.95
Mtr.42769.1.S1_at	Hypothetical protein	-3.95
Mtr.9315.1.S1_at	TRANSPARENT TESTA 12 protein	-3.95
Mtr.16385.1.S1_s_at	Hypothetical protein	-3.95
Mtr.25344.1.S1_at	Hypothetical protein	-3.95
Mtr.15048.1.S1_s_at	HAD-superfamily subfamily IIA hydrolase	-3.95
Mtr.35361.1.S1_at	Fructose-6-phosphate 2-kinase	-3.95
Mtr.42285.1.S1_at	Phosphoglycerate mutase	-3.95
Mtr.41311.1.S1_at	Hypothetical protein	-3.95
Mtr.34788.1.S1_at	Glyceraldehyde-3-phosphate dehydrogenase (NADP)	-3.94
Mtr.9586.1.S1_at	Hypothetical protein	-3.94
Mtr.12375.1.S1_at	Photosystem I reaction center subunit III	-3.94
Msa.1635.1.S1_at	Hypothetical protein	-3.94
Mtr.16346.1.S1_at	Hypothetical protein	-3.94
Mtr.9049.1.S1_at	Sbp65a protein	-3.94
Mtr.19730.1.S1_s_at	Microtubule-associated protein	-3.94
Mtr.7060.1.S1_at	Hypothetical protein	-3.94
Mtr.20212.1.S1_s_at	Aspartate/glutamate/uridylate kinase	-3.94
Mtr.38268.1.S1_at	Hypothetical protein	-3.93
Mtr.5449.1.S1_at	Hypothetical protein	-3.93
Mtr.5274.1.S1_s_at	Cellulose synthase	-3.93
Mtr.41476.1.S1_at	Cytochrome P450 71D11	-3.93
Mtr.47027.1.S1_at	Ubiquitin-conjugating enzyme	-3.92
Mtr.732.1.S1_s_at	Phosphoglucomutase	-3.92
Mtr.36687.1.S1_at	Hypothetical protein	-3.92

Mtr.29047.1.S1_at	Hypothetical protein	-3.92
Mtr.35868.1.S1_at	Hypothetical protein	-3.92
Mtr.25786.1.S1_at	Hypothetical protein	-3.92
Mtr.24495.1.S1_at	Photosystem II 32 kDa protein	-3.92
Mtr.2596.1.S1_at	Protein kinase	-3.91
Mtr.13516.1.S1_s_at	ZF-HD homeobox protein	-3.91
Mtr.45048.1.S1_s_at	Hypothetical protein	-3.91
Mtr.29878.1.S1_at	Hypothetical protein	-3.91
Mtr.7873.1.S1_at	Ribonuclease HII	-3.90
Mtr.42172.1.S1_s_at	Cytochrome P450	-3.90
Mtr.45399.1.S1_at	Hypothetical protein	-3.90
Mtr.50318.1.S1_at	Cys/Met metabolism pyridoxal-phosphate-dependent enzymes	-3.90
Mtr.26276.1.S1_at	CER1 protein	-3.90
Mtr.44644.1.S1_s_at	Hydrolase-like protein	-3.90
Mtr.36903.1.S1_at	Hypothetical protein	-3.90
Mtr.40844.1.S1_at	Diphosphonucleotide phosphatase 1	-3.90
Mtr.23750.1.S1_at	Hypothetical protein	-3.90
Mtr.42878.1.S1_at	Hypothetical protein	-3.90
Mtr.33436.1.S1_at	Hypothetical protein	-3.89
Mtr.40343.1.S1_s_at	Dihydrofolate reductas	-3.89
Mtr.2083.1.S1_s_at	Glutamate decarboxylase 1	-3.89
Mtr.25726.1.S1_s_at	Hypothetical protein	-3.88
Mtr.28571.1.S1_at	Phosphoribosylanthranilate transferase	-3.88
Msa.2726.1.S1_at	Hypothetical protein	-3.88
Mtr.22143.1.S1_at	Acetolactate synthase	-3.87
Mtr.11893.1.S1_at	4-Diphosphocytidyl-2C-methyl-D-erythritol synthase	-3.87
Mtr.20499.1.S1_at	Phosphatidylinositol-4-phosphate 5-kinase	-3.87
Mtr.44552.1.S1_at	Hypothetical protein	-3.87
Mtr.8532.1.S1_at	Alpha-expansin 3	-3.87
Mtr.24331.1.S1_s_at	Hypothetical protein	-3.86
Mtr.43421.1.S1_at	Hypothetical protein	-3.86
Mtr.17456.1.S1_at	Hypothetical protein	-3.86
Mtr.15141.1.S1_at	Hypothetical protein	-3.86
Mtr.17396.1.S1_s_at	Cellulose synthase	-3.86
Msa.1301.1.S1_at	Hypothetical protein	-3.86
Mtr.43528.1.S1_at	Fructokinase 3	-3.86
Mtr.33519.1.S1_at	Cold acclimation responsive protein	-3.86
Mtr.1806.1.S1_s_at	Hypothetical protein	-3.85
Mtr.4867.1.S1_s_at	Zygosaccharomyces rouxii pheromone response protein	-3.85
Mtr.36799.1.S1_s_at	Hypothetical protein	-3.85
Mtr.32655.1.S1_at	Hypothetical protein	-3.85
Mtr.27813.1.S1_at	6-4 photolyase	-3.85
Msa.1391.1.S1_at	Hypothetical protein	-3.85
Mtr.49013.1.S1_at	Hypothetical protein	-3.85
Mtr.13357.1.S1_at	EspB-like protein	-3.84
Mtr.6757.1.S1_at	Thaumatine-like protein	-3.84
Mtr.12787.1.S1_at	Membrane-associated 30 kDa protein	-3.84
Mtr.44288.1.S1_at	Hypothetical protein	-3.84
Mtr.7486.1.S1_at	Zinc finger protein	-3.84
Mtr.8707.1.S1_at	Hypothetical protein	-3.84
Mtr.41014.1.S1_at	Hypothetical protein	-3.84
Mtr.32192.1.S1_at	Hypothetical protein	-3.84
Mtr.41982.1.S1_at	Sulfate transporter ATST1	-3.83
Mtr.12978.1.S1_at	3-hydroxyisobutyrate dehydrogenase	-3.83

Mtr.26689.1.S1_at	Serine/threonine protein kinase	-3.83
Mtr.43458.1.S1_s_at	Hypothetical protein	-3.83
Mtr.4126.1.S1_at	Laccase	-3.83
Mtr.13194.1.S1_s_at	RanGAP1 interacting protein	-3.83
Mtr.44607.1.S1_at	Pyrroline-5-carboxylate synthetase 2	-3.83
Mtr.34726.1.S1_at	Photosystem I reaction center subunit II	-3.83
Mtr.10347.1.S1_at	Receptor-like protein kinase	-3.83
Mtr.36413.1.S1_at	Hypothetical protein	-3.82
Mtr.38367.1.S1_at	Hypothetical protein	-3.82
Mtr.4975.1.S1_s_at	SRG1 protein	-3.82
Mtr.44678.1.S1_at	Hypothetical protein	-3.82
Msa.1209.1.S1_at	Hypothetical protein	-3.82
Mtr.7840.1.S1_at	Hypothetical protein	-3.82
Mtr.51453.1.S1_at	Hypothetical protein	-3.81
Mtr.13051.1.S1_at	Glucose-6-phosphate 1-dehydrogenase 1	-3.81
Mtr.12218.1.S1_at	Cytochrome B6-F complex iron-sulfur subunit	-3.81
Mtr.45513.1.S1_at	Hypothetical protein	-3.80
Mtr.14002.1.S1_at	Calcium binding protein	-3.80
Mtr.25980.1.S1_s_at	Hypothetical protein	-3.80
Mtr.49776.1.S1_x_at	Auxin responsive SAUR protein	-3.80
Mtr.13136.1.S1_at	Fasciclin-like AGP 10	-3.80
Mtr.7841.1.S1_at	Hypothetical protein	-3.79
Mtr.22161.1.S1_at	Hypothetical protein	-3.79
Mtr.6710.1.S1_at	MYB transcription factor	-3.79
Mtr.39462.1.S1_at	Glu-tRNA(Gln) amidotransferase subunit A	-3.79
Mtr.39920.1.S1_at	Receptor like protein	-3.79
Mtr.29366.1.S1_at	Serine/threonine protein kinase	-3.79
Mtr.38303.1.S1_at	Hypothetical protein	-3.79
Mtr.39027.1.S1_at	Hypothetical protein	-3.78
Mtr.1744.1.S1_at	CRS2-associated factor 2	-3.78
Mtr.6773.1.S1_at	Myosin heavy chain-like protein	-3.78
Mtr.43598.1.S1_at	Polygalacturonase-like protein	-3.78
Mtr.4292.1.S1_at	Beta-galactosidase	-3.78
Mtr.8220.1.S1_at	Hypothetical protein	-3.78
Mtr.8775.1.S1_at	Hypothetical protein	-3.78
Msa.1594.1.S1_at	Hypothetical protein	-3.77
Mtr.9854.1.S1_at	Hypothetical protein	-3.77
Mtr.40816.1.S1_at	Thylakoid lumenal 15 kDa protein	-3.77
Mtr.24581.1.S1_s_at	1,2-diacylglycerol 3-beta-galactosyltransferase	-3.77
Mtr.32523.1.S1_at	Peptide transporter	-3.77
Mtr.43311.1.S1_at	BURP domain-containing protein	-3.77
Mtr.5622.1.S1_at	Hypothetical protein	-3.76
Mtr.15765.1.S1_at	Hypothetical protein	-3.76
Msa.1951.1.S1_s_at	Hypothetical protein	-3.76
Mtr.11444.1.S1_at	Receptor-like protein kinase	-3.76
Mtr.33858.1.S1_at	Metal-transporting P-type ATPase	-3.76
Mtr.33470.1.S1_at	Hypothetical protein	-3.76
Mtr.10814.1.S1_at	Epoxide hydrolase	-3.75
Mtr.42737.1.S1_at	Hypothetical protein	-3.75
Mtr.43288.1.S1_at	Diphenol oxidase	-3.75
Mtr.32700.1.S1_s_at	Hypothetical protein	-3.75
Mtr.35618.1.S1_at	Multi resistance protein	-3.75
Mtr.5133.1.S1_at	Hypothetical protein	-3.75
Mtr.42232.1.S1_at	Hypothetical protein	-3.75

Mtr.36799.1.S1_at	Hypothetical protein	-3.75
Mtr.18262.1.S1_at	Hypothetical protein	-3.75
Mtr.34410.1.S1_at	Hypothetical protein	-3.74
Mtr.39047.1.S1_at	RNA helicase-like protein	-3.74
Mtr.17229.1.S1_at	Na+/H+ antiporter-like protein	-3.74
Mtr.44265.1.S1_at	Hypothetical protein	-3.74
Mtr.10451.1.S1_at	Hypothetical protein	-3.74
Mtr.20815.1.S1_at	Hypothetical protein	-3.74
Mtr.46334.1.S1_at	Serine/threonine protein kinase	-3.74
Mtr.28691.1.S1_at	Nine-cis-epoxycarotenoid dioxygenase3	-3.74
Mtr.31196.1.S1_at	Hypothetical protein	-3.74
Mtr.16358.1.S1_at	Glyoxalase/extradiol ring-cleavage dioxygenase	-3.73
Mtr.10402.1.S1_at	Type II chlorophyll a/b binding protein from photosystem I	-3.73
Mtr.38643.1.S1_s_at	Sucrose-phosphate synthase	-3.73
Mtr.23112.1.S1_at	Chloride channel-like (CLC) protein	-3.73
Mtr.38333.1.S1_at	Hypothetical protein	-3.73
Mtr.4979.1.S1_at	Hypothetical protein	-3.73
Mtr.10428.1.S1_at	NADP-specific isocitrate dehydrogenase	-3.73
Mtr.43479.1.S1_at	Hypothetical protein	-3.73
Mtr.43131.1.S1_at	Endo-beta-1,4-glucanase	-3.72
Mtr.13309.1.S1_at	Hypothetical protein	-3.72
Mtr.9665.1.S1_at	Oikosin 4A protein	-3.72
Mtr.42216.1.S1_at	RanGAP1 interacting protein	-3.72
Mtr.17883.1.S1_at	Hypothetical protein	-3.72
Mtr.10665.1.S1_at	Hypothetical protein	-3.72
Mtr.1107.1.S1_at	Pectate lyase	-3.71
Mtr.31153.1.S1_at	Hypothetical protein	-3.71
Mtr.25499.1.S1_s_at	NifU-related metallocluster assembly factor	-3.71
Mtr.36224.1.S1_at	Hypothetical protein	-3.71
Mtr.31189.1.S1_at	Trehalose-6-phosphate phosphatase	-3.71
Msa.509.1.S1_at	Hypothetical protein	-3.71
Mtr.26982.1.S1_at	Hypothetical protein	-3.71
Mtr.21612.1.S1_at	Hypothetical protein	-3.71
Mtr.6279.1.S1_at	LIM domain containing protein	-3.71
Mtr.13288.1.S1_at	ADP-glucose pyrophosphorylase large subunit	-3.70
Mtr.12858.1.S1_at	Hypothetical protein	-3.70
Mtr.39136.1.S1_at	Hypothetical protein	-3.70
Mtr.38216.1.S1_at	Hypothetical protein	-3.70
Mtr.42457.1.S1_at	Hypothetical protein	-3.70
Mtr.5452.1.S1_at	ABC transporter	-3.70
Mtr.26358.1.S1_at	Hypothetical protein	-3.70
Mtr.10036.1.S1_at	Phosphate/phosphoenolpyruvate translocator	-3.69
Mtr.44112.1.S1_at	Uracil phosphoribosyltransferase	-3.69
Mtr.26277.1.S1_at	Lycopene beta-cyclase	-3.69
Mtr.15273.1.S1_at	Peptidase M14	-3.69
Mtr.48864.1.S1_at	Zn-finger, Dof type	-3.68
Mtr.13184.1.S1_at	Prolyl carboxypeptidase like protein	-3.68
Mtr.5291.1.S1_at	Hypothetical protein	-3.68
Mtr.28150.1.S1_at	Hypothetical protein	-3.68
Mtr.37287.1.S1_s_at	Beta-glucosidase	-3.68
Mtr.41882.1.S1_at	Hypothetical protein	-3.68
Mtr.5497.1.S1_s_at	Hypothetical protein	-3.68
Mtr.48836.1.S1_at	Hypothetical protein	-3.68
Mtr.39226.1.S1_at	MAP kinase	-3.67

Mtr.6733.1.S1_at	Rhomboid family protein	-3.67
Mtr.37084.1.S1_at	HSR203J protein	-3.67
Mtr.17822.1.S1_at	Cytochrome P450	-3.67
Mtr.45126.1.S1_at	Hypothetical protein	-3.67
Mtr.28642.1.S1_at	Hypothetical protein	-3.66
Mtr.29065.1.S1_at	GTP cyclohydrolase I	-3.66
Mtr.115.1.S1_at	Hypothetical protein	-3.66
Mtr.20947.1.S1_at	Serine/threonine protein kinase	-3.66
Mtr.16234.1.S1_at	Hypothetical protein	-3.66
Mtr.15646.1.S1_at	Hypothetical protein	-3.66
Mtr.26166.1.S1_at	Heat shock protein	-3.65
Mtr.8212.1.S1_at	Protein kinase	-3.65
Mtr.13236.1.S1_at	Glucose-6-phosphate dehydrogenase	-3.65
Mtr.44410.1.S1_at	RNA-binding protein FUS	-3.65
Mtr.20939.1.S1_at	Hypothetical protein	-3.65
Mtr.6532.1.S1_s_at	3-ketoacyl-ACP synthase	-3.65
Msa.1046.1.S1_at	Hypothetical protein	-3.65
Mtr.41314.1.S1_at	Pyruvate, phosphate dikinase	-3.64
Mtr.7496.1.S1_at	Hypothetical protein	-3.64
Mtr.8778.1.S1_at	Hypothetical protein	-3.64
Mtr.12200.1.S1_at	Hypothetical protein	-3.64
Mtr.3013.1.S1_at	UDP-glucuronosyltransferase	-3.64
Mtr.44368.1.S1_at	Hypothetical protein	-3.64
Mtr.756.1.S1_at	Hypothetical protein	-3.64
Mtr.37615.1.S1_at	Hypothetical protein	-3.64
Mtr.22763.1.S1_at	Hypothetical protein	-3.64
Mtr.9455.1.S1_at	Hypothetical protein	-3.64
Mtr.33398.1.S1_s_at	Hypothetical protein	-3.63
Mtr.23870.1.S1_at	Hypothetical protein	-3.63
Mtr.27067.1.S1_at	DNA binding with one finger 4 protein	-3.63
Mtr.44825.1.S1_at	POZ domain protein	-3.63
Mtr.5433.1.S1_at	Hypothetical protein	-3.63
Mtr.35648.1.S1_at	bHLH transcription factor	-3.63
Mtr.8614.1.S1_at	Mitochondrial solute carrier protein	-3.63
Mtr.20814.1.S1_at	Zn-finger, CCHC type	-3.62
Mtr.10888.1.S1_at	Hypothetical protein	-3.62
Msa.1349.1.S1_at	Hypothetical protein	-3.62
Mtr.38263.1.S1_at	Hypothetical protein	-3.62
Mtr.10826.1.S1_at	Rho GDP dissociation inhibitor 1	-3.62
Mtr.13656.1.S1_at	Galactomannan galactosyltransferase	-3.62
Mtr.10403.1.S1_at	Photosystem I reaction center subunit IV A	-3.62
Mtr.27391.1.S1_at	N-acylethanolamine amidohydrolase	-3.61
Mtr.39931.1.S1_at	Hypothetical protein	-3.61
Mtr.38311.1.S1_at	Hypothetical protein	-3.61
Mtr.48712.1.S1_s_at	Facilitated glucose transporter	-3.61
Mtr.16555.1.S1_s_at	Hypothetical protein	-3.61
Msa.3107.1.S1_at	Hypothetical protein	-3.61
Mtr.8790.1.S1_at	Peripheral-type benzodiazepine receptor	-3.61
Mtr.39691.1.S1_at	Hypothetical protein	-3.61
Mtr.39777.1.S1_at	Hypothetical protein	-3.60
Msa.2735.1.S1_at	Hypothetical protein	-3.60
Mtr.23994.1.S1_at	Homeodomain transcription	-3.60
Mtr.10506.1.S1_at	GH1 protein	-3.60
Mtr.12741.1.S1_at	Hypothetical protein	-3.60

Mtr.25499.1.S1_at	NifU-related metallocluster assembly factor	-3.60
Msa.2906.1.S1_at	Hypothetical protein	-3.60
Mtr.15378.1.S1_at	Hypothetical protein	-3.60
Mtr.27411.1.S1_s_at	Ribonucleoprotein	-3.60
Msa.2839.1.S1_at	Hypothetical protein	-3.59
Mtr.8418.1.S1_at	Xylulose kinase	-3.59
Mtr.13733.1.S1_at	Hypothetical protein	-3.59
Mtr.15083.1.S1_at	Pentaxin	-3.59
Mtr.29167.1.S1_s_at	Hypothetical protein	-3.59
Mtr.12617.1.S1_at	Hypothetical protein	-3.59
Mtr.42899.1.S1_a_at	Carboxylesterase	-3.59
Mtr.40827.1.S1_at	Hypothetical protein	-3.59
Mtr.44327.1.S1_at	Adenylate kinase 2	-3.58
Mtr.11200.1.S1_at	Zinc finger protein	-3.58
Msa.1509.1.S1_at	Hypothetical protein	-3.58
Msa.1866.1.S1_x_at	Hypothetical protein	-3.58
Mtr.31536.1.S1_at	Hypothetical protein	-3.58
Mtr.43478.1.S1_at	Serine carboxypeptidase	-3.58
Mtr.14183.1.S1_at	Annexin	-3.58
Mtr.20781.1.S1_at	Hypothetical protein	-3.58
Mtr.30783.1.S1_at	Cytochrome P450	-3.58
Mtr.18939.1.S1_at	Amino acid/polyamine transporter II	-3.57
Mtr.46001.1.S1_at	Hypothetical protein	-3.57
Mtr.33630.1.S1_at	Hypothetical protein	-3.57
Mtr.29098.1.S1_at	Long chain acyl-CoA synthetase 5	-3.57
Mtr.43980.1.S1_at	Endopeptidase	-3.57
Mtr.50700.1.S1_s_at	Serine/threonine protein kinase	-3.56
Mtr.37613.1.S1_at	Annexin-like protein	-3.56
Mtr.27110.1.S1_at	C2 domain-containing protein	-3.56
Mtr.12382.1.S1_at	Hypothetical protein	-3.56
Mtr.8973.1.S1_at	Hypothetical protein	-3.56
Mtr.40237.1.S1_at	Hypothetical protein	-3.56
Mtr.40341.1.S1_at	Dihydrofolate reductase	-3.56
Mtr.40906.1.S1_at	Quinone oxidoreductase	-3.56
Msa.3017.1.S1_at	Hypothetical protein	-3.55
Msa.1448.1.S1_at	Hypothetical protein	-3.55
Mtr.37317.1.S1_at	Plastocyanin	-3.55
Mtr.15596.1.S1_at	Hypothetical protein	-3.55
Mtr.18703.1.S1_at	Serine/threonine protein kinase	-3.55
Mtr.10229.1.S1_at	EspB-like protein	-3.55
Mtr.31183.1.S1_at	Long-chain acyl-CoA synthetase 6	-3.55
Mtr.6163.1.S1_at	Hypothetical protein	-3.55
Mtr.17550.1.S1_at	Plant lipid transfer protein	-3.54
Mtr.8509.1.S1_at	Hypothetical protein	-3.54
Mtr.2078.1.S1_at	Cell death regulator	-3.54
Mtr.44411.1.S1_at	Peptide methionine sulfoxide reductase msrB	-3.54
Mtr.15321.1.S1_at	Serine/threonine-specific protein phosphatase	-3.54
Mtr.32558.1.S1_at	N-acylethanolamine amidohydrolase	-3.54
Msa.1757.1.S1_at	Hypothetical protein	-3.54
Mtr.41803.1.S1_at	Hypothetical protein	-3.54
Mtr.10507.1.S1_s_at	GH1 protein	-3.54
Msa.3012.1.S1_at	Hypothetical protein	-3.54
Mtr.50427.1.S1_at	Lipoxygenase	-3.54
Mtr.2549.1.S1_at	Hypothetical protein	-3.53

Msa.1068.1.S1_at	Hypothetical protein	-3.53
Mtr.13662.1.S1_s_at	Protein kinase	-3.53
Msa.2703.1.S1_at	Hypothetical protein	-3.53
Mtr.37908.1.S1_at	Peroxidase	-3.53
Mtr.25541.1.S1_at	Hypothetical protein	-3.53
Mtr.13230.1.S1_at	Glycosyl transferase	-3.53
Mtr.32901.1.S1_at	Hypothetical protein	-3.53
Mtr.28673.1.S1_at	Hypothetical protein	-3.53
Mtr.28056.1.S1_at	Hypothetical protein	-3.53
Mtr.28405.1.S1_at	Negative transcription regulator	-3.53
Mtr.42999.1.S1_at	Photosystem I psaH protein	-3.52
Mtr.8663.1.S1_at	Calcium/calmodulin-dependent protein kinase CaMK3	-3.52
Mtr.8859.1.S1_at	Proline rich protein	-3.52
Mtr.15511.1.S1_at	Hypothetical protein	-3.52
Mtr.34694.1.S1_at	Hypothetical protein	-3.52
Mtr.12439.1.S1_at	50S ribosomal protein L27	-3.51
Mtr.15297.1.S1_at	Hypothetical protein	-3.51
Mtr.38636.1.S1_s_at	Hydrolase	-3.51
Msa.3135.1.S1_at	Hypothetical protein	-3.51
Mtr.37813.1.S1_s_at	Hypothetical protein	-3.51
Mtr.4282.1.S1_at	HAP3-like transcriptional-activator	-3.51
Mtr.16432.1.S1_at	Myb, DNA-binding	-3.51
Mtr.35464.1.S1_at	Triose phosphate/phosphate translocator	-3.51
Mtr.1068.1.S1_s_at	P-glycoprotein	-3.51
Mtr.12206.1.S1_at	Ribulose 1,5-bisphosphate carboxylase small subunit	-3.51
Mtr.45998.1.S1_at	Hypothetical protein	-3.51
Mtr.44526.1.S1_at	Hypothetical protein	-3.51
Mtr.46406.1.S1_at	Hypothetical protein	-3.51
Mtr.33116.1.S1_at	Receptor protein kinase	-3.50
Msa.1536.1.S1_at	Hypothetical protein	-3.50
Mtr.43358.1.S1_at	Outer membrane lipoprotein	-3.50
Mtr.39445.1.S1_at	Polygalacturonase isoenzyme 1 beta subunit	-3.50
Mtr.40583.1.S1_at	Polygalacturonase-like protein	-3.50
Mtr.2343.1.S1_at	Hypothetical protein	-3.50
Mtr.38045.1.S1_at	Fiber protein Fb19	-3.50
Mtr.19564.1.S1_s_at	Hypothetical protein	-3.49
Mtr.9004.1.S1_at	Hypothetical protein	-3.49
Mtr.9173.1.S1_at	Hypothetical protein	-3.49
Mtr.23748.1.S1_s_at	C3HC4-type zinc finger protein	-3.49
Mtr.11805.1.S1_at	Hypothetical protein	-3.49
Mtr.9599.1.S1_at	Hypothetical protein	-3.49
Msa.1612.1.S1_at	Hypothetical protein	-3.49
Mtr.1621.1.S1_at	Hypothetical protein	-3.49
Mtr.38739.1.S1_at	Receptor-like protein kinase	-3.49
Mtr.32736.1.S1_at	Hypothetical protein	-3.49
Mtr.44584.1.S1_at	Transfactor-like protein	-3.48
Mtr.35998.1.S1_at	cinnamoyl-CoA reductase	-3.48
Mtr.49327.1.S1_at	Hypothetical protein	-3.48
Mtr.40308.1.S1_at	Monofunctional aspartokinase	-3.48
Mtr.42201.1.S1_at	Hypothetical protein	-3.48
Mtr.2715.1.S1_s_at	Hypothetical protein	-3.48
Mtr.34418.1.S1_at	Hypothetical protein	-3.48
Mtr.34160.1.S1_at	Hypothetical protein	-3.48
Mtr.8457.1.S1_s_at	Hypothetical protein	-3.47

Mtr.30774.1.S1_at	Hypothetical protein	-3.46
Mtr.34395.1.S1_at	Hypothetical protein	-3.46
Mtr.10227.1.S1_at	Cytochrome P450	-3.46
Mtr.13443.1.S1_at	Hypothetical protein	-3.46
Mtr.10035.1.S1_at	Hypothetical protein	-3.46
Mtr.10662.1.S1_at	Heat shock 22 kDa protein	-3.46
Mtr.22314.1.S1_at	ATP citrate lyase b-subunit	-3.46
Mtr.12724.1.S1_at	PDI-like protein	-3.46
Mtr.42323.1.S1_at	Hypothetical protein	-3.46
Mtr.42188.1.S1_at	Yippee-like protein	-3.46
AFFX-Mtr-gsta-M_x_at	2,4-D inducible glutathione S-transferase	-3.46
Mtr.3440.1.S1_at	Hypothetical protein	-3.46
Mtr.10009.1.S1_at	Hypothetical protein	-3.45
Mtr.50826.1.S1_at	Beta-ketoacyl synthase	-3.45
Mtr.40922.1.S1_at	Hypothetical protein	-3.45
Mtr.39103.1.S1_at	Peptide deformylase	-3.45
Mtr.15836.1.S1_at	Cycloeucalenol cycloisomerase	-3.45
Msa.1725.1.S1_at	Hypothetical protein	-3.45
Mtr.42622.1.S1_s_at	Maturase	-3.45
Mtr.9328.1.S1_at	Carboxyl terminal protease	-3.45
Mtr.51837.1.S1_at	Thymidylate synthase	-3.45
Mtr.19465.1.S1_at	Thaumatin	-3.45
Mtr.10605.1.S1_at	Hypothetical protein	-3.45
Mtr.24463.1.S1_at	Hypothetical protein	-3.45
Mtr.38468.1.S1_at	Proline-rich protein	-3.44
Mtr.26216.1.S1_at	Auxin response factor 5	-3.44
Mtr.43423.1.S1_at	Hypothetical protein	-3.44
Mtr.39039.1.S1_at	Ankyrin like protein	-3.44
Mtr.9554.1.S1_at	Histone H4	-3.44
Mtr.17339.1.S1_at	50s ribosomal protein l18	-3.44
Mtr.8568.1.S1_at	Nitrite reductase	-3.44
Mtr.39964.1.S1_at	Hypothetical protein	-3.44
Mtr.712.1.S1_at	Thioredoxin	-3.44
Mtr.11023.1.S1_at	Ethylene-responsive family protein	-3.43
Mtr.25491.1.S1_at	Hypothetical protein	-3.43
Mtr.43439.1.S1_at	Hypothetical protein	-3.43
Mtr.12714.1.S1_at	Hypothetical protein	-3.43
Mtr.15596.1.S1_s_at	Hypothetical protein	-3.42
Mtr.769.1.S1_at	Harpin-induced protein 1 family (HIN1)	-3.42
Mtr.38670.1.S1_at	Homoserine kinase	-3.42
Mtr.34929.1.S1_at	Zinc finger protein	-3.42
Mtr.11058.1.S1_at	Snakin-1	-3.42
Mtr.2019.1.S1_at	Hypothetical protein	-3.41
Mtr.50467.1.S1_at	Serine/threonine protein kinase	-3.41
Msa.411.1.S1_at	Hypothetical protein	-3.41
Mtr.44047.1.S1_at	Hypothetical protein	-3.41
Mtr.9977.1.S1_at	Hypothetical protein	-3.41
Mtr.20484.1.S1_at	Cobalamin synthesis protein	-3.41
Mtr.44602.1.S1_at	PINHEAD protein	-3.41
Mtr.33344.1.S1_at	Disease resistance protein	-3.41
Mtr.15800.1.S1_s_at	Zn-binding protein	-3.40
Mtr.32741.1.S1_at	Nodulin-like protein	-3.40
Mtr.15660.1.S1_at	Peptidase M	-3.40
Mtr.32230.1.S1_at	Glycine-sarcosine methyltransferase	-3.40

Mtr.33453.1.S1_at	ATFP3	-3.40
Mtr.18482.1.S1_at	Hypothetical protein	-3.40
Mtr.12607.1.S1_at	Heat shock protein	-3.39
Mtr.27421.1.S1_s_at	NADPH HC toxin reductase	-3.39
Mtr.47145.1.S1_at	C2H2-type zinc finger protein	-3.39
Mtr.12270.1.S1_at	Alternative oxidase	-3.39
Mtr.42606.1.S1_at	Hypothetical protein	-3.39
Mtr.12080.1.S1_at	Hypothetical protein	-3.39
Mtr.38420.1.S1_at	Hypothetical protein	-3.39
Mtr.21401.1.S1_at	Hypothetical protein	-3.39
Mtr.11805.1.S1_s_at	Hypothetical protein	-3.39
Mtr.7251.1.S1_s_at	Beta-defensin prepropeptide	-3.39
Mtr.6658.1.S1_at	Auxin-induced protein	-3.39
Mtr.37387.1.S1_s_at	Inhibitor of apoptosis-like protein	-3.38
Mtr.26048.1.S1_at	Hypothetical protein	-3.38
Mtr.41201.1.S1_at	50S ribosomal protein L28	-3.38
Mtr.40447.1.S1_at	50S ribosomal protein L34	-3.38
Mtr.13068.1.S1_at	Amino acid transporter	-3.38
Msa.1262.1.S1_at	Hypothetical protein	-3.38
Mtr.11554.1.S1_at	Alpha-amylase	-3.38
Mtr.40192.1.S1_at	Transcription factor EREBP-like protein	-3.38
Mtr.32151.1.S1_at	Hypothetical protein	-3.38
Mtr.8916.1.S1_at	Hypothetical protein	-3.38
Msa.1840.1.S1_at	Hypothetical protein	-3.38
Mtr.35850.1.S1_at	Transcription factor WRKY44	-3.38
Msa.2547.1.S1_at	Hypothetical protein	-3.38
Mtr.40093.1.S1_at	Ribosomal protein L12.C	-3.38
Msa.1814.1.S1_at	Hypothetical protein	-3.37
Mtr.50620.1.S1_at	RimM protein	-3.37
Mtr.11974.1.S1_at	Hypothetical protein	-3.37
Mtr.49444.1.S1_at	Protease-associated PA	-3.37
Mtr.19693.1.S1_at	Zinc-containing alcohol dehydrogenase	-3.37
Mtr.2292.1.S1_s_at	Hypothetical protein	-3.37
Mtr.12113.1.S1_at	Adenylate isopentenyltransferase	-3.37
Mtr.14341.1.S1_at	Calponin-like actin-binding protein	-3.37
Mtr.34112.1.S1_at	Ferredoxin-thioredoxin reductase	-3.37
Mtr.9816.1.S1_at	Hypothetical protein	-3.37
Mtr.11399.1.S1_at	Proline-rich protein	-3.36
Mtr.39076.1.S1_at	Hypothetical protein	-3.36
Mtr.13691.1.S1_at	ABC transporter	-3.36
Mtr.35041.1.S1_s_at	Glutamate receptor 3.3	-3.36
Msa.1716.1.S1_at	Hypothetical protein	-3.36
Mtr.24561.1.S1_s_at	UDP-glycosyltransferase	-3.36
Mtr.29161.1.S1_at	Serine/threonine protein kinase	-3.36
Mtr.44469.1.S1_at	Prolyl carboxypeptidase like protein	-3.36
Msa.2828.1.S1_at	Hypothetical protein	-3.36
Mtr.37210.1.S1_at	Chlorophyll a-b binding protein 215	-3.36
Mtr.45403.1.S1_s_at	Cellulose synthase	-3.36
Mtr.43904.1.S1_at	Cyclase-like protein	-3.36
Mtr.1040.1.S1_at	Hypothetical protein	-3.36
Mtr.10790.1.S1_at	Lysosomal Pro-X carboxypeptidase	-3.36
Msa.2287.1.S1_at	Hypothetical protein	-3.36
Msa.1866.1.S1_at	Hypothetical protein	-3.36
Mtr.40090.1.S1_at	Malate dehydrogenase	-3.35

Mtr.43486.1.S1_at	70 kDa peptidylprolyl isomerase	-3.35
Mtr.37326.1.S1_at	Pectinesterase	-3.35
Msa.2778.1.S1_at	Hypothetical protein	-3.35
Mtr.43893.1.S1_at	Immunophilin	-3.35
Mtr.28734.1.S1_at	Hypothetical protein	-3.35
Mtr.38649.1.S1_at	Seed maturation-like protein	-3.35
Mtr.34698.1.S1_at	Phosphatidylinositol 4-phosphate 5-kinase	-3.35
Mtr.13626.1.S1_s_at	ZF-HD homeobox protein	-3.35
Msa.2966.1.S1_s_at	Hypothetical protein	-3.34
Mtr.18038.1.S1_at	Hypothetical protein	-3.34
Mtr.34832.1.S1_at	Hypothetical protein	-3.34
Mtr.41837.1.S1_s_at	Hypothetical protein	-3.34
Mtr.12001.1.S1_at	Phosphoribosylformimino-5-aminoimidazole carboxamide ribotide isomerase	-3.34
Mtr.27947.1.S1_at	Hypothetical protein	-3.34
Mtr.35486.1.S1_at	Hypothetical protein	-3.34
Mtr.12384.1.S1_at	Endo-beta-1,4-D-glucanase	-3.34
Mtr.27585.1.S1_at	GTP-binding protein	-3.34
Mtr.29292.1.S1_at	Hypothetical protein	-3.34
Mtr.42539.1.S1_at	Hypothetical protein	-3.34
Mtr.28009.1.S1_at	Hypothetical protein	-3.34
Mtr.43742.1.S1_at	bZIP protein BZO2H2	-3.34
Mtr.38897.1.S1_at	Pectinesterase PPE8B	-3.34
Mtr.41569.1.S1_at	Hypothetical protein	-3.34
Mtr.42228.1.S1_at	RNA-binding protein cp33	-3.34
Mtr.41016.1.S1_at	Hypothetical protein	-3.33
Mtr.21146.1.S1_at	Zn-finger, RING	-3.33
Mtr.51665.1.S1_s_at	Photosynthetic reaction centre protein	-3.33
Mtr.50907.1.S1_at	Glycoside hydrolase	-3.33
Msa.1045.1.S1_at	Hypothetical protein	-3.33
Mtr.2062.1.S1_at	Hypothetical protein	-3.33
Mtr.11306.1.S1_at	ATP-dependent RNA helicase	-3.33
Mtr.5919.1.S1_at	Hypothetical protein	-3.33
Mtr.7170.1.S1_at	Ribulose bisphosphate carboxylase small chain	-3.33
Msa.883.1.S1_at	Hypothetical protein	-3.33
Mtr.3793.1.S1_at	Hypothetical protein	-3.33
Mtr.39110.1.S1_at	Hypothetical protein	-3.32
Mtr.14599.1.S1_at	Hypothetical protein	-3.32
Mtr.13551.1.S1_at	mRNA cleavage factor subunit-like protein	-3.32
Mtr.28082.1.S1_s_at	Hypothetical protein	-3.32
Mtr.142.1.S1_at	LysM domain-containing receptor-like kinase 3	-3.32
Mtr.42024.1.S1_at	Hypothetical protein	-3.31
Mtr.12588.1.S1_at	Hypothetical protein	-3.31
Mtr.22065.1.S1_at	Hypothetical protein	-3.31
Mtr.11390.1.S1_at	Hypothetical protein	-3.31
Mtr.44412.1.S1_at	Hypothetical protein	-3.31
Mtr.36078.1.S1_at	Abscisic acid-induced-like protein	-3.31
Mtr.23551.1.S1_at	Late embryogenesis abundant protein D-34	-3.31
Mtr.32341.1.S1_s_at	Hypothetical protein	-3.30
Mtr.12491.1.S1_at	3-ketoacyl-acyl carrier protein reductase	-3.30
Mtr.13396.1.S1_at	Hypothetical protein	-3.30
Mtr.45994.1.S1_at	Hypothetical protein	-3.30
Mtr.22790.1.S1_at	UDP-glucose pyrophosphorylase	-3.30
Mtr.23638.1.S1_at	Hypothetical protein	-3.30
Mtr.11881.1.S1_at	Hypothetical protein	-3.30

Mtr.25700.1.S1_at	Hypothetical protein	-3.30
Mtr.32826.1.S1_at	Hypothetical protein	-3.30
Mtr.41949.1.S1_at	Hypothetical protein	-3.30
Mtr.42914.1.S1_at	Ribulose 1,5-bisphosphate carboxylase small subunit	-3.30
Mtr.16874.1.S1_at	Hypothetical protein	-3.29
Mtr.4733.1.S1_s_at	Peroxidase	-3.29
Mtr.10104.1.S1_at	Ornithine decarboxylase	-3.29
Mtr.19083.1.S1_at	ABC transporter	-3.29
Mtr.27521.1.S1_s_at	Hypothetical protein	-3.29
Mtr.27607.1.S1_at	Hypothetical protein	-3.29
Mtr.43790.1.S1_at	Ankyrin like protein	-3.29
Mtr.15800.1.S1_at	Zn-binding protein	-3.29
Mtr.10305.1.S1_at	Glyoxalase I	-3.29
Mtr.41104.1.S1_at	Hypothetical protein	-3.28
Mtr.11249.1.S1_s_at	Calcineurin B-like protein 10	-3.28
Mtr.11116.1.S1_at	Hypothetical protein	-3.28
Mtr.9451.1.S1_at	Hypothetical protein	-3.28
Mtr.40819.1.S1_at	Hypothetical protein	-3.28
Mtr.13163.1.S1_at	Hypothetical protein	-3.28
Mtr.34829.1.S1_at	Hypothetical protein	-3.28
Mtr.2592.1.S1_at	Disease resistance protein	-3.28
Mtr.2599.1.S1_at	Hypothetical protein	-3.28
Mtr.27398.1.S1_at	Hypothetical protein	-3.28
Mtr.44038.1.S1_at	Hypothetical protein	-3.28
Mtr.43384.1.S1_at	Hypothetical protein	-3.28
Mtr.10876.1.S1_at	Hypothetical protein	-3.28
Mtr.27941.1.S1_at	Hypothetical protein	-3.28
Mtr.51633.1.S1_at	Hypothetical protein	-3.27
Mtr.5084.1.S1_at	Hypothetical protein	-3.27
Mtr.44381.1.S1_at	Beta-galactosidase	-3.27
Msa.2619.1.S1_at	Hypothetical protein	-3.27
Mtr.51862.1.S1_at	Hypothetical protein	-3.27
Mtr.9594.1.S1_at	Hypothetical protein	-3.27
Mtr.26509.1.S1_s_at	ARP2/3 complex 20 kDa subunit	-3.27
Mtr.48199.1.S1_at	Hypothetical protein	-3.27
Mtr.38545.1.S1_s_at	Kinesin 1	-3.27
Mtr.7067.1.S1_at	Beta-galactosidase	-3.27
Mtr.11244.1.S1_at	RGA2 protein	-3.27
Mtr.13689.1.S1_at	Dehydration-responsive protein	-3.26
Mtr.45459.1.S1_at	Hypothetical protein	-3.26
Mtr.14184.1.S1_s_at	Annexin	-3.26
Mtr.19629.1.S1_at	Hypothetical protein	-3.26
Mtr.6638.1.S1_at	Hypothetical protein	-3.26
Mtr.26297.1.S1_at	Hypothetical protein	-3.26
Mtr.41205.1.S1_at	Mysoin-like protein	-3.26
Mtr.12012.1.S1_at	Phosphate starvation response regulator 1	-3.26
Mtr.5545.1.S1_at	Hypothetical protein	-3.26
Mtr.37882.1.S1_at	Nucleotide pyrophosphatase/phosphodiesterase	-3.26
Mtr.24749.1.S1_at	Hypothetical protein	-3.25
Mtr.33769.1.S1_s_at	Prolyl carboxypeptidase	-3.25
Mtr.17483.1.S1_s_at	Adenosine kinase	-3.25
Mtr.45240.1.S1_at	Hypothetical protein	-3.25
Mtr.12701.1.S1_at	Om(1E) protein	-3.25
Mtr.36865.1.S1_s_at	Hypothetical protein	-3.25

Mtr.11482.1.S1_at	Na+/H+ antiporter	-3.25
Msa.1148.1.S1_at	Hypothetical protein	-3.25
Mtr.17107.1.S1_at	Inositol monophosphatase	-3.25
Mtr.1770.1.S1_at	Protein kinase	-3.24
Mtr.10025.1.S1_at	Phosphoribosylanthranilate transferase	-3.24
Mtr.13326.1.S1_at	Hypothetical protein	-3.24
Mtr.38132.1.S1_a_at	Homeobox 2 protein	-3.24
Mtr.11994.1.S1_at	ABC transporter	-3.24
Mtr.37742.1.S1_s_at	Methionine sulfoxide reductase A	-3.24
Mtr.11318.1.S1_at	Hypothetical protein	-3.24
Mtr.25921.1.S1_at	PPR repeat-containing protein	-3.24
Msa.1230.1.S1_at	Hypothetical protein	-3.24
Mtr.8254.1.S1_at	Hypothetical protein	-3.24
Mtr.39543.1.S1_at	DNAJ heat shock N-terminal domain-containing protein	-3.24
Mtr.48366.1.S1_at	Hypothetical protein	-3.24
Mtr.32786.1.S1_at	Hypothetical protein	-3.23
Mtr.11197.1.S1_at	Hypothetical protein	-3.23
Mtr.29352.1.S1_at	Hypothetical protein	-3.23
Mtr.11205.1.S1_at	Hypothetical protein	-3.23
Mtr.8959.1.S1_at	Beta-expansin 3	-3.23
Mtr.152.1.S1_at	Hypothetical protein	-3.23
Msa.2683.1.S1_at	Hypothetical protein	-3.23
AFFX-Msa-gsta-M_x_at	Hypothetical protein	-3.23
Mtr.33655.1.S1_at	Hypothetical protein	-3.23
Mtr.4250.1.S1_at	Hypothetical protein	-3.23
Mtr.9069.1.S1_at	Myb family transcription factor	-3.23
Mtr.13091.1.S1_at	Loricrin-like protein	-3.22
Mtr.32341.1.S1_at	Hypothetical protein	-3.22
Mtr.45958.1.S1_s_at	Hypothetical protein	-3.22
Mtr.48305.1.S1_x_at	Hypothetical protein	-3.22
Mtr.26115.1.S1_at	Protein kinase	-3.22
Mtr.3083.1.S1_at	ABC transporter	-3.22
Mtr.9501.1.S1_at	Lysine decarboxylase	-3.22
Msa.2419.1.S1_at	Hypothetical protein	-3.22
Mtr.15386.1.S1_at	Hypothetical protein	-3.22
Mtr.44813.1.S1_at	Aromatic rich glycoprotein	-3.22
Mtr.4613.1.S1_at	4-methyl-5(B-hydroxyethyl)-thiazole monophosphate biosynthesis enzyme	-3.22
Mtr.41140.1.S1_at	Hypothetical protein	-3.22
Mtr.29108.1.S1_at	Hypothetical protein	-3.22
Mtr.11830.1.S1_at	ATP-dependent RNA helicase DBP9 (DEAD-box protein 9)	-3.21
Mtr.15494.1.S1_at	Cinnamyl alcohol dehydrogenase	-3.21
Mtr.38700.1.S1_at	Hypothetical protein	-3.21
Mtr.11631.1.S1_at	Chloroplast alpha-glucan water dikinase isoform 3	-3.21
Msa.1957.1.S1_s_at	Hypothetical protein	-3.21
Mtr.28655.1.S1_at	Hypothetical protein	-3.21
Mtr.41301.1.S1_at	Hypothetical protein	-3.21
Mtr.30682.1.S1_at	Hypothetical protein	-3.21
Mtr.500.1.S1_at	Hypothetical protein	-3.21
Mtr.3033.1.S1_at	Hydroquinone glucosyltransferase	-3.21
Mtr.41620.1.S1_at	Hypothetical protein	-3.21
Mtr.12509.1.S1_at	Hypothetical protein	-3.21
Mtr.14048.1.S1_s_at	Hypothetical protein	-3.20
Msa.897.1.S1_s_at	Hypothetical protein	-3.20
Mtr.45338.1.S1_at	Cytochrome P450	-3.20

Mtr.12798.1.S1_at	Benzoyl coenzyme A	-3.20
Mtr.19808.1.S1_at	Hypothetical protein	-3.20
Mtr.15549.1.S1_at	Hypothetical protein	-3.20
Mtr.46514.1.S1_s_at	Alpha/beta-amyrin synthase	-3.20
Mtr.24048.1.S1_at	Hypothetical protein	-3.20
Mtr.41480.1.S1_at	Polygalacturonase isoenzyme 1 beta subunit	-3.20
Mtr.11216.1.S1_at	Preprotein translocase secY subunit	-3.20
Mtr.12851.1.S1_at	Acyl CoA reductase	-3.20
Mtr.39201.1.S1_s_at	Hypothetical protein	-3.20
Mtr.6192.1.S1_at	Hypothetical protein	-3.20
Mtr.33457.1.S1_s_at	Galactinol synthase	-3.20
Mtr.27907.1.S1_at	Pectate lyase-like protein	-3.20
Msa.1595.1.S1_at	Hypothetical protein	-3.19
Mtr.29239.1.S1_at	Phosphatidylinositol-specific phospholipase C	-3.19
Mtr.36992.1.S1_at	Seed maturation-like protein	-3.19
Mtr.20364.1.S1_at	Annexin	-3.19
Mtr.9229.1.S1_at	Hypothetical protein	-3.19
Mtr.40199.1.S1_at	ARG10	-3.19
Msa.2803.1.S1_at	Hypothetical protein	-3.18
Mtr.33415.1.S1_at	Polygalacturonase inhibitor protein	-3.18
Mtr.51652.1.S1_at	Cytochrome P450	-3.18
Mtr.42368.1.S1_at	Hypothetical protein	-3.18
Mtr.32562.1.S1_at	Hypothetical protein	-3.18
Mtr.42200.1.S1_s_at	Protein kinase APK1B	-3.18
Mtr.37392.1.S1_at	Elongation factor Tu	-3.18
Mtr.36341.1.S1_s_at	Tetracycline transporter	-3.18
Mtr.28728.1.S1_at	Hypothetical protein	-3.18
Mtr.20633.1.S1_at	General substrate transporter	-3.18
Mtr.12473.1.S1_at	Isoflavonoid glucosyltransferase	-3.17
Mtr.10608.1.S1_at	Glutamine cyclotransferase	-3.17
Mtr.2580.1.S1_at	Leptin receptor	-3.17
Mtr.9332.1.S1_at	Hypothetical protein	-3.17
Mtr.41473.1.S1_at	Hypothetical protein	-3.17
Mtr.37602.1.S1_at	Proline-rich protein	-3.17
Msa.1246.1.S1_at	Hypothetical protein	-3.17
Mtr.44436.1.S1_at	Glutaredoxin-like protein	-3.17
Mtr.34636.1.S1_at	Hypothetical protein	-3.17
Mtr.31198.1.S1_at	Hypothetical protein	-3.16
Mtr.39941.1.S1_at	Hypothetical protein	-3.16
Mtr.22618.1.S1_at	Mismatch repair protein MutS	-3.16
Mtr.13868.1.S1_at	Hypothetical protein	-3.16
Mtr.5788.1.S1_s_at	Hypothetical protein	-3.16
Mtr.5333.1.S1_at	Hypothetical protein	-3.16
Mtr.12074.1.S1_s_at	Coronin binding protein	-3.16
Mtr.38661.1.S1_at	Hypothetical protein	-3.15
Msa.2549.1.S1_at	Hypothetical protein	-3.15
Mtr.35064.1.S1_at	Cucumisin-like serine protease	-3.15
Mtr.40618.1.S1_at	Thylakoid lumenal 35.8 kDa protein	-3.15
Mtr.19981.1.S1_at	Hypothetical protein	-3.15
Mtr.44708.1.S1_at	Hypothetical protein	-3.15
Mtr.10321.1.S1_at	Glycerophosphoryl diester phosphodiesterase 2	-3.15
Msa.1684.1.S1_at	Hypothetical protein	-3.15
Mtr.25480.1.S1_at	Mitochondrial carrier protein	-3.15
Mtr.8604.1.S1_at	Isoflavone reductase	-3.15

Mtr.40580.1.S1_at	30S ribosomal protein S17	-3.14
Mtr.13752.1.S1_at	Calcineurin B-like protein 10	-3.14
Mtr.32476.1.S1_at	Starch phosphorylase	-3.14
Mtr.28077.1.S1_at	Hypothetical protein	-3.14
Mtr.40407.1.S1_at	Hypothetical protein	-3.14
Mtr.40255.1.S1_at	Hydroquinone glucosyltransferase	-3.14
Mtr.44681.1.S1_at	Hypothetical protein	-3.14
Mtr.32541.1.S1_at	Xyloglucan endotransglucosylase/hydrolase	-3.14
Mtr.9050.1.S1_at	Hypothetical protein	-3.14
Mtr.32078.1.S1_at	Hypothetical protein	-3.14
Mtr.41534.1.S1_at	Hypothetical protein	-3.14
Mtr.17706.1.S1_at	Hypothetical protein	-3.14
Mtr.13141.1.S1_at	Chloroplast nucleoid DNA binding protein	-3.14
Mtr.41817.1.S1_at	Sulfate transporter ATST1	-3.14
Mtr.36188.1.S1_at	Hypothetical protein	-3.14
Mtr.41000.1.S1_at	Hypothetical protein	-3.14
Mtr.38498.1.S1_at	Mitochondrial transcription termination factor	-3.14
Mtr.34390.1.S1_at	Hypothetical protein	-3.13
Mtr.43838.1.S1_at	ATP-dependent Clp protease	-3.13
Mtr.11113.1.S1_at	Hypothetical protein	-3.13
Mtr.48466.1.S1_at	Uroporphyrinogen decarboxylase	-3.13
Mtr.42951.1.S1_at	Hypothetical protein	-3.13
Mtr.44312.1.S1_at	Hypothetical protein	-3.13
Mtr.12769.1.S1_at	Hypothetical protein	-3.13
Mtr.40397.1.S1_at	Cytochrome P450 78A3	-3.13
Mtr.50217.1.S1_s_at	AFG1-like ATPase	-3.13
Mtr.7379.1.S1_at	Hypothetical protein	-3.13
Mtr.32751.1.S1_at	Leucine-rich repeat receptor-like protein kinase 1	-3.13
Msa.2326.1.S1_at	Hypothetical protein	-3.13
Mtr.46616.1.S1_at	Hypothetical protein	-3.13
Mtr.41264.1.S1_at	Zinc finger protein 4	-3.13
Mtr.41768.1.S1_at	Verticillium wilt disease resistance protein	-3.12
Mtr.38127.1.S1_at	Hypothetical protein	-3.12
Mtr.43324.1.S1_s_at	Polygalacturonase-like protein	-3.12
Mtr.927.1.S1_at	Purple acid phosphatase	-3.12
Mtr.2090.1.S1_at	Cation transport ATPase	-3.12
Mtr.48713.1.S1_at	Ribosomal protein L10E	-3.12
Mtr.13889.1.S1_at	Hypothetical protein	-3.12
Mtr.33661.1.S1_at	Subtilisin-like serine proteinase	-3.12
Mtr.6340.1.S1_at	Cyclic nucleotide-gated ion channel 2	-3.12
Mtr.10771.1.S1_at	One helix protein	-3.12
Mtr.2197.1.S1_at	Hypothetical protein	-3.12
Mtr.12864.1.S1_at	Cytochrome P450	-3.12
Mtr.39924.1.S1_at	Ethylene-responsive family protein	-3.11
Mtr.3319.1.S1_s_at	Hypothetical protein	-3.11
Mtr.13460.1.S1_at	Chloroplast ribosomal protein L10	-3.11
Mtr.618.1.S1_s_at	Hypothetical protein	-3.11
Mtr.4373.1.S1_at	Hypothetical protein	-3.11
Mtr.41145.1.S1_at	Peroxisomal copper-containing amine oxidase	-3.10
Mtr.22822.1.S1_at	Hypothetical protein	-3.10
Mtr.37245.1.S1_s_at	Chlorophyll a-b binding protein 215	-3.10
Mtr.37989.1.S1_at	Hypothetical protein	-3.10
Mtr.17598.1.S1_at	Hypothetical protein	-3.10
Mtr.8824.1.S1_at	CONSTANS-like protein 2	-3.10

Mtr.34503.1.S1_at	Hypothetical protein	-3.10
Mtr.18775.1.S1_s_at	Hypothetical protein	-3.10
Mtr.17361.1.S1_at	Beta-Ig-H3/fasciclin	-3.10
Mtr.14047.1.S1_s_at	Hypothetical protein	-3.09
Mtr.12366.1.S1_s_at	Transcription factor Myb1	-3.09
Mtr.11702.1.S1_at	ABC transporter	-3.09
Mtr.42197.1.S1_at	Hypothetical protein	-3.09
Mtr.37704.1.S1_at	CDC48-interacting UBX-domain protein	-3.09
Mtr.24519.1.S1_at	Globulin-like protein	-3.09
Mtr.12637.1.S1_at	Red chlorophyll catabolite reductase	-3.09
Mtr.1559.1.S1_s_at	Calcineurin B-like protein 10	-3.09
Mtr.7204.1.S1_at	Microtubule-associated protein EB1-like protein	-3.09
Mtr.43182.1.S1_at	Hypothetical protein	-3.09
Mtr.39248.1.S1_at	Hypothetical protein	-3.09
Mtr.39913.1.S1_at	Hypothetical protein	-3.09
Mtr.33925.1.S1_at	Hypothetical protein	-3.08
Msa.1400.1.S1_at	Hypothetical protein	-3.08
Mtr.13554.1.S1_at	Hypothetical protein	-3.08
Mtr.9877.1.S1_at	Short-chain dehydrogenase/reductase	-3.08
Mtr.11441.1.S1_at	Aspartyl protease	-3.08
Mtr.42358.1.S1_at	Cysteine synthase	-3.08
Mtr.50947.1.S1_s_at	Myb, DNA-binding	-3.08
Mtr.41696.1.S1_at	Hypothetical protein	-3.08
Mtr.21515.1.S1_at	Cellulose synthase	-3.08
Msa.1941.1.S1_at	Hypothetical protein	-3.08
Mtr.14988.1.S1_at	Pectin lyase-like protein	-3.07
Mtr.9322.1.S1_at	Fatty acid elongase-like protein (Cer2-like)	-3.07
Mtr.18398.1.S1_at	Nodulin-like protein	-3.07
Mtr.39062.1.S1_at	Mitogen-activated protein kinase KSS1	-3.07
Mtr.34540.1.S1_s_at	Hypothetical protein	-3.07
Mtr.15922.1.S1_at	Chloroplast channel forming outer membrane protein	-3.07
Mtr.4623.1.S1_at	WRKY transcription factor 32	-3.07
Mtr.9912.1.S1_at	Hypothetical protein	-3.07
Mtr.31487.1.S1_at	Agmatine Iminohydrolase	-3.07
Msa.1570.1.S1_at	Hypothetical protein	-3.07
Mtr.13908.1.S1_at	HSR203J protein-like protein	-3.07
Mtr.18494.1.S1_at	ZF-HD homeobox protein	-3.06
Mtr.29383.1.S1_at	Hypothetical protein	-3.06
Msa.1219.1.S1_at	Hypothetical protein	-3.06
Mtr.32290.1.S1_at	Hypothetical protein	-3.06
Mtr.15412.1.S1_at	Hypothetical protein	-3.06
Mtr.8858.1.S1_at	Biogenesis protein	-3.06
Mtr.5543.1.S1_at	Multidrug resistance-associated protein	-3.06
Mtr.13011.1.S1_at	Receptor protein kinase PERK1	-3.06
Mtr.32315.1.S1_at	Hypothetical protein	-3.06
Msa.1697.1.S1_at	Hypothetical protein	-3.06
Mtr.7219.1.S1_at	Dehydration-responsive protein RD22	-3.06
Mtr.29302.1.S1_at	Hypothetical protein	-3.06
Mtr.5819.1.S1_at	Hypothetical protein	-3.05
Mtr.38018.1.S1_at	Ids4-like protein	-3.05
Mtr.1230.1.S1_s_at	Hypothetical protein	-3.05
Mtr.5795.1.S1_at	Hypothetical protein	-3.05
Mtr.1068.1.S1_at	P-glycoprotein	-3.05
Mtr.2138.1.S1_at	Hypothetical protein	-3.05

Mtr.33445.1.S1_at	Plakoglobin/armadillo/beta-catenin-like protein	-3.05
Mtr.11785.1.S1_at	Hypothetical protein	-3.05
Mtr.51674.1.S1_at	Mitochondrial carrier protein	-3.04
Mtr.50961.1.S1_s_at	Anthranilate phosphoribosyl transferase	-3.04
Mtr.50778.1.S1_at	Hypothetical protein	-3.04
Mtr.34709.1.S1_at	Ser-thr protein kinase	-3.04
Msa.2664.1.S1_at	Hypothetical protein	-3.04
Mtr.38668.1.S1_at	Hypothetical protein	-3.04
Mtr.20400.1.S1_at	Hypothetical protein	-3.04
Mtr.43815.1.S1_at	Hypothetical protein	-3.04
Mtr.9776.1.S1_at	Hypothetical protein	-3.04
Mtr.40612.1.S1_at	Myo-inositol monophosphatase 2	-3.04
Mtr.35867.1.S1_at	Hypothetical protein	-3.04
Mtr.23330.1.S1_at	Hypothetical protein	-3.03
Mtr.33737.1.S1_at	Hypothetical protein	-3.03
Mtr.23638.1.S1_s_at	Hypothetical protein	-3.03
Mtr.37859.1.S1_at	Patatin-like protein	-3.03
Mtr.12148.1.S1_s_at	Hypothetical protein	-3.03
Mtr.42622.1.S1_at	Maturase	-3.03
Mtr.48816.1.S1_at	Phosphoglycerate mutase	-3.03
Mtr.32155.1.S1_at	Hypothetical protein	-3.03
Mtr.43393.1.S1_at	Nicotianamine synthase	-3.03
Mtr.14808.1.S1_at	Nascent polypeptide-associated complex NAC	-3.02
Mtr.9216.1.S1_at	Aspartyl aminopeptidase	-3.02
Mtr.6308.1.S1_at	DnaK-type molecular chaperone hsc70	-3.02
Mtr.43133.1.S1_at	Triosephosphate isomerase	-3.02
Mtr.44307.1.S1_s_at	Hypothetical protein	-3.02
Mtr.8532.1.S1_s_at	Alpha-expansin 3	-3.02
Mtr.36189.1.S1_at	Hypothetical protein	-3.02
Mtr.29554.1.S1_s_at	Hypothetical protein	-3.02
Msa.2953.1.S1_at	Hypothetical protein	-3.02
Mtr.36426.1.S1_at	Hypothetical protein	-3.02
Msa.1443.1.S1_at	Hypothetical protein	-3.02
Mtr.14518.1.S1_at	HAD-superfamily hydrolase	-3.02
Mtr.8662.1.S1_at	Calcium/calmodulin-dependent protein kinase CaMK3	-3.02
Mtr.28518.1.S1_at	Hypothetical protein	-3.02
Mtr.37597.1.S1_s_at	Hypothetical protein	-3.02
Mtr.43643.1.S1_at	Sucrose-phosphatase	-3.02
Mtr.23930.1.S1_s_at	Crp1 protein	-3.02
Msa.1387.1.S1_at	Hypothetical protein	-3.01
Msa.1906.1.S1_at	Hypothetical protein	-3.01
Mtr.43274.1.S1_at	Gamma hydroxybutyrate dehydrogenase	-3.01
Mtr.42142.1.S1_at	GTP-binding protein	-3.01
Mtr.10483.1.S1_at	Esterase D	-3.01
Mtr.30224.1.S1_at	Hypothetical protein	-3.01
Mtr.11718.1.S1_at	Hypothetical protein	-3.01
Mtr.50053.1.S1_at	Hypothetical protein	-3.01
Mtr.37626.1.S1_at	Peroxidase	-3.00
Mtr.9768.1.S1_at	Hypothetical protein	-3.00
Mtr.20785.1.S1_at	Homoserine dehydrogenase	-3.00

Supplementary Table S5. Upregulated phytohormone related genes at 12 hrs after inoculation with soybean rust

Probesets	Target Description	Phytohormone	Fold reduction
Mtr.40147.1.S1_s_at	Class 10 PR protein	Salicylic acid	199.02
Mtr.10391.1.S1_at	Class 10 PR protein	Salicylic acid	78.99
Mtr.41478.1.S1_at	Class 10 PR protein	Salicylic acid	60.99
Mtr.42966.1.S1_at	Disease resistance response protein Pi49 (PR10)	Salicylic acid	52.33
Mtr.12615.1.S1_at	Pathogenesis-related protein PR10A	Salicylic acid	29.95
Mtr.22903.1.S1_at	Chitinase (Class II)	Salicylic acid	20.89
Mtr.33334.1.S1_at	Exo-1,3-beta-glucanase	Salicylic acid	13.20
Mtr.6341.1.S1_at	Beta-1, 3-glucanase	Salicylic acid	11.64
Mtr.4464.1.S1_at	Thaumatin-like protein 1	Salicylic acid	11.00
Mtr.8763.1.S1_at	Thaumatin-like protein PR-5a	Salicylic acid	10.06
Mtr.7638.1.S1_at	Endo-1,3-beta-glucanase	Salicylic acid	7.87
Mtr.12277.1.S1_at	Class 10 PR protein	Salicylic acid	7.49
Mtr.43119.1.S1_at	Chorismate synthase 1	Salicylic acid	7.09
Mtr.35960.1.S1_at	1, 3-beta-glucanase-like protein	Salicylic acid	5.89
Mtr.12525.1.S1_at	Chitinase	Salicylic acid	5.10
Mtr.8884.1.S1_at	Pathogenesis-related protein 4A	Salicylic acid	5.06
Mtr.35231.1.S1_s_at	Pathogenesis-related protein	Salicylic acid	4.44
Mtr.41245.1.S1_at	Chorismate mutase	Salicylic acid	4.44
Mtr.45398.1.S1_at	Exo-1,3-beta-glucanase	Salicylic acid	4.23
Mtr.10325.1.S1_at	Chitinase	Salicylic acid	3.95
Mtr.15053.1.S1_at	Thaumatin	Salicylic acid	3.94
Mtr.42147.1.S1_at	Intracellular pathogenesis related protein	Salicylic acid	3.67
Mtr.5770.1.S1_at	Endo-1,3-beta-glucanase	Salicylic acid	3.34
Mtr.8944.1.S1_at	Chorismate mutase	Salicylic acid	3.31
Mtr.9221.1.S1_at	Salicylic acid glucosyltransferase	Salicylic acid	3.23
Mtr.33283.1.S1_at	Chitinase	Salicylic acid	3.16
Mtr.35231.1.S1_at	Pathogenesis-related protein	Salicylic acid	3.13
Mtr.42989.1.S1_at	Thaumatin-like protein PR-5b	Salicylic acid	126.14
Mtr.48288.1.S1_s_at	Senescence-associated protein	Jasmonic acid	4.48
Mtr.318.1.S1_at	OPR3 (12-oxophytodienoic acid 10,11-reductase)	Jasmonic acid	4.41
Mtr.37633.1.S1_at	Allene oxide synthase	Jasmonic acid	4.07
Mtr.320.1.S1_at	12-oxophytodienoic acid 10, 11-reductase	Jasmonic acid	4.01
Mtr.320.1.S1_x_at	12-oxophytodienoic acid 10, 11-reductase	Jasmonic acid	3.62
Mtr.41703.1.S1_at	Lipoxygenase	Jasmonic acid	3.55
Mtr.32884.1.S1_s_at	Jasmonic acid 2	Jasmonic acid	3.17
Mtr.12917.1.S1_at	Jasmonic acid 2	Jasmonic acid	3.11
Mtr.8462.1.S1_at	Lipoxygenase	Jasmonic acid	3.07
Mtr.44496.1.S1_at	AP2 domain-containing protein AP29-like	Etlylene	168.51
Mtr.5385.1.S1_at	Ethylene reponse factor-like AP2 domain transcription factor	Etlylene	79.56
Mtr.23418.1.S1_s_at	AP2 domain transcription factor	Etlylene	78.36
Mtr.5750.1.S1_at	Nicotiana EREBP-3-like protein	Etlylene	47.69
Mtr.7260.1.S1_at	Ethylene-induced esterase	Etlylene	41.86
Mtr.32914.1.S1_at	Ethylene-induced esterase	Etlylene	25.61
Mtr.9223.1.S1_at	Ethylene reponse factor-like AP2 domain transcription factor	Etlylene	23.58
Mtr.41694.1.S1_s_at	AP2 domain transcription factor	Etlylene	21.86
Mtr.9788.1.S1_at	ACC synthase	Etlylene	15.31
Mtr.45912.1.S1_at	Pathogenesis-related transcriptional factor, ERF	Etlylene	13.53
Mtr.12456.1.S1_at	AP2-related transcription factor	Etlylene	11.61

Mtr.43497.1.S1_at	Ethylene-forming-enzyme-like dioxygenase	Etlylene	11.00
Mtr.4906.1.S1_s_at	1-aminocyclopropane-1-carboxylate oxidase (ACC oxidase)	Etlylene	6.57
Mtr.10165.1.S1_at	Ethylene receptor	Etlylene	6.41
Mtr.6378.1.S1_at	1-aminocyclopropanecarboxylic acid oxidase	Etlylene	4.71
Mtr.10424.1.S1_at	Ethylene responsive element binding factor	Etlylene	4.20
Mtr.39861.1.S1_at	Ethylene response factor ERF1	Etlylene	4.11
Mtr.39021.1.S1_at	AP2 domain transcription factor	Etlylene	4.02
Mtr.7556.1.S1_at	AP2 domain containing protein	Etlylene	3.98
Mtr.43104.1.S1_at	1-aminocyclopropanecarboxylic acid oxidase	Etlylene	3.82
Mtr.9002.1.S1_at	1-aminocyclopropane-1-carboxylic acid oxidase	Etlylene	3.54
Mtr.10425.1.S1_at	Ethylene responsive element binding factor	Etlylene	3.33
Mtr.13557.1.S1_at	Ethylene receptor homolog	Etlylene	3.32
Mtr.39020.1.S1_at	Ethylene receptor homolog	Etlylene	3.27
Mtr.42021.1.S1_at	1-aminocyclopropane-1-carboxylate oxidase	Etlylene	3.08
Mtr.27695.1.S1_at	Auxin response factor 30	Auxin	44.90
Mtr.38270.1.S1_at	ABA-responsive protein	Auxin	21.11
Mtr.35852.1.S1_at	Auxin-induced protein	Auxin	10.96
Mtr.35357.1.S1_at	Auxin-regulated gene	Auxin	10.10
Mtr.25476.1.S1_at	Auxin efflux carrier protein family	Auxin	9.31
Mtr.696.1.S1_at	Auxin-induced (indole-3-acetic acid induced) protein	Auxin	8.13
Mtr.49400.1.S1_at	Auxin responsive SAUR protein	Auxin	7.58
Mtr.10147.1.S1_at	Auxin-induced SAUR-like protein	Auxin	3.50
Mtr.13714.1.S1_at	Auxin-induced protein	Auxin	3.25
Mtr.12959.1.S1_s_at	Auxin response factor	Auxin	3.25
Mtr.44219.1.S1_at	Cytokinin oxidase-like protein	Cytokinin	8.86
Mtr.38799.1.S1_s_at	Cytokinin oxidase-like protein	Cytokinin	7.86
Mtr.38799.1.S1_at	Cytokinin oxidase-like protein	Cytokinin	6.06
Mtr.48768.1.S1_at	Gibberellin regulated protein	Gibberellin	38.44
Mtr.15448.1.S1_at	Gibberellin regulated protein	Gibberellin	5.14
Mtr.1887.1.S1_at	Gibberellin oxidase-like protein	Gibberellin	3.10

Supplementary Table S6. Downregulated phytohormone related genes at 12 hrs post inoculation with soybean rust

Probesets	Target Description	Pytohormone	Fold reduction
Mtr.39853.1.S1_at	Basic PR-1 protein	Salicylic acid	-46.12
Mtr.8977.1.S1_at	Pathogenesis-related protein	Salicylic acid	-37.07
Mtr.8572.1.S1_at	Thaumatin-like protein	Salicylic acid	-14.68
Mtr.7850.1.S1_s_at	Thaumatin-like protein	Salicylic acid	-10.98
Mtr.9686.1.S1_at	Beta-1,3-glucanase	Salicylic acid	-9.64
Mtr.30196.1.S1_at	S-adenosyl-L-methionine:salicylic acid carboxyl methyltransferase	Salicylic acid	-9.25
Mtr.9418.1.S1_s_at	Pathogenesis-related protein	Salicylic acid	-8.69
Mtr.19902.1.S1_at	Isochorismate synthases	Salicylic acid	-7.64
Mtr.6757.1.S1_at	Thaumatine-like protein	Salicylic acid	-6.25
Mtr.31555.1.S1_at	Beta-1,3-glucanase	Salicylic acid	-4.57
Mtr.43889.1.S1_at	Beta-1,3-glucanase	Salicylic acid	-4.41
Mtr.19007.1.S1_at	Barwin-related endoglucanase	Salicylic acid	-4.28
Mtr.10332.1.S1_at	Class Ib chitinase	Salicylic acid	-3.96
Mtr.40247.1.S1_at	Salicylic acid glucosyltransferase	Salicylic acid	-3.50
Mtr.8427.1.S1_at	Lipoxygenase	Jasmonic acid	-469.91
Mtr.52215.1.S1_at	Lipoxygenase	Jasmonic acid	-159.17
Mtr.6142.1.S1_at	Jasmonate O-methyltransferase	Jasmonic acid	-95.18
Mtr.50430.1.S1_at	Lipoxygenase	Jasmonic acid	-68.30
Mtr.5628.1.S1_s_at	Lipoxygenase	Jasmonic acid	-45.95
Mtr.50426.1.S1_at	Lipoxygenase	Jasmonic acid	-38.23
Mtr.13356.1.S1_at	Allene oxide cyclase C4	Jasmonic acid	-21.46
Mtr.40691.1.S1_at	Lipoxygenase	Jasmonic acid	-20.46
Mtr.11326.1.S1_at	Leaf senescence protein	Jasmonic acid	-20.38
Mtr.12720.1.S1_at	Lipoxygenase	Jasmonic acid	-16.43
Mtr.10593.1.S1_at	Allene oxide cyclase	Jasmonic acid	-14.25
Mtr.25367.1.S1_at	12-oxophytodienoic acid 10, 11-reductase	Jasmonic acid	-8.80
Mtr.15289.1.S1_at	Lipoxygenase	Jasmonic acid	-7.73
Mtr.8435.1.S1_at	Lipoxygenase	Jasmonic acid	-4.79
Mtr.29279.1.S1_at	Lipoxygenase	Jasmonic acid	-4.68
Mtr.13736.1.S1_at	Senescence-associated protein SAG102	Jasmonic acid	-3.53
Mtr.17317.1.S1_at	S-adenosyl-methionine-sterol-C-methyltransferase	Etlylene	-43.67
Mtr.34453.1.S1_at	1-aminocyclopropane-1-carboxylate synthase	Etlylene	-4.44
Mtr.28159.1.S1_at	AP2 domain transcription factor	Etlylene	-4.38
Mtr.42121.1.S1_at	1-aminocyclopropane-1-carboxylate synthase	Etlylene	-3.54
Mtr.11023.1.S1_at	Ethylene-responsive family protein	Etlylene	-3.11
Mtr.39924.1.S1_at	Ethylene-responsive family protein	Etlylene	-3.10
Mtr.25935.1.S1_at	Auxin-induced protein 15A	Auxin	-30.87
Mtr.25935.1.S1_x_at	Auxin-induced protein 15A	Auxin	-28.10
Mtr.49774.1.S1_at	Auxin responsive SAUR	Auxin	-16.68
Mtr.39011.1.S1_at	Auxin-binding protein	Auxin	-13.64
Mtr.49776.1.S1_x_at	Auxin responsive SAUR	Auxin	-5.14
Mtr.6658.1.S1_at	Auxin-induced protein	Auxin	-4.55
Mtr.19917.1.S1_at	Auxin responsive SAUR	Auxin	-3.44
Mtr.18240.1.S1_at	Auxin responsive SAUR protein	Auxin	-3.28
Mtr.26216.1.S1_at	Auxin response factor 5	Auxin	-3.19
Mtr.20619.1.S1_x_at	Auxin responsive SAUR	Auxin	-3.05
Mtr.28644.1.S1_at	Cytokinin receptor	Cytokinin	-10.36

Mtr.14157.1.S1_at	Gibberellin regulated protein	Gibberellin	-5.16

Supplementary Table S7. Upregulated phytohormone related genes at 24 hrs post inoculation with soybean rust

Probesets	Target Description	Phytohormone	Fold reduction
Mtr.40147.1.S1_s_at	Class 10 PR protein	Salicylic acid	212.17
Mtr.42989.1.S1_at	Thaumatin-like protein PR-5b	Salicylic acid	148.87
Mtr.331.1.S1_at	Chitinase	Salicylic acid	132.80
Mtr.10391.1.S1_at	Class 10 PR protein	Salicylic acid	98.15
Mtr.40555.1.S1_at	Osmotin-like protein	Salicylic acid	68.96
Mtr.43852.1.S1_at	Acidic glucanase	Salicylic acid	65.97
Mtr.8763.1.S1_at	Thaumatin-like protein PR-5a	Salicylic acid	53.55
Mtr.41478.1.S1_at	Class 10 PR protein	Salicylic acid	42.29
Mtr.45398.1.S1_at	Exo-1,3-beta-glucanase	Salicylic acid	21.29
Mtr.12615.1.S1_at	Pathogenesis-related protein PR10A	Salicylic acid	18.12
Mtr.12277.1.S1_at	Class 10 PR protein	Salicylic acid	17.56
Mtr.32629.1.S1_s_at	Thaumatin-like protein PR-5a	Salicylic acid	14.39
Mtr.8884.1.S1_at	Pathogenesis-related protein 4A	Salicylic acid	13.09
Mtr.12466.1.S1_at	Chitinase	Salicylic acid	11.91
Mtr.33334.1.S1_at	Exo-1,3-beta-glucanase	Salicylic acid	11.09
Mtr.35231.1.S1_s_at	Pathogenesis-related protein	Salicylic acid	8.62
Mtr.22903.1.S1_at	Chitinase (Class II)	Salicylic acid	8.09
Mtr.15053.1.S1_at	Thaumatin	Salicylic acid	7.67
Mtr.10325.1.S1_at	Chitinase	Salicylic acid	6.63
Mtr.35960.1.S1_at	1, 3-beta-glucanase	Salicylic acid	6.27
Mtr.9569.1.S1_at	Thaumatin-like protein 1	Salicylic acid	5.80
Mtr.35231.1.S1_at	Pathogenesis-related protein	Salicylic acid	5.66
Mtr.39139.1.S1_at	Pathogenesis-related protein 4A	Salicylic acid	4.87
Mtr.7638.1.S1_at	Endo-1,3-beta-glucanase	Salicylic acid	4.68
Mtr.12525.1.S1_at	Chitinase	Salicylic acid	4.60
Mtr.15054.1.S1_at	Thaumatin	Salicylic acid	4.60
Mtr.6341.1.S1_at	Beta-1, 3-glucanase	Salicylic acid	4.44
Mtr.10968.1.S1_at	Osmotin-like protein	Salicylic acid	4.34
Mtr.7638.1.S1_s_at	Endo-1,3-beta-glucanase	Salicylic acid	4.05
Mtr.41245.1.S1_at	Chorismate mutase CM2	Salicylic acid	3.71
Mtr.9221.1.S1_at	Salicylic acid glucosyltransferase	Salicylic acid	3.35
Mtr.35896.1.S1_at	Chitinase	Salicylic acid	3.26
Mtr.3520.1.S1_s_at	Exo-beta-glucanase	Salicylic acid	3.26
Mtr.39487.1.S1_s_at	Lipoxygenase	Jasmonic acid	3.88
Mtr.6540.1.S1_at	Lipoxygenase	Jasmonic acid	3.73
Mtr.20079.1.S1_at	Lipoxygenase	Jasmonic acid	3.65
Mtr.12917.1.S1_at	Jasmonic acid 2	Jasmonic acid	3.36
Mtr.13483.1.S1_at	13-lipoxygenase	Jasmonic acid	3.19
Mtr.32884.1.S1_s_at	Jasmonic acid 2	Jasmonic acid	3.14
Mtr.23418.1.S1_s_at	AP2 domain transcription factor	Etlylene	130.97
Mtr.42129.1.S1_at	AP2 domain transcription factor	Etlylene	69.26
Mtr.44496.1.S1_at	AP2 domain-containing protein	Etlylene	34.36
Mtr.7260.1.S1_at	Ethylene-induced esterase	Etlylene	27.08
Mtr.5385.1.S1_at	AP2 domain transcription factor	Etlylene	22.86
Mtr.9223.1.S1_at	AP2 domain transcription factor	Etlylene	16.36
Mtr.7556.1.S1_at	AP2 domain containing protein	Etlylene	9.36
Mtr.39267.1.S1_at	1-aminocyclopropane-1-carboxylate synthase	Etlylene	9.14
Mtr.45912.1.S1_at	Pathogenesis-related transcriptional factor and ERF	Etlylene	8.86

Mtr.4906.1.S1_s_at	1-aminocyclopropane-1-carboxylate oxidase	Etlylene	6.87
Mtr.43497.1.S1_at	Ethylene-forming-enzyme-like dioxygenase	Etlylene	6.62
Mtr.10165.1.S1_at	Ethylene receptor	Etlylene	5.89
Mtr.12456.1.S1_at	AP2-related transcription factor	Etlylene	5.43
Mtr.32914.1.S1_at	Ethylene-induced esterase	Etlylene	4.29
Mtr.5750.1.S1_at	EREBP-3-like protein	Etlylene	4.16
Mtr.43466.1.S1_at	Ethylene receptor	Etlylene	3.75
Mtr.10424.1.S1_at	Ethylene responsive element binding factor	Etlylene	3.54
Mtr.51291.1.S1_at	Pathogenesis-related transcriptional factor and ERF	Etlylene	3.52
Mtr.10425.1.S1_at	Ethylene responsive element binding factor	Etlylene	3.51
Mtr.39020.1.S1_at	Ethylene receptor homolog	Etlylene	3.23
Mtr.41694.1.S1_s_at	AP2 domain transcription factor	Etlylene	3.22
Mtr.35057.1.S1_at	1-aminocyclopropane-1-carboxylate synthase 1	Etlylene	3.10
Mtr.11873.1.S1_at	Ethylene-overproduction protein	Etlylene	3.09
Mtr.43104.1.S1_at	1-aminocyclopropanecarboxylic acid oxidase	Etlylene	3.07
Mtr.39368.1.S1_at	Ethylene receptor homolog	Etlylene	3.03
Mtr.13557.1.S1_at	Ethylene receptor homolog	Etlylene	3.01
Mtr.49400.1.S1_at	Auxin responsive SAUR protein	Auxin	15.16
Mtr.35852.1.S1_at	Auxin-induced protein	Auxin	12.47
Mtr.27695.1.S1_at	Auxin response factor	Auxin	11.68
Mtr.696.1.S1_at	Auxin-induced (indole-3-acetic acid induced) protein	Auxin	11.06
Mtr.25476.1.S1_at	Auxin efflux carrier protein	Auxin	8.01
Mtr.35357.1.S1_at	Auxin-regulated gene	Auxin	5.63
Mtr.10147.1.S1_at	Auxin-induced SAUR-like protein	Auxin	5.42
Mtr.49791.1.S1_at	Auxin responsive SAUR protein	Auxin	4.90
Mtr.49791.1.S1_x_at	Auxin responsive SAUR protein	Auxin	4.77
Mtr.13714.1.S1_at	Auxin-induced protein 22D	Auxin	3.64
Mtr.44219.1.S1_at	Cytokinin oxidase-like protein	Cytokinin	3.95
Mtr.38799.1.S1_s_at	Cytokinin oxidase	Cytokinin	3.55
Mtr.38799.1.S1_at	Cytokinin oxidase	Cytokinin	3.26
Mtr.48768.1.S1_at	Gibberellin regulated protein	Gibberellin	38.94
Mtr.15448.1.S1_at	Gibberellin regulated protein	Gibberellin	5.72
Mtr.18228.1.S1_at	Gibberellin regulated protein	Gibberellin	5.59

Supplementary Table S8. Downregulated phytohormone related genes at 24 hrs post inoculation with soybean rust

Probesets	Target Description	Phytohormone	Fold reduction
Mtr.39853.1.S1_at	Basic PR-1 protein	Salicylic acid	-47.85
Mtr.8977.1.S1_at	Pathogenesis-related protein 1	Salicylic acid	-27.66
Mtr.7850.1.S1_s_at	Thaumatin-like protein	Salicylic acid	-15.79
Mtr.9686.1.S1_at	Beta-1,3-glucanase	Salicylic acid	-14.00
Mtr.9418.1.S1_s_at	Pathogenesis-related protein	Salicylic acid	-11.85
Mtr.19902.1.S1_at	Isochorismate synthases	Salicylic acid	-11.55
Mtr.30196.1.S1_at	Salicylic acid carboxyl methyltransferase	Salicylic acid	-9.42
Mtr.43889.1.S1_at	Beta-1,3-glucanase-like protein	Salicylic acid	-6.36
Mtr.31555.1.S1_at	Beta-1,3-glucanase	Salicylic acid	-5.24
Mtr.8572.1.S1_at	Thaumatin-like protein	Salicylic acid	-4.84
Mtr.19007.1.S1_at	Barwin-related endoglucanase	Salicylic acid	-4.32
Mtr.6757.1.S1_at	Thaumatine-like protein	Salicylic acid	-3.84
Mtr.19465.1.S1_at	Thaumatin	Salicylic acid	-3.45
Mtr.8427.1.S1_at	Lipoxygenase	Jasmonic acid	-456.09
Mtr.50430.1.S1_at	Lipoxygenase	Jasmonic acid	-157.13
Mtr.52215.1.S1_at	Lipoxygenase	Jasmonic acid	-155.36
Mtr.5628.1.S1_s_at	Lipoxygenase	Jasmonic acid	-111.69
Mtr.6142.1.S1_at	Jasmonate O-methyltransferase	Jasmonic acid	-86.62
Mtr.50426.1.S1_at	Lipoxygenase	Jasmonic acid	-72.43
Mtr.40691.1.S1_at	Lipoxygenase	Jasmonic acid	-30.40
Mtr.12720.1.S1_at	Lipoxygenase	Jasmonic acid	-26.16
Mtr.10593.1.S1_at	Allene oxide cyclase	Jasmonic acid	-23.13
Mtr.11326.1.S1_at	Leaf senescence protein	Jasmonic acid	-22.46
Mtr.25367.1.S1_at	12-oxophytodienoic acid 10, 11-reductase	Jasmonic acid	-21.34
Mtr.13356.1.S1_at	Allene oxide cyclase C4	Jasmonic acid	-20.12
Mtr.15289.1.S1_at	Lipoxygenase	Jasmonic acid	-8.64
Mtr.13736.1.S1_at	Senescence-associated protein SAG102	Jasmonic acid	-5.29
Mtr.8435.1.S1_at	Lipoxygenase	Jasmonic acid	-5.26
Mtr.29279.1.S1_at	Lipoxygenase	Jasmonic acid	-5.20
Mtr.44182.1.S1_s_at	LEA protein	Jasmonic acid	-4.46
Mtr.50427.1.S1_at	Lipoxygenase	Jasmonic acid	-3.54
Mtr.17317.1.S1_at	S-adenosyl-methionine-sterol-C-methyltransferase	Etlylene	-50.99
Mtr.43282.1.S1_at	S-adenosyl-L-methionine: 2,7,4'-trihydroxyisoflavanone 4'-O-methyltransferase	Etlylene	-11.70
Mtr.42121.1.S1_at	1-aminocyclopropane-1-carboxylate synthase	Etlylene	-4.17
Mtr.28159.1.S1_at	AP2 domain transcription factor	Etlylene	-3.98
Mtr.11023.1.S1_at	Ethylene-responsive family protein	Etlylene	-3.43
Mtr.39924.1.S1_at	Ethylene-responsive family protein	Etlylene	-3.11
Mtr.25935.1.S1_at	Auxin-induced protein 15A	Auxin	-135.38
Mtr.25935.1.S1_x_at	Auxin-induced protein 15A	Auxin	-118.89
Mtr.49764.1.S1_at	Auxin Efflux Carrier	Auxin	-20.20
Mtr.39011.1.S1_at	Auxin-binding protein ABP19b	Auxin	-18.44
Mtr.26217.1.S1_at	Auxin response factor 5	Auxin	-5.24
Mtr.49774.1.S1_at	Auxin responsive SAUR protein	Auxin	-4.38
Mtr.49776.1.S1_x_at	Auxin responsive SAUR protein	Auxin	-3.80
Mtr.26216.1.S1_at	Auxin response factor 5	Auxin	-3.44
Mtr.6658.1.S1_at	Auxin-induced protein	Auxin	-3.39

Mtr.28644.1.S1_at	Cytokinin receptor, partial (26%)	Cytokinin	-7.53
Mtr.14157.1.S1_at	Gibberellin regulated protein	Gibberellin	-5.06

Supplementary Table S9. The expression of genes encoding enzymes for chlorophyll catabolism

Gene	Probesets	Ratio (ASR12/mock)	Ratio (ASR24/mock)
SGR	Mtr.40782.1.S1_at	3.56	6.53
NYC1	Mtr.43688.1.S1_at	0.61	3.14
CHL	Mtr.39762.1.S1_at	50.16	15.94
PAO	Mtr.50948.1.S1_at	1.08	3.23
RCCR	Mtr.12637.1.S1_at	0.36	0.33

Supplementary Table S10. List of primers used in this study

Gene	Probesets	FW primer	RV primer
Ubiquitin	Mtr.40698.1.S1_at	CTGACAGCCCACTGAATTGTGA	TTTTGGCATTGCTGCAAGC
PAL	Mtr.40166.1.S1_s_at	TCCACTTTCTTGATCGCGCTTTGC	ACTCCCTGTCGACAACTTTGAGCA
CHS	Mtr.40122.1.S1_s_at	CATGTGTGATAAATCTATGATCAA	CAGACAACCAACACACGAGCACCT
CHR	Mtr.17404.1.S1_s_at	GGAGATGAACCTTGCATGGCAACA	TGGTTGGTCCAGGGATCAAACGAT
CHI	Mtr.37413.1.S1_s_at	TCACTAGCTGCCAAATGGAAGGGT	GCAGCCAAACAGCGCTTAAGATCA
IFS	Mtr.11725.1.S1_at	ACCTGCTCAACGCCACCACTATTA	TGCTCGCCCTCTATGATTTCTCCA
HI4'OMT	Mtr.8652.1.S1_at	GAAGCTTTGAAGGAGGTGTGAGAG	AGCCATGGAACTAGGCATAGCTGA
2HID	Mtr.10466.1.S1_at	AGAACTTCCACCTCTTCTCCGTGT	AATCCCACCAGGTGCATCAGGATA
I2'H	Mtr.8605.1.S1_at	TGCAGCAGCTCCATTATTAGTGCC	AGCCCTTCTACCCAACCCAAATGA
IFR	Msa.2530.1.S1_s_at	ATGTCACTGAGGCTGATGTTGGGA	AGCAGTGGTGTATGTGACATCAGG
VR	Mtr.25021.1.S1_at	TGACTTCGCTGTGAGTGAACCAGA	GCGTCCACGAGTTTCTTCGTGTTT
NYC1	Mtr.43688.1.S1_at	AATATGGACGGTGCAGGTTCTGGA	AACGACTCGCATCCGTGGAACTAA
CHL	Mtr.39762.1.S1_at	TTACACCGACAGTGGAAGGCACAT	ACTTCACATGGTCCTGGCATAGGT
PAO	Mtr.50948.1.S1_at	GGGTTGCTCTTTGTTTGGCCTGAT	ACTGATCCGTGGGTTCCCTTCATT
RCCR	Mtr.12637.1.S1_at	CAACACTTGCTTCGAAACCGGTCA	TGATCCGAAGCAGTCCAACAATGC
SGR	Mtr.40782.1.S1_at	TTGCAAAGGGATGAAGTTGTGGCG	AGGTTGTGAAGCCTCCTTTAGTGG
PR3	Mtr.331.1.S1_at	GCTGCTTTCTTGGGTCAAAC	GTATGTGCTGCTGGGATTCT
PR4	Msa.3006.1.S1_at	GGCAAGTGCTTGAGGGTGACAAAT	ATCCAAGTTACGTGTAGCACCGCT
PR10	Mtr.34114.1.S1_s_at	AGAGTGTTGAAACCGTCGAGGGAA	TAGACCCTCCATTTGGGCCTTCAA
Hsr203J	Mtr.40659.1.S1_at	TTCCCATCCATCCTGGGTTTGTCA	AATGGCGGCAGTTTCAATCCATCG
